# GenoLIB: a database of biological parts derived from a library of common plasmid features

**DOI:** 10.1093/nar/gkv272

**Published:** 2015-04-29

**Authors:** Neil R. Adames, Mandy L. Wilson, Gang Fang, Matthew W. Lux, Benjamin S. Glick, Jean Peccoud

**Affiliations:** 1 Virginia Bioinformatics Institute, Virginia Tech, 1015 Life Science Circle, Blacksburg, VA 24061, USA; 2 School of Biological Technology, Xi'an University of Arts and Science, Xi'an, Shaanxi Province 710065, China; 3 Biosciences Division, Edgewood Chemical Biological Center, 5183 Blackhawk Rd Aberdeen Proving Grounds MD 21010, USA; 4 Molecular Genetics & Cell Biology, University of Chicago, 920 E. 58th St., Chicago, IL 60637, USA; 5 GSL Biotech LLC, 5211 S. Kenwood Ave., Chicago, IL 60615, USA

## Abstract

Synthetic biologists rely on databases of biological parts to design genetic devices and systems. The sequences and descriptions of genetic parts are often derived from features of previously described plasmids using ad hoc, error-prone and time-consuming curation processes because existing databases of plasmids and features are loosely organized. These databases often lack consistency in the way they identify and describe sequences. Furthermore, legacy bioinformatics file formats like GenBank do not provide enough information about the purpose of features. We have analyzed the annotations of a library of ∼2000 widely used plasmids to build a non-redundant database of plasmid features. We looked at the variability of plasmid features, their usage statistics and their distributions by feature type. We segmented the plasmid features by expression hosts. We derived a library of biological parts from the database of plasmid features. The library was formatted using the Synthetic Biology Open Language, an emerging standard developed to better organize libraries of genetic parts to facilitate synthetic biology workflows. As proof, the library was converted into GenoCAD grammar files to allow users to import and customize the library based on the needs of their research projects.

## INTRODUCTION

The concept of standard biological parts is central to synthetic biology. Biological parts are annotated DNA sequences that can be combined to make larger genetic systems (1,2). Initially, parts standardization focused on specific assembly strategies such as the BioBrick (3) or BglBrick (4) standards. Synthetic biologists have also recognized the need to use standard representations to describe parts. The Registry of Standard Biological Parts (www.partsregistry.org) supporting the iGEM competition was the first attempt at standardizing parts data (5). This pioneering experiment led to the idea of reporting data describing parts’ functions as standardized datasheets (6).

The most essential piece of information associated with a biological part is its sequence. Getting quality sequence information has proved more problematic than one might expect. For instance, an early review entitled ‘Genetic parts to program bacteria’ included hardly any references to sequence data (7). A frequent occurrence in the literature is defining constructs by names of standard promoters and CDSs, allowing the sequences to be deduced, but providing no information about other key sequences, such as 5′ non-coding regions. An assessment of the Registry of Standard Biological Parts uncovered several problems, such as missing part sequences or discrepancies between the published and physical sequences of biological parts (8). More generally, many journals have sequence disclosure policies, but these policies do not apply to plasmid sequences (9). A number of other reasons, such as tight budgets or limited access to a sequencing facility, also explain why so many plasmids described in the literature lack complete sequences. Repositories such as Addgene (10,11) aim to address this issue by documenting plasmids in their collections with sequence and annotation data, and also by associating plasmids with phenotype data, but such efforts have covered only a small fraction of the plasmids in published papers.

To overcome these limitations, we sought alternatives to peer-reviewed publications for obtaining quality sequence data that could provide a solid foundation for the development of a comprehensive database of genetic parts. Many cloning and expression vectors have been used for decades by molecular biologists. Companies and research consortia distributing these vectors typically provide detailed sequence information.

Developers of bioinformatics software have used annotations of plasmid sequences to develop automatic mapping capabilities. Using a list of features commonly found in plasmid sequences, the PlasMapper software was able to automatically annotate and generate maps of raw DNA sequences (12). This approach is now used by other bioinformatics packages including SnapGene (www.snapgene.com). However, each software team is developing its own database of common plasmid features using proprietary curation methods. As a result, it has not been possible to use an existing database of common plasmid features to develop a library of genetic parts.

We analyzed the features found in 1901 annotated sequence files with the goal of developing a database of plasmid features that supports unambiguous annotation of plasmid sequences. These features can be used as biological parts for synthetic biology with the aid of a sophisticated system of categories (13) compatible with the Synthetic Biology Open Language (SBOL) (14).

## MATERIALS AND METHODS

### Dataset

SnapGene is a commercial software to plan, visualize and document molecular biology procedures. The resources section of its web site includes a large library of annotated sequence files provided in the SnapGene proprietary format. We exported these files to GenBank format. This dataset was selected because it includes commonly used vectors for a wide array of applications. Furthermore, these sequences have been annotated using a combination of automatic and manual curation methods for accurate determination of feature sequences, borders and functional descriptions.

Since this library is frequently updated, it should be noted that this analysis was performed using files downloaded on 12 May 2014; at that point, the library had 1901 files grouped in 13 different collections (Supplementary Table S1). Duplicate plasmids and features were eliminated from this library as described in the Online Supplement and below. After elimination of duplicate files, 1718 distinct sequence files remained that included 1557 plasmid sequences and 161 single feature sequences (Table 1).

**Table 1.** Description of the different datasets

Dataset	Numbers	Comment
SnapGene File Library	1901 files	The entire collection of annotated sequence files available from the SnapGene web site.
Collections	13 collections	The different groups of files in the SnapGene File Library (Supplementary Table S1).
Non-Redundant File Library	1718 files	The SnapGene File Library after removal of duplicated sequences, feature orientation variants and topological variants of the same plasmid.
Non-Redundant Plasmid Library	1557 plasmid files	Subset of the Non-Redundant File Library after removal of single feature sequence files.
Feature Library	21 594 features	All the features extracted from the files in the Non-Redundant File Library.
Non-Redundant Feature Library	2046 features	Content of the Feature Library after removing duplicate features found in multiple files.
Standard Features Library	1943 features	Content of the Non-Redundant Feature Library after disambiguation of feature variants.
Expression Host	12 hosts	The SnapGene files are associated with 12 different expression hosts but some files do not include any host information.
SBOL Files	14 files	12 files corresponding to each of the expression hosts, 1 file for features with unspecified hosts, and a file with all the features.
GenoCAD Grammar	1967 parts 17 parts libraries 112 categories 67 rules	The GenoCAD grammar includes all the Standard Features to which we added 24 new parts not annotated in the SnapGene files, removed 14 CDS that differ only by the stop codon from other parts, and added 4 sequence delimiter parts. Parts are organized in 112 categories and
	17 libraries.	Finally, 67 rules define relations between categories.
	112 categories	
	67 rules	

### Database structure

We developed scripts to import the GenBank files into a MySQL database. The import scripts populated two tables. The *Plasmids* table captured the overarching summary information for each GenBank plasmid file, including the name, definition, sequence, application domain (category of plasmid, e.g. ‘Basic Cloning Vectors’ or ‘Insect Vectors’) and the entire text of the GenBank file in case we needed to go back and obtain additional information.

The *Features* table included the information available in the FEATURES block of each GenBank file; in addition to the location information (start, end and complement) and the feature key (named ‘feature_qualifier’ in our database), we also chose the first ‘note’ field within the FEATURE block for the name and, where present, the second ‘note’ field for the feature description.

The database includes a second set of tables to isolate sanitized data used in this analysis. The *standard_plasmids* table contains the plasmids after duplicates were resolved; we added a *standard_origins* lookup table that included a list of those application domains and a *standard_plasmid_origins* table that mapped the plasmids to their origins. The *standard_features* table contains the features after the duplicates were resolved; the *standard_feature_instances* table contains the feature keys and location for each feature within its associated plasmid, information that is needed to calculate statistical information regarding feature usage.

### Sequence alignments

Differences between feature variants were determined by multiple alignment of all variants of a particular feature. DNA and translated protein sequences were aligned in ClustalX 2.1 (15) using the default multiple alignment parameters. To find un-annotated promoter sequences, multiple alignments were performed using 300 bp of the 5′ non-coding region for a particular promoterless open reading frame (ORF) for all plasmids in which that promoterless ORF occurred. Many plasmids contained identical 300 bp 5′ non-coding sequences so only representative examples are shown in alignments (Supplemental File S1).

### Data availability

The data has been made available in various formats in the online supplement to the article.

## RESULTS

### Characterization of the plasmid library

**Figure 1.**
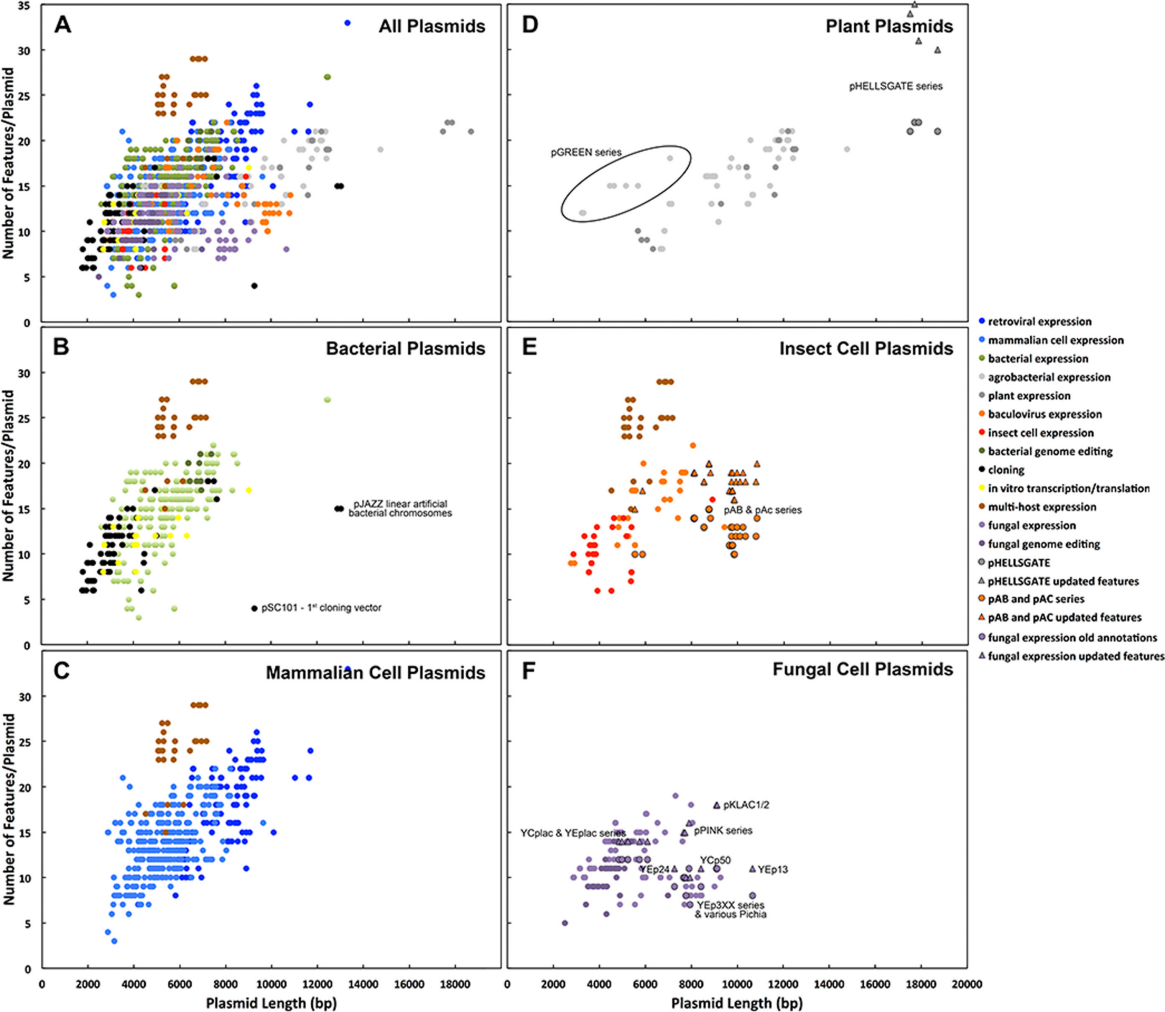
Correlation between plasmid length and number of features per plasmid. (**A**) All plasmids. Types of plasmid are indicated by color in the figure legends. Panels (**B**)–(**F**) are grouped by lab host with the specific type of plasmid indicated in color as in panel (A). Outliers with low or high feature densities are labeled. The outlined data points denote plasmids that had three or more additional features detected by SnapGene that were not annotated in the original downloaded files. The outlined circles show the original feature densities for these plasmids and the outlined triangles show the updated feature densities.

In order to get a better understanding of plasmid characteristics by application domain, we manually assigned the plasmids into different functional categories and host organisms. For each host organism, we generated scatter plots of plasmid lengths versus number of features subdivided by functional category (Figure 1). As expected, the number of features per plasmid tends to increase with plasmid length. Most of the plasmids cluster in the 2–10 kb size range with 5–25 features per plasmid. Unsurprisingly, plasmids for higher order hosts (mammalian, insect) were larger than simpler hosts (bacteria, fungus). Similarly, multi-hosts plasmids and retroviral vectors tend to have more features than other categories of plasmids. In general, it appears that most plasmids have a large amount of extraneous and apparently non-functional sequence and could be made more compact by designing the plasmids with shorter functional features. Good examples of efficient design are the pGREEN plasmids for expression in plant cells, and the multi-host expression plasmids in the pTriEx and pQE-TriSystem series (Figure 1).

### Resolving duplicate and inconsistent features

After the initial extraction of features from the files in the Non-Redundant File Library, there were 21 594 features in our dataset. Because many features are used across multiple plasmids, this first raw set of plasmid features included duplicates and inconsistent sequences that were not appropriate for our Standard Features Library. The steps we took to refine the data are described below:

- First, we queried the database to find perfect duplicate features, or those with the same sequence, name and description. We included only one copy of such a feature in the Standard Features Library while keeping track of all the instances of this feature in the SnapGene Plasmid Library. This step reduced our initial dataset from 21 594 features to 2046.
- Next, we removed all features that we flagged as ‘inconsistent’. This would include features with sequences that contained characters other than a, t, g and c (for example, n, h, d, w and y) because these features are too ambiguous to be included in a database of standard features. Similarly, we eliminated CDS features with joined locations corresponding to introns and exons because these features add a new level of complexity not well supported by automated mapping algorithms. This reduced the remaining feature set from 2046 to 2036.
- We also considered the case of features with the same sequence but different names in the raw feature set. In this case, we included the feature with the most commonly used name in the Standard Features Library while including the name variants as synonyms in a separate field. This step reduced the feature set from 2036 to 1994 features. We also noted a couple of cases where the sequences were the same, but the names included some position information. HIV-1 5′ LTR and HIV-1 3′ LTR have the same sequence. We eliminated the duplicate and renamed this feature HIV-1 LTR. We did the same operation for the truncated version of this feature.
- Then, we considered the case where the name and sequence were the same, but the description was different. As in the case of different names for the same sequence, we selected the most commonly used description for that feature. This step reduced the feature set from 1994 to 1943.
- Finally, we looked for features having the same names but different sequences. This situation corresponds to sequence variants calling for disambiguation of the feature name. Hence, we indexed the different feature variants when including them in the Standard Features Library by adding a number after the name, as in MCS-001, MCS-002, MCS-003, etc. In addition, there were four features that did not have any name or description; those were examined and manually named. These steps had no impact on the number of features, but 1518 features had adjustments to their names.

After all the duplicates had been eliminated, the Standard Features Library included 1943 features.

### Statistical analysis of standard features library

#### Usage statistics

We examined the frequency with which each feature occurred in the plasmid library (Supplementary Figure S2, top). Surprisingly, 766 features (∼40%) appeared only once in the plasmid set, but most of them (448) are variants of more common features. A number of fluorescent proteins that were imported from single-feature files are also in this situation because they are not used in any of the plasmids. At the other end of the distribution, 13 features were used more than 200 times in the Non-Redundant File Library (Supplementary Table S2). This set includes features required for plasmid propagation in *Escherichia coli* (antibiotic resistance, origins of replication), sequencing primer sites, and prokaryotic and mammalian promoters.

Many important common features have multiple variants. For instance, AmpR promoter-009 occurs in 967 (62%) of the plasmids, but there are 12 AmpR promoter variants that occur in 1110 (71%) of the plasmids. In some cases, one of the variants is used much more frequently than any other, but in other cases different variants have substantial usage statistics (discussed below).

The variability of feature sequences may result from annotation errors, errors in the plasmid sequences or from mutations—deliberate or not (Table 2). For instance, codon optimization can be a source of sequence variability at the DNA level. To evaluate the possible contribution of annotation and sequence errors to the overall variability of feature sequences, we partitioned the feature database into variable features versus conserved features having no variants. We found 272 variable features and 432 conserved features (Supplemental File S1). Only six of the conserved features are present in more than 100 plasmids (T7 promoter, ATG, M13 rev, M13 fwd, lacI promoter and EM7 promoters). Apart from the lacI promoter, these features do not appear under a different name in the list of variable features. This observation shows that, at least in the case of well-defined features with short sequences, the process used to edit and annotate sequences is robust enough to prevent the introduction of spurious errors.

**Table 2.** Statistics for non-coding and protein coding feature variants

Feature	No. of variants^a^	No. of occurrences	No. of bp changes^b^	Total length (bp)	Changes/ Variant	Changes/ 1000 bp	Length only variants^c^
Non-coding features							
AmpR promoter	12	1110	12	1154	1.0	10.4	3 (25.0%)
CMV enhancer	15	519	15	4954	1.0	3.0	5 (35.7%)
CMV promoter	10	511	29	2039	2.9	14.2	3 (30.0%)
SV40 promoter^d^	23	897	28	4613	1.2	6.1	7 (30.4%)
f1/M13 ori	22	651	85	9773	3.9	8.7	3 (13.6%)
ori	22	1490	48	12 689	2.2	3.8	2 (9.1%)
IRES	16	82	21	8767	1.3	2.4	4 (25.0%)
Total	120	5260	238	43 989	2.0	5.4	27 (22.5%)
**Mean/Feature**	**17**	**751**	**34**	**6284**			

Coding features							
AmpR/bla(M)	23	1065	161	19 734	7.0	8.2	4 (17.4%)
CmR	16	211	25	10 605	1.6	2.4	1 (6.3%)
HygR	14	101	282	14 376	20.1	19.6	3 (21.4%)
KanR	19	119	131	15 474	6.9	8.5	0 (0.0%)
NeoR/KanR	23	354	66	18 312	2.9	3.6	2 (8.7%)
PuroR	11	75	131	6627	11.9	19.8	0 (0.0%)
lacZ-α	74	144	14	27 102	0.2	0.5	70 (95.0%)
MBP	10	36	40	11 022	4.0	3.6	1 (10.0%)
Total^e^	116	1961	836	96 150	7.2	8.7	11 (9.5%)
	190	2105	850	123 152	4.5	6.9	81 (42.6%)
**Mean/Feature**	**17**	**263**	**119**	**13 736**			
		
	24	280	106	15 394			

^a^After consolidation of identical features upon correction of sequence or annotation errors.

^b^Base pair changes relative to the consensus sequence, including missense mutations and indels, but excluding differences in feature borders.

^c^Variants that differ from the consensus only by their borders. It does not include variants missing only START or STOP codons.

^d^Includes all variants of SV40 ori, SV40 enhancer and SV40 promoter.

^e^Values in bold exclude lacZ-α variants as the majority of these differ only in their in-frame multiple cloning sites.

#### Analysis of feature variants

We examined the common features that had 10 or more variants to identify the sources of this variability. We performed sequence alignments of the feature variant sequences and the translation products for coding regions. The variants were either pure length variants in which only the borders of the feature differed, or pure sequence variants that had the same borders as the consensus feature, but contained mismatches or indels or a mix of both. Usually, the most used variant matches the consensus sequence. Interestingly, many of the variants were specific to plasmids from a single source or supplier, even when there were dozens of instances of the variant (Supplemental File S1).

Supplementary Figure S3 shows the usage distributions for features that have 10 or more variants. Variant usage for non-coding features such as enhancers, promoters and origins of replication tends to be conservative, with one or two variants dominating the number of instances, and a large proportion of the non-coding feature variants differing only in their borders (Table 2). The exceptions were the IRES (internal ribosome entry site), which showed more even use of the variants (Supplementary Figure S3). However, most of the IRES variants are functionally distinct (Supplemental File S1).

In contrast to most non-coding features, variants of protein coding features were more broadly used, and few of the coding variants differed in length (Supplementary Figure S3). Instead, these features displayed a high level of sequence variation (Table 2). Nevertheless, the majority of sequence changes were synonymous codon changes and many of the variants encoded identical translation products (Table 3).

**Table 3.** Types of variations in protein coding features

Feature	Synonymous codon changes^a^	Conservative residue changes^a^	Non-conservative residue changes^a^	Variants no aa changes^b^
AmpR/bla(M)	73%	17%	10%	39%
CmR	84%	16%	0%	81%
HygR	87%	1%	12%	57%
KanR	54%	6%	40%	32%
NeoR/KanR	62%	14%	24%	65%
PuroR	94%	1%	5%	73%
lacZ-α^c^	93%	7%	0%	95%
MBP	27%	2%	71%	60%
Total no.	641	54	156	134
Total Mean	75%	7%	18%	71%

^a^Percentage of all bp changes including mismatches and indels but excluding border differences.

^b^Percentage of variants that produce no changes in the translated protein.

^c^Excluding the multiple cloning sites.

In contrast to marker genes, affinity tag variants such as MBP and GST differed mostly in whether they included START/STOP codons or in-frame extensions such as linkers or MCS sites (Supplemental File S1), but epitope tag variants such as HA and Myc were uniform in length and rife with synonymous codon changes, usually as a result of codon optimization (16,17). Affinity tags are almost exclusively used for bacterial expression and protein purification, while the epitope tags are used in a variety of host cells for immunoprecipitation and immunofluorescence, and therefore require codon optimization for each host. More details on feature variants are provided in the Online Supplement.

#### Feature sequence length

The sequence length for the features was highly variable, with the shortest features (pUC sequence origin and splice donor mutation) coming in at 1 bp and the longest (adenoviral DNA) at 30 549 bp, with a median sequence length of 267 bp. The statistical distribution of feature sequence lengths is bimodal (Supplementary Figure S2, bottom). The majority of features have sequences shorter than 120 bp. Another peak centered around 700 bp consists mostly of coding sequences.

#### Number of features in each feature qualifier

Of the 63 GenBank feature keys currently available from the International Nucleotide Sequence Database Collaboration http://www.insdc.org/documents/feature-table, 25 were represented in the SnapGene Plasmid Library. Plotting the distribution of features according to feature keys shows that the vast majority (71%) falls in only two categories (Supplementary Figure S4). CDS was used the most often (867 times) followed by MISC_FEATURE (515 times). The over-representation of two categories is an indication that the GenBank feature keys do not have the resolution necessary to represent plasmid features. For instance, purification tags are annotated as CDS, but should be identified as tags. Similarly, one could argue that sequences coding for fluorescent proteins should be distinguished from other coding sequences, and multiple cloning sites or stop codons/signals are common enough to justify identifying them with new feature keys.

### Segmentation by expression host

Some features are host-specific. For example, promoters are often specific to an expression host. Other features, such as coding sequences and structural elements allowing the propagation of a plasmid in *E. coli*, can be used in shuttle plasmids for a number of different hosts. We looked at the expression host specified in the GenBank files of the plasmids. After some cleanup to address inconsistent spelling of the hosts, we found 12 different hosts represented in this dataset, 13 if including those where the host was unspecified; this list of lab hosts includes *E. coli*, Mammalian Cells, *Bacillus subtilis*, Gram-negative bacteria, *Drosophila melanogaster, Saccharomyces cerevisiae*, Insect Cells, Plant Cells, *Schizosaccharomyces pombe, Pichia pastoris, Aspergillus nidulans, Kluyveromyces lactis* and Unspecified. We then associated each feature with their expression hosts by querying the features’ hosts from their related plasmids. Most of the features (1629) were associated with only one lab host (and, of those, 139 were associated only with Unspecified hosts). One hundred seventy-five features were associated with more than one lab host, and 21 of those were associated with five or more lab hosts.

### Development of a library of biological parts

The SBOL is a community-driven standard for exchanging synthetic biology data between applications (14). In order to generate SBOL files of the Standard Features Library, we first developed a short Java program that could read the contents of a flat file that we could generate from the database. This program relied on the libSBOLj library (https://github.com/SynBioDex/libSBOLj) to reformat that information and output the features as collections of parts (DnaComponents). One challenge for this approach is that the features were categorized using the GenBank feature keys, but SBOL relies on the Sequence Ontology (SO) to categorize its parts (18,19). BioPerl provides a script for translating GenBank Feature Keys to SO identifiers that we used for developing the mapping table reported in Supplementary Table S3. The flat file output included a display_id, the feature name, the description, the sequence and the SO identifier corresponding to the associated GenBank feature key.

We generated an SBOL file for each lab host, and one for the parts with the Unspecified host. Finally, we generated a file that includes the collections for all of the hosts (Supplemental File S4).

### Development of a GenoCAD grammar

In order to facilitate the use of these standard features as genetic parts, we edited the features database and imported it into GenoCAD, a computer assisted design application for synthetic biology (20,21). We used GenoCAD to edit the database of genetic parts by adding new parts, defining new categories of parts and rules (22) describing relations between part categories, and finally organizing the parts in different libraries as previously described (23). The grammar is available online as Supplemental File S5.

#### Removal of START and STOP codons

Many of the features annotated as CDS included coding sequences such epitope tags or fluorescent protein domains that could be used in fusion with other coding sequences. In order to facilitate the combination of coding sequences, we removed the start and stop codons found at the extremities of CDS features. After removal of these codons, 14 CDS feature variants were identical to other variants and were merged with them. We introduced START and STOP codons as separate parts in the database.

#### Part categorization

The GenBank qualifiers do not provide the resolution necessary to properly describe the function of genetic parts and organize a large library accordingly. As a result, we recategorized the parts library using a custom categorization system that relies as much as possible on existing SO terms. In some cases, we took advantage of commonly used terms that may not yet be part of the SO. The specification of each category includes a long category name and a short category code. The category description includes a reference to the corresponding SO terms along with the SO definition when applicable. The names of categories without a corresponding SO term start with a + in order to facilitate their identification. Each category is mapped to a GenBank feature key (Supplementary Table S3). Finally, each category is associated with an icon used to represent it graphically.

In addition, we defined syntactic rules for relationships between part categories. These rules are mostly derived from the SO parts definition. For instance, CDS (SO:0000316) is defined as ‘A contiguous sequence which begins with, and includes, a start codon and ends with, and includes, a stop codon’. Using the information in this definition, it is possible to define a rule stating that a CDS is composed of a START codon (SO:00003180), an ORF (so:0000236) and a STOP codon (SO:0000319). Other rules express that some categories of parts are subsets of a larger category. For instance, it is possible to express that a Bacterial terminator (SO:0000614) is a Terminator (SO:0000141).

#### Correction of annotation errors

A methodic review of the database content unveiled a number of sequence annotation issues, such as feature orientation errors and sequence errors resulting in nonsense mutations. We also merged parts that differed only in the START or STOP codons (see Online Supplement for details).

#### Addition of new parts

We noticed that many of the coding features in some plasmids had no annotated promoters, and this was still the case after we updated the annotations in the current version of SnapGene. To determine the functional promoters for these genes, we aligned their sequences upstream of the start codon with a set of all annotated promoters (one or two variants of each) from our library and performed BLAST searches on the sequences. Some of these promoter regions were new variants of the AmpR promoter, the CAT promoter and the Pc promoter (Supplemental File S3). The rest were promoters that had no counterparts in our features set. We have defined new native promoters for NeoR/KanR, KanR (*aph(3*′*)-Ia*), KanR (*aphA-3*), P2 (*SmR*; works in combination with Pc promoter) and *ccdB*. Plasmids from Oxford Genetics (pSF series) also have an apparent synthetic promoter used for both NeoR/KanR and AmpR. A total of seven new promoters were added to the parts database after this analysis. We also recommend 36 variants that match the consensus/natural (GenBank) sequences for highly variant features, and 17 new versions of features that match the consensus when none of the existing variants do, or comply with optimal sequences from structure-function studies (24–32). See Supplemental Files S1 and S2.

#### Parts libraries

We organized the parts in libraries. One library includes all of the parts in the database. We also have libraries for each of the 13 expression hosts and for the parts having an unspecified expression host. Finally, we have singled out the most popular parts as those having been used in 17 or more plasmids in the SnapGene Sequence library. We also created a library for the new parts described above.

### Use of the GenoCAD grammar

The GenoCAD grammar can be customized for specific applications as previously described (23). Customization starts by adding new categories of parts specific to the application. By convention, the custom category names all start with ‘c-’ i.e. c-Lac Promoter or c-AmpR gene to help identify them quickly among all the existing categories. In the second step, rules are added to describe how parts of different categories can be combined to make a valid construct. Finally, a new parts library specific to a project is created and populated with a selection of parts found in other libraries. It is also possible to import new parts not already in the grammar.

**Figure 2.**
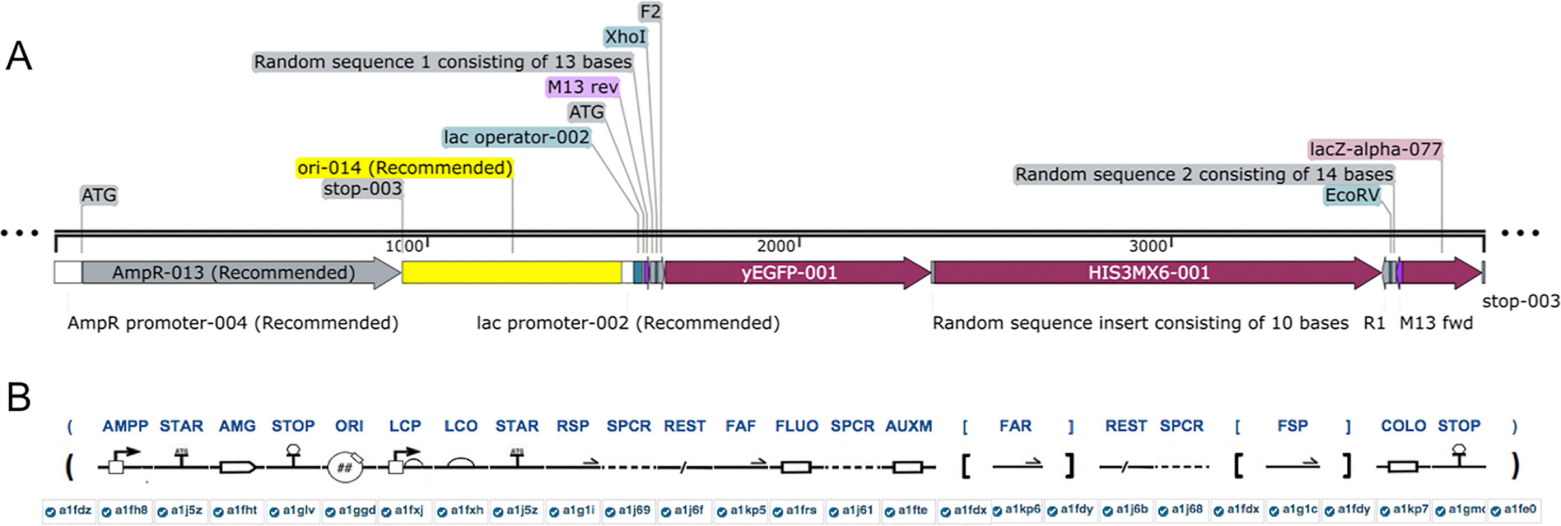
Structure of a plasmid to tag *S. cerevisiae* genes with a fluorescent protein. (**A**) Map of the empty vector and insert derived from the GenBank files exported from GenoCAD. (**B**) Structure of the same plasmid represented using SBOLv icons.

We illustrate this feature by modifying the grammar to make it suitable to design cassettes for tagging *S. cerevisiae* genes with a fluorescent protein (Figure 2). The plasmid has an ampicillin resistance marker and an origin of replication. It also includes a cloning module allowing for blue/white screening. The LacZ-alpha gene is placed under the control of a Lac promoter. A cloning site and two sequencing primers are placed between the start codon and the LacZ-alpha ORF. Randomly generated sequences are inserted between the primers and the cloning site in order to ensure that the borders of the insert can be sequenced.

The cassette itself is composed of a fluorescent protein tag and an auxotrophic marker separated by a short random sequence. The entire cassette is flanked by two polymerase chain reaction (PCR) primer binding sites F2 and R1 used to amplify the cassette to generate PCR fragments for homologous recombination.

## DISCUSSION

### Toward standard sequence features

As synthetic biology is coming of age (33) and its methods and concepts gain acceptance among life scientists, it becomes necessary to develop a common framework for sequence annotation. The analysis of a large library of popular plasmids provided an opportunity to reconcile two different approaches to sequence annotation.

The annotation of plasmid sequences by molecular biologists is a top-down process that starts with the entire sequence of a plasmid. Plasmid sequences are analyzed using a combination of bioinformatics tools and manual curation with the goal of recognizing specific features. The purpose of annotating sequences is to produce maps used to gain an understanding of the plasmid structure and function. Maps are also used for designing cloning strategies to derive new plasmids from existing ones.

Annotation of sequences in synthetic biology is a bottom-up process built into the sequence design workflow. When combining genetic parts to form genetic devices and systems, the annotation of the plasmid sequence is inherited from the annotation of the underlying building blocks.

This difference of approaches can partly explain the surprising diversity of feature sequences observed in our database. In many cases, feature names are associated with a broad range of functionally equivalent DNA sequences that are not uniquely identified (e.g. KanR). Mapping algorithms are designed to tolerate some variability of feature sequences. This approach is very different from the way synthetic biologists tend to identify individual sequences with unique and standardized part numbers.

It would be desirable to reconcile these two approaches by standardizing sequence features. Unambiguous annotations of plasmid sequences using uniquely identified features corresponding to specific DNA sequences would probably greatly improve the usability of plasmid maps. Disambiguation of sequence annotation is particularly important for community resources such as Addgene (10,11) and DNASU (34).

### Conflicting categorization systems

Sequence annotations rely on a system of feature/part categories. One difficulty is the existence of several independent categorization systems. The International Nucleotide Sequence Database Collaboration (35) maintains a list of feature types (http://www.insdc.org/documents/feature-table) used by the GenBank file format. The SO project (18,19) is an alternative. The SO provides a much richer vocabulary and the project allows community members to request new terms. SO terms are used by the SBOL file format (14). Because sequence annotations are used to generate maps, the graphical representation of sequence features in maps needs to be consistent. Even though bioinformatics software applications use fairly standardized arrows and other pictograms to draw plasmid maps, there is no consistent relationship between the type of sequence features and the shape and colors of the blocks used to represent them on the map. SBOL also includes a system of icons called SBOLv but there are many fewer icons than SO terms. Finally, existing vocabularies have been primarily designed to annotate genomic sequences. Many of their terms correspond to features not found in synthetic DNA sequences. Conversely, many of the terms used by the synthetic biology community are not yet present in existing vocabularies.

### Variability of feature sequences

The sequence variability of many features is somewhat unexpected and its origin is difficult to explain. In some cases, mutations are deliberately introduced for functional or cloning purposes. For example, many of the auxotrophic marker variants used in yeast plasmids contain synonymous mutations that remove restriction sites that are also present in the multiple cloning site of the plasmid (36). However, most of the mutations we saw in feature variants had no obvious purpose. Variability can have its source at the sequence level or at the annotation level, and it is not clear when the feature sequence variability reflects the true physical sequences. It is likely that very few plasmids have been completely sequenced, so there may be less variability in the physical sequences of plasmids than in their published sequences. The top–down annotation process is another source of variability. The variability of our feature set reflects the tolerance of sequence annotation algorithms and the different conventions used by different curators when manually annotating sequences.

This dataset does not allow us to estimate what fraction of the variability is attributable to sequencing and annotation errors. However, one should keep in mind the possibility of errors in plasmid sequences and annotations, as such errors can result in lost time and experimental failures. Moreover, if the reported variant sequences are correct, one needs to be aware of which feature variants are present in plasmids as some standard PCR and sequencing primers might not work with all plasmids/variants. Ongoing improvements in software are expected to promote a cultural change in which DNA manipulations are routinely simulated *in silico*, thereby generating complete electronic records of cloning procedures. An essential part of this process will be the development of tools for annotating DNA features in an accurate, consistent manner.

### Anticipated evolution of the database

Looking forward, we anticipate extending the coverage of the database by including sequences from other sources. Plasmids deposited in repositories like Addgene or DNASU could provide a dynamic content fueled by scientific publications. Since not all plasmids deposited in these repositories come with a fully annotated sequence, the curation process would have to be modified in order to make the most of partial annotations likely to reflect new trends in plasmid designs.

The synthetic biology community heavily relies on databases of genetic parts such as the Registry of Standard Biological Parts (6,8), JBEI-ICE (37) and the Virtual Parts Registry (38). It would be interesting to assess to what extent this collection of parts derived from plasmid features complements or overlaps with the current content of existing registries. Databases of genetic parts are also embedded into many software applications (20,39,40). Our large collection of parts validated by decades of use in various expression systems will provide a comprehensive set of quality parts that can be readily imported into such design tools. Conversely, registries of biological parts could provide another stream of annotated sequences to include in the database of standard plasmid features.

All repositories are performing some level of quality control by collecting sequencing data and comparing the physical and reference sequences of the submissions they receive. By automating the comparison of sequencing reads with the features’ published sequences (41), it will be possible to develop a more robust curation process that can rely on this body of experimental data to resolve some annotation problems. Finally, a new generation of algorithms needs to be developed that will be capable of recognizing variants of known features and annotating them in a non-ambiguous way by interacting with the database of standard plasmid features.

## SUPPLEMENTARY DATA

Supplementary Data are available at NAR Online.

SUPPLEMENTARY DATA
