# Supplementary material for: GenoLIB: a database of biological parts derived from a library of common plasmid features
Source: Nucleic Acids Res. 2015 Apr 29;43(10):4823–32. doi: 10.1093/nar/gkv272 (PMC4446419; doi:10.1093/nar/gkv272)
Supplement: SUPPLEMENTARY DATA [file supp_43_10_4823__index.html]

GenoLIB: a database of biological parts derived from a library of common plasmid features — GenoLIB: a database of biological parts derived from a library of common plasmid features — SUPPLEMENTARY DATA 

# GenoLIB: a database of biological parts derived from a library of common plasmid features

## SUPPLEMENTARY DATA

**Files in this Data Supplement:**

- SUPPLEMENTARY DATA
- SUPPLEMENTARY DATA
- SUPPLEMENTARY DATA
- SUPPLEMENTARY DATA
- SUPPLEMENTARY DATA
- SUPPLEMENTARY DATA
