# Supplementary material for: GenoLIB: a database of biological parts derived from a library of common plasmid features
Source: Nucleic Acids Res. 2015 Apr 29;43(10):4823–32. doi: 10.1093/nar/gkv272 (PMC4446419; doi:10.1093/nar/gkv272)
Supplement: SUPPLEMENTARY DATA [file supp_gkv272_nar-03388-h-2014-File005.pdf]

Supplement to: GenoLIB: a database of biological parts derived from a library of common plasmid features

Neil R. Adames[1*], Mandy L. Wilson[1*], Gang Fang[1,2], Matthew W Lux[1,3], Benjamin S. Glick[4,5], Jean Peccoud[1$]

[1]Virginia Bioinformatics Institute, Virginia Tech, Blacksburg (USA)

[2]School of Biological Technology, Xi'an Univ. of Arts and Science, Xi'an (China)

[3]Biosciences Division, Edgewood Chemical Biological Center, Aberdeen Proving Grounds, Maryland, USA

[4]Molecular Genetics & Cell Biology, University of Chicago, Chicago (USA)

[5]GSL Biotech LLC, Chicago (USA).

SnapGene File Library

Annotations

First, the raw DNA sequence provided online is analyzed by SnapGene's common feature detection. This custom algorithm compares sequences in the plasmid to a database of previously annotated common features. A limited number of mismatches or indels are tolerated. When multiple features in the database show partial identity to a region of the plasmid, the best match is chosen. The software annotates each detected feature with a standardized name, appearance, and description.

Second, an annotation is adjusted if appropriate. For example, Clontech's pLVX-Puro vector includes two long terminal repeat (LTR) features that closely match the 3' LTR of human immunodeficiency virus (accession number K03455), but the upstream match was renamed to "5' LTR" to indicate its functional activity in the context of the vector.

Third, extraneous annotated features are removed. For example, many mammalian expression vectors such as pcDNA3.1(+) from Life Technologies contain copies of the lac promoter and lac operator, but these features are remnants of the construction history and are not functionally useful.

Fourth, based on data provided by the company or consortium, relevant features that are not suitable for inclusion in the common features database are annotated. The most common example is a vector-specific multiple cloning site (MCS).

Fifth, if compelling evidence is present that the DNA sequence provided by the company or consortium is erroneous, the error is corrected. For example, many plant vectors in the pCAMBIA series (http://www.cambia.org/daisy/cambia/585) contain a translated feature encoding the RepA replication protein derived from plasmid pVS1, but RepA was not annotated in these vectors due to a missing G in

codon 2 (Heeb, Itoh et al. 2000). The missing G was added before annotating the RepA feature.

Finally, if the company or consortium describes a feature that is likely to be of interest to the research community but is absent from the common features database, that feature is added to the database by the following procedure.

a) With a translated feature, the coding sequence in the plasmid is compared to the originally described coding sequence in the published literature, and in GenBank records when available. If appropriate, the sequence is corrected to match the original or most common version. This process may include adjusting the boundaries of the feature. For example, in pHom-1 from Clontech, the FKBP12-derived DmrB dimerization domain is annotated as spanning bases 676-1008, but the FKBP12-derived sequence actually starts at base 688 (the fifth codon). The SnapGene annotation reflects the FKBP12-derived sequence.

If a translated feature is likely to be encoded using a variety of alternative codons, the plasmid sequence is scanned not only for approximate matches to the DNA sequence, but also for exact matches to the protein sequence. For example, epitope tags are encoded in a variety of ways in different vectors, and are detected and annotated by SnapGene based on their protein sequences.

b) With a non-translated feature, appropriate boundaries are determined. Many such features have been annotated historically in a variety of ways, and the boundaries provided by the company or consortium are sometimes adjusted to be more accurate or informative. Examples of such adjustments include the following.

A feature annotated as being monolithic may actually be a combination of smaller features. For example, plasmids such as pcDNA3.1(+) are annotated as containing the human cytomegalovirus (CMV) immediate early promoter, but actually include both enhancer and promoter regions (Boshart, Weber et al. 1985). In such a case, the SnapGene annotation is broken down into two consecutive features labeled “CMV enhancer” and “CMV promoter”.

A feature annotation may include nonfunctional DNA. For example, in pCMV-3Tag-1a from Agilent Technologies, the herpes simplex virus (HSV) thymidine kinase polyadenylation signal is annotated as being 456 bp, but the polyadenylation signal is only ~49 bp long as determined by functional analysis (Cole and Stacy 1985). The SnapGene annotation covers just these 49 bp.

A feature annotation may exclude potentially functional DNA. For example, in pMT-DEST48 from Life Technologies, the *Drosophila* metallothionein promoter is annotated as spanning bases 406-772, but the sequence derived from the control region of the metallothionein gene spans bases 401-827. This longer sequence is annotated as the metallothionein promoter in other plasmids such as pECIA-14 (GenBank accession number KF444903), and is accordingly used for the SnapGene annotation.

A feature annotation may be offset relative to the original DNA sequence. For example, pQE-TriSystem from Qiagen is annotated as including a chicken β-actin

promoter that spans bases 371-648, but the rabbit DNA sequence actually spans bases 373-650. The SnapGene annotation corresponds to the rabbit DNA sequence.

c) In some cases, this analysis has revealed that multiple variants of a feature are in common use. Such variants may have different endpoints or may include indels. If the differences are substantial, the common features database includes multiple variants. For example, variants of the yeast *ADH1* promoter share the same 3' endpoint but differ at the 5' endpoint, and the common features database includes three such variants with lengths of 397, 402, and 705 bp.

Elimination of duplicate files

The SnapGene File Library is organized in 13 collections (Table S1; Supplemental File S1). One collection includes basic cloning vectors. The other collections correspond to expression hosts (mammalian, viral, plant, insect, and yeast expression vectors), specific projects such as the I.M.A.G.E. or Structural Genomics consortia, different categories of reporter genes (luciferase and fluorescent proteins), and different vendors (Novagen, Qiagen, and GE Healthcare). This library was assembled over a period of several years by retrieving plasmid sequences and descriptions available from various suppliers, and by annotating each plasmid according to SnapGene conventions as described in the Online Supplement.

We first reviewed this library to eliminate irrelevant entries such as two phage genomes (lambda and phiX174), and to eliminate redundancies due to plasmids that have identical features but different feature orientations (e.g., pBlueScript SK (+).dna and pBlueScript SK (-).dna) or different topologies (e.g., pEZSeq KAN.dna and pEZSeq KAN (linearized).dna). Next, we eliminated duplications for 24 sequences that were found in more than one collection, and for seven sequences that appeared more than once under different names. This process left us with a Non-Redundant File Library (Table 1).

The Non-Redundant File Library includes non-plasmid files that contain just a single feature. For instance, the Fluorescent Protein Genes and Plasmids set includes 143 sequence files corresponding to fluorescent protein coding sequences, and the Basic Cloning Vectors set includes 17 single feature files. We kept these files because some of the features may not be used by plasmids in the library.

When the above issues were resolved, 1718 distinct sequence files remained from the original 1901 (SnapGene File Library); of the 1718 remaining files (Non-Redundant File Library), 1557 were plasmids (Non-Redundant Plasmid Library) and 161 were single features.

Characterization

Table S1 reports the number of sequence files found in each collection along with the range and median sequence size in each collection and the entire dataset.

The features in the Non-Redundant File Library were imported into a database by converting the SnapGene format files into GenBank files and then parsing the GenBank files. We recorded each feature's name, description, GenBank qualifier,

location within the plasmid, orientation, and sequence. We also associated each feature with the plasmid from which it originated. We tried to automatically eliminate single feature files to generate a Non-Redundant Plasmid Library, but some single feature files included promoters or selection cassettes annotated with several features. We also observed that our dataset included modified baculoviruses that do not qualify as plasmids. Therefore, we manually reviewed the content of the Non-Redundant File Library and eliminated 15 non-plasmid files (13 single feature files and 2 baculovirus vectors).

We then characterized the complexity of the plasmids included in the Non-Redundant Plasmid Library. Figure S1A shows a histogram of the plasmid sequence lengths. The shortest plasmid is 1,691 bp long (pYX-Asc), the longest is 49,757 bp long (pCAMBIA5105), and the median plasmid length is 5.2 kb.

The statistical distributions of the number of features per sequence file in each collection and for the entire library are reported in Table S1 and Figure S1B. Two plasmids have only 3 features. pCAMBIA5105 has 47 features. With 33 features, the lentiviral vector pTRIPZ is the second most feature-rich plasmid. The median number of features per plasmid is 14.

	**Sequence Files**				**Features**			
	Number	**Sequence Length**			***Number***	**Features/File**		
**Collection**		Min	Med.	Max		Min	Med.	Max
Basic Cloning Vectors	*172*	17	3,029	48,514	*2,079*	1	12	187
Fluorescent Protein Genes and Plasmids	*312*	657	3,336	5,443	*1,952*	1	8	13
I.M.A.G.E. Consortium Plasmids	*59*	1,691	4,095	7,620	*723*	5	12	17
Insect Cell Vectors	*133*	2,231	5,517	139,378	*2,024*	6	13	29
Luciferase Vectors	*162*	3,110	5,414	8,721	*1,980*	6	12	20
Mammalian Expression Vectors	*280*	2,605	5,319	9,621	*4,167*	4	15	22
pET & Duet Vectors (Novagen)	*120*	3,306	5,422	12,474	*1,811*	5	16	27
pGEX Vectors (GE Healthcare)	*13*	4,947	4,970	4,985	*143*	10	11	12
Plant Vectors	*91*	3,228	10,454	49,757	*1,528*	8	16	48
Qiagen Vectors	*56*	276	3,852	5,796	*835*	4	14	25
Structural Genomics Vectors	*195*	2,115	5,822	8,538	*3,120*	10	16	22
Viral Expression & Packaging Vectors	*137*	3,144	8,139	36,686	*2,389*	3	17	33
Yeast Plasmids	*171*	267	4,967	10,667	*1,842*	1	11	19
**Entire Library**	***1,901***	**17**	**5,092**	**139,378**	***24,593***	**1**	**13**	**187**

Table S 1: Overview of the SnapGene File Library.

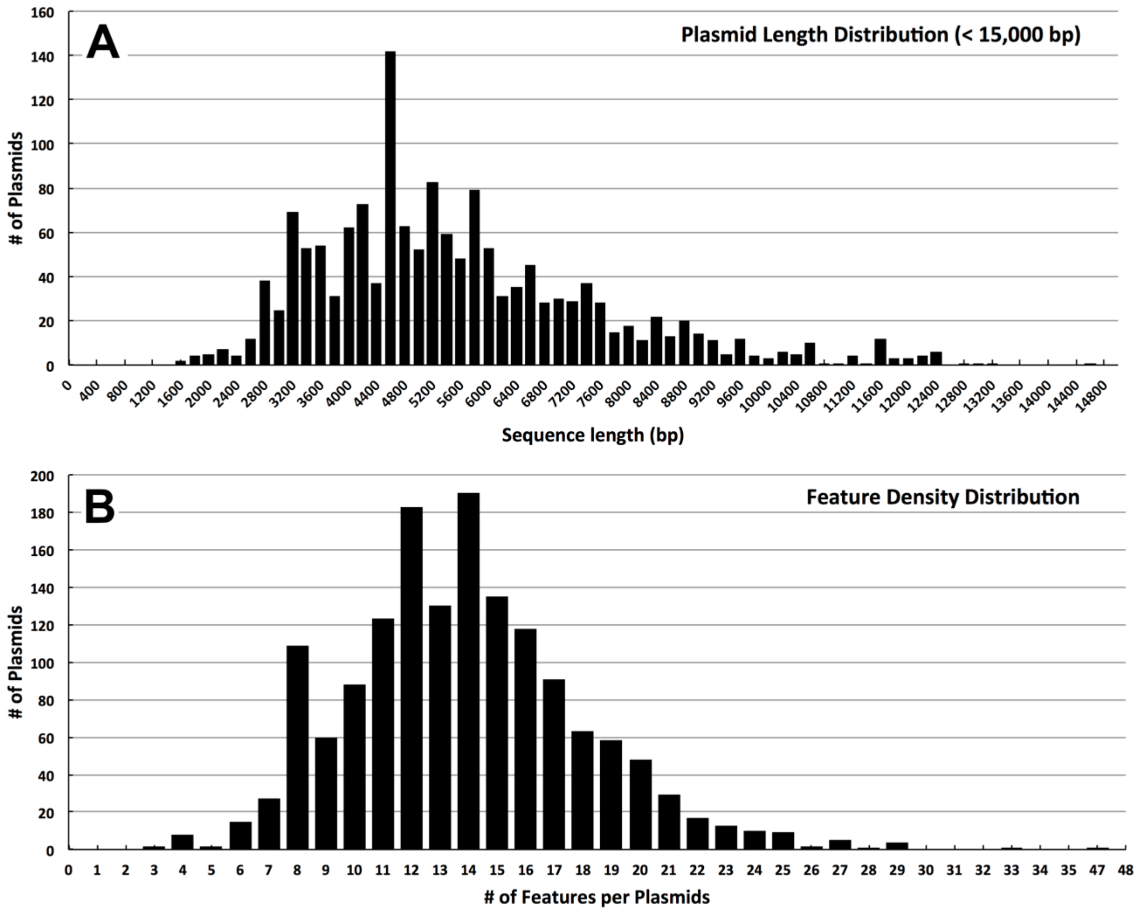

Figure S1: Statistical Distributions of plasmid lengths and feature densities. (A) Histogram of the plasmid sequence length. The plasmid lengths vary considerably, with a minimum length of 1,691 bp, a maximum of 49,757 bp, and a median of 5283 bp. Bins are 200 bp. (B) Histogram of the number of features per plasmid. The minimum number of features per plasmid was 3 features, the maximum was 47 features, with a median of 14.

In Figure 1, scatter plots show the correlation between plasmid length and the number of features, in other words feature density. Plasmids were manually grouped by type of plasmid/features and lab host. Not shown in the scatter plots are vectors containing large regions of natural viral or bacterial genomes. Two linear vectors encoding baculoviruses (Baculodirect, ~139 kb) have only 32 features and hundreds of un-annotated ORFs. The SnapGene File Library also includes 10 adenoviral vectors for mammalian gene therapy. These vectors are large (~32.6-36.7 kb), but carry only up to 36 features and are mostly composed of 24-30 kb of the adenovirus genome, which is annotated as a single feature although this region encodes over a dozen ORFs. Finally, two plant vectors, pSB1 (36.9 kb, 27 annotated features) and pCAMBIA5105 (49.7 kb, 47 annotated features), carry large portions of the Agrobacterium pTiBo542 plasmid encoding many virulence genes in their natural context.

As a group, the multi-host expression plasmids are feature-rich (Figure 1). Because these plasmids allow for expression in 2 or 3 different hosts (bacteria-insect, bacteria-mammal, insect-mammal, and bacteria-insect-mammal), they contain

multiple selectable markers, promoters and terminators specific to each host, accounting for their high feature densities.

Plasmids for cloning tend to be small (several kb), requiring only a cloning site, a bacterial origin of replication and a selectable marker, but pSC101 and the pJAZZ series of vectors were extreme outliers. pSC101 is the very first bacterial (*Salmonella enterica*) plasmid used for cloning. Consequently, construction of the plasmid using available natural restriction enzyme sites left large regions that flank the functional features and encode multiple ORFs that have no function for cloning or plasmid propagation. The pJAZZ vectors are linear vectors for cloning DNA that is unstable in circular plasmids. These vectors are based on coliphage N15 chromosomes and carry bacterial telomeres and several ORFs required for chromosome maintenance.

Plant vectors tend to be relatively feature-sparse, mostly due to relatively large numbers of ORFs. For example, the pEarleyGate and pCAMBIA series encode multiple selectable markers and several large ORFs required for plasmid maintenance and have relatively large intergenic regions. In contrast, the pGREEN plasmid series is more characteristic of mammalian and bacterial plasmid feature densities. These plasmids have small origins of replication and little intergenic DNA. The pHELLSGATE series appeared to have low feature densities, but were actually under-annotated in the downloaded files. The current version of SnapGene detected 9-13 additional features in these plasmids, which gave them a feature density consistent with the rest of the plant plasmids.

Among the insect plasmids, there were a large group of baculoviral-transfer vectors that had lower feature densities than the bulk of the plant vectors, including other baculovirus vectors. Most of these plasmids were in the pAc and pAB series of plasmids and were found to have 5-6 additional features detected with the current version of SnapGene. However, even with the updated features, many of these plasmids showed slightly lower feature densities. This is likely due to their common use of a baculovirus recombination region that includes up to 4.5 kb of flanking DNA. Other baculoviral-transfer vectors, such as the pBAC series, carry a more compact recombination region with only ~400 bp of flanking DNA.

Many outliers among the fungal plasmids were also under-annotated in the downloaded files. Once the features were updated in SnapGene, plasmids in the pPINK series (*Pichia*) and pKLAC series (*Kluyveromyces*) showed typical feature densities. YEp24, YEp13 and YCp50, were still feature sparse after the updates, but these vectors were among the first yeast expression vectors and carry significant amounts of non-functional DNA (over 3 kb for YEp13) flanking their chromosomally-derived features. For example, the *CEN/ARS* of YCp50 is 1850 bp compared to only 504 for pRS316. The other plasmids in the YEp3XX series or *Pichia* expression plasmids carry several large features (e.g. lacZ ~3 kb, 2μ origin ~ 1.3 kb, *AOX1* promoter ~ 0.9 kb, *PpHIS4* ~ 2.5 kb) that reduce the feature density.

Analysis of the standard feature library

Feature usage statistics

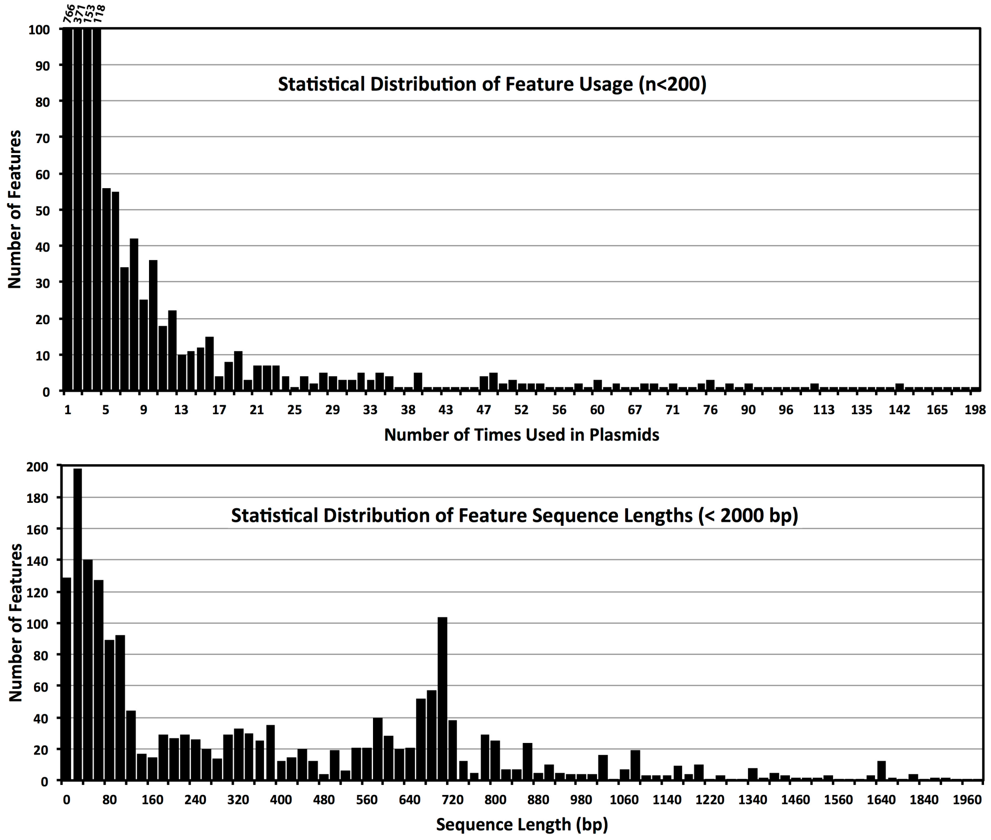

Figure S2: Statistical distributions of feature lengths and usage statistics (Top) Cumulative distribution function of feature usage. The x axis corresponds to the number of times each feature has been used. The y axis is the probability of the features being used at least n times. Partial view of the CDF corresponding to features used less than 200 times. (Bottom) Histogram of the feature sequence length. The x-axis corresponds to the length of the feature sequence in base pairs (bp). The y axis indicates how many features have that sequence length. Bins are 20 bp.

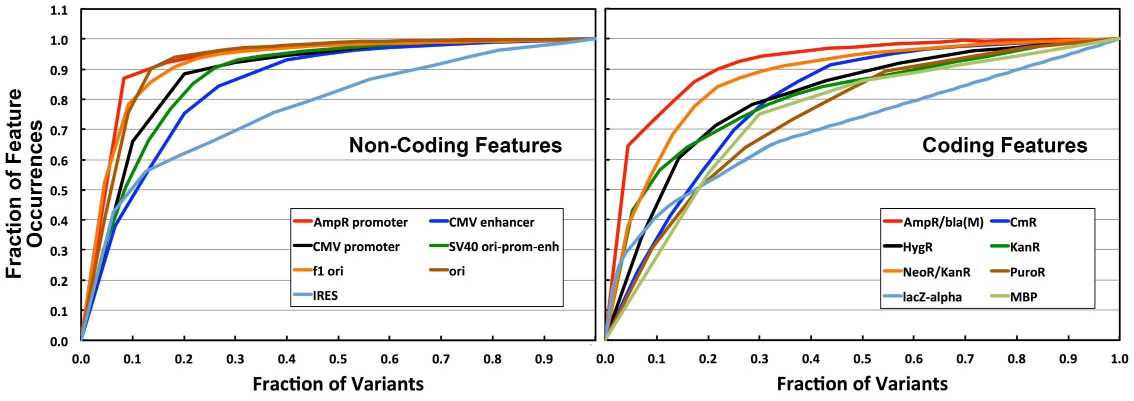

Figure S3: Statistical distributions of feature variant usage. Cumulative distribution functions of feature variant usage for non-coding (left panel) and coding features (right panel). Each feature has 10 or more annotated variants. Because the number of variants for each feature is different, the x-axis is normalized to the fraction of the total number of variants for each feature.

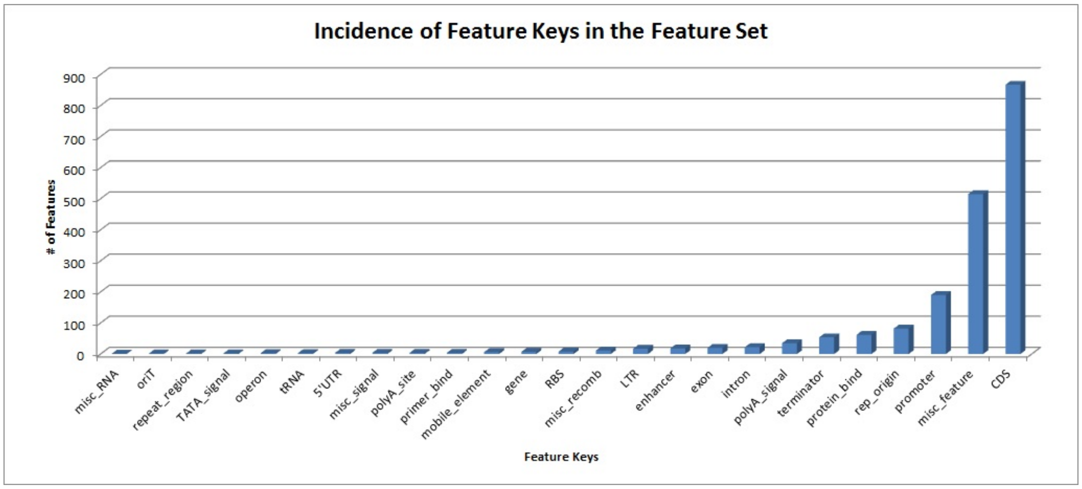

Figure S4: Distribution of features according to the GenBank feature key. The least commonly used feature key, misc_RNA, was used once; the most commonly used, CDS, was used 867 times; the median feature key, gene, was used 8 times.

Name of Feature	Feature Key	# of occurrences	# of occurrences of all variants of the feature
AmpR promoter-009	promoter	967	1110
AmpR-013	CDS	688	1060
T7 promoter	promoter	625	625
ori-014	rep_origin	602	1494
ori-013	rep_origin	524	1494
ATG	CDS	414	414
f1 ori-008	rep_origin	342	651[1]
CMV promoter-004	promoter	338	511
SV40 poly(A) signal-003	polyA_signal	338	645
lac operator-003	protein_bind	278	613[2]
tet operator-001	protein_bind	277	326[2]
M13 rev	primer_bind	270	270
SV40 ori-004	rep_origin	261	427
M13 fwd	primer_bind	239	239
ori-009	rep_origin	220	1494
lac promoter-002	promoter	205	225

Table S 2: Features that occur over 200 times in the Non-Redundant Plasmid Library

1 - includes features with M13 ori feature name.
2 – operator occurrences include only instances in which the operator is annotated (does not include instances in which the operator is included in a promoter, but is not annotated) .

Analysis of Feature Variants

There were two cases in which features were misidentified: the M13 and f1 origins of replication differ by a single nucleotide, and f1 ori-006, -013 and -015 variants are actually M13 origins. We also found that TetR-003 was misnamed in a series of yeast plasmids. Although functionally conferring tetracycline resistance, TetR is

properly the transcriptional repressor protein, while TetR-003 is actually TcR, the tetracycline efflux pump. Both cases occurred because each sequence was automatically given its most commonly used (and incorrect) name.

The use of non-coding feature variants was relatively conservative. The most used non-coding variant, AmpR prom-009, is used in 87% of AmpR promoter instances. The least conserved non-coding feature is the IRES (internal ribosome entry site from encaphalocardiomyopathy virus), mostly because some of the variants have functional differences in translational activity. The maximum number of mismatches and indels for the non-coding feature variants was 20 (f1 ori-004), but the average was just 2 bp changes/variant (5.4/1,000 bp). Misannotations of non-coding features tended to be the wrong orientation, although a few variants are likely sequence errors, as the changes are known or predicted to impair function (Table S1).

Variants of protein coding features were more broadly used and less conserved. The most conserved of these features, AmpR-013, is used in 64% of instances. The least conserved coding feature is lacZ-α. However, lacZ-α is a special case because the majority of lacZ-α variants (70/74) differ only in the multiple cloning site that occurs in-frame near the N-terminus and is used for blue-white screening of inserts. The next least conserved coding feature is maltose binding protein MBP, in which the most common variant, MBP-004, is used in only 28% of instances. Extreme examples of the use of synonymous codons are the AmpR-021 variant (102 synonymous codons/861), HygR-010 (222/1038) and PuroR-007 (106/600), which are synthetic sequences used by Promega (Supplemental File S3). Misannotations of coding regions were largely due to trimming of additional in-frame sequences that removes parts of the conserved sequence (mostly START and STOP codons), but there were also cases of clear sequence errors that introduce nonsense mutations (Online Supplement).

Overall, mismatches and indels were sporadically distributed in sequences and each mismatch tended to occur in multiple variants. Along with the observation that most mismatches in coding region variants resulted in synonymous codon changes, these observations suggest that most variants are the result of unplanned mutations (real or due to sequence errors) that have little or no functional consequences and have been propagated during plasmid construction/annotation. Misannotations of coding regions were largely due to trimming of additional in-frame sequences that removes parts of the conserved sequence (mostly START and STOP codons), but there were also cases of clear sequence errors that introduce nonsense mutations (Table S1).

Inclusion relations

A query was run to determine how frequently one feature sequence contained the sequence from another feature (ie, a promoter feature contained within another promoter or CDS feature.) The purpose of this analysis is to detect complex features that include several smaller features, or to help identify situations where a feature appears in the sequence of another feature by accident. Features with sequences under 6 bp were omitted from consideration because they were likely to be included

too often to be meaningful. After those features were omitted, there were 1557 cases where one feature sequence was included within another feature sequence; since many of those were truncated versions of the same feature (ie, a truncated MCS found within a longer MCS sequence), we decided not to include these types of inclusion relationships in Figure S. In the final analysis, there were 994 inclusion relationships within the features set; this includes 176 features whose sequences are included within the sequences of other features and 432 features that include the sequences of other features within their sequence; in total, there are 579 features that are involved in some kind of inclusion relationship (Figure 4). This network can be explored interactively by loading File S5 in Cytoscape (Shannon, Markiel et al. 2003). Of the 1943 total features, 932 did not appear on either side of an inclusion relationship. Finally, the results of the inclusion query were joined with the annotations from the original GenBank files to determine whether the inclusion was annotated or not in the original file. Interestingly, 705 inclusion relationships were not annotated, compared to 289 documented inclusion relationships.

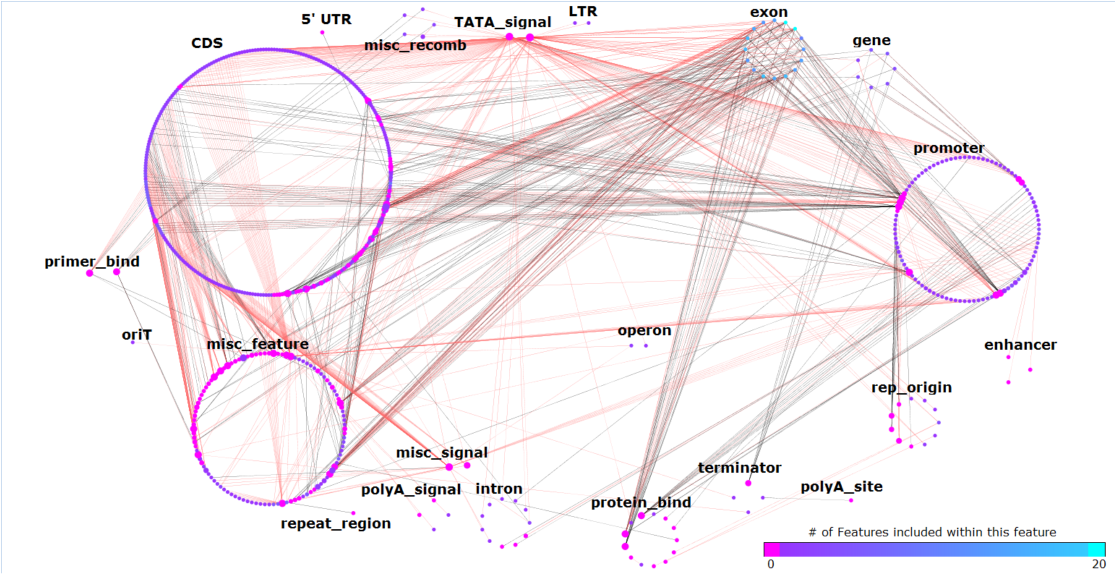

Figure S5: Network of feature inclusion relationships. The color of the nodes indicates how many features are included within that feature (from Magenta for 0 inclusions to Cyan for the maximum number). The size of the node indicates how often the feature is included within longer features. Black edges between nodes indicate that the inclusion was annotated in the original GenBank file; red edges indicate that the inclusion was not annotated. Finally, the nodes are grouped by feature key.

Library of standard genetic parts

Generation of SBOL files

GenBank Key	GenBank Description	SO Qualifier	Identifier
5'utr	5' untranslated region (leader)	five_prime_utr	so:0000204
cds	Sequence coding for amino acids in protein (includes stop codon)	cds	so:0000316
enhancer	Cis-acting enhancer of promoter function	enhancer	so:0000165

exon	Region that codes for part of spliced mRNA	exon	so:0000147
gene	region of biological interest identified as a gene and for which a name has been assigned;	gene	so:0000704
intron	Transcribed region excised by mRNA splicing	intron	so:0000188
LTR	Long terminal repeat	long_terminal_repeat	so:0000286
misc_feature	Region of biological significance that cannot be described by any other feature	region	SO:0000001
misc_recomb	Miscellaneous recombination feature	recombination_feature	so:0000298
misc_RNA	Miscellaneous transcript feature not defined by other RNA keys	mature_transcript	so:0000233
misc_signal	Miscellaneous signal	regulatory_region	so:0005836
mobile_element	region of genome containing mobile elements;	mobile_element_insertion	SO:0001837
operon	region containing polycistronic transcript including a cluster of genes that are under the control of the same regulatory sequences/promotor and in the same biological pathway	operon	so:0000178
oriT	origin of transfer;	origin_of_transfer	so:0000724
polyA_signal	Signal for cleavage & polyadenylation	polyA_signal_sequence	so:0000551
polyA_site	Site at which polyadenine is added to mRNA	polyA_site	so:0000553
primer_bind	Non-covalent primer binding site	primer_binding_site	so:0005850
promoter	A region involved in transcription initiation	promoter	so:0000167
protein_bind	Non-covalent protein binding site on DNA or RNA	protein_binding_site	so:0000410
RBS	Ribosome binding site	Shine_Dalgarno_sequence	SO:0000552
rep_origin	Replication origin for duplex DNA	origin_of_replication	SO:0000296
repeat_region	Sequence containing repeated subsequences	repeat_region	so:0000657
TATA_signal	`TATA box' in eukaryotic promoters	tata_box	so:0000174
terminator	Sequence causing transcription termination	terminator	so:0000141
tRNA	Transfer RNA	tRNA	so:0000253

Table S 3: Mapping of GenBank feature qualifiers to Sequence Ontology (SO) terms

Correction of feature annotation issues

To our knowledge, there is little experimental functional comparison of the feature variants we describe here. For non-coding features, it is difficult to predict the effects of these variations on transcription or replication, but the effects must be small since most of the variants we examined are essential for plasmid function. The most common annotation error among the non-coding feature variants was in assigning orientation (Supplemental File S3; Table S2). Many of the enhancer and replication origin variants were actually reverse complement sequences (relative to the consensus), some of which were identical to another variant. In such cases, these variants were merged for statistical analysis. Some variants were reverse complements of the consensus, but unique. In these cases, we simply corrected the

orientation of the variant sequence. These orientation annotation errors likely arose because these features can function independently of orientation.

There were a few sequence variants that, if the sequence is correct, would be functionally impaired. The f1 ori variants -013 and -017 contain inserts in regions that form stem structures important for replication. We found only one other annotation error among the non-coding features. CMV-pro-007 was actually a combination of the CMV enhancer and promoter, which are separately annotated in all other cases (Supplemental File S3; Table S2).

Sequence or annotation errors were more obvious in the coding features. Two of the feature variants we examined – AmpR-002 and lacZ-α-041 – had nonsense mutations (Supplemental File S3; Table S2). Some coding features have inactivated variants containing deliberate nonsense mutations, but these were not included as variants of the "wild-type" feature. Neither AmpR-002 nor lacZ-α-041 were annotated as "inactivated" features and both are identical to another variant when their sequence is corrected to change the single bp change from consensus that produces a premature in-frame STOP codon. Therefore, these variants likely contain a sequence error.

There were also numerous annotation errors in assigning the sequence coordinates of the open reading frames. These resulted in either removing the N-terminus (including the START codon) or the C-terminus of the translated protein (including the STOP codon). When we corrected the sequences to include the missing START or STOP codons, the variant was usually identical to another more common variant and was merged with it. Good examples of this type of mis-annotation were two bla(M) variants (Supplemental File S3; Table S2). Bla(M) is AmpR with the signal sequence removed. However, bla(M)-001 and -003 were missing the START codon. When we reviewed their corresponding plasmid sequences, we found that the in-frame START codon occurred well upstream of the annotated ORF, adding the conserved signal sequence. In fact, bla(M)-001 and bla(M)-003 were identical to AmpR-001 and AmpR-008, respectively, and were merged with those variants.

In a few cases, the coding feature annotations were correct, but were missing START or STOP codons because they occur as in-frame fusions with another coding feature (Table S2).

Feature Variant	Problem	Cause	Change to Sequence	Result
CMV_enh-001	Orientation	Reverse complement not indicated in annotation	Reverse complement	Merged with CMV_enh-003
CMV_enh-014	Orientation	Reverse complement not indicated in annotation	Reverse complement	Merged with CMV_enh-015
CMV_pro-007	Composite sequence	Includes both enhancer and promoter	Split sequence	Enhancer is unique CMV-enh variant<br>Promoter merged with CMV_pro-004
f1 ori-013	3 bp insert in Stem-Loop C	Likely sequence error	None	Not a suggested sequence
f1 ori-017	17 bp insert in Stem-Loop D	Likely sequence error	None	Not a suggested sequence
SV40_ori-006	Orientation	Reverse complement not indicated in annotation	Reverse complement	Remains SV40_ori-006
SV40_ori-005	Orientation	Reverse complement not indicated in annotation	Reverse complement	Merged with SV40_ori-004
SV40_enh	Orientation	Reverse complement not indicated in annotation	Reverse complement	Remains SV40_enh
ori2-002	Orientation	Reverse complement not indicated in annotation	Reverse complement	Merged with ori2-001
oriV-004	Orientation	Reverse complement not indicated in annotation	Reverse complement	Merged with oriV-003
pSC101ori-002	Orientation	Reverse complement not indicated in annotation	Reverse complement	Merged with pSC101ori-001
lac operator-003	Orientation	Reverse complement not indicated in annotation	Reverse complement	Merged with lac operator-001
lac operator-004	Orientation	Reverse complement not indicated in annotation	Reverse complement	Merged with lac operator-002
tet operator-002	Orientation	Reverse complement not indicated in annotation	Reverse complement	Merged with tet operator-001
CAP binding site-002	Orientation	Reverse complement not indicated in annotation	Reverse complement	Merged with CAP binding site-001
AmpR-002	Nonsense mutation	C to A at position 624 bp/codon 208	A to C	Merged with AmpR-004
AmpR-009	Missing START	Error in plasmid annotation coordinates	Coordinates corrected	Merged with AmpR-016
Bla(M)-001	Missing START	Error in plasmid annotation coordinates	Coordinates corrected	Merged with AmpR-001
Bla(M)-003	Missing START	Error in plasmid annotation coordinates	Coordinates corrected	Merged with AmpR-008
CmR-009	Missing STOP	Error in plasmid annotation coordinates	Coordinates corrected	Remains CmR-009
KanR-016	Name ambiguity	Sequence is a fusion of *nos* (nopaline synthase) N-terminus and *nptII/aph(3')-II* (NeoR/KanR) but most KanR are *aph(3')-Ia*	None	Suggest new name KanR-nos-nptII
KanR-020	Name ambiguity	Sequence is *aphA-3* but most KanR are *aph(3')-Ia*	None	Suggest new name KanR-nptII
NeoR/ KanR-010	Missing START	An in-frame fusion with hRluc	None	Identical to NeoR/KanR-002 but remains as NeoR/KanR-010
lacZ-α-016	Missing STOP	An in-frame fusion with ccdB	None	Identical to lacZ-α-053 but remains as lacZ-α-016
lacZ-α-021	Missing STOP	An in-frame fusion with ccdB	None	Identical to lacZ-α-029 but remains as lacZ-α-021
lacZ-α-036	Missing STOP	Error in plasmid annotation coordinates	Coordinates corrected	Merged with lacZ-α-052
lacZ-α-041	Frame-shift mutation	Single bp deletion at position 115 (GAATC) leads to frameshift	T inserted to regenerate EcoRI site (GAATTC)	Merged with lacZ-α-023
TetR-003	Mis-annotated	Mis-named TetR in a series of yeast plasmids derived from pSC101 (in which it is correctly annotated as TcR). Most common name used for the feature.		Actually a unique variant of TcR (tetC)

Table S 4: Feature variants with sequence problems.
