# Supplementary material for: GenoLIB: a database of biological parts derived from a library of common plasmid features
Source: Nucleic Acids Res. 2015 Apr 29;43(10):4823–32. doi: 10.1093/nar/gkv272 (PMC4446419; doi:10.1093/nar/gkv272)
Supplement: SUPPLEMENTARY DATA [file supp_gkv272_nar-03388-h-2014-File006.pdf]

Sequence Alignments

Non-Coding Sequences

All -35 and -10 bacterial promoter sequences are predicted by BPROM (http://linux1.softberry.com/berry.phtml) and some are additionally annotated in public sequences.

Name of Variant	# of Occur	Alignment	Size (bp)	# Var bp	Sources*
AmpR_prom-009	967	CGCGGAACCCCTATTTGTTTATTTTTCTAAATACATTCAAATATGTATCCGCTCATGAGACAATAACCCT-GATAAATGCTTCAATAAT-ATTGAAAAAGGAAGAGT	105	0	In,No,Or,Pr, etc
AmpR_prom-001	58	CGCGGAACCCCTATTTGTTTATTTTTCTAAATACATTCAAATATGTATCCGCTCATGAGACAATAACCCT-GATAAATGCTTCAATAAT--TTGAAAAAGGAAGAGT	104	0/1d	MCSG
AmpR_prom-006	32	CGCGGAACCCCTATTTGTTTATTTTTCTAAATACATTCAAATATGTATCCGCTCATGAGACAATAACCCT-GATAAATGCTTCAATAAT-ATTGAAAAAGGAAGA--	103	0/2d	Ag
AmpR_prom-003	20	-------------TTTGTTTATTTTTCTAAATACATTCAAATATGTATCCGCTCATGAGACAATAACCCT-GATAAATGCTTCAATAAT-ATTGAAAAAGGAAGAGT	92	0	No,BR,Or,In
AmpR_prom-005	11	-------------TTTGTTTATTTTTCTAAATACATTCAAATATGTATCCGCTCATGAGACAATAACCCT-GATAAATGCTTCAATCATGATTGAAAAAGGAAGAGT	93	1/1i	No
AmpR_prom-008	10	CGCGGGACCCCTATTTGTTTATTTTTCTAAATACATTCAAATATGTATCCGCTCATGAGACAATAACCCT-GATAAATGCTTCAATAAT-ATTGAAAAAGGAAGAGT	105	1	Or
AmpR_prom-010	5	-------------TTTGTTTATTTTTCTAAATACATTCAAATATGTATCCGCTCATGAGACAATAACCCT-GATAAATGCTTCAATAAT-AATGAAAAAGGAAGAGT	92	1	MCSG
AmpR_prom-004	2	---------------------------------CATTCAAATATGTATCCGCTCATGAGACAATAACCCT-GATAAATGCTTCAATAAT-ATTGAAAAAGGAAGAGT	72	0	Lu
AmpR_prom-011	2	CGCGGAACCCCTATTTGTTTATTTTTCTAAATACATTCAAATATGTATCCGCTCATGAGACAATAACCCT-GATAAATGCTTCAATAAT-ATTGAAAAAAGAAGAGT	105	1	Ta
AmpR_prom-002	1	CGCGGAACCCCTATTTGTTTATTTTTCTAAATACATTCAAATATGTATCCGCTCATGAGACAATAACCCTTGATAAATGCTTCAATAAT-ATTGAAAAAGGAAGAGT	106	0/1i	Ag
AmpR_prom-007	1	---------------------------------CCTTCAAATATGTATCCGCTCAGGAGACAATAACCCT-GATAAATGCTTCAATAAT-ATTGAAAAAGGAAGAGT	72	2	No
AmpR_prom-012	1	CGCGGAACCCCTATTTCTTTATTTTTCTAAATACATTCAAATATGTATCCGCTCATGAGACAATAACCCT-GATAAATGCTTCAATAAT-ATTGAAAAAGGAAGAGT	105	1	BD
		* ******************* ************** *************** ** ******* *****			
		-35 *bla TEM* prom -10 RBS			

Key ATGC = change in nucleotide relative to consensus sequence – non-coding sequence

AB – AB Vector
AG – AddGene
Ag – Agilent
BD – BD Biosciences
BR – Bio-Rad
Ca – Cambia
Cl – Clontech
Ev – Evrogen
GB – Geneva Biotech
GE – GE Life Sciences
iGEM – international Genetically Engineered Machine Foundation
IMAGE – I.M.A.G.E. Consortium
In – Invitrogen
Iv – InvivoGen
Lu – Lucigen
MBL – MBL International
MCSG – Midwest Center for Structural Genomics
Mo – MoBiTec
Ne – New England Biolabs
No – Novagen
OB – Open Biosystems
Or – OriGene
Ox – Oxford Genetics
Pr – Promega
Qi – Qiagen
SG – SwitchGear Genomics
Si – Sigma-Aldrich
Ta – TaKaRa
Th – Thermo Scientific

Sequence Alignments

```
Name of        # of   Alignment                                                                                                                   Size   # Var   Sources*
Variant        Occur                                                                                                                              (bp)   bp
CMV_enh-003    198+9  ----------------------------------------------------------------------------CGTTACATAACTTACGGTAAATGGCCCGCCTGGCTGACCGCCCA  304    0       Cl,Ev,Ox,In
CMV_enh-015    97+4   GACATTGATTATTGACTAGTTATTAATAGTAATCAATTACGGGGTCATTAGTTCATAGCCCATATATGGAGTTCCGCGTTACATAACTTACGGTAAATGGCCCGCCTGGCTGACCGCCCA  380    2       Or,Si
CMV_enh-009     96    GACATTGATTATTGACTAGTTATTAATAGTAATCAATTACGGGGTCATTAGTTCATAGCCCATATATGGAGTTCCGCGTTACATAACTTACGGTAAATGGCCCGCCTGGCTGACCGCCCA  380    0       In,Cl,Or
CMV_enh-008    47     ---------------------------------------------------------------------------GCGTTACATAACTTACGGTAAATGGCCCGCCTGGCTGACCGCCCA  305    0       Ag,Mo
CMV_enh-005    23     GACATTGATTATTGACTAGTTATTAATAGTAATCAATTACGGGGTCATTAGTTCATAGCCCATATATGGAGTTCCGCGTTACATAACTTACGGTAAATGGCCCGCCTGGCTGACCGCCCA  380    1       In,Ne
CMV_enh-010    22     ----------------------------------------------------------------------------CGTTACATAACTTACGGTAAATGGCCCGCCTGGCTGACCGCCCA  304    2       Pr,No,Or
CMV_enh-011    10     ----------------------------------------------------------------------------CGTTACATAACTTACGGTAAATGGCCCGCCTGGCTGACCGCCCA  304    1       Cl
CMV_enh-016    7      --------------------------------------------------------------------------CGCGTTACATAACTTACGGTAAATGGCCCGCCTGGCTGACCGCCCA  306    0       Qi
CMV_enh-002    4      GACATTGATTATTGACTAGTTATTAATAGTAATCAATTACGGGGTCATTAGTTCATAGCCCATATATGGAGTTCCGCGTTACATAACTTACGGTAAATGGCCCGCCTGGCTGACCGCCCA  381    0/1i    Cl
CMV_enh-006    4      ----------------------------------------------------------------------------CGTTACATAACTTACGGTAAATGGCCCGCCTGGCTGACCGCCCA  304    1       No
CMV_enh-013    3      ---------------------------------------------------------------------------GCGTTACATAACTTACGGTAAATGGCCCGCCTGGCTGACCGCCCA  305    2       Pr,Cl
CMV_enh-012    2      ----------------------------------------------------------------------------CGTTACATAACTTACGCTAAATGGCCCGCCTGGCTGACCGCCCA  304    1       Cl
CMV_enh-004    2      ----------------------------------------------------------------------------CGTTACATAACTTACGGTAAATGGCCCGCCTGGCTGACCGCCCA  304    2       Lu
CMV_enh-007    2      GGCATTGATTATTGACTAGTTATTAATAGTAATCAATTACGGGGTCATTAGTTCATAGCCCATATATGGAGTTCCGCGTTACATAACTTACGGTAAATGGCCCGCCTGGCTGACCGCCCA  380    2       Cl
CMV_pro-007    2      -------------------------------------------------------------------GGAGTTCCGCGTTACATAACTTACGGTAAATGGCCCGCCTGGCTGACCGCCCA  517    0       Cl
                                                                                                  **************** ***************************

CMV_enh-003           ACGACCCCCGCCCATTGACGTCAATAATGACGTATGTTCCCATAGTAACGCCAATAGGGACTTTCCATTGACGTCAATGGGTGGAGTATTTACGGTAAACTGCCCACTTGGCAGTACATC
CMV_enh-015           ACGACCCCCGCCCATTGACGTCAATAATGACGTATGTTCCCATAGTAACGCCAATAGGGACTTTCCATTGACGTCAATGGGTGGAGTATTTACGGTAAACTGCCCACTTGGCAGTACATC
CMV_enh-009           ACGACCCCCGCCCATTGACGTCAATAATGACGTATGTTCCCATAGTAACGCCAATAGGGACTTTCCATTGACGTCAATGGGTGGAGTATTTACGGTAAACTGCCCACTTGGCAGTACATC
CMV_enh-008           ACGACCCCCGCCCATTGACGTCAATAATGACGTATGTTCCCATAGTAACGCCAATAGGGACTTTCCATTGACGTCAATGGGTGGAGTATTTACGGTAAACTGCCCACTTGGCAGTACATC
CMV_enh-005           ACGACCCCCGCCCATTGACGTCAATAATGACGTATGTTCCCATAGTAACGCCAATAGGGACTTTCCATTGACGTCAATGGGTGGACTATTTACGGTAAACTGCCCACTTGGCAGTACATC
CMV_enh-010           ACGACCCCCGCCCATTGACGTCAATAATGACGTATGTTCCCATAGTAACGCCAATAGGGACTTTCCATTGACGTCAATGGGTGGAGTATTTACGGTAAACTGCCCACTTGGCAGTACATC
CMV_enh-011           ACGACCCCCGCCCATTGACGTCAATAATGACGTATGTTCCCATAGTAACGCCAATAGGGACTTTCCATTGACGTCAATGGGTGGAGTATTTACGGTAAACTGCCCACTTGGCAGTACATC
CMV_enh-016           ACGACCCCCGCCCATTGACGTCAATAATGACGTATGTTCCCATAGTAACGCCAATAGGGACTTTCCATTGACGTCAATGGGTGGAGTATTTACGGTAAACTGCCCACTTGGCAGTACATC
CMV_enh-002           ACGACCCCCGCCCATTGACGTCAATAATGACGTATGTTCCCATAGTAACGCCAATAGGGACTTTCCATTGACGTCAATGGGTGGAGTATTTACGGTAAACTGCCCACTTGGCAGTACATC
CMV_enh-006           ACGACCCCCGCCCATTGACGTCAATAATGACGTATGTTCCCATAGTAACGCCAATAGGGACTTTCCATTGACGTCAATGGGTGGACTATTTACGGTAAACTGCCCACTTGGCAGTACATC
CMV_enh-013           ACGACCCCCGCCCATTGACGTCAATAATGACGTATGTTCCCATAGTAACGCCAATAGGGACTTTCCATTGACGTCAATGGGTGGAGTATTTACGGTAAACTGCCCACTTGGCAGTACATC
CMV_enh-012           ACGACCCCCGCCCATTGACGTCAATAATGACGTATGTTCCCATAGTAACGCCAATAGGGACTTTCCATTGACGTCAATGGGTGGAGTATTTACGGTAAACTGCCCACTTGGCAGTACATC
CMV_enh-004           ACGACCCCCGCCCATTGACGTCAATAATGACGTATGTTCCCATAGTAACGCCAATAGGGACTTTCCATTGACGTCAATGGGTGGAGTATTTACGGTAAACTGCCCACTTGGCAGTACATC
CMV_enh-007           ACGACCCCCGCCCATTGACGTCAATAATGACGTATGTTCCCATAGTAACGCCAATAGGGACTTTCCATTGACGTCAATGGGTGGAGTATTTACGGTAAACTGCCCACTTGGCAGTACATC
CMV_pro-007           ACGACCCCCGCCCATTGACGTCAATAATGACGTATGTTCCCATAGTAACGCCAATAGGGACTTTCCATTGACGTCAATGGGTGGAGTATTTACGGTAAACTGCCCACTTGGCAGTACATC
                      ************************************************************************************* **********************************

CMV_enh-003           AAGTGTATCATATGCCAAGTACGCCCCCTATTGACGTCAATGACGGTAAATGGCCCGC-CTGGCATTATGCCCAGTACATGACCTTATGGGACTTTCCTACTTGGCAGTACATCTACGTA
CMV_enh-015           AAGTGTATCATATGCCAAGTCCGCCCCCTATTGACGTCAATGACGGTAAATGGCCCGC-CTGGCATTATGCCCAGTACATGACCTTACGGGACTTTCCTACTTGGCAGTACATCTACGTA
CMV_enh-009           AAGTGTATCATATGCCAAGTACGCCCCCTATTGACGTCAATGACGGTAAATGGCCCGC-CTGGCATTATGCCCAGTACATGACCTTATGGGACTTTCCTACTTGGCAGTACATCTACGTA
CMV_enh-008           AAGTGTATCATATGCCAAGTACGCCCCCTATTGACGTCAATGACGGTAAATGGCCCGC-CTGGCATTATGCCCAGTACATGACCTTATGGGACTTTCCTACTTGGCAGTACATCTACGTA
CMV_enh-005           AAGTGTATCATATGCCAAGTACGCCCCCTATTGACGTCAATGACGGTAAATGGCCCGC-CTGGCATTATGCCCAGTACATGACCTTATGGGACTTTCCTACTTGGCAGTACATCTACGTA
CMV_enh-010           AAGTGTATCATATGCCAAGTCCGCCCCCTATTGACGTCAATGACGGTAAATGGCCCGC-CTGGCATTATGCCCAGTACATGACCTTACGGGACTTTCCTACTTGGCAGTACATCTACGTA
CMV_enh-011           AAGTGTATCATATGCCAAGTACGCCCCCTATTGACGTCAATGACGGTAAATGGCCCGC-CTGGCATTATGCCCAGTACATGACCTTACGGGACTTTCCTACTTGGCAGTACATCTACGTA
CMV_enh-016           AAGTGTATCATATGCCAAGTACGCCCCCTATTGACGTCAATGACGGTAAATGGCCCGC-CTGGCATTATGCCCAGTACATGACCTTATGGGACTTTCCTACTTGGCAGTACATCTACGTA
CMV_enh-002           AAGTGTATCATATGCCAAGTACGCCCCCTATTGACGTCAATGACGGTAAATGGCCCGCTCTGGCATTATGCCCAGTACATGACCTTATGGGACTTTCCTACTTGGCAGTACATCTACGTA
CMV_enh-006           AAGTGTATCATATGCCAAGTACGCCCCCTATTGACGTCAATGACGGTAAATGGCCCGC-CTGGCATTATGCCCAGTACATGACCTTATGGGACTTTCCTACTTGGCAGTACATCTACGTA
CMV_enh-013           AAGTGTATCATATGCCAAGTCCGCCCCCTATTGACGTCAATGACGGTAAATGGCCCGC-CTGGCATTATGCCCAGTACATGACCTTACGGGACTTTCCTACTTGGCAGTACATCTACGTA
CMV_enh-012           AAGTGTATCATATGCCAAGTACGCCCCCTATTGACGTCAATGACGGTAAATGGCCCGC-CTGGCATTATGCCCAGTACATGACCTTATGGGACTTTCCTACTTGGCAGTACATCTACGTA
CMV_enh-004           AAGTGTATCACATGCCAAGTACGCCCCCTATTGACGTCAATGACGGTAAATGGCCCGC-CTGGCATTATGCCCAGTACATGACCTTATGGGACTTTCCTACTTGGCAGTACATCTACGTA
CMV_enh-007           AAGTGTATCATATGCCAAGTCCGCCCCCTATTGACGTCAATGACGGTAAATGGCCCGC-CTGGCATTATGCCCAGTACATGACCTTACGGGACTTTCCTACTTGGCAGTACATCTACGTA
CMV_pro-007           AAGTGTATCATATGCCAAGTACGCCCCCTATTGACGTCAATGACGGTAAATGGCCCGC-CTGGCATTATGCCCAGTACATGACCTTATGGGACTTTCCTACTTGGCAGTACATCTACGTA
                      ********** ********* ************************************* *************************** *********************************

CMV_enh-003           TTAGTCATCGCTATTACCATG
CMV_enh-015           TTAGTCATCGCTATTACCATG
CMV_enh-009           TTAGTCATCGCTATTACCATG
CMV_enh-008           TTAGTCATCGCTATTACCATG
CMV_enh-005           TTAGTCATCGCTATTACCATG
CMV_enh-010           TTAGTCATCGCTATTACCATG
CMV_enh-011           TTAGTCATCGCTATTACCATG
CMV_enh-016           TTAGTCATCGCTATTACCATG
CMV_enh-002           TTAGTCATCGCTATTACCATG
CMV_enh-006           TTAGTCATCGCTATTACCATG
CMV_enh-013           TTAGTCATCGCTATTACCATG
CMV_enh-012           TTAGTCATCGCTATTACCATG
CMV_enh-004           TTAGTCATCGCTATTACCATA
CMV_enh-007           TTAGTCATCGCTATTACCATG
CMV_pro-007           TTAGTCATCGCTATTACCATGGTGATGCGGTTTTGGCAGTACATCAATGGGCGTGGATAG...
                      ********************
```

CMV_enh-001 is reverse complement of CMV_enh-003. CMV_enh-001 was merged with CMV_enh-003
CMV_enh-014 is reverse complement of CMV_enh-015. CMV_enh-014 was merged with CMV_enh-015
CMV_pro-007 is actually promoter plus enhancer. When split the enhancer segment is unique among the enhancers.

Key   ATGC = change in nucleotide relative to consensus sequence – non-coding sequence

Sequence Alignments

```
Name of      # of    Alignment                                                                                                                  Size     # Var    Sources*
Variant      Occur                                                                                                                              (bp)     bp
CMV_pro-004   338+2  ------------------------------------------------------------------------------------------------------------------------   204      0        Cl,In,Ag,Ev
CMV_pro-009   114    ------------------------------------------------------------------------------------------------------------------------   204      4        Or,Si,Pr
CMV_pro-008   19     ------------------------------------------------------------------------------------------------------------------------   199      0        Or
CMV_pro-010   12     ------------------------------------------------------------------------------------------------------------------------   204      1        Ox
CMV_pro-005   10     ------------------------------------------------------------------------------------------------------------------------   200      4        No
CMV_pro-006   10     ------------------------------------------------------------------------------------------------------------------------   204      1        Cl
CMV_pro-002   4      ------------------------------------------------------------------------------------------------------------------------   204      1        In
CMV_pro-007          GGAGTTCCGCGTTACATAACTTACGGTAAATGGCCCGCCTGGCTGACCGCCCAACGACCCCCGCCCATTGACGTCAATAATGACGTATGTTCCCATAGTAACGCCAATAGGGACTTTCCA   517      0        Cl
CMV_pro-001   1      ------------------------------------------------------------------------------------------------------------------------   204      7        Iv
CMV_pro-003   1      ------------------------------------------------------------------------------------------------------------------------   212      3/8i     Cl

CMV_pro-004          ------------------------------------------------------------------------------------------------------------------------
CMV_pro-009          ------------------------------------------------------------------------------------------------------------------------
CMV_pro-008          ------------------------------------------------------------------------------------------------------------------------
CMV_pro-010          ------------------------------------------------------------------------------------------------------------------------
CMV_pro-005          ------------------------------------------------------------------------------------------------------------------------
CMV_pro-006          ------------------------------------------------------------------------------------------------------------------------
CMV_pro-002          ------------------------------------------------------------------------------------------------------------------------
CMV_pro-007          TTGACGTCAATGGGTGGAGTATTTACGGTAAACTGCCCACTTGGCAGTACATCAAGTGTATCATATGCCAAGTACGCCCCCTATTGACGTCAATGACGGTAAATGGCCCGCCTGGCATTA
CMV_pro-001          ------------------------------------------------------------------------------------------------------------------------
CMV_pro-003          ------------------------------------------------------------------------------------------------------------------------

CMV_pro-004          -------------------------------------------------------------------------GTGATGCGGTTTTGGCAGTACATCAATGGGCGTGGATAGCGGTTTGA
CMV_pro-009          -------------------------------------------------------------------------GTGATGCGGTTTTGGCAGTACACCAATGGGCGTGGATAGCGGTTTGA
CMV_pro-008          --------------------------------------------------------------------------TGATGCGGTTTTGGCAGTACATCAATGGGCGTGGATAGCGGTTTGA
CMV_pro-010          -------------------------------------------------------------------------CTGATGCGGTTTTGGCAGTACATCAATGGGCGTGGATAGCGGTTTGA
CMV_pro-005          --------------------------------------------------------------------------TGATGCGGTTTTGGCAGTACACCAATGGGCGTGGATAGCGGTTTGA
CMV_pro-006          -------------------------------------------------------------------------GTGATGCGGTTTTGGCAGTACACCAATGGGCGTGGATAGCGGTTTGA
CMV_pro-002          -------------------------------------------------------------------------GTGATGCGGTTTTGGCAGTACATCAATGGGCGTGGATAGCGGTTTGA
CMV_pro-007          TGCCCAGTACATGACCTTATGGGACTTTCCTACTTGGCAGTACATCTACGTATTAGTCATCGCTATTACCATGGTGATGCGGTTTTGGCAGTACATCAATGGGCGTGGATAGCGGTTTGA
CMV_pro-001          -------------------------------------------------------------------------ATGATGCGGTTTTGGCAGTACATCAATGGGCGTGGATAGCGGTTTGA
CMV_pro-003          -------------------------------------------------------------------------GTGATGCGGTTTTGGCAGTACACCAATGGGCGTGGATAGCGGTTTGA
                                                                                               ********************* ************************

CMV_pro-004          CTCACGGGGATTTCCAAGTCTCCACCCCATTGACGTCAATGGGAGTTTGTTTTGGCACCAAAATCAACGGGACTTTCCAAAATGTCGTAACAAC--------TCCGCCCCATTGACGCAA
CMV_pro-009          CTCACGGGGATTTCCAAGTCTCCACCCCATTGACGTCAATGGGAGTTTGTTTTGGCACCAAAATCAACGGGACTTTCCAAAATGTCGTAATAAC--------CCCGCCCCGTTGACGCAA
CMV_pro-008          CTCACGGGGATTTCCAAGTCTCCACCCCATTGACGTCAATGGGAGTTTGTTTTGGCACCAAAATCAACGGGACTTTCCAAAATGTCGTAACAAC--------TCCGCCCCATTGACGCAA
CMV_pro-010          CTCACGGGGATTTCCAAGTCTCCACCCCATTGACGTCAATGGGAGTTTGTTTTGGCACCAAAATCAACGGGACTTTCCAAAATGTCGTAACAAC--------TCCGCCCCATTGACGCAA
CMV_pro-005          CTCACGGGGATTTCCAAGTCTCCACCCCATTGACGTCAATGGGAGTTTGTTTTGGCACCAAAATCAACGGGACTTTCCAAAATGTCGTAATAAC--------CCCGCCCCGTTGACGCAA
CMV_pro-006          CTCACGGGGATTTCCAAGTCTCCACCCCATTGACGTCAATGGGAGTTTGTTTTGGCACCAAAATCAACGGGACTTTCCAAAATGTCGTAACAAC--------TCCGCCCCATTGACGCAA
CMV_pro-002          CTCACGGGGATTTCCAAGTCTCCACCCCATTGACGTCAATGGGAGTTTGTTTTGGAACCAAAATCAACGGGACTTTCCAAAATGTCGTAACAAC--------TCCGCCCCATTGACGCAA
CMV_pro-007          CTCACGGGGATTTCCAAGTCTCCACCCCATTGACGTCAATGGGAGTTTGTTTTGGCACCAAAATCAACGGGACTTTCCAAAATGTCGTAACAAC--------TCCGCCCCATTGACGCAA
CMV_pro-001          CTCACGGGGATTTCCAAGTCTCCACCCCATTGACGTCAATGGGAGTTTGTTTTGACTAGTAAATCAACGGGACTTTCCAAAATGTCGTAACAAC--------TCCGCCCCATTGACGCAA
CMV_pro-003          CTCACGGGGATTTCCAAGTCTCCACCCCATTGACGTCAATGGGAGTTTGTTTTGGCACCAAAATCAACGGGACTTTCCAAAATGTCGTAACAACTGCGATCGCCCGCCCCGTTGACGCAA
                     ******************************************************      ****************************** ***         ******* *********

CMV_pro-004          ATGGGCGGTAGGCGTGTACGGTGGGAGGTCTATATAAGCAGAGCT
CMV_pro-009          ATGGGCGGTAGGCGTGTACGGTGGGAGGTCTATATAAGCAGAGCT
CMV_pro-008          ATGGGCGGTAGGCGTGTACGGTGGGAGGTCTATATAAGCAG----
CMV_pro-010          ATGGGCGGTAGGCGTGTACGGTGGGAGGTCTATATAAGCAGAGCT
CMV_pro-005          ATGGGCGGTAGGCGTGTACGGTGGGAGGTCTATATAAGCAGA---
CMV_pro-006          ATGGGCGGTAGGCGTGTACGGTGGGAGGTCTATATAAGCAGAGCT
CMV_pro-002          ATGGGCGGTAGGCGTGTACGGTGGGAGGTCTATATAAGCAGAGCT
CMV_pro-007          ATGGGCGGTAGGCGTGTACGGTGGGAGGTCTATATAAGCAGAGCT
CMV_pro-001          ATGGGCGGTAGGCGTGTACGGTGGGAGGTCTATATAAGCAGAGCT
CMV_pro-003          ATGGGCGGTAGGCGTGTACGGTGGGAGGTCTATATAAGCAGAGCT
                     *****************************************
```

CMV_pro-007 is actually promoter plus enhancer. When split the promoter segment is identical to CMV_pro-004.

Key   ATGC = change in nucleotide relative to consensus sequence – non-coding sequence

Sequence Alignments

Name of Variant	# of Occur	Alignment	Size (bp)	# Var bp	Sources*
SV40_ori-002	1	--	135	1	Or
SV40_ori-001	1	--	136	1	Lu
SV40_ori-003	139	--	136	1	In,Ne,GE,OB,Lu, etc
SV40_ori-006	8	--	136	1	Cl
SV40_ori-004	261+12	--	136	0	Cl,Or,Pr,Ev,Ag, etc
SV40_ori-007	5	--	136	1	Pr,Ne
SV40_prom-010	8	--	196	1/1i	Cl
SV40_prom-014	2	--	195	1	Ne
SV40_prom-008	10	--	197	0	Or,Cl,Pr
SV40_prom-013	5	--	197	1	Pr
SV40_prom-002	6	---GGTGTGGAAAGTCCCCAGGCTCCCCAG-CAGGCAGAAGTATGCAAAGCATGCATCTCAATTAGTCAGCAACCA	303	0	Si
SV40_prom-001	4	---GGTGTGGAAAGTCCCCAGGCTCCCCAG-CAGGCAGAAGTATGCAAAGCATGCATCTCAATTAGTCAGCAACCA	317	1	In
SV40_prom-006	1	---GGTGTGGAAAGTCCCCAGGCTCCCCAG-CAGGCAGAAGTATGCAAAGCATGCATCTCAATTAGTCAGCAACCA	317	0	?
SV40_prom-009	1	---GGTGTGGAAAGTCCCCAGGCTCCCCAG-CAGGCAGAAGTATGCAAAGCATGCATCTCAATTAGTCAGCAACCA	302	1/2d3i	Si
SV40_prom-015	3	---GGTGTGGAAAGTCCCCAGGCTCCCCAG-CAGGCAGAAGTATGCAAAGCATGCATCTCAATTAGTCAGCAACCA	299	1	Si
SV40_prom-005	94	----------------------------GTGTGTCAGTTAGGGTGTGGAAAGTCCCCAGGCTCCCCAG-CAGGCAGAAGTATGCAAAGCATGCATCTCAATTAGTCAGCAACCA	330	0	Or,Cl
SV40_prom-007	77	----------------------------GTGTGTCAGTTAGGGTGTGGAAAGTCCCCAGGCTCCCCAG-CAGGCAGAAGTATGCAAAGCATGCATCTCAATTAGTCAGCAACCA	298	1	In,Th,Cl,GE
SV40_prom-012	23	----------------------------GTGTGTCAGTTAGGGTGTGGAAAGTCCCCAGGCTCCCCAGGCAGGCAGAAGTATGCAAAGCATGCATCTCAATTAGTCAGCAACCA	298	1/1i	In,Ne
SV40_prom-003	1	CTGAGGCGGAAAGAACCAGCTGTGGAATGTGTGTCAGTTAGGGTGTGGAAAGTCCCCAGGCTCCCCAG-CAGGCAGAAGTATGCAAAGCATGCATCTCAATTAGTCAGCAACCA	358	1	Lu
SV40_prom-004	182	CTGAGGCGGAAAGAACCAGCTGTGGAATGTGTGTCAGTTAGGGTGTGGAAAGTCCCCAGGCTCCCCAG-CAGGCAGAAGTATGCAAAGCATGCATCTCAATTAGTCAGCAACCA	358	0	Cl,Ag,Ev,Pr,In,Or
SV40_prom-011	48	CTGAGGCGGAAAGAACCAGCTGTGGAATGTGTGTCAGTTAGGGTGTGGAAAGTCCCCAGGCTCCCCAG-CAGGCAGAAGTATGCAAAGCATGCATCTCAATTAGTCAGCAACCA	358	1	Pr,Lu
SR-alpha_prom	1	----------------------------GTGTGTCAGTTAGGGTGTGGAAAGTCCCCAGGCTCCCCAG-CAGGCAGAAGTATGCAAAGCATGCATCTCAATTAGTCAGCAACCA	615	1	?
SV40_enhancer	4	------------------GCTGTGGAATGTGTGTCAGTTAGGGTGTGGAAAGTCCCCAGGCTCCCCAG-CAGGCAGAAGTATGCAAAGCATGCATCTCAATTAGTCAGCAACCA	230	0	Pr

72 bp repeat II (enhancer)

SV40_ori-002	--TCCCGCCCC---TAACTC
SV40_ori-001	---ATCCCGCCCC---TAACTC
SV40_ori-003	---ATCCCGCCCC---TAACTC
SV40_ori-006	---ATCCCGCCCC---TAACTC
SV40_ori-004	---ATCCCGCCCC---TAACTC
SV40_ori-007	---ATCCCGCCCC---TAACTC
SV40_prom-010	---CAATTAGTCAGCAACCATAGTCCCGCCCCTAACTCCGCCCATCCCGCCCC---TAACTC
SV40_prom-014	---GCATCTCAATTAGTCAGCAACCATAGTCCCGCCCCTAACTCCGCCCATCCCGCCCC---TAACTC
SV40_prom-008	--TGCATCTCAATTAGTCAGCAACCATAGTCCCGCCCCTAACTCCGCCCATCCCGCCCC---TAACTC
SV40_prom-013	--TGCATCTCAATTAGTCAGCAACCATAGTCCCGCCCCTAACTCCGCCCATCCCGCCCC---TAACTC
SV40_prom-002	GGTGTGGAAAGTCCCCAGGCTCCCCAGCAGGCAGAAGTATGCAAAGCATGCATCTCAATTAGTCAGCAACCATAGTCCCGCCCCTAACTCCGCCCATCCCGCCCC---TAACTC
SV40_prom-001	GGTGTGGAAAGTCCCCAGGCTCCCCAGCAGGCAGAAGTATGCAAAGCATGCATCTCAATTAGTCAGCAACCATAGTCCCGCCCCTAACTCCGCCCATCCCGCCCC---TAACTC
SV40_prom-006	GGTGTGGAAAGTCCCCAGGCTCCCCAGCAGGCAGAAGTATGCAAAGCATGCATCTCAATTAGTCAGCAACCATAGTCCCGCCCCTAACTCCGCCCATCCCGCCCC---TAACTC
SV40_prom-009	GGTGTGGAAAGTCCCCAGGCTCCCCAGCAGGCAGAAGTATGCAAAGCATGCATCTCAATTAGTCAGCAACCATAGTCCCGCCCCTAACTCCGCCCATCCCGCCTCCGCTAACTC
SV40_prom-015	GGTGTGGAAAGTCCCCAGGCTCCCCAGCAGGCAGAAGTATGCAAAGCATGCATCTCAATTAGTCAGCAACCATAGTCCCGCCCCTAACTCCGCCCATCCCGCCTCCGCTAACTC
SV40_prom-005	GGTGTGGAAAGTCCCCAGGCTCCCCAGCAGGCAGAAGTATGCAAAGCATGCATCTCAATTAGTCAGCAACCATAGTCCCGCCCCTAACTCCGCCCATCCCGCCCC---TAACTC
SV40_prom-007	GGTGTGGAAAGTCCCCAGGCTCCCCAGCAGGCAGAAGTATGCAAAGCATGCATCTCAATTAGTCAGCAACCATAGTCCCGCCCCTAACTCCGCCCATCCCGCCCC---TAACTC
SV40_prom-012	GGTGTGGAAAGTCCCCAGGCTCCCCAGCAGGCAGAAGTATGCAAAGCATGCATCTCAATTAGTCAGCAACCATAGTCCCGCCCCTAACTCCGCCCATCCCGCCCC---TAACTC
SV40_prom-003	GGTGTGGAAAGTCCCCAGGCTCCCCAGCAGGCAGAAGTATGCAAAGCATGCATCTCAATTAGTCAGCAACCATAGTCCCGCCCCTAACTCCGCCCATCCCGCCCC---TAACTC
SV40_prom-004	GGTGTGGAAAGTCCCCAGGCTCCCCAGCAGGCAGAAGTATGCAAAGCATGCATCTCAATTAGTCAGCAACCATAGTCCCGCCCCTAACTCCGCCCATCCCGCCCC---TAACTC
SV40_prom-011	GGTGTGGAAAGTCCCCAGGCTCCCCAGCAGGCAGAAGTATGCAAAGCATGCATCTCAATTAGTCAGCAACCATAGTCCCGCCCCTAACTCCGCCCATCCCGCCCC---TAACTC
SR-alpha_prom	GGTGTGGAAAGTCCCCAGGCTCCCCAGCAGGCAGAAGTATGCAAAGCATGCATCTCAATTAGTCAGCAACCATAGTCCCGCCCCTAACTCCGCCCATCCCGCCCC---TAACTC
SV40_enhancer	GGTGTGGAAAGTCCCCAGGCTCCCCAGCAGGCAGAAGTATGCAAAGCATGCATCTCAATTAGTCAGCAACCATAGTCCCGCCCCTAACTCCGCCCATCCCGCCCC---TAACTC

72 bp repeat I (enhancer)

21 bp repeat III

21 bp repeat II

auxiliary SV40 ori

Sequence Alignments

```
SV40_ori-002      CGCCCAGTTCCGCCCATTCTCCGCCCCATGGCTGACTAATTTTTTTTATTTATGCAGAGGCCGAGGCCGCCTCGGCCTCTGAGCTATTCCAGAAGTAGTGAGGAGGCTTTTTTGGAGGCC------------------------------------
SV40_ori-001      CGCCCAGTTCCGCCCATTCTCCGCCCCTTGGCTGACTAATTTTTTTTATTTATGCAGAGGCCGAGGCCGCCTCGGCCTCTGAGCTATTCCAGAAGTAGTGAGGAGGCTTTTTTGGAGGCC------------------------------------
SV40_ori-003      CGCCCAGTTCCGCCCATTCTCCGCCCCATGGCTGACTAATTTTTTTTATTTATGCAGAGGCCGAGGCCGCCTCTGCCTCTGAGCTATTCCAGAAGTAGTGAGGAGGCTTTTTTGGAGGCC------------------------------------
SV40_ori-006      CGCCCAGTTCCGCCCATTCTCCGCCCCATGGCTGACTAATTTTTTTTATTTATGCAGAGGCCGAGGCCGCCTCGGCCTCTGAGCTATTCCAGAAGTAGTGAAGAGGCTTTTTTGGAGGCC------------------------------------
SV40_ori-004      CGCCCAGTTCCGCCCATTCTCCGCCCCATGGCTGACTAATTTTTTTTATTTATGCAGAGGCCGAGGCCGCCTCGGCCTCTGAGCTATTCCAGAAGTAGTGAGGAGGCTTTTTTGGAGGCC------------------------------------
SV40_ori-007      CGCCCAGTTCCGCCCATTCTCCGCCCCATCGCTGACTAATTTTTTTTATTTATGCAGAGGCCGAGGCCGCCTCGGCCTCTGAGCTATTCCAGAAGTAGTGAGGAGGCTTTTTTGGAGGCC------------------------------------
SV40_prom-010     CGCCCAGTTCCGCCCATTCTCCGCCCCATGGCTGACTAATTTTTTTTATTTATGCAGAGGCCGAGGCCGCCTCGGCCTCTGAGCTATTCCAGAAGTAGTGAAGAGGCTTTTTTGGAGGCCTAGGGCTTTTGCAAAAGCT-----------------
SV40_prom-014     CGCCCAGTTCCGCCCATTCTCCGCCCCATCGCTGACTAATTTTTTTTATTTATGCAGAGGCCGAGGCCGCCTCGGCCTCTGAGCTATTCCAGAAGTAGTGAGGAGGCTTTTTTGGAGGCCTAGG-CTTTTGCAA----------------------
SV40_prom-008     CGCCCAGTTCCGCCCATTCTCCGCCCCATGGCTGACTAATTTTTTTTATTTATGCAGAGGCCGAGGCCGCCTCGGCCTCTGAGCTATTCCAGAAGTAGTGAGGAGGCTTTTTTGGAGGCCTAGG-CTTTTGCAA----------------------
SV40_prom-013     CGCCCAGTTCCGCCCATTCTCCGCCCCATCGCTGACTAATTTTTTTTATTTATGCAGAGGCCGAGGCCGCCTCGGCCTCTGAGCTATTCCAGAAGTAGTGAGGAGGCTTTTTTGGAGGCCTAGG-CTTTTGCAA----------------------
SV40_prom-002     CGCCCAGTTCCGCCCATTCTCCGCCCCATGGCTGACTAATTTTTTTTATTTATGCAGAGGCCGAGGCCGCCTCGGCCTCTGAGCTATTCCAGAAGTAGTGAGGAGGCTTTTTTGGAGGCC------------------------------------
SV40_prom-001     CGCCCAGTTCCGCCCATTCTCCGCCCCATGGCTGACTAATTTTTTTTATTTATGCAGAGGCCGAGGCCGCCTCTGCCTCTGAGCTATTCCAGAAGTAGTGAGGAGGCTTTTTTGGAGGCCTAGG-CTTTTGCAAA---------------------
SV40_prom-006     CGCCCAGTTCCGCCCATTCTCCGCCCCATGGCTGACTAATTTTTTTTATTTATGCAGAGGCCGAGGCCGCCTCGGCCTCTGAGCTATTCCAGAAGTAGTGAGGAGGCTTTTTTGGAGGCCTAGG-CTTTTGCAAA---------------------
SV40_prom-009     CGCCCAGTTCCGCCCATTCTCCGCCCCATGGCTGACTAATTTTTTTTATTTATGCA--GGCCGAGGCCGCCTCGGCCTCTGAGCTATTCCAGAAGTAGTGAGGAGGCTTTTTTGGAGG--------------------------------------
SV40_prom-015     CGCCCAGTTCCGCCCATTCTCCGCCCCATGGCTGACTAATTTTTTTTATTTATGCA--GGCCGAGGCCGCCTCGGCCTCTGAGCTATTCCAGAAGTAGTGAGGAGGCTTTTTTGG-----------------------------------------
SV40_prom-005     CGCCCAGTTCCGCCCATTCTCCGCCCCATGGCTGACTAATTTTTTTTATTTATGCAGAGGCCGAGGCCGCCTCGGCCTCTGAGCTATTCCAGAAGTAGTGAGGAGGCTTTTTTGGAGGCCTAGG-CTTTTGCAAA---------------------
SV40_prom-007     CGCCCAGTTCCGCCCATTCTCCGCCCCATGGCTGACTAATTTTTTTTATTTATGCAGAGGCCGAGGCCGCCTCTGCCTCTGAGCTATTCCAGAAGTAGTGAGGAGGCTTTTTTGGAGGCCTAGG-CTTTTGCAAA---------------------
SV40_prom-012     CGCCCAGTTCCGCCCATTCTCCGCCCCATGGCTGACTAATTTTTTTTATTTATGCAGAGGCCGAGGCCGCCTCTGCCTCTGAGCTATTCCAGAAGTAGTGAGGAGGCTTTTTTGGAGGCCTAGG-CTTTTGCAAA---------------------
SV40_prom-003     CGCCCAGTTCCGCCCATTCTCCGCCCCTTGGCTGACTAATTTTTTTTATTTATGCAGAGGCCGAGGCCGCCTCGGCCTCTGAGCTATTCCAGAAGTAGTGAGGAGGCTTTTTTGGAGGCCTAGG-CTTTTGCAAA---------------------
SV40_prom-004     CGCCCAGTTCCGCCCATTCTCCGCCCCATGGCTGACTAATTTTTTTTATTTATGCAGAGGCCGAGGCCGCCTCGGCCTCTGAGCTATTCCAGAAGTAGTGAGGAGGCTTTTTTGGAGGCCTAGG-CTTTTGCAAA---------------------
SV40_prom-011     CGCCCAGTTCCGCCCATTCTCCGCCCCATGGCTGACTAATTTTTTTTATTTATGCAGAGGCCGAGGCCGCCTCTGCCTCTGAGCTATTCCAGAAGTAGTGAGGAGGCTTTTTTGGAGGCCTAGG-CTTTTGCAAA---------------------
SR-alpha_prom     CGCCCAGTTCCGCCCATTCTCCGCCCCATGGCTGACTAATTTTTTTTATTTATGCAGAGGCCGAGGCCGCCTCGGCCTCTGAGCTATTCCAGAAGTAGTGAGGAGGCTTTTTTGGAGGCCTAGG-CTTTTGCAAAAAGCTCCTCCGATCGAGG...
SV40_enhancer     CGCCCAGTTCCGCCCATTCTCCGC------------------------------------------------------------------------------------------------------------------------------------
```

21 bp repeat I — TATA box — TSS

auxiliary SV40 ori — core SV40 ori

Sequence structural elements are from Byrne et al. (1983) *Proc. Natl. Acad. Sci. USA* **80**:721-725.
Enhancer consists of 72 bp repeats
Promoter consists of 21 bp repeats and TATA box

SR-alpha promoter is a composite of the SV40 enhancer/promoter and the LTR from HTLV-1

SV40 ori-006 is reverse complement but unique
SV40 ori-005 is identical to SV40 ori-004 but reverse complement – SV40 ori-005 was merged with -004
SV40 enhancer is reverse complement

Key ATGC = change in nucleotide relative to consensus sequence – non-coding sequence
ATGC = HTLV-1 LTR

Sequence Alignments

```
Name of        # of     Alignment                                                                                                                                        Size    # Var    Sources*
Variant        Occur                                                                                                                                                     (bp)    bp
f1_ori-008     342      ACGCGCCCTGTAGCGGCGCATTAAGCGCGGCGGGTGTGGTGGTTACGCGCAGCGTGACCGCTACACTTGCCAGCGCCCTAGCGCCCGCTCCTTTCGCTTTCTTCCCTTCCTTTCTCGCCA                456     1        Cl,Ag,Or,Ev, etc
f1_ori-007     168      ACGCGCCCTGTAGCGGCGCATTAAGCGCGGCGGGTGTGGTGGTTACGCGCAGCGTGACCGCTACACTTGCCAGCGCCCTAGCGCCCGCTCCTTTCGCTTTCTTCCCTTCCTTTCTCGCCA                456     0        No,In,Cl
f1_ori-009     48       ACGCGCCCTGTAGCGGCGCATTAAGCGCGGCGGGTGTGGTGGTTACGCGCAGCGTGACCGCTACACTTGCCAGCGCCCTAGCGCCCGCTCCTTTCGCTTTCTTCCCTTCCTTTCTCGCCA                429     0        In,Or,Cl
f1_ori-003     34       ACGCGCCCTGTAGCGGCGCATTAAGCGCGGCGGGTGTGGTGGTTACGCGCAGCGTGACCGCTACACTTGCCAGCGCCCTAGCGCCCGCTCCTTTCGCTTTCTTCCCTTCCTTTCTCGCCA                429     4        In,Ne
f1_ori-006     19       ACGCGCCCTGTAGCGGCGCATTAAGCGCGGCGGGTGTGGTGGTTACGCGCAGCGTGACCGCTACACTTGCCAGCGCCCTAGCGCCCGCTCCTTTCGCTTTCTTCCCTTCCTTTCTCGCCA                456     2        BD,Cl,Ta
f1_ori-004     8        ACGCGCCCTGTAGCGCCCCATTAAGCGCGGCGGGTGTGGTGGTTACGCCCAGCGTGACCCCTACACTTCCCACCGCCCTAGCCCCCGCTCCTTTCGCTTTCTTCCCTTCCTTTCTCGCCA                456     20       In
M13_ori-003    6        ACGCGCCCTGTAGCGGCGCATTAAGCGCGGCGGGTGTGGTGGTTACGCGCAGCGTGACCGCTACACTTGCCAGCGCCCTAGCGCCCGCTCCTTTCGCTTTCTTCCCTTCCTTTCTCGCCA                510     1/1d     7669286
f1_ori-011     4        ACGCGCCCTGTAGCGGCGCATTAAGCGCGGCGGGTGTGGTGGTTACGCGCAGCGTGACCGCTACACTTGCCAGCGCCCTAGCGCCCGCTCCTTTCGCTTTCTTCCCTTCCTTTCTCGCCA                456     1        In
f1_ori-014     4        ACGCGCCCTGTAGCGGCGCATTAAGCGCGGCGGGTGTGGTGGTTACGCGCAGCGTGACCGCTACACTTGCCAGCGCCCTAGCGCCCGCTCCTTTCGCTTTCTTCCCTTCCTTTCTCGCCA                429     2        In,Mo
f1_ori-017     4        ACGCGCCCTGTAGCGGCGCATTAAGCGCGGCGGGTGTGGTGGTTACGCGCAGCGTGACCGCTACACTTGCCAGCGCCCTAGCGCCCGCTCCTTTCGCTTTCTTCCCTTCCTTTCTCGCCA                473     2/17i    Si
f1_ori-005     2        ACGCGCCCTGTAGCGGCGCATTAAGCGCGGCGGGTGTGGTGGTTACGCGCAGCGTGACCGCTACACTTGCCAGCGCCCTAGCGCCCGCTCCTTTCGCTTTCTTCCCTTCCTTTCTCGCCA                456     2        19906724
M13_ori-004    2        ACGCGCCCTGTAGCGGCGCATTAAGCGCGGCGGGTGTGGTGGTTACGCGCAGCGTGACCGCTACACTTGCCAGCGCCCTAGCGCCCGCTCCTTTCGCTTTCTTCCCTTCCTTTCTCGCCA                381     1        Ne
f1_ori-001     1        ACGCGCCCTGTAGCGGCGCATTAAGCGCGGCGGGTGTGGTGGTTACGCGCAGCGTGACCGCTACACTTGCCAGCGCCCTAGCGCCCGCTCCTTTCGCTTTCTTCCCTTCCTTTCTCGCCA                456     2        Pr
f1_ori-002     1        ACGCGCCCTGTAGCGGCGCATTAAGCGCGGCGGGTGTGGTGGTTACGCGCAGCGTGACCGCTACACTTGCCAGCGCCCTAGCGCCCGCTCCTTTCGCTTTCTTCCCTCCCTTTCTCGCCA                456     2        IMAGE
f1_ori-010     1        ACGCGCCCTGTAGCGGCGCATTAAGCGCGGCGGGTGTGGTGGTTACGCGCAGCGTGACCGCTACACTTGCCAGCGCCCTAGCGCCCGCTCCTTTCGCTTTCTTCCCTTCCTTTCTCGCCA                427     0/2d     In
f1_ori-012     1        ACGCGCCCTGTAGCGGCGCATTAAGCGCGGCGGGTGTGGTGGTTACGCGCAGCGTGACCGCTACACTTGCCAGCGCCCTAGCGCCCGCTCCTTTCGCTTTCTTCCCTTCCTTTCTCGCCA                456     2        Pr
f1_ori-013     1        ACGCGCCCTGTAGCGGCGCATTAAGCGCGGCGGGTGTGGTGGTTACGCGCAGCGTGACCGCTACACTTGCCAGCGCCCTAGCGCCCGCTCCTTTCGCTTTCTTCCCTTCCTTTCTCGCCA                459     2/3i     In
f1_ori-015     1        ACGCGCCCTGTAGCGGCGCATTAAGCGCGGCGGGTGTGGTGGTTACGCGCAGCGTGACCGCTACACTTGCCAGCGCCCTAGCGCCCGCTCCTTTCGCTTTCTTCCCTTCCTTTCTCGCCA                456     5        In
f1_ori-016     1        ACGCGCCCTGTAGCGGCGCATTAAGCGCGGCGGGTGTGGTGGTTACGCGCAGCGTGACCGCTACACTTGCCAGCGCCCTAGCGCCCGCTCCTTTCGCTTTCTTCCCTTCCTTTCTCGCCA                459     1/3i     MCSG
M13_ori-001    1        ACGCGCCCTGTAGCGGCGCATTAAGCGCGGCGGGTGTGGTGGTTACGCGCAGCGTGACCGCTACACTTGCCAGCGCCTTAGCGCCCGCTCCTTTCGCTTTCTTCCCTTCCTTTCTCGCCA                456     2        3323803
M13_ori-002    1        ACGCGCCCTGTAGCGGCGCATTAAGCGCG-CGGGTGTGGTGGTTACGCGCAGCGTGACCGCTACACTTGCCAGCGCCCTAGCGCCCGCTCCTTTCGCTTTCTTCCCTTCCTTTCTCGCCA                380     5        In
M13_ori-005    1        ACGCGCCCTGTAGCGGCGCATTAAGCGCGGCGGGTGTGGTGGTTACGCGCAGCGTGACCGCTACACTTGCCAGCGCCCTAGCGCCCGCTCCTTTCGCTTTCTTCCCTTCCTTTCTCGCCA                381     4        Ca
                        *************** * *********** ****************** ********** ******** *** **** **** ************************ ************

f1_ori-008              CGTTCGCCGGCTTTCCCCGTCAAGCTCTAAATCGGGGGCTCCCTTTAGGGTTCCGATTTAGTGCTTTACGGCACCTCGACCCCAAAAAACTTGATTAGGGTGATGGTTCACGTAGTGGGC
f1_ori-007              CGTTCGCCGGCTTTCCCCGTCAAGCTCTAAATCGGGGGCTCCCTTTAGGGTTCCGATTTAGTGCTTTACGGCACCTCGACCCCAAAAAACTTGATTAGGGTGATGGTTCACGTAGTGGGC
f1_ori-009              CGTTCGCCGGCTTTCCCCGTCAAGCTCTAAATCGGGGGCTCCCTTTAGGGTTCCGATTTAGTGCTTTACGGCACCTCGACCCCAAAAAACTTGATTAGGGTGATGGTTCACGTAGTGGGC
f1_ori-003              CGTTCGCCGGCTTTCCCCGTCAAGCTCTAAATCGGGGCATCCCTTTAGGGTTCCGATTTAGTGCTTTACGGCACCTCGACCCCAAAAAACTTGATTAGGGTGATGGTTCACGTAGTGGGC
f1_ori-006              CGTTCGCCGGCTTTCCCCGTCAAGCTCTAAATCGGGGGCTCCCTTTAGGGTTCCGATTTAGTGCTTTACGGCACCTCGACCCCAAAAAACTTGATTTGGGTGATGGTTCACGTAGTGGGC
f1_ori-004              CGTTCGCCGGCTTTCCCCGTCAAGCTCTAAATGGGGGCATCCGTTTACCCTTCCGATTTACTGCTTTACGGCACCTCGACCCCAAAAAACTTGATTAGGGTGATGGTTCACGTAGTGGGC
M13_ori-003             CGTTCGC----TTTCCCCGTCAAGCTCTAAATCGGGGGCTCCCTTTAGGGTTCCGATTTAGTGCTTTACGGCACCTCGACCCCAAAAAACTTGATTTGGGTGATGGTTCACGTAGTGGGC
f1_ori-011              CGTTCGCCGGCTTTCCCCGTCAAGCTCTAAATCGGGGGCTCCCTTTAGGGTTCCGATTTAGTGCTTTACGGCACCTCGACCCCAAAAAACTTGATTAGGGTGATGGTTCACGTAGTGGGC
f1_ori-014              CGTTCGCCGGCTTTCCCCGTCAAGCTCTAAATCGGGGCATCCCTTTAGGGTTCCGATTTAGTGCTTTACGGCACCTCGACCCCAAAAAACTTGATTAGGGTGATGGTTCACGTAGTGGGC
f1_ori-017              CGTTCGCCGGCTTTCCCCGTCAAGCTCTAAATCGGGGGCTCCCTTTAGGGTTCCGATTTAGTGCTTTACGGCACCTCGACCCCAAAAAACTTGATTAGGGTGATGGTTCACGTAGTGGGC
f1_ori-005              CGTTCGCCGGCTTTCCCCGTCAAGCTCTAAATCGGGGGCTCCCTTTAGGGTTCCGATTTAGTGCTTTACGGCACCTCGACCCCAAAAAACTTGATTAGGGTGATGGTTCACGTAGTGGGC
M13_ori-004             CGTTCGCCGGCTTTCCCCGTCAAGCTCTAAATCGGGGGCTCCCTTTAGGGTTCCGATTTAGTGCTTTACGGCACCTCGACCCCAAAAAACTTGATTTGGGTGATGGTTCACGTAGTGGGC
f1_ori-001              CGTTCGCGGGCTTTCCCCGTCAAGCTCTAAATCGGGGGCTCCCTTTAGGGTTCCGATTTAGTGCTTTACGGCACCTCGACCCCAAAAAACTTGATTAGGGTGATGGTTCACGTAGTGGGC
f1_ori-002              CGTTCGCCGGCTTTCCCCGTCAAGCTCTAAATCGGGGGCTCCCTTTAGGGTTCCGATTTAGTGCTTTACGGCACCTCGACCCCAAAAAACTTGATTAGGGTGATGGTTCACGTAGTGGGC
f1_ori-010              CGTTCGCCGGCTTTCCC-GTCAAGCTCTAAATCGGGGGCTCCCTTTAGGGTTCCGATTTAGTGCTTTACGGCACCTCGACCCCAAAAA-CTTGATTAGGGTGATGGTTCACGTAGTGGGC
f1_ori-012              CGTTCGCCGGCTTTCCCCGTCAAGCTCTAAATCGGGGGCTCCCTTTAGGGTTCCGATTTAGTGCTTTACGGCACCTCGACCGCAAAAAACTTGATTAGGGTGATGGTTCACGTAGTGGGC
f1_ori-013              CGTTCGCCGGCTTTCCCCGTCAAGCTCTAAATCGGGGGCTCCCTTTAGGGTTCCGATTTAGTGCTTTACGGCACCTCGACCCCAAAAAACTTGATTTGGGTGATGGTTCACGTAGTGGGC
f1_ori-015              CGTTCGCCGGCTTTCCCCGTCAAGCTCTAAATCGGGGGCTCCCTTTAGGGTTCCGATTTAGAGCTTTACGGCACCTCGACCGCAAAAAACTTGATTTGGGTGATGGTTCACGTAGTGGGC
f1_ori-016              CGTTCGCCGGCTTTCCCCGTCAAGCTCTAAATCGGGGGCTCCCTTTAGGGTTCCGATTTAGTGCTTTACGGCACCTCGACCCCAAAAAACTTGATTAGGGTGATGGTTCACGTAGTGGGC
M13_ori-001             CGTTCGCCGGCTTTCCCCGTCAAGCTCTAAATCGGGGGCTCCCTTTAGGGTTCCGATTTAGTGCTTTACGGCACCTCGACCCCAAAAAACTTGATTTGGGTGATGGTTCACGTAGTGGGC
M13_ori-002             CGTTCGCCGGCTTTCCCCGTCAAGCTCTAAATCGGGGGCTCCCTTTAGGGTTCCGATTTAGAGCTTTACGGCACCTCGACCGCAAAAAACTTGATTTGGGTGATGGTTCACGTAGTGGGC
M13_ori-005             CGTTCGCCGGCTTTCCCCGTCAAGCTCTAAATCGGGGGCTCCCTTTAGGGTTCCGATTTAGAGCTTTACGGCACCTCGACCGCAAAAAACTTGATTTGGGTGATGGTTCACGTAGTGGGC
                        *******    ****** ************** ****  *** ****   **********  ******************* ****** ******* ***********************
                                              Stem-Loop B                                                   T=M13     Stem-Loop C

f1_ori-008              ---CATCGCCCTGATAGACGGTTTTTCGCCCTTTGACGTTGGAGTCCACGTTCTTTAATAGT-----------------GGACTCTTGTTCCAAACTGGAACAACACTCAACCCTATCTC
f1_ori-007              ---CATCGCCCTGATAGACGGTTTTTCGCCCTTTGACGTTGGAGTCCACGTTCTTTAATAGT-----------------GGACTCTTGTTCCAAACTGGAACAACACTCAACCCTATCTC
f1_ori-009              ---CATCGCCCTGATAGACGGTTTTTCGCCCTTTGACGTTGGAGTCCACGTTCTTTAATAGT-----------------GGACTCTTGTTCCAAACTGGAACAACACTCAACCCTATCTC
f1_ori-003              ---CATCGCCCTGATAGACGGTTTTTCGCCCTTTGACGTTGGAGTCCACGTTCTTTAATAGT-----------------GGACTCTTGTTCCAAACTGGAACAACACTCAACCCTATCTC
f1_ori-006              ---CATCGCCCTGATAGACGGTTTTTCGCCCTTTGACGTTGGAGTCCACGTTCTTTAATAGT-----------------GGACTCTTGTTCCAAACTGGAACAACACTCAACCCTATCTC
f1_ori-004              ---CATCGCCCTGATAGACCCTTTTTCGCCCTTTGACGTTGGAGTCCACGTTCTTTAATAGT-----------------GGACTCTTGTTGGAAACTGGAACAACACTCAACCCTATCTC
M13_ori-003             ---CATCGCCCTGATAGACGGTTTTTCGCCCTTTGACGTTGGAGTCCACGTTCTTTAATAGT-----------------GGACTCTTGTTCCAAACTGGAACAACACTCAACCCTATCTC
f1_ori-011              ---CATCGCCCTGATAGACGGTTTTTCGCCCTTTGACGTTGGAGTCCACGTTCTTTAATAGT-----------------GGACTCTTGTTCCAAACTGGAACAACACTCAACCCTATCTC
f1_ori-014              ---CATCGCCCTGATAGACGGTTTTTCGCCCTTTGACGTTGGAGTCCACGTTCTTTAATAGT-----------------GGACTCTTGTTCCAAACTGGAACAACACTCAACCCTATCTC
f1_ori-017              ---CATCGCCCTGATAGACGGTTTTTCGCCCTTTGACGTTGGAGTCCACGTTCTTTAAGAGTCCACTATTAAAGAACGTGGACTCTTGTTCCAAACTGGAACAACACTCAACCCTATCTC
f1_ori-005              ---CATCGCCCTGATAGACGGTTTTTCGCCCTTTGACGTTGGAGTCCACGTTCTTTAATAGT-----------------GGACTCTTGTTCCAAACTGGAACAACACTCAACCCTATCTC
M13_ori-004             ---CATCGCCCTGATAGACGGTTTTTCGCCCTTTGACGTTGGAGTCCACGTTCTTTAATAGT-----------------GGACTCTTGTTCCAAACTGGAACAACACTCAACCCTATCTC
f1_ori-001              ---CATCGCCCTGATAGACGGTTTTTCGCCCTTTGACGTTGGAGTCCACGTTCTTTAATAGT-----------------GGACTCTTGTTCCAAACTGGAACAACACTCAACCCTATCTC
f1_ori-002              ---CATCGCCCTGATAGACGGTTTTTCGCCCTTTGACGTTGGAGTCCACGTTCTTTAATAGT-----------------GGACTCTTGTTCCAAACTGGAACAACACTCAACCCTATCTC
f1_ori-010              ---CATCGCCCTGATAGACGGTTTTTCGCCCTTTGACGTTGGAGTCCACGTTCTTTAATAGT-----------------GGACTCTTGTTCCAAACTGGAACAACACTCAACCCTATCTC
f1_ori-012              ---CATCGCCCTGATAGACGGTTTTTCGCCCTTTGACGTTGGAGTCCACGTTCTTTAATAGT-----------------GGACTCTTGTTCCAAACTGGAACAACACTCAACCCTATCTC
f1_ori-013              GGCCATCGCCCTGATAGACGGTTTTTCGCCCTTTGACGTTGGAGTCCACGTTCTTTAATAGT-----------------GGACTCTTGTTCCAAACTGGAACAACACTCAACCCTATCTC
f1_ori-015              ---CATCGCCCTGATAGACGGTTTTTCGCCCTTTGACGTTGGAGTCCACGTTCTTTAATAGT-----------------GGACTCTTGTTCCAAACTGGAACAACACTCAACCCTATCGC
f1_ori-016              ---CATCGCCCTGATAGACGGTTTTTCGCCCTTTGACGTTGGAGTCCACGTTCTTTAATAGT-----------------GGACTCTTGTTCCAAACTGGAACAACACTCAACCCTATCTC
M13_ori-001             ---CATCGCCCTGATAGACGGTTTTTCGCCCTTTGACGTTGGAGTCCACGTTCTTTAATAGT-----------------GGACTCTTGTTCCAAACTGGAACAACACTCAACTCTATCTC
M13_ori-002             ---CATCGCCCTGATAGACGGTTTTTCGCCCTTTGACGTTGGAGTCCACGTTCTTTAATAGT-----------------GGACTCTTGTTCCAAACTGGAACAACACTCAACCCTATCGC
M13_ori-005             ---CATCGCCCTGATAGACGGTTTTTCGCCCTTTGACGTTGGAGTCCACGTTCTTTAATAGT-----------------GGACTCTTGTTCCAAACTGGAACAACACTCAACCCTATCGC
                           ****************  ************************************* ***                 ***********  ******************** ***** *
                           Primer RNA (- strand synthesis)                           Stem-Loop D                   Stem-Loop E
                                                                 Gene II nick site (+ strand initiation)
```

Sequence Alignments

```
f1_ori-008          GGTCTATTCTTTTGATTTATAAGGGATTTTGCCGATTTCGGCCTATTGGTTAAAAAATGAGCTGATTTAACAAAAATTTAACGCGAATTTTAACAAAAT---ATTAACGCTTACAATTT-
f1_ori-007          GGTCTATTCTTTTGATTTATAAGGGATTTTGCCGATTTCGGCCTATTGGTTAAAAAATGAGCTGATTTAACAAAAATTTAACGCGAATTTTAACAAAAT---ATTAACGTTTACAATTT-
f1_ori-009          GGTCTATTCTTTTGATTTATAAGGGATTTTGCCGATTTCGGCCTATTGGTTAAAAAATGAGCTGATTTAACAAAAATTTAACGCGAATT-------------------------------
f1_ori-003          GGTCTATTCTTTTGATTTATAAGGGATTTTGGGGATTTCGGCCTATTGGTTAAAAAATGAGCTGATTTAACAAAAATTTAACGCGAATT-------------------------------
f1_ori-006          GGGCTATTCTTTTGATTTATAAGGGATTTTGCCGATTTCGGCCTATTGGTTAAAAAATGAGCTGATTTAACAAAAATTTAACGCGAATTTTAACAAAAT---ATTAACGTTTACAATTT-
f1_ori-004          GGTCTATTCTTTTGATTTATAAGGGATTTTGCCGATTTCGGGCTATTCGTTAAAAAATGAGCTGATTTAACAAAAATTTAACGCGAATTTTAACAAAAT---ATTAACGTTTACAATTT-
M13_ori-003         GGGCTATTCTTTTGATTTATAAGGGATTTTGCCGATTTCGGCCTATTGGTTAAAAAATGAGCTGATTTAACAAAAATTTAACGCGAATTTTAACAAAAT---ATTAACGTTTACAATTTA
f1_ori-011          GGTCTATTCTTTTGATTTATAAGGGATTTTGCCGATTTCGGCCTATTGGTTAAAAAATGAGCTGATTTAACAAATATTTAACGCGAATTTTAACAAAAT---ATTAACGTTTACAATTT-
f1_ori-014          GGTCTATTCTTTTGATTTATAAGGGATTTTGCCGATTTCGGCCTATTGGTTAAAAAATGAGCTGATTTAACAAAAATTTAACGCGAATT-------------------------------
f1_ori-017          GGTCTATTCTTTTGATTTATAAGGGATTTTGCCGATTTCGGCCTATTGGTTAAAAAATGAGCTGATTTAACAAAAATTTAACGCGAATTTTAACAAAAT---ATTAACGCTTACAATTT-
f1_ori-005          GGTCTATTCTTTTGATTTATAAGGGATTTTGCCGATTTCGGCCTATTGGTTAAAAAATGAGCTGATTTAACAAAAATTTAACGCGAATTTTAACAAACT---AGTAACGTTTACAATTT-
M13_ori-004         GGGCTATTCTTTTGATTTATAAGGGATTTTGCCGATTTCGG------------------------------------------------------------------------------
f1_ori-001          GGTCTATTCTTTTGATTTATAAGGGATTTTGCCGATTTCGGCCTATTGGTTAAAAAATGAGCTGATTTAACAAAAATTTAACGCGAATTTTAACAAAAT---ATTAACGCTTACAATTT-
f1_ori-002          GGTCTATTCTTTTGATTTATAAGGGATTTTGCCGATTTCGGCCTATTGGTTAAAAAATGAGCTGATTTAACAAAAATTTAACGCGAATTTTAACAAAAT---ATTAACGCTTACAATTT-
f1_ori-010          GGTCTATTCTTTTGATTTATAAGGGATTTTGCCGATTTCGGCCTATTGGTTAAAAAATGAGCTGATTTAACAAAAATTTAACGCGAATT-------------------------------
f1_ori-012          GGTCTATTCTTTTGATTTATAAGGGATTTTGCCGATTTCGGCCTATTGGTTAAAAAATGAGCTGATTTAACAAAAATTTAACGCGAATTTTAACAAAAT---ATTAACGCTTACAATTT-
f1_ori-013          GGGCTATTCTTTTGATTTATAAGGGATTTTGCCGATTTCGGCCTATTGGTTAAAAAATGAGCTGATTTAACAAAAATTTAACGCGAATTTTAACAAAAT---ATTAACGTTTACAATTT-
f1_ori-015          GGTCTATTCTTTTGATTTATAAGGGATTTTGCCGATTTCGGCCTATTGGTTAAAAAATGAGCTGATTTAACAAATATTTAACGCGAATTTTAACAAAAT---ATTAACGTTTACAATTT-
f1_ori-016          GGTCTATTCTTTTGATTTATAAGGGATTTTGCCGATTTCGGCCTATTGGTTAAAAAATGAGCTGATTTAACAAAAATTTAACGCGAATTTTAACAAAATCACATTAACGCTTACAATTT-
M13_ori-001         GGGCTATTCTTTTGATTTATAAGGGATTTTGCCGATTTCGGTCTATTGGTTAAAAAATGAGCTGATTTAACAAAAATTTAACGCGAATTTTAACAAAAT---ATTAACGTTTACAATTT-
M13_ori-002         GGTCTATTCTTTTGATTTATAAGGGATGTTGCCGATTTCGG------------------------------------------------------------------------------
M13_ori-005         GGTCTATTCTTTTGATTTATAAGGGATTTTGCCGATTTCGG------------------------------------------------------------------------------
                    ** ************************ ***  ********
```

Domain B (AT-rich enhancer of + strand synthesis)

```
f1_ori-008          ---------------------------------------------------------
f1_ori-007          ---------------------------------------------------------
f1_ori-009          ---------------------------------------------------------
f1_ori-003          ---------------------------------------------------------
f1_ori-006          ---------------------------------------------------------
f1_ori-004          ---------------------------------------------------------
M13_ori-003         AATATTTGCTTATACAATCTTCCTGTTTTTGGGGCTTTTCTGATTATCAACCGGGGT
f1_ori-011          ---------------------------------------------------------
f1_ori-014          ---------------------------------------------------------
f1_ori-017          ---------------------------------------------------------
f1_ori-005          ---------------------------------------------------------
M13_ori-004         ---------------------------------------------------------
f1_ori-001          ---------------------------------------------------------
f1_ori-002          ---------------------------------------------------------
f1_ori-010          ---------------------------------------------------------
f1_ori-012          ---------------------------------------------------------
f1_ori-013          ---------------------------------------------------------
f1_ori-015          ---------------------------------------------------------
f1_ori-016          ---------------------------------------------------------
M13_ori-001         ---------------------------------------------------------
M13_ori-002         ---------------------------------------------------------
M13_ori-005         ---------------------------------------------------------
```

Structures are from Dotto et al. (1984) *J. Mol. Biol.* **172:**507-521 and from Baas (1985) *Biochimica et Biophysica Acta* **825:**11-139.

Yellow highlights indicate nucleotides that form bulges or loops in the indicated stem-loop structures formed when the DNA is single-stranded.
f1 ori variants -006, -013 and -015 are actually M13 ori as the two origins differ only by an A or T at the indicated position.

If the sequences of variants -013 and -017 are correct, these origins are almost certainly compromised or devoid of minus strand and plus strand synthesis, respectively.

Key ATGC = change in nucleotide relative to consensus sequence – non-coding sequence

Sequence Alignments

Name of Variant	# of Occur	Alignment (SL3, SL2, α-stem)	Size (bp)	# Var bp	Sources*
ori-014	602	TTGAGATCCTTTTTTTCTGCGCGTAATCTGCTGCTTGCAAACAAAAAAACCACCGCTACCAGCGGTGGTTTGTTTGCCGGATCAAGAGCTACCAACTCTTTTTCCGAAGGTAACTGGCTT	589	1	No,Cl,In,Ca,Ev, etc
ori-013	524	TTGAGATCCTTTTTTTCTGCGCGTAATCTGCTGCTTGCAAACAAAAAAACCACCGCTACCAGCGGTGGTTTGTTTGCCGGATCAAGAGCTACCAACTCTTTTTCCGAAGGTAACTGGCTT	589	0	Cl,In,Or,Pr,Ox, etc
ori-009	220	TTGAGATCCTTTTTTTCTGCGCGTAATCTGCTGCTTGCAAACAAAAAAACCACCGCTACCAGCGGTGGTTTGTTTGCCGGATCAAGAGCTACCAACTCTTTTTCCGAAGGTAACTGGCTT	589	3	In,Qi,MBL,Ne, etc
ori-012	55	TTGAGATCCTTTTTTTCTGCGCGTAATCTGCTGCTTGCAAACAAAAAA---ACCGCTACCAGCGGTGGTTTGTTTGCCGGATCAAGAGCTACCAACTCTTTTTCCGAAGGTAACTGGCTT	585	0/4d	Or,Cl,In,GE
ori-008	19	TTGAGATCCTTTTTTTCTGCGCGTAATCTGCTGCTTGCAAACAAAAAAACCACCGCTACCAGCGGTGGTTTGTTTGCCGGATCAAGAGCTACCAACTCTTTTTCCGAAGGTAACTGGCTT	589	2	No,Qi,GE
ori-003	15	TTGAGATCCTTTTTTTCTGCGCGTAATCTGCTGCTTGCAAACAAAAAAACCACCGCTACCAGCGGTGGTTTGTTTGCCGGATCAAGAGCTACCAACTCTTTTTCCGAAGGTAACTGGCTT	589	1	Th
ori-021	12	TTGAGATCCTTTTTTTCTGCGCGTAATCTGCTACTTGCAAACAAAAAAACCACCGCTACCAGCGGTGGTTTGTTTGCCGGATCAAGAGCTACCAACTCTTTTTCCGAAGGTAACTGGCTT	589	4	24050148
ori-002	8	TTGAGATCCTTTTTTTCTGCGCGTAATCTGCTGCTTGCAAACAAAAAAACCACCGCTACCAGCGGTGGTTTGTTTGCCGGATCAAGAGCTACCAACTCTTTTTCCGA-GGTAACTGGCTT	588	1/1d	Lu
ori-016	8	TTGAGATCCTTTTTTTCTGCGCGTAATCTGCTGCTTGCAAACAAAAAAACCACCGCTACCAGCGGTGGTTTGTTTGCCGGATCAAGAGCTACCAACTCTTTTTCCGAAGGTAACTGGCTT	589	4	2659436
ori-010	5	TTGAGATCCTTTTTTTCTGCGCGTAATCTGCTGCTTGCAAACAAAAAAACCACCGCTACCAGCGGTGGTTTGTTTGCCGGATCAAGAGCTACCAACTCTTCTTCCGAAGGTAACTGGCTT	589	1	10890530
ori-011	5	TTGAGATCCTTTTTTTCTGCGCGTAATCTGCTGCTTGCAAACAAAAAAACCACCGCTACCAGCGGTGGTTTGTTTGCCGGATCAAGAGCTACCAACTCTTTTTCCGAAGGTAACTGGCTT	588	0/1d	Qi,No
ori-005	4	TTGAAATCCTTTTTTTCTGCGCGTAATCTGCTGCTTGCAAACAAAAAAACCACCGCTACCAGCGGTGGTTTGTTTGCCGGATCAAGAGCTACCAACTCTTTTTCCGAAGGTAACTGGCTT	589	2	Pr
ori-018	4	TTGATATCCTTTTTTTCTGCGCGTAATCTGCTGCTTGCAAACAAAAAAACCACCGCTACCAGCGGTGGTTTGTTTGCCGGATCAAGAGCTACCAACTCTTTTTCCGAAGGTAACTGGCTT	589	4	15644173
ori-001	1	TTGAGATCCTTTTTTTCTGCGCGTAATCTGCTGCTTGCAAACAAAAAAACCACCGCTACCAGCGGTGGTTTGTTTGCCGGATCAAGAGCTACCAACTCTTTTTCCGAAGGTAACTGGCTT	589	1	Or
ori-004	1	TTGAGATCCTTTTTTTCTGCGCGTAATCTGCTGCTTGCAAACAAAAAAACCACCGCTACCAGCGGTGGTTTGTTTGCCGGATCAAGAGCTACCAACTCTTTTTCCGAAGGTAACTGGCTT	589	2	IMAGE
ori-006	1	TTGAGATCCTTTTTTTCTGCGCGTAATCTGCTGCTTGCAAACAAAAAAACCACCGCTACCAGCGGTGGTTTGTTTGCCGGATCAAGAGCTACCAACTCTTTTTCCGAAGGTAACTGGCTT	589	1	iGEM
ori-007	1	TTGAGATCCTTTTTTTCTGCGCGTAATCTGCTGCTTGCAAACAAAAAA-CCACCGCTACCAGCGGTGGTTTGTTTGCCGGATCAAGAGCTACCAACTCTTTTTCCGAAGGTAACTGGCTT	588	0/1d	Or
ori-015	1	TTGAGATCCTTTTTTTCTGCGCGTAATCTGCTGCTTGCAAACAAAAAACCAACCGCTACCAGCGGTGGTTTGTTTGCCGGATCAAGAGCTACCAACTCTTTTTCCGAAGGTAACTGGCTT	589	2	In
ori-017	1	--------------------GCGTAATCTGCTGCTTGCAAACAAAAAAACCACCGCTACCAGCGGTGGTTTGTTTGCCGGATCAAGAGCTACCAACTCTTTTTCCGAAGGTAACTGGCTT	569	3	In
ori-019	1	TTGAGATCCTTTTTTTCTGCGCGTAATCTGCTGCTTGCAAACAAAAAAACCACCGCTACCAGCGGTGGTTTGTTTGCCGGATCAAGAGCTACCAACTCTTTTTCCGAAGGTAACTGGCTT	583	2/6d	No
ori-020	1	TTGAGATCCTTTTTTTCTGCGCGTAATCTGCTGCTTGCAAACAAAAAAACCACCGCTACCAGCGGTGGTTTGTTTGCCGGATCAAGAGCTACCAACTCTTTTTCCGAAGGTAACTGGCTT	589	1	Cl
pBR322ori-t	1	--	353	0	7655517

-35 RNAII prom -10

RNAII

RNAI

Name of Variant	Alignment (SL1, β-stem)
ori-014	CAGCAGAGCGCAGATACCAAATACTGTCCTTCTAGTGTAGCCGTAGTTAGGCCACCACTTCAAGAACTCTGTAGCACCGCCTACATACCTCGCTCTGCTAATCCTGTTACCAGTGGCTGC
ori-013	CAGCAGAGCGCAGATACCAAATACTGTTCTTCTAGTGTAGCCGTAGTTAGGCCACCACTTCAAGAACTCTGTAGCACCGCCTACATACCTCGCTCTGCTAATCCTGTTACCAGTGGCTGC
ori-009	CAGCAGAGCGCAGATACCAAATACTGTCCTTCTAGTGTAGCCGTAGTTAGGCCACCACTTCAAGAACTCTGTAGCACCGCCTACATACCTCGCTCTGCTAATCCTGTTACCAGTGGCTGC
ori-012	CAGCAGAGCGCAGATACCAAATACTGTTCTTCTAGTGTAGCCGTAGTTAGGCCACCACTTCAAGAACTCTGTAGCACCGCCTACATACCTCGCTCTGCTAATCCTGTTACCAGTGGCTGC
ori-008	CAGCAGAGCGCAGATACCAAATACTGTTCTTCTAGTGTAGCCGTAGTTAGGCCACCACTTCAAGAACTCTGTAGCACCGCCTACATACCTCGCTCTGCTAATCCTGTTACCAGTGGCTGC
ori-003	CAGCAGAGCGCAGATACCAAATACTGTTCTTCTAGTGTAGCCGTAGTTAGCCCACCACTTCAAGAACTCTGTAGCACCGCCTACATACCTCGCTCTGCTAATCCTGTTACCAGTGGCTGC
ori-021	TAGCAGAGCGCAGATACCAAATACTGTCCTTCTAGTGTAGCCGTAGTTAGGCCACCACTTCAAGAACTCTGTAGCACCGCCTACATACCTCGCTCTGCTAATCCTGTTACCAGTGGCTGC
ori-002	CAGCAGAGCGCAGATACCAAATACTGTTCTTCTAGTGTAGCCGTAGTTAGGCCACCACTTCAAGAACTCTGTAGCACCGCCTACATACCTCGCTCTGCTAATCCTGTTACCAGTGGCTGC
ori-016	CAGCAGAGCGCAGATACCAAATACTGTCCTTCTAGTGTAGCCGTAGTTAGGCCACCACTTCAAGAACTCTGTAGCACCGCCTACATACCTCGCTCTGCTAATCCTGTTACCAGTGGCTGC
ori-010	CAGCAGAGCGCAGATACCAAATACTGTTCTTCTAGTGTAGCCGTAGTTAGGCCACCACTTCAAGAACTCTGTAGCACCGCCTACATACCTCGCTCTGCTAATCCTGTTACCAGTGGCTGC
ori-011	CAGCAGAGCGCAGATACCAAATACTGTTCTTCTAGTGTAGCCGTAGTTAGGCCACCACTTCAAGAACTCTGTAGCACCGCCTACATACCTCGCTCTGCTAATCCTGTTACCAGTGGCTGC
ori-005	CAGCAGAGCGCAGATACCAAATACTGTCCTTCTAGTGTAGCCGTAGTTAGGCCACCACTTCAAGAACTCTGTAGCACCGCCTACATACCTCGCTCTGCTAATCCTGTTACCAGTGGCTGC
ori-018	CAGCAGAGCGCAGATACCAAATACTGTCCTTCTAGTGTAGCCGTAGTTAGGCCACCACTTCAAGAACTCTGTAGCACCGCCTACATACCTCGCTCTGCTAATCCTGTTACCAGTGGCTGC
ori-001	CAGCAGAGCGCAGATACCAAATACTGTTCTTCTAGTGTAGCCGTAGTTAGGCCACCACTTCAAGAACTCTGTAGCACCGCCTACATACCTCGCTCTGCTAATCCTGTTACCAGTGGCTGC
ori-004	CAGCAGAGCGCAGATACCAAATACTGTTCTTCTAGTGTAGCCGTAGTTAGGCCACCACTTCAAGAACTCTGTAGCACCGCCTACATACCTCGCTCTGCTAATCCTGTTACCAGTGGCTGC
ori-006	CAGCAGAGCGCAGATACCAAATACTGTTCTTCTAGTGTAGCCGTAGTTAGGCCACCACTTCAAGAACTCTGTAGCACCGCCTACATACCTCGCTCTGCTAATCCTGTTACCAGTGGCTGC
ori-007	CAGCAGAGCGCAGATACCAAATACTGTTCTTCTAGTGTAGCCGTAGTTAGGCCACCACTTCAAGAACTCTGTAGCACCGCCTACATACCTCGCTCTGCTAATCCTGTTACCAGTGGCTGC
ori-015	CAGCAGAGCGCAGATACCAAATACTGTTCTTCTAGTGTAGCCGTAGTTAGGCCACCACTTCAAGAACTCTGTAGCACCGCCTACATACCTCGCTCTGCTAATCCTGTTACCAGTGGCTGC
ori-017	CAGCAGAGCGCAGATACCAAATACTGTCCTTCTAGTGTAGCCGTAGTTAGGCCACCACTTCAAGAACTCTGTAGCACCGCCTACATACCTCGCTCTGCTAATCCTGTTACCAGTGGCTGC
ori-019	CAGCAGAGCGCAGATACCAAATACTGTCCTTCTAGTGTAGCCGTAGTTAGGCCACCACTTCAAGAACTCTGTAGCACCGCCTACATACCTCGCTCTGCTAATCCTGTTACCA------GC
ori-020	CAGCAGAGCGCAGATACCAAATACTGTTCTTCTAGTGTAGCCGTAGTTAGGCCACCACTTCAAGAACTCTGTAGCACCGCCTACATACCTCGCTCTGCTAATCCTGTTACCAGTGGCTGC
pBR322ori-t	---CTGC

-10 RNAI prom -35

RNAII

RNAI

Sequence Alignments

```
                                           γ-stem
ori-014         TGCCAGTGGCGATAAGTCGTGTCTTACCGGGTTGGACTCAAGACGATAGTTACCGGATAAGGCGCAGCGGTCGGGCTGAACGGGGGGTTCGTGCACACAGCCCAGCTTGGAGCGAACGAC
ori-013         TGCCAGTGGCGATAAGTCGTGTCTTACCGGGTTGGACTCAAGACGATAGTTACCGGATAAGGCGCAGCGGTCGGGCTGAACGGGGGGTTCGTGCACACAGCCCAGCTTGGAGCGAACGAC
ori-009         TGCCAGTGGCGATAAGTCGTGTCTTACCGGGTTGGACTCAAGACGATAGTTACCGGATAAGGCGCAGCGGTCGGGCTGAACGGGGGGTTCGTGCACACAGCCCAGCTTGGAGCGAACGAC
ori-012         TGCCAGTGGCGATAAGTCGTGTCTTACCGGGTTGGACTCAAGACGATAGTTACCGGATAAGGCGCAGCGGTCGGGCTGAACGGGGGGTTCGTGCACACAGCCCAGCTTGGAGCGAACGAC
ori-008         TGCCAGTGGCGATAAGTCGTGTCTTACCGGGTTGGACTCAAGACGATAGTTACCGGATAAGGCGCAGCGGTCGGGCTGAACGGGGGGTTCGTGCACACAGCCCAGCTTGGAGCGAACGAC
ori-003         TGCCAGTGGCGATAAGTCGTGTCTTACCGGGTTGGACTCAAGACGATAGTTACCGGATAAGGCGCAGCGGTCGGGCTGAACGGGGGGTTCGTGCACACAGCCCAGCTTGGAGCGAACGAC
ori-021         TGCCAGTGGCGATAAGTCGTGTCTTACCGGGTTGGACTCAAGACGATAGTTACCGGATAAGGCGCAGCGGTCGGGCTGAACGGGGGGTTCGTGCACACAGCCCAGCTTGGAGCGAACGAC
ori-002         TGCCAGTGGCGATAAGTCGTGTCTTACCGGGTTGGACTCAAGACGATAGTTACCGGATAAGGCGCAGCGGTCGGGCTGAACGGGGGGTTCGTGCACACAGCCCAGCTTGGAGCGAACGAC
ori-016         TGCCAGTGGCGATAAGTCGTGTCTTACCGGGTTGGACTCAAGACGATAGTTACCGGATAAGGCGCAGCGGTCGGGCTGAACGGGGGGTTCGTGCACACAGCCCAGCTTGGAGCGAACGAC
ori-010         TGCCAGTGGCGATAAGTCGTGTCTTACCGGGTTGGACTCAAGACGATAGTTACCGGATAAGGCGCAGCGGTCGGGCTGAACGGGGGGTTCGTGCACACAGCCCAGCTTGGAGCGAACGAC
ori-011         TGCCAGTGGCGATAAGTCGTGTCTTACCGGGTTGGACTCAAGACGATAGTTACCGGATAAGGCGCAGCGGTCGGGCTGAACGGGGGGTTCGTGCACACAGCCCAGCTTGGAGCGAACGAC
ori-005         TGCCAGTGGCGATAAGTCGTGTCTTACCGGGTTGGACTCAAGACGATAGTTACCGGATAAGGCGCAGCGGTCGGGCTGAACGGGGGGTTCGTGCACACAGCCCAGCTTGGAGCGAACGAC
ori-018         TGCCAGTGGCGATAAGTCGTGTCTTACCGGGTTGGACTCAAGACGATAGTTACCGGATAAGGCGCAGCGGTCGGGCTGAACGGGGGGTTCGTGCACACAGCCCAGCTTGGAGCGAACGAC
ori-001         TGCCAGTGGCGATAAGTCGTGTCTTACCGGGTTGGACTCAAGACGATAGTTACCGGATAAGGCGCAGCGGTCGGGCTGAACGGGGGGTTCGTGCACACAGCCCAGCTTGGAGCGAACGAC
ori-004         TGCCAGTGGCGATAAGTCGTGTCTTACCGGGTTGGACTCAAGACGATAGTTACCGGATAAGGCGCAGCGGTCGGGCTGAACGGGGGGTTCGTGCACACAGCCCAGCTTGGAGCGAACGAC
ori-006         TGCCAGTGGCGATAAGTCGTGTCTTACCGGGTTGGACTCAAGACGATAGTTACCGGATAAGGCGCAGCGGTCGGGCTGAACGGGGGGTTCGTGCACACAGCCCAGCTTGGAGCGAACGAC
ori-007         TGCCAGTGGCGATAAGTCGTGTCTTACCGGGTTGGACTCAAGACGATAGTTACCGGATAAGGCGCAGCGGTCGGGCTGAACGGGGGGTTCGTGCACACAGCCCAGCTTGGAGCGAACGAC
ori-015         TGCCAGTGGCGATAAGTCGTGTCTTACCGGGTTGGACTCAAGACGATAGTTACCGGATAAGGCGCAGCGGTCGGGCTGAACGGGGGGTTCGTGCACACAGCCCAGCTTGGAGCGAACGAC
ori-017         TGCCAGTGGCGATAAGTCGTGTCTTACCGGGTTGGACTCAAGACGATAGTTACCGGATAAGGCGCAGCGGTCGGGCTGAACGGGGGGTTCGTGCACACAGCCCAGCTTGGAGCGAACGAC
ori-019         TGCCAGTGGCGATAAGTCGTGTCTTACCGGGTTGGACTCAAGACGATAGTTACCGGATAAGGCGCAGCGGTCGGGCTGAACGGGGGGTTCGTGCACACAGCCCAGCTTGGAGCGAACGAC
ori-020         TGCCAGTGGCGATAAGTCGTGTCTTACCGGGTTGGACTCAAGACGATAGTTACCGGATAAGGCGCAGCGGTCGGGCTGAACGGGGGGTTCGTGCACACAGCCCAGCTTGGAGCGAACGAC
pBR322ori-t     TGCCAGTGGCGATAAGTCGTGTCTTACCGGGTTGGACTCAAGACGATAGTTACCGGATAAGGCGCAGCGGTCGGGCTGAACGGGGGGTTCGTGCACACAGCCCAGCTTGGAGCGAACGAC
                ************************************************************************************************************************
                                                                      RNAII
```

```
                                                          Hairpin 1
ori-014         CTACACCGAACTGAGATACCTACAGCGTGAGCTATGAGAAAGCGCCACGCTTCCCGAAGGGAGAAAGGCGGACAGGTATCCGGTAAGCGGCAGGGTCGGAACAGGAGAGCGCACGAGGGA
ori-013         CTACACCGAACTGAGATACCTACAGCGTGAGCTATGAGAAAGCGCCACGCTTCCCGAAGGGAGAAAGGCGGACAGGTATCCGGTAAGCGGCAGGGTCGGAACAGGAGAGCGCACGAGGGA
ori-009         CTACACCGAACTGAGATACCTACAGCGTGAGCATTGAGAAAGCGCCACGCTTCCCGAAGGGAGAAAGGCGGACAGGTATCCGGTAAGCGGCAGGGTCGGAACAGGAGAGCGCACGAGGGA
ori-012         CTACACCGAACTGAGATACCTACAGCGTGAGCTATGAGAAAGCGCCACGCTTCCCGAAGGGAGAAAGGCGGACAGGTATCCGGTAAGCGGCAGGGTCGGAACAGGAGAGCGCACGAGGGA
ori-008         CTACACCGAACTGAGATACCTACAGCGTGAGCATTGAGAAAGCGCCACGCTTCCCGAAGGGAGAAAGGCGGACAGGTATCCGGTAAGCGGCAGGGTCGGAACAGGAGAGCGCACGAGGGA
ori-003         CTACACCGAACTGAGATACCTACAGCGTGAGCTATGAGAAAGCGCCACGCTTCCCGAAGGGAGAAAGGCGGACAGGTATCCGGTAAGCGGCAGGGTCGGAACAGGAGAGCGCACGAGGGA
ori-021         CTACACCGAACTGAGATACCTACAGCGTGAGCTATGAGAAAGCGCCACGCTTCCCGAAGGGAGAAAGGCGGACAGGTATCCGGTAAGCGGCAGGGTCGGAACAGGAGAGCGCACGAGGGA
ori-002         CTACACCGAACTGAGATACCTACAGCGTGAGCTATGAGAAAGCGCCACGCTTCCCGAAGGGAGAAAGGCGGACAGGTATCCGGTAAGCGGCAGGGTCGGAACAGGAGAGCGCACGAGGGA
ori-016         CTACACCGAACTGAGATACCTACAGCGTGAGCATTGAGAAAGCGCCACGCTTCCCGAAGGGAGAAAGGCGGACAGGTATCCGGTAAGCGGCAGGGTCGGAACAGGAGAGCGCACGAGGGA
ori-010         CTACACCGAACTGAGATACCTACAGCGTGAGCTATGAGAAAGCGCCACGCTTCCCGAAGGGAGAAAGGCGGACAGGTATCCGGTAAGCGGCAGGGTCGGAACAGGAGAGCGCACGAGGGA
ori-011         CTACACCGAACTGAGATACCTACAGCGTGAGCTATGAGAAAGCGCCACGCTTCCCGAAGGGAGAAAGGCGGACAGGTATCCGGTAAGCGGCAGGGTCGGAACAGGAGAGCGCACGAGGGA
ori-005         CTACACCGAACTGAGATACCTACAGCGTGAGCTATGAGAAAGCGCCACGCTTCCCGAAGGGAGAAAGGCGGACAGGTATCCGGTAAGCGGCAGGGTCGGAACAGGAGAGCGCACGAGGGA
ori-018         CTACACCGAACTGAGATACCTACAGCGTGAGCATTGAGAAAGCGCCACGCTTCCCGAAGGGAGAAAGGCGGACAGGTATCCGGTAAGCGGCAGGGTCGGAACAGGAGAGCGCACGAGGGA
ori-001         CTACACGGAACTGAGATACCTACAGCGTGAGCTATGAGAAAGCGCCACGCTTCCCGAAGGGAGAAAGGCGGACAGGTATCCGGTAAGCGGCAGGGTCGGAACAGGAGAGCGCACGAGGGA
ori-004         CTACACCGAACTGAGATACCTACAGCGTGAGCATTGAGAAAGCGCCACGCTTCCCGAAGGGAGAAAGGCGGACAGGTATCCGGTAAGCGGCAGGGTCGGAACAGGAGAGCGCACGAGGGA
ori-006         CTACACCGAACTGAGATACCTACAGCGTGAGCTATGAGAAAGCGCCACGCTTCCCGAAGGGAGAAAGGCGGACAGGTATCCGGTAAGCGGCAGGGTCGGAACAGGAGAGCGCACGAGGGA
ori-007         CTACACCGAACTGAGATACCTACAGCGTGAGCTATGAGAAAGCGCCACGCTTCCCGAAGGGAGAAAGGCGGACAGGTATCCGGTAAGCGGCAGGGTCGGAACAGGAGAGCGCACGAGGGA
ori-015         CTACACCGAACTGAGATACCTACAGCGTGAGCTATGAGAAAGCGCCACGCTTCCCGAAGGGAGAAAGGCGGACAGGTATCCGGTAAGCGGCAGGGTCGGAACAGGAGAGCGCACGAGGGA
ori-017         CTACACCGAACTGAGATACCTACAGCGTGAGCATTGAGAAAGCGCCACGCTTCCCGAAGGGAGAAAGGCGGACAGGTATCCGGTAAGCGGCAGGGTCGGAACAGGAGAGCGCACGAGGGA
ori-019         CTACACCGAACTGAGATACCTACAGCGTGAGCTATGAGAAAGCGCCACGCTTCCCGAAGGGAGAAAGGCGGACAGGTATCCGGTAAGCGGCAGGGTCGGAACAGGAGAGCGCACGAGGGA
ori-020         CTACACCGAACTGAGATACCTACAGCGTGAGCTATGAGAAAGCGCCACGCTTCCCGAAGGGAGAAAGGCGGACAGGTATCCGGAAAGCGGCAGGGTCGGAACAGGAGAGCGCACGAGGGA
pBR322ori-t     CTACACCGAACTGAGATACCTACAGCGTGAGCTATGAGAAAGCGCCACGCTTCCCGAAGGGAGAAAGGCGGACAGGTATCCGGTAAGCGGCAGGGTCGGAACAGGAGAGCGCACGAGGGA
                ****** *************************  ************************************************* ************************************
                                                                      RNAII
```

Sequence Alignments

```
                          Hairpin 2                                                                       Displaced Strand
ori-014       GCTTCCAGGGGGAAACGCCTGGTATCTTTATAGTCCTGTCGGGTTTCGCCACCTCTGACTTGAGCGTCGATTTTTGTGATGCTCGTCAGGGGGGCGGAGCCTATGGAAA
ori-013       GCTTCCAGGGGGAAACGCCTGGTATCTTTATAGTCCTGTCGGGTTTCGCCACCTCTGACTTGAGCGTCGATTTTTGTGATGCTCGTCAGGGGGGCGGAGCCTATGGAAA
ori-009       GCTTCCAGGGGGAAACGCCTGGTATCTTTATAGTCCTGTCGGGTTTCGCCACCTCTGACTTGAGCGTCGATTTTTGTGATGCTCGTCAGGGGGGCGGAGCCTATGGAAA
ori-012       GCTTCCAGGGGGAAACGCCTGGTATCTTTATAGTCCTGTCGGGTTTCGCCACCTCTGACTTGAGCGTCGATTTTTGTGATGCTCGTCAGGGGG-CGGAGCCTATGGAAA
ori-008       GCTTCCAGGGGGAAACGCCTGGTATCTTTATAGTCCTGTCGGGTTTCGCCACCTCTGACTTGAGCGTCGATTTTTGTGATGCTCGTCAGGGGGGCGGAGCCTATGGAAA
ori-003       GCTTCCAGGGGGAAACGCCTGGTATCTTTATAGTCCTGTCGGGTTTCGCCACCTCTGACTTGAGCGTCGATTTTTGTGATGCTCGTCAGGGGGGCGGAGCCTATGGAAA
ori-021       GCTTCCAGGGGGAAACGCCTGGTATCTTTATAGTCCTGTCGGGTTTCGCCACCTCTGACTTGAGCATCGATTTTTGTGATGCTCGTCAGGGGGGCGGAGCCTATGGAAA
ori-002       GCTTCCAGGGGGAAACGCCTGGTATCTTTATAGTCCTGTCGGGTTTCGCCACCTCTGACTTGAGCGTCGATTTTTGTGATGCTCGTCAGGGGGGCCGAGCCTATGGAAA
ori-016       GCTTCCAGGGGGGAACGCCTGGTATCTTTATAGTCCTGTCGGGTTTCGCCACCTCTGACTTGAGCGTCGATTTTTGTGATGCTCGTCAGGGGGGCGGAGCCTATGGAAA
ori-010       GCTTCCAGGGGGAAACGCCTGGTATCTTTATAGTCCTGTCGGGTTTCGCCACCTCTGACTTGAGCGTCGATTTTTGTGATGCTCGTCAGGGGGGCGGAGCCTATGGAAA
ori-011       GCTTCCAGGGGGAAACGCCTGGTATCTTTATAGTCCTGTCGGGTTTCGCCACCTCTGACTTGAGCGTCGATTTTTGTGATGCTCGTCAGGGGG-CGGAGCCTATGGAAA
ori-005       GCTTCCAGGGGGAAACGCCTGGTATCTTTATAGTCCTGTCGGGTTTCGCCACCTCTGACTTGAGCGTCGATTTTTGTGATGCTCGTCAGGGGGGCGGAGCCTATGGAAA
ori-018       GCTTCCAGGGGGAAACGCCTGGTATCTTTATAGTCCTGTCGGGTTTCGCCACCTCTGACTTGAGCGTCGATTTTTGTGATGCTCGTCAGGGGGGCGGAGCCTATGGAAA
ori-001       GCTTCCAGGGGGAAACGCCTGGTATCTTTATAGTCCTGTCGGGTTTCGCCACCTCTGACTTGAGCGTCGATTTTTGTGATGCTCGTCAGGGGGGCGGAGCCTATGGAAA
ori-004       GCTTCCAGGGGGAAACGCCTGGTATCTTTATAGTCCTGTCGGGTTTCGCCACCTCTGACTTGAGCGTCGATTTTTGTGATGCTCGTCAGGGGGGCGGAGCCTATGGAAA
ori-006       GCTTCCAGGGGGAAACGCCTGGTATCTTTATAGTCCTGTCGGGTTTCGCCACCTCTGACTTGAGCGTCGATTTTTGTGATGCTCGTCAGGGGGGCGGAGCCTGTGGAAA
ori-007       GCTTCCAGGGGGAAACGCCTGGTATCTTTATAGTCCTGTCGGGTTTCGCCACCTCTGACTTGAGCGTCGATTTTTGTGATGCTCGTCAGGGGGGCGGAGCCTATGGAAA
ori-015       GCTTCCAGGGGGAAACGCCTGGTATCTTTATAGTCCTGTCGGGTTTCGCCACCTCTGACTTGAGCGTCGATTTTTGTGATGCTCGTCAGGGGGGCGGAGCCTATGGAAA
ori-017       GCTTCCAGGGGGAAACGCCTGGTATCTTTATAGTCCTGTCGGGTTTCGCCACCTCTGACTTGAGCGTCGATTTTTGTGATGCTCGTCAGGGGGGCGGAGCCTATGGAAA
ori-019       GCTTCCAGGGGGAAACGCCTGGTATCTTTATAGTCCTGTCGGGTTTCGCCACCTCTGACTTGAGCGTCGATTTTTGTGATGCTCGTCAGGGGGGCGGAGCCTATGGAAA
ori-020       GCTTCCAGGGGGAAACGCCTGGTATCTTTATAGTCCTGTCGGGTTTCGCCACCTCTGACTTGAGCGTCGATTTTTGTGATGCTCGTCAGGGGGGCGGAGCCTATGGAAA
pBR322ori-t   GCTTCCAGGGGGAAACGCCTGGTATCTTTATAGTCCTGTCGGGTTTCGCCACCTCTGACTTGAGCGTCGATTTTTGTGATGCTCGTCAGGGGGGCGGAGCCTATGGAAA
              ************ **************************************************** *************************** * ****** ******
                                                   RNAII
```

Structural elements from Selzer et al. (1983) *Cell* **32**:119-129. These ori sequences are ColE1/pMB1 derived origins of replication. RNAII (black arrow) is a transcript that hybridizes with single-stranded DNA in the displaced strand (cyan) region to form a "D-loop" and is processed by RNAse H to form an RNA primer for DNA pol. RNAII is transcribed from a promoter at the extreme 5′ end of the sequence.

RNAI is an antisense RNA transcribed from RNAI promoter. RNAI and RNAII form small stem loop structures (SL1-3; red) that can interact and facilitate full sense-antisense hybridization. RNAI-RNAII covers the α-stem region (yellow; SL1 overlaps), leaving the free region of RNAII to form a large β-γ stem loop, which inhibits D-loop formation and replication. Rop protein, expressed from a separate locus not part of ori, stabilizes RNAI-RNAII, preventing replication.

Free RNAII forms an α-β stem loop and small hairpin structures (green) that facilitate D-loop formation and replication.

Plasmids with wild-type ColE1/pMB1 origins and Rop exist at ~15-20 copies/cell. An example of this type of medium copy number plasmid is pBR322.
High copy number (up to 700/cell) ColE1/pMB1 plasmids, such as the pUC and pET series, lack Rop and also carry a pMB1 derivative in which the A at -1 from the start of the RNAI transcript is changed to G (T-C in the above orientation). This mutation moves the transcription start site +3, which removes the "anti-tail" portion of RNAI, which is crucial to nucleate RNAI-RNAII hybrid formation.

The apparent mutations in ori-007, -012 and -015 in SL3 could destabilize stem-loop formation and reduce the ability of RNAI to anneal to RNAII, resulting in higher copy numbers than pBR322.

The truncated pBR322 ori is non-functional. The plasmid carrying this (pBINPLUS) uses oriV instead for its replication. pMB1/ColE1 origins can be truncated up to the β-stem, but a strong promoter must be placed in front to make the origin functional.

ori-017 lacks the RNAII promoter. Unless there is a cryptic promoter in SV40 ori immediately 5′ to the sequence shown, this ori should be non-functional. BPROM identifies a potential -35 and -10 promoter about 96 bp 5′ to ori within SV40 ori.

Key ATGC = change in nucleotide relative to consensus sequence – non-coding sequence

Sequence Alignments

ColE1-Related Promoters using a similar RNA based replication initiation mechanism

```
                                                   RNAII prom                             SL3                                SL2                 α-stem  SL1
pBR322_ori      ----------------------------TTGAGATCCTTTTTTTCTGCGCGTAATCTGCTGCTTGCAAACAAAA-AAACCACCGCTACCAGCGGTGGTTT-GTTTGCCGGATCAAGAGCTACCAACTCTTT-TTCCGAAGGTAACTGGCTTCAGCAGAGCGCAGATACCAAATACTGTCC
RSF_ori         ------AACGGAATAGCTGTTCGTTGACTTGATAGACCGATTGATTCATCATCTCAT-------AAATAAAGA-AAAACCACCGCTACCAACGGTGGTTTT-CTCAAGGTTCGCTGAGCTACCAACTCTTT-GAACCAAGGTAAGTGGGTTGGAGGACCGCACT-CACCAAAATCTGTTC
p15A_ori-001    ----------------------------TTGAGATCGTTTTGGTCTGCGCGTAATCTCTTGCTCTGAAAACGA---AAAAACCGCCTTGCAGGGAGGTTTTT-CGAAGGTTCTCTGAGCTACCAACTCTTT-GAACCGAGGTAACTGGCTTGCAGGAGCGCAGT-CACCAAAACTTGTCC
ColA_ori        TGGTGTCGGGAATCCGTAAAGGATCTTCTTGAGATCCTTTTACGATCGTCGTAATCTCCTGCTCTGTAAACGAAA---AAACCGCCTGGGGAGGCGGTTT-GATCGAAGGTTAAGTCAGTTGGGGAACTGCTTAACCT-GGTAACTGGCTTTAGTGGAGCGCAGATACCAAATACTGTCC
CloDF13_ori     -------------GATCAAAGGATCTTCTTGAGATCCTTTTTTTCTGCGCGTAATCTTTTGCCCTGTAAACGAAA---AAACCACCTGGGGAGGTGGTTT-GATCGAAGGTTAAGTCAGTTGGGGAACTGCTTAACCGTGGTAACTGGCTTTCGCAGAGCACAGCAACCAAAT-CTGTCC
                                            **** *      *    *     *    *       *       ***   *****  *      ** *****       ** *        *       *       *** ***** **  **   * **        ******   *** *

                                 RNAI prom                                β-stem                                  γ-stem
pBR322_ori      TTCTAGTGTAGCCGTAGTTAGGCCACCACTTCAAGA--ACTCTGTAGCACCGCCTACATACCTCGCTCTGCTAATCCTGTTACCAGTGGCTGCTGCCAGTGGCGATAAGTCGTGTCTTACCGGGTTGGACTCAAGACGATAGTTACCGGATAAGGCGCAGCGGTCGGGCTGAACGGGGGG
RSF_ori         TTTCAGTTTAGCCTTAACAGGTGCATAACTTCAAGA-------------------------CAAAGTCCTCTAAATCAGTTACCAATGGCTGCTGCCAGTGGCGATAAGTCGTGTCTTACCGGGTTGGACTCAAGACGATAGTTACCGGATAAGGCGCAGCGGTCGGGCTGAACGGGGGG
p15A_ori-001    TTTCAGTTTAGCCTTAACCGGCGCATGACTTCAAGA-------------------------CTAACTCCTCTAAATCAATTACCAGTGGCTGCTGCCAGTGGTGCTTTTGCATGTCTTTCCGGGTTGGACTCAAGACGATAGTTACCGGATAAGGCGCAGCGGTCGGACTGAACGGGGGG
ColA_ori        TTTCAGTGTAGCCTCTGTTAGGCCACCACTTCAAGA-------------CTCTCGATATCTAAATCCACTAATTCTCAGTTACCAATGGCTGCTGCCAGTGGCGTTTTGTCGTGTCTTTCCGGGTTGGACTCAAGATGATAGTTACCGGATAAGGCGCAGCAGTCGGGCTGAACGGGGGG
CloDF13_ori     TTCCAGTGTAGCCGGACTTTGGCGCACACTTCAAGAGCAACCGCGTGTTTAGCTAAACAAATCCTCTGCGAACTCCCAGTTACCAATGGCTGCTGCCAGTGGCGTTTTACCGTGCTTTTCCGGGTTGGACTCAAGTGAACAGTTACCGGATAAGGCGCAGCAGTCGGGCTGAACGGGGAG
                **  *** *****       *      *********                            **  * **   *   ****** *************** *  *    *  **  **  ***************    *  ********************* ***** ********** *

                                                                                         Hairpin 1                                                             Hairpin 2
pBR322_ori      TTCGTGCACACAGCCCAGCTTGGAGCGAACGACCTACACCGAACTGAGATACCTACAGCGTGAGCTATGAGAAAGCGCCACGCTTCCCGAAGGGAGAAAGGCGGA--CAGGTATCCGGTAAGCGGCAGGGTCGGAACAGGAGAGCGCACGAGGGAGCTTCCAGGGGGAAACGCCTGGTAT
RSF_ori         TTCGTGCACACAGCCCAGCTTGGAGCGAACGACCTACACCGAACTGAGATACCAACAGCGTGAGCTATGAGAAAGCGCCACGCTTCCCGAAGGGAGAAAGGCGGA--CAGGTATCCGGTAAGCGGCAGGGTCGGAACAGGAGAGCGCACGAGGGAGCTTCCAGGGGGAAACGCCTGGTAT
p15A_ori-001    TTCGTGCATACAGTCCAGCTTGGAGCGAACTGCCTACCCGGAACTGAGTGTCAGGCGTGGAATGAGACAAACGCGGCCATAACAGCGGA------------------ATGACACCGGTAAACCGAAAGGCAGGAACAGGAGAGCGCACGAGGGAGCCGCCAGGGGGAAACGCCTGGTAT
ColA_ori        TTCTTGCACACAGCCCAGCTTGGAGCGAACTGTCTACACGGAACGGACGTGGTGATTTGGGTAAAGCCTCCACCACAACACGGACGCCGCAGGA-----------------------------------CGGGAACAGGAGAGCGCAAGAGGGAGCCCATCAGGGGGAAACGCCTGGTAT
CloDF13_ori     TTCTTGCTTACAGCCCAGCTTGGAGCGAACGACCTACACCGAGCCGAGATACC-AGTGTGTGAGCTATGAGAAAGCGCCACACTTCCCGTAAGGGAGAAAGGCGGAACAGGTATCCGGTAAACGGCAGGGTCGGAACAGGAGAGCGCAAGAGGGAGCGACCCGCCGGAAACGGTGGGGAT
                *** ***  **** ****************   **** * ** * *             ***      *  *       **    *         *       ***                          *************** ********    * *  *******   ** **

                                                                                                               Displaced Strand
pBR322_ori      CTTTATAGTCCTGTCGGGTTTCGCCACCTCTGACTTG-----------------------------------------AGCGTCGATTTTTGTGATGCTCGTCAGGGGGCGGAGCCTATGGAAA--------------------
RSF_ori         CTTTATAGTCCTGTCGGGTTTCGCCACCTCTGGCTTG-----------------------------------------AGCGTCGATTTTTGTGATGCTCGTCAGGGGGCGGAGCCTATGGAAAAACGCCTGCGGCGTTGGCTT
p15A_ori-001    CTTTATAGTCCTGTCGGGTTTCGCCACCACTGATTTG-----------------------------------------AGCGTCAGATTTCGTGATGCTTGTCAGGGG-CGGAGCCTATGGAAA--------------------
ColA_ori        CTTTATAGTCCTGTCGGGTTTCGCCACCACTGATTTG-----------------------------------------AGCGTCAGATTTCGTGATGTTCGTCAGGGGGCGGAGCCTATGGAAAAACGGCTTCGCTCCGGCCTT
CloDF13_ori     CTTTA-AGTCCTGTCGGGTTTCGCCCGTACTGTCAGATTCATGGTTGAGCCTCACGGCTCCCACAGATGCACCGGAAAAGCGTCTGTTTATGTGAACTCTGGCAGGAGGGCGGAGCCTATGGAAAAACGCCACCGGCGCGGCCCT
                ***** *******************    ***                                              ******   **  ****     * **** ** **************
```

Colour scheme for structural elements same as for pMB1/ColE1 ori alignment. From Selzer et al. (1983) *Cell* **32**:119-129.

Plasmids using p15A, ColA, RSF1030 or CloDF13 origins can be co-transformed into cells with plasmids carrying pMB1/ColE1 origins because the RNA sequences are sufficiently different to prevent competition between replication origins. Two plasmid systems are useful for bacterial co-expression of proteins, especially for purification of protein complexes that are difficult to express as individual proteins.

Three plasmid co-expression can be accomplished with a third plasmid that uses an iteron-based replicon. Iterons are repeat sequences to which a DNA-binding protein binds, bending the origin and facilitating its melting and entry of the replication machinery. These plasmids must carry the gene for the DNA-binding protein to express some of these proteins. Alternatively, some *E. coli* strains are engineered to express an iteron binding protein, and some can do so conditionally to control plasmid copy number.

Examples of iteron origins are ori2/oriS from F plasmid (uses repE protein), oriV from RK2/IncP-α plasmid (uses trfA protein), oriV from pSC101 plasmid (uses rep101/repA protein), oriγ from R6K plasmid (uses pi protein), and oriV from pSa plasmid (uses repA protein).

Some plasmids, such as bacterial artificial chromosomes (BACs) based on the F plasmid ori2/repE replicon, are very low copy number (1-2/cell) to reduce toxic effects of cloned genes or recombination in the insert. These plasmids require partitioning mechanisms (SopA/B/C) to ensure the plasmid is divided between daughter cells. Some plasmids carry two compatible promoters and use conditional expression of iteron proteins to increase copy number when plasmid isolation is required.

The two annotated ori2 variants in the features list are identical but reverse complements
oriV-003 and 004 are identical but reverse complements
The two annotated pSC101 ori variants are identical but reverse complements

Key ATGC = change in nucleotide relative to consensus sequence – non-coding sequence

Sequence Alignments

Name of Variant	# of Occur	Alignment	Size (bp)	# Var bp	Sources*
IRES-014	4	--GAGGGCCC	464	1/1i	No
IRES-001	4	--GTTATTTTCCACCATATTGCCGTCTTTTGGCAATGTGAGGGCCC	463	2	No
IRES-013	2	--GTTATTTTCCACCATATTGCCGTCTTTTGGCAATGTGAGGGCCC	499	1/1d	15232106
IRES-012	1	--GTTATTTTCCACCATATTGCCGTCTTTTGGCAATGTGAGGGCCC	485	1/1d	No
IRES-004	2	-------------------------CGTTACTGGCCGAAGCCGCTTGGAATAAGGCCGGTGTGCGTTTGTCTATATGTTATTTTCCACCATATTGCCGTCTTTTGGCAATGTGAGGGCCC	551	2/1d	Ox
IRES-008	1+1	------------------------ACGTTACTGGCCGAAGCCGCTTGGAATAAGGCCGGTGTGCGTTTGTCTATATGTTATTTTCCACCATATTGCCGTCTTTTGGCAATGTGAGGGCCC	553	0	Or
IRES-015	3	------------------------ACGTTACTGGCCGAAGCCGCTTGGAATAAGGCCGGTGTGCGTTTGTCTATATGTTATTTTCCACCATATTGCCGTCTTTTGGCAATGTGAGGGCCC	552	1	Or
IRES-010	2	CCCCTCTCCCTCCCCCCCCCC-TAACGTTACTGGCCGAAGCCGCTTGGAATAAGGCCGGTGTGCGTTTGTCTATATGTTATTTTCCACCATATTGCCGTCTTTTGGCAATGTGAGGGCCC	569	0	OB
IRES-002	4	CCCCTCTCCCTCCCCCCCCCC-TAACGTTACTGGCCGAAGCCGCTTGGAATAAGGCCGGTGTGCGTTTGTCTATATGTTATTTTCCACCATATTGCCGTCTTTTGGCAATGTGAGGGCCC	573	1/1d	Cl
IRES-005	3	CCCCTCTCCCTCCCCCCCCCC-TAACGTTACTGGCCGAAGCCGCTTGGAATAAGGCCGGTGTGCGTTTGTCTATATGTTATTTTCCACCATATTGCCGTCTTTTGGCAATGTGAGGGCCC	573	1d	Cl
IRES-003	35	CCCCTCTCCCTCCCCCCCCCC-TAACGTTACTGGCCGAAGCCGCTTGGAATAAGGCCGGTGTGCGTTTGTCTATATGTTATTTTCCACCATATTGCCGTCTTTTGGCAATGTGAGGGCCC	574	0	Cl,Or
IRES-006	1	CCCCTCTCCCTCCCCCCCCCC-TAACGTTACTGGCCGAAGCCGCTTGGAATAAGGCCGGTGTGCGTTTGTCTATATGTTATTTTCCACCATATTGCCGTCTTTTGGCAATGTGAGGGCCC	574	1	Cl
IRES-011	2	CCCCTCTCCCTCCCCCCCCCCCTAACGTTACTGGCCGAAGCCGCTTGGAATAAGGCCGGTGTGCGTTTGTCTATATGTTATTTTCCACCATATTGCCGTCTTTTGGCAATGTGAGGGCCC	575	1/1i	Cl
IRES2-001	3	CCCCTCTCCCTCCCCCCCCCCCTAACGTTACTGGCCGAAGCCGCTTGGAATAAGGCCGGTGTGCGTTTGTCTATATGTTATTTTCCACCATATTGCCGTCTTTTGGCAATGTGAGGGCCC	588	1/1i	Cl
IRES2-002	11	CCCCTCTCCCTCCCCCCCCCC-TAACGTTACTGGCCGAAGCCGCTTGGAATAAGGCCGGTGTGCGTTTGTCTATATGTTATTTTCCACCATATTGCCGTCTTTTGGCAATGTGAGGGCCC	587	1	Cl
IRES2-003	4	CCCCTCTCCCTCCCCCCCCCC-TAACGTTACTGGCCGAAGCCGCTTGGAATAAGGCCGGTGTGCGTTTGTCTATATGTTATTTTCCACCATATTGCCGTCTTTTGGCAATGTGAGGGCCC	587	0	Cl,Or
		**Stem-Loop D** **Stem-Loop E** **Stem-Loop F**			

IRES-014	GGAAACCTGGCCCTGTCTTCTTGACGAGCATTCCTAGGGGTCTTTCCCCTCTCGCCAAAGGAATGCAAGGTCTGTTGAATGTCGTGAAGGAAGCAGTTCCTCTGGAAGCTTCTTGAAGAC
IRES-001	GGAAACCTGGCCCTGTCTTCTTGACGAGCATTCCTAGGGGTCTTTCCCCTCTCGCCAAAGGAATGCAAGGTCTGTTGAATGTCGTGAAGGAAGCAGTTCCTCTGGAAGCTTCTTGAAGAC
IRES-013	GGAAACCTGGCCCTGTCTTCTTGACGAGCATTCCTAGGGGTCTTTCCCCTCTCGCCAAAGGAATGCAAGGTCTGTTGAATGTCGTGAAGGAAGCAGTTCCTCTGGAAGCTTCTTGAAGAC
IRES-012	GGAAACCTGGCCCTGTCTTCTTGACGAGCATTCCTAGGGGTCTTTCCCCTCTCGCCAAAGGAATGCAAGGTCTGTTGAATGTCGTGAAGGAAGCAGTTCCTCTGGAAGCTTCTTGAAGAC
IRES-004	GGAAACCTGGCCCTGTCTTCTTGACGAGCATTCCTAGGGGTCTTTCCCCTCTCGCCAAAGGAATGCAAGGTCTGTTGAATGTCGTGAAGGAAGCAGTTCCTCTGGAAGCTTCTTGAAGAC
IRES-008	GGAAACCTGGCCCTGTCTTCTTGACGAGCATTCCTAGGGGTCTTTCCCCTCTCGCCAAAGGAATGCAAGGTCTGTTGAATGTCGTGAAGGAAGCAGTTCCTCTGGAAGCTTCTTGAAGAC
IRES-015	GGAAACCTGGCCCTGTCTTCTTGACGAGCATTCCTAGGGGTCTTTCCCCTCTCGCCAAAGGAATGCAAGGTCTGTTGAATGTCGTGAAGGAAGCAGTTCCTCTGGAAGCTTCTTGAAGAC
IRES-010	GGAAACCTGGCCCTGTCTTCTTGACGAGCATTCCTAGGGGTCTTTCCCCTCTCGCCAAAGGAATGCAAGGTCTGTTGAATGTCGTGAAGGAAGCAGTTCCTCTGGAAGCTTCTTGAAGAC
IRES-002	GGAAACCTGGCCCTGTCTTCTTGACGAGCATTCCTAGGGGTCTTTCCCCTCTCGCCAAAGGAATGCAAGGTCTGTTGAATGTCGTGAAGGAAGCAGTTCCTCTGGAAGCTTCTTGAAGAC
IRES-005	GGAAACCTGGCCCTGTCTTCTTGACGAGCATTCCTAGGGGTCTTTCCCCTCTCGCCAAAGGAATGCAAGGTCTGTTGAATGTCGTGAAGGAAGCAGTTCCTCTGGAAGCTTCTTGAAGAC
IRES-003	GGAAACCTGGCCCTGTCTTCTTGACGAGCATTCCTAGGGGTCTTTCCCCTCTCGCCAAAGGAATGCAAGGTCTGTTGAATGTCGTGAAGGAAGCAGTTCCTCTGGAAGCTTCTTGAAGAC
IRES-006	GGAAACCTGGCCCTGTCTTCTTGACGAGCATTCCTAGGGGTCTTTCCCCTCTCGCCAAAGGAATGCAAGGTCTGTTGAATGTCGTGAAGGAAGCAGTTCCTCTGGAAGCTTCTTGAAGAC
IRES-011	GGAAACCTGGCCCTGTCTTCTTGACGAGCATTCCTAGGGGTCTTTCCCCTCTCGCCAAAGGAATGCAAGGTCTGTTGAATGTCGTGAAGGAAGCAGTTCCTCTGGAAGCTTCTTGAAGAC
IRES2-001	GGAAACCTGGCCCTGTCTTCTTGACGAGCATTCCTAGGGGTCTTTCCCCTCTCGCCAAAGGAATGCAAGGTCTGTTGAATGTCGTGAAGGAAGCAGTTCCTCTGGAAGCTTCTTGAAGAC
IRES2-002	GGAAACCTGGCCCTGTCTTCTTGACGAGCATTCCTAGGGGTCTTTCCCCTCTCGCCAAAGGAATGCAAGGTCTGTTGAATGTCGTGAAGGAAGCAGTTCCTCTGGAAGCTTCTTGAAGAC
IRES2-003	GGAAACCTGGCCCTGTCTTCTTGACGAGCATTCCTAGGGGTCTTTCCCCTCTCGCCAAAGGAATGCAAGGTCTGTTGAATGTCGTGAAGGAAGCAGTTCCTCTGGAAGCTTCTTGAAGAC

IRES-014	AAACAACGTCTGTAGCGACCCTTTGCAGGCAGCGGAACCCCCCACCTGGCGACAGGTGCCTCTGCGGCCAAAAGCCACGTGTATAAGATACACCTGCAAAGGCGGCACAACCCCAGTGCC
IRES-001	AAACAACGTCTGTAGCGACCCTTTGCAGGCAGCGGAACCCCCCACCTGGCGACAGGTGCCTCTGCGGCCAAAAGCCACGTGTATAAGATACACCTGCAAAGGCGGCACAACCCCAGTGCC
IRES-013	AAACAACGTCTGTAGCGACCCTTTGCAGGCAGCGGAACCCCCCACCTGGCGACAGGTGCCTCTGCGGCCAAAAGCCACGTGTATAAGATACACCTGCAAAGGCGGCACAACCCCAGTGCC
IRES-012	AAACAACGTCTGTAGCGACCCTTTGCAGGCAGCGGAACCCCCCACCTGGCGACAGGTGCCTCTGCGGCCAAAAGCCACGTGTATAAGATACACCTGCAAAGGCGGCACAACCCCAGTGCC
IRES-004	AAACAACGTCTGTAGCGACCCTTTGCAGGCAGCGGAACCCCCCACCTGGCGACAGGTGCCTCTGCGGCCAAAAGCCACGTGTATAAGATACACCTGCAAAGGCGGCACAACCCCAGTGCC
IRES-008	AAACAACGTCTGTAGCGACCCTTTGCAGGCAGCGGAACCCCCCACCTGGCGACAGGTGCCTCTGCGGCCAAAAGCCACGTGTATAAGATACACCTGCAAAGGCGGCACAACCCCAGTGCC
IRES-015	AAACAACGTCTGTAGCGACCCTTTGCAGGCAGCGGAACCCCCCACCTGGCGACAGGTGCCTCTGCGGCCAAAAGCCACGTGTATAAGATACACCTGCAAAGGCGGCACAACCCCAGTGCC
IRES-010	AAACAACGTCTGTAGCGACCCTTTGCAGGCAGCGGAACCCCCCACCTGGCGACAGGTGCCTCTGCGGCCAAAAGCCACGTGTATAAGATACACCTGCAAAGGCGGCACAACCCCAGTGCC
IRES-002	AAACAACGTCTGTAGCGACCCTTTGCAGGCAGCGGAACCCCCCACCTGGCGACAGGTGCCTCTGCGGCCAAAAGCCACGTGTATAAGATACACCTGCAAAGGCGGCACAACCCCAGTGCC
IRES-005	AAACAACGTCTGTAGCGACCCTTTGCAGGCAGCGGAACCCCCCACCTGGCGACAGGTGCCTCTGCGGCCAAAAGCCACGTGTATAAGATACACCTGCAAAGGCGGCACAACCCCAGTGCC
IRES-003	AAACAACGTCTGTAGCGACCCTTTGCAGGCAGCGGAACCCCCCACCTGGCGACAGGTGCCTCTGCGGCCAAAAGCCACGTGTATAAGATACACCTGCAAAGGCGGCACAACCCCAGTGCC
IRES-006	AAACAACGTCTGTAGCGACCCTTTGCAGGCAGCGGAACCCCCCACCTGGCGACAGGTGCCTCTGCGGCCAAAAGCCACGTGTATAAGATACACCTGCAAAGGCGGCACAACCCCAGTGCC
IRES-011	AAACAACGTCTGTAGCGACCCTTTGCAGGCAGCGGAACCCCCCACCTGGCGACAGGTGCCTCTGCGGCCAAAAGCCACGTGTATAAGATACACCTGCAAAGGCGGCACAACCCCAGTGCC
IRES2-001	AAACAACGTCTGTAGCGACCCTTTGCAGGCAGCGGAACCCCCCACCTGGCGACAGGTGCCTCTGCGGCCAAAAGCCACGTGTATAAGATACACCTGCAAAGGCGGCACAACCCCAGTGCC
IRES2-002	AAACAACGTCTGTAGCGACCCTTTGCAGGCAGCGGAACCCCCCACCTGGCGACAGGTGCCTCTGCGGCCAAAAGCCACGTGTATAAGATACACCTGCAAAGGCGGCACAACCCCAGTGCC
IRES2-003	AAACAACGTCTGTAGCGACCCTTTGCAGGCAGCGGAACCCCCCACCTGGCGACAGGTGCCTCTGCGGCCAAAAGCCACGTGTATAAGATACACCTGCAAAGGCGGCACAACCCCAGTGCC

Sequence Alignments

```
IRES-014     ACGTTGTGAGTTGGATAGTTGTGGAAAGAGTCAAATGGCTCACCTCAAGCGTATTCAACAAGGGGCTGAAGGATGCCCAGAAGGTACCCCATTGTATGGGAATCTGATCTGGGGCCTCGG
IRES-001     ACGTTGTGAGTTGGATAGTTGTGGAAAGAGTCAAATGGCTCACCTCAAGCGTATTCAACAAGGGGCTGAAGGATGCCCAGAAGGTACCCCATTGTATGGGA-TCTGATCTGGGGCCTCGG
IRES-013     ACGTTGTGAGTTGGATAGTTGTGGAAAGAGTCAAATGGCTCACCTCAAGCGTATTCAACAAGGGGCTGAAGGATGCCCAGAAGGTACCCCATTGTATGGGA-TCTGATCTGGGGCCTCGG
IRES-012     ACGTTGTGAGTTGGATAGTTGTGGAAAGAGTCAAATGGCTCACCTCAAGCGTATTCAACAAGGGGCTGAAGGATGCCCAGAAGGTACCCCATTGTATGGGA-TCTGATCTGGGGCCTCGG
IRES-004     ACGTTGTGAGTTGGATAGTTGTGGAAAGAGTCAAATGGCTCCCCTCAAGCGTATTCAACAAGGGGCTGAAGGATGCCCAGAAGGTACCCCATTGTATGGGA-TCTGATCTGGGGCCTCGG
IRES-008     ACGTTGTGAGTTGGATAGTTGTGGAAAGAGTCAAATGGCTCTCCTCAAGCGTATTCAACAAGGGGCTGAAGGATGCCCAGAAGGTACCCCATTGTATGGGA-TCTGATCTGGGGCCTCGG
IRES-015     ACGTTGTGAGTTGGATAGTTGTGGAAAGAGTCAAATGGCTCTCCTCAAGCGTATTCAACAAGGGGCTGAAGGATGCCCAGAAGGTACCCCATTGTATGGGA-TCTGATCTGGGGCCTCGG
IRES-010     ACGTTGTGAGTTGGATAGTTGTGGAAAGAGTCAAATGGCTCTCCTCAAGCGTATTCAACAAGGGGCTGAAGGATGCCCAGAAGGTACCCCATTGTATGGGA-TCTGATCTGGGGCCTCGG
IRES-002     ACGTTGTGAGTTGGATAGTTGTGGAAAGAGTCAAATGGCTCACCTCAAGCGTATTCAACAAGGGGCTGAAGGATGCCCAGAAGGTACCCCATTGTATGGGA-TCTGATCTGGGGCCTCGG
IRES-005     ACGTTGTGAGTTGGATAGTTGTGGAAAGAGTCAAATGGCTCTCCTCAAGCGTATTCAACAAGGGGCTGAAGGATGCCCAGAAGGTACCCCATTGTATGGGA-TCTGATCTGGGGCCTCGG
IRES-003     ACGTTGTGAGTTGGATAGTTGTGGAAAGAGTCAAATGGCTCTCCTCAAGCGTATTCAACAAGGGGCTGAAGGATGCCCAGAAGGTACCCCATTGTATGGGA-TCTGATCTGGGGCCTCGG
IRES-006     ACGTTGTGAGTTGGATAGTTGTGGAAAGAGTCAAATGGCTCTCCTCAAGCGTATTCAACAAGGGGCTGAAGGATGCCCAGAAGGTACCCCATTGTATGGGA-TCTGATCTGGGGCCTCGG
IRES-011     ACGTTGTGAGTTGGATAGTTGTGGAAAGAGTCAAATGGCTCTCCTCAAGCGTATTCAACAAGGGGCTGAAGGATGCCCAGAAGGTACCCCATTGTATGGGA-TCTGATCTGGGGCCTCGG
IRES2-001    ACGTTGTGAGTTGGATAGTTGTGGAAAGAGTCAAATGGCTCTCCTCAAGCGTATTCAACAAGGGGCTGAAGGATGCCCAGAAGGTACCCCATTGTATGGGA-TCTGATCTGGGGCCTCGG
IRES2-002    ACGTTGTGAGTTGGATAGTTGTGGAAAGAGTCAAATGGCTCTCCTCAAGCGTATTCAACAAGGGGCTGAAGGATGCCCAGAAGGTACCCCATTGTATGGGA-TCTGATCTGGGGCCTCGG
IRES2-003    ACGTTGTGAGTTGGATAGTTGTGGAAAGAGTCAAATGGCTCTCCTCAAGCGTATTCAACAAGGGGCTGAAGGATGCCCAGAAGGTACCCCATTGTATGGGA-TCTGATCTGGGGCCTCGG
                                    Stem-Loop I                                                             Stem-Loop J       Stem-Loop K
```

```
IRES-014     TGCACATGCTTTACATGTGTTTAGTCGAGGTTAAAAAAACGTCTAGGCCCCCCGAACCACGGGGACGTGGTTTTCCTTTGAAAAACACGATGATAA-------------
IRES-001     TGCACATGCTTTACATGTGTTTAGTCGAGGTTAAAAAAACGTCTAGGCCCCCCGAACCACGGGGACGTGGTTTTCCTTTGAAAAACACGATGATAA-------------
IRES-013     TGCACATGCTTTACATGTGTTTAGTCGAGGTTAAAAAA-CGTCTAGGCCCCCCGAACCACGGGGACGTGGTTTTCCTTTGAAAAACACGATGATAAT------------
IRES-012     TGCACATGCTTTACATGTGTTTAGTCGAGGTTAAAAAA-CGTCTAGGCCCCCCGAACCACGGGGACGTGGTTTTCCTTTGAAA--------------------------
IRES-004     TGCACATGCTTTTCATGTGTTTAGTCGAGGTTAAAAAA-CGTCTAGGCCCCCCGAACCACGGGGACGTGGTTTTCCTTTGAAAAACACGATGATAATA-----------
IRES-008     TGCACATGCTTTACATGTGTTTAGTCGAGGTTAAAAAAACGTCTAGGCCCCCCGAACCACGGGGACGTGGTTTTCCTTTGAAAAACACGATGATAATA-----------
IRES-015     TGCACATGCTTTACATGTGTTTAGTCGAGGTTAAAAAAACGTCTAGGCCCCCCGAACCACGGGGACGTGGTTTTCCTTTGAAAAACACGATGATCAT------------
IRES-010     TGCACATGCTTTACATGTGTTTAGTCGAGGTTAAAAAAACGTCTAGGCCCCCCGAACCACGGGGACGTGGTTTTCCTTTGAAAAACACGAT------------------
IRES-002     TGCACATGCTTTACATGTGTTTAGTCGAGGTTAAAAAA-CGTCTAGGCCCCCCGAACCACGGGGACGTGGTTTTCCTTTGAAAAACACGATGATAA-------------
IRES-005     TGCACATGCTTTACATGTGTTTAGTCGAGGTTAAAAAA-CGTCTAGGCCCCCCGAACCACGGGGACGTGGTTTTCCTTTGAAAAACACGATGATAA-------------
IRES-003     TGCACATGCTTTACATGTGTTTAGTCGAGGTTAAAAAAACGTCTAGGCCCCCCGAACCACGGGGACGTGGTTTTCCTTTGAAAAACACGATGATAA-------------
IRES-006     TACACATGCTTTACATGTGTTTAGTCGAGGTTAAAAAAACGTCTAGGCCCCCCGAACCACGGGGACGTGGTTTTCCTTTGAAAAACACGATGATAA-------------
IRES-011     TACACATGCTTTACATGTGTTTAGTCGAGGTTAAAAAAACGTCTAGGCCCCCCGAACCACGGGGACGTGGTTTTCCTTTGAAAAACACGATGATAA-------------
IRES2-001    TACACATGCTTTACATGTGTTTAGTCGAGGTTAAAAAAACGTCTAGGCCCCCCGAACCACGGGGACGTGGTTTTCCTTTGAAAAACACGATGATAATATGGCCACAACCATG
IRES2-002    TACACATGCTTTACATGTGTTTAGTCGAGGTTAAAAAAACGTCTAGGCCCCCCGAACCACGGGGACGTGGTTTTCCTTTGAAAAACACGATGATAATATGGCCACAACCATG
IRES2-003    TGCACATGCTTTACATGTGTTTAGTCGAGGTTAAAAAAACGTCTAGGCCCCCCGAACCACGGGGACGTGGTTTTCCTTTGAAAAACACGATGATAATATGGCCACAACCATG
                       Stem-Loop K           A Bulge Stem-Loop J      Stem-Loop L                          834
```

Red highlighting indicates the IRES borders were mis-annotated. The sequence actually includes the highlighted region.

IRES-009 is derived from foot and mouth disease virus (FMDV) and does not align well with other IRES variants derived from encephalomyocarditis virus (EMCV). However both EMCV and FMDV are picornaviruses and utilize Group III IRES, which initiate translation directly at the START codon without ribosome scanning. This means that the placement of the START codon is important for translation efficiency. The native START codon is highlighted in green. An alternative START codon is highlighted in cyan.

The 5′ portion of IRES is dispensable for function up to stem-loop H (not shown), but interference of stem-loop H formation can occur in constructs in which the first cistron is too close. Therefore, it is preferable to include the 5′ stem-loops as spacers.

IRES-002, -004, -005, -012 and -013 have wild-type number of A (A6) in the oligo(A) bulge at the K-J border. This bulge is important for interaction with eIF4G. A7 (e.g. in pIRES) has reduced translational activity compared to A6 (Bochkov and Palmenberg. 2006. Biotechniques 41:283-292). Because A6 and A7 variants have slightly different interactions with the translation machinery, they show different activities in different cell types.

The position of the START codon of the downstream ORF is also important for translational efficiency. Translation of the polypeptide from the native START codon at position 834 (native IRES numbering) provides optimal spacing. Including the first few amino acids of native IRES (IRES2 variants) allows the leader polypeptide to interact properly with the ribosome for the most efficient translation. Using the IRES START codon at position 826 reduces translational efficiency due to steric constraints on this leader peptide ribosome interaction. Moreover, placing the start codon too far downstream in a MCS reduces translation efficiency as the ribosome does not scan in Group III IRESs.

Structures are from Kaminski and Jackson (1998) *RNA* 4:626-638. Invariant stem-loops are not shown.

IRES-007 is identical to IRES-008 but reverse complement – IRES-007 was merged with -008

Key ATGC = change in nucleotide relative to consensus sequence – non-coding sequence

Sequence Alignments

Un-Annotated Promoters

Alignments do not include all plasmids carrying the un-annotated promoter

Un-annotated AmpR promoters (colored segments = vector backbone)

```
Name of Plasmid     Alignment
Or Feature
AmpR_prom-009       CGCGGAACCCCTATTTGTTTATTTTTCTAAATACATTCAAATATGTATCCGCTCATGAGACAATAACCCT-GATAAATGCTTCAATAAT-ATTGAAAAAGGAAGAGT
AmpR_prom-001       CGCGGAACCCCTATTTGTTTATTTTTCTAAATACATTCAAATATGTATCCGCTCATGAGACAATAACCCT-GATAAATGCTTCAATAAT--TTGAAAAAGGAAGAGT
AmpR_prom-006       CGCGGAACCCCTATTTGTTTATTTTTCTAAATACATTCAAATATGTATCCGCTCATGAGACAATAACCCT-GATAAATGCTTCAATAAT-ATTGAAAAAGGAAGA--
AmpR_prom-008       CGCGGGACCCCTATTTGTTTATTTTTCTAAATACATTCAAATATGTATCCGCTCATGAGACAATAACCCT-GATAAATGCTTCAATAAT-ATTGAAAAAGGAAGAGT
AmpR_prom-011       CGCGGAACCCCTATTTGTTTATTTTTCTAAATACATTCAAATATGTATCCGCTCATGAGACAATAACCCT-GATAAATGCTTCAATAAT-ATTGAAAAAAGAAGAGT
AmpR_prom-002       CGCGGAACCCCTATTTGTTTATTTTTCTAAATACATTCAAATATGTATCCGCTCATGAGACAATAACCCTTGATAAATGCTTCAATAAT-ATTGAAAAAGGAAGAGT
AmpR_prom-012       CGCGGAACCCCTATTTCTTTATTTTTCTAAATACATTCAAATATGTATCCGCTCATGAGACAATAACCCT-GATAAATGCTTCAATAAT-ATTGAAAAAGGAAGAGT
pCMV-Cypridina_Luc  ACACTGACCCCTATTTGTTTATTTTTCTAAATACATTCAAATATGTATCCGCTCATGAGACAATAACCCT-GATAAATGCTTCAATAAT-ATTGAAAAAGGAAGAAT
pTK-Cypridina       ACACTGACCCCTATTTGTTTATTTTTCTAAATACATTCAAATATGTATCCGCTCATGAGACAATAACCCT-GATAAATGCTTCAATAAT-ATTGAAAAAGGAAGAAT
pET-43.1a(+)        TGGTCATGACCTATTTGTTTATTTTTCTAAATACATTCAAATATGTATCCGCTCATGAGACAATAACCCT-GATAAATGCTTCAATCATGATTGAAAAAGGAAGAGT
pET-44a(+)          TGGTCATGACCTATTTGTTTATTTTTCTAAATACATTCAAATATGTATCCGCTCATGAGACAATAACCCT-GATAAATGCTTCAATCATGATTGAAAAAGGAAGAGT
pET-45b(+)          TGGTCATGACCTATTTGTTTATTTTTCTAAATACATTCAAATATGTATCCGCTCATGAGACAATAACCCT-GATAAATGCTTCAATCATGATTGAAAAAGGAAGAGT
pET-51b(+)          TGGTCATGACCTATTTGTTTATTTTTCTAAATACATTCAAATATGTATCCGCTCATGAGACAATAACCCT-GATAAATGCTTCAATCATGATTGAAAAAGGAAGAGT
pET-52b(+)          TGGTCATGACCTATTTGTTTATTTTTCTAAATACATTCAAATATGTATCCGCTCATGAGACAATAACCCT-GATAAATGCTTCAATCATGATTGAAAAAGGAAGAGT
AmpR_prom-005       -------------TTTGTTTATTTTTCTAAATACATTCAAATATGTATCCGCTCATGAGACAATAACCCT-GATAAATGCTTCAATCATGATTGAAAAAGGAAGAGT
AmpR_prom-003       -------------TTTGTTTATTTTTCTAAATACATTCAAATATGTATCCGCTCATGAGACAATAACCCT-GATAAATGCTTCAATAAT-ATTGAAAAAGGAAGAGT
AmpR_prom-010       -------------TTTGTTTATTTTTCTAAATACATTCAAATATGTATCCGCTCATGAGACAATAACCCT-GATAAATGCTTCAATAAT-AATGAAAAAGGAAGAGT
AmpR_prom-004       ---------------------------------CATTCAAATATGTATCCGCTCATGAGACAATAACCCT-GATAAATGCTTCAATAAT-ATTGAAAAAGGAAGAGT
AmpR_prom-007       ---------------------------------CCTTCAAATATGTATCCGCTCAGGAGACAATAACCCT-GATAAATGCTTCAATAAT-ATTGAAAAAGGAAGAGT
pCR4-TOPO           TCTACGGGGTCTGACGCTCAGTGGAACGAAAACTCACGTTAAGGGATTTTGGTCATGAGACAATAACCCT-GATAAATGCTTCAATAAT-ATTGAAAAAGGAAGAGT
pCAS-Guide          GCCCGTGTCTCAAAATCTCTGATGTTACATTGCACAAGATAAAATAATATCATCATGAGACAATAACCCT-GATAAATGCTTCAATAAT-ATTGAAAAAGGAAGAGT
pCMV_SPORT          AATTATGTGCTGTGTAGGGATCGCTGGTATCAAATATGTGTGCCCACCCCTGGCATGAGACAATAACCCT-GATAAATGCTTCAATAAT-ATTGAAAAAGGAAGAGT
pCMV_SPORT2         AATTATGTGCTGTGTAGGGATCGCTGGTATCAAATATGTGTGCCCACCCCTGGCATGAGACAATAACCCT-GATAAATGCTTCAATAAT-ATTGAAAAAGGAAGAGT
pZL1                AATTATGTGCTGTGTAGGGATCGCTGGTATCAAATATGTGTGCCCACCCCTGGCATGAGACAATAACCCT-GATAAATGCTTCAATAAT-ATTGAAAAAGGAAGAGT
pTriEx-1.1          GCGCGTTTATACACATCTTGGGATTTGATTAAAAGATGCAGGAAACGCGCGGACATGAGACAATAACCCT-GATAAATGCTTCAATAAT-ATTGAAAAAGGAAGAGT
pTriEx-2            GCGCGTTTATACACATCTTGGGATTTGATTAAAAGATGCAGGAAACGCGCGGACATGAGACAATAACCCT-GATAAATGCTTCAATAAT-ATTGAAAAAGGAAGAGT
pQE-TriSystem_5     GCGCGTTTATACACATCTTGGGATTTGATTAAAAGATGCAGGAAACGCGCGGACATGAGACAATAACCCT-GATAAATGCTTCAATAAT-ATTGAAAAAGGAAGAGT
pQE-TriSystem_6     GCGCGTTTATACACATCTTGGGATTTGATTAAAAGATGCAGGAAACGCGCGGACATGAGACAATAACCCT-GATAAATGCTTCAATAAT-ATTGAAAAAGGAAGAGT
pDream2.1_MCS       GCGCGTTTATACACATCTTGGGATTTGATTAAAAGATGCAGGAAACGCGCGGACATGAGACAATAACCCT-GATAAATGCTTCAATAAT-ATTGAAAAAGGAAGAGT
pQE-TriSystem       ----GTTTATACACATCTTGGGATTTGATTAAAAGATGCAGGAAACGCGCGGACATGAGACAATAACCCT-GATAAATGCTTCAATAAT-ATTGAAAAAGGAAGAGT
pQE-TriSys_His-Str  ----GTTTATACACATCTTGGGATTTGATTAAAAGATGCAGGAAACGCGCGGACATGAGACAATAACCCT-GATAAATGCTTCAATAAT-ATTGAAAAAGGAAGAGT
pGL4.70-hRluc       -GCGGCCTGTCCAATACCTCCCGTACCTTAATATTACTTACTTATCCTTGAGAGACGTACTAGTAACCCT-GATAAATGCTTCAATAAT-ATTGAAAAAGGACGAGT
pLightSwitch_Prom   -GCGGCCTGTCCAATACCTCCCGTACCTTAATATTACTTACTTATCCTTGAGAGACGTACTAGTAACCCT-GATAAATGCTTCAATAAT-ATTGAAAAAGGACGAGT
pNL1.1-Nluc         -GCGGCCTGTCCAATACCTCCCGTACCTTAATATTACTTACTTATCCTTGAGAGACGTACTAGTAACCCT-GATAAATGCTTCAATAAT-ATTGAAAAAGGACGAGT
pNL2.1-Nluc         -GCGGCCTGTCCAATACCTCCCGTACCTTAATATTACTTACTTATCCTTGAGAGACGTACTAGTAACCCT-GATAAATGCTTCAATAAT-ATTGAAAAAGGACGAGT
pGL4.10-luc2        -GCGGCCTGTCCAATACCTCCCGTACCTTAATATTACTTACTTATCCTTGAGAGACGTACTAGTAACCCT-GATAAATGCTTCAATAAT-ATTGAAAAAGGACGAGT
Lafmid_BA           CTTATACAATCTTCCTGTTTTTGGGGCTTTTCTGATTATCAACCGGGGTACATATGATTGACATGCTAGTTTTACGATTACCGTTCATCGATTGAAAAAGGAAGAGT
pLIC-SGC1           AAGCTCTAAATCGGGGGCTCCCTTTAGGGTTCCGATTTAGTGCTTTACGGCACCTCGACCCCAAAAAACTTGATTAGGGTGATGGTTCACATTGAAAAAGGAAGAGT
pIB_V5-His-DEST     CGAGGAGCAGGACTGACACGTCCCGGGAGATCTGCATGTCTACTAAACTCACAAATTAGAGCTTCAATTTAATTATATCAGTTATTACCCATTGAAAAAGGAAGAGT
pIB_V5-His          CGAGGAGCAGGACTGACACGTCCCGGGAGATCTGCATGTCTACTAAACTCACAAATTAGAGCTTCAATTTAATTATATCAGTTATTACCCATTGAAAAAGGAAGAGT
pMIB_V5-His_A       CGAGGAGCAGGACTGACACGTCCCGGGAGATCTGCATGTCTACTAAACTCACAAATTAGAGCTTCAATTTAATTATATCAGTTATTACCCATTGAAAAAGGAAGAGT
pYC6_CT             CGAGGAGCAGGACTGACACGTCCCGGGAGATCTGCATGTCTACTAAACTCACAAATTAGAGCTTCAATTTAATTATATCAGTTATTACCCATTGAAAAAGGAAGAGT
pYES6_CT            CGAGGAGCAGGACTGACACGTCCCGGGAGATCTGCATGTCTACTAAACTCACAAATTAGAGCTTCAATTTAATTATATCAGTTATTACCCATTGAAAAAGGAAGAGT
pYC2_CT             ------------AAAACTGTATTATAAGTAAATGCATGTATACTAAACTCACAAATTAGAGCTTCAATTTAATTATATCAGTTATTACCCATTGAAAAAGGAAGAGT
pYES2_CT            ------------AAAACTGTATTATAAGTAAATGCATGTATACTAAACTCACAAATTAGAGCTTCAATTTAATTATATCAGTTATTACCCATTGAAAAAGGAAGAGT
pCR2.1-TOPO         ------------------------------------------------------------------------------------------ATTGAAAAAGGAAGAGT

                                                      -35  bla TEM prom   -10                            RBS
```

Sequences were trimmed at the maximum length shown or at the junction of an adjacent feature (producing the shorter sequences). The boxed -35 and -10 regions correspond to those of native *bla TEM* from *E. coli* Tn3.

pCAS-Guide uses the KanR (*aph(3')-Ia*) promoter from Tn5 (brown region; -35 and -10 sequences highlighted in yellow).
The violet region for pCMV-SPORT, pCMV-SPORT2 and pZL1 are part of a defunct incA (RepA binding sites) region, but BPROM promoter prediction algorithm suggests ATGTGT forms a viable -35 region with the intact -10.
The pink region for the pTriEx and pQE-TriSystem series are part of baculovirus recombination region (lef2), but BPROM suggests ATGCAG forms a viable -35 region with the intact -10.
The light green region for the luciferase vectors appears to be an artificial promoter (there are several synthetic sequences in these plasmids), but BPROM does not predict a -35 and -10.
The blue region in Lafmid_BA corresponds to the 3'end of M13 ori-003 and is predicted by BPROM to form -35 and -10 (yellow highlight).
The orange sequence of pLIC-SGC1 is identical to a portion of f1 origin. The highlighted areas correspond to -35 and -10 sites for RNApol σ^{70} which normally produces an RNA template for DNA polymerase, but has weak promoter activity when the DNA is double-stranded (Higashitani et al. 1997. PNAS USA 94:2909).
The cyan region of the PIB and pMIB series, and pYC and pYES series is *S. cerevisiae URA3* 3' flanking sequence, but is predicted by BPROM to form a viable -35 and -10 (yellow highlight).
The AmpR of pCR2.1-TOPO is produced as a bicistronic transcript with NeoR/KanR from the NeoR/KanR promoter.

Key   ATGC = change in nucleotide relative to consensus sequence – non-coding sequence

Sequence Alignments

Un-annotated CAT promoters (colored segments = vector backbone)

```
Name of Plasmid     Alignment
Or Feature
CAT_prom-001        TGATCGG-CACGTAAGAGGTTCCAACTTTCACCATAATGAAATAAGATCACTACCGGGCGTATTTTTTGAGTTGTCGAGATTTTCAGGAGCTAAGGAAGCTAAA
CAT_prom-002        TGATCGG-CACGTAAGAGGGTCCAACTTTCACCATAATGAAATAAGATCACTACCGGGCGTATTTTTTGAGTTATCGAGATTTTCAGGAGCTAAGGAAGCTAAA
CAT_prom-003        TGATCGG-CACGTAAGAGGTTCCAACTTTCACCATAATGAAATAAGATCACTACCGGGCGTATTTTT-GAGTTATCGAGATTTTCAGGAGCTAAGGAAGCTAAA
CAT_prom-004        TGATCGG-CACGTAAGAGGTTCCAACTTTCACCATAATGAAATAAGATCACTACCGGGCGTATTTTTTGAGTTATCGAGATTTTCAGGAGCTAAGGAAGCTAAA
pSMART_HCKan(KanR)  TGATCGG-CACGTAAGAGGTTCCAACTTTCACCATAATGAAATAAGATCACTACCGGGCGTATTTTTTGAGTTATCGAGATTTTCAGGAGCTAAGGAAGCTAAA
pSMART_LCKan(KanR)  TGATCGG-CACGTAAGAGGTTCCAACTTTCACCATAATGAAATAAGATCACTACCGGGCGTATTTTTTGAGTTATCGAGATTTTCAGGAGCT------------
pSB1C3(CmR)         TGATCGGGCACGTAAGAGGTTCCAACTTTCACCATAATGAAATAAGATCACTACCGGGCGTATTTTTTGAGTTATCGAGATTTTCAGGAGCTAAGGAAGCTAAA
pRANGER-BTB-1(AmpR) ---------ACGTAAGAGGTTCCAACTTTCACCATAATGAAATAAGATCACTACCGGGCGTATTTTTTGAGTTATCGAGATTTTCAGGAGCTAAGGAAGCTAAA
pRANGER-BTB-2(KanR) ---------ACGTAAGAGGTTCCAACTTTCACCATAATGAAATAAGATCACTACCGGGCGTATTTTTTGAGTTATCGAGATTTTCAGGAGCTAAGGAAGCTAAA
pRANGER-BTB-3(CmR)  ---------ACGTAAGAGGTTCCAACTTTCACCATAATGAAATAAGATCACTACCGGGCGTATTTTTTGAGTTATCGAGATTTTCAGGAGCTAAGGAAGCTAAA
pRANGER-BTB-5(TpR)  ---------ACGTAAGAGGTTCCAACTTTCACCATAATGAAATAAGATCACTACCGGGCGTATTTTTTGAGTTATCGAGATTTTCAGGAGCTAAGGAAGCTAAA
pRFP-C-RS(CmR)      ATATGTATCCGCTCATGACTCATGTTTGACAGCTTATCATCGATAAGCTAGCGGCCGCTAGCTTTAATGAGTTATCGAGATTTTCAGGAGCTAAGGAAGCTAAA
pVP13(CmR)          -------------------------------------------------------------CCAAGCTAGCTTGGCGAGATTTTCAGGAGCTAAGGAAGCTAAA
pVP16(CmR)          -------------------------------------------------------------CCAAGCTAGCTTGGCGAGATTTTCAGGAGCTAAGGAAGCTAAA
pVP33K(CmR)         -------------------------------------------------------------CCAAGCTAGCTTGGCGAGATTTTCAGGAGCTAAGGAAGCTAAA
pVP56K(CmR)         -------------------------------------------------------------CCAAGCTAGCTTGGCGAGATTTTCAGGAGCTAAGGAAGCTAAA
pDNR-LIB(CmR)       ----------------------------------------------------------------------------GGATCTTTCAGGAGCTAAGGAAGCTAAA
pDNR-Dual(CmR)      ----------------------------------------------------------------------------GGATCTTTCAGGAGCTAAGGAAGCTAAA
                                                                                                *    ***********

                               -35     CAT prom     -10
```

Sequences were trimmed at the maximum length shown or at the junction of an adjacent feature (producing the shorter sequences).
These CAT promoters drive expression of various marker genes indicated in parentheses.

In pSMART_LCKan, CmR is the only selectable marker. Therefore this sequences must be active.
The cyan portion of pRFP-C-RS is pBR322 backbone but is predicted by BPROM to have -35 and -10 sequences (highlighted in yellow).
The pVP series has two CmR genes – one behind lac UV5 promoter, and the other with the above 5′ region, which is not active.
pDNR-LIB/Dual have only CmR as the selectable marker. In the case of pDNR-LIB, CmR is head-to-tail with SacB and is likely produced from a bicistronic transcript with SacB. However, in pDNR-Dual, CmR and SacB are head-to-head. In this case, it seems that the SacB upstream region includes a bidirectional promoter that can drive expression of CmR. Alternatively, the loxP site between CmR and SacB in both plasmids has cryptic promoter activity.

Un-annotated NeoR/KanR promoters (colored segments = unknown sequence)

```
Name of Plasmid     Alignment
Or Feature
pREP4               TCTGGTAAGGTTGGGAAGCCCTGCAAAGTAAACTGGATGGCTTTCTTGCCGCCAAGGATCTGATGGCGCAGGGGATCAAGATCTGATCAAGAGACAGGAT---GACGGTCGTTTCGC
pVP56K              TCTGGTAAGGTTGGGAAGCCCTGCAAAGTAAACTGGATGGCTTTCTTGCCGCCAAGGATCTGATGGCGCAGGGGATCAAGATCTGATCAAGAGACAGGAT---GACGGTCGTTTCGC
pVP68K              TCTGGTAAGGTTGGGAAGCCCTGCAAAGTAAACTGGATGGCTTTCTTGCCGCCAAGGATCTGATGGCGCAGGGGATCAAGATCTGATCAAGAGACAGGAT---GACGGTCGTTTCGC
pVP65K              TCTGGTAAGGTTGGGAAGCCCTGCAAAGTAAACTGGATGGCTTTCTTGCCGCCAAGGATCTGATGGCGCAGGGGATCAAGATCTGATCAAGAGACAGGAT---GACGGTCGTTTCGC
pVP81K              TCTGGTAAGGTTGGGAAGCCCTGCAAAGTAAACTGGATGGCTTTCTTGCCGCCAAGGATCTGATGGCGCAGGGGATCAAGATCTGATCAAGAGACAGGAT---GACGGTCGTTTCGC
pVP33K              TCTGGTAAGGTTGGGAAGCCCTGCAAAGTAAACTGGATGGCTTTCTTGCCGCCAAGGATCTGATGGCGCAGGGGATCAAGATCTGATCAAGAGACAGGAT---GACGGTCGTTTCGC
pSpark_III          TCTGGTAAGGTTGGGAAGCCCTGCAAAGTAAACTGGATGGCTTTCTTGCCGCCAAGGATCTGATGGCGCAGGGGATCAAGCTCTGATCAAGAGACAGGAT---GAGGATCGTTTCGC
pCAMBIA5105         TCTGGTAAGGTTGGGAAGCCCTGCAAAGTAAACTGGATGGCTTTCTTGCCGCCAAGGATCTGATGGCGCAGGGGATCAAGATCTGATCAAGAGACAGGAT---GAGGATCGTTTCGC
pZErO-2             TCTGGTAAGGTTGGGAAGCCCTGCAAAGTAAACTGGATGGCTTTCTCGCCGCCAAGGATCTGATGGCGCAGGGGATCAAGATCTGATCAAGAGACAGGAT---GAGGATCGTTTCGC
pSF-pA-PromMCS-Fluc GGCAGCGTACCGATCTGTTTAAACCTAGATATTGATAGTCTGATCGGTCAACGTATAATCGAGTCCTAGCTTTTGCAAACATCT-ATCAAGAGACAGGATCAGCAGGAGGCTTTCGC
pSF-pA-CMVe-Rluc    GGCAGCGTACCGATCTGTTTAAACCTAGATATTGATAGTCTGATCGGTCAACGTATAATCGAGTCCTAGCTTTTGCAAACATCT-ATCAAGAGACAGGATCAGCAGGAGGCTTTCGC
pSF-pA-PromMCS-Rluc GGCAGCGTACCGATCTGTTTAAACCTAGATATTGATAGTCTGATCGGTCAACGTATAATCGAGTCCTAGCTTTTGCAAACATCT-ATCAAGAGACAGGATCAGCAGGAGGCTTTCGC
pSF-PromMCS-Fluc    GGCAGCGTACCGATCTGTTTAAACCTAGATATTGATAGTCTGATCGGTCAACGTATAATCGAGTCCTAGCTTTTGCAAACATCT-ATCAAGAGACAGGATCAGCAGGAGGCTTTCGC
pSF-PromMCS-Rluc    GGCAGCGTACCGATCTGTTTAAACCTAGATATTGATAGTCTGATCGGTCAACGTATAATCGAGTCCTAGCTTTTGCAAACATCT-ATCAAGAGACAGGATCAGCAGGAGGCTTTCGC
pSF-pA-CMVe-Fluc    GGCAGCGTACCGATCTGTTTAAACCTAGATATTGATAGTCTGATCGGTCAACGTATAATCGAGTCCTAGCTTTTGCAAACATCT-ATCAAGAGACAGGATCAGCAGGAGGCTTTCGC
pSF-CMVe-Fluc       GGCAGCGTACCGATCTGTTTAAACCTAGATATTGATAGTCTGATCGGTCAACGTATAATCGAGTCCTAGCTTTTGCAAACATCT-ATCAAGAGACAGGATCAGCAGGAGGCTTTCGC
pSF-CMVe-Rluc       GGCAGCGTACCGATCTGTTTAAACCTAGATATTGATAGTCTGATCGGTCAACGTATAATCGAGTCCTAGCTTTTGCAAACATCT-ATCAAGAGACAGGATCAGCAGGAGGCTTTCGC
                                                                                                     *** ***************      *    ******

                                                    -35?                  -10?
```

The top 9 promoters show identity to the native NeoR/KanR (*nptII*) promoter from *E. coli* transposon *Tn5*.

BPROM did not identify putative -35 and -10 sequences in the native promoter.

The pSF series from Oxford Genetics appear to have a related promoter, but this sequence does not appear to be natural as it generates no hits from homology searches against public databases. Highlighted regions are potential -35 and -10 sequences (BPROM). This promoter is also used to drive AmpR in pSF-Core, pSF-CMV-Fluc and pSF-CMV-Rluc.

Key ATGC = change in nucleotide relative to consensus sequence – non-coding sequence

Sequence Alignments

Un-annotated KanR promoters (colored segments = plasmid backbone)

```
Name of Plasmid     Alignment
Or Feature
pShuttle2           ACAAAGCCACGTTGTGTCTCAAAATCTCTGATGTTACATTGCACAAGATAAAAATATATCATCATGAACAATAAAACTGTCTGCTTACATAAACAGTAATACAAGGGGTGTT
pSIREN-Shuttle      ACAAAGCCACGTTGTGTCTCAAAATCTCTGATGTTACATTGCACAAGATAAAAATATATCATCATGAACAATAAAACTGTCTGCTTACATAAACAGTAATACAAGGGGTGTT
pACYC177            ACAAAGCCACGTTGTGTCTCAAAATCTCTGATGTTACATTGCACAAGATAAAAATATATCATCATGAACAATAAAACTGTCTGCTTACATAAACAGTAATACAAGGGGTGTT
pMCSG77             ACAAAGCCACGTTGTGTCTCAAAATCTCTGATGTTACATTGCACAAGATAAAAATATATCATCATGAACAATAAAACTGTCTGCTTACATAAACAGTAATACAAGGGGTGTT
pHSG298             ACAAAGCCACGTTGTGTCTCAAAATCTCTGATGTTACATTGCACAAGATAAAAATATATCATCATGAACAATAAAACTGTCTGCTTACATAAACAGTAATACAAGGGGTGTT
pHSG299             ACAAAGCCACGTTGTGTCTCAAAATCTCTGATGTTACATTGCACAAGATAAAAATATATCATCATGAACAATAAAACTGTCTGCTTACATAAACAGTAATACAAGGGGTGTT
pGreenII_0049       ACAAAGCCACGTTGTGTCTCAAAATCTCTGATGTTACATTGCACAAGATAAAAATATATCATCATGAACAATAAAACTGTCTGCTTACATAAACAGTAATACAAGGGGTGTT
pGreen_0029         ACAAAGCCACGTTGTGTCTCAAAATCTCTGATGTTACATTGCACAAGATAAAAATATATCATCATGAACAATAAAACTGTCTGCTTACATAAACAGTAATACAAGGGGTGTT
pGreen              ACAAAGCCACGTTGTGTCTCAAAATCTCTGATGTTACATTGCACAAGATAAAAATATATCATCATGAACAATAAAACTGTCTGCTTACATAAACAGTAATACAAGGGGTGTT
pKF_18k-2           ACAAAGCCACGTTGTGTCTCAAAATCTCTGATGTTACATTGCACAAGATAAAAATATATCATCATGAACAATAAAACTGTCTGCTTACATAAACAGTAATACAAGGGGTGTT
pKF_19k-2           ACAAAGCCACGTTGTGTCTCAAAATCTCTGATGTTACATTGCACAAGATAAAAATATATCATCATGAACAATAAAACTGTCTGCTTACATAAACAGTAATACAAGGGGTGTT
pOSIP-KT            TGTAACGCACGTTGTGTCTCAAAATCTCTGATGTTACATTGCACAAGATAAAAATATATCATCATGAACAATAAAACTGTCTGCTTACATAAACAGTAATACAAGGGGTGTT
pOSIP-KP            TGTAACGCACGTTGTGTCTCAAAATCTCTGATGTTACATTGCACAAGATAAAAATATATCATCATGAACAATAAAACTGTCTGCTTACATAAACAGTAATACAAGGGGTGTT
pOSIP-KL            TGTAACGCACGTTGTGTCTCAAAATCTCTGATGTTACATTGCACAAGATAAAAATATATCATCATGAACAATAAAACTGTCTGCTTACATAAACAGTAATACAAGGGGTGTT
pOSIP-KO            TGTAACGCACGTTGTGTCTCAAAATCTCTGATGTTACATTGCACAAGATAAAAATATATCATCATGAACAATAAAACTGTCTGCTTACATAAACAGTAATACAAGGGGTGTT
pOSIP-KH            TGTAACGCACGTTGTGTCTCAAAATCTCTGATGTTACATTGCACAAGATAAAAATATATCATCATGAACAATAAAACTGTCTGCTTACATAAACAGTAATACAAGGGGTGTT
pOSIP-KC            CTACTTACACGTTGTGTCTCAAAATCTCTGATGTTACATTGCACAAGATAAAAATATATCATCATGAACAATAAAACTGTCTGCTTACATAAACAGTAATACAAGGGGTGTT
pUC57-Kan           GCAGCTCTGGCCCGTGTCTCAAAATCTCTGATGTTACATTGCACAAGATAAAAATATATCATCATGAACAATAAAACTGTCTGCTTACATAAACAGTAATACAAGGGGTGTT
pCONR201            GCAGCTCTGGCCCGTGTCTCAAAATCTCTGATGTTACATTGCACAAGATAAAAATATATCATCATGAACAATAAAACTGTCTGCTTACATAAACAGTAATACAAGGGGTGTT
pMCentr2            GCAGCTCTGGCCCGTGTCTCAAAATCTCTGATGTTACATTGCACAAGATAAAATAATATCATCATGAACAATAAAACTGTCTGCTTACATAAACAGTAATACAAGGGGTGTT
pMCentr3            GCAGCTCTGGCCCGTGTCTCAAAATCTCTGATGTTACATTGCACAAGATAAAATAATATCATCATGAACAATAAAACTGTCTGCTTACATAAACAGTAATACAAGGGGTGTT
pQE-80L             TTCTCGAGGTGAAGACGAAAGGGCCTCGTGATACGCCTATTTTTATAGGTTAATGTCATGGTCATGAACAATAAAACTGTCTGCTTACATAAACAGTAATACAAGGGGTGTT
pQE-81L             TTCTCGAGGTGAAGACGAAAGGGCCTCGTGATACGCCTATTTTTATAGGTTAATGTCATGGTCATGAACAATAAAACTGTCTGCTTACATAAACAGTAATACAAGGGGTGTT
pQE-82L             TTCTCGAGGTGAAGACGAAAGGGCCTCGTGATACGCCTATTTTTATAGGTTAATGTCATGGTCATGAACAATAAAACTGTCTGCTTACATAAACAGTAATACAAGGGGTGTT
pET-9a              TTGATCTTTTCTACGGGGTCTGACGCTCAGTGGAACGAAAACTCACGTTAAGGGATTTTGGTCATGAACAATAAAACTGTCTGCTTACATAAACAGTAATACAAGGGGTGTT
pET-9b              TTGATCTTTTCTACGGGGTCTGACGCTCAGTGGAACGAAAACTCACGTTAAGGGATTTTGGTCATGAACAATAAAACTGTCTGCTTACATAAACAGTAATACAAGGGGTGTT
pET-9c              TTGATCTTTTCTACGGGGTCTGACGCTCAGTGGAACGAAAACTCACGTTAAGGGATTTTGGTCATGAACAATAAAACTGTCTGCTTACATAAACAGTAATACAAGGGGTGTT
pET-9d              TTGATCTTTTCTACGGGGTCTGACGCTCAGTGGAACGAAAACTCACGTTAAGGGATTTTGGTCATGAACAATAAAACTGTCTGCTTACATAAACAGTAATACAAGGGGTGTT
pGFP-B-RS           GGAAATGTGCGCGGAACCCCTATTTGTTTATTTTTCTAAATACATTCAAATATGTATCCGCTCATGAACAATAAAACTGTCTGCTTACATAAACAGTAATACAAGGGGTGTT
pGFP-V-RS           GGAAATGTGCGCGGAACCCCTATTTGTTTATTTTTCTAAATACATTCAAATATGTATCCGCTCATGAACAATAAAACTGTCTGCTTACATAAACAGTAATACAAGGGGTGTT
pCMV6-Entry2        GGAAATGTGCGCGGAACCCCTATTTGTTTATTTTTCTAAATACATTCAAATATGTATCCGCTCATGAACAATAAAACTGTCTGCTTACATAAACAGTAATACAAGGGGTGTT
AmpR_prom-009       ---------CGCGGAACCCCTATTTGTTTATTTTTCTAAATACATTCAAATATGTATCCGCTCATGA...
                                                                                      ***************************************************
                                                     -35  aph(3')-Ia prom   -10                                            RBS
```

These promoters are the natural *aph(3')-Ia* promoter from *E. coli Tn5* and all drive expression of KanR (*aph(3')-IaI*). Highlighting shows putative -35 and -10 sequences (BPROM). The pGFP and pCMV series have a hybrid promoter with AmpR promoter (cyan highlighting) from replacing AmpR with KanR during plasmid construction (TCATGA is a BspHI restriction site).

```
Name of Plasmid     Alignment
Or Feature
                    GATCACCGCGGTTTCAAAATCGGCTCCGTCGATACTATGTTATACGC.GAACAGTGAATTGGAGTTCGTCTTGTTATAATTAGCTTCTTGGGGTATCTTTAAATACTGTAGAAAAGAGGAAGGAAATAATAA
pCAMBIA0305.1       GATCACCGCGGTTTCAAAATCGGCTCCGTCGATACTATGTTATACGC.GAACAGTGAATTGGAGTTCGTCTTGTTATAATTAGCTTCTTGGGGTATCTTTAAATACTGTAGAAAAGAGGAAGGAAATAATAA
pCAMBIA0305.2       GATCACCGCGGTTTCAAAATCGGCTCCGTCGATACTATGTTATACGC.GAACAGTGAATTGGAGTTCGTCTTGTTATAATTAGCTTCTTGGGGTATCTTTAAATACTGTAGAAAAGAGGAAGGAAATAATAA
pCAMBIA03080        GATCACCGCGGTTTCAAAATCGGCTCCGTCGATACTATGTTATACGC.GAACAGTGAATTGGAGTTCGTCTTGTTATAATTAGCTTCTTGGGGTATCTTTAAATACTGTAGAAAAGAGGAAGGAAATAATAA
pCAMBIA03090        GATCACCGCGGTTTCAAAATCGGCTCCGTCGATACTATGTTATACGC.GAACAGTGAATTGGAGTTCGTCTTGTTATAATTAGCTTCTTGGGGTATCTTTAAATACTGTAGAAAAGAGGAAGGAAATAATAA
pEarleyGate_100     GATCACCGCGGTTTCAAAATCGGCTCCGTCGATACTATGTTATACGC.GAACAGTGAATTGGAGTTCGTCTTGTTATAATTAGCTTCTTGGGGTATCTTTAAATACTGTAGAAAAGAGGAAGGAAATAATAA
pEarleyGate_101     GATCACCGCGGTTTCAAAATCGGCTCCGTCGATACTATGTTATACGC.GAACAGTGAATTGGAGTTCGTCTTGTTATAATTAGCTTCTTGGGGTATCTTTAAATACTGTAGAAAAGAGGAAGGAAATAATAA
pEarleyGate_102     GATCACCGCGGTTTCAAAATCGGCTCCGTCGATACTATGTTATACGC.GAACAGTGAATTGGAGTTCGTCTTGTTATAATTAGCTTCTTGGGGTATCTTTAAATACTGTAGAAAAGAGGAAGGAAATAATAA
pFGC5941            GATCACCGCGGTTTCAAAATCGGCTCCGTCGATACTATGTTATACGC.GAACAGTGAATTGGAGTTCGTCTTGTTATAATTAGCTTCTTGGGGTATCTTTAAATACTGTAGAAAAGAGGAAGGAAATAATAA
pBI121              CATAATTGTGGTTTCAAAATCGGCTCCGTCGATACTATGTTATACGC.GAACAGTGAATTGGAGTTCGTCTTGTTATAATTAGCTTCTTGGGGTATCTTTAAATACTGTAGAAAAGAGGAAGGAAATAATAA
pRI_101-AN          CATAATTGTGGTTTCAAAATCGGCTCCGTCGATACTATGTTATACGC.GAACAGTGAATTGGAGTTCGTCTTGTTATAATTAGCTTCTTGGGGTATCTTTAAATACTGTAGAAAAGAGGAAGGAAATAATAA
pRI_101-ON          CATAATTGTGGTTTCAAAATCGGCTCCGTCGATACTATGTTATACGC.GAACAGTGAATTGGAGTTCGTCTTGTTATAATTAGCTTCTTGGGGTATCTTTAAATACTGTAGAAAAGAGGAAGGAAATAATAA
pRI_201-AN          CATAATTGTGGTTTCAAAATCGGCTCCGTCGATACTATGTTATACGC.GAACAGTGAATTGGAGTTCGTCTTGTTATAATTAGCTTCTTGGGGTATCTTTAAATACTGTAGAAAAGAGGAAGGAAATAATAA
pRI_201-ON          CATAATTGTGGTTTCAAAATCGGCTCCGTCGATACTATGTTATACGC.GAACAGTGAATTGGAGTTCGTCTTGTTATAATTAGCTTCTTGGGGTATCTTTAAATACTGTAGAAAAGAGGAAGGAAATAATAA
pRI_909             CATAATTGTGGTTTCAAAATCGGCTCCGTCGATACTATGTTATACGC.GAACAGTGAATTGGAGTTCGTCTTGTTATAATTAGCTTCTTGGGGTATCTTTAAATACTGTAGAAAAGAGGAAGGAAATAATAA
pRI_910             CATAATTGTGGTTTCAAAATCGGCTCCGTCGATACTATGTTATACGC.GAACAGTGAATTGGAGTTCGTCTTGTTATAATTAGCTTCTTGGGGTATCTTTAAATACTGTAGAAAAGAGGAAGGAAATAATAA
pBINPLUS            CATAATTGTGGTTTCAAAATCGGCTCCGTCGATACTATGTTATACGC.GAACAGTGAATTGGAGTTCGTCTTGTTATAATTAGCTTCTTGGGGTATCTTTAAATACTGTAGAAAAGAGGAAGGAAATAATAA
                     ** *  * ***************************************************************************************************************************
                                                                         -35   aphA-3 prom   -10
```

These plasmids all use KanR. These promoters are the natural *aphA-3* (KanR-020) promoter from *Staphylococcus aureus*.

Key ATGC = change in nucleotide relative to consensus sequence – non-coding sequence

Sequence Alignments

Un-annotated SmR promoters

```
Name of Plasmid     Alignment
Or Feature
pDEST20-Pc_prom     ACCCAGTTGACATAAGCCT-GTTCGGTTCGTAAACTGTAATGCAAGTAGCGTA--TGCGCTCACGCAACTGGTCCAGAACCTTGACCGAACGCAGCGGTGGTAACGGCGCAGTGGCGGTTTTCAT
pIDS                -------------------------------------------AAGTAGCGTA--TGCGCTCACGCAACTGGTCCAGAACCTTGACCGAACGCAGCGGTGGTAACGGCGCAGTGGCGGTTTTCAT
pDONR223            ACCCAGTGGACATAAGCCT-GTTCGGTTCGTAAGCTGTAATGCAAGTAGCGTA--TGCGCTCACGCAACTGGTCCAGAACCTTGACCGAACGCAGCGGTGGTAACGGCGCAGTGGCGGTTTTCAT
pZP100              ACCCAGTGGACATAAGCCTCGTTCGGTTCGTAAGCTGTAATGCAAGTAGCGTAACTGCCGTCACGCAACTGGTCCAGAACCTTGACCGAACGCAGCGGTGGTAACGGCGCAGTGGCGGTTTTCAT
pZP101              ACCCAGTGGACATAAGCCTCGTTCGGTTCGTAAGCTGTAATGCAAGTAGCGTAACTGCCGTCACGCAACTGGTCCAGAACCTTGACCGAACGCAGCGGTGGTAACGGCGCAGTGGCGGTTTTCAT
pZP222              ACCCAGTGGACATAAGCCTCGTTCGGTTCGTAAGCTGTAATGCAAGTAGCGTAACTGCCGTCACGCAACTGGTCCAGAACCTTGACCGAACGCAGCGGTGGTAACGGCGCAGTGGCGGTTTTCAT
pZP221              ACCCAGTGGACATAAGCCTCGTTCGGTTCGTAAGCTGTAATGCAAGTAGCGTAACTGCCGTCACGCAACTGGTCCAGAACCTTGACCGAACGCAGCGGTGGTAACGGCGCAGTGGCGGTTTTCAT
pZP212              ACCCAGTGGACATAAGCCTCGTTCGGTTCGTAAGCTGTAATGCAAGTAGCGTAACTGCCGTCACGCAACTGGTCCAGAACCTTGACCGAACGCAGCGGTGGTAACGGCGCAGTGGCGGTTTTCAT
pZP211              ACCCAGTGGACATAAGCCTCGTTCGGTTCGTAAGCTGTAATGCAAGTAGCGTAACTGCCGTCACGCAACTGGTCCAGAACCTTGACCGAACGCAGCGGTGGTAACGGCGCAGTGGCGGTTTTCAT
pZP202              ACCCAGTGGACATAAGCCTCGTTCGGTTCGTAAGCTGTAATGCAAGTAGCGTAACTGCCGTCACGCAACTGGTCCAGAACCTTGACCGAACGCAGCGGTGGTAACGGCGCAGTGGCGGTTTTCAT
pZP201              ACCCAGTGGACATAAGCCTCGTTCGGTTCGTAAGCTGTAATGCAAGTAGCGTAACTGCCGTCACGCAACTGGTCCAGAACCTTGACCGAACGCAGCGGTGGTAACGGCGCAGTGGCGGTTTTCAT
pZP200              ACCCAGTGGACATAAGCCTCGTTCGGTTCGTAAGCTGTAATGCAAGTAGCGTAACTGCCGTCACGCAACTGGTCCAGAACCTTGACCGAACGCAGCGGTGGTAACGGCGCAGTGGCGGTTTTCAT
pCAMBIA1200         ACCCAGTGGACATAAGCCTCGTTCGGTTCGTAAGCTGTAATGCAAGTAGCGTAACTGCCGTCACGCAACTGGTCCAGAACCTTGACCGAACGCAGCGGTGGTAACGGCGCAGTGGCGGTTTTCAT
pCAMBIA1105.1R      ACCCAGTGGACATAAGCCTCGTTCGGTTCGTAAGCTGTAATGCAAGTAGCGTAACTGCCGTCACGCAACTGGTCCAGAACCTTGACCGAACGCAGCGGTGGTAACGGCGCAGTGGCGGTTTTCAT
pCAMBIA1105.1       ACCCAGTGGACATAAGCCTCGTTCGGTTCGTAAGCTGTAATGCAAGTAGCGTAACTGCCGTCACGCAACTGGTCCAGAACCTTGACCGAACGCAGCGGTGGTAACGGCGCAGTGGCGGTTTTCAT
pCAMBIA0105.1R      ACCCAGTGGACATAAGCCTCGTTCGGTTCGTAAGCTGTAATGCAAGTAGCGTAACTGCCGTCACGCAACTGGTCCAGAACCTTGACCGAACGCAGCGGTGGTAACGGCGCAGTGGCGGTTTTCAT
pCAMBIA5105         ACCCAGTGGACATAAGCCTCGTTCGGTTCGTAAGCTGTAATGCAAGTAGCGTAACTGCCGTCACGCAACTGGTCCAGAACCTTGACCGAACGCAGCGGTGGTAACGGCGCAGTGGCGGTTTTCAT
                                                               **********  ***  *****************************************************************
                        -35     Pc promoter    -10
```

IntI1

```
pDEST20-Pc_prom     GGC---TTGTTATGAC-TGTTTTTTTG---TACAGTCTATGCCTCGGGCATCCAAGCAGCAAGCGCGTTACGCCGTGGGTCGATGTTTGATGTTATGGAGCAGCAACG--------------------------------------------
pIDS                GGC---TTGTTATGAC-TGTTTTTTTGGGGTACAGTCTATGCCTCGGGCATCCAAGCAGCAAGCGCGTTACGCCGTGGGTCGATGTTTGATGTTATGGAGCAGCAACGATGTTACGCAGCAGGGCAGTCGCCCTAAAACAAAGTTAAACATC
pDONR223            GGC---TTGTTATGAC-TGTTTTTTTGGGGTACAGTCTATGCCTCGGGCATCCAAGCAGCAAGCGCGTTACGCCGTGGGTCGATGTTTGATGTTATGGAGCAGCAACGATGTTACGCAGCAGGGCAGTCGCCCTAAAACAAAGTTAAACATC
pZP100              GGCTTCTTGTTATGACATGTTTTTTTGGGGTACAGTCTATGCCTCGGGCATCCAAGCAGCAAGCGCGTTACGCCGTGGGTCGATGTTTGATGTTATGGAGCAGCAACGATGTTACGCAGCAGGGCAGTCGCCCTAAAACAAAGTTAAACATC
pZP101              GGCTTCTTGTTATGACATGTTTTTTTGGGGTACAGTCTATGCCTCGGGCATCCAAGCAGCAAGCGCGTTACGCCGTGGGTCGATGTTTGATGTTATGGAGCAGCAACGATGTTACGCAGCAGGGCAGTCGCCCTAAAACAAAGTTAAACATC
pZP222              GGCTTCTTGTTATGACATGTTTTTTTGGGGTACAGTCTATGCCTCGGGCATCCAAGCAGCAAGCGCGTTACGCCGTGGGTCGATGTTTGATGTTATGGAGCAGCAACGATGTTACGCAGCAGGGCAGTCGCCCTAAAACAAAGTTAAACATC
pZP221              GGCTTCTTGTTATGACATGTTTTTTTGGGGTACAGTCTATGCCTCGGGCATCCAAGCAGCAAGCGCGTTACGCCGTGGGTCGATGTTTGATGTTATGGAGCAGCAACGATGTTACGCAGCAGGGCAGTCGCCCTAAAACAAAGTTAAACATC
pZP212              GGCTTCTTGTTATGACATGTTTTTTTGGGGTACAGTCTATGCCTCGGGCATCCAAGCAGCAAGCGCGTTACGCCGTGGGTCGATGTTTGATGTTATGGAGCAGCAACGATGTTACGCAGCAGGGCAGTCGCCCTAAAACAAAGTTAAACATC
pZP211              GGCTTCTTGTTATGACATGTTTTTTTGGGGTACAGTCTATGCCTCGGGCATCCAAGCAGCAAGCGCGTTACGCCGTGGGTCGATGTTTGATGTTATGGAGCAGCAACGATGTTACGCAGCAGGGCAGTCGCCCTAAAACAAAGTTAAACATC
pZP202              GGCTTCTTGTTATGACATGTTTTTTTGGGGTACAGTCTATGCCTCGGGCATCCAAGCAGCAAGCGCGTTACGCCGTGGGTCGATGTTTGATGTTATGGAGCAGCAACGATGTTACGCAGCAGGGCAGTCGCCCTAAAACAAAGTTAAACATC
pZP201              GGCTTCTTGTTATGACATGTTTTTTTGGGGTACAGTCTATGCCTCGGGCATCCAAGCAGCAAGCGCGTTACGCCGTGGGTCGATGTTTGATGTTATGGAGCAGCAACGATGTTACGCAGCAGGGCAGTCGCCCTAAAACAAAGTTAAACATC
pZP200              GGCTTCTTGTTATGACATGTTTTTTTGGGGTACAGTCTATGCCTCGGGCATCCAAGCAGCAAGCGCGTTACGCCGTGGGTCGATGTTTGATGTTATGGAGCAGCAACGATGTTACGCAGCAGGGCAGTCGCCCTAAAACAAAGTTAAACATC
pCAMBIA1200         GGCTTCTTGTTATGACATGTTTTTTTGGGGTACAGTCTATGCCTCGGGCATCCAAGCAGCAAGCGCGTTACGCCGTGGGTCGATGTTTGATGTTATGGAGCAGCAACGATGTTACGCAGCAGGGCAGTCGCCCTAAAACAAAGTTAAACATC
pCAMBIA1105.1R      GGCTTCTTGTTATGACATGTTTTTTTGGGGTACAGTCTATGCCTCGGGCATCCAAGCAGCAAGCGCGTTACGCCGTGGGTCGATGTTTGATGTTATGGAGCAGCAACGATGTTACGCAGCAGGGCAGTCGCCCTAAAACAAAGTTAAACATC
pCAMBIA1105.1       GGCTTCTTGTTATGACATGTTTTTTTGGGGTACAGTCTATGCCTCGGGCATCCAAGCAGCAAGCGCGTTACGCCGTGGGTCGATGTTTGATGTTATGGAGCAGCAACGATGTTACGCAGCAGGGCAGTCGCCCTAAAACAAAGTTAAACATC
pCAMBIA0105.1R      GGCTTCTTGTTATGACATGTTTTTTTGGGGTACAGTCTATGCCTCGGGCATCCAAGCAGCAAGCGCGTTACGCCGTGGGTCGATGTTTGATGTTATGGAGCAGCAACGATGTTACGCAGCAGGGCAGTCGCCCTAAAACAAAGTTAAACATC
pCAMBIA5105         GGCTTCTTGTTATGACATGTTTTTTTGGGGTACAGTCTATGCCTCGGGCATCCAAGCAGCAAGCGCGTTACGCCGTGGGTCGATGTTTGATGTTATGGAGCAGCAACGATGTTACGCAGCAGGGCAGTCGCCCTAAAACAAAGTTAAACATC
                    ***   ********** **********   ******************************************************************************
                       -35     P2 promoter     -10
```

inactivated *att1* site

The native SmR promoter for these plasmids is derived from *E. coli* plasmid R538 and drives expression of the spectinomycin and streptomycin resistance gene (SmR) *aadA* (aminoglycoside 3″-adenylyltransferase), which is a class 1 integron and includes two promoters to drive expression of gene cassettes that integrate at the *att1* site (mutated in these constructs) of the plasmid. The Pc promoter is embedded in the 5′ end of a gene, *IntI1*, that encodes the integrase responsible for recombination between the *attC* containing integron and the *att1* site behind the promoter. *IntI1* expression is driven by a promoter, Pint (not shown), that partially overlaps the P2 promoter, but is inverted. With the exception of pIDS, which is missing Pc, these plasmids all have a truncated *IntI1*.

Promoter strength analysis based on Jove et al. (2010) PloS Genet. 6:e1000793. The Pc promoter of pDEST20 and derivatives is a strong version of the promoter called PcS. It has optimal -35 to -10 spacing and two bp changes relative to the other Pc promoters in the alignment, which are weak (PcW). However, the P2 promoter of pDEST20 is probably inactive as it has only 14 bp between the -35 and -10 hexamers.

SmR expression from pIDS is driven only by P2 which has the optimal spacing (17 bp) between the -35 and -10 hexamers.

Both the PcW and P2 promoters of pDONR223 have optimal spacing.

The pZP and pCAMBIA series have sub-optimal spacing (18 bp) in both the PcS and P2 promoters.

In pDEST20 and derivatives, the Pc promoter is used to express GmR (gentamycin resistance; aacC1, gentamycin acetyltransferase).
In pZP1XX series, Pc drives expression of CmR.

Sequence Alignments

Un-annotated SmR promoters

**Name of Plasmid Or Feature**	**Alignment**
pHELLSGATE-sat-aadA1	CAATTTGTACGCAAAATGTGGCTTTACTCTCGGCGGCATTGACCTGTTCACG.CAATTCATTCAAGCCGACACCGCTTCGCGGCGCGGCTTAATTCAGGAGTTAAACATC
pHELLSGATE4-sat-aadA1	CAATTTGTACGCAAAATGTGGCTTTACTCTCGGCGGCATTGACCTGTTCACG.CAATTCATTCAAGCCGACACCGCTTCGCGGCGCGGCTTAATTCAGGAGTTAAACATC
pHELLSGATE8-sat-aadA1	CAATTTGTACGCAAAATGTGGCTTTACTCTCGGCGGCATTGACCTGTTCACG.CAATTCATTCAAGCCGACACCGCTTCGCGGCGCGGCTTAATTCAGGAGTTAAACATC
pHELLSGATE12-sat-aadA1	CAATTTGTACGCAAAATGTGGCTTTACTCTCGGCGGCATTGACCTGTTCACG.CAATTCATTCAAGCCGACACCGCTTCGCGGCGCGGCTTAATTCAGGAGTTAAACATC
pSB11-sat-aadA1	CAATTTGTACGCAAAATGTGGCTTTACTCTCGGCGGCATTGACCTGTTCACG.CAATTCATTCAAGCCGACACCGCTTCGCGGCGCGGCTTAATTCAGGAGTTAAACATC
	** ***

**Name of Plasmid Or Feature**	**Alignment**
pHELLSGATE_5'sat1	AAGTGGCAGCAACGGATTCGCAAACCTGTCACGCCTTTTGTGCCAAAAGCCGCGCCAGGTTTGCGATCCGCTGTGCCAGGCGTTAGGCGTCAT
pHELLSGATE4_5'sat1	AAGTGGCAGCAACGGATTCGCAAACCTGTCACGCCTTTTGTGCCAAAAGCCGCGCCAGGTTTGCGATCCGCTGTGCCAGGCGTTAGGCGTCAT
pHELLSGATE8_5'sat1	AAGTGGCAGCAACGGATTCGCAAACCTGTCACGCCTTTTGTGCCAAAAGCCGCGCCAGGTTTGCGATCCGCTGTGCCAGGCGTTAGGCGTCAT
pHELLSGATE12_5'sat1	AAGTGGCAGCAACGGATTCGCAAACCTGTCACGCCTTTTGTGCCAAAAGCCGCGCCAGGTTTGCGATCCGCTGTGCCAGGCGTTAGGCGTCAT
pSB11_5'sat1	AAGTGGCAGCAACGGATTCGCAAACCTGTCACGCCTTTTGTGCCAAAAGCCGCGCCAGGTTTGCGATCCGCTGTGCCAGGCGTTAGGCGTCAT
	***

SmR in these plasmids is derived from *E. coli* Tn7 which contains a class 2 integron including *dfrAI* (dihydrofolate reductase conferring resistance to trimethoprim), *sat* (streptothricin acetyltransferase), *aadA1* (SmR - spectinomycin and streptomycin resistance, aminoglycoside adenylyltransferase), and ybeA (pseudouridine methyltransferase).

Class 2 integrons have a similar structure to class 1, but the sequences of the Pc promoter (-35 TTTAAT 16 bp TAAAAT -10) and the integrase (*IntI2*) are different. The Pc promoter of Tn7 drives expression of a polycistronic transcript (*dfrA1-sat-aadA1-ybeA*). However, neither the pSB11 nor the pHELLSGATE plasmids include the Pc promoter from Tn7, which is upstream of *dfrA1*. These plasmids all include *sat*, *aadA1* and *ybeA*, but pSB11 includes just a portion of the end of *dfrA1*, while the pHELLSGATE plasmids start just 5' to *sat*. Therefore, it is not clear what sequence constitutes a promoter in these constructs.

In the alignments, we show the region covering the 3' end of sat and intergenic region between sat and SmR (top alignment), and the region upstream of *sat* common to pSB11 and pHELLSGATE (bottom alignment), which has no putative -35 and -10 site. It seems likely that there is a cryptic promoter within these regions or the *sat* ORF. BPROM predicts a putative promoter at the end of the *sat* ORF (top alignment; yellow highlight). This region is ~100 bp upstream of the *aadA1* START codon.

Key ATGC = change in nucleotide relative to consensus sequence – non-coding sequence

Sequence Alignments

Un-annotated ccdB promoters

```
Name of Plasmid     Alignment
Or Feature
ccdB_nat_prom       TAAAATACATAAGGCTTACTAAAAGCCAGATAACAGTATGCGTATTTGCGCGCTGATTTTTGCGGTATAAGAATATATACTGATATGTATACCCGAAGTATGTCAAAAAGAGGTGTGCTATGAAGCAGCGTATTACAGTGACAGTTGACA
pDEST26             AGATCTGGATCCGGCTTACTAAAAGCCAGATAACAGTATGCGTATTTGCGCGCTGATTTTTGCGGTATAAGAATATATACTGATATGTATACCCGAAGTATGTCAAAAAGAGGTGTGCTATGAAGCAGCGTATTACAGTGACAGTTGACA
pLenti4_V5-DEST     AGATCTGGATCCGGCTTACTAAAAGCCAGATAACAGTATGCGTATTTGCGCGCTGATTTTTGCGGTATAAGAATATATACTGATATGTATACCCGAAGTATGTCAAAAAGAGGTGTGCTATGAAGCAGCGTATTACAGTGACAGTTGACA
pT-Tex-DEST30       AGATCTGGATCCGGCTTACTAAAAGCCAGATAACAGTATGCGTATTTGCGCGCTGATTTTTGCGGTATAAGAATATATACTGATATGTATACCCGAAGTATGTCAAAAAGAGGTGTGCTATGAAGCAGCGTATTACAGTGACAGTTGACA
pcDNA-DEST40        AGATCTGGATCCGGCTTACTAAAAGCCAGATAACAGTATGCGTATTTGCGCGCTGATTTTTGCGGTATAAGAATATATACTGATATGTATACCCGAAGTATGTCAAAAAGAGGTGTGCTATGAAGCAGCGTATTACAGTGACAGTTGACA
pANT7-nHA           AGATCTGGATCCGGCTTACTAAAAGCCAGATAACAGTATGCGTATTTGCGCGCTGATTTTTGCGGTATAAGAATATATACTGATATGTATACCCGAAGTATGTCAAAAAGAGGTGTGCTATGAAGCAGCGTATTACAGTGACAGTTGACA
pDEST8              ACGCGTGGATCCGGCTTACTAAAAGCCAGATAACAGTATGCGTATTTGCGCGCTGATTTTTGCGGTATAAGAATATATACTGATATGTATACCCGAAGTATGTCAAAAAGAGGTGTGCTATGAAGCAGCGTATTACAGTGACAGTTGACA
pAd_BLOCK-iT-DEST   ACGCGTGGATCCGGCTTACTAAAAGCCAGATAACAGTATGCGTATTTGCGCGCTGATTTTTGCGGTATAAGAATATATACTGATATGTATACCCGAAGTATGTCAAAAAGAGGTGTGCTATGAAGCAGCGTATTACAGTGACAGTTGACA
pDONR201            TCGCGTGGATCCGGCTTACTAAAAGCCAGATAACAGTATGCGTATTTGCGCGCTGATTTTTGCGGTATAAGAATATATACTGATATGTATACCCGAAGTATGTCAAAAAGAGGTGTGCTATGAAGCAGCGTATTACAGTGACAGTTGACA
pAd_PL-DEST         ACGCGTGGATCCGGCTTACTAAAAACCAGATAACAGTATGCGTATTTGCGCGCTGATTTTTGCGGTATAAGAATATATACTGATATGTATACCCGAAGTATGTCAAAAAGAGGTGTGCTATGAAGCAGCGTATTACAGTGACAGTTGACA
pET-53-DEST         ACGCGTGGATCCGGCTTACTAAAAGCCAGATAACAGTATGCGTATTTGCGCGCTGATTTTTGCGGTATAAGAATATATACTGATATGTATACCCGAAGTATGTCAAAAAGAGGTATGCTATGAAGCAGCGTATTACAGTGACAGTTGACA
pIB_V5-His-DEST     ACGCGTGGATCCGGCTTACTAAAAGCCAGATAACAGTATGCGTATTTGCGCGCTGATTTTTGCGGTATAAGAATATATACTGATATGTATACCCGAAGTATGTCAAAAAGAGGTATGCTATGAAGCAGCGTATTACAGTGACAGTTGACA
pMT_BioEase-DEST    ACGCGTGGATCCGGCTTACTAAAAGCCAGATAACAGTATGCGTATTTGCGCGCTCGCGA---------------------ACCGGTGTATACCCGAAGTATGTCAAAAAGAGGTGTGCTATGAAGCAGCGTATTACAGTGACAGTTGACA
pCMV_SPORT6ccdB     GGCCGCCGATCCGGCTTACTAAAAGCCAGATAACAGTATGCGTATTTGCGCGCTGATTTTTGCGGTATAAGAATATATACTGATATGTATACCCGAAGTATGTCAAAAAGAGGTGTGCTTCTAGA-------------------------
pOSIP-CH            GATCCTCTAGAGGGCTTACTAAAAGCCAGATAACAGTATGCGTATTTGCGCGCTGATTTTTGCGGTATAAGAATATATACTGATATGTATACCCGAAGTATGTCAAAAAGAGGTATGCT-------------------------------
                            *   ************ *****************************                               ***************************** ****
                                                                          -35                  -10
                       MCS                                               NruI                    AgeI                                    XbaI      ccdA

ccdB_nat_prom       GCGACAGCTATCAGTTGCTCAAGGCATA--TGATGTCAATATCTCCGGTCTGGTAAGCACAACCATGCAGAATGAAGCCCGTCGTCTGCGTGCCGAACGC.AATGAACGGCTCTTTTGCTGACGAGAACAGGGACTGGTGAA
pDEST26             GCGACAGCTATCAGTTGCTCAAGGCATATATGATGTCAATATCTCCGGTCTGGTAAGCACAACCATGCAGAATGAAGCCCGTCGTCTGCGTGCCGAACGC.AATGAACGGCTCTTTTGCTGACGAGAACAGGGACTGGTGAA
pLenti4_V5-DEST     GCGACAGCTATCAGTTGCTCAAGGCATATATGATGTCAATATCTCCGGTCTGGTAAGCACAACCATGCAGAATGAAGCCCGTCGTCTGCGTGCCGAACGC.AATGAACGGCTCTTTTGCTGACGAGAACAGGGACTGGTGAA
pT-Tex-DEST30       GCGACAGCTATCAGTTGCTCAAGGCATATATGATGTCAATATCTCCGGTCTGGTAAGCACAACCATGCAGAATGAAGCCCGTCGTCTGCGTGCCGAACGC.AATGAACGGCTCTTTTGCTGACGAGAACAGGGACTGGTGAA
pcDNA-DEST40        GCGACAGCTATCAGTTGCTCAAGGCATATATGATGTCAATATCTCCGGTCTGGTAAGCACAACCATGCAGAATGAAGCCCGTCGTCTGCGTGCCGAACGC.AATGAACGGCTCTTTTGCTGACGAGAACAGGGACTGGTGAA
pANT7-nHA           GCGACAGCTATCAGTTGCTCAAGGCATATATGATGTCAATATCTCCGGTCTGGTAAGCACAACCATGCAGAATGAAGCCCGTCGTCTGCGTGCCGAACGC.AATGAACGGCTCTTTTGCTGACGAGAACAGGGACTGGTGAA
pDEST8              GCGACAGCTATCAGTTGCTCAAGGCATATATGATGTCAATATCTCCGGTCTGGTAAGCACAACCATGCAGAATGAAGCCCGTCGTCTGCGTGCCGAACGC.AATGAACGGCTCTTTTGCTGACGAGAACAGGGACTGGTGAA
pAd_BLOCK-iT-DEST   GCGACAGCTATCAGTTGCTCAAGGCATATATGATGTCAATATCTCCGGTCTGGTAAGCACAACCATGCAGAATGAAGCCCGTCGTCTGCGTGCCGAACGC.AATGAACGGCTCTTTTGCTGACGAGAACAGGGACTGGTGAA
pDONR201            GCGACAGCTATCAGTTGCTCAAGGCATATATGATGTCAATATCTCCGGTCTGGTAAGCACAACCATGCAGAATGAAGCCCGTCGTCTGCGTGCCGAACGC.AATGAACGGCTCTTTTGCTGACGAGAACAGGGACTGGTGAA
pAd_PL-DEST         GCGACAGCTATCAGTTGCTCAAGGCATATATGATGTCAATATCTCCGGTCTGGTAAGCACAACCATGCAGAATGAAGCCCGTCGTCTGCGTGCCGAACGC.AATGAACGGCTCTTTTGCTGACGAGAACAGGGGCTGGTGAA
pET-53-DEST         GCGACAGCTATCAGTTGCTCAAGGCATATATGATGTCAATATCTCCGGTCTGGTAAGCACAACCATGCAGAATGAAGCCCGTCGTCTGCGTGCCGAACGC.AATGAACGGCTCTTTTGCTGACGAGAACAGGGGCTGGTGAA
pIB_V5-His-DEST     GCGACAGCTATCAGTTGCTCAAGGCATATATGATGTCAATATCTCCGGTCTGGTAAGCACAACCATGCAGAATGAAGCCCGTCGTCTGCGTGCCGAACGC.AATGAACGGCTCTTTTGCTGACGAGAACAGGGGCTGGTGAA
pMT_BioEase-DEST    GCGACAGCTATCAGTTGCTCAAGGCATATATGATGTCAATATCTCCGGTCTGGTAAGCACAACCATGCAGAATGAAGCCCGTCGTCTGCGTGCCGAACGC.AATGAACGGCTCTTTTGCTGACGAGAACAGGGACTGGTGAA
pCMV_SPORT6ccdB     ---------------------------------------------------------------------------------------------------- -----------------------------------------
pOSIP-CH            ---------------------------------------------------------------------------------------------------- -----------------------------------------
                                    -35?               -10?
                                                                     ccdA
```

The native ccdB promoter (ccdB_nat_prom) from *E. coli* F plasmid is shown in the first row. *ccdB* is transcribed as part of a multi-cistronic transcript along with *ccdA* (arrow), an inhibitor of ccdB protein. *ccdA* is inactivated in *ccdB* plasmids by a two bp insertion, or by complete deletion of the ORF (pCMV_SPORT6ccdB and pOSIP-CH).

Restriction enzyme sites used for cloning (MCS in pOSIP-CH) or for construction of the plasmids are shown.

The -10 and -35 sequences are missing from pMT_BioEase-DEST, but a second potential -35 and -10 is present in the disrupted *ccdA* ORF (BPROM). Deletion of the natural promoter may allow more efficient transcription from this cryptic promoter, bypassing transcription of the entire *ccdA* ORF.

Sequence Alignments

Coding Sequences

Name of Variant	# of Occur	Alignment	Size (bp)	# Var bp	Sources*
AmpR-013	688	ATGAGTATTCAACATTTCCGTGTCGCCCTTATTCCCTTTTTTGCGGCATTTTGCCTTCCTGTTTTTGCTCACCCAGAAACGCTGGTGAAAGTAAAAGATGCTGAAGATCAGTTGGGTGCA	861	0	Most
AmpR-017	78	ATGAGTATTCAACATTTCCGTGTCGCCCTTATTCCCTTTTTTGCGGCATTTTGCCTTCCTGTTTTTGCTCACCCAGAAACGCTGGTGAAAGTAAAAGATGCTGAAGATCAGTTGGGTGCA	861	2	In,No,GE,Cl,Ne
AmpR-001	76+2	ATGAGTATTCAACATTTCCGTGTCGCCCTTATTCCCTTTTTTGCGGCATTTTGCCTTCCTGTTTTTGCTCACCCAGAAACGCTGGTGAAAGTAAAAGATGCTGAAGATCAGTTGGGTGCA	861	1	No,GS,MCSG
AmpR-021	69	ATGAGCATCCAACATTTTCGTGTCGCACTCATTCCCTTCTTTGCGGCATTTTGCTTGCCTGTTTTTGCACACCCCGAAACGCTGGTGAAAGTAAAAGATGCTGAAGATCAACTGGGTGCA	861	102	Pr,Lu
AmpR-016	45+1	ATGAGTATTCAACATTTCCGTGTCGCCCTTATTCCCTTTTTTGCGGCATTTTGCCTTCCTGTTTTTGCTCACCCAGAAACGCTGGTGAAAGTAAAAGATGCTGAAGATCAGTTGGGTGCA	861	1	Cl,Or,In
AmpR-022	28	ATGAGTATTCAACATTTCCGTGTCGCCCTTATTCCCTTTTTTGCGGCATTTTGCCTTCCTGTTTTTGCTCACCCAGAAACGCTGGTGAAAGTAAAAGATGCTGAAGATCAGTTGGGTGCA	861	1	Ev,Qi
AmpR-010	16	ATGAGTATTCAACATTTCCGTGTCGCCCTTATTCCCTTTTTTGCGGCATTTTGCCTTCCTGTTTTTGCTCACCCAGAAACGCTGGTGAAAGTAAAAGATGCTGAAGATCAGTTGGGTGCA	861	4	Th,SG
AmpR-004	9+3	ATGAGTATTCAACATTTCCGTGTCGCCCTTATTCCCTTTTTTGCGGCATTTTGCCTTCCTGTTTTTGCTCACCCAGAAACGCTGGTGAAAGTAAAAGATGCTGAAGATCAGTTGGGTGCA	861	8	In
AmpR-019	8	ATGAGTATTCAACATTTCCGTGTCGCCCTTATTCCCTTTTTTGCGGCATTTTGCCTTCCTGTTTTTGCTCACCCAGAAACGCTGGTGAAAGTAAAAGATGCTGAAGATCAGTTGGGTGCA	861	2	2659436
AmpR-020	8	ATGAGTATTCAACATTTCCGTGTCGCCCTTATTCCCTTTTTTGCGGCATTTTGCCTTCCTGTTTTTGCTCACCCAGAAACGCTGGTGAAAGTAAAAGATGCTGAAGATCAGTTGGGTGCA	873	0/12i	Cl
AmpR-014	6	ATGAGTATTCAACATTTCCGTGTCGCCCTTATTCCCTTTTTTGCGGCATTTTGCCTTCCTGTTTTTGCTCACCCAGAAACGCTGGTGAAAGTAAAAGATGCTGAAGATCAGTTGGGTGCA	861	2	7669286
**AmpR-007**	**5**	**ATGAGTATTCAACATTTCCGTGTCGCCCTTATTCCCTTTTTTGCGGCATTTTGCCTTCCTGTTTTTGCTCACCCAGAAACGCTGGTGAAAGTAAAAGATGCTGAAGATCAGTTGGGTGCA**	**858**	**0/3d**	**Ag**
AmpR-015	5	ATGAGTATTCAACATTTCCGTGTCGCCCTTATTCCCTTTTTTGCGGCATTTTGCCTTCCTGTTTTTGCTCACCCAGAAACGCTGGTGAAAGTAAAAGATGCTGAAGATCAGTTGGGTGCA	861	5	Lu
AmpR-011	4	ATGAGTATTCAACATTTCCGTGTCGCCCTTATTCCCTTTTTTGCGGCATTTTGCCTTCCTGTTTTTGCTCACCCAGAAACGCTGGTGAAAGTAAAAGATGCTGAAGATCAGTTGGGTGCA	861	2	Pr
**AmpR-002**		**ATGAGTATTCAACATTTCCGTGTCGCCCTTATTCCCTTTTTTGCGGCATTTTGCCTTCCTGTTTTTGCTCACCCAGAAACGCTGGTGAAAGTAAAAGATGCTGAAGATCAGTTGGGTGCA**	**861**	**9**	**In**
AmpR-018	3	ATGAGTATTCAACATTTCCGTGTCGCCCTTATTCCCTTTTTTGCGGCATTTTGCCTTCCTGTTTTTGCTCACCCAGAAACGCTGGTGAAAGTAAAAGATGCTGAAGATCAGTTGGGTGCG	861	7	Ox
AmpR-003	2	ATGAGTATTCAACATTTCCGTGTCGCCCTTATTCCCTTTTTTGCGGCATTTTGCCTTCCTGTTTTTGCTCACCCAGAAACGCTGGTGAAAGTAAAAGATGCTGAAGATCAGTTGGGTGCA	861	1	15782178
AmpR-008	2+1	ATGAGTATTCAACATTTCCGTGTCGCCCTTATTCCCTTTTTTGCGGCATTTTGCCTTCCTGTTTTTGCTCACCCAGAAACGCTGGTGAAAGTAAAAGATGCTGAAGATCAGTTGGGTGCA	861	1	No,Or
AmpR-005	1	ATGAGTATTCAACATTTCCGTGTCGCCCTTATTCCCTTTTTTGCGGCATTTTGCCTTCCTGTTTTTGCTCACCCAGAAACGCTGGTGAAAGTAAAAGATGCTGAAGATCAGTTGGGTGCA	861	2	In
AmpR-006	1	ATGAGTATTCAACATTTCCGTGTCGCCCTTATTCCCTTTTTTGCGGCATTTTGCCTTCCTGTTTTTGCTCACCCAGAAACGCTGGTGAAAGTAAAAGATGCTGAAGATCAGTTGGGTGCA	861	3	IMAGE
**AmpR-009**		**---AGTATTCAACATTTCCGTGTCGCCCTTATTCCCTTTTTTGCGGCATTTTGCCTTCCTGTTTTTGCTCACCCAGAAACGCTGGTGAAAGTAAAAGATGCTGAAGATCAGTTGGGTGCA**	**861**	**1/3d3i**	**8242750**
AmpR-012	1	ATGAGTATTCAACATTTCCGTGTCGCCCTTATTCCCTTTTTTGCGGCATTTTGCCTTCCTGTTTTTGCTCACCCAGAAACGCTGGTGAAAGTAAAAGATGCTGAAGATCAGTTGGGTGCA	861	1	In
AmpR-023	1	ATGAGTATTCAACATTTCCGTGTCGCCCTTATTCCCTTTTTTGCGGCATTTTGCCTTCCTGTTTTTGCTCACCCAGAAACGCTGGTGAAAGTAAAAGATGCTGAAGATCAGTTGGGTGCA	861	4	MCSG
**bla(M)-001**		**--CCAGAAACGCTGGTGAAAGTAAAAGATGCTGAAGATCAGTTGGGTGCA**	**789**	**1/6d**	**12071693**
bla(M)-002	1	--ATGGACCCAGAAACGCTGGTGAAAGTAAAAGATGCTGAAGATCAGTTGGGTGCA	795	0	In
**bla(M)-003**		**--CCAGAAACGCTGGTGAAAGTAAAAGATGCTGAAGATCAGTTGGGTGCA**	**789**	**1/6d**	**No**
**bla(M)-004**	**1**	**--ATGGACCCAGAAACGCTGGTGAAAGTAAAAGATGCTGAAGATCAGTTGGGTGCA**	**792**	**0/3d**	**In**
AmpR-007corr		ATGAGTATTCAACATTTCCGTGTCGCCCTTATTCCCTTTTTTGCGGCATTTTGCCTTCCTGTTTTTGCTCACCCAGAAACGCTGGTGAAAGTAAAAGATGCTGAAGATCAGTTGGGTGCA	870	0	
AmpR-009corr		ATGAGTATTCAACATTTCCGTGTCGCCCTTATTCCCTTTTTTGCGGCATTTTGCCTTCCTGTTTTTGCTCACCCAGAAACGCTGGTGAAAGTAAAAGATGCTGAAGATCAGTTGGGTGCA	861	1	
bla(M)-001corr		ATGAGTATTCAACATTTCCGTGTCGCCCTTATTCCCTTTTTTGCGGCATTTTGCCTTCCTGTTTTTGCTCACCCAGAAACGCTGGTGAAAGTAAAAGATGCTGAAGATCAGTTGGGTGCA	861	1	
bla(M)-003corr		ATGAGTATTCAACATTTCCGTGTCGCCCTTATTCCCTTTTTTGCGGCATTTTGCCTTCCTGTTTTTGCTCACCCAGAAACGCTGGTGAAAGTAAAAGATGCTGAAGATCAGTTGGGTGCA	861	1	
bla(M)-004corr		--ATGGACCCAGAAACGCTGGTGAAAGTAAAAGATGCTGAAGATCAGTTGGGTGCA	837	0	
		** ** ******** ******** ** ******** *************** * ********* **** *********************************** *******			
AmpR-013		CGAGTGGGTTACATCGAACTGGATCTCAACAGCGGTAAGATCCTTGAGAGTTTTCGCCCCGAAGAACGTTTTC------------CAATGATGAGCACTTTTAAAGTTCTGCTATGTGGC			
AmpR-017		CGAGTGGGTTACATCGAACTGGATCTCAACAGCGGTAAGATCCTTGAGAGTTTTCGCCCCGAAGAACGTTTTC------------CAATGATGAGCACTTTTAAAGTTCTGCTATGTGGC			
AmpR-001		CGAGTGGGTTACATCGAACTGGATCTCAACAGCGGTAAGATCCTTGAGAGTTTTCGCCCCGAAGAACGTTTTC------------CAATGATGAGCACTTTTAAAGTTCTGCTATGTGGC			
AmpR-021		AGAGTGGGCTATATCGAACTGGATCTCAATAGCGGCAAGATCCTTGAGTCTTTCCGCCCCGAAGAACGATTCC------------CGATGATGAGCACTTTCAAAGTACTGCTATGTGGC			
AmpR-016		CGAGTGGGTTACATCGAACTGGATCTCAACAGCGGTAAGATCCTTGAGAGTTTTCGCCCCGAAGAACGTTTTC------------CAATGATGAGCACTTTTAAAGTTCTGCTATGTGGC			
AmpR-022		CGAGTGGGTTACATCGAACTGGATCTCAACAGCGGTAAGATCCTTGAGAGTTTTCGCCCCGAAGAACGTTTTC------------CAATGATGAGCACTTTTAAAGTTCTGCTATGTGGC			
AmpR-010		CGAGTGGGTTACATCGAACTGGATCTCAACAGCGGTAAGATCCTTGAGAGTTTTCGCCCCGAAGAACGTTTTC------------CAATGATGAGCACTTTTAAAGTTCTGCTATGTGGC			
AmpR-004		CGAGTGGGTTACATCGAACTGGATCTCAACAGCGGTAAGATCCTTGAGAGTTTTCGCCCCGAAGAACGTTTTC------------CAATGATGAGCACTTTTAAAGTTCTGCTATGTGAT			
AmpR-019		CGAGTGGGTTACATCGAACTGGATCTCAACAGCGGTAAGATCCTTGAGAGTTTTCGCCCCGAAGAACGTTTTC------------CAATGATGAGCACTTTTAAAGTTCTGCTATGTGGC			
AmpR-020		CGAGTGGGTTACATCGAACTGGATCTCAACAGCGGTAAGATCCTTGAGAGTTTTCGCCCCGAAGAACGTTTTCAGGTGGAGGTGGCAATGATGAGCACTTTTAAAGTTCTGCTATGTGGC			
AmpR-014		CGAGTGGGTTACATCGAACTGGATCTCAACAGCGGTAAGATCCTTGAGAGTTTTCGCCCCGAAGAACGTTCTC------------CAATGATGAGCACTTTTAAAGTTCTGCTATGTGGC			
**AmpR-007**		**CGAGTGGGTTACATCGAACTGGATCTCAACAGCGGTAAGATCCTTGAGAGTTTTCGCCCCGAAGAACGTTTTC------------CAATGATGAGCACTTTTAAAGTTCTGCTATGTGGC**			
AmpR-015		CGAGTGGGTTACATCGAACTGGATCTCAACAGCGGTAAGATCCTTGAGAGTTTACGCCCCGAAGAACGTTTTC------------CAATGATGAGCACTTTTAAAGTTCTGCTATGTGGC			
AmpR-011		CGAGTGGGTTACATCGAACTGGATCTCAACAGCGGTAAGATCCTTGAGAGTTTTCGCCCCGAAGAACGTTTTC------------CAATGATGAGCACTTTCAAAGTTCTGCTATGTGGC			
**AmpR-002**		**CGAGTGGGTTACATCGAACTGGATCTCAACAGCGGTAAGATCCTTGAGAGTTTTCGCCCCGAAGAACGTTTTC------------CAATGATGAGCACTTTTAAAGTTCTGCTATGTGAT**			
AmpR-018		CGAGTGGGTTACATCGAACTGGATCTCAACAGCGGTAAGATCCTTGAGAGTTTTCGCCCCGAAGAACGCTTTC------------CAATGATGAGCACTTTTAAAGTTCTGCTATGTGGC			
AmpR-003		CGAGTGGGTTACATCGAACTGGATCTCAACAGCGGTAAGATCCTTGAGAGTTTTCGCCCCGAAGAACGTTTTC------------CAATGATGAGCACTTTTAAAGTTCTGCTATGTGGC			
AmpR-008		CGAGTGGGTTACATCGAACTGGATCTCAACAGCGGTAAGATCCTTGAGAGTTTTCGCCCCGAAGAACGTTTTC------------CAATGATGAGCACTTTTAAAGTTCTGCTATGTGGC			
AmpR-005		CGAGTGGGTTACATCGAACTGGATCTCAACAGCGGTAAGATCCTTGAGAGTTTTCGCCCCGAAGAACGTTTTC------------CAATGATGAGCACTTTTAAAGTTCTGCTATGTGGC			
AmpR-006		CGAGTGGGTTACATCGAACTGGATCTCAACAGCGGTAAGATCCTTGAGAGTTTTCGCCCCGAAGAACGTTTTC------------CAATGATGAGCACTTTTAAAGTTCTGCTATGTGGC			
**AmpR-009**		**CGAGTGGGTTACATCGAACTGGATCTCAACAGCGGTAAGATCCTTGAGAGTTTTCGCCCCGAAGAACGTTTTC------------CAATGATGAGCACTTTTAAAGTTCTGCTATGTGGC**			
AmpR-012		CGAGTGGGTTACATCGAACTGGATCTCAACAGCGGTAAGATCCTTGAGAGTTTTCGCCCCGAAGAACGTTTTC------------CAATGATGAGCACTTTTAAAGTTCTGCTATGTGGC			
AmpR-023		CGAGTGGGTTACATCGAACTGGATCTCAACAGCGGTAAGATCCTTGAGAGTTTTCGCCCCGAAGAACGTTTTC------------CAATGATGAGCACTTTTAAAGTTCTGCTATGTGGC			
**bla(M)-001**		**CGAGTGGGTTACATCGAACTGGATCTCAACAGCGGTAAGATCCTTGAGAGTTTTCGCCCCGAAGAACGTTTTC------------CAATGATGAGCACTTTTAAAGTTCTGCTATGTGGC**			
bla(M)-002		CGAGTGGGTTACATCGAACTGGATCTCAACAGCGGTAAGATCCTTGAGAGTTTTCGCCCCGAAGAACGTTTTC------------CAATGATGAGCACTTTTAAAGTTCTGCTATGTGGC			
**bla(M)-003**		**CGAGTGGGTTACATCGAACTGGATCTCAACAGCGGTAAGATCCTTGAGAGTTTTCGCCCCGAAGAACGTTTTC------------CAATGATGAGCACTTTTAAAGTTCTGCTATGTGGC**			
**bla(M)-004**		**CGAGTGGGTTACATCGAACTGGATCTCAACAGCGGTAAGATCCTTGAGAGTTTTCGCCCCGAAGAACGTTTTC------------CAATGATGAGCACTTTTAAAGTTCTGCTATGTGGC**			
AmpR-007corr		CGAGTGGGTTACATCGAACTGGATCTCAACAGCGGTAAGATCCTTGAGAGTTTTCGCCCCGAAGAACGTTTTC------------CAATGATGAGCACTTTTAAAGTTCTGCTATGTGGC			
AmpR-009corr		CGAGTGGGTTACATCGAACTGGATCTCAACAGCGGTAAGATCCTTGAGAGTTTTCGCCCCGAAGAACGTTTTC------------CAATGATGAGCACTTTTAAAGTTCTGCTATGTGGC			
bla(M)-001corr		CGAGTGGGTTACATCGAACTGGATCTCAACAGCGGTAAGATCCTTGAGAGTTTTCGCCCCGAAGAACGTTTTC------------CAATGATGAGCACTTTTAAAGTTCTGCTATGTGGC			
bla(M)-003corr		CGAGTGGGTTACATCGAACTGGATCTCAACAGCGGTAAGATCCTTGAGAGTTTTCGCCCCGAAGAACGTTTTC------------CAATGATGAGCACTTTTAAAGTTCTGCTATGTGGC			
bla(M)-004corr		CGAGTGGGTTACATCGAACTGGATCTCAACAGCGGTAAGATCCTTGAGAGTTTTCGCCCCGAAGAACGTTTTC------------CAATGATGAGCACTTTTAAAGTTCTGCTATGTGGC			
		******* ** ***************** ***** ************ *** ************** * * * ************** ***** **********			

Sequence Alignments

AmpR-013	GCGGTATTATCCCGTATTGACGCCGGGCAAGAGCAACTCGGTCGCCGCATACACTATTCTCAGAATGACTTGGTTGAGTACTCACCAGTCACAGAAAAGCATCTTACGGATGGCATGACA
AmpR-017	GCGGTATTATCCCGTGTTGACGCCGGGCAAGAGCAACTCGGTCGCCGCATACACTATTCTCAGAATGACTTGGTTGAGTACTCACCAGTCACAGAAAAGCATCTTACGGATGGCATGACA
AmpR-001	GCGGTATTATCCCGTATTGACGCCGGGCAAGAGCAACTCGGTCGCCGCATACACTATTCTCAGAATGACTTGGTTGAGTACTCACCAGTCACAGAAAAGCATCTTACGGATGGCATGACA
AmpR-021	GCGGTGTTGTCCCGTATAGACGCCGGGCAAGAGCAGCTTGGTCGCCGTATACACTACTCACAAAACGACTTGGTTGAGTACTCGCCGGTCACGGAAAAGCATCTTACGGATGGCATGACG
AmpR-016	GCGGTATTATCCCGTGTTGACGCCGGGCAAGAGCAACTCGGTCGCCGCATACACTATTCTCAGAATGACTTGGTTGAGTACTCACCAGTCACAGAAAAGCATCTTACGGATGGCATGACA
AmpR-022	GCGGTATTATCCCGTATTGACGCCGGGCAAGAGCAACTCGGTCGCCGCATACACTATTCTCAGAATGACTTGGTTGAGTACTCACCAGTCACAGAAAAGCATCTTACGGATGGCATGACA
AmpR-010	GCGGTATTATCCCGTATTGACGCCGGGCAAGAGCAACTCGGTCGCCGCATACACTATTCTCAGAATGACTTGGTTGAGTACTCACCAGTCACAGAAAAGCATCTTACGGATGGCATGACA
AmpR-004	ACACTATTATCCCGTATTGACGCCGGGCAAGAGCAACTCGGTCGCCGCATACACTATTCTCAGAATGACTTGGTTGAGTACTCACCAGTCACAGAAAAGCATCTTACGGATGGCATGACA
AmpR-019	GCGGTATTATCCCGTATTGACGCCGGGCAAGAGCAACTCGGTCGCCGCATACACTATTCTCAGAATGACTTGGTTGAGTACTCACCAGTCACAGAAAAGCATCTTACGGATGGCATGACA
AmpR-020	GCGGTATTATCCCGTATTGACGCCGGGCAAGAGCAACTCGGTCGCCGCATACACTATTCTCAGAATGACTTGGTTGAGTACTCACCAGTCACAGAAAAGCATCTTACGGATGGCATGACA
AmpR-014	GCGGTATTATCCCGTGTTGACGCCGGGCAAGAGCAACTCGGTCGCCGCATACACTATTCTCAGAATGACTTGGTTGAGTACTCACCAGTCACAGAAAAGCATCTTACGGATGGCATGACA
**AmpR-007**	**GCGGTATTATCCCGTATTGACGCCGGGCAAGAGCAACTCGGTCGCCGCATACACTATTCTCAGAATGACTTGGTTGAGTACTCACCAGTCACAGAAAAGCATCTTACGGATGGCATGACA**
AmpR-015	GCGGTATTATCCCGTATTGACGCCGGGCAAGAGCAACTCGGTCGCCGCATACACTATTCTCAGAATGACTTGGTTGAGTACTCACCAGTCACAGAAAAGCATCTCACGGATGGCATGACA
AmpR-011	GCGGTATTATCCCGTATTGACGCCGGGCAAGAGCAACTCGGTCGCCGCATACACTATTCTCAGAATGACTTGGTTGAGTACTCACCAGTCACAGAAAAGCATCTTACGGATGGCATGACA
**AmpR-002**	**ACACTATTATCCCGTATTGACGCCGGGCAAGAGCAACTCGGTCGCCGCATACACTATTCTCAGAATGACTTGGTTGAGTACTCACCAGTCACAGAAAAGCATCTTACGGATGGCATGACA**
AmpR-018	GCGGTATTATCCCGTATTGACGCCGGGCAAGAGCAACTCGGTCGCCGCATACACTATTCTCAGAATGACTTGGTTGAGTATTCACCAGTCACAGAAAAGCATCTTACGGATGGCATGACA
AmpR-003	GCGGTATTATCCCGTATTGACGCCGGGCAAGAGCAACTCGGTCGCCGCATACGCTATTCTCAGAATGACTTGGTTGAGTACTCACCAGTCACAGAAAAGCATCTTACGGATGGCATGACA
AmpR-008	GCGGTATTATCCCGTATTGACGCCGGGCAAGAGCAACTCGGTCGCCGCATACACTATTCTCAGAATGACTTGGTTGAGTACTCACCAGTCACAGAAAAGCATCTTACGGATGGCATGACA
AmpR-005	GCGGTATTATCCCGTGTTGACGCCGGGCAAGAGCAACTCGGTCGCCGCATACACTATTCTCAGAATGACTTGGTTGAGTACTCACCAGTCACAGAAAAGCATCTTACGGATGGCATGACA
AmpR-006	GCGGTATTATCCCGTGATGACGCCGGGCAAGAGCAACTCGGTCGCCGCATACACTATTCTCAGAATGACTTGGTTGAGTACTCACCAGTCACAGAAAAGCATCTTACGGATGGCATGACA
**AmpR-009**	**GCGGTATTATCCCGTGTTGACGCCGGGCAAGAGCAACTCGGTCGCCGCATACACTATTCTCAGAATGACTTGGTTGAGTACTCACCAGTCACAGAAAAGCATCTTACGGATGGCATGACA**
AmpR-012	GCGGTATTATCCCGTATTGACGCCGGGCAAGAGCAACTCGGTCGCCGCATACACTATTCTCAGAATGACTTGGTTGAGTACTCACCAGTCACAGAAAAGCATCTTACGGATGGCATGACA
AmpR-023	GCGGTATTATCCCGTATTGACGCCGGGCAAGAGCAACTCGGTCGCCGCATACACTATTCTCAGAATGACTTGGTTGAGTACTCACCAGTCACAGAAAAGCATCTTACGGATGGCATGACA
**bla(M)-001**	**GCGGTATTATCCCGTATTGACGCCGGGCAAGAGCAACTCGGTCGCCGCATACACTATTCTCAGAATGACTTGGTTGAGTACTCACCAGTCACAGAAAAGCATCTTACGGATGGCATGACA**
bla(M)-002	GCGGTATTATCCCGTATTGACGCCGGGCAAGAGCAACTCGGTCGCCGCATACACTATTCTCAGAATGACTTGGTTGAGTACTCACCAGTCACAGAAAAGCATCTTACGGATGGCATGACA
**bla(M)-003**	**GCGGTATTATCCCGTATTGACGCCGGGCAAGAGCAACTCGGTCGCCGCATACACTATTCTCAGAATGACTTGGTTGAGTACTCACCAGTCACAGAAAAGCATCTTACGGATGGCATGACA**
**bla(M)-004**	**GCGGTATTATCCCGTATTGACGCCGGGCAAGAGCAACTCGGTCGCCGCATACACTATTCTCAGAATGACTTGGTTGAGTACTCACCAGTCACAGAAAAGCATCTTACGGATGGCATGACA**
AmpR-007corr	GCGGTATTATCCCGTATTGACGCCGGGCAAGAGCAACTCGGTCGCCGCATACACTATTCTCAGAATGACTTGGTTGAGTACTCACCAGTCACAGAAAAGCATCTTACGGATGGCATGACA
AmpR-009corr	GCGGTATTATCCCGTGTTGACGCCGGGCAAGAGCAACTCGGTCGCCGCATACACTATTCTCAGAATGACTTGGTTGAGTACTCACCAGTCACAGAAAAGCATCTTACGGATGGCATGACA
bla(M)-001corr	GCGGTATTATCCCGTATTGACGCCGGGCAAGAGCAACTCGGTCGCCGCATACACTATTCTCAGAATGACTTGGTTGAGTACTCACCAGTCACAGAAAAGCATCTTACGGATGGCATGACA
bla(M)-003corr	GCGGTATTATCCCGTATTGACGCCGGGCAAGAGCAACTCGGTCGCCGCATACACTATTCTCAGAATGACTTGGTTGAGTACTCACCAGTCACAGAAAAGCATCTTACGGATGGCATGACA
bla(M)-004corr	GCGGTATTATCCCGTATTGACGCCGGGCAAGAGCAACTCGGTCGCCGCATACACTATTCTCAGAATGACTTGGTTGAGTACTCACCAGTCACAGAAAAGCATCTTACGGATGGCATGACA
	* * ** ****** ***************** ** ******** **** *** ** ** ** ************* ** ** ***** ********** *************

AmpR-013	GTAAGAGAATTATGCAGTGCTGCCATAACCATGAGTGATAACACTGCGGCCAACTTACTTCTGACAACGATCGGAGGACCGAAGGAGCTAACCGCTTTTTTGCACAACATGGGGGATCAT
AmpR-017	GTAAGAGAATTATGCAGTGCTGCCATAACCATGAGTGATAACACTGCGGCCAACTTACTTCTGACAACGATCGGAGGACCGAAGGAGCTAACCGCTTTTTTGCACAACATGGGGGATCAT
AmpR-001	GTAAGAGAATTATGCAGTGCTGCCATAACCATGAGTGATAACACTGCGGCCAACTTACTTCTGACAACGATCGGAGGACCGAAGGAGCTAACCGCTTTTTTGCACAACATGGGGGATCAT
AmpR-021	GTAAGAGAATTGTGTAGTGCTGCCATTACCATGAGCGACAACACCGCGGCCAACTTACTTCTGACAACGATCGGAGGCCCTAAGGAGCTGACTGCATTTCTTCATAATATGGGTGATCAT
AmpR-016	GTAAGAGAATTATGCAGTGCTGCCATAACCATGAGTGATAACACTGCGGCCAACTTACTTCTGACAACGATCGGAGGACCGAAGGAGCTAACCGCTTTTTTGCACAACATGGGGGATCAT
AmpR-022	GTAAGAGAATTATGCAGTGCTGCCATAACCATGAGTGATAACACTGCGGCCAACTTACTTCTGACAACGATCGGAGGACCGAAGGAGCTAACCGCTTTTTTGCACAACATGGGGGATCAT
AmpR-010	GTAAGAGAATTATGCAGTGCTGCCATAACCATGAGTGATAACACTGCGGCCAACTTACTTCTGACAACGATCGGAGGACCGAAGGAGCTAACCGCTTTTTTGCACAACATGGGGGATCAT
AmpR-004	GTAAGAGAATTATGCAGTGCTGCCATAACCATGAGTGATAACACTGCGGCCAACTTACTTCTGACAACGATCGGAGGACCGAAGGAGCTAACCGCTTTTTTGCACAACATGGGGGATCAT
AmpR-019	GTAAGAGAATTATGCAGTGCTGCCATAACCATGAGTGATAACACTGCGGCCAACTTACTTCTGACAACGATCGGAGGACCGAAGGAGCTAACCGCTTTTTTTCACAACATGGGGGATCAT
AmpR-020	GTAAGAGAATTATGCAGTGCTGCCATAACCATGAGTGATAACACTGCGGCCAACTTACTTCTGACAACGATCGGAGGACCGAAGGAGCTAACCGCTTTTTTGCACAACATGGGGGATCAT
AmpR-014	GTAAGAGAATTATGCAGTGCTGCCATAACCATGAGTGATAACACTGCGGCCAACTTACTTCTGACAACGATCGGAGGACCGAAGGAGCTAACCGCTTTTTTGCACAACATGGGGGATCAT
**AmpR-007**	**GTAAGAGAATTATGCAGTGCTGCCATAACCATGAGTGATAACACTGCGGCCAACTTACTTCTGACAACGATCGGAGGACCGAAGGAGCTAACCGCTTTTTTGCACAACATGGGGGATCAT**
AmpR-015	GTAAGAGAATTATGCAGTGCTGCCATAACCATGAGTGATAACACTGCGGCCAACTTACTTCTGGCAACGATCGGAGGACCGAAGGAGCTAACCGCTTTTTTGCACAACATGGGGGATCAT
AmpR-011	GTAAGAGAATTATGCAGTGCTGCCATAACCATGAGTGATAACACTGCGGCCAACTTACTTCTGACAACTATCGGAGGACCGAAGGAGCTAACCGCTTTTTTGCACAACATGGGGGATCAT
**AmpR-002**	**GTAAGAGAATTATGCAGTGCTGCCATAACCATGAGTGATAACACTGCGGCCAACTTACTTCTGACAACGATCGGAGGACCGAAGGAGCTAACCGCTTTTTTGCACAACATGGGGGATCAT**
AmpR-018	GTAAGAGAATTATGCAGTGCTGCCATAACCATGAGTGATAACACTGCGGCCAACTTACTTCTGACAACGATTGGAGGACCGAAGGAGCTAACCGCTTTTTTGCACAACATGGGGGATCAT
AmpR-003	GTAAGAGAATTATGCAGTGCTGCCATAACCATGAGTGATAACACTGCGGCCAACTTACTTCTGACAACGATCGGAGGACCGAAGGAGCTAACCGCTTTTTTGCACAACATGGGGGATCAT
AmpR-008	GTAAGAGAATTATGCAGTGCTGCCATAACCATGAGTGATAACACTGCGGCCAACTTACTTCTGACAACGATCGGAGGACCGAAGGAGCTAACCGCTTTTTTGCACAACATGGGGGATCAT
AmpR-005	GTAAGAGAATTATGCAGTGCTGCCATAACCATGAGTGATAACACTGCGGCCAACTTACTTCTGACAACGATCGGAGGACCGAAGGAGCTAACCGCTTTTTTGCACAACATGGGGGATCAT
AmpR-006	GTAAGAGAATTATGCAGTGCTGCCATAACCATGAGTGATAACACTGCGGCCAACTTACTTCTGACAACGATCGGAGGACCGAAGGAGCTAACCGCTTTTTTGCACAACATGGGGGATCAT
**AmpR-009**	**GTAAGAGAATTATGCAGTGCTGCCATAACCATGAGTGATAACACTGCGGCCAACTTACTTCTGACAACGATCGGAGGACCGAAGGAGCTAACCGCTTTTTTGCACAACATGGGGGATCAT**
AmpR-012	GTAAGAGAATTATGCAGTGCTGCCATAACCATGAGTGATAACACTGCGGCCAACTTACTTCTGACAACGATCGGAGGACCGAAGGAGCTAACCGCTTTTTTGCACAACATGGGGGATCAT
AmpR-023	GTAAGAGAATTATGCAGTGCTGCCATAACCATGAGTGATAACACTGCGGCCAACTTACTTCTGACAACGATCGGAGGACCGAAGGAGCTAACCGCTTTTTTGCACAACATGGGGGATCAT
**bla(M)-001**	**GTAAGAGAATTATGCAGTGCTGCCATAACCATGAGTGATAACACTGCGGCCAACTTACTTCTGACAACGATCGGAGGACCGAAGGAGCTAACCGCTTTTTTGCACAACATGGGGGATCAT**
bla(M)-002	GTAAGAGAATTATGCAGTGCTGCCATAACCATGAGTGATAACACTGCGGCCAACTTACTTCTGACAACGATCGGAGGACCGAAGGAGCTAACCGCTTTTTTGCACAACATGGGGGATCAT
**bla(M)-003**	**GTAAGAGAATTATGCAGTGCTGCCATAACCATGAGTGATAACACTGCGGCCAACTTACTTCTGACAACGATCGGAGGACCGAAGGAGCTAACCGCTTTTTTGCACAACATGGGGGATCAT**
**bla(M)-004**	**GTAAGAGAATTATGCAGTGCTGCCATAACCATGAGTGATAACACTGCGGCCAACTTACTTCTGACAACGATCGGAGGACCGAAGGAGCTAACCGCTTTTTTGCACAACATGGGGGATCAT**
AmpR-007corr	GTAAGAGAATTATGCAGTGCTGCCATAACCATGAGTGATAACACTGCGGCCAACTTACTTCTGACAACGATCGGAGGACCGAAGGAGCTAACCGCTTTTTTGCACAACATGGGGGATCAT
AmpR-009corr	GTAAGAGAATTATGCAGTGCTGCCATAACCATGAGTGATAACACTGCGGCCAACTTACTTCTGACAACGATCGGAGGACCGAAGGAGCTAACCGCTTTTTTGCACAACATGGGGGATCAT
bla(M)-001corr	GTAAGAGAATTATGCAGTGCTGCCATAACCATGAGTGATAACACTGCGGCCAACTTACTTCTGACAACGATCGGAGGACCGAAGGAGCTAACCGCTTTTTTGCACAACATGGGGGATCAT
bla(M)-003corr	GTAAGAGAATTATGCAGTGCTGCCATAACCATGAGTGATAACACTGCGGCCAACTTACTTCTGACAACGATCGGAGGACCGAAGGAGCTAACCGCTTTTTTGCACAACATGGGGGATCAT
bla(M)-004corr	GTAAGAGAATTATGCAGTGCTGCCATAACCATGAGTGATAACACTGCGGCCAACTTACTTCTGACAACGATCGGAGGACCGAAGGAGCTAACCGCTTTTTTGCACAACATGGGGGATCAT
	*********** ** *********** ******** ** ***** ***************** **** ** ***** ** ******** ** ** *** * ** ** ***** ******

Sequence Alignments

AmpR-013	GTAACTCGCCTTGATCGTTGGGAACCGGAGCTGAATGAAGCCATACCAAACGACGAGCGTGACACCACGATGCCTGTAGCAATGGCAACAACGTTGCGCAAACTATTAACTGGCGAACTA
AmpR-017	GTAACTCGCCTTGATCGTTGGGAACCGGAGCTGAATGAAGCCATACCAAACGACGAGCGTGACACCACGATGCCTGCAGCAATGGCAACAACGTTGCGCAAACTATTAACTGGCGAACTA
AmpR-001	GTAACTCGCCTTGATCGTTGGGAACCGGAGCTGAATGAAGCCATACCAAACGACGAGCGTGACACCACGATGCCTGCAGCAATGGCAACAACGTTGCGCAAACTATTAACTGGCGAACTA
AmpR-021	GTGACCCGGCTTGACCGCTGGGAACCAGAGTTGAACGAAGCCATACCGAACGACGAGCGTGATACCACGATGCCAGTAGCAATGGCCACAACTCTTCGGAAACTACTCACTGGCGAACTT
AmpR-016	GTAACTCGCCTTGATCGTTGGGAACCGGAGCTGAATGAAGCCATACCAAACGACGAGCGTGACACCACGATGCCTGTAGCAATGGCAACAACGTTGCGCAAACTATTAACTGGCGAACTA
AmpR-022	GTAACTCGCCTTGATCGTTGGGAACCGGAGCTGAATGAAGCCATACCAAACGACGAGCGTGACACCACGATGCCTGTAGCAATGGCAACAACGTTGCGCAAACTATTAACTGGCGAACTA
AmpR-010	GTAACTCGCCTTGATCGTTGGGAACCGGAGCTGAATGAAGCCATACCAAACGACGAGCGTGACACCACGATGCCTGTAGCGATGGCAACAACGTTGCGCAAACTATTAACTGGCGAACTA
AmpR-004	GTAACTCGCCTTGATCGTTGGGAACCGGAGCTGAATGAAGCCATACCAAACGACGAGAGTGACACCACGATGCCTGTAGCAATGCCAACAACGTTGCGCAAACTATTAACTGGCGAACTA
AmpR-019	GTAACTCGCCTTGATCGTTGGGAACCGGAGCTGAATGAAGCCATACCAAACGACGAGCGTGACACCACGATGCCTGTAGCAATGGCAACAACGTTGCGCAAACTATTAACTGGCGAACTA
AmpR-020	GTAACTCGCCTTGATCGTTGGGAACCGGAGCTGAATGAAGCCATACCAAACGACGAGCGTGACACCACGATGCCTGTAGCAATGGCAACAACGTTGCGCAAACTATTAACTGGCGAACTA
AmpR-014	GTAACTCGCCTTGATCGTTGGGAACCGGAGCTGAATGAAGCCATACCAAACGACGAGCGTGACACCACGATGCCTGTAGCAATGGCAACAACGTTGCGCAAACTATTAACTGGCGAACTA
**AmpR-007**	**GTAACTCGCCTTGATCGTTGGGAACCGGAGCTGAATGAAGCCATACCAAACGACGAGCGTGACACCACGATGCCTGTAGCAATGGCAACAACGTTGCGCAAACTATTAACTGGCGAACTA**
AmpR-015	GTAACTCGCCTTGATCGTTGGGAACCGGAGCTGAATGAAGCCATACCAAACGACGAGCGTGACACCACGATGCCTGTAGCAATGGCAACAACGTTGCGCAAACTATTAACTGGCGAACTA
AmpR-011	GTAACTCGCCTTGATCGTTGGGAACCGGAGCTGAATGAAGCCATACCAAACGACGAGCGTGACACCACGATGCCTGTAGCAATGGCAACAACGTTGCGCAAACTATTAACTGGCGAACTA
**AmpR-002**	**GTAACTCGCCTTGATCGTTGGGAACCGGAGCTGAATGAAGCCATACCAAACGACGAGAGTGACACCACGATGCCTGTAGCAATGCCAACAACGTTGCGCAAACTATTAACTGGCGAACTA**
AmpR-018	GTAACTCGCCTTGATCGTTGGGAACCGGAGCTGAATGAAGCCATACCAAACGACGAGCGTGACACCACGATGCCTGTAGCAATGGCAACAACCTTGCGTAAACTATTAACTGGCGAACTA
AmpR-003	GTAACTCGCCTTGATCGTTGGGAACCGGAGCTGAATGAAGCCATACCAAACGACGAGCGTGACACCACGATGCCTGTAGCAATGGCAACAACGTTGCGCAAACTATTAACTGGCGAACTA
AmpR-008	GTAACTCGCCTTGATCGTTGGGAACCGGAGCTGAATGAAGCCATACCAAACGACGAGCGTGACACCACGATGCCTGTAGCAATGGCAACAACGTTGCGCAAACTATTAACTGGCGAACTA
AmpR-005	GTAACTCGCCTTGATCGTTGGGAACCGGAGCTGAATGAAGCCATACCAAACGACGAGCGTGACACCACGATGCCTGTAGCAATGGCAACAACGTTGCGCAAACTATTAACTGGCGAACTA
AmpR-006	GTAACTCGCCTTGATCGTTGGGAACCGGAGCTGAATGAAGCCATACCAAACGACGAGCGTGACACCACGATGCCAGCAGCAATGGCAACAACGTTGCGCAAACTATTAACTGGCGAACTA
**AmpR-009**	**GTAACTCGCCTTGATCGTTGGGAACCGGAGCTGAATGAAGCCATACCAAACGACGAGCGTGACACCACGATGCCTGTAGCAATGGCAACAACGTTGCGCAAACTATTAACTGGCGAACTA**
AmpR-012	GTAACTCGCCTTGATCGTTGGGAACCGAAGCTGAATGAAGCCATACCAAACGACGAGCGTGACACCACGATGCCTGTAGCAATGGCAACAACGTTGCGCAAACTATTAACTGGCGAACTA
AmpR-023	GTAACTCGCCTTGATCGTTGGGAACCGGAGCTGAATGAAGCCATACCAAACGACGAGCGTGACACCACGATGCCTGCAGCAATGGCAACAACGTTGCGCAAACTATTAACTGGCGAACTA
**bla(M)-001**	**GTAACTCGCCTTGATCGTTGGGAACCGGAGCTGAATGAAGCCATACCAAACGACGAGCGTGACACCACGATGCCTGCAGCAATGGCAACAACGTTGCGCAAACTATTAACTGGCGAACTA**
bla(M)-002	GTAACTCGCCTTGATCGTTGGGAACCGGAGCTGAATGAAGCCATACCAAACGACGAGCGTGACACCACGATGCCTGTAGCAATGGCAACAACGTTGCGCAAACTATTAACTGGCGAACTA
**bla(M)-003**	**GTAACTCGCCTTGATCGTTGGGAACCGGAGCTGAATGAAGCCATACCAAACGACGAGCGTGACACCACGATGCCTGTAGCAATGGCAACAACGTTGCGCAAACTATTAACTGGCGAACTA**
**bla(M)-004**	**GTAACTCGCCTTGATCGTTGGGAACCGGAGCTGAATGAAGCCATACCAAACGACGAGCGTGACACCACGATGCCTGTAGCAATGGCAACAACGTTGCGCAAACTATTAACTGGCGAACTA**
AmpR-007corr	GTAACTCGCCTTGATCGTTGGGAACCGGAGCTGAATGAAGCCATACCAAACGACGAGCGTGACACCACGATGCCTGTAGCAATGGCAACAACGTTGCGCAAACTATTAACTGGCGAACTA
AmpR-009corr	GTAACTCGCCTTGATCGTTGGGAACCGGAGCTGAATGAAGCCATACCAAACGACGAGCGTGACACCACGATGCCTGTAGCAATGGCAACAACGTTGCGCAAACTATTAACTGGCGAACTA
bla(M)-001corr	GTAACTCGCCTTGATCGTTGGGAACCGGAGCTGAATGAAGCCATACCAAACGACGAGCGTGACACCACGATGCCTGCAGCAATGGCAACAACGTTGCGCAAACTATTAACTGGCGAACTA
bla(M)-003corr	GTAACTCGCCTTGATCGTTGGGAACCGGAGCTGAATGAAGCCATACCAAACGACGAGCGTGACACCACGATGCCTGTAGCAATGGCAACAACGTTGCGCAAACTATTAACTGGCGAACTA
bla(M)-004corr	GTAACTCGCCTTGATCGTTGGGAACCGGAGCTGAATGAAGCCATACCAAACGACGAGCGTGACACCACGATGCCTGTAGCAATGGCAACAACGTTGCGCAAACTATTAACTGGCGAACTA
	** ** ** ***** ** ******** ** **** *********** ********* **** *********** * *** *** * ***** * ** ****** * ***********

AmpR-013	CTTACTCTAGCTTCCCGGCAACAATTAATAGACTGGATGGAGGCGGATAAAGTTGCAGGACCACTTCTGCGCTCGGCCCTTCCGGCTGGCTGGTTTATTGCTGATAAATCTGGAGCCGGT
AmpR-017	CTTACTCTAGCTTCCCGGCAACAATTAATAGACTGGATGGAGGCGGATAAAGTTGCAGGACCACTTCTGCGCTCGGCCCTTCCGGCTGGCTGGTTTATTGCTGATAAATCTGGAGCCGGT
AmpR-001	CTTACTCTAGCTTCCCGGCAACAATTAATAGACTGGATGGAGGCGGATAAAGTTGCAGGACCACTTCTGCGCTCGGCCCTTCCGGCTGGCTGGTTTATTGCTGATAAATCTGGAGCCGGT
AmpR-021	CTTACTCTAGCATCACGACAGCAGCTCATAGACTGGATGGAGGCGGACAAAGTAGCAGGACCACTTCTTCGCTCGGCCCTCCCTGCTGGCTGGTTCATTGCTGACAAATCGGGGGCCGGT
AmpR-016	CTTACTCTAGCTTCCCGGCAACAATTAATAGACTGGATGGAGGCGGATAAAGTTGCAGGACCACTTCTGCGCTCGGCCCTTCCGGCTGGCTGGTTTATTGCTGATAAATCTGGAGCCGGT
AmpR-022	CTTACTCTAGCTTCCCGGCAACAATTAATAGACTGGATGGAGGCGGATAAAGTTGCAGGACCACTTCTGCGCTCGGCCCTTCCGGCTGGCTGGTTTATTGCTGATAAATCTGGAGCCGGT
AmpR-010	CTTACTCTAGCTTCCCGGCAACAATTAATAGACTGGATGGAGGCGGATAAAGTTGCAGGACCACTTCTGCGCTCGGCCCTTCCGGCTGGCTGGTTTATTGCTGATAAATCCGGAGCCGGT
AmpR-004	CTTACTCTAGCTTCCCGGCAACAATTAATAGACTGGATGGAGGCGGATAAAGTTGCAGGACCACTTCTGCGCTCGGCCCTTCCGGCTGGCTGGTTTATTGCTGATAAATCTGGAGCCGGT
AmpR-019	CTTACTCTAGCTTCCCGGCAACAATTAATAGACTGGATGGAGGCGGATAAAGTTGCAGGACCACTTCTGCGCTCGGCCCTTCCGGCTGGCTGGTTTATTGCTGATAAATCTGGAGCCGGT
AmpR-020	CTTACTCTAGCTTCCCGGCAACAATTAATAGACTGGATGGAGGCGGATAAAGTTGCAGGACCACTTCTGCGCTCGGCCCTTCCGGCTGGCTGGTTTATTGCTGATAAATCTGGAGCCGGT
AmpR-014	CTTACTCTAGCTTCCCGGCAACAATTAATAGACTGGATGGAGGCGGATAAAGTTGCAGGACCACTTCTGCGCTCGGCCCTTCCGGCTGGCTGGTTTATTGCTGATAAATCTGGAGCCGGT
**AmpR-007**	**CTTACTCTAGCTTCCCGGCAACAATTAATAGACTGGATGGAGGCGGATAAAGTTGCAGGACCACTTCTGCGCTCGGCCCTTCCGGCTGGCTGGTTTATTGCTGATAAATCTGGAGCCGGT**
AmpR-015	CTTACTCTAGCTTCCCGGCAACAATTAATAGACTGGATGGAGGCGGATAAAGTTGCAGGATCACTTCTGCGCTCGGCCCTCCCGGCTGGCTGGTTTATTGCTGATAAATCTGGAGCCGGT
AmpR-011	CTTACTCTAGCTTCCCGGCAACAATTAATAGACTGGATGGAGGCGGATAAAGTTGCAGGACCACTTCTGCGCTCGGCCCTTCCGGCTGGCTGGTTTATTGCTGATAAATCTGGAGCCGGT
**AmpR-002**	**CTTACTCTAGCTTCCCGGCAACAATTAATAGACTGAATGGAGGCGGATAAAGTTGCAGGACCACTTCTGCGCTCGGCCCTTCCGGCTGGCTGGTTTATTGCTGATAAATCTGGAGCCGGT**
AmpR-018	CTTACTCTAGCTTCCCGGCAACAGTTGATAGACTGGATGGAGGCGGATAAAGTTGCAGGACCACTTCTGCGCTCGGCCCTTCCGGCTGGCTGGTTTATTGCTGATAAATCTGGAGCCGGT
AmpR-003	CTTACTCTAGCTTCCCGGCAACAATTAATAGACTGGATGGAGGCGGATAAAGTTGCAGGACCACTTCTGCGCTCGGCCCTTCCGGCTGGCTGGTTTATTGCTGATAAATCTGGAGCCGGT
AmpR-008	CTTACTCTAGCTTCCCGGCAACAATTAATAGACTGGATGGAGGCGGATAAAGTTGCAGGACCACTTCTGCGCTCGGCCCTTCCGGCTGGCTGGTTTATTGCTGATAAATCTGGAGCCGGT
AmpR-005	CTTACTCTAGCTTCCCGGCAACAATTAATAGACTGGATGGAGGCGGATAAAGTTGCAGGACCACTTCTGCGCTCGGCCCTTCCGGCTGGCTGGTTTATTGCTGATAAATCTGGAGCCGGT
AmpR-006	CTTACTCTAGCTTCCCGGCAACAATTAATAGACTGGATGGAGGCGGATAAAGTTGCAGGACCACTTCTGCGCTCGGCCCTTCCGGCTGGCTGGTTTATTGCTGATAAATCTGGAGCCGGT
**AmpR-009**	**CTTACTCTAGCTTCCCGGCAACAATTAATAGACTGGATGGAGGCGGATAAAGTTGCAGGACCACTTCTGCGCTCGGCCCTTCCGGCTGGCTGGTTTATTGCTGATAAATCTGGAGCCGGT**
AmpR-012	CTTACTCTAGCTTCCCGGCAACAATTAATAGACTGGATGGAGGCGGATAAAGTTGCAGGACCACTTCTGCGCTCGGCCCTTCCGGCTGGCTGGTTTATTGCTGATAAATCTGGAGCCGGT
AmpR-023	CTTACTCTAGCTTCCCGGCAACAATTCATAGACTGGATGGAGGCGGATAAAGTTGCAGGACCACTTCTGCGCTCGGCCCTTCCGGCTGGCTGGTTTATTGCTGATAAATCTGGAGCCGGT
**bla(M)-001**	**CTTACTCTAGCTTCCCGGCAACAATTAATAGACTGGATGGAGGCGGATAAAGTTGCAGGACCACTTCTGCGCTCGGCCCTTCCGGCTGGCTGGTTTATTGCTGATAAATCTGGAGCCGGT**
bla(M)-002	CTTACTCTAGCTTCCCGGCAACAATTAATAGACTGGATGGAGGCGGATAAAGTTGCAGGACCACTTCTGCGCTCGGCCCTTCCGGCTGGCTGGTTTATTGCTGATAAATCTGGAGCCGGT
**bla(M)-003**	**CTTACTCTAGCTTCCCGGCAACAATTAATAGACTGGATGGAGGCGGATAAAGTTGCAGGACCACTTCTGCGCTCGGCCCTTCCGGCTGGCTGGTTTATTGCTGATAAATCTGGAGCCGGT**
**bla(M)-004**	**CTTACTCTAGCTTCCCGGCAACAATTAATAGACTGGATGGAGGCGGATAAAGTTGCAGGACCACTTCTGCGCTCGGCCCTTCCGGCTGGCTGGTTTATTGCTGATAAATCTGGAGCCGGT**
AmpR-007corr	CTTACTCTAGCTTCCCGGCAACAATTAATAGACTGGATGGAGGCGGATAAAGTTGCAGGACCACTTCTGCGCTCGGCCCTTCCGGCTGGCTGGTTTATTGCTGATAAATCTGGAGCCGGT
AmpR-009corr	CTTACTCTAGCTTCCCGGCAACAATTAATAGACTGGATGGAGGCGGATAAAGTTGCAGGACCACTTCTGCGCTCGGCCCTTCCGGCTGGCTGGTTTATTGCTGATAAATCTGGAGCCGGT
bla(M)-001corr	CTTACTCTAGCTTCCCGGCAACAATTAATAGACTGGATGGAGGCGGATAAAGTTGCAGGACCACTTCTGCGCTCGGCCCTTCCGGCTGGCTGGTTTATTGCTGATAAATCTGGAGCCGGT
bla(M)-003corr	CTTACTCTAGCTTCCCGGCAACAATTAATAGACTGGATGGAGGCGGATAAAGTTGCAGGACCACTTCTGCGCTCGGCCCTTCCGGCTGGCTGGTTTATTGCTGATAAATCTGGAGCCGGT
bla(M)-004corr	CTTACTCTAGCTTCCCGGCAACAATTAATAGACTGGATGGAGGCGGATAAAGTTGCAGGACCACTTCTGCGCTCGGCCCTTCCGGCTGGCTGGTTTATTGCTGATAAATCTGGAGCCGGT
	*********** ** ** ** ** * ******** *********** ***** ****** ******* *********** ** *********** ******** ***** ** ******

Sequence Alignments

```
AmpR-013          GAGCGTGGGTCTCGCGGTATCATTGCAGCACTGGGGCCAGATGGTAAGCCCTCCCGTATCGTAGTTATCTACACGACGGGGAGTCAGGCAACTATGGATGAACGAAATAGACAGATCGCT
AmpR-017          GAGCGTGGGTCTCGCGGTATCATTGCAGCACTGGGGCCAGATGGTAAGCCCTCCCGTATCGTAGTTATCTACACGACGGGGAGTCAGGCAACTATGGATGAACGAAATAGACAGATCGCT
AmpR-001          GAGCGTGGGTCTCGCGGTATCATTGCAGCACTGGGGCCAGATGGTAAGCCCTCCCGTATCGTAGTTATCTACACGACGGGGAGTCAGGCAACTATGGATGAACGAAATAGACAGATCGCT
AmpR-021          GAACGCGGCTCTCGCGGCATCATTGCTGCGCTGGGGCCTGATGGTAAGCCCTCACGAATCGTAGTGATCTACACGACGGGGAGTCAGGCCACTATGGACGAACGAAATAGGCAGATCGCT
AmpR-016          GAGCGTGGGTCTCGCGGTATCATTGCAGCACTGGGGCCAGATGGTAAGCCCTCCCGTATCGTAGTTATCTACACGACGGGGAGTCAGGCAACTATGGATGAACGAAATAGACAGATCGCT
AmpR-022          GAGCGTGGGTCTCGCGGTATCATTGCAGCACTGGGGCCAGATGGTAAGCCCTCCCGTATCGTAGTTATCTACACGACGGGGAGTCAGGCAGCTATGGATGAACGAAATAGACAGATCGCT
AmpR-010          GAGCGTGGTTCTCGCGGTATCATCGCAGCGCTGGGGCCAGATGGTAAGCCCTCCCGTATCGTAGTTATCTACACGACGGGGAGTCAGGCAACTATGGATGAACGAAATAGACAGATCGCT
AmpR-004          GAGCGTGGGTCTCGCGGTATCATTGCAGCACTGGGGCCAGATGGTAAGCGCTCCCGTATCGTAGTTATCTACACGACGGGGAGTCAGGCAACTATGGATGAACGAAATAGACAGATCGCT
AmpR-019          GAGCGTGGGTCTCGCGGTATCATTGCAGCACTGGGGCCAGATGGTAAGCCCTCCCGTATCGTAGTTATCTACACGACGGGCAGTCAGGCAACTATGGATGAACGAAATAGACAGATCGCT
AmpR-020          GAGCGTGGGTCTCGCGGTATCATTGCAGCACTGGGGCCAGATGGTAAGCCCTCCCGTATCGTAGTTATCTACACGACGGGGAGTCAGGCAACTATGGATGAACGAAATAGACAGATCGCT
AmpR-014          GAGCGTGGGTCTCGCGGTATCATTGCAGCACTGGGGCCAGATGGTAAGCCCTCCCGTATCGTAGTTATCTACACGACGGGGAGTCAGGCAACTATGGATGAACGAAATAGACAGATCGCT
AmpR-007          GAGCGTGGGTCTCGCGGTATCATTGCAGCACTGGGGCCAGATGGTAAGCCCTCCCGTATCGTAGTTATCTACACGACGGGGAGTCAGGCAACTATGGATGAACGAAATAGACAGATCGCT
AmpR-015          GAGCGTGGGTCTCGCGGTATCATTGCAGCACTGGGGCCAGATGGTAAGCCCTCCCGCATCGTAGTTATCTACACGACGGGGAGTCAGGCAACTATGGATGAACGAAATAGACAGATCGCT
AmpR-011          GAGCGTGGGTCTCGCGGTATCATTGCAGCACTGGGGCCAGATGGTAAGCCCTCCCGTATCGTAGTTATCTACACGACGGGGAGTCAGGCAACTATGGATGAACGAAATAGACAGATCGCT
AmpR-002          GAGCGTGGGTCTCGCGGTATCATTGCAGCACTGGGGCCAGATGGTAAGCGCTCCCGTATCGTAGTTATCTACACGACGGGGAGTCAGGCAACTATGGATGAACGAAATAGACAGATCGCT
AmpR-018          GAGCGTGGGTCTCGCGGTATCATTGCAGCACTGGGGCCAGATGGTAAGCCCTCCCGTATCGTAGTTATCTACACGACGGGGAGTCAGGCAACTATGGATGAACGAAATAGACAGATCGCT
AmpR-003          GAGCGTGGGTCTCGCGGTATCATTGCAGCACTGGGGCCAGATGGTAAGCCCTCCCGTATCGTAGTTATCTACACGACGGGGAGTCAGGCAACTATGGATGAACGAAATAGACAGATCGCT
AmpR-008          GAGCGTGGTTCTCGCGGTATCATTGCAGCACTGGGGCCAGATGGTAAGCCCTCCCGTATCGTAGTTATCTACACGACGGGGAGTCAGGCAACTATGGATGAACGAAATAGACAGATCGCT
AmpR-005          GAGCGTGGGTCTCGCGGTATCATTGCAGCACTGGGGCCAGATGGTAAGCGCTCCCGTATCGTAGTTATCTACACGACGGGGAGTCAGGCAACTATGGATGAACGAAATAGACAGATCGCT
AmpR-006          GAGCGTGGGTCTCGCGGTATCATTGCAGCACTGGGGCCAGATGGTAAGCCCTCCCGTATCGTAGTTATCTACACGACGGGGAGTCAGGCAACTATGGATGAACGAAATAGACAGATCGCT
AmpR-009          GAGCGTGGGTCTCGCGGTATCATTGCAGCACTGGGGCCAGATGGTAAGCCCTCCCGTATCGTAGTTATCTACACGACGGGGAGTCAGGCAACTATGGATGAACGAAATAGACAGATCGCT
AmpR-012          GAGCGTGGGTCTCGCGGTATCATTGCAGCACTGGGGCCAGATGGTAAGCCCTCCCGTATCGTAGTTATCTACACGACGGGGAGTCAGGCAACTATGGATGAACGAAATAGACAGATCGCT
AmpR-023          GAGCGTGGCTCTCGCGGTATCATTGCAGCACTGGGGCCAGATGGTAAGCCCTCCCGTATCGTAGTTATCTACACGACGGGGAGTCAGGCAACTATGGATGAACGAAATAGACAGATCGCT
bla(M)-001        GAGCGTGGGTCTCGCGGTATCATTGCAGCACTGGGGCCAGATGGTAAGCCCTCCCGTATCGTAGTTATCTACACGACGGGGAGTCAGGCAACTATGGATGAACGAAATAGACAGATCGCT
bla(M)-002        GAGCGTGGGTCTCGCGGTATCATTGCAGCACTGGGGCCAGATGGTAAGCCCTCCCGTATCGTAGTTATCTACACGACGGGGAGTCAGGCAACTATGGATGAACGAAATAGACAGATCGCT
bla(M)-003        GAGCGTGGTTCTCGCGGTATCATTGCAGCACTGGGGCCAGATGGTAAGCCCTCCCGTATCGTAGTTATCTACACGACGGGGAGTCAGGCAACTATGGATGAACGAAATAGACAGATCGCT
bla(M)-004        GAGCGTGGGTCTCGCGGTATCATTGCAGCACTGGGGCCAGATGGTAAGCCCTCCCGTATCGTAGTTATCTACACGACGGGGAGTCAGGCAACTATGGATGAACGAAATAGACAGATCGCT
AmpR-007corr      GAGCGTGGGTCTCGCGGTATCATTGCAGCACTGGGGCCAGATGGTAAGCCCTCCCGTATCGTAGTTATCTACACGACGGGGAGTCAGGCAACTATGGATGAACGAAATAGACAGATCGCT
AmpR-009corr      GAGCGTGGGTCTCGCGGTATCATTGCAGCACTGGGGCCAGATGGTAAGCCCTCCCGTATCGTAGTTATCTACACGACGGGGAGTCAGGCAACTATGGATGAACGAAATAGACAGATCGCT
bla(M)-001corr    GAGCGTGGGTCTCGCGGTATCATTGCAGCACTGGGGCCAGATGGTAAGCCCTCCCGTATCGTAGTTATCTACACGACGGGGAGTCAGGCAACTATGGATGAACGAAATAGACAGATCGCT
bla(M)-003corr    GAGCGTGGTTCTCGCGGTATCATTGCAGCACTGGGGCCAGATGGTAAGCCCTCCCGTATCGTAGTTATCTACACGACGGGGAGTCAGGCAACTATGGATGAACGAAATAGACAGATCGCT
bla(M)-004corr    GAGCGTGGGTCTCGCGGTATCATTGCAGCACTGGGGCCAGATGGTAAGCCCTCCCGTATCGTAGTTATCTACACGACGGGGAGTCAGGCAACTATGGATGAACGAAATAGACAGATCGCT
                  ** ** ** ******** ***** ** ** ******** ********** *** ** ******** ************* *********  ******* *********** *********

AmpR-013          GAGATAGGTGCCTCACTGATTAAGCATTGGTAA----------------------------------------
AmpR-017          GAGATAGGTGCCTCACTGATTAAGCATTGGTAA----------------------------------------
AmpR-001          GAGATAGGTGCCTCACTGATTAAGCATTGGTAA----------------------------------------
AmpR-021          GAGATCGGTGCCTCACTGATCAAGCACTGGTAA----------------------------------------
AmpR-016          GAGATAGGTGCCTCACTGATTAAGCATTGGTAA----------------------------------------
AmpR-022          GAGATAGGTGCCTCACTGATTAAGCATTGGTAA----------------------------------------
AmpR-010          GAGATAGGTGCCTCACTGATTAAGCATTGGTAA----------------------------------------
AmpR-004          GAGATAGGTGCCTCACTGATTAAGCATTGGTAA----------------------------------------
AmpR-019          GAGATAGGTGCCTCACTGATTAAGCATTGGTAA----------------------------------------
AmpR-020          GAGATAGGTGCCTCACTGATTAAGCATTGGTAA----------------------------------------
AmpR-014          GAGATAGGTGCCTCACTGATTAAGCATTGGTAA----------------------------------------
AmpR-007          GAGATAGGTGCCTCACTGATTAAGCATTGG---------------------------------------------
AmpR-015          GAGATAGGTGCCTCACTGATTAAGCATTGGTAA----------------------------------------
AmpR-011          GAGATAGGTGCCTCACTGATTAAGCATTGGTAA----------------------------------------
AmpR-002          GAGATAGGTGCCTCACTGATTAAGCATTGGTAA----------------------------------------
AmpR-018          GAGATAGGTGCCTCACTGATTAAGCATTGGTAA----------------------------------------
AmpR-003          GAGATAGGTGCCTCACTGATTAAGCATTGGTAA----------------------------------------
AmpR-008          GAGATAGGTGCCTCACTGATTAAGCATTGGTAA----------------------------------------
AmpR-005          GAGATAGGTGCCTCACTGATTAAGCATTGGTAA----------------------------------------
AmpR-006          GAGATAGGTGCCTCACTGATTAAGCATTGGTAA----------------------------------------
AmpR-009          GAGATAGGTGCCTCACTGATTAAGCATTGGTAACTG-------------------------------------
AmpR-012          GAGATAGGTGCCTCACTGATTAAGCATTGGTAA----------------------------------------
AmpR-023          GAGATAGGTGCCTCACTGATTAAGCATTGGTAG----------------------------------------
bla(M)-001        GAGATAGGTGCCTCACTGATTAAGCATTGGTAA----------------------------------------
bla(M)-002        GAGATAGGTGCCTCACTGATTAAGCATTGGTAA----------------------------------------
bla(M)-003        GAGATAGGTGCCTCACTGATTAAGCATTGGTAA----------------------------------------
bla(M)-004        GAGATAGGTGCCTCACTGATTAAGCATTGG---------------------------------------------
AmpR-007corr      GAGATAGGTGCCTCACTGATTAAGCATTGGCCTCAGGATTAG---------------------------------
AmpR-009corr      GAGATAGGTGCCTCACTGATTAAGCATTGGTAA----------------------------------------
bla(M)-001corr    GAGATAGGTGCCTCACTGATTAAGCATTGGTAA----------------------------------------
bla(M)-003corr    GAGATAGGTGCCTCACTGATTAAGCATTGGTAA----------------------------------------
bla(M)-004corr    GAGATAGGTGCCTCACTGATTAAGCATTGGCTGTTATCAACAAGTTTGTACAAAAAAGCTGAACGAGAAACGTAA
                  ***** ************** ***** ***
```

ATGC = synonymous
ATGC = conservative/semi-conservative amino acid substitution
ATGC = non-conservative amino acid substitution

AmpR gene is *bla* (β-lactamase) from *E. coli*
bla(M) lacks the signal peptide
Sequences in bold are incorrectly annotated
AmpR-009 was identified by coordinates that were +3 from the actual start codon and is actually identical to AmpR-016 – -009 merged with -016
AmpR-002 is probably identical to AmpR-004 but the sequence contains an in-frame STOP codon. This is likely a sequence error as AmpR is only bacterial selection marker for the plasmids carrying AmpR-002 – -002 merged with -004
bla(M)-001 coordinates did not start at the START codon. It is identical to AmpR-001 and has been merged with it
Similarly bla(M)-003 is identical to AmpR-008 and has been merged with it

Sequence Alignments

**Name of Variant**	**Alignment of Translated Protein**	**Size (aa)**	**# Var aa**
AmpR-013	MSIQHFRVALIPFFAAFCLPVFAHPETLVKVKDAEDQLGARVGYIELDLNSGKILESFRPEERFP----MMSTFKVLLCGAVLSRIDAGQEQLGRRIHYSQNDLVEYSPVTEKHLTDGMT	286	0
AmpR-017	MSIQHFRVALIPFFAAFCLPVFAHPETLVKVKDAEDQLGARVGYIELDLNSGKILESFRPEERFP----MMSTFKVLLCGAVLSRVDAGQEQLGRRIHYSQNDLVEYSPVTEKHLTDGMT	286	2
AmpR-001	MSIQHFRVALIPFFAAFCLPVFAHPETLVKVKDAEDQLGARVGYIELDLNSGKILESFRPEERFP----MMSTFKVLLCGAVLSRIDAGQEQLGRRIHYSQNDLVEYSPVTEKHLTDGMT	286	1
AmpR-021	MSIQHFRVALIPFFAAFCLPVFAHPETLVKVKDAEDQLGARVGYIELDLNSGKILESFRPEERFP----MMSTFKVLLCGAVLSRIDAGQEQLGRRIHYSQNDLVEYSPVTEKHLTDGMT	286	0
AmpR-016	MSIQHFRVALIPFFAAFCLPVFAHPETLVKVKDAEDQLGARVGYIELDLNSGKILESFRPEERFP----MMSTFKVLLCGAVLSRVDAGQEQLGRRIHYSQNDLVEYSPVTEKHLTDGMT	286	1
AmpR-022	MSIQHFRVALIPFFAAFCLPVFAHPETLVKVKDAEDQLGARVGYIELDLNSGKILESFRPEERFP----MMSTFKVLLCGAVLSRIDAGQEQLGRRIHYSQNDLVEYSPVTEKHLTDGMT	286	1
AmpR-010	MSIQHFRVALIPFFAAFCLPVFAHPETLVKVKDAEDQLGARVGYIELDLNSGKILESFRPEERFP----MMSTFKVLLCGAVLSRIDAGQEQLGRRIHYSQNDLVEYSPVTEKHLTDGMT	286	0
AmpR-004	MSIQHFRVALIPFFAAFCLPVFAHPETLVKVKDAEDQLGARVGYIELDLNSGKILESFRPEERFP----MMSTFKVLLCDTLLSRIDAGQEQLGRRIHYSQNDLVEYSPVTEKHLTDGMT	286	6
AmpR-019	MSIQHFRVALIPFFAAFCLPVFAHPETLVKVKDAEDQLGARVGYIELDLNSGKILESFRPEERFP----MMSTFKVLLCGAVLSRIDAGQEQLGRRIHYSQNDLVEYSPVTEKHLTDGMT	286	1
AmpR-020	MSIQHFRVALIPFFAAFCLPVFAHPETLVKVKDAEDQLGARVGYIELDLNSGKILESFRPEERFQVEVAMMSTFKVLLCGAVLSRIDAGQEQLGRRIHYSQNDLVEYSPVTEKHLTDGMT	290	1/4i
AmpR-014	MSIQHFRVALIPFFAAFCLPVFAHPETLVKVKDAEDQLGARVGYIELDLNSGKILESFRPEERSP----MMSTFKVLLCGAVLSRVDAGQEQLGRRIHYSQNDLVEYSPVTEKHLTDGMT	286	2
AmpR-007corr	MSIQHFRVALIPFFAAFCLPVFAHPETLVKVKDAEDQLGARVGYIELDLNSGKILESFRPEERFP----MMSTFKVLLCGAVLSRIDAGQEQLGRRIHYSQNDLVEYSPVTEKHLTDGMT	286	0
AmpR-015	MSIQHFRVALIPFFAAFCLPVFAHPETLVKVKDAEDQLGARVGYIELDLNSGKILESLRPEERFP----MMSTFKVLLCGAVLSRIDAGQEQLGRRIHYSQNDLVEYSPVTEKHLTDGMT	286	3
AmpR-011	MSIQHFRVALIPFFAAFCLPVFAHPETLVKVKDAEDQLGARVGYIELDLNSGKILESFRPEERFP----MMSTFKVLLCGAVLSRIDAGQEQLGRRIHYSQNDLVEYSPVTEKHLTDGMT	286	0
**AmpR-002**	**MSIQHFRVALIPFFAAFCLPVFAHPETLVKVKDAEDQLGARVGYIELDLNSGKILESFRPEERFP----MMSTFKVLLCDTLLSRIDAGQEQLGRRIHYSQNDLVEYSPVTEKHLTDGMT**	**166**	**5/79d**
AmpR-018	MSIQHFRVALIPFFAAFCLPVFAHPETLVKVKDAEDQLGARVGYIELDLNSGKILESFRPEERFP----MMSTFKVLLCGAVLSRIDAGQEQLGRRIHYSQNDLVEYSPVTEKHLTDGMT	286	0
AmpR-003	MSIQHFRVALIPFFAAFCLPVFAHPETLVKVKDAEDQLGARVGYIELDLNSGKILESFRPEERFP----MMSTFKVLLCGAVLSRIDAGQEQLGRRIRYSQNDLVEYSPVTEKHLTDGMT	286	1
AmpR-008	MSIQHFRVALIPFFAAFCLPVFAHPETLVKVKDAEDQLGARVGYIELDLNSGKILESFRPEERFP----MMSTFKVLLCGAVLSRIDAGQEQLGRRIHYSQNDLVEYSPVTEKHLTDGMT	286	0
AmpR-005	MSIQHFRVALIPFFAAFCLPVFAHPETLVKVKDAEDQLGARVGYIELDLNSGKILESFRPEERFP----MMSTFKVLLCGAVLSRVDAGQEQLGRRIHYSQNDLVEYSPVTEKHLTDGMT	286	2
AmpR-006	MSIQHFRVALIPFFAAFCLPVFAHPETLVKVKDAEDQLGARVGYIELDLNSGKILESFRPEERFP----MMSTFKVLLCGAVLSRDDAGQEQLGRRIHYSQNDLVEYSPVTEKHLTDGMT	286	2
AmpR-012	MSIQHFRVALIPFFAAFCLPVFAHPETLVKVKDAEDQLGARVGYIELDLNSGKILESFRPEERFP----MMSTFKVLLCGAVLSRIDAGQEQLGRRIHYSQNDLVEYSPVTEKHLTDGMT	286	1
AmpR-023	MSIQHFRVALIPFFAAFCLPVFAHPETLVKVKDAEDQLGARVGYIELDLNSGKILESFRPEERFP----MMSTFKVLLCGAVLSRIDAGQEQLGRRIHYSQNDLVEYSPVTEKHLTDGMT	286	1
bla(M)-002	----------------------MDPETLVKVKDAEDQLGARVGYIELDLNSGKILESFRPEERFP----MMSTFKVLLCGAVLSRIDAGQEQLGRRIHYSQNDLVEYSPVTEKHLTDGMT	264	0
bla(M)-004corr	----------------------MDPETLVKVKDAEDQLGARVGYIELDLNSGKILESFRPEERFP----MMSTFKVLLCGAVLSRIDAGQEQLGRRIHYSQNDLVEYSPVTEKHLTDGMT	278	0
	********************* **********************************:***** **********.::*** ***********:**********************		

AmpR-013	VRELCSAAITMSDNTAANLLLTTIGGPKELTAFLHNMGDHVTRLDRWEPELNEAIPNDERDTTMPVAMATTLRKLLTGELLTLASRQQLIDWMEADKVAGPLLRSALPAGWFIADKSGAG
AmpR-017	VRELCSAAITMSDNTAANLLLTTIGGPKELTAFLHNMGDHVTRLDRWEPELNEAIPNDERDTTMPAAMATTLRKLLTGELLTLASRQQLIDWMEADKVAGPLLRSALPAGWFIADKSGAG
AmpR-001	VRELCSAAITMSDNTAANLLLTTIGGPKELTAFLHNMGDHVTRLDRWEPELNEAIPNDERDTTMPAAMATTLRKLLTGELLTLASRQQLIDWMEADKVAGPLLRSALPAGWFIADKSGAG
AmpR-021	VRELCSAAITMSDNTAANLLLTTIGGPKELTAFLHNMGDHVTRLDRWEPELNEAIPNDERDTTMPVAMATTLRKLLTGELLTLASRQQLIDWMEADKVAGPLLRSALPAGWFIADKSGAG
AmpR-016	VRELCSAAITMSDNTAANLLLTTIGGPKELTAFLHNMGDHVTRLDRWEPELNEAIPNDERDTTMPVAMATTLRKLLTGELLTLASRQQLIDWMEADKVAGPLLRSALPAGWFIADKSGAG
AmpR-022	VRELCSAAITMSDNTAANLLLTTIGGPKELTAFLHNMGDHVTRLDRWEPELNEAIPNDERDTTMPVAMATTLRKLLTGELLTLASRQQLIDWMEADKVAGPLLRSALPAGWFIADKSGAG
AmpR-010	VRELCSAAITMSDNTAANLLLTTIGGPKELTAFLHNMGDHVTRLDRWEPELNEAIPNDERDTTMPVAMATTLRKLLTGELLTLASRQQLIDWMEADKVAGPLLRSALPAGWFIADKSGAG
AmpR-004	VRELCSAAITMSDNTAANLLLTTIGGPKELTAFLHNMGDHVTRLDRWEPELNEAIPNDESDTTMPVAMPTTLRKLLTGELLTLASRQQLIDWMEADKVAGPLLRSALPAGWFIADKSGAG
AmpR-019	VRELCSAAITMSDNTAANLLLTTIGGPKELTAFFHNMGDHVTRLDRWEPELNEAIPNDERDTTMPVAMATTLRKLLTGELLTLASRQQLIDWMEADKVAGPLLRSALPAGWFIADKSGAG
AmpR-020	VRELCSAAITMSDNTAANLLLTTIGGPKELTAFLHNMGDHVTRLDRWEPELNEAIPNDERDTTMPVAMATTLRKLLTGELLTLASRQQLIDWMEADKVAGPLLRSALPAGWFIADKSGAG
AmpR-014	VRELCSAAITMSDNTAANLLLTTIGGPKELTAFLHNMGDHVTRLDRWEPELNEAIPNDERDTTMPVAMATTLRKLLTGELLTLASRQQLIDWMEADKVAGPLLRSALPAGWFIADKSGAG
AmpR-007corr	VRELCSAAITMSDNTAANLLLTTIGGPKELTAFLHNMGDHVTRLDRWEPELNEAIPNDERDTTMPVAMATTLRKLLTGELLTLASRQQLIDWMEADKVAGPLLRSALPAGWFIADKSGAG
AmpR-015	VRELCSAAITMSDNTAANLLLATIGGPKELTAFLHNMGDHVTRLDRWEPELNEAIPNDERDTTMPVAMATTLRKLLTGELLTLASRQQLIDWMEADKVAGSLLRSALPAGWFIADKSGAG
AmpR-011	VRELCSAAITMSDNTAANLLLTTIGGPKELTAFLHNMGDHVTRLDRWEPELNEAIPNDERDTTMPVAMATTLRKLLTGELLTLASRQQLIDWMEADKVAGPLLRSALPAGWFIADKSGAG
**AmpR-002**	**VRELCSAAITMSDNTAANLLLTTIGGPKELTAFLHNMGDHVTRLDRWEPELNEAIPNDESDTTMPVAMPTTLRKLLTGELLTLASRQQLID-MEADKVAGPLLRSALPAGWFIADKSGAG**
AmpR-018	VRELCSAAITMSDNTAANLLLTTIGGPKELTAFLHNMGDHVTRLDRWEPELNEAIPNDERDTTMPVAMATTLRKLLTGELLTLASRQQLIDWMEADKVAGPLLRSALPAGWFIADKSGAG
AmpR-003	VRELCSAAITMSDNTAANLLLTTIGGPKELTAFLHNMGDHVTRLDRWEPELNEAIPNDERDTTMPVAMATTLRKLLTGELLTLASRQQLIDWMEADKVAGPLLRSALPAGWFIADKSGAG
AmpR-008	VRELCSAAITMSDNTAANLLLTTIGGPKELTAFLHNMGDHVTRLDRWEPELNEAIPNDERDTTMPVAMATTLRKLLTGELLTLASRQQLIDWMEADKVAGPLLRSALPAGWFIADKSGAG
AmpR-005	VRELCSAAITMSDNTAANLLLTTIGGPKELTAFLHNMGDHVTRLDRWEPELNEAIPNDERDTTMPVAMATTLRKLLTGELLTLASRQQLIDWMEADKVAGPLLRSALPAGWFIADKSGAG
AmpR-006	VRELCSAAITMSDNTAANLLLTTIGGPKELTAFLHNMGDHVTRLDRWEPELNEAIPNDERDTTMPAAMATTLRKLLTGELLTLASRQQLIDWMEADKVAGPLLRSALPAGWFIADKSGAG
AmpR-009corr	VRELCSAAITMSDNTAANLLLTTIGGPKELTAFLHNMGDHVTRLDRWEPELNEAIPNDERDTTMPVAMATTLRKLLTGELLTLASRQQLIDWMEADKVAGPLLRSALPAGWFIADKSGAG
AmpR-012	VRELCSAAITMSDNTAANLLLTTIGGPKELTAFLHNMGDHVTRLDRWEPKLNEAIPNDERDTTMPVAMATTLRKLLTGELLTLASRQQLIDWMEADKVAGPLLRSALPAGWFIADKSGAG
AmpR-023	VRELCSAAITMSDNTAANLLLTTIGGPKELTAFLHNMGDHVTRLDRWEPELNEAIPNDERDTTMPAAMATTLRKLLTGELLTLASRQQLIDWMEADKVAGPLLRSALPAGWFIADKSGAG
bla(M)-002	VRELCSAAITMSDNTAANLLLTTIGGPKELTAFLHNMGDHVTRLDRWEPELNEAIPNDERDTTMPVAMATTLRKLLTGELLTLASRQQLIDWMEADKVAGPLLRSALPAGWFIADKSGAG
bla(M)-004corr	VRELCSAAITMSDNTAANLLLTTIGGPKELTAFLHNMGDHVTRLDRWEPELNEAIPNDERDTTMPVAMATTLRKLLTGELLTLASRQQLIDWMEADKVAGPLLRSALPAGWFIADKSGAG
	*********************:***********:***************:********* *****.**.********************** ********.*******************

Sequence Alignments

```
AmpR-013           ERGSRGIIAALGPDGKPSRIVVIYTTGSQATMDERNRQIAEIGASLIKHW--------------
AmpR-017           ERGSRGIIAALGPDGKPSRIVVIYTTGSQATMDERNRQIAEIGASLIKHW--------------
AmpR-001           ERGSRGIIAALGPDGKPSRIVVIYTTGSQATMDERNRQIAEIGASLIKHW--------------
AmpR-021           ERGSRGIIAALGPDGKPSRIVVIYTTGSQATMDERNRQIAEIGASLIKHW--------------
AmpR-016           ERGSRGIIAALGPDGKPSRIVVIYTTGSQATMDERNRQIAEIGASLIKHW--------------
AmpR-022           ERGSRGIIAALGPDGKPSRIVVIYTTGSQAAMDERNRQIAEIGASLIKHW--------------
AmpR-010           ERGSRGIIAALGPDGKPSRIVVIYTTGSQATMDERNRQIAEIGASLIKHW--------------
AmpR-004           ERGSRGIIAALGPDGKRSRIVVIYTTGSQATMDERNRQIAEIGASLIKHW--------------
AmpR-019           ERGSRGIIAALGPDGKPSRIVVIYTTGSQATMDERNRQIAEIGASLIKHW--------------
AmpR-020           ERGSRGIIAALGPDGKPSRIVVIYTTGSQATMDERNRQIAEIGASLIKHW--------------
AmpR-014           ERGSRGIIAALGPDGKPSRIVVIYTTGSQATMDERNRQIAEIGASLIKHW--------------
AmpR-007corr       ERGSRGIIAALGPDGKPSRIVVIYTTGSQATMDERNRQIAEIGASLIKHWPQD-----------
AmpR-015           ERGSRGIIAALGPDGKPSRIVVIYTTGSQATMDERNRQIAEIGASLIKHW--------------
AmpR-011           ERGSRGIIAALGPDGKPSRIVVIYTTGSQATMDERNRQIAEIGASLIKHW--------------
AmpR-002           ERGSRGIIAALGPDGKRSRIVVIYTTGSQATMDERNRQIAEIGASLIKHW--------------
AmpR-018           ERGSRGIIAALGPDGKPSRIVVIYTTGSQATMDERNRQIAEIGASLIKHW--------------
AmpR-003           ERGSRGIIAALGPDGKPSRIVVIYTTGSQATMDERNRQIAEIGASLIKHW--------------
AmpR-008           ERGSRGIIAALGPDGKPSRIVVIYTTGSQATMDERNRQIAEIGASLIKHW--------------
AmpR-005           ERGSRGIIAALGPDGKRSRIVVIYTTGSQATMDERNRQIAEIGASLIKHW--------------
AmpR-006           ERGSRGIIAALGPDGKPSRIVVIYTTGSQATMDERNRQIAEIGASLIKHW--------------
AmpR-009           ERGSRGIIAALGPDGKPSRIVVIYTTGSQATMDERNRQIAEIGASLIKHW--------------
AmpR-012           ERGSRGIIAALGPDGKPSRIVVIYTTGSQATMDERNRQIAEIGASLIKHW--------------
AmpR-023           ERGSRGIIAALGPDGKPSRIVVIYTTGSQATMDERNRQIAEIGASLIKHW--------------
bla(M)-002         ERGSRGIIAALGPDGKPSRIVVIYTTGSQATMDERNRQIAEIGASLIKHW--------------
bla(M)-004corr     ERGSRGIIAALGPDGKPSRIVVIYTTGSQATMDERNRQIAEIGASLIKHWLLSTSLYKKAERET
                   **************** *************:*******************
```

```
Key     ATGC = change in nucleotide relative to consensus sequence – synonymous
        ATGC = change in nucleotide relative to consensus sequence – conservative/semi-conservative amino acid substitution
        ATGC = change in nucleotide relative to consensus sequence – non-conservative amino acid substitution
        ATGC = predicted protein sequence following a nonsense mutation
```

Sequence Alignments

Name of Variant	# of Occur	Alignment	Size (bp)	# Var bp	Sources*
CmR-013	48	ATGGAGAAAAAAATCACTGGATATACCACCGTTGATATATCCCAATGGCATCGTAAAGAACATTTTGAGGCATTTCAGTCAGTTGCTCAATGTACCTATAACCAGACCGTTCAGCTGGAT	660	0	In,No,Ne
CmR-016	39	ATGGAGAAAAAAATCACTGGATATACCACCGTTGATATATCCCAATGGCATCGTAAAGAACATTTTGAGGCATTTCAGTCAGTTGCTCAATGTACCTATAACCAGACCGTTCAGCTGGAT	660	1	In,No,Ne
CmR-015	31	ATGGAGAAAAAAATCACTGGATATACCACCGTTGATATATCCCAATGGCATCGTAAAGAACATTTTGAGGCATTTCAGTCAGTTGCTCAATGTACCTATAACCAGACCGTTCAGCTGGAT	660	2	Qi,Ev,Ta
CmR-014	29	ATGGAGAAAAAAATCACTGGATATACCACCGTTGATATATCCCAATGGCATCGTAAAGAACATTTTGAGGCATTTCAGTCAGTTGCTCAATGTACCTATAACCAGACCGTTCAGCTGGAT	660	1	Qi,Cl,Lu
CmR-010	21	ATGGAGAAAAAAATCACTGGATATACCACCGTTGATATATCCCAATGGCATCGTAAAGAACATTTTGAGGCATTTCAGTCAGTTGCTCAATGTACCTATAACCAGACCGTTCAGCTGGAT	660	1	Or,IMAGE
CmR-004	13	ATGGAGAAAAAAATCACTGGATATACCACCGTTGATATATCCCAATGGCATCGTAAAGAACATTTTGAGGCATTTCAGTCAGTTGCTCAATGTACCTATAACCAGACCGTTCAGCTGGAT	660	2	Ca
CmR-011	12	ATGGAGAAAAAAATCACTGGATATACCACCGTTGATATATCCCAATGGCATCGTAAAGAACATTTTGAGGCATTTCAGTCAGTTGCTCAATGTACCTATAACCAGACCGTTCAGCTGGAT	660	1	No
CmR-002	4	ATGGAGAAAAAAATCACTGGATATACCACCGTTGATATATCCCAATGGCATCGTAAAGAACATTTTGAGGCATTTCAGTCAGTTGCTCAATGTACCTATAACCAGACCGTTCAGCTGGAT	660	1	16377204
CmR-012	4	ATGGAGAAAAAAATCACTGGATATACCACCGTTGATATATCCCAATGGCATCGTAAAGAACATTTTGAGGCATTTCAGTCAGTTGCTCAATGTACCTATAACCAGACCGTTCAGCTGGAT	660	3	MCSG
CmR-001	3	ATGGAGAAAAAAATCACTGGATATACCACCGTTGATATATCCCAATGGCATCGTAAAGAACATTTTGAGGCATTTCAGTCAGTTGCTCAATGTACCTATAACCAGACCGTTCAGCTGGAT	660	1	Ag
CmR-005	2	ATGGAGAAAAAAATCACTGGATATACCACCGTTGATATATCCCAATGGCATCGTAAAGAACATTTTGAGGCATTTCAGTCAGTTGCTCAATGTACCTATAACCAGACCGTTCAGCTGGAT	660	2	Or
CmR-003	1	ATGGAGAAAAAAATCACTGGATATACCACCGTTGATATATCCCAATGGCATCGTAAAGAACATTTTGAGGCATTTCAGTCAGTTGCTCAATGTACCTATAACCAGACCGTTCAGCTCGAT	660	2	Qi
CmR-006	1	ATGGAGAAAAAAATCACTGGATATACCACCGTTGATATATCCCAATGGCATCGTAAAGAACATTTTGAGGCATTTCAGTCAGTTGCTCAATGTACCTATAACCAGACCGTTCAGCTGGAT	660	3	No
CmR-007	1	ATGGAGAAAAAAATCACTGGATATACCACCGTTGATATATCCCAATGGCATCGTAAAGAACATTTTGAGGCATTTCAGTCAGTTGCTCAATGTACCTATAACCAGACCGTTCAGCTGGAT	660	2	In
CmR-008	1	ATGGAGAAAAAAATCACTGGATATACCACCGTTGATATATCCCAATGGCATCGTAAAGAACATTTTGAGGCATTTCAGTCAGTTGCTCAATGTACCTATAACCAGACCGTTCAGCTGGAT	681	1/1d	In
**CmR-009**	**1**	**ATGGAGAAAAAAATCACTGGATATACCACCGTTGATATATCCCAATGGCATCGTAAAGAACATTTTGAGGCATTTCAGTCAGTTGCTCAATGTACCTATAACCAGACCGTTCAGCTGGAT**	**654**	**6d**	**In**
CmR-009corr		ATGGAGAAAAAAATCACTGGATATACCACCGTTGATATATCCCAATGGCATCGTAAAGAACATTTTGAGGCATTTCAGTCAGTTGCTCAATGTACCTATAACCAGACCGTTCAGCTGGAT	684	1d	
		** ***			

CmR-013	ATTACGGCCTTTTTAAAGACCGTAAAGAAAAATAAGCACAAGTTTTATCCGGCCTTTATTCACATTCTTGCCCGCCTGATGAATGCTCATCCGGAATTCCGTATGGCAATGAAAGACGGT
CmR-016	ATTACGGCCTTTTTAAAGACCGTAAAGAAAAATAAGCACAAGTTTTATCCGGCCTTTATTCACATTCTTGCCCGCCTGATGAATGCTCATCCGGAATTCCGTATGGCAATGAAAGACGGT
CmR-015	ATTACGGCCTTTTTAAAGACCGTAAAGAAAAATAAGCACAAGTTTTATCCGGCCTTTATTCACATTCTTGCCCGCCTGATGAATGCTCATCCGGAATTTCGTATGGCAATGAAAGACGGT
CmR-014	ATTACGGCCTTTTTAAAGACCGTAAAGAAAAATAAGCACAAGTTTTATCCGGCCTTTATTCACATTCTTGCCCGCCTGATGAATGCTCATCCGGAATTTCGTATGGCAATGAAAGACGGT
CmR-010	ATTACGGCCTTTTTAAAGACCGTAAAGAAAAATAAGCACAAGTTTTATCCGGCCTTTATTCACATTCTTGCCCGCCTGATGAATGCTCATCCGGAGTTCCGTATGGCAATGAAAGACGGT
CmR-004	ATTACGGCCTTTTTAAAGACCGTAAAGAAAAATAAGCACAAGTTTTATCCGGCCTTTATTCACATTCTTGCCCGCCTGATGAATGCTCATCCGGAGTTCCGTATGGCAATGAAAGACGGT
CmR-011	ATTACGGCCTTTTTAAAGACCGTAAAGAAAAATAAGCACAAGTTTTATCCGGCCTTTATTCACATTCTTGCCCGCCTGATGAATGCTCATCCGGAATTCCGTATGGCAATGAAAGACGGT
CmR-002	ATTACGGCCTTTTTAAAGACCGTAAAGAAAAATAAGCACAAGTTTTATCCGGCCTTTATTCACATTCTTGCCCGCCTGATGAATGCTCATCCGGAATTCCGTATGGCAATGAAAGACGGT
CmR-012	ATTACGGCCTTTTTAAAGACCGTAAAGAAAAATAAGCACAAGTTTTATCCGGCCTTTATTCACATTCTTGCCCGCCTGATGAATGCTCATCCGGAGTTCCGTATGGCAATGAAAGACGGT
CmR-001	ATTACGGCCTTTTTAAAGACCGTAAAGAAAAATAAGCACAAGTTTTATCCGGCCTTTATTCACATTCTTGCCCGCCTGATGAATGCTCATCCGGAATTACGTATGGCAATGAAAGACGGT
CmR-005	ATTACGGCCTTTTTAAAGACCGTAAAGAAAAATAAGCACAAGTTTTATCCGGCCTTTATTCACATTCTTGCCCGCCTGATGAATGCTCATCCGGAGTTCCGTATGGCAATGAAAGACGGT
CmR-003	ATTACGGCCTTTTTAAAGACCGTAAAGAAAAATAAGCACAAGTTTTATCCGGCCTTTATTCACATTCTTGCCCGCCTGATGAATGCTCATCCGGAATTTCGTATGGCAATGAAAGACGGT
CmR-006	ATTACGGCCTTTTTAAAGACCGTAAAGAAAAATAAGCACAAGTTTTATCCGGCCTTTATTCACATTCTTGCCCGCCTGATGAATGCTCATCCGGAGTTCCGTATGGCAATGAAAGACGGT
CmR-007	ATTACGGCCTTTTTAAAGACCGTAAAGAAAAATAAGCACAAGTTTTATCCGGCCTTTATTCACATTCTTGCCCGCCTGATGAATGCTCATCCGGAATTCCGTATGGCAATGAAAGACGGT
CmR-008	ATTACGGCCTTTTTAAAGACCGTAAAGAAAAATAAGCACAAGTTTTATCCGGCCTTTATTCACATTCTTGCCCGCCTGATGAATGCTCATCCGGAATTCCGTATGGCAATGAAAGACGGT
**CmR-009**	**ATTACGGCCTTTTTAAAGACCGTAAAGAAAAATAAGCACAAGTTTTATCCGGCCTTTATTCACATTCTTGCCCGCCTGATGAATGCTCATCCGGAATTCCGTATGGCAATGAAAGACGGT**
CmR-009corr	ATTACGGCCTTTTTAAAGACCGTAAAGAAAAATAAGCACAAGTTTTATCCGGCCTTTATTCACATTCTTGCCCGCCTGATGAATGCTCATCCGGAATTCCGTATGGCAATGAAAGACGGT
	*** ** *********************

CmR-013	GAGCTGGTGATATGGGATAGTGTTCACCCTTGTTACACCGTTTTCCATGAGCAAACTGAAACGTTTTCATCGCTCTGGAGTGAATACCACGACGATTTCCGGCAGTTTCTACACATATAT
CmR-016	GAGCTGGTGATATGGGATAGTGTTCACCCTTGTTACACCGTTTTCCATGAGCAAACTGAAACGTTTTCATCGCTCTGGAGTGAATACCACGACGATTTCCGGCAGTTTCTACACATATAT
CmR-015	GAGCTGGTGATATGGGATAGTGTTCACCCTTGTTACACCGTTTTCCATGAGCAAACTGAAACGTTTTCATCGCTCTGGAGTGAATACCACGACGATTTCCGGCAGTTTCTACACATATAT
CmR-014	GAGCTGGTGATATGGGATAGTGTTCACCCTTGTTACACCGTTTTCCATGAGCAAACTGAAACGTTTTCATCGCTCTGGAGTGAATACCACGACGATTTCCGGCAGTTTCTACACATATAT
CmR-010	GAGCTGGTGATATGGGATAGTGTTCACCCTTGTTACACCGTTTTCCATGAGCAAACTGAAACGTTTTCATCGCTCTGGAGTGAATACCACGACGATTTCCGGCAGTTTCTACACATATAT
CmR-004	GAGCTGGTGATATGGGATAGTGTTCACCCTTGTTACACCGTTTTCCATGAGCAAACTGAAACGTTTTCATCGCTCTGGAGTGAATACCACGACGATTTCCGGCAGTTTCTACACATATAT
CmR-011	GAGCTGGTGATATGGGATAGTGTTCACCCTTGTTACACCGTTTTCCATGAGCAAACTGAAACGTTTTCATCGCTCTGGAGTGAATACCACGACGATTTCCGGCAGTTTCTACACATATAT
CmR-002	GAGCTGGTGATATGGGATAGTGTTCACCCTTGTTACACCGTTTTCCATGAGCAAACTGAAACGTTTTCATCGCTCTGGAGTGAATACCACGACGATTTCCGGCAGTTTCTACACATATAT
CmR-012	GAGCTGGTGATATGGGATAGTGTTCACCCTTGTTACACCGTTTTCCATGAGCAAACTGAAACGTTTTCATCGCTCTGGAGTGAATACCACGACGATTTCCGGCAGTTTCTACACATATAT
CmR-001	GAGCTGGTGATATGGGATAGTGTTCACCCTTGTTACACCGTTTTCCATGAGCAAACTGAAACGTTTTCATCGCTCTGGAGTGAATACCACGACGATTTCCGGCAGTTTCTACACATATAT
CmR-005	GAGCTGGTGATATGGGATAGTGTTCACCCTTGTTACACCGTTTTCCATGAGCAAACTGAAACGTTTTCATCGCTCTGGAGTGAATACCACGACGATTTCCGGCAGTTTCTACACATATAT
CmR-003	GAGCTGGTGATATGGGATAGTGTTCACCCTTGTTACACCGTTTTCCATGAGCAAACTGAAACGTTTTCATCGCTCTGGAGTGAATACCACGACGATTTCCGGCAGTTTCTACACATATAT
CmR-006	GAGCTGGTGATATGGGATAGTGTTCACCCTTGTTACACCGTTTTCCATGAGCAAACTGAAACGTTTTCATCGCTCTGGAGTGAATACCACGACGATTTCCGGCAGTTTCTACACATATAT
CmR-007	GAGCTGGTGATATGGGATAGTGTTCACCCTTGTTACACCGTTTTCCATGAGCAAACTGAAACGTTTTCATCGCTCTGGAGTGAATACCACGACGATTTCCGGCAGTTTCTACACATATAT
CmR-008	GAGCTGGTGATATGGGATAGTGTTCACCCTTGTTACACCGTTTTCCATGAGCAAACTGAAACGTTTTCATCGCTCTGGAGTGAATACCACGACGATTTCCGGCAGTTTCTACACATATAT
**CmR-009**	**GAGCTGGTGATATGGGATAGTGTTCACCCTTGTTACACCGTTTTCCATGAGCAAACTGAAACGTTTTCATCGCTCTGGAGTGAATACCACGACGATTTCCGGCAGTTTCTACACATATAT**
CmR-009corr	GAGCTGGTGATATGGGATAGTGTTCACCCTTGTTACACCGTTTTCCATGAGCAAACTGAAACGTTTTCATCGCTCTGGAGTGAATACCACGACGATTTCCGGCAGTTTCTACACATATAT
	**

Sequence Alignments

CmR-013	TCGCAAGATGTGGCGTGTTACGGTGAAAACCTGGCCTATTTCCCTAAAGGGTTTATTGAGAATATGTTTTTCGTCTCAGCCAATCCCTGGGTGAGTTTCACCAGTTTTGATTTAAACGTG
CmR-016	TCGCAAGATGTGGCGTGTTACGGTGAAAACCTGGCCTATTTCCCTAAAGGGTTTATTGAGAATATGTTTTTCGTCTCAGCCAATCCCTGGGTGAGTTTCACCAGTTTTGATTTAAACGTG
CmR-015	TCGCAAGATGTGGCGTGTTACGGTGAAAACCTGGCCTATTTCCCTAAAGGGTTTATTGAGAATATGTTTTTCGTCTCAGCCAATCCCTGGGTGAGTTTCACCAGTTTTGATTTAAACGTG
CmR-014	TCGCAAGATGTGGCGTGTTACGGTGAAAACCTGGCCTATTTCCCTAAAGGGTTTATTGAGAATATGTTTTTCGTCTCAGCCAATCCCTGGGTGAGTTTCACCAGTTTTGATTTAAACGTG
CmR-010	TCGCAAGATGTGGCGTGTTACGGTGAAAACCTGGCCTATTTCCCTAAAGGGTTTATTGAGAATATGTTTTTCGTCTCAGCCAATCCCTGGGTGAGTTTCACCAGTTTTGATTTAAACGTG
CmR-004	TCGCAAGATGTGGCGTGTTACGGTGAAAACCTGGCCTATTTCCCTAAAGGGTTTATTGAGAATATGTTTTTCGTCTCAGCCAATCCCTGGGTGAGTTTCACCAGTTTTGATTTAAACGTG
CmR-011	TCGCAAGATGTGGCGTGTTACGGTGAAAACCTGGCCTATTTCCCTAAAGGGTTTATTGAGAATATGTTTTTCGTCTCAGCCAATCCCTGGGTGAGTTTCACCAGTTTTGATTTAAACGTG
CmR-002	TCGCAAGATGTGGCGTGTTACGGTGAAAACCTGGCCTATTTCCCTAAAGGGTTTATTGAGAATATGTTTTTCGTCTCAGCCAATCCCTGGGTGAGTTTCACCAGTTTTGATTTAAACGTG
CmR-012	TCGCAAGATGTGGCGTGTTACGGTGAAAACCTGGCCTATTTCCCTAAAGGGTTTATTGAGAATATGTTTTTCGTCTCAGCCAATCCCTGGGTGAGTTTCACCAGTTTTGATTTAAACGTG
CmR-001	TCGCAAGATGTGGCGTGTTACGGTGAAAACCTGGCCTATTTCCCTAAAGGGTTTATTGAGAATATGTTTTTCGTCTCAGCCAATCCCTGGGTGAGTTTCACCAGTTTTGATTTAAACGTG
CmR-005	TCGCAAGATGTGGCGTGTTACGGTGAAAACCTGGCCTATTTCCCTAAAGGGTTTATTGAGAATATGTTTTTCGTTTCAGCCAATCCCTGGGTGAGTTTCACCAGTTTTGATTTAAACGTG
CmR-003	TCGCAAGATGTGGCGTGTTACGGTGAAAACCTGGCCTATTTCCCTAAAGGGTTTATTGAGAATATGTTTTTCGTCTCAGCCAATCCCTGGGTGAGTTTCACCAGTTTTGATTTAAACGTG
CmR-006	TCGCAAGATGTGGCGTGTTACGGTGAAAACCTGGCCTATTTCCCTAAAGGGTTTATTGAGAATATGTTTTTCGTCTCAGCCAATCCCTGGGTGAGTTTCACCAGTTTTGATTTAAACGTG
CmR-007	TCGCAAGATGTGGCGTGTTACGGTGAAAACCTGGCCTATTTCCCTAAAGGGTTTATTGAGAATATGTTTTTCGTCTCAGCCAATCCCTGGGTGAGTTTCACCAGTTTTGATTTAAACGTG
CmR-008	TCGCAAGATGTGGCGTGTTACGGTGAAAACCTGGCCTATTTCCCTAAAGGGTTTATTGAGAATATGTTTTTCGTCTCAGCCAATCCCTGGGTGAGTTTCACCAGTTTTGATTTAAACGTG
**CmR-009**	**TCGCAAGATGTGGCGTGTTACGGTGAAAACCTGGCCTATTTCCCTAAAGGGTTTATTGAGAATATGTTTTTCGTCTCAGCCAATCCCTGGGTGAGTTTCACCAGTTTTGATTTAAACGTG**
CmR-009corr	TCGCAAGATGTGGCGTGTTACGGTGAAAACCTGGCCTATTTCCCTAAAGGGTTTATTGAGAATATGTTTTTCGTCTCAGCCAATCCCTGGGTGAGTTTCACCAGTTTTGATTTAAACGTG
	** ***

CmR-013	GCCAATATGGACAACTTCTTCGCCCCCGTTTTCACCATGGGCAAATATTATACGCAAGGCGACAAGGTGCTGATGCCGCTGGCGATTCAGGTTCATCATGCCGTTTGTGATGGCTTCCAT
CmR-016	GCCAATATGGACAACTTCTTCGCCCCCGTTTTCACCATGGGCAAATATTATACGCAAGGCGACAAGGTGCTGATGCCGCTGGCGATTCAGGTTCATCATGCCGTCTGTGATGGCTTCCAT
CmR-015	GCCAATATGGACAACTTCTTCGCCCCCGTTTTCACCATGGGCAAATATTATACGCAAGGCGACAAGGTGCTGATGCCGCTGGCGATTCAGGTTCATCATGCCGTCTGTGATGGCTTCCAT
CmR-014	GCCAATATGGACAACTTCTTCGCCCCCGTTTTCACCATGGGCAAATATTATACGCAAGGCGACAAGGTGCTGATGCCGCTGGCGATTCAGGTTCATCATGCCGTTTGTGATGGCTTCCAT
CmR-010	GCCAATATGGACAACTTCTTCGCCCCCGTTTTCACCATGGGCAAATATTATACGCAAGGCGACAAGGTGCTGATGCCGCTGGCGATTCAGGTTCATCATGCCGTTTGTGATGGCTTCCAT
CmR-004	GCCAATATGGACAACTTCTTCGCCCCCGTTTTCACAATGGGCAAATATTATACGCAAGGCGACAAGGTGCTGATGCCGCTGGCGATTCAGGTTCATCATGCCGTTTGTGATGGCTTCCAT
CmR-011	GCCAATATGGACAACTTCTTCGCCCCCGTTTTCACTATGGGCAAATATTATACGCAAGGCGACAAGGTGCTGATGCCGCTGGCGATTCAGGTTCATCATGCCGTTTGTGATGGCTTCCAT
CmR-002	GCCAATATGGACAACTTCTTCGCCCCCGTTTTCACGATGGGCAAATATTATACGCAAGGCGACAAGGTGCTGATGCCGCTGGCGATTCAGGTTCATCATGCCGTTTGTGATGGCTTCCAT
CmR-012	GCCAATATGGACAACTTCTTCGCCCCCGTTTTCACTATGGGCAAATACTATACGCAAGGCGACAAGGTGCTGATGCCGCTGGCGATTCAGGTTCATCATGCCGTCTGTGATGGCTTCCAT
CmR-001	GCCAATATGGACAACTTCTTCGCCCCCGTTTTCACCATGGGCAAATATTATACGCAAGGCGACAAGGTGCTGATGCCGCTGGCGATTCAGGTTCATCATGCCGTTTGTGATGGCTTCCAT
CmR-005	GCCAATATGGACAACTTCTTCGCCCCCGTTTTCACCATGGGCAAATATTATACGCAAGGCGACAAGGTGCTGATGCCGCTGGCGATTCAGGTTCATCATGCCGTTTGTGATGGCTTCCAT
CmR-003	GCCAATATGGACAACTTCTTCGCCCCCGTTTTCACCATGGGCAAATATTATACGCAAGGCGACAAGGTGCTGATGCCGCTGGCGATTCAGGTTCATCATGCCGTTTGTGATGGCTTCCAT
CmR-006	GCCAATATGGACAACTTCTTCGCCCCCGTTTTCACTATGGGCAAATATTATACGCAAGGCGACAAGGTGCTGATGCCGCTGGCGATTCAGGTTCATCATGCCGTCTGTGATGGCTTCCAT
CmR-007	GCCAATATGGACAATTTCTTCGCCCCCGTTTTCACCATGGGCAAATATTATACGCAAGGCGACAAGGTGCTGATGCCGCTGGCGATTCAGGTTCATCATGCCGTCTGTGATGGCTTCCAT
CmR-008	GCCAATATGGACAACTTCTTCGCCCCCGTTTTCACCATGGGCAAATATTATACGCAAGGCGACAAGGTGCTGATGCCGCTGGCGATTCAGGTTCATCATGCCGTTTGTGATGGCTTCCAT
**CmR-009**	**GCCAATATGGACAACTTCTTCGCCCCCGTTTTCACCATGGGCAAATATTATACGCAAGGCGACAAGGTGCTGATGCCGCTGGCGATTCAGGTTCATCATGCCGTTTGTGATGGCTTCCAT**
CmR-009corr	GCCAATATGGACAACTTCTTCGCCCCCGTTTTCACCATGGGCAAATATTATACGCAAGGCGACAAGGTGCTGATGCCGCTGGCGATTCAGGTTCATCATGCCGTTTGTGATGGCTTCCAT
	************** ******************** *********** ** ***************

CmR-013	GTCGGCAGAATGCTTAATGAATTACAACAGTACTGCGATGAGTGGCAGGGCGGGGCGTAA-------------------------
CmR-016	GTCGGCAGAATGCTTAATGAATTACAACAGTACTGCGATGAGTGGCAGGGCGGGGCGTAA-------------------------
CmR-015	GTCGGCAGAATGCTTAATGAATTACAACAGTACTGCGATGAGTGGCAGGGCGGGGCGTAA-------------------------
CmR-014	GTCGGCAGAATGCTTAATGAATTACAACAGTACTGCGATGAGTGGCAGGGCGGGGCGTAA-------------------------
CmR-010	GTCGGCAGAATGCTTAATGAATTACAACAGTACTGCGATGAGTGGCAGGGCGGGGCGTAA-------------------------
CmR-004	GTCGGCAGAATGCTTAATGAATTACAACAGTACTGCGATGAGTGGCAGGGCGGGGCGTAA-------------------------
CmR-011	GTCGGCAGAATGCTTAATGAATTACAACAGTACTGCGATGAGTGGCAGGGCGGGGCGTAA-------------------------
CmR-002	GTCGGCAGAATGCTTAATGAATTACAACAGTACTGCGATGAGTGGCAGGGCGGGGCGTAA-------------------------
CmR-012	GTCGGCAGAATGCTTAATGAATTACAACAGTACTGCGATGAGTGGCAGGGCGGGGCGTAA-------------------------
CmR-001	GTCGGCAGAATGCTTAATGAATTACAACAGTACTGCGATGAGTGGCAGGGCGGGGCGTAA-------------------------
CmR-005	GTCGGCAGAATGCTTAATGAATTACAACAGTACTGCGATGAGTGGCAGGGCGGGGCGTAA-------------------------
CmR-003	GTCGGCAGAATGCTTAATGAATTACAACAGTACTGCGATGAGTGGCAGGGCGGGGCGTAA-------------------------
CmR-006	GTCGGCAGAATGCTTAATGAATTACAACAGTACTGCGATGAGTGGCAGGGCGGGGCGTAA-------------------------
CmR-007	GTCGGCAGAATGCTTAATGAATTACAACAGTACTGCGATGAGTGGCAGGGCGGGGCGTAA-------------------------
CmR-008	GTCGGCAGAATGCTTAATGAATTACAACAGTACTGCGATGAGTGGCAGGC-GGGGCGTAATCTA---GAGGATCCGGCTTACTAA
**CmR-009**	**GTCGGCAGAATGCTTAATGAATTACAACAGTACTGCGATGAGTGGCAGGG-GGGG------------------------------**
CmR-009corr	GTCGGCAGAATGCTTAATGAATTACAACAGTACTGCGATGAGTGGCAGGG-GGGGCGTAAACGCCGCGTGGATCCGGCTTACTAA
	*** ****

CmR gene is *cat* (chloramphenicol actyltransferase) from *E. coli*
Sequences in bold are incorrectly annotated
CmR-009 missing STOP codon. Corrected sequence still unique variant

Key
ATGC = change in nucleotide relative to consensus sequence – synonymous
ATGC = change in nucleotide relative to consensus sequence – conservative/semi-conservative amino acid substitution
ATGC = change in nucleotide relative to consensus sequence – non-conservative amino acid substitution

Sequence Alignments

Name of Variant	Alignment of Translated Protein	Size (aa)	# Var aa
CmR-013	MEKKITGYTTVDISQWHRKEHFEAFQSVAQCTYNQTVQLDITAFLKTVKKNKHKFYPAFIHILARLMNAHPEFRMAMKDGELVIWDSVHPCYTVFHEQTETFSSLWSEYHDDFRQFLHIY	219	0
CmR-016	MEKKITGYTTVDISQWHRKEHFEAFQSVAQCTYNQTVQLDITAFLKTVKKNKHKFYPAFIHILARLMNAHPEFRMAMKDGELVIWDSVHPCYTVFHEQTETFSSLWSEYHDDFRQFLHIY	219	0
CmR-015	MEKKITGYTTVDISQWHRKEHFEAFQSVAQCTYNQTVQLDITAFLKTVKKNKHKFYPAFIHILARLMNAHPEFRMAMKDGELVIWDSVHPCYTVFHEQTETFSSLWSEYHDDFRQFLHIY	219	0
CmR-014	MEKKITGYTTVDISQWHRKEHFEAFQSVAQCTYNQTVQLDITAFLKTVKKNKHKFYPAFIHILARLMNAHPEFRMAMKDGELVIWDSVHPCYTVFHEQTETFSSLWSEYHDDFRQFLHIY	219	0
CmR-010	MEKKITGYTTVDISQWHRKEHFEAFQSVAQCTYNQTVQLDITAFLKTVKKNKHKFYPAFIHILARLMNAHPEFRMAMKDGELVIWDSVHPCYTVFHEQTETFSSLWSEYHDDFRQFLHIY	219	0
CmR-004	MEKKITGYTTVDISQWHRKEHFEAFQSVAQCTYNQTVQLDITAFLKTVKKNKHKFYPAFIHILARLMNAHPEFRMAMKDGELVIWDSVHPCYTVFHEQTETFSSLWSEYHDDFRQFLHIY	219	0
CmR-011	MEKKITGYTTVDISQWHRKEHFEAFQSVAQCTYNQTVQLDITAFLKTVKKNKHKFYPAFIHILARLMNAHPEFRMAMKDGELVIWDSVHPCYTVFHEQTETFSSLWSEYHDDFRQFLHIY	219	0
CmR-002	MEKKITGYTTVDISQWHRKEHFEAFQSVAQCTYNQTVQLDITAFLKTVKKNKHKFYPAFIHILARLMNAHPEFRMAMKDGELVIWDSVHPCYTVFHEQTETFSSLWSEYHDDFRQFLHIY	219	0
CmR-012	MEKKITGYTTVDISQWHRKEHFEAFQSVAQCTYNQTVQLDITAFLKTVKKNKHKFYPAFIHILARLMNAHPEFRMAMKDGELVIWDSVHPCYTVFHEQTETFSSLWSEYHDDFRQFLHIY	219	0
CmR-001	MEKKITGYTTVDISQWHRKEHFEAFQSVAQCTYNQTVQLDITAFLKTVKKNKHKFYPAFIHILARLMNAHPEFRMAMKDGELVIWDSVHPCYTVFHEQTETFSSLWSEYHDDFRQFLHIY	219	0
CmR-005	MEKKITGYTTVDISQWHRKEHFEAFQSVAQCTYNQTVQLDITAFLKTVKKNKHKFYPAFIHILARLMNAHPELRMAMKDGELVIWDSVHPCYTVFHEQTETFSSLWSEYHDDFRQFLHIY	219	1
CmR-003	MEKKITGYTTVDISQWHRKEHFEAFQSVAQCTYNQTVQLDITAFLKTVKKNKHKFYPAFIHILARLMNAHPEFRMAMKDGELVIWDSVHPCYTVFHEQTETFSSLWSEYHDDFRQFLHIY	219	0
CmR-006	MEKKITGYTTVDISQWHRKEHFEAFQSVAQCTYNQTVQLDITAFLKTVKKNKHKFYPAFIHILARLMNAHPEFRMAMKDGELVIWDSVHPCYTVFHEQTETFSSLWSEYHDDFRQFLHIY	219	0
CmR-007	MEKKITGYTTVDISQWHRKEHFEAFQSVAQCTYNQTVQLDITAFLKTVKKNKHKFYPAFIHILARLMNAHPEFRMAMKDGELVIWDSVHPCYTVFHEQTETFSSLWSEYHDDFRQFLHIY	219	0
CmR-008	MEKKITGYTTVDISQWHRKEHFEAFQSVAQCTYNQTVQLDITAFLKTVKKNKHKFYPAFIHILARLMNAHPEFRMAMKDGELVIWDSVHPCYTVFHEQTETFSSLWSEYHDDFRQFLHIY	226	2
CmR-009corr	MEKKITGYTTVDISQWHRKEHFEAFQSVAQCTYNQTVQLDITAFLKTVKKNKHKFYPAFIHILARLMNAHPEFRMAMKDGELVIWDSVHPCYTVFHEQTETFSSLWSEYHDDFRQFLHIY	227	1
	:*		

CmR-013	SQDVACYGENLAYFPKGFIENMFFVSANPWVSFTSFDLNVANMDNFFAPVFTMGKYYTQGDKVLMPLAIQVHHAVCDGFHVGRMLNELQQYCDEWQGGA-------
CmR-016	SQDVACYGENLAYFPKGFIENMFFVSANPWVSFTSFDLNVANMDNFFAPVFTMGKYYTQGDKVLMPLAIQVHHAVCDGFHVGRMLNELQQYCDEWQGGA-------
CmR-015	SQDVACYGENLAYFPKGFIENMFFVSANPWVSFTSFDLNVANMDNFFAPVFTMGKYYTQGDKVLMPLAIQVHHAVCDGFHVGRMLNELQQYCDEWQGGA-------
CmR-014	SQDVACYGENLAYFPKGFIENMFFVSANPWVSFTSFDLNVANMDNFFAPVFTMGKYYTQGDKVLMPLAIQVHHAVCDGFHVGRMLNELQQYCDEWQGGA-------
CmR-010	SQDVACYGENLAYFPKGFIENMFFVSANPWVSFTSFDLNVANMDNFFAPVFTMGKYYTQGDKVLMPLAIQVHHAVCDGFHVGRMLNELQQYCDEWQGGA-------
CmR-004	SQDVACYGENLAYFPKGFIENMFFVSANPWVSFTSFDLNVANMDNFFAPVFTMGKYYTQGDKVLMPLAIQVHHAVCDGFHVGRMLNELQQYCDEWQGGA-------
CmR-011	SQDVACYGENLAYFPKGFIENMFFVSANPWVSFTSFDLNVANMDNFFAPVFTMGKYYTQGDKVLMPLAIQVHHAVCDGFHVGRMLNELQQYCDEWQGGA-------
CmR-002	SQDVACYGENLAYFPKGFIENMFFVSANPWVSFTSFDLNVANMDNFFAPVFTMGKYYTQGDKVLMPLAIQVHHAVCDGFHVGRMLNELQQYCDEWQGGA-------
CmR-012	SQDVACYGENLAYFPKGFIENMFFVSANPWVSFTSFDLNVANMDNFFAPVFTMGKYYTQGDKVLMPLAIQVHHAVCDGFHVGRMLNELQQYCDEWQGGA-------
CmR-001	SQDVACYGENLAYFPKGFIENMFFVSANPWVSFTSFDLNVANMDNFFAPVFTMGKYYTQGDKVLMPLAIQVHHAVCDGFHVGRMLNELQQYCDEWQGGA-------
CmR-005	SQDVACYGENLAYFPKGFIENMFFVSANPWVSFTSFDLNVANMDNFFAPVFTMGKYYTQGDKVLMPLAIQVHHAVCDGFHVGRMLNELQQYCDEWQGGA-------
CmR-003	SQDVACYGENLAYFPKGFIENMFFVSANPWVSFTSFDLNVANMDNFFAPVFTMGKYYTQGDKVLMPLAIQVHHAVCDGFHVGRMLNELQQYCDEWQGGA--------
CmR-006	SQDVACYGENLAYFPKGFIENMFFVSANPWVSFTSFDLNVANMDNFFAPVFTMGKYYTQGDKVLMPLAIQVHHAVCDGFHVGRMLNELQQYCDEWQGGA--------
CmR-007	SQDVACYGENLAYFPKGFIENMFFVSANPWVSFTSFDLNVANMDNFFAPVFTMGKYYTQGDKVLMPLAIQVHHAVCDGFHVGRMLNELQQYCDEWQGGA--------
CmR-008	SQDVACYGENLAYFPKGFIENMFFVSANPWVSFTSFDLNVANMDNFFAPVFTMGKYYTQGDKVLMPLAIQVHHAVCDGFHVGRMLNELQQYCDEWQAGRNLE-DPAY
CmR-009corr	SQDVACYGENLAYFPKGFIENMFFVSANPWVSFTSFDLNVANMDNFFAPVFTMGKYYTQGDKVLMPLAIQVHHAVCDGFHVGRMLNELQQYCDEWQGGRKRRVDPAY
	**.*

Key
ATGC = change in nucleotide relative to consensus sequence – synonymous
ATGC = change in nucleotide relative to consensus sequence – conservative/semi-conservative amino acid substitution
ATGC = change in nucleotide relative to consensus sequence – non-conservative amino acid substitution

Sequence Alignments

Name of Variant	# of Occur	Alignment	Size (bp)	# Var bp	Sources*
HygR-010	35	ATG---AAGAAGCCCGAACTCACCGCTACCAGCGTTGAAAAATTTCTCATCGAGAAGTTCGACAGTGTGAGCGACCTGATGCAGTTGTCGGAGGGCGAAGAGAGCCGAGCCTTCAGCTTC	1038	222	Pr
HygR-012	26	ATG---AAAAAGCCTGAACTCACCGCGACGTCTGTCGAGAAGTTTCTGATCGAAAAGTTCGACAGCGTCTCCGACCTGATGCAGCTCTCGGAGGGCGAAGAATCTCGTGCTTTCAGCTTC	1026	8	Ca
HygR-008	11	ATG---AAAAAGCCTGAACTCACCGCGACGTCTGTCGAGAAGTTTCTGATCGAAAAGTTCGACAGCGTCTCCGACCTGATGCAGCTCTCGGAGGGCGAAGAATCTCGTGCTTTCAGCTTC	1026	0	In,Cl,OB
HygR-005	7	---------AAGCCTGAACTCACCGCGACGTCTGTCGAGAAGTTTCTGATCGAAAAGTTCGACAGCGTCTCCGACCTGATGCAGCTCTCGGAGGGCGAAGAATCTCGTGCTTTCAGCTTC	1020	0/6d	Cl
HygR-002	4	ATG---AAAAAGCCTGAACTCACCGCGACGTCTGTCGAGAAGTTTCTGATCGAAAAGTTCGACAGCGTCTCCGACCTGATGCAGCTCTCGGAGGGCGAAGAATCTCGTGCTTTCAGCTTC	1038	13	Ag
HygR-011	4	---------AAGCCTGAACTCACCGCGACGTCTGTTGAGAAGTTTCTGATCGAAAAGTTCGACAGCGTCTCCGACCTGATGCAGCTCTCGGAGGGCGAAGAATCTCGTGCTTTCAGCTTC	1020	4/6d	No
HygR-004	3	ATGGGTAAAAAGCCTGAACTCACCGCGACGTCTGTCGAGAAGTTTCTGATCGAAAAGTTCGACAGCGTCTCCGACCTGATGCAGCTCTCGGAGGGCGAAGAATCTCGTGCTTTCAGCTTC	1029	0	16200533
HygR-007	3	------AAAAAGCCTGAACTCACCGCGACGTCTGTCGAGAAGTTTCTGATCGAAAAGTTCGACAGCGTCTCCGACCTGATGCAGCTCTCGGAGGGCGAAGAATCTCGTGCTTTCAGCTTC	1023	0/3d	In
HygR-001	2	ATG---AAAAAGCCTGAACTCACCGCGACGTCTGTCGAGAAGTTTCTGATCGAAAAGTTCGACAGCGTCTCCGACCTGATGCAGCTCTCGGAGGGCGAAGAATCTCGTGCTTTCAGCTTC	1026	1	Cl
hphMX6*	2	ATGGGTAAAAAGCCTGAACTCACCGCGACGTCTGTCGAGAAGTTTCTGATCGAAAAGTTCGACAGCGTCTCCGACCTGATGCAGCTCTCGGAGGGCGAAGAATCTCGTGCTTTCAGCTTC	1029	0	16200533
HygR-003	1	ATG---GGAAAGCCTGAGCGTACCGCGACGTCTGTCGAGAAGTTTCTGATCGAAAAGTTCGACAGCGTCTCCGACCTGATGCAGCTCTCGGAGGGCGAAGAATCTCGTGCTTTCAGCTTC	1026	6	In
HygR-006	1	ATG---AAAAAGCCTGAACTCACCGCGACGTCTGTCGAGAAGTTTCTGATCGAAAAGTTCGACAGCGTCTCCGACCTGATGCAGCTCTCGGAGGGCGAAGAATCTCGTGCTTTCAGCTTC	1026	5	10890530
HygR-009	1	ATG---AAAAAGCCTGAACTCACCGCGACGTCTGTTGAGAAGTTTCTGATCGAAAAGTTCGACAGCGTCTCCGACCTGATGCAGCTCTCGGAGGGCGAAGAATCTCGTGCTTTCAGCTTC	1026	4	Or
**HygR-013**	**1**	**ATG---AAAAAGCCTGAACTCACCGCGACGTCTGTCGAGAAGTTTCTGATCGAAAAGTTCGACAGCGTCTCCGACCTGATGCAGCTCTCGGAGGGCGAAGAATCTCGTGCTTTCAGCTTC**	**1023**	**1/3d**	**2194165**
HygR-013corr		ATG---AAAAAGCCTGAACTCACCGCGACGTCTGTCGAGAAGTTTCTGATCGAAAAGTTCGACAGCGTCTCCGACCTGATGCAGCTCTCGGAGGGCGAAGAATCTCGTGCTTTCAGCTTC	1023	1/3d	
		***** ** * ***** ** ** ** ** ***** *********** ** ************* * ************** ** ** *********			
HygR-010		GATGTCGGCGGACGCGGCTATGTACTGCGGGTGAATAGCTGCGCTGATGGCTTCTACAAAGACCGCTACGTGTACCGCCACTTCGCCAGCGCTGCACTACCCATCCCCGAAGTGTTGGAC			
HygR-012		GATGTAGGAGGGCGTGGATATGTCCTGCGGGTAAATAGCTGCGCCGATGGTTTCTACAAAGATCGTTATGTTTATCGGCACTTTGCATCGGCCGCGCTCCCGATTCCGGAAGTGCTTGAC			
HygR-008		GATGTAGGAGGGCGTGGATATGTCCTGCGGGTAAATAGCTGCGCCGATGGTTTCTACAAAGATCGTTATGTTTATCGGCACTTTGCATCGGCCGCGCTCCCGATTCCGGAAGTGCTTGAC			
HygR-005		GATGTAGGAGGGCGTGGATATGTCCTGCGGGTAAATAGCTGCGCCGATGGTTTCTACAAAGATCGTTATGTTTATCGGCACTTTGCATCGGCCGCGCTCCCGATTCCGGAAGTGCTTGAC			
HygR-002		GATGTAGGAGGGCGTGGATATGTCCTGCGGGTAAATAGCTGCGCCGATGGTTTCTACAAAGATCGTTATGTTTATCGGCACTTTGCATCGGCCGCGCTCCCGATTCCGGAAGTGCTTGAC			
HygR-011		GATGTAGGAGGGCGTGGATATGTCCTGCGGGTAAATAGCTGCGCCGATGGTTTCTACAAAGATCGTTATGTTTATCGGCACTTTGCATCGGCCGCGCTCCCGATTCCGGAAGTGCTTGAC			
HygR-004		GATGTAGGAGGGCGTGGATATGTCCTGCGGGTAAATAGCTGCGCCGATGGTTTCTACAAAGATCGTTATGTTTATCGGCACTTTGCATCGGCCGCGCTCCCGATTCCGGAAGTGCTTGAC			
HygR-007		GATGTAGGAGGGCGTGGATATGTCCTGCGGGTAAATAGCTGCGCCGATGGTTTCTACAAAGATCGTTATGTTTATCGGCACTTTGCATCGGCCGCGCTCCCGATTCCGGAAGTGCTTGAC			
HygR-001		GATGTAGGAGGGCGTGGATATGTCCTGCGGGTAAATAGCTGCGCCGATGGTTTCTACAAAGATCGTTATGTTTATCGGCACTTTGCATCGGCCGCGCTCCCGATTCCGGAAGTGCTTGAC			
hphMX6		GATGTAGGAGGGCGTGGATATGTCCTGCGGGTAAATAGCTGCGCCGATGGTTTCTACAAAGATCGTTATGTTTATCGGCACTTTGCATCGGCCGCGCTCCCGATTCCGGAAGTGCTTGAC			
HygR-003		GATGTAGGAGGGCGTGGATATGTCCTGCGGGTAAATAGCTGCGGCGATGGTTTCTACAAAGATCGTTATGTTTATCGGCACTTTGCATCGGCCGCGCTCCCGATTCCGGAAGTGCTTGAC			
HygR-006		GATGTAGGAGGGCGTGGATATGTCCTGCGGGTAAATAGCTGCGCCGATGGTTTCTACAAAGATCGTTATGTTTATCGGCACTTTGCATCGGCCGCGCTCCCGATTCCGGAAGTGCTTGAC			
HygR-009		GATGTAGGAGGGCGTGGATATGTCCTGCGGGTAAATAGCTGCGCCGATGGTTTCTACAAAGATCGTTATGTTTATCGGCACTTTGCATCGGCCGCGCTCCCGATTCCGGAAGTGCTTGAC			
**HygR-013**		**GATGTAGGAGGGCGTGGATATGTCCTGCGGGTAAATAGCTGCGCCGATGGTTTCTACAAAGATCGTTATGTTTATCGGCACTTTGCATCGGCCGCGCTCCCGATTCCGGAAGTGCTTGAC**			
HygR-013corr		GATGTAGGAGGGCGTGGATATGTCCTGCGGGTAAATAGCTGCGCCGATGGTTTCTACAAAGATCGTTATGTTTATCGGCACTTTGCATCGGCCGCGCTCCCGATTCCGGAAGTGCTTGAC			
		***** ** ** ** ** ***** ******** ********** ***** *********** ** ** ** ** ** ***** ** ** ** ** ** ** ** ****** * ***			
HygR-010		ATCGGCGAGTTCAGCGAGAGCCTGACATACTGCATCAGTAGACGCGCCCAAGGCGTTACTCTCCAAGACCTCCCCGAAACAGAGCTGCCTGCTGTGTTACAGCCTGTCGCCGAAGCTATG			
HygR-012		ATTGGGGAGTTTAGCGAGAGCCTGACCTATTGCATCTCCCGCCGTGCACAGGGTGTCACGTTGCAAGACCTGCCTGAAACCGAACTGCCCGCTGTTCTACAACCGGTCGCGGAGGCTATG			
HygR-008		ATTGGGGAATTCAGCGAGAGCCTGACCTATTGCATCTCCCGCCGTGCACAGGGTGTCACGTTGCAAGACCTGCCTGAAACCGAACTGCCCGCTGTTCTGCAGCCGGTCGCGGAGGCCATG			
HygR-005		ATTGGGGAATTCAGCGAGAGCCTGACCTATTGCATCTCCCGCCGTGCACAGGGTGTCACGTTGCAAGACCTGCCTGAAACCGAACTGCCCGCTGTTCTGCAGCCGGTCGCGGAGGCCATG			
HygR-002		ATTGGGGAATTCAGCGAGAGCCTGACCTATTGCATCTCCCGCCGTGCACAGGGTGTCACGTTGCAAGACCTGCCTGAAACCGAACTGCCCGCTGTTCTGCAGCCGGTCGCGGAGGCCATG			
HygR-011		ATTGGGGAATTTAGCGAGAGCCTGACCTATTGCATCTCCCGCCGTGCACAGGGTGTCACGTTGCAAGACCTGCCTGAAACCGAACTGCCCGCTGTTCTGCAACCGGTCGCGGAGGCTATG			
HygR-004		ATTGGGGAATTCAGCGAGAGCCTGACCTATTGCATCTCCCGCCGTGCACAGGGTGTCACGTTGCAAGACCTGCCTGAAACCGAACTGCCCGCTGTTCTGCAGCCGGTCGCGGAGGCCATG			
HygR-007		ATTGGGGAATTCAGCGAGAGCCTGACCTATTGCATCTCCCGCCGTGCACAGGGTGTCACGTTGCAAGACCTGCCTGAAACCGAACTGCCCGCTGTTCTGCAGCCGGTCGCGGAGGCCATG			
HygR-001		ATTGGGGAGTTCAGCGAGAGCCTGACCTATTGCATCTCCCGCCGTGCACAGGGTGTCACGTTGCAAGACCTGCCTGAAACCGAACTGCCCGCTGTTCTGCAGCCGGTCGCGGAGGCCATG			
hphMX6		ATTGGGGAATTCAGCGAGAGCCTGACCTATTGCATCTCCCGCCGTGCACAGGGTGTCACGTTGCAAGACCTGCCTGAAACCGAACTGCCCGCTGTTCTGCAGCCGGTCGCGGAGGCCATG			
HygR-003		ATTGGGGAATTCAGCGAGAGCCTGACCTATTGCATCTCCCGCCGTGCACAGGGTGTCACGTTGCAAGACCTGCCTGAAACCGAACTGCCCGCTGTTCTGCAGCCGGTCGCGGAGGCCATG			
HygR-006		ATTGGGGAGTTCAGCGAGAGCCTGACCTATTGCATCTCCCGCCGTGCACAGGGTGTCACGTTGCAAGACCTGCCTGAAACCGAACTGCCCGCTGTTCTTCAGCCGGTCGCGGAGGCTATG			
HygR-009		ATTGGGGAATTTAGCGAGAGCCTGACCTATTGCATCTCCCGCCGTGCACAGGGTGTCACGTTGCAAGACCTGCCTGAAACCGAACTGCCCGCTGTTCTGCAACCGGTCGCGGAGGCCATG			
**HygR-013**		**ATTGGGGAGTTCAGCGAGAGCCTGACCTATTGCATCTCCCGCCGTGCACAGGGTGTCACGTTGCAAGACCTGCCTGAAACCGAACTGCCCGCTGTTCTGCAGCCGGTCGCGGAGGCCATG**			
HygR-013corr		ATTGGGGAGTTCAGCGAGAGCCTGACCTATTGCATCTCCCGCCGTGCACAGGGTGTCACGTTGCAAGACCTGCCTGAAACCGAACTGCCCGCTGTTCTGCAGCCGGTCGCGGAGGCCATG			
		** ** ** ** ************** ** ****** * ** ** ** ** ** ** ** * ******** ** ***** ** ***** ***** * ** ** ***** ** ** ***			
HygR-010		GATGCTATTGCCGCCGCCGACCTCAGTCAAACCAGCGGCTTCGGCCCATTCGGGCCCCAAGGCATCGGCCAGTACACAACCTGGCGGGATTTCATTTGCGCCATTGCTGATCCCCATGTC			
HygR-012		GATGCGATCGCTGCGGCCGATCTTAGCCAGACGAGCGGGTTCGGCCCATTCGGACCGCAAGGAATCGGTCAATACACTACATGGCGTGATTTCATATGCGCGATTGCTGATCCCCATGTG			
HygR-008		GATGCGATCGCTGCGGCCGATCTTAGCCAGACGAGCGGGTTCGGCCCATTCGGACCGCAAGGAATCGGTCAATACACTACATGGCGTGATTTCATATGCGCGATTGCTGATCCCCATGTG			
HygR-005		GATGCGATCGCTGCGGCCGATCTTAGCCAGACGAGCGGGTTCGGCCCATTCGGACCGCAAGGAATCGGTCAATACACTACATGGCGTGATTTCATATGCGCGATTGCTGATCCCCATGTG			
HygR-002		GATGCGATCGCTGCGGCCGATCTTAGCCAGACGAGCGGGTTCGGCCCATTCGGACCGCAAGGAATCGGTCAATACACTACATGGCGTGATTTCATATGCGCGATTGCTGATCCCCATGTG			
HygR-011		GATGCGATCGCTGCGGCCGATCTTAGCCAGACGAGCGGGTTCGGCCCATTCGGACCGCAAGGAATCGGTCAATACACTACATGGCGTGATTTCATATGCGCGATTGCTGATCCCCATGTG			
HygR-004		GATGCGATCGCTGCGGCCGATCTTAGCCAGACGAGCGGGTTCGGCCCATTCGGACCGCAAGGAATCGGTCAATACACTACATGGCGTGATTTCATATGCGCGATTGCTGATCCCCATGTG			
HygR-007		GATGCGATCGCTGCGGCCGATCTTAGCCAGACGAGCGGGTTCGGCCCATTCGGACCGCAAGGAATCGGTCAATACACTACATGGCGTGATTTCATATGCGCGATTGCTGATCCCCATGTG			
HygR-001		GATGCGATCGCTGCGGCCGATCTTAGCCAGACGAGCGGGTTCGGCCCATTCGGACCGCAAGGAATCGGTCAATACACTACATGGCGTGATTTCATATGCGCGATTGCTGATCCCCATGTG			
hphMX6		GATGCGATCGCTGCGGCCGATCTTAGCCAGACGAGCGGGTTCGGCCCATTCGGACCGCAAGGAATCGGTCAATACACTACATGGCGTGATTTCATATGCGCGATTGCTGATCCCCATGTG			
HygR-003		GATGCGATCGCTGCGGCCGATCTTAGCCAGACGAGCGGGTTCGGCCCATTCGGACCGCAAGGAATCGGTCAATACACTACATGGCGTGATTTCATATGCGCGATTGCTGATCCCCATGTG			
HygR-006		GATGCGATCGCTGCGGCCGATCTTAGCCAGACGAGCGGGTTCGGCCCATTCGGACCGCAAGGAATCGGTCAATACACTACATGGCGTGATTTCATATGCGCGATTGCTGATCCCCATGTG			
HygR-009		GATGCAATCGCTGCGGCCGATCTTAGCCAGACGAGCGGGTTCGGCCCATTCGGACCGCAAGGAATCGGTCAATACACTACATGGCGTGATTTCATATGCGCGATTGCTGATCCCCATGTG			
**HygR-013**		**GATGCGATCGCTGCGGCCGATCTTAGCCAGACGAGCGGGTTCGGCCCATTCGGACCGCAAGGAATCGGTCAATACACTACATGGCGTGATTTCATATGCGCGATTGCTGATCCCCATGTG**			
HygR-013corr		GATGCGATCGCTGCGGCCGATCTTAGCCAGACGAGCGGGTTCGGCCCATTCGGACCGCAAGGAATCGGTCAATACACTACATGGCGTGATTTCATATGCGCGATTGCTGATCCCCATGTG			
		***** ** ** ** ***** ** ** ** ** ***** ************** ** ***** ***** ** ***** ** ***** ******** ***** *****************			

Sequence Alignments

```
HygR-010        TACCACTGGCAGACCGTGATGGACGACACCGTGTCCGCCAGCGTAGCTCAAGCCCTGGACGAACTGATGCTGTGGGCCGAAGACTGTCCCGAGGTGCGCCACCTCGTCCATGCCGACTTC
HygR-012        TATCACTGGCAAACTGTGATGGACGACACCGTCAGTGCGTCCGTCGCGCAGGCTCTCGATGAGCTGATGCTTTGGGCCGAGGACTGCCCCGAAGTCCGGCACCTCGTGCACGCGGATTTC
HygR-008        TATCACTGGCAAACTGTGATGGACGACACCGTCAGTGCGTCCGTCGCGCAGGCTCTCGATGAGCTGATGCTTTGGGCCGAGGACTGCCCCGAAGTCCGGCACCTCGTGCACGCGGATTTC
HygR-005        TATCACTGGCAAACTGTGATGGACGACACCGTCAGTGCGTCCGTCGCGCAGGCTCTCGATGAGCTGATGCTTTGGGCCGAGGACTGCCCCGAAGTCCGGCACCTCGTGCACGCGGATTTC
HygR-002        TATCACTGGCAAACTGTGATGGACGACACCGTCAGTGCGTCCGTCGCGCAGGCTCTCGATGAGCTGATGCTTTGGGCCGAGGACTGCCCCGAAGTCCGGCACCTCGTGCACGCGGATTTC
HygR-011        TATCACTGGCAAACTGTGATGGACGACACCGTCAGTGCGTCCGTCGCGCAGGCTCTCGATGAGCTGATGCTTTGGGCCGAGGACTGCCCCGAAGTCCGGCACCTCGTGCACGCGGATTTC
HygR-004        TATCACTGGCAAACTGTGATGGACGACACCGTCAGTGCGTCCGTCGCGCAGGCTCTCGATGAGCTGATGCTTTGGGCCGAGGACTGCCCCGAAGTCCGGCACCTCGTGCACGCGGATTTC
HygR-007        TATCACTGGCAAACTGTGATGGACGACACCGTCAGTGCGTCCGTCGCGCAGGCTCTCGATGAGCTGATGCTTTGGGCCGAGGACTGCCCCGAAGTCCGGCACCTCGTGCACGCGGATTTC
HygR-001        TATCACTGGCAAACTGTGATGGACGACACCGTCAGTGCGTCCGTCGCGCAGGCTCTCGATGAGCTGATGCTTTGGGCCGAGGACTGCCCCGAAGTCCGGCACCTCGTGCACGCGGATTTC
hphMX6          TATCACTGGCAAACTGTGATGGACGACACCGTCAGTGCGTCCGTCGCGCAGGCTCTCGATGAGCTGATGCTTTGGGCCGAGGACTGCCCCGAAGTCCGGCACCTCGTGCACGCGGATTTC
HygR-003        TATCACTGGCAAACTGTGATGGACGACACCGTCAGTGCGTCCGTCGCGCAGGCTCTCGATGAGCTGATGCTTTGGGCCGAGGACTGCCCCGAAGTCCGGCACCTCGTGCACGCGGATTTC
HygR-006        TATCACTGGCAAACTGTGATGGACGACACCGTCAGTGCGTCCGTCGCGCAGGCTCTCGATGAGCTGATGCTTTGGGCCGAGGACTGCCCCGAAGTCCGGCACCTCGTGCACGCGGATTTC
HygR-009        TATCACTGGCAAACTGTGATGGACGACACCGTCAGTGCGTCCGTCGCGCAGGCTCTCGATGAGCTGATGCTTTGGGCCGAGGACTGCCCCGAAGTCCGGCACCTCGTGCACGCGGATTTC
HygR-013        TATCACTGGCAAACTGTGATGGACGACACCGTCAGTGCGTCCGTCGCGCAGGCTCTCGATGAGCTGATGCTTTGGGCCGAGGACTGCCCCGAAGTCCGGCACCTCGTGCACGCGGATTTC
HygR-013corr    TATCACTGGCAAACTGTGATGGACGACACCGTCAGTGCGTCCGTCGCGCAGGCTCTCGATGAGCTGATGCTTTGGGCCGAGGACTGCCCCGAAGTCCGGCACCTCGTGCACGCGGATTTC
                ** ******** ** *****************    **   *** ** ** ** ** ** ** ******** ******** ***** ***** ** ** ******** ** ** ** ***

HygR-010        GGCAGCAACAACGTCCTGACCGACAACGGCCGCATCACCGCCGTAATCGACTGGTCCGAAGCTATGTTCGGGGACAGTCAGTACGAGGTGGCCAACATCTTCTTCTGGCGGCCCTGGCTG
HygR-012        GGCTCCAACAATGTCCTGACGGACAATGGCCGCATAACAGCGGTCATTGACTGGAGCGAGGCGATGTTCGGGGATTCCCAATACGAGGTCGCCAACATCTTCTTCTGGAGGCCGTGGTTG
HygR-008        GGCTCCAACAATGTCCTGACGGACAATGGCCGCATAACAGCGGTCATTGACTGGAGCGAGGCGATGTTCGGGGATTCCCAATACGAGGTCGCCAACATCTTCTTCTGGAGGCCGTGGTTG
HygR-005        GGCTCCAACAATGTCCTGACGGACAATGGCCGCATAACAGCGGTCATTGACTGGAGCGAGGCGATGTTCGGGGATTCCCAATACGAGGTCGCCAACATCTTCTTCTGGAGGCCGTGGTTG
HygR-002        GGCTCCAACAATGTCCTGACGGACAATGGCCGCATAACAGCGGTCATTGACTGGAGCGAGGCGATGTTCGGGGATTCCCAATACGAGGTCGCCAACATCTTCTTCTGGAGGCCGTGGTTG
HygR-011        GGCTCCAACAATGTCCTGACGGACAATGGCCGCATAACAGCGGTCATTGACTGGAGCGAGGCGATGTTCGGGGATTCCCAATACGAGGTCGCCAACATCTTCTTCTGGAGGCCGTGGTTG
HygR-004        GGCTCCAACAATGTCCTGACGGACAATGGCCGCATAACAGCGGTCATTGACTGGAGCGAGGCGATGTTCGGGGATTCCCAATACGAGGTCGCCAACATCTTCTTCTGGAGGCCGTGGTTG
HygR-007        GGCTCCAACAATGTCCTGACGGACAATGGCCGCATAACAGCGGTCATTGACTGGAGCGAGGCGATGTTCGGGGATTCCCAATACGAGGTCGCCAACATCTTCTTCTGGAGGCCGTGGTTG
HygR-001        GGCTCCAACAATGTCCTGACGGACAATGGCCGCATAACAGCGGTCATTGACTGGAGCGAGGCGATGTTCGGGGATTCCCAATACGAGGTCGCCAACATCTTCTTCTGGAGGCCGTGGTTG
hphMX6          GGCTCCAACAATGTCCTGACGGACAATGGCCGCATAACAGCGGTCATTGACTGGAGCGAGGCGATGTTCGGGGATTCCCAATACGAGGTCGCCAACATCTTCTTCTGGAGGCCGTGGTTG
HygR-003        GGCTCCAACAATGTCCTGACGGACAATGGCCGCATAACAGCGGTCATTGACTGGAGCGAGGCGATGTTCGGGGATTCCCAATACGAGGTCGCCAACATCTTCTTCTGGAGGCCGTGGTTG
HygR-006        GGCTCCAACAATGTCCTGACGGACAATGGCCGCATAACAGCGGTCATTGACTGGAGCGAGGCGATGTTCGGGGATTCCCAATACGAGGTCGCCAACATCTTCTTCTGGAGGCCGTGGTTG
HygR-009        GGCTCCAACAATGTCCTGACGGACAATGGCCGCATAACAGCGGTCATTGACTGGAGCGAGGCGATGTTCGGGGATTCCCAATACGAGGTCGCCAACATCTTCTTCTGGAGGCCGTGGTTG
HygR-013        GGCTCCAACAATGTCCTGACGGACAATGGCCGCATAACAGCGGTCATTGACTGGAGCGAGGCGATGTTCGGGGATTCCCAATACGAGGTCGCCAACATCTTCTTCTGGAGGCCGTGGTTG
HygR-013corr    GGCTCCAACAATGTCCTGACGGACAATGGCCGCATAACAGCGGTCATTGACTGGAGCGAGGCGATGTTCGGGGATTCCCAATACGAGGTCGCCAACATCTTCTTCTGGAGGCCGTGGTTG
                ***  ****** ******** ***** ******** ** ** ** ** ******  *** ** ***********    ** ******** ****************** **** *** **

HygR-010        GCTTGCATGGAGCAGCAGACTCGCTACTTCGAGCGCCGGCATCCCGAGCTGGCCGGCAGCCCTCGTCTGCGAGCCTACATGCTGCGCATCGGCCTGGATCAGCTCTACCAGAGCCTCGTG
HygR-012        GCTTGTATGGAGCAGCAGACGCGCTACTTCGAGCGGAGGCATCCGGAGCTTGCAGGATCGCCACGACTCCGGGCGTATATGCTCCGCATTGGTCTTGACCAACTCTATCAGAGCTTGGTT
HygR-008        GCTTGTATGGAGCAGCAGACGCGCTACTTCGAGCGGAGGCATCCGGAGCTTGCAGGATCGCCGCGGCTCCGGGCGTATATGCTCCGCATTGGTCTTGACCAACTCTATCAGAGCTTGGTT
HygR-005        GCTTGTATGGAGCAGCAGACGCGCTACTTCGAGCGGAGGCATCCGGAGCTTGCAGGATCGCCGCGGCTCCGGGCGTATATGCTCCGCATTGGTCTTGACCAACTCTATCAGAGCTTGGTT
HygR-002        GCTTGTATGGAGCAGCAGACGCGCTACTTCGAGCGGAGGCATCCGGAGCTTGCAGGATCGCCGCGGCTCCGGGCGTATATGCTCCGCATTGGTCTTGACCAACTCTATCAGAGCTTGGTT
HygR-011        GCTTGTATGGAGCAGCAGACGCGCTACTTCGAGCGGAGGCATCCGGAGCTTGCAGGATCGCCGCGGCTCCGGGCGTATATGCTCCGCATTGGTCTTGACCAACTCTATCAGAGCTTGGTT
HygR-004        GCTTGTATGGAGCAGCAGACGCGCTACTTCGAGCGGAGGCATCCGGAGCTTGCAGGATCGCCGCGGCTCCGGGCGTATATGCTCCGCATTGGTCTTGACCAACTCTATCAGAGCTTGGTT
HygR-007        GCTTGTATGGAGCAGCAGACGCGCTACTTCGAGCGGAGGCATCCGGAGCTTGCAGGATCGCCGCGGCTCCGGGCGTATATGCTCCGCATTGGTCTTGACCAACTCTATCAGAGCTTGGTT
HygR-001        GCTTGTATGGAGCAGCAGACGCGCTACTTCGAGCGGAGGCATCCGGAGCTTGCAGGATCGCCGCGGCTCCGGGCGTATATGCTCCGCATTGGTCTTGACCAACTCTATCAGAGCTTGGTT
hphMX6          GCTTGTATGGAGCAGCAGACGCGCTACTTCGAGCGGAGGCATCCGGAGCTTGCAGGATCGCCGCGGCTCCGGGCGTATATGCTCCGCATTGGTCTTGACCAACTCTATCAGAGCTTGGTT
HygR-003        GCTTGTATGGAGCAGCAGACGCGCTACTTCGAGCGGAGGCATCCGGAGCTTGCAGGATCGCCGCGGCTCCGGGCGTATATGCTCCGCATTGGTCTTGACCAACTCTATCAGAGCTTGGTT
HygR-006        GCTTGTATGGAGCAGCAGACGCGCTACTTCGAGCGGAGGCATCCGGAGCTTGCAGGATCGCCACGCCTCCGGGCGTATATGCTCCGCATTGGTCTTGACCAACTCTATCAGAGCTTGGTT
HygR-009        GCTTGTATGGAGCAGCAGACGCGCTACTTCGAGCGGAGGCATCCGGAGCTTGCAGGATCGCCGCGGCTCCGGGCGTATATGCTCCGCATTGGTCTTGACCAACTCTATCAGAGCTTGGTT
HygR-013        GCTTGTATGGAGCAGCAGACGCGCTACTTCGAGCGGAGGCATCCGGAGCTTGCAGGATCGCCGCGGCTCCGGGCGTATATGCTCCGCATTGGTCTTGACCAACTCTATCAGAGCTTGGTT
HygR-013corr    GCTTGTATGGAGCAGCAGACGCGCTACTTCGAGCGGAGGCATCCGGAGCTTGCAGGATCGCCGCGGCTCCGGGCGTATATGCTCCGCATTGGTCTTGACCAACTCTATCAGAGCTTGGTT
                ***** ************** **************  ******* ***** ** **    ** ** ** ** ** ** ***** ***** ** ** ** ** ***** ****** * **

HygR-010        GACGGCAACTTCGACGATGCTGCCTGGGCTCAAGGCCGCTGCGATGCCATCGTCCGCAGCGGGGCCGGCACCGTCGGTCGCACACAAATCGCTCGCCGGAGCGCAGCCGTATGGACCGAC
HygR-012        GACGGCAATTTCGATGATGCAGCTTGGGCGCAGGGTCGATGCGACGCAATCGTCCGATCCGGAGCCGGGACTGTCGGGCGTACACAAATCGCCCGCAGAAGCGCGGCCGTCTGGACCGAT
HygR-008        GACGGCAATTTCGATGATGCAGCTTGGGCGCAGGGTCGATGCGACGCAATCGTCCGATCCGGAGCCGGGACTGTCGGGCGTACACAAATCGCCCGCAGAAGCGCGGCCGTCTGGACCGAT
HygR-005        GACGGCAATTTCGATGATGCAGCTTGGGCGCAGGGTCGATGCGACGCAATCGTCCGATCCGGAGCCGGGACTGTCGGGCGTACACAAATCGCCCGCAGAAGCGCGGCCGTCTGGACCGAT
HygR-002        GACGGCAATTTCGATGATGCAGCTTGGGCGCAGGGTCGATGCGACGCAATCGTCCGATCCGGAGCCGGGACTGTCGGGCGTACACAAATCGCCCGCAGAAGCGCGGCCGTCTGGACCGAT
HygR-011        GACGGCAATTTCGATGATGCAGCTTGGGCGCAGGGTCGATGCGACGCAATCGTCCGATCCGGAGCCGGGACTGTCGGGCGTACACAAATCGCCCGCAGAAGCGCGGCCGTCTGGACCGAT
HygR-004        GACGGCAATTTCGATGATGCAGCTTGGGCGCAGGGTCGATGCGACGCAATCGTCCGATCCGGAGCCGGGACTGTCGGGCGTACACAAATCGCCCGCAGAAGCGCGGCCGTCTGGACCGAT
HygR-007        GACGGCAATTTCGATGATGCAGCTTGGGCGCAGGGTCGATGCGACGCAATCGTCCGATCCGGAGCCGGGACTGTCGGGCGTACACAAATCGCCCGCAGAAGCGCGGCCGTCTGGACCGAT
HygR-001        GACGGCAATTTCGATGATGCAGCTTGGGCGCAGGGTCGATGCGACGCAATCGTCCGATCCGGAGCCGGGACTGTCGGGCGTACACAAATCGCCCGCAGAAGCGCGGCCGTCTGGACCGAT
hphMX6          GACGGCAATTTCGATGATGCAGCTTGGGCGCAGGGTCGATGCGACGCAATCGTCCGATCCGGAGCCGGGACTGTCGGGCGTACACAAATCGCCCGCAGAAGCGCGGCCGTCTGGACCGAT
HygR-003        GACGGCAATTTCGATGATGCAGCTTGGGCGCAGGGTCGATGCGACGCAATCGTCCGATCCGGAGCCGGGACTGTCGGGCGTACACAAATCGCCCGCAGAAGCGCGGCCGTCTGGACCGAT
HygR-006        GACGGCAATTTCGATGATGCAGCTTGGGCGCAGGGTCGATGCGACGCAATCGTCCGATCCGGAGCCGGGACTGTCGGGCGTACACAAATCGCCCGCAGAAGCGCGGCCGTCTGGACCGAT
HygR-009        GACGGCAATTTCGATGATGCAGCTTGGGCGCAGGGTCGATGCGACGCAATCGTCCGATCCGGAGCCGGGACTGTCGGGCGTACACAAATCGCCCGCAGAAGCGCGGCCGTCTGGACCGAT
HygR-013        GACGGCAATTTCGATGATGCAGCTTGGGCGCAGGGTCGATGCGACGCAATCGTCCGATCCGGAGCCGGGACTGTCGGGCGTACACAAATCGCCCGCAGAAGCGCGGCCGTCTGGACCGAT
HygR-013corr    GACGGCAATTTCGATGATGCAGCTTGGGCGCAGGGTCGATGCGACGCAATCGTCCGATCCGGAGCCGGGACTGTCGGGCGTACACAAATCGCCCGCAGAAGCGCGGCCGTCTGGACCGAT
                ******** ***** ***** ** ***** ** ** ** ***** ** ********   *** ***** ** ***** ** *********** *** * ***** ***** ********
```

Sequence Alignments

```
HygR-010        GGCTGCGTCGAGGTGCTGGCCGACAGCGGCAACCGCCGGCCCAGTACACGAACCGCGCGCTAAGGAGGTAGGTCGAGTTTAA
HygR-012        GGCTGTGTAGAAGTACTCGCCGATAGTGGAAACCGACGCCCCAGCACTCGTCCGAGGGCAAAGAAATAG------------
HygR-008        GGCTGTGTAGAAGTACTCGCCGATAGTGGAAACCGACGCCCCAGCACTCGTCCGAGGGCAAAGGAATAG------------
HygR-005        GGCTGTGTAGAAGTACTCGCCGATAGTGGAAACCGACGCCCCAGCACTCGTCCGAGGGCAAAGGAATAG------------
HygR-002        GGCTGTGTAGAAGTACTCGCCGATAGTGGAAACCGACGCCCCAGCACTCGTCCGGATCGGGAGATGGGGGAGGCTAACTGA
HygR-011        GGCTGTGTAGAAGTACTCGCCGATAGTGGAAACCGACGCCCCAGCACTCGTCCGAGGGCAAAGGAATAG------------
HygR-004        GGCTGTGTAGAAGTACTCGCCGATAGTGGAAACCGACGCCCCAGCACTCGTCCGAGGGCAAAGGAATAA------------
HygR-007        GGCTGTGTAGAAGTACTCGCCGATAGTGGAAACCGACGCCCCAGCACTCGTCCGAGGGCAAAGGAATAG------------
HygR-001        GGCTGTGTAGAAGTACTCGCCGATAGTGGAAACCGACGCCCCAGCACTCGTCCGAGGGCAAAGGAATAG------------
hphMX6          GGCTGTGTAGAAGTACTCGCCGATAGTGGAAACCGACGCCCCAGCACTCGTCCGAGGGCAAAGGAATAA------------
HygR-003        GGCTGTGTAGAAGTACTCGCCGATAGTGGAAACCGACGCCCCAGCACTCGTCCGAGGGCAAAGGAATAG------------
HygR-006        GGCTGTGTAGAAGTACTCGCCGATAGTGGAAACCGACGCCCCAGCACTCGTCCGAGGGCAAAGGAATAG------------
HygR-009        GGCTGTGTAGAAGTACTCGCCGATAGTGGAAACCGACGCCCCAGCACTCGTCCGAGGGCAAAGGAATAG------------
HygR-013        GGCTGTGTAGAAGTACTCGCCGATAGTGGAAACCGACGCCCCAGCACTCGTCCGAGGGCAAAGGAA---------------
HygR-013corr    GGCTGTGTAGAAGTACTCGCCGATAGTGGAAACCGACGCCCCAGCACTCGTCCGAGGGCAAAGGAATATCGATAA------------
                ***** ** ** ** ** ***** ** ** ***** ** ***** ** ** ***       **
```

HygR gene is *aph(4)-Ia* (aminoglycoside phosphotransferase) from *E. coli*
hphMX6 is identical to HygR-004 except it includes the *Ashbya gossypii TEF1* promoter and terminator, which have been trimmed out of the sequence shown
Sequences in bold are incorrectly annotated
HygR-013 missing STOP codon. Corrected sequence still unique variant

```
Name of         Alignment of Translated Protein                                                                                                                  Size    # Var
Variant                                                                                                                                                          (aa)    aa
HygR-010        -MKKPELTATSVEKFLIEKFDSVSDLMQLSEGEESRAFSFDVGGRGYVLRVNSCADGFYKDRYVYRHFASAALPIPEVLDIGEFSESLTYCISRRAQGVTLQDLPETELPAVLQPVAEAM                               341     0
HygR-012        -MKKPELTATSVEKFLIEKFDSVSDLMQLSEGEESRAFSFDVGGRGYVLRVNSCADGFYKDRYVYRHFASAALPIPEVLDIGEFSESLTYCISRRAQGVTLQDLPETELPAVLQPVAEAM                               345     1
HygR-008        -MKKPELTATSVEKFLIEKFDSVSDLMQLSEGEESRAFSFDVGGRGYVLRVNSCADGFYKDRYVYRHFASAALPIPEVLDIGEFSESLTYCISRRAQGVTLQDLPETELPAVLQPVAEAM                               341     0
HygR-005        ---KPELTATSVEKFLIEKFDSVSDLMQLSEGEESRAFSFDVGGRGYVLRVNSCADGFYKDRYVYRHFASAALPIPEVLDIGEFSESLTYCISRRAQGVTLQDLPETELPAVLQPVAEAM                               342     2
HygR-002        -MKKPELTATSVEKFLIEKFDSVSDLMQLSEGEESRAFSFDVGGRGYVLRVNSCADGFYKDRYVYRHFASAALPIPEVLDIGEFSESLTYCISRRAQGVTLQDLPETELPAVLQPVAEAM                               339     4
HygR-011        ---KPELTATSVEKFLIEKFDSVSDLMQLSEGEESRAFSFDVGGRGYVLRVNSCADGFYKDRYVYRHFASAALPIPEVLDIGEFSESLTYCISRRAQGVTLQDLPETELPAVLQPVAEAM                               341     2
HygR-004        MGKKPELTATSVEKFLIEKFDSVSDLMQLSEGEESRAFSFDVGGRGYVLRVNSCADGFYKDRYVYRHFASAALPIPEVLDIGEFSESLTYCISRRAQGVTLQDLPETELPAVLQPVAEAM                               340     0
HygR-007        --KKPELTATSVEKFLIEKFDSVSDLMQLSEGEESRAFSFDVGGRGYVLRVNSCADGFYKDRYVYRHFASAALPIPEVLDIGEFSESLTYCISRRAQGVTLQDLPETELPAVLQPVAEAM                               341     1
HygR-001        -MKKPELTATSVEKFLIEKFDSVSDLMQLSEGEESRAFSFDVGGRGYVLRVNSCADGFYKDRYVYRHFASAALPIPEVLDIGEFSESLTYCISRRAQGVTLQDLPETELPAVLQPVAEAM                               341     0
hphMX6          MGKKPELTATSVEKFLIEKFDSVSDLMQLSEGEESRAFSFDVGGRGYVLRVNSCADGFYKDRYVYRHFASAALPIPEVLDIGEFSESLTYCISRRAQGVTLQDLPETELPAVLQPVAEAM                               345     0
HygR-003        MG-KPERTATSVEKFLIEKFDSVSDLMQLSEGEESRAFSFDVGGRGYVLRVNSCGDGFYKDRYVYRHFASAALPIPEVLDIGEFSESLTYCISRRAQGVTLQDLPETELPAVLQPVAEAM                               339     3
HygR-006        -MKKPELTATSVEKFLIEKFDSVSDLMQLSEGEESRAFSFDVGGRGYVLRVNSCADGFYKDRYVYRHFASAALPIPEVLDIGEFSESLTYCISRRAQGVTLQDLPETELPAVLQPVAEAM                               341     0
HygR-009        -MKKPELTATSVEKFLIEKFDSVSDLMQLSEGEESRAFSFDVGGRGYVLRVNSCADGFYKDRYVYRHFASAALPIPEVLDIGEFSESLTYCISRRAQGVTLQDLPETELPAVLQPVAEAM                               341     0
HygR-013corr    -MKKPELTATSVEKFLIEKFDSVSDLMQLSEGEESRAFSFDVGGRGYVLRVNSCADGFYKDRYVYRHFASAALPIPEVLDIGEFSESLTYCISRRAQGVTLQDLPETELPAVLQPVAEAM                               342     0
                   *** ***************************************************.*****************************************************************

HygR-010        DAIAAADLSQTSGFGPFGPQGIGQYTTWRDFICAIADPHVYHWQTVMDDTVSASVAQALDELMLWAEDCPEVRHLVHADFGSNNVLTDNGRITAVIDWSEAMFGDSQYEVANIFFWRPWL
HygR-012        DAIAAADLSQTSGFGPFGPQGIGQYTTWRDFICAIADPHVYHWQTVMDDTVSASVAQALDELMLWAEDCPEVRHLVHADFGSNNVLTDNGRITAVIDWSEAMFGDSQYEVANIFFWRPWL
HygR-008        DAIAAADLSQTSGFGPFGPQGIGQYTTWRDFICAIADPHVYHWQTVMDDTVSASVAQALDELMLWAEDCPEVRHLVHADFGSNNVLTDNGRITAVIDWSEAMFGDSQYEVANIFFWRPWL
HygR-005        DAIAAADLSQTSGFGPFGPQGIGQYTTWRDFICAIADPHVYHWQTVMDDTVSASVAQALDELMLWAEDCPEVRHLVHADFGSNNVLTDNGRITAVIDWSEAMFGDSQYEVANIFFWRPWL
HygR-002        DAIAAADLSQTSGFGPFGPQGIGQYTTWRDFICAIADPHVYHWQTVMDDTVSASVAQALDELMLWAEDCPEVRHLVHADFGSNNVLTDNGRITAVIDWSEAMFGDSQYEVANIFFWRPWL
HygR-011        DAIAAADLSQTSGFGPFGPQGIGQYTTWRDFICAIADPHVYHWQTVMDDTVSASVAQALDELMLWAEDCPEVRHLVHADFGSNNVLTDNGRITAVIDWSEAMFGDSQYEVANIFFWRPWL
HygR-004        DAIAAADLSQTSGFGPFGPQGIGQYTTWRDFICAIADPHVYHWQTVMDDTVSASVAQALDELMLWAEDCPEVRHLVHADFGSNNVLTDNGRITAVIDWSEAMFGDSQYEVANIFFWRPWL
HygR-007        DAIAAADLSQTSGFGPFGPQGIGQYTTWRDFICAIADPHVYHWQTVMDDTVSASVAQALDELMLWAEDCPEVRHLVHADFGSNNVLTDNGRITAVIDWSEAMFGDSQYEVANIFFWRPWL
HygR-001        DAIAAADLSQTSGFGPFGPQGIGQYTTWRDFICAIADPHVYHWQTVMDDTVSASVAQALDELMLWAEDCPEVRHLVHADFGSNNVLTDNGRITAVIDWSEAMFGDSQYEVANIFFWRPWL
hphMX6          DAIAAADLSQTSGFGPFGPQGIGQYTTWRDFICAIADPHVYHWQTVMDDTVSASVAQALDELMLWAEDCPEVRHLVHADFGSNNVLTDNGRITAVIDWSEAMFGDSQYEVANIFFWRPWL
HygR-003        DAIAAADLSQTSGFGPFGPQGIGQYTTWRDFICAIADPHVYHWQTVMDDTVSASVAQALDELMLWAEDCPEVRHLVHADFGSNNVLTDNGRITAVIDWSEAMFGDSQYEVANIFFWRPWL
HygR-006        DAIAAADLSQTSGFGPFGPQGIGQYTTWRDFICAIADPHVYHWQTVMDDTVSASVAQALDELMLWAEDCPEVRHLVHADFGSNNVLTDNGRITAVIDWSEAMFGDSQYEVANIFFWRPWL
HygR-009        DAIAAADLSQTSGFGPFGPQGIGQYTTWRDFICAIADPHVYHWQTVMDDTVSASVAQALDELMLWAEDCPEVRHLVHADFGSNNVLTDNGRITAVIDWSEAMFGDSQYEVANIFFWRPWL
HygR-013corr    DAIAAADLSQTSGFGPFGPQGIGQYTTWRDFICAIADPHVYHWQTVMDDTVSASVAQALDELMLWAEDCPEVRHLVHADFGSNNVLTDNGRITAVIDWSEAMFGDSQYEVANIFFWRPWL
                ************************************************************************************************************************

HygR-010        ACMEQQTRYFERRHPELAGSPRLRAYMLRIGLDQLYQSLVDGNFDDAAWAQGRCDAIVRSGAGTVGRTQIARRSAAVWTDGCVEVLADSGNRRPSTRPRAKEVGRV
HygR-012        ACMEQQTRYFERRHPELAGSPRLRAYMLRIGLDQLYQSLVDGNFDDAAWAQGRCDAIVRSGAGTVGRTQIARRSAAVWTDGCVEVLADSGNRRPSTRPRAKK----
HygR-008        ACMEQQTRYFERRHPELAGSPRLRAYMLRIGLDQLYQSLVDGNFDDAAWAQGRCDAIVRSGAGTVGRTQIARRSAAVWTDGCVEVLADSGNRRPSTRPRAKE----
HygR-005        ACMEQQTRYFERRHPELAGSPRLRAYMLRIGLDQLYQSLVDGNFDDAAWAQGRCDAIVRSGAGTVGRTQIARRSAAVWTDGCVEVLADSGNRRPSTRPRAKE----
HygR-002        ACMEQQTRYFERRHPELAGSPRLRAYMLRIGLDQLYQSLVDGNFDDAAWAQGRCDAIVRSGAGTVGRTQIARRSAAVWTDGCVEVLADSGNRRPSTRPDREMGEAN
HygR-011        ACMEQQTRYFERRHPELAGSPRLRAYMLRIGLDQLYQSLVDGNFDDAAWAQGRCDAIVRSGAGTVGRTQIARRSAAVWTDGCVEVLADSGNRRPSTRPRAKE----
HygR-004        ACMEQQTRYFERRHPELAGSPRLRAYMLRIGLDQLYQSLVDGNFDDAAWAQGRCDAIVRSGAGTVGRTQIARRSAAVWTDGCVEVLADSGNRRPSTRPRAKE----
HygR-007        ACMEQQTRYFERRHPELAGSPRLRAYMLRIGLDQLYQSLVDGNFDDAAWAQGRCDAIVRSGAGTVGRTQIARRSAAVWTDGCVEVLADSGNRRPSTRPRAKE----
HygR-001        ACMEQQTRYFERRHPELAGSPRLRAYMLRIGLDQLYQSLVDGNFDDAAWAQGRCDAIVRSGAGTVGRTQIARRSAAVWTDGCVEVLADSGNRRPSTRPRAKE----
hphMX6          ACMEQQTRYFERRHPELAGSPRLRAYMLRIGLDQLYQSLVDGNFDDAAWAQGRCDAIVRSGAGTVGRTQIARRSAAVWTDGCVEVLADSGNRRPSTRPRAKE----
HygR-003        ACMEQQTRYFERRHPELAGSPRLRAYMLRIGLDQLYQSLVDGNFDDAAWAQGRCDAIVRSGAGTVGRTQIARRSAAVWTDGCVEVLADSGNRRPSTRPRAKE----
HygR-006        ACMEQQTRYFERRHPELAGSPRLRAYMLRIGLDQLYQSLVDGNFDDAAWAQGRCDAIVRSGAGTVGRTQIARRSAAVWTDGCVEVLADSGNRRPSTRPRAKE----
HygR-009        ACMEQQTRYFERRHPELAGSPRLRAYMLRIGLDQLYQSLVDGNFDDAAWAQGRCDAIVRSGAGTVGRTQIARRSAAVWTDGCVEVLADSGNRRPSTRPRAKE----
HygR-013corr    ACMEQQTRYFERRHPELAGSPRLRAYMLRIGLDQLYQSLVDGNFDDAAWAQGRCDAIVRSGAGTVGRTQIARRSAAVWTDGCVEVLADSGNRRPSTRPRAKEYR--
                **************************************************************************************************   :
```

Sequence Alignments

```
Name of   # of     Alignment                                                                                                                 Size   # Var   Sources*
Variant   Occur                                                                                                                              (bp)   bp
KanR-009  51       ATGAGCCATATTCAACGGGAAACGTCTTGCTCTAGGCCGCGATTAAATTCCAACATGGATGCTGATTTATATGGGTATAAATGGGCTCGCGATAATGTCGGGCAATCAGGTGCGACAAT  816    1       No,Qi,19906724
KanR-021  16       ATGGGTAAGGAAAAGACTCACGTTT------CGAGGCCGCGATTAAATTCCAACATGGATGCTGATTTATATGGGTATAAATGGGCTCGCGATAATGTCGGGCAATCAGGTGCGACAAT  810    19/6d   9717241,7747518
KanR-005  9        ATGAGCCATATTCAACGGGAAACGTCTTGCTCGAGGCCGCGATTAAATTCCAACATGGATGCTGATTTATATGGGTATAAATGGGCTCGCGATAATGTCGGGCAATCAGGTGCGACAAT  816    1       No,Cl,In
KanR-010  6        ATGAGCCATATTCAACGGGAAACGTCTTGCTCTAGGCCGCGATTAAATTCCAACATGGATGCTGATTTATATGGGTATAAATGGGCTCGCGATAATGTCGGGCAATCAGGTGCGACAAT  816    2       No
KanR-015  6        ATGAGCCATATTCAACGGGAAACGTCTTGCTCCCGTCCGCGCTTAAACTCCAACATGGACGCTGATTTATATGGGTATAAATGGGCTCGCGATAATGTCGGGCAATCAGGTGCGACAAT  816    30      24050148
KanR-008  5        ATGAGCCATATTCAACGGGAAACGTCTTGCTCAAGGCCGCGATTAAATTCCAACATGGATGCTGATTTATATGGGTATAAATGGGCTCGCGATAATGTCGGGCAATCAGGTGCGACAAT  816    4       10890530
KanR-019  4        ATGAGCCATATTCAACGGGAAACGTCTTGCTCTAGGCCGCGATTAAATTCCAACATGGATGCTGATTTATATGGGTATAAATGGGCTCGCGATAATGTCGGGCAATCAGGTGCGACAAT  816    3       No
KanR-014  3        ATGAGCCATATTCAACGGGAAACGTCTTGCTCGAGGCCGCGATTAAATTCCAACATGGATGCTGATTTATATGGGTATAAATGGGCTCGCGATAATGTCGGGCAATCAGGTGCGACAAT  816    6       Lu
KanR-001  2        ATGAGCCATATTCAACGGGAAACGTCTTGCTCCAGGCCGCGATTAAATTCCAACATGGATGCTGATTTATATGGGTATAAATGGGCTCGCGATAATGTCGGGCAATCAGGTGCGACAAT  816    3       10890530
KanR-002  2        ATGAGCCATATTCAACGGGAAACGTCTTGCTCGAGGCCGCGATTAAATTCCAACATGGATGCTGATTTATATGGGTATAAATGGGCTCGCGATAATGTCGGGCAATCAGGTGCGACAAT  816    7       Lu,16496398
KanR-003  2        ATGAGCCATATTCAACGGGAAACGTCTTGCTCTAGGCCGCGATTAAATTCCAACATGGATGCTGATTTATATGGGTATAAATGGGCTCGCGATAATGTCGGGCAATCAGGTGCGACAAT  816    2       19906724
KanR-004  2        ATGAGCCATATTCAACGGGAAACGT------CGAGGCCGCGATTAAATTCCAACATGGATGCTGATTTATATGGGTATAAATGGGCTCGCGATAATGTCGGGCAATCAGGTGCGACAAT  810    2/6d    In,GE
KanR-006  2        ATGAGCCATATTCAACGGGAAACGTCTTGCTCTAGGCCGCGATTAAATTCCAACATGGATGCTGATTTATATGGGTATAAATGGGCTCGCGATAATGTCGGGCAATCAGGTGCGACAAT  816    2       19906724
KanR-007  2        ATGAGCCATATTCAACGGGAAACGT------CGAGGCCGCGATTAAATTCCAACATGGATGCTGATTTATATGGGTATAAATGGGCTCGCGATAATGTCGGGCAATCAGGTGCGACAAT  810    3/6d    Or
KanR-011  2        ATGAGCCATATTCAACGGGAAACGT------CGAGGCCGCGATTAAATTCCAACATGGATGCTGATTTATATGGGTATAAATGGGCTCGCGATAATGTCGGGCAATCAGGTGCGACAAT  810    3/6d    MCSG
KanR-012  2        ATGAGCCATATTCAACGGGAAACGTCTTGCTCGAAGCCGCGATTAAATTCCAACATGGATGCTGATTTATATGGGTATAAATGGGCTCGCGATAATGTCGGGCAATCAGGTGCGACAAT  816    2       3327753
KanR-013  1        ATGAGCCATATTCAACGGGAAACGT------CGAGGCCGCGATTAAATTCCAACATGGATGCTGATTTATATGGGTATAAATGGGCTCGCGATAATGTCGGGCAATCAGGTGCGACAAT  810    3/6d    Or
KanR-017  1        ATGAGCCATATTCAACGGGAAACGTCTTGCTCGAGGCCGCGATTAAATTCCAACATGGATGCTGATTTATATGGGTATAAATGGGCTCGCGATAATGTCGGGCAATCAGGTGCGACAAT  816    3       24057978
KanR-018  1        ATGAGCCATATTCAACGGGAAACGTCTTGCTCGAGGCCGCGATTAAATTCCAACATGGATGCTGATTTATATGGGTATAAATGGGCTCGCGATAATGTCGGGCAATCAGGTGCGACAAT  816    5       20040575
                   *** *  *     *     *    *      *    ***** ***** *********** ***********************************************************

KanR-009           CTATCGATTGTATGGGAAGCCCGATGCGCCAGAGTTGTTTCTGAAACATGGCAAAGGTAGCGTTGCCAATGATGTTACAGATGAGATGGTCAGACTAAACTGGCTGACGGAATTTATGCC
KanR-021           CTATCGATTGTATGGGAAGCCCGATGCGCCAGAGTTGTTTCTGAAACATGGCAAAGGTAGCGTTGCCAATGATGTTACAGATGAGATGGTCAGACTAAACTGGCTGACGGAATTTATGCC
KanR-005           CTATCGATTGTATGGGAAGCCCGATGCGCCAGAGTTGTTTCTGAAACATGGCAAAGGTAGCGTTGCCAATGATGTTACAGATGAGATGGTCAGACTAAACTGGCTGACGGAATTTATGCC
KanR-010           CTATCGATTGTATGGGAAGCCCGATGCGCCAGAGTTGTTTCTGAAACATGGCAAAGGTAGCGTTGCCAATGATGTTACAGATGAGATGGTCAGACTAAACTGGCTGACGGAATTTATGCC
KanR-015           CTATCGCTTGTATGGGAAGCCCGATGCGCCAGAGTTGTTTCTGAAACATGGCAAAGGTAGCGTTGCCAATGATGTTACAGATGAGATGGTCCGTCTCAACTGGCTGACGGAGTTTATGCC
KanR-008           CTACCGATTGTATGGGAAGCCCGATGCGCCAGAGTTGTTTCTGAAACATGGCAAAGGTAGCGTTGCCAATGTTGTTACAGATGAGATGGTCAGACTAAACTGGCTGACGGAATTTATGCC
KanR-019           CTATCGATTGTATGGGAAGCCCGATGCGCCAGAGTTGTTTCTGAAACATGGCAAAGGTAGCGTTGCCAATGATGTTACAGATGAGATGGTCAGACTAAACTGGCTGACGGAATTTATGCC
KanR-014           CTATCGATTGTATGGGAAGCCCGATGCGCCAGAGTTGTTTCTGAAACATGGCAAAGGTAGCGTTGCCAATGATGTTACAGATGAGATGGTCAGGCTAAACTGGCTGACGGAATTTATGCC
KanR-001           CTATGGATTGTATGGGAAGCCCGATGCGCCAGAGTTGTTTCTGAAACATGGCAAAGGTAGCGTTGCCAATGATGTTACAGATGAGATGGTCAGACTAAACTGGCTGACGGAATTTATGCC
KanR-002           CTATCGATTGTATGGGAAGCCCGATGCGCCAGAGTTGTTTCTGAAACATGGCAAAGGTAGCGTTGCCAATGATGTTACAGATGAGATGGTCAGGCTAAACTGGCTGACGGAATTTATGCC
KanR-003           CTATCGATTGTATGGGAAGCCCGATGCGCCAGAGTTGTTTCTGAAACATGGCAAAGGTAGCGTTGCCAATGATGTTACAGATGAGATGGTCAGACTAAACTGGCTGACGGAATTTATGCC
KanR-004           CTATCGCTTGTATGGGAAGCCCGATGCGCCAGAGTTGTTTCTGAAACATGGCAAAGGTAGCGTTGCCAATGATGTTACAGATGAGATGGTCAGACTAAACTGGCTGACGGAATTTATGCC
KanR-006           CTATCGATTGTATGGGAAGCCCGATGCGCCAGAGTTGTTTCTGAAACATGGCAAAGGTAGCGTTGCCAATGATGTTACAGATGAGATGGTCAGACTAAACTGGCTGACGGAATTTATGCC
KanR-007           CTATCGCTTGTATGGGAAGCCCGATGCGCCAGAGTTGTTTCTGAAACATGGCAAAGGTAGCGTTGCCAATGATGTTACAGATGAGATGGTCAGACTAAACTGGCTGACGGAATTTATGCC
KanR-011           CTATCGCTTGTATGGGAAGCCCGATGCGCCAGAGTTGTTTCTGAAACATGGCAAAGGTAGCGTTGCCAATGATGTTACAGATGAGATGGTCAGACTAAACTGGCTGACGGAATTTATGCC
KanR-012           CTATCGATTGTATGGGAAGCCCGATGCGCCAGAGTTGTTTCTGAAACATGGCAAAGGTAGCGTTGCCAATGATGTTACAGATGAGATGGTCAGACTAAACTGGCTGACGGAATTTATGCC
KanR-013           CTATCGCTTGTATGGGAAGCCCGATGCGCCAGAGTTGTTTCTGAAACATGGCAAAGGTAGCGTTGCCAATGATGTTACAGATGAGATGGTCAGACTAAACTGGCTGACGGAATTTATGCC
KanR-017           CTATCGATTGTATGGGAAGCCCGATGCGCCAGAGTTGTTTCTGAAACATGGCAAAGGTAGCGTTGCCAATGATGTTACAGATGAGATGGTCAGACTAAACTGGCTGACGGAATTTATGCC
KanR-018           CTATCGATTGTATGGGAAGCCCGATGCGCCAGAGTTGTTTCTGAAACATGGCAAAGGTAGCGTTGCCAATGATGTTACAGATGAGATGGTCAGGCTAAACTGGCTGACGGAATTTATGCC
                   ***  * **************************************************************** ******************* * ** ************** ********

KanR-009           TCTTCCGACCATCAAGCATTTTATCCGTACTCCTGATGATGCATGGTTACTCACCACTGCGATCCCCGGGAAAACAGCATTCCAGGTATTAGAAGAATATCCTGATTCAGGTGAAAATAT
KanR-021           TCTTCCGACCATCAAGCATTTTATCCGTACTCCTGATGATGCATGGTTACTCACCACTGCGATCCCCGGCAAAACAGCATTCCAGGTATTAGAAGAATATCCTGATTCAGGTGAAAATAT
KanR-005           TCTTCCGACCATCAAGCATTTTATCCGTACTCCTGATGATGCATGGTTACTCACCACTGCGATCCCCGGGAAAACAGCATTCCAGGTATTAGAAGAATATCCTGATTCAGGTGAAAATAT
KanR-010           TCTTCCGACCATCAAGCATTTTATCCGTACTCCTGATGATGCATGGTTACTCACCACTGCGATCCCCGGGAAAACAGCATTCCAGGTATTAGAAGAATATCCTGATTCAGGTGAAAATAT
KanR-015           TCTCCCGACCATCAAGCATTTTATCCGTACTCCTGATGATGCGTGGTTACTCACCACCGCGATTCCTGGGAAAACAGCCTTCCAGGTATTAGAAGAATATCCTGATTCAGGTGAAAATAT
KanR-008           TCTTCCGACCATCAAGCATTTTATCCGTACTCCTGATGATGCATGGTTACTCACCACTGCGATCCCAGGGAAAACAGCATTCCAGGTATTAGAAGAATATCCTGATTCAGGTGAAAATAT
KanR-019           TCTTCCGACCATCAAGCATTTTATCCGTACTCCTGATGATGCATGGTTACTCACCACTGCGATCCCCGGCAAAACAGCATTCCAGGTATTAGAAGAATATCCTGATTCAGGTGAAAATAT
KanR-014           TCTTCCGACCATCAAGCATTTTATCCGTACTCCTGATGATGCATGGTTACTCACCACTGCGATCCCAGGGAAAACAGCATTCCAGGTATTAGAAGAATATCCTGATTCAGGTGAAAATAT
KanR-001           TCTTCCGACCATCAAGCATTTTATCCGTACTCCTGATGATGCATGGTTACTCACCACTGCGATCCCAGGGAAAACAGCATTCCAGGTATTAGAAGAATATCCTGATTCAGGTGAAAATAT
KanR-002           TCTTCCGACCATCAAGCATTTTATCCGTACTCCTGATGATGCATGGTTACTCACCACTGCGATCCCAGGGAAAACAGCATTCCAGGTATTAGAAGAATATCCTGATTCAGGTGAAAATAT
KanR-003           TCTTCCGACCATCAAGCATTTTATCCGTACTCCTGATGATGCATGGTTACTCACCACTGCGATCCCCAGGAAAACAGCATTCCAGGTATTAGAAGAATATCCTGATTCAGGTGAAAATAT
KanR-004           TCTTCCGACCATCAAGCATTTTATCCGTACTCCTGATGATGCATGGTTACTCACCACTGCGATCCCCGGAAAAACAGCATTCCAGGTATTAGAAGAATATCCTGATTCAGGTGAAAATAT
KanR-006           TCTTCCGACCATCAAGCATTTTATCCGTACTCCTGATGATGCATGGTTACTCACCACTGCGATCCCCGGGAAAACAGCATTCCAGGTATTAGAAGAATATCCTGATTCAGGTGAAAACAT
KanR-007           TCTTCCGACCATCAAGCATTTTATCCGTACTCCTGATGATGCATGGTTACTCACCACTGCGATCCCCGGAAAAACAGCATTCCAGGTATTAGAAGAATATCCTGATTCAGGTGAAAATAT
KanR-011           TCTTCCGACCATCAAGCATTTTATCCGTACTCCTGATGATGCATGGTTACTCACCACTGCGATCCCCGGAAAAACAGCATTCCAGGTATTAGAAGAATATCCTGATTCAGGTGAAAACAT
KanR-012           TCTTCCGACCATCAAGCATTTTATCCGTACTCCTGATGATGCATGGTTACTCACCACTGCGATCCCCGGGAAAACAGCATTCCAGGTATTAGAAGAATATCCTGATTCAGGTGAAAATAT
KanR-013           TCTTCCGACCATCAAGCATTTTATCCGTACTCCTGATGATGCATGGTTACTCACCACTGCGATCCCCGGAAAAACAGCATTCCAGGTATTAGAAGAATATCCTGATTCAGGTGAAAATAT
KanR-017           TCTTCCGACCATCAAGCATTTTATCCGTACTCCTGATGATGCATGGTTACTCACCACTGCGATCCCCCGGAAAACAGCATTCCAGGTATTAGAAGAATATCCTGATTCAGGTGAAAACAT
KanR-018           TCTTCCGACCATCAAGCATTTTATCCGTACTCCTGATGATGCATGGTTACTCACCACTGCGATCCCAGGGAAAACAGCATTCCAGGTATTAGAAGAATATCCTGATTCAGGTGAAAATAT
                   *** ************************************** ************** ***** **  * ******** ************************************** **
```

Sequence Alignments

```
KanR-009          TGTTGATGCGCTGGCAGTGTTCCTGCGCCGGTTGCATTCGATTCCTGTTTGTAATTGTCCTTTTAACAGCGATCGCGTATTTCGTCTCGCTCAGGCGCAATCACGAATGAATAACGGTTT
KanR-021          TGTTGATGCGCTGGCAGTGTTCCTGCGCCGGTTGCATTCGATTCCTGTTTGTAATTGTCCTTTTAACAGCGATCGCGTATTTCGTCTCGCTCAGGCGCAATCACGAATGAATAACGGTTT
KanR-005          TGTTGATGCGCTGGCAGTGTTCCTGCGCCGGTTGCATTCGATTCCTGTTTGTAATTGTCCTTTTAACAGCGATCGCGTATTTCGTCTCGCTCAGGCGCAATCACGAATGAATAACGGTTT
KanR-010          TGTTGATGCGCTGGCAGTGTTCCTGCGCCGGTTGCATTCGATTCCTGTTTGTAATTGTCCTTTTAACAGCGACCGCGTATTTCGTCTCGCTCAGGCGCAATCACGAATGAATAACGGTTT
KanR-015          TGTTGATGCGCTGGCCGTGTTCCTGCGCCGGTTACATTCGATTCCTGTTTGTAATTGTCCTTTTAACAGCGATCGTGTATTTCGTCTTGCTCAGGCGCAATCACGCATGAATAACGGTTT
KanR-008          TGTTGATGCGCTGGCAGTGTTCCTGCGCCGGTTGCATTCGATTCCTGTTTGTAATTGTCCTTTTAACAGCGATCGCGTATTTCGTCTCGCTCAGGCGCAATCACGAATGAATAACGGTTT
KanR-019          TGTTGATGCGCTGGCAGTGTTCCTGCGCCGGTTGCATTCGATTCCTGTTTGTAATTGTCCTTTTAACAGTGATCGCGTATTTCGTCTCGCTCAGGCGCAATCACGAATGAATAACGGTTT
KanR-014          TGTTGATGCGCTGGCAGTGTTCCTGCGCCGGTTGCATTCGATTCCTGTTTGTAATTGTCCTTTTAACGGCGATCGCGTATTTCGTCTCGCTCAGGCGCAATCACGAATGAATAACGGTTT
KanR-001          TGTTGATGCGCTGGCAGTGTTCCTGCGCCGGTTGCATTCGATTCCTGTTTGTAATTGTCCTTTTAACAGCGATCGCGTATTTCGTCTCGCTCAGGCGCAATCACGAATGAATAACGGTTT
KanR-002          TGTTGATGCGCTGGCAGTGTTCCTGCGCCGGTTGCATTCGATTCCTGTTTGTAATTGTCCTTTTAACGGCGATCGCGTATTTCGTCTCGCTCAGGCGCAATCACGAATGAATAACGGTTT
KanR-003          TGTTGATGCGCTGGCAGTGTTCCTGCGCCGGTTGCATTCGATTCCTGTTTGTAATTGTCCTTTTAACAGCGATCGCGTATTTCGTCTCGCTCAGGCGCAATCACGAATGAATAACGGTTT
KanR-004          TGTTGATGCGCTGGCAGTGTTCCTGCGCCGGTTGCATTCGATTCCTGTTTGTAATTGTCCTTTTAACAGCGATCGCGTATTTCGTCTCGCTCAGGCGCAATCACGAATGAATAACGGTTT
KanR-006          TGTTGATGCGCTGGCAGTGTTCCTGCGCCGGTTGCATTCGATTCCTGTTTGTAATTGTCCTTTTAACAGCGATCGCGTATTTCGTCTCGCTCAGGCGCAATCACGAATGAATAACGGTTT
KanR-007          TGTTGATGCGCTGGCAGTGTTCCTGCGCCGGTTGCATTCGATTCCTGTTTGTAATTGTCCTTTTAACAGCGATCGAGTATTTCGTCTCGCTCAGGCGCAATCACGAATGAATAACGGTTT
KanR-011          TGTTGATGCGCTGGCAGTGTTCCTGCGCCGGTTGCATTCGATTCCTGTTTGTAATTGTCCTTTTAACAGCGATCGCGTATTTCGTCTCGCTCAGGCGCAATCACGAATGAATAACGGTTT
KanR-012          TGTTGATGCGCTGGCAGTGTTCCTGCGCCGGTTGCATTCGATTCCTGTTTGTAATTGTCCTTTTAACAGCGATCGCGTATTTCGTCTCGCTCAGGCGCAATCACGAATGAATAACGGTTT
KanR-013          TGTTGATGCGCTGGCAGTGTTCCTGCGCCGGTTGCATTCGATTCCTGTTTGTAATTGTCCTTTTAACAGCGGTCGCGTATTTCGTCTCGCTCAGGCGCAATCACGAATGAATAACGGTTT
KanR-017          TGTTGATGCGCTGGCAGTGTTCCTGCGCCGGTTGCATTCGATTCCTGTTTGTAATTGTCCTTTTAACAGCGATCGCGTATTTCGTCTCGCTCAGGCGCAATCACGAATGAATAACGGTTT
KanR-018          TGTTGATGCGCTGGCAGTGTTCCTGCGCCGGTTGCATTCGATTCCTGTTTGTAATTGTCCTTTTAACGGCGATCGCGTATTTCGTCTCGCTCAGGCGCAATCACGAATGAATAACGGTTT
                  *************** ***************** ********************************* * * * * *********** ***************** **************

KanR-009          GGTTGATGCGAGTGATTTTGATGACGAGCGTAATGGCTGGCCTGTTGAACAAGTCTGGAAAGAAATGCATAAACTTTTGCCATTCTCACCGGATTCAGTCGTCACTCATGGTGATTTCTC
KanR-021          GGTTGATGCGAGTGATTTTGATGACGAGCGTAATGGCTGGCCTGTTGAACAAGTCTGGAAAGAAATGCATAAGCTTTTGCCATTCTCACCGGATTCAGTCGTCACTCATGGTGATTTCTC
KanR-005          GGTTGATGCGAGTGATTTTGATGACGAGCGTAATGGCTGGCCTGTTGAACAAGTCTGGAAAGAAATGCATAAGCTTTTGCCATTCTCACCGGATTCAGTCGTCACTCATGGTGATTTCTC
KanR-010          GGTTGATGCGAGTGATTTTGATGACGAGCGTAATGGCTGGCCTGTTGAACAAGTCTGGAAAGAAATGCATAAACTTTTGCCATTCTCACCGGATTCAGTCGTCACTCATGGTGATTTCTC
KanR-015          GGTTGATGCGAGTGATTTTGATGACGAGCGTAATGGCTGGCCTGTTGAACAAGTCTGGAAAGAAATGCACAAGCTCTTGCCATTCTCACCGGATTCAGTCGTCACTCATGGTGATTTCTC
KanR-008          GGTTGATGCGAGTGATTTTGATGACGAGCGTAATGGCTGGCCTGTTGAACAAGTCTGGAAAGAAATGCATAAACTTTTGCCATTCTCACCGGATTCAGTCGTCACTCATGGTGATTTCTC
KanR-019          GGTTGATGCGAGTGATTTTGATGACGAGCGTAATGGCTGGCCTGTTGAACAAGTCTGGAAAGAAATGCATAAACTTTTGCCATTCTCACCGGATTCAGTCGTCACTCATGGTGATTTCTC
KanR-014          GGTTGGTGCGAGTGATTTTGATGACGAGCGTAATGGCTGGCCTGTTGAACAAGTCTGGAAAGAAATGCATAAGCTTTTGCCATTCTCACCGGATTCAGTCGTCACTCATGGTGATTTCTC
KanR-001          GGTTGATGCGAGTGATTTTGATGACGAGCGTAATGGCTGGCCTGTTGAACAAGTCTGGAAAGAAATGCATAAACTTTTGCCATTCTCACCGGATTCAGTCGTCACTCATGGTGATTTCTC
KanR-002          GGTTGGTGCGAGTGATTTTGATGACGAGCGTAATGGCTGGCCTGTTGAACAAGTCTGGAAAGAAATGCATAAGCTTTTGCCATTCTCACCGGATTCAGTCGTCACTCATGGTGATTTCTC
KanR-003          GGTTGATGCGAGTGATTTTGATGACGAGCGTAATGGCTGGCCTGTTGAACAAGTCTGGAAAGAAATGCATAAACTTTTGCCATTCTCACCGGATTCAGTCGTCACTCATGGTGATTTCTC
KanR-004          GGTTGATGCGAGTGATTTTGATGACGAGCGTAATGGCTGGCCTGTTGAACAAGTCTGGAAAGAAATGCATAAACTTTTGCCATTCTCACCGGATTCAGTCGTCACTCATGGTGATTTCTC
KanR-006          GGTTGATGCGAGTGATTTTGATGACGAGCGTAATGGCTGGCCTGTTGAACAAGTCTGGAAAGAAATGCATAAACTTTTGCCATTCTCACCGGATTCAGTCGTCACTCATGGTGATTTCTC
KanR-007          GGTTGATGCGAGTGATTTTGATGACGAGCGTAATGGCTGGCCTGTTGAACAAGTCTGGAAAGAAATGCATAAACTTTTGCCATTCTCACCGGATTCAGTCGTCACTCATGGTGATTTCTC
KanR-011          GGTTGATGCGAGTGATTTTGATGACGAGCGTAATGGCTGGCCTGTTGAACAAGTCTGGAAAGAAATGCATAAACTTTTGCCATTCTCACCGGATTCAGTCGTCACTCATGGTGATTTCTC
KanR-012          GGTTGATGCGAGTGATTTTGATGACGAGCGTAATGGCTGGCCTGTTGAACAAGTCTGGAAAGAAATGCATAAGCTTTTGCCATTCTCACCGGATTCAGTCGTCACTCATGGTGATTTCTC
KanR-013          GGTTGATGCGAGTGATTTTGATGACGAGCGTAATGGCTGGCCTGTTGAACAAGTCTGGAAAGAAATGCATAAACTTTTGCCATTCTCACCGGATTCAGTCGTCACTCATGGTGATTTCTC
KanR-017          GGTTGATGCGAGTGATTTTGATGACGAGCGTAATGGCTGGCCTGTTGAACAAGTCTGGAAAGAAATGCATAAGCTTTTGCCATTCTCACCGGATTCAGTCGTCACTCATGGTGATTTCTC
KanR-018          GGTTGGTGCGAGTGATTTTGATGACGAGCGTAATGGCTGGCCTGTTGAACAAGTCTGGAAAGAAATGCATAAACTTTTGCCATTCTCACCGGATTCAGTCGTCACTCATGGTGATTTCTC
                  ***** *************************************************************** ** ** ********************************************

KanR-009          ACTTGATAACCTTATTTTTGACGAGGGGAAATTAATAGGTTGTATTGATGTTGGACGAGTCGGAATCGCAGACCGATACCAGGATCTTGCCATCCTATGGAACTGCCTCGGTGAGTTTTC
KanR-021          ACTTGATAACCTTATTTTTGACGAGGGGAAATTAATAGGTTGTATTGATGTTGGACGAGTCGGAATCGCAGACCGATACCAGGATCTTGCCATCCTATGGAACTGCCTCGGTGAGTTTTC
KanR-005          ACTTGATAACCTTATTTTTGACGAGGGGAAATTAATAGGTTGTATTGATGTTGGACGAGTCGGAATCGCAGACCGATACCAGGATCTTGCCATCCTATGGAACTGCCTCGGTGAGTTTTC
KanR-010          ACTTGATAACCTTATTTTTGACGAGGGGAAATTAATAGGTTGTATTGATGTTGGACGAGTCGGAATCGCAGACCGATACCAGGATCTTGCCATCCTATGGAACTGCCTCGGTGAGTTTTC
KanR-015          ACTTGATAACCTTATTTTTGACGAGGGGAAATTAATAGGTTGTATTGATGTTGGACGGGTCGGAATCGCAGACCGTTACCAGGACCTTGCCATTCTTTGGAACTGCCTCGGTGAGTTTTC
KanR-008          ACTTGATAACCTTATTTTTGACGAGGGGAAATTAATAGGTTGTATTGATGTTGGACGAGTCGGAATCGCAGACCGATACCAGGATCTTGCCATCCTATGGAACTGCCTCGGTGAGTTTTC
KanR-019          ACTTGATAACCTTATTTTTGACGAGGGGAAATTAATAGGTTGTATTGATGTTGGACGAGTCGGAATCGCAGACCGATACCAGGATCTTGCCATCCTATGGAACTGCCTCGGTGAGTTTTC
KanR-014          ACTTGATAACCTTATTTTTGACGAGGGGAAATTAATAGGTTGTATTGATGTTGGACGAGTCGGAATCGCAGACCGATACCAGGATCTTGCCATCCTATGGAACTGCCTCGGTGAGTTTTC
KanR-001          ACTTGATAACCTTATTTTTGACGAGGGGAAATTAATAGGTTGTATTGATGTTGGACGAGTCGGAATCGCAGACCGATACCAGGATCTTGCCATCCTATGGAACTGCCTCGGTGAGTTTTC
KanR-002          ACTTGATAACCTTATTTTTGACGAGGGGAAATTAATAGGTTGTATTGATGTTGGACGAGTCGGAATCGCAGACCGATACCAGGATCTTGCCATCCTATGGAACTGCCTCGGTGAGTTTTC
KanR-003          ACTTGATAACCTTATTTTTGACGAGGGGAAATTAATAGGTTGTATTGATGTTGGACGAGTCGGAATCGCAGACCGATACCAGGATCTTGCCATCCTATGGAACTGCCTCGGTGAGTTTTC
KanR-004          ACTTGATAACCTTATTTTTGACGAGGGGAAATTAATAGGTTGTATTGATGTTGGACGAGTCGGAATCGCAGACCGATACCAGGATCTTGCCATCCTATGGAACTGCCTCGGTGAGTTTTC
KanR-006          ACTTGATAACCTTATTTTTGACGAGGGGAAATTAATAGGTTGTATTGATGTTGGACGAGTCGGAATCGCAGACCGATACCAGGATCTTGCCATCCTATGGAACTGCCTCGGTGAGTTTTC
KanR-007          ACTTGATAACCTTATTTTTGACGAGGGGAAATTAATAGGTTGTATTGATGTTGGACGAGTCGGAATCGCAGACCGATACCAGGATCTTGCCATCCTATGGAACTGCCTCGGTGAGTTTTC
KanR-011          ACTTGATAACCTTATTTTTGACGAGGGGAAATTAATAGGTTGTATTGATGTTGGACGAGTCGGAATCGCAGACCGATACCAGGATCTTGCCATCCTATGGAACTGCCTCGGTGAGTTTTC
KanR-012          ACTTGATAACCTTATTTTTGACGAGGGGAAATTAATAGGTTGTATTGATGTTGGACGAGTCGGAATCGCAGACCGATACCAGGATCTTGCCATCCTATGGAACTGCCTCGGTGAGTTTTC
KanR-013          ACTTGATAACCTTATTTTTGACGAGGGGAAATTAATAGGTTGTATTGATGTTGGACGAGTCGGAATCGCAGACCGATACCAGGATCTTGCCATCCTATGGAACTGCCTCGGTGAGTTTTC
KanR-017          ACTTGATAACCTTATTTTTGACGAGGGGAAATTAATAGGTTGTATTGATGTTGGACGAGTCGGAATCGCAGACCGATACCAGGATCTTGCCATCCTATGGAACTGCCTCGGTGAGTTTTC
KanR-018          ACTTGATAACCTTATTTTTGACGAGGGGAAATTAATAGGTTGTATTGATGTTGGACGAGTCGGAATCGCAGACCGATACCAGGATCTTGCCATCCTATGGAACTGCCTCGGTGAGTTTTC
                  ********************************************************* ***************** ******** ******** ** ***********************
```

Sequence Alignments

KanR-009	TCCTTCATTACAGAAACGGCTTTTTCAAAAATATGGTATTGATAATCCTGATATGAATAAATTGCAGTTTCATTTGATGCTCGATGAGTTTTTCTAA
KanR-021	TCCTTCATTACAGAAACGGCTTTTTCAAAAATATGGTATTGATAATCCTGATATGAATAAATTGCAGTTTCATTTGATGCTCGATGAGTTTTTCTAA
KanR-005	TCCTTCATTACAGAAACGGCTTTTTCAAAAATATGGTATTGATAATCCTGATATGAATAAATTGCAGTTTCATTTGATGCTCGATGAGTTTTTCTAA
KanR-010	TCCTTCATTACAGAAACGGCTTTTTCAAAAATATGGTATTGATAATCCTGATATGAATAAATTGCAGTTTCATTTGATGCTCGATGAGTTTTTCTAA
KanR-015	TCCTTCATTACAGAAACGGCTTTTTCAAAAATATGGTATTGATAATCCTGATATGAATAAATTGCAGTTTCATTTGATGCTCGATGAGTTTTTCTAA
KanR-008	TCCTTCATTACAGAAACGGCTTTTTCAAAAATATGGTATTGATAATCCTGATATGAATAAATTGCAGTTTCATTTGATGCTCGATGAGTTTTTCTAA
KanR-019	TCCTTCATTACAGAAACGGCTTTTTCAAAAATATGGTATTGATAATCCTGATATGAATAAATTGCAGTTTCATTTGATGCTCGATGAGTTTTTCTAA
KanR-014	TCCTTCATTACAGAAACGGCTTTTTCAAAAATATGGTATTGATAATCCTGATATGAATAAATTGCAGTTTCACTTGATGCTCGATGAGTTTTTCTAA
KanR-001	TCCTTCATTACAGAAACGGCTTTTTCAAAAATATGGTATTGATAATCCTGATATGAATAAATTGCAGTTTCATTTGATGCTCGATGAGTTTTTCTAA
KanR-002	TCCTTCATTACAGAAACGGCTTTTTCAAAAATATGGTATTGATAATCCTGATATGAATAAATTGCAGTTTCACTTGATGCTCGATGAGTTTTTCTGA
KanR-003	TCCTTCATTACAGAAACGGCTTTTTCAAAAATATGGTATTGATAATCCTGATATGAATAAATTGCAGTTTCATTTGATGCTCGATGAGTTTTTCTAA
KanR-004	TCCTTCATTACAGAAACGGCTTTTTCAAAAATATGGTATTGATAATCCTGATATGAATAAATTGCAGTTTCATTTGATGCTCGATGAGTTTTTCTAA
KanR-006	TCCTTCATTACAGAAACGGCTTTTTCAAAAATATGGTATTGATAATCCTGATATGAATAAATTGCAGTTTCATTTGATGCTCGATGAGTTTTTCTAA
KanR-007	TCCTTCATTACAGAAACGGCTTTTTCAAAAATATGGTATTGATAATCCTGATATGAATAAATTGCAGTTTCATTTGATGCTCGATGAGTTTTTCTAA
KanR-011	TCCTTCATTACAGAAACGGCTTTTTCAAAAATATGGTATTGATAATCCTGATATGAATAAATTGCAGTTTCATTTGATGCTCGATGAGTTTTTCTAA
KanR-012	TCCTTCATTACAGAAACGGCTTTTTCAAAAATATGGTATTGATAATCCTGATATGAATAAATTGCAGTTTCATTTGATGCTCGATGAGTTTTTCTAA
KanR-013	TCCTTCATTACAGAAACGGCTTTTTCAAAAATATGGTATTGATAATCCTGATATGAATAAATTGCAGTTTCATTTGATGCTCGATGAGTTTTTCTAA
KanR-017	TCCTTCATTACAGAAACGGCTTTTTCAAAAATATGGTATTGATAATCCTGATATGAATAAATTGCAGTTTCATTTGATGCTCGATGAGTTTTTCTAA
KanR-018	TCCTTCATTACAGAAACGGCTTTTTCAAAAATATGGTATTGATAATCCTGATATGAATAAATTGCAGTTTCACTTGATGCTCGATGAGTTTTTCTAA
	*** *********************** *

KanR is *aph(3')-Ia* (aminoglycoside 3'-phosphotransferase) from *E. coli Tn5* transposon.
KanR-016 (5 occurrences) is *nptII* identical to NeoR/KanR but has an additional N-term nos (nopaline synthase) fusion.
KanR-020 (50 occurrences) is *aphA-3* from *Staphylococcus aureus*. There is only one variant of this.

**Name of Variant**	**Alignment of Translated Protein**	**Size (aa)**	**# Var aa**
KanR-009	MSHIQRETSCSRPRLNSNMDADLYGYKWARDNVGQSGATIYRLYGKPDAPELFLKHGKGSVANDVTDEMVRLNWLTEFMPLPTIKHFIRTPDDAWLLTTAIPGKTAFQVLEEYPDSGENI	271	0
KanR-021	MG--KEKTHVSRPRLNSNMDADLYGYKWARDNVGQSGATIYRLYGKPDAPELFLKHGKGSVANDVTDEMVRLNWLTEFMPLPTIKHFIRTPDDAWLLTTAIPGKTAFQVLEEYPDSGENI	269	6/2d
KanR-005	MSHIQRETSCSRPRLNSNMDADLYGYKWARDNVGQSGATIYRLYGKPDAPELFLKHGKGSVANDVTDEMVRLNWLTEFMPLPTIKHFIRTPDDAWLLTTAIPGKTAFQVLEEYPDSGENI	271	0
KanR-010	MSHIQRETSCSRPRLNSNMDADLYGYKWARDNVGQSGATIYRLYGKPDAPELFLKHGKGSVANDVTDEMVRLNWLTEFMPLPTIKHFIRTPDDAWLLTTAIPGKTAFQVLEEYPDSGENI	271	0
KanR-015	MSHIQRETSCSRPRLNSNMDADLYGYKWARDNVGQSGATIYRLYGKPDAPELFLKHGKGSVANDVTDEMVRLNWLTEFMPLPTIKHFIRTPDDAWLLTTAIPGKTAFQVLEEYPDSGENI	271	0
KanR-008	MSHIQRETSCSRPRLNSNMDADLYGYKWARDNVGQSGATIYRLYGKPDAPELFLKHGKGSVANVVTDEMVRLNWLTEFMPLPTIKHFIRTPDDAWLLTTAIPGKTAFQVLEEYPDSGENI	271	1
KanR-019	MSHIQRETSCSRPRLNSNMDADLYGYKWARDNVGQSGATIYRLYGKPDAPELFLKHGKGSVANDVTDEMVRLNWLTEFMPLPTIKHFIRTPDDAWLLTTAIPGKTAFQVLEEYPDSGENI	271	0
KanR-014	MSHIQRETSCSRPRLNSNMDADLYGYKWARDNVGQSGATIYRLYGKPDAPELFLKHGKGSVANDVTDEMVRLNWLTEFMPLPTIKHFIRTPDDAWLLTTAIPGKTAFQVLEEYPDSGENI	271	2
KanR-001	MSHIQRETSCSRPRLNSNMDADLYGYKWARDNVGQSGATIYGLYGKPDAPELFLKHGKGSVANDVTDEMVRLNWLTEFMPLPTIKHFIRTPDDAWLLTTAIPGKTAFQVLEEYPDSGENI	271	1
KanR-002	MSHIQRETSCSRPRLNSNMDADLYGYKWARDNVGQSGATIYRLYGKPDAPELFLKHGKGSVANDVTDEMVRLNWLTEFMPLPTIKHFIRTPDDAWLLTTAIPGKTAFQVLEEYPDSGENI	271	2
KanR-003	MSHIQRETSCSRPRLNSNMDADLYGYKWARDNVGQSGATIYRLYGKPDAPELFLKHGKGSVANDVTDEMVRLNWLTEFMPLPTIKHFIRTPDDAWLLTTAIPRKTAFQVLEEYPDSGENI	271	1
KanR-004	MSHIQRET--SRPRLNSNMDADLYGYKWARDNVGQSGATIYRLYGKPDAPELFLKHGKGSVANDVTDEMVRLNWLTEFMPLPTIKHFIRTPDDAWLLTTAIPGKTAFQVLEEYPDSGENI	269	2d
KanR-006	MSHIQRETSCSRPRLNSNMDADLYGYKWARDNVGQSGATIYRLYGKPDAPELFLKHGKGSVANDVTDEMVRLNWLTEFMPLPTIKHFIRTPDDAWLLTTAIPGKTAFQVLEEYPDSGENI	271	0
KanR-007	MSHIQRET--SRPRLNSNMDADLYGYKWARDNVGQSGATIYRLYGKPDAPELFLKHGKGSVANDVTDEMVRLNWLTEFMPLPTIKHFIRTPDDAWLLTTAIPGKTAFQVLEEYPDSGENI	269	2d
KanR-011	MSHIQRET--SRPRLNSNMDADLYGYKWARDNVGQSGATIYRLYGKPDAPELFLKHGKGSVANDVTDEMVRLNWLTEFMPLPTIKHFIRTPDDAWLLTTAIPGKTAFQVLEEYPDSGENI	269	2d
KanR-012	MSHIQRETSCSKPRLNSNMDADLYGYKWARDNVGQSGATIYRLYGKPDAPELFLKHGKGSVANDVTDEMVRLNWLTEFMPLPTIKHFIRTPDDAWLLTTAIPGKTAFQVLEEYPDSGENI	271	1
KanR-013	MSHIQRET--SRPRLNSNMDADLYGYKWARDNVGQSGATIYRLYGKPDAPELFLKHGKGSVANDVTDEMVRLNWLTEFMPLPTIKHFIRTPDDAWLLTTAIPGKTAFQVLEEYPDSGENI	269	1/2d
KanR-017	MSHIQRETSCSRPRLNSNMDADLYGYKWARDNVGQSGATIYRLYGKPDAPELFLKHGKGSVANDVTDEMVRLNWLTEFMPLPTIKHFIRTPDDAWLLTTAIPRKTAFQVLEEYPDSGENI	271	1
KanR-018	MSHIQRETSCSRPRLNSNMDADLYGYKWARDNVGQSGATIYRLYGKPDAPELFLKHGKGSVANDVTDEMVRLNWLTEFMPLPTIKHFIRTPDDAWLLTTAIPGKTAFQVLEEYPDSGENI	271	2
	. :.: *:***************************** ********************* ************************************** *****************		

KanR-009	VDALAVFLRRLHSIPVCNCPFNSDRVFRLAQAQSRMNNGLVDASDFDDERNGWPVEQVWKEMHKLLPFSPDSVVTHGDFSLDNLIF...PSLQKRLFQKYGIDNPDMNKLQFHLMLDEFF
KanR-021	VDALAVFLRRLHSIPVCNCPFNSDRVFRLAQAQSRMNNGLVDASDFDDERNGWPVEQVWKEMHKLLPFSPDSVVTHGDFSLDNLIF...PSLQKRLFQKYGIDNPDMNKLQFHLMLDEFF
KanR-005	VDALAVFLRRLHSIPVCNCPFNSDRVFRLAQAQSRMNNGLVDASDFDDERNGWPVEQVWKEMHKLLPFSPDSVVTHGDFSLDNLIF...PSLQKRLFQKYGIDNPDMNKLQFHLMLDEFF
KanR-010	VDALAVFLRRLHSIPVCNCPFNSDRVFRLAQAQSRMNNGLVDASDFDDERNGWPVEQVWKEMHKLLPFSPDSVVTHGDFSLDNLIF...PSLQKRLFQKYGIDNPDMNKLQFHLMLDEFF
KanR-015	VDALAVFLRRLHSIPVCNCPFNSDRVFRLAQAQSRMNNGLVDASDFDDERNGWPVEQVWKEMHKLLPFSPDSVVTHGDFSLDNLIF...PSLQKRLFQKYGIDNPDMNKLQFHLMLDEFF
KanR-008	VDALAVFLRRLHSIPVCNCPFNSDRVFRLAQAQSRMNNGLVDASDFDDERNGWPVEQVWKEMHKLLPFSPDSVVTHGDFSLDNLIF...PSLQKRLFQKYGIDNPDMNKLQFHLMLDEFF
KanR-019	VDALAVFLRRLHSIPVCNCPFNSDRVFRLAQAQSRMNNGLVDASDFDDERNGWPVEQVWKEMHKLLPFSPDSVVTHGDFSLDNLIF...PSLQKRLFQKYGIDNPDMNKLQFHLMLDEFF
KanR-014	VDALAVFLRRLHSIPVCNCPFNGDRVFRLAQAQSRMNNGLVGASDFDDERNGWPVEQVWKEMHKLLPFSPDSVVTHGDFSLDNLIF...PSLQKRLFQKYGIDNPDMNKLQFHLMLDEFF
KanR-001	VDALAVFLRRLHSIPVCNCPFNSDRVFRLAQAQSRMNNGLVDASDFDDERNGWPVEQVWKEMHKLLPFSPDSVVTHGDFSLDNLIF...PSLQKRLFQKYGIDNPDMNKLQFHLMLDEFF
KanR-002	VDALAVFLRRLHSIPVCNCPFNGDRVFRLAQAQSRMNNGLVGASDFDDERNGWPVEQVWKEMHKLLPFSPDSVVTHGDFSLDNLIF...PSLQKRLFQKYGIDNPDMNKLQFHLMLDEFF
KanR-003	VDALAVFLRRLHSIPVCNCPFNSDRVFRLAQAQSRMNNGLVDASDFDDERNGWPVEQVWKEMHKLLPFSPDSVVTHGDFSLDNLIF...PSLQKRLFQKYGIDNPDMNKLQFHLMLDEFF
KanR-004	VDALAVFLRRLHSIPVCNCPFNSDRVFRLAQAQSRMNNGLVDASDFDDERNGWPVEQVWKEMHKLLPFSPDSVVTHGDFSLDNLIF...PSLQKRLFQKYGIDNPDMNKLQFHLMLDEFF
KanR-006	VDALAVFLRRLHSIPVCNCPFNSDRVFRLAQAQSRMNNGLVDASDFDDERNGWPVEQVWKEMHKLLPFSPDSVVTHGDFSLDNLIF...PSLQKRLFQKYGIDNPDMNKLQFHLMLDEFF
KanR-007	VDALAVFLRRLHSIPVCNCPFNSDRVFRLAQAQSRMNNGLVDASDFDDERNGWPVEQVWKEMHKLLPFSPDSVVTHGDFSLDNLIF...PSLQKRLFQKYGIDNPDMNKLQFHLMLDEFF
KanR-011	VDALAVFLRRLHSIPVCNCPFNSDRVFRLAQAQSRMNNGLVDASDFDDERNGWPVEQVWKEMHKLLPFSPDSVVTHGDFSLDNLIF...PSLQKRLFQKYGIDNPDMNKLQFHLMLDEFF
KanR-012	VDALAVFLRRLHSIPVCNCPFNSDRVFRLAQAQSRMNNGLVDASDFDDERNGWPVEQVWKEMHKLLPFSPDSVVTHGDFSLDNLIF...PSLQKRLFQKYGIDNPDMNKLQFHLMLDEFF
KanR-013	VDALAVFLRRLHSIPVCNCPFNSGRVFRLAQAQSRMNNGLVDASDFDDERNGWPVEQVWKEMHKLLPFSPDSVVTHGDFSLDNLIF...PSLQKRLFQKYGIDNPDMNKLQFHLMLDEFF
KanR-017	VDALAVFLRRLHSIPVCNCPFNSDRVFRLAQAQSRMNNGLVDASDFDDERNGWPVEQVWKEMHKLLPFSPDSVVTHGDFSLDNLIF...PSLQKRLFQKYGIDNPDMNKLQFHLMLDEFF
KanR-018	VDALAVFLRRLHSIPVCNCPFNGDRVFRLAQAQSRMNNGLVGASDFDDERNGWPVEQVWKEMHKLLPFSPDSVVTHGDFSLDNLIF...PSLQKRLFQKYGIDNPDMNKLQFHLMLDEFF
	**********************..*****************.** *******************************

Key
ATGC = change in nucleotide relative to consensus sequence – synonymous
ATGC = change in nucleotide relative to consensus sequence – conservative/semi-conservative amino acid substitution
ATGC = change in nucleotide relative to consensus sequence – non-conservative amino acid substitution

Sequence Alignments

```
Name of        # of  Alignment                                                                                                                     Size     # Var    Sources*
Variant        Occur                                                                                                                               (bp)     bp
NeoR/KanR-013  135   ---------ATGATTGAACAAGATGGATTGCACGCAGGTTCTCCGGCCGCTTGGGTGGAGAGGCTATTCGGCTATGACTGGGCACAACAGACAATCGGCTGCTCTGATGCCGCCGTGTTC       795      2        Cl,Ev,In,Mo,Ag
NeoR/KanR-002  56    ---------ATGATTGAACAAGATGGATTGCACGCAGGTTCTCCGGCCGCTTGGGTGGAGAGGCTATTCGGCTATGACTGGGCACAACAGACAATCGGCTGCTCTGATGCCGCCGTGTTC       795      0        In,Si,Ne,Cl
NeoR/KanR-016  51    ---------ATGATTGAACAAGATGGATTGCACGCAGGTTCTCCGGCCGCTTGGGTGGAGAGGCTATTCGGCTATGACTGGGCACAACAGACAATCGGCTGCTCTGATGCCGCCGTGTTC       795      1        Or
NeoR/KanR-012  33    ---------ATGATTGAACAAGATGGATTGCACGCAGGTTCTCCGGCCGCTTGGGTGGAGAGGCTATTCGGCTATGACTGGGCACAACAGACAATCGGCTGCTCTGATGCCGCCGTGTTC       795      4        Ag
NeoR/KanR-017  23    ---------ATGATTGAACAAGATGGATTGCACGCAGGTTCTCCGGCGGCTTGGGTGGAGAGGCTATTCGGCTATGACTGGGCACAACAGACAATCGGCTGCTCTGATGCCGCCGTGTTC       795      12       Ox
NeoR/KanR-008  10    ---------ATGGCTGAACAAGATGGATTGCACGCAGGTTCTCCGGCCGCTTGGGTGGAGAGGCTATTCGGCTATGACTGGGCACAACAGACAATCGGCTGCTCTGATGCCGCCGTGTTC       795      2        Cl
NeoR/KanR-014  8     ------ATGGGGATTGAACAAGATGGATTGCACGCAGGTTCTCCGGCCGCTTGGGTGGAGAGGCTATTCGGCTATGACTGGGCACAACAGACAATCGGCTGCTCTGATGCCGCCGTGTTC       798      1        Ca,7919208
NeoR/KanR-022  6     ---------ATGATTGAACAAGATGGATTGCACGCAGGTTCTCCGGCCGCTTGGGTGGAGAGGCTATTCGGCTATGACTGGGCACAACAGACAATCGGCTGCTCTGATGCCGCCGTGTTC       795      2        Ra
NeoR/KanR-004  5     ---------ATGCTTGAACAAGATGGATTGCACGCAGGTTCTCCGGCCGCTTGGGTGGAGAGGCTATTCGGCTATGACTGGGCACAACAGACAATCGGCTGCTCTGATGCCGCCGTGTTC       795      7        16377204
NeoR/KanR-001  4     ATGGGATCGGCCATTGAACAAGATGGATTGCACGCAGGTTCTCCGGCCGCTTGGGTGGAGAGGCTATTCGGCTATGACTGGGCACAACAGACAATCGGCTGCTCTGATGCCGCCGTGTTC       804      4        No
NeoR/KanR-010  4     ------------ATTGAACAAGATGGATTGCACGCAGGTTCTCCGGCCGCTTGGGTGGAGAGGCTATTCGGCTATGACTGGGCACAACAGACAATCGGCTGCTCTGATGCCGCCGTGTTC       792      3d       Pr
NeoR/KanR-015  3     ---------ATGATTGAACAAGATGGATTGCACGCAGGTTCTCCGGCCGCTTGGGTGGAGAGGCTATTCGGCTATGACTGGGCACAACAGACAATCGGCTGCTCTGATGCCGCCGTGTTC       795      3        Or
NeoR/KanR-007  2     ---------ATGATTGAACAAGATGGATTGCACGCAGGTTCTCCGGCCGCTTGGGTGGAGAGGCTATTCGGCTATGACTGGGCACAACAGACAATCGGCTGCTCTGATGCCGCCGTGTTC       795      5        10890530
NeoR/KanR-009  2     ---------ATGATTGAACAAGATGGATTGCACGCAGGTTCTCCGGCCGCTTGGGTGGAGAGGCTATTCGGCTATGACTGGGCACAACAGACAATCGGCTGCTCTGATGCCGCCGTGTTC       795      1        6095209
NeoR/KanR-018  2     ---------ATGATTGAACAAGATGGATTGCACGCAGGTTCTCCGGCCGCTTGGGTGGAGAGGCTATTCGGCTATGACTGGGCACAACAGACAATCGGCTGCTCTGATGCCGCCGTGTTC       795      1        In
NeoR/KanR-020  2     ---------ATGATTGAACAAGATGGATTGCACGCAGGTTCTCCGGCCGCTTGGGTGGAGAGGCTATTCGGCTATGACTGGGCACAACAGACAATCGGCTGCTCTGATGCCGCCGTGTTC       795      3        Cl,CB
NeoR/KanR-023  2     ATGGGATCGGCCATTGAACAAGATGGATTGCACGCAGGTTCTCCGGCCGCTTGGGTGGAGAGGCTATTCGGCTATGACTGGGCACAACAGACAATCGGCTGCTCTGATGCCGCCGTGTTC       804      1        Cl
NeoR/KanR-003  1     ATGGGATCGGCCATTGAACAAGATGGATTGCACGCAGGTTCTCCGGCCGCTTGGGTGGAGAGGCTATTCGGCTATGACTGGGCACAACAGACAATCGGCTGCTCTGATGCCGCCGTGTTC       804      0        Cl
NeoR/KanR-005  1     ---------ATGATTGAACAAGATGGATTGCACGCAGGTTCTCCGGCCGCTTGGGTGGAGAGGCTATTCGGCTATGACTGGGCACAACAGACAATCGGCTGCTCTGATGCCGCCGTGTTC       795      2        Lu
NeoR/KanR-006  1     ---------ATGCTTGAACAAGATGGATTGCACGCAGGTTCTCCGGCCGCTTGGGTGGAGAGGCTATTCGGCTATGACTGGGCACAACAGACAATCGGCTGCTCTGATGCCGCCGTGTTC       795      1        Qi
NeoR/KanR-011  1     ---------ATGATTGAACAAGATGGATTGCACGCAGGTTCTCCGGCCGCTTGGGTGGAGAGGCTATTCGGCTATGACTGGGCACAACAGACAATCGGCTGCTCTGATGCCGCCGTGTTC       795      4        Or
NeoR/KanR-019  1     ---------ATGATTGAACAAGATGGATTGCACGCAGGTTCTCCGGCCGCTTGGGTGGAGAGGCTATTCGGCTATGACTGGGCACAACAGACAATCGGCTGCTCTGATGCCGCCGTGTTC       795      1        GB
NeoR/KanR-021  1     ---------ATGATTGAACAAGATGGATTGCACGCAGGTTCTCCGGCCGCTTGGGTGGAGAGGCTATTCGGCTATGACTGGGCACAACAGACAATCGGCTGCTCTGATGCCGCCGTGTTC       795      3        No
                                   ********************************* ************************************************************************


NeoR/KanR-013        CGGCTGTCAGCGCAGGGGCGCCCGGTTCTTTTTGTCAAGACCGACCTGTCCGGTGCCCTGAATGAACTGCAAGACGAGGCAGCGCGGCTATCGTGGCTGGCCACGACGGGCGTTCCTTGC
NeoR/KanR-002        CGGCTGTCAGCGCAGGGGCGCCCGGTTCTTTTTGTCAAGACCGACCTGTCCGGTGCCCTGAATGAACTGCAGGACGAGGCAGCGCGGCTATCGTGGCTGGCCACGACGGGCGTTCCTTGC
NeoR/KanR-016        CGGCTGTCAGCGCAGGGGCGCCCGGTTCTTTTTGTCAAGACCGACCTGTCCGGTGCCCTGAATGAACTGCAGGACGAGGCAGCGCGGCTATCGTGGCTGGCCACGACGGGCGTTCCTTGC
NeoR/KanR-012        CGGCTGTCAGCGCAGGGGCGCCCGGTTCTTTTTGTCAAGACCGACCTGTCCGGTGCCCTGAATGAACTGCAAGACGAGGCAGCGCGGCTATCGTGGCTGGCCACGACGGGCGTTCCTTGC
NeoR/KanR-017        CGGCTGTCAGCGCAGGGGCGTCCGGTTCTTTTTGTCAAGACCGACCTGTCCGGTGCCCTGAATGAACTGCAAGACGAGGCAGCGCGGCTATCGTGGCTGGCGACGACGGGCGTTCCTTGC
NeoR/KanR-008        CGGCTGTCAGCGCAGGGGCGCCCGGTTCTTTTTGTCAAGACCGACCTGTCCGGTGCCCTGAATGAACTGCAGGACGAGGCAGCGCGGCTATCGTGGCTGGCCACGACGGGCGTTCCTTGC
NeoR/KanR-014        CGGCTGTCAGCGCAGGGGCGCCCGGTTCTTTTTGTCAAGACCGACCTGTCCGGTGCCCTGAATGAACTGCAGGACGAGGCAGCGCGGCTATCGTGGCTGGCCACGACGGGCGTTCCTTGC
NeoR/KanR-022        CGGCTGTCAGCGCAGGGGCGCCCGGTTCTTTTTGTCAAGACCGACCTGTCCGGTGCCCTGAATGAACTGCAGGACGAGGCAGCGCGGCTATCGTGGCTGGCCACGACGGGCGTTCCTTGC
NeoR/KanR-004        CGGCTGTCAGCGCAGGGGCGCCCGGTTCTTTTTGTCAAGACCGACCTGTCCGGTGCCCTGAATGAACTGCAGGACGAGGCAGCGCGGCTATCGTGGCTGGCCACGACGGGCGTTCCTTGC
NeoR/KanR-001        CGGCTGTCAGCGCAGGGGCGCCCGGTTCTTTTTGTCAAGACCGACCTGTCCGGTGCCCTGAATGAACTGCAGGACGAGGCAGCGCGGCTATCGTGGCTGGCCACGACGGGCGTTCCTTGC
NeoR/KanR-010        CGGCTGTCAGCGCAGGGGCGCCCGGTTCTTTTTGTCAAGACCGACCTGTCCGGTGCCCTGAATGAACTGCAGGACGAGGCAGCGCGGCTATCGTGGCTGGCCACGACGGGCGTTCCTTGC
NeoR/KanR-015        CGGCTGTCAGCGCAGGGGCGCCCGGTTCTTTTTGTCAAGACCGACCTGTCCGGTGCCCTGAATGAACTGCAAGACGAGGCAGCGCGGCTATCGTGGCTGGCCACGACGGGCGTTCCTTGC
NeoR/KanR-007        CGGCTGTCAGCGCAGGGGCGCCCGGTTCTTTTTGTCAAGACCGACCTGTCCGGTGCCCTGAATGAACTCCAAGACGAGGCAGCGCGGCTATCGTGGCTGGCCACGACGGGCGTTCCTTGC
NeoR/KanR-009        CGGCTGTCAGCGCAGGGGCGCCCGGTTCTTTTTGTCAAGACCGACCTGTCCGGTGCCCTGAATGAACTGCAGGACGAGGCAGCGCGGCTATCGTGGCTGGCCACGACGGGCGTTCCTTGC
NeoR/KanR-018        CGGCTGTCAGCGCAGGGGCGCCCGGTTCTTTTTGTCAAGACCGACCTGTCCGGTGCCCTGAATGAACTGCAGGACGAGGCAGCGCGGCTATCGTGGCTGGCCACGACGGGCGTTCCTTGC
NeoR/KanR-020        CGGCTGTCAGCGCAGGGGCGCCCGGTTCTTTTTGTCAAGACCGACCTGTCCGGTGCCCTGAATGAACTGCAAGACGAGGCAGCGCGGCTATCGTGGCTGGCCACGACGGGCGTTCCTTGC
NeoR/KanR-023        CGGCTGTCAGCGCAGGGGCGCCCGGTTCTTTTTGTCAAGACCGACCTGTCCGGTGCCCTGAATGAACTGCAGGACGAGGCAGCGCGGCTATCGTGGCTGGCCACGACGGGCGTTCCTTGC
NeoR/KanR-003        CGGCTGTCAGCGCAGGGGCGCCCGGTTCTTTTTGTCAAGACCGACCTGTCCGGTGCCCTGAATGAACTGCAGGACGAGGCAGCGCGGCTATCGTGGCTGGCCACGACGGGCGTTCCTTGC
NeoR/KanR-005        CGGCTGTCAGCGCAGGGGCGCCCGGTTCTTTTTGTCAAGACCGACCTGTCCGGTGCCCTGAATGAACTGCAAGACGAGGCAGCGCGGCTATCGTGGCTGGCCACGACGGGCGTTCCTTGC
NeoR/KanR-006        CGGCTGTCAGCGCAGGGGCGCCCGGTTCTTTTTGTCAAGACCGACCTGTCCGGTGCCCTGAATGAACTGCAGGACGAGGCAGCGCGGCTATCGTGGCTGGCCACGACGGGCGTTCCTTGC
NeoR/KanR-011        CGGCTGTCAGCGCAGGGGCGCCCGGTTCTTTTTGTCAAGACCGACCTGTCCGGTGCCCTGAATGAACTGCAAGACGAGGCAGCGCGGCTATCGTGGCTGGCCACGACGGGCGTTCCTTGC
NeoR/KanR-019        CGGCTGTCAGCGCAGGGGCGCCCGGTTCTTTTTGTCAAGACCGACCTGTCCGGTGCCCTGAATGAACTGCAGGACGAGGCAGCGCGGCTATCGTGGCTGGCCACGACGGGCGTTCCTTGC
NeoR/KanR-021        CGGCTGTCAGCGCAGGGGCGCCCGGTTCTTTTTGTCAAGACCGACCTGTCCGGTGCCCTGAATGAACTGCAAGACGAGGCAGCGCGGCTATCGTGGCTGGCCACGACGGGCGTTCCTTGC
                     ******************** *********************************************** ** ***************************** ******************
```

Sequence Alignments

```
NeoR/KanR-013        GCAGCTGTGCTCGACGTTGTCACTGAAGCGGGAAGGGACTGGCTGCTATTGGGCGAAGTGCCGGGGCAGGATCTCCTGTCATCTCACCTTGCTCCTGCCGAGAAAGTATCCATCATGGCT
NeoR/KanR-002        GCAGCTGTGCTCGACGTTGTCACTGAAGCGGGAAGGGACTGGCTGCTATTGGGCGAAGTGCCGGGGCAGGATCTCCTGTCATCTCACCTTGCTCCTGCCGAGAAAGTATCCATCATGGCT
NeoR/KanR-016        GCAGCTGTGCTCGACGTTGTCACTGAAGCGGGAAGGGACTGGCTGCTATTGGGCGAAGTGCCGGGGCAGGATCTCCTGTCATCTCACCTTGCTCCTGCCGAGAAAGTATCCATCATGGCT
NeoR/KanR-012        GCAGCTGTGCTCGACGTTGTCACTGAAGCGGGAAGGGACTGGCTGCTATTGGGCGAAGTGCCGGGGCAGGATCTCCTGTCATCTCACCTTGCTCCTGCCGAGAAAGTATCCATCATGGCT
NeoR/KanR-017        GCGGCTGTGCTCGACGTTGTCACTGAAGCGGGAAGGGACTGGCTGCTATTGGGCGAAGTGCCGGGGCAGGATCTCCTGTCATCTCACCTTGCTCCTGCCGAGAAAGTATCCATCATGGCT
NeoR/KanR-008        GCAGCTGTGCTCGACGTTGTCACTGAAGCGGGAAGGGACTGGCTGCTATTGGGCGAAGTGCCGGGGCAGGATCTCCTGTCATCTCACCTTGCTCCTGCCGAGAAAGTATCCATCATGGCT
NeoR/KanR-014        GCAGCTGTGCTCGACGTTGTCACTGAAGCGGGAAGGGACTGGCTGCTATTGGGCGAAGTGCCGGGGCAGGATCTCCTGTCATCTCACCTTGCTCCTGCCGAGAAAGTATCCATCATGGCT
NeoR/KanR-022        GCAGCTGTGCTCGACGTTGTCACTGAAGCGGGAAGGGACTGGCTGCTATTGGGCGAAGTGCCGGGGCAGGATCTCCTGTCATCTCACCTTGCTCCTGCCGAGAAAGTATCCATCATGGCT
NeoR/KanR-004        GCAGCTGTGCTCGACGTTGTCACTGAAGCGGGAAGGGACTGGCTGCTATTGGGCGAAGTGCCGGGGCAGGATCTCCTGTCATCTCACCTTGCTCCTGCCGAGAAAGTATCCATCATGGCT
NeoR/KanR-001        GCAGCAGTGCTCGACGTTGTCACTGAAGCGGGAAGGGACTGGCTGCTATTGGGCGAAGTGCCGGGGCAGGATCTCCTGTCATCTCACCTTGCTCCTGCCGAGAAAGTATCCATCATGGCT
NeoR/KanR-010        GCAGCTGTGCTCGACGTTGTCACTGAAGCGGGAAGGGACTGGCTGCTATTGGGCGAAGTGCCGGGGCAGGATCTCCTGTCATCTCACCTTGCTCCTGCCGAGAAAGTATCCATCATGGCT
NeoR/KanR-015        GCAGCTGTGCTCGACGTTGTCACTGAAGCGGGAAGGGACTGGCTGCTATTGGGCGAAGTGCCGGGGCAGGATCTCCTGTCATCTCACCTTGCTCCTGCCGAGAAAGTATCCATCATGGCT
NeoR/KanR-007        GCAGCTGTGCTCGACGTTGTCACTGAAGCGGGAAGGGACTGGCTGCTATTGGGCGAAGTGCCGGGGCAGGATCTCCTGTCATCTCACCTTGCTCCTGCCGAGAAAGTATCCATCATGGCT
NeoR/KanR-009        GCAGCTGTGCTCGACGTTGTCACTGAAGCGGGAAGGGACTGGCTGCTATTGGGCGAAGTGCCGGGGCAGGATCTCCTGTCATCTCACCTTGCTCCTGCCGAGAAAGTATCCATCATGGCT
NeoR/KanR-018        GCAGCTGTGCTCGACGTTGTCACTGAAGCGGGAAGGGACTGGCTGCTATTGGGCGAAGTGCCGGGGCAGGATCTCCTGTCATCCCACCTTGCTCCTGCCGAGAAAGTATCCATCATGGCT
NeoR/KanR-020        GCAGCTGTGCTCGACGTTGTCACTGAAGCGGGAAGGGACTGGCTGCTATTGGGCGAAGTGCCGGGGCAGGATCTCCTGTCATCTCACCTTGCTCCTGCCGAGAAAGTATCCATCATGGCT
NeoR/KanR-023        GCAGCTGTGCTCGACGTTGTCACTGAAGCGGGAAGGGACTGGCTGCTATTGGGCGAAGTGCCGGGGCAGGATCTCCTGTCATCTCACCTTGCTCCTGCCGAGAAAGTATCCATCATGGCT
NeoR/KanR-003        GCAGCTGTGCTCGACGTTGTCACTGAAGCGGGAAGGGACTGGCTGCTATTGGGCGAAGTGCCGGGGCAGGATCTCCTGTCATCTCACCTTGCTCCTGCCGAGAAAGTATCCATCATGGCT
NeoR/KanR-005        GCAGCTGTGCTCGACGTTGTCACTGAAGCGGGAAGGGACTGGCTGCTATTGGGCGAAGTGCCGGGGCAGGATCTCCTGTCATCTCACCTTGCTCCTGCCGAGAAAGTATCCATCATGGCT
NeoR/KanR-006        GCAGCTGTGCTCGACGTTGTCACTGAAGCGGGAAGGGACTGGCTGCTATTGGGCGAAGTGCCGGGGCAGGATCTCCTGTCATCTCACCTTGCTCCTGCCGAGAAAGTATCCATCATGGCT
NeoR/KanR-011        GCAGCTGTGCTCGACGTTGTCACTGAAGCGGGAAGGGACTGGCTGCTATTGGGCGAAGTGCCGGGGCAGGATCTCCTGTCATCTCACCTTGCTCCTGCCGAGAAAGTATCCATCATGGCT
NeoR/KanR-019        GCAGCTGTGCTCGACGTTGTCACTGAAGCGGGAAGGGACTGGCTGCTATTGGGCGAAGTGCCGGGGCAGGATCTCCTGTCATCTCACCTTGCTCCTGCCGAGAAAGTATCCATCATGGCT
NeoR/KanR-021        GCAGCTGTGCTCGACGTTGTCACTGAAGCGGGAAGGGACTGGCTGCTATTGGGCGAAGTGCCGGGGCAGGATCTCCTGTCATCTCACCTTGCTCCTGCCGAGAAAGTATCCATCATGGCT
                     ** ** ***************************************************************************** ************************************

NeoR/KanR-013        GATGCAATGCGGCGGCTGCATACGCTTGATCCGGCTACCTGCCCATTCGACCACCAAGCGAAACATCGCATCGAGCGAGCACGTACTCGGATGGAAGCCGGTCTTGTCGATCAGGATGAT
NeoR/KanR-002        GATGCAATGCGGCGGCTGCATACGCTTGATCCGGCTACCTGCCCATTCGACCACCAAGCGAAACATCGCATCGAGCGAGCACGTACTCGGATGGAAGCCGGTCTTGTCGATCAGGATGAT
NeoR/KanR-016        GATGCAATGCGGCGGCTGCATACGCTTGATCCGGCTACCTGCCCATTCGACCACCAAGCGAAACATCGCATCGAGCGAGCACGTACTCGGATGGAAGCCGGTCTTGTCGATCAGGATGAT
NeoR/KanR-012        GATGCAATGCGGCGGCTGCATACGCTTGATCCGGCTACCTGCCCATTCGACCACCAAGCGAAACATCGCATCGAGCGAGCACGTACTCGGATGGAAGCCGGTCTTGTCGATCAGGATGAT
NeoR/KanR-017        GATGCAATGCGGCGGCTGCATACGCTTGATCCGGCTACCTGCCCATTCGACCACCAAGCGAAACATCGCATCGAGCGAGCACGTACTCGGATGGAAGCCGGTCTTGTCGATCAGGATGAT
NeoR/KanR-008        GATGCAATGCGGCGGCTGCATACGCTTGATCCGGCTACCTGCCCATTCGACCACCAAGCGAAACATCGCATCGAGCGAGCACGTACTCGGATGGAAGCCGGTCTTGTCGATCAGGATGAT
NeoR/KanR-014        GATGCAATGCGGCGGCTGCATACGCTTGATCCGGCTACCTGCCCATTCGACCACCAAGCGAAACATCGCATCGAGCGAGCACGTACTCGGATGGAAGCCGGTCTTGTCGATCAGGATGAT
NeoR/KanR-022        GATGCAATGCGGCGGCTGCATACGCTTGATCCGGCTACCTGCCCATTCGACCACCAAGCGAAACATCGCATCGAGCGAGCACGTACTCGGATGGAAGCCGGTCTTGTCGATCAGGATGAT
NeoR/KanR-004        GATGCAATGCGGCGGCTGCATACGCTTGATCCGGCTACCTGCCCATTCGACCACCAAGCGAAACATCGCATCGAGCGAGCACGCACTCGGATGGAAGCCGGTCTTGTCGATCAGGATGAT
NeoR/KanR-001        GATGCAATGCGGCGGCTGCATACGCTTGATCCGGCTACCTGCCCATTCGACCACCAAGCGAAACATCGCATCGAGCGAGCACGTACTCGGATGGAAGCCGGTCTTGTCGATCAGGATGAT
NeoR/KanR-010        GATGCAATGCGGCGGCTGCATACGCTTGATCCGGCTACCTGCCCATTCGACCACCAAGCGAAACATCGCATCGAGCGAGCACGTACTCGGATGGAAGCCGGTCTTGTCGATCAGGATGAT
NeoR/KanR-015        GATGCAATGCGGCGGCTGCATACGCTTGATCCGGCTACCTGCCCATTCGACCACCAAGCGAAACATCGCATCGAGCGAGCACGTACTCGGATGGAAGCCGGTCTTGTCGATCAGGATGAT
NeoR/KanR-007        GATGCAATGCGGCGGCTGCATACGCTTGATCCGGCTACCTGCCCATTCGACCACCAAGCGAAACATCGCATCGAGCGAGGACGTACTCGGATGGAAGCCGGTCTTGTCGATCAGGATGAT
NeoR/KanR-009        GATGCAATGCGGCGGCTGCATACGCTTGATCCGGCTACCTGCCCATTCGACCACCAAGCGAAACATCGCATCGAGCGAGCACGTACTCGGATGGAAGCCGGTCTTGTCGATCAGGATGAT
NeoR/KanR-018        GATGCAATGCGGCGGCTGCATACGCTTGATCCGGCTACCTGCCCATTCGACCACCAAGCGAAACATCGCATCGAGCGAGCACGTACTCGGATGGAAGCCGGTCTTGTCGATCAGGATGAT
NeoR/KanR-020        GATGCAATGCGGCGGCTGCATACGCTTGATCCGGCTACCTGCCCATTCGACCACCAAGCGAAACATCGCATCGAGCGAGCACGTACTCGGATGGAAGCCGGTCTTGTCGATCAGGATGAT
NeoR/KanR-023        GATGCAATGCGGCGGCTGCATACGCTTGATCCGGCTACCTGCCCATTCGACCACCAAGCGAAACATCGCATCGAGCGAGCACGTACTCGGATGGAAGCCGGTCTTGTCGATCAGGATGAT
NeoR/KanR-003        GATGCAATGCGGCGGCTGCATACGCTTGATCCGGCTACCTGCCCATTCGACCACCAAGCGAAACATCGCATCGAGCGAGCACGTACTCGGATGGAAGCCGGTCTTGTCGATCAGGATGAT
NeoR/KanR-005        GATGCAATGCGGCGGCTGCATACGCTTGATCCGGCTACCTGCCCATTCGACCACCAAGCGAAACATCGCATCGAGCGAGCACGTACTCGGATGGAAGCCGGTCTTGTCGATCAGGATGAT
NeoR/KanR-006        GATGCAATGCGGCGGCTGCATACGCTTGATCCGGCTACCTGCCCATTCGACCACCAAGCGAAACATCGCATCGAGCGAGCACGTACTCGGATGGAAGCCGGTCTTGTCGATCAGGATGAT
NeoR/KanR-011        GATGCAATGCGGCGGCTGCATACGCTTGATCCGGCTACCTGCCCATTCGACCACCAAGCGAAACATCGCATCGAGCGAGCACGTACTCGGATGGAAGCCGGTCTTGTCGATCAGGATGAT
NeoR/KanR-019        GATGCAATGCGGCGGCTGCATACGCTTGATCCGGCTACCTGCCCATTCGACCACCAAGCGAAACATCGCATCGAGCGAGCACGTACTCGGATGGAAGCCGGTCTTGTCGATCAGGATGAT
NeoR/KanR-021        GATGCAATGCGGCGGCTGCATACGCTTGATCCGGCTACCTGCCCATTCGACCACCAAGCGAAACATCGCATCGAGCGAGCACGTACTCGGATGGAAGCCGGTCTTGTCGATCAGGATGAT
                     ******************************************************************************* *** ************************************

NeoR/KanR-013        CTGGACGAAGAGCATCAGGGGCTCGCGCCAGCCGAACTGTTCGCCAGGCTCAAGGCGAGCATGCCCGACGGCGAGGATCTCGTCGTGACCCATGGCGATGCCTGCTTGCCGAATATCATG
NeoR/KanR-002        CTGGACGAAGAGCATCAGGGGCTCGCGCCAGCCGAACTGTTCGCCAGGCTCAAGGCGCGCATGCCCGACGGCGAGGATCTCGTCGTGACCCATGGCGATGCCTGCTTGCCGAATATCATG
NeoR/KanR-016        CTGGACGAAGAGCATCAGGGGCTCGCGCCAGCCGAACTGTTCGCCAGGCTCAAGGCGCGCATGCCCGACGGCGAGGATCTCGTCGTGACCCATGGCGATGCCTGCTTGCCGAATATCATG
NeoR/KanR-012        CTGGACGAAGAACATCAGGGGCTCGCGCCAGCCGAACTGTTCGCCAGGCTCAAGGCGAGCATGCCCGACGGCGAGGATCTCGTCGTGACCCATGGCGATGCCTGCTTGCCGAATATCATG
NeoR/KanR-017        CTGGACGAAGAGCATCAGGGGCTCGCGCCAGCCGAACTGTTCGCCAGGCTCAAGGCGTCTATGCCCGACGGCGAGGATCTCGTCGTGACCCACGGCGATGCCTGCTTGCCGAATATCATG
NeoR/KanR-008        CTGGACGAAGAGCATCAGGGGCTCGCGCCAGCCGAACTGTTCGCCAGGCTCAAGGCGCGCATGCCCGACGGCGAGGATCTCGTCGTGACCCATGGCGATGCCTGCTTGCCGAATATCATG
NeoR/KanR-014        CTGGACGAAGAGCATCAGGGGCTCGCGCCAGCCGAACTGTTCGCCAGGCTCAAGGCGCGCATGCCCGACGGCGAGGATCTCGTCGTGACACATGGCGATGCCTGCTTGCCGAATATCATG
NeoR/KanR-022        CTGGACGAAGAGCATCAGGGGCTCGCGCCAGCCGAACTGTTCGCCAGGCTCAAGGCGCGTATGCCCGACGGCGATGATCTCGTCGTGACCCATGGCGATGCCTGCTTGCCGAATATCATG
NeoR/KanR-004        CTGGACGAAGAGCATCAGGGGCTCGCGCCAGCCGAACTGTTCGCCAGGCTCAAGGCGCGTATGCCGGATGGTGAGGATCTCGTCGTGACTCATGGCGATGCCTGCTTGCCGAATATCATG
NeoR/KanR-001        CTGGACGAAGAGCATCAGGGGCTCGCGCCAGCCGAACTGTTCGCCAGGCTCAAGGCGCGTATGCCCGACGGCGATGATCTCGTCGTGACTCATGGCGATGCCTGCTTGCCGAATATCATG
NeoR/KanR-010        CTGGACGAAGAGCATCAGGGGCTCGCGCCAGCCGAACTGTTCGCCAGGCTCAAGGCGCGCATGCCCGACGGCGAGGATCTCGTCGTGACCCATGGCGATGCCTGCTTGCCGAATATCATG
NeoR/KanR-015        CTGGACGAAGAGCATCAGGGGCTCGCGCCAGCCGAACTGTTCGCCAGGCTCAAGGCGAGCATGCCCGACGGCGAGGATCTCGTCGTGACCCATGGCGATGCCTGCTTGCCGAATATCATG
NeoR/KanR-007        CTGGACGAAGAGCATCAGGGGCTCGCGCCAGCCGAACTGTTCGCCAGGCTCAAGGCGCGGATGCCCGACGGCGAGGATCTCGTCGTGACCCAGGGCGATGCCTGCTTGCCGAATATCATG
NeoR/KanR-009        CTGGACGAAGAGCATCAGGGGCTCGCGCCAGCCGAACTGTTCGCCAGGCTCAAGGCGCGCATGCCCGACGGCGATGATCTCGTCGTGACCCATGGCGATGCCTGCTTGCCGAATATCATG
NeoR/KanR-018        CTGGACGAAGAGCATCAGGGGCTCGCGCCAGCCGAACTGTTCGCCAGGCTCAAGGCGCGCATGCCCGACGGCGAGGATCTCGTCGTGACCCATGGCGATGCCTGCTTGCCGAATATCATG
NeoR/KanR-020        CTGGACGAAGAGCATCAGGGGCTCGCGCCAGCCGAACTGTTCGCCAGGCTCAAGGCGAGCATGCCCGACGGCGAGGATCTCGTCGTGACTCATGGCGATGCCTGCTTGCCGAATATCATG
NeoR/KanR-023        CTGGACGAAGAGCATCAGGGGCTCGCGCCAGCCGAACTGTTCGCCAGGCTCAAGGCGCGCATGCCCGACGGCGATGATCTCGTCGTGACCCATGGCGATGCCTGCTTGCCGAATATCATG
NeoR/KanR-003        CTGGACGAAGAGCATCAGGGGCTCGCGCCAGCCGAACTGTTCGCCAGGCTCAAGGCGCGCATGCCCGACGGCGAGGATCTCGTCGTGACCCATGGCGATGCCTGCTTGCCGAATATCATG
NeoR/KanR-005        CTGGACGAAGAGCATCAGGGGCTCGCGCCAGCCGAACTGTTCGCCAGGCTCAAGGCGAGCATGCCCGACGGCGAGGATCTCGTCGTGACCCATGGCGATGCCTGCTTGCCGAATATCATG
NeoR/KanR-006        CTGGACGAAGAGCATCAGGGGCTCGCGCCAGCCGAACTGTTCGCCAGGCTCAAGGCGCGCATGCCCGACGGCGAGGATCTCGTCGTGACCCATGGCGATGCCTGCTTGCCGAATATCATG
NeoR/KanR-011        CTGGACGAAGAGCATCAGGGGCTCGCGCCAGCCGAACTGTTCGCCAGGCTCAAGGCGAGCATGCCCGACGGCGAGGATCTCGTCGTGACCCATGGCGATGCCTGCTTGCCGAATATCATG
NeoR/KanR-019        CTGGACGAAGAGCATCAGGGGCTCGCGCCAGCCGAACTGTTCGCCAGGCTCAAGGCGCGCATGCCCGACGGCGAGGATCTCGTCGTGACACATGGCGATGCCTGCTTGCCGAATATCATG
NeoR/KanR-021        CTGGACGAAGAGCATCAGGGGCTCGCGCCAGCCGAACTGTTCGCCAGGCTCAAGGCGAGCATGCCCGACGGCGAGGATCTCGTCGTGACGCATGGCGATGCCTGCTTGCCGAATATCATG
                     *********** ********************************************   ***** ** ** ** *************** ** ***************************
```

Sequence Alignments

```
NeoR/KanR-013       GTGGAAAATGGCCGCTTTTCTGGATTCATCGACTGTGGCCGGCTGGGTGTGGCGGACCGCTATCAGGACATAGCGTTGGCTACCCGTGATATTGCTGAAGAGCTTGGCGGCGAATGGGCT
NeoR/KanR-002       GTGGAAAATGGCCGCTTTTCTGGATTCATCGACTGTGGCCGGCTGGGTGTGGCGGACCGCTATCAGGACATAGCGTTGGCTACCCGTGATATTGCTGAAGAGCTTGGCGGCGAATGGGCT
NeoR/KanR-016       GTGGAAAATGGCCGCTTTTCTGGATTCATCGACTGTGGCCGGCTGGGTGTGGCCGACCGCTATCAGGACATAGCGTTGGCTACCCGTGATATTGCTGAAGAGCTTGGCGGCGAATGGGCT
NeoR/KanR-012       GTGGAAAATGGCCGCTTTTCTGGATTCATCGACTGTGGCCGGCTGGGTGTGGCGGACCGCTATCAGGACATAGCGTTGGCTACCCGTGATATTGCTGAAGAACTTGGCGGCGAATGGGCT
NeoR/KanR-017       GTGGAAAATGGCCGCTTTTCTGGATTCATCGACTGTGGCCGTCTGGGTGTGGCGGACCGCTATCAGGACATAGCGTTGGCTACCCGTGATATTGCTGAAGAGCTTGGCGGCGAATGGGCT
NeoR/KanR-008       GTGGAAAATGGCCGCTTTTCTGGATTCATCGACTGTGGCCGGCTGGGTGTGGCGGACCGCTATCAGGACATAGCGTTGGCTACCCGTGATATTGCTGAAGAGCTTGGCGGCGAATGGGCT
NeoR/KanR-014       GTGGAAAATGGCCGCTTTTCTGGATTCATCGACTGTGGCCGGCTGGGTGTGGCGGACCGCTATCAGGACATAGCGTTGGCTACCCGTGATATTGCTGAAGAGCTTGGCGGCGAATGGGCT
NeoR/KanR-022       GTGGAAAATGGCCGCTTTTCTGGATTCATCGACTGTGGCCGGCTGGGTGTGGCGGACCGCTATCAGGACATAGCGTTGGCTACCCGTGATATTGCTGAAGAGCTTGGCGGCGAATGGGCT
NeoR/KanR-004       GTGGAAAATGGCCGCTTTTCTGGATTCATCGACTGTGGCCGGCTGGGTGTGGCGGACCGCTATCAGGACATAGCGTTGGCTACCCGTGATATTGCTGAAGAGCTTGGCGGCGAATGGGCT
NeoR/KanR-001       GTGGAAAATGGCCGCTTTTCTGGATTCATCGACTGTGGCCGGCTGGGTGTGGCGGACCGCTATCAGGACATAGCGTTGGCTACCCGTGATATTGCTGAAGAGCTTGGCGGCGAATGGGCT
NeoR/KanR-010       GTGGAAAATGGCCGCTTTTCTGGATTCATCGACTGTGGCCGGCTGGGTGTGGCGGACCGCTATCAGGACATAGCGTTGGCTACCCGTGATATTGCTGAAGAGCTTGGCGGCGAATGGGCT
NeoR/KanR-015       GTGGAAAATGGCCGCTTTTCTGGATTCATCGACTGTGGCCGGCTGGGTGTGGCGGATCGCTATCAGGACATAGCGTTGGCTACCCGTGATATTGCTGAAGAGCTTGGCGGCGAATGGGCT
NeoR/KanR-007       GTGGAAAATGGCCGCTTTTCTGGATTCATCGACTGTGGCCGGCTGGGTGTGGCGGACCGCTATCAGGACATAGCGTTGGCTACCCGTGATATTGCTGAAGAGCTTGGCGGCGAATGGGCT
NeoR/KanR-009       GTGGAAAATGGCCGCTTTTCTGGATTCATCGACTGTGGCCGGCTGGGTGTGGCGGACCGCTATCAGGACATAGCGTTGGCTACCCGTGATATTGCTGAAGAGCTTGGCGGCGAATGGGCT
NeoR/KanR-018       GTGGAAAATGGCCGCTTTTCTGGATTCATCGACTGTGGCCGGCTGGGTGTGGCGGACCGCTATCAGGACATAGCGTTGGCTACCCGTGATATTGCTGAAGAGCTTGGCGGCGAATGGGCT
NeoR/KanR-020       GTGGAAAATGGCCGCTTTTCTGGATTCATCGACTGTGGCCGGCTGGGTGTGGCGGACCGCTATCAGGACATAGCGTTGGCTACCCGTGATATTGCTGAAGAGCTTGGCGGCGAATGGGCT
NeoR/KanR-023       GTGGAAAATGGCCGCTTTTCTGGATTCATCGACTGTGGCCGGCTGGGTGTGGCGGACCGCTATCAGGACATAGCGTTGGCTACCCGTGATATTGCTGAAGAGCTTGGCGGCGAATGGGCT
NeoR/KanR-003       GTGGAAAATGGCCGCTTTTCTGGATTCATCGACTGTGGCCGGCTGGGTGTGGCGGACCGCTATCAGGACATAGCGTTGGCTACCCGTGATATTGCTGAAGAGCTTGGCGGCGAATGGGCT
NeoR/KanR-005       GTGGAAAATGGCCGCTTTTCTGGATTCATCGACTGTGGCCGGCTGGGTGTGGCGGACCGCTATCAGGACATAGCGTTGGCTACCCGTGATATTGCTGAAGAGCTTGGCGGCGAATGGGCT
NeoR/KanR-006       GTGGAAAATGGCCGCTTTTCTGGATTCATCGACTGTGGCCGGCTGGGTGTGGCGGACCGCTATCAGGACATAGCGTTGGCTACCCGTGATATTGCTGAAGAGCTTGGCGGCGAATGGGCT
NeoR/KanR-011       GTGGAAAATGGCCGCTTTTCTGGATTCATCGACTGTGGCCGGCTGGGTATGGCGGATCGCTATCAGGACATAGCGTTGGCTACCCGTGATATTGCTGAAGAGCTTGGCGGCGAATGGGCT
NeoR/KanR-019       GTGGAAAATGGCCGCTTTTCTGGATTCATCGACTGTGGCCGGCTGGGTGTGGCGGACCGCTATCAGGACATAGCGTTGGCTACCCGTGATATTGCTGAAGAGCTTGGCGGCGAATGGGCT
NeoR/KanR-021       GTGGAAAATGGCCGCTTTTCTGGATTCATCGACTGTGGCCGGCTGGGTGTGGCGGACCGCTATCAGGACATAGCGTTGGCTACCCGTGATATTGCTGAAGAGCTTGGCGGCGAATGGGCT
                    ***************************************** ****** **** ** ******************************************** ******************


NeoR/KanR-013       GACCGCTTCCTCGTGCTTTACGGTATCGCCGCTCCCGATTCGCAGCGCATCGCCTTCTATCGCCTTCTTGACGAGTTCTTCTGA
NeoR/KanR-002       GACCGCTTCCTCGTGCTTTACGGTATCGCCGCTCCCGATTCGCAGCGCATCGCCTTCTATCGCCTTCTTGACGAGTTCTTCTGA
NeoR/KanR-016       GACCGCTTCCTCGTGCTTTACGGTATCGCCGCTCCCGATTCGCAGCGCATCGCCTTCTATCGCCTTCTTGACGAGTTCTTCTGA
NeoR/KanR-012       GACCGCTTCCTCGTGCTTTACGGTATCGCCGCTCCCGATTCGCAGCGCATCGCCTTCTATCGCCTTCTTGACGAGTTCTTCTGA
NeoR/KanR-017       GACCGCTTCCTTGTGCTTTACGGTATCGCCGCGCCCGATTCGCAGCGCATCGCCTTCTATCGCCTTCTTGACGAGTTCTTCTGA
NeoR/KanR-008       GACCGCTTCCTCGTGCTTTACGGTATCGCCGCTCCCGATTCGCAGCGCATCGCCTTCTATCGCCTTCTTGACGAGTTCTTCTGA
NeoR/KanR-014       GACCGCTTCCTCGTGCTTTACGGTATCGCCGCTCCCGATTCGCAGCGCATCGCCTTCTATCGCCTTCTTGACGAGTTCTTCTGA
NeoR/KanR-022       GACCGCTTCCTCGTGCTTTACGGTATCGCCGCTCCCGATTCGCAGCGCATCGCCTTCTATCGCCTTCTTGACGAGTTCTTCTGA
NeoR/KanR-004       GACCGCTTCCTCGTGCTTTACGGTATCGCCGCTCCCGATTCGCAGCGCATCGCCTTCTATCGCCTTCTTGACGAGTTCTTCTGA
NeoR/KanR-001       GACCGCTTCCTCGTGCTTTACGGTATCGCCGCTCCCGATTCGCAGCGCATCGCCTTCTATCGCCTTCTTGACGAGTTCTTCTGA
NeoR/KanR-010       GACCGCTTCCTCGTGCTTTACGGTATCGCCGCTCCCGATTCGCAGCGCATCGCCTTCTATCGCCTTCTTGACGAGTTCTTCTGA
NeoR/KanR-015       GACCGCTTCCTCGTGCTTTACGGTATCGCCGCTCCCGATTCGCAGCGCATCGCCTTCTATCGCCTTCTTGACGAGTTCTTCTGA
NeoR/KanR-007       GACCGCTTCCTCGTGCTTTACGGTATCGCCGCTCCCGATTCGCAGCGCATCGCCTTCTATCGCCTTCTTGACGAGTTCTTCTGA
NeoR/KanR-009       GACCGCTTCCTCGTGCTTTACGGTATCGCCGCTCCCGATTCGCAGCGCATCGCCTTCTATCGCCTTCTTGACGAGTTCTTCTGA
NeoR/KanR-018       GACCGCTTCCTCGTGCTTTACGGTATCGCCGCTCCCGATTCGCAGCGCATCGCCTTCTATCGCCTTCTTGACGAGTTCTTCTGA
NeoR/KanR-020       GACCGCTTCCTCGTGCTTTACGGTATCGCCGCTCCCGATTCGCAGCGCATCGCCTTCTATCGCCTTCTTGACGAGTTCTTCTGA
NeoR/KanR-023       GACCGCTTCCTCGTGCTTTACGGTATCGCCGCTCCCGATTCGCAGCGCATCGCCTTCTATCGCCTTCTTGACGAGTTCTTCTGA
NeoR/KanR-003       GACCGCTTCCTCGTGCTTTACGGTATCGCCGCTCCCGATTCGCAGCGCATCGCCTTCTATCGCCTTCTTGACGAGTTCTTCTGA
NeoR/KanR-005       GACCGCTTCCTCGTGCTTTACGGTATCGCCGCTCCCGATTCGCAGCGCATCGCCTTCTATCGCCTTCTTGACGAGTTCTTCTGA
NeoR/KanR-006       GACCGCTTCCTCGTGCTTTACGGTATCGCCGCTCCCGATTCGCAGCGCATCGCCTTCTATCGCCTTCTTGACGAGTTCTTCTGA
NeoR/KanR-011       GACCGCTTCCTCGTGCTTTACGGTATCGCCGCTCCCGATTCGCAGCGCATCGCCTTCTATCGCCTTCTTGACGAGTTCTTCTGA
NeoR/KanR-019       GACCGCTTCCTCGTGCTTTACGGTATCGCCGCTCCCGATTCGCAGCGCATCGCCTTCTATCGCCTTCTTGACGAGTTCTTCTGA
NeoR/KanR-021       GACCGCTTCCTCGTGCTTTACGGTATCGCCGCTCCCGATTCGCAGCGCATCGCCTTCTATCGCCTTCTTGACGAGTTCTTCTGA
                    *********** ******************** ***************************************************
```

NeoR/KanR is *aph(3')-II* (aminoglycoside 3'-phosphotransferase) aka *nptII* (neomycin phosphotransferase) from Tn5 transposon

NeoR/KanR-010 is identical to NeoR/KanR-002 but missing the START codon because it is in frame with hRLuc

Key
- ATGC = change in nucleotide relative to consensus sequence – synonymous
- ATGC = change in nucleotide relative to consensus sequence – conservative/semi-conservative amino acid substitution
- ATGC = change in nucleotide relative to consensus sequence – non-conservative amino acid substitution

Sequence Alignments

Name of Variant	Alignment of Translated Protein	Size (aa)	# Var aa
NeoR/KanR-013	---MIEQDGLHAGSPAAWVERLFGYDWAQQTIGCSDAAVFRLSAQGRPVLFVKTDLSGALNELQDEAARLSWLATTGVPCAAVLDVVTEAGRDWLLLGEVPGQDLLSSHLAPAEKVSIMA	264	1
NeoR/KanR-002	---MIEQDGLHAGSPAAWVERLFGYDWAQQTIGCSDAAVFRLSAQGRPVLFVKTDLSGALNELQDEAARLSWLATTGVPCAAVLDVVTEAGRDWLLLGEVPGQDLLSSHLAPAEKVSIMA	264	0
NeoR/KanR-016	---MIEQDGLHAGSPAAWVERLFGYDWAQQTIGCSDAAVFRLSAQGRPVLFVKTDLSGALNELQDEAARLSWLATTGVPCAAVLDVVTEAGRDWLLLGEVPGQDLLSSHLAPAEKVSIMA	264	0
NeoR/KanR-012	---MIEQDGLHAGSPAAWVERLFGYDWAQQTIGCSDAAVFRLSAQGRPVLFVKTDLSGALNELQDEAARLSWLATTGVPCAAVLDVVTEAGRDWLLLGEVPGQDLLSSHLAPAEKVSIMA	264	1
NeoR/KanR-017	---MIEQDGLHAGSPAAWVERLFGYDWAQQTIGCSDAAVFRLSAQGRPVLFVKTDLSGALNELQDEAARLSWLATTGVPCAAVLDVVTEAGRDWLLLGEVPGQDLLSSHLAPAEKVSIMA	264	1
NeoR/KanR-008	---MAEQDGLHAGSPAAWVERLFGYDWAQQTIGCSDAAVFRLSAQGRPVLFVKTDLSGALNELQDEAARLSWLATTGVPCAAVLDVVTEAGRDWLLLGEVPGQDLLSSHLAPAEKVSIMA	264	1
NeoR/KanR-014	--MGIEQDGLHAGSPAAWVERLFGYDWAQQTIGCSDAAVFRLSAQGRPVLFVKTDLSGALNELQDEAARLSWLATTGVPCAAVLDVVTEAGRDWLLLGEVPGQDLLSSHLAPAEKVSIMA	265	0
NeoR/KanR-022	---MIEQDGLHAGSPAAWVERLFGYDWAQQTIGCSDAAVFRLSAQGRPVLFVKTDLSGALNELQDEAARLSWLATTGVPCAAVLDVVTEAGRDWLLLGEVPGQDLLSSHLAPAEKVSIMA	264	1
NeoR/KanR-004	---MLEQDGLHAGSPAAWVERLFGYDWAQQTIGCSDAAVFRLSAQGRPVLFVKTDLSGALNELQDEAARLSWLATTGVPCAAVLDVVTEAGRDWLLLGEVPGQDLLSSHLAPAEKVSIMA	264	1
NeoR/KanR-001	MGSAIEQDGLHAGSPAAWVERLFGYDWAQQTIGCSDAAVFRLSAQGRPVLFVKTDLSGALNELQDEAARLSWLATTGVPCAAVLDVVTEAGRDWLLLGEVPGQDLLSSHLAPAEKVSIMA	267	1
NeoR/KanR-010	----IEQDGLHAGSPAAWVERLFGYDWAQQTIGCSDAAVFRLSAQGRPVLFVKTDLSGALNELQDEAARLSWLATTGVPCAAVLDVVTEAGRDWLLLGEVPGQDLLSSHLAPAEKVSIMA	263	1
NeoR/KanR-015	---MIEQDGLHAGSPAAWVERLFGYDWAQQTIGCSDAAVFRLSAQGRPVLFVKTDLSGALNELQDEAARLSWLATTGVPCAAVLDVVTEAGRDWLLLGEVPGQDLLSSHLAPAEKVSIMA	264	0
NeoR/KanR-007	---MIEQDGLHAGSPAAWVERLFGYDWAQQTIGCSDAAVFRLSAQGRPVLFVKTDLSGALNELQDEAARLSWLATTGVPCAAVLDVVTEAGRDWLLLGEVPGQDLLSSHLAPAEKVSIMA	264	0
NeoR/KanR-009	---MIEQDGLHAGSPAAWVERLFGYDWAQQTIGCSDAAVFRLSAQGRPVLFVKTDLSGALNELQDEAARLSWLATTGVPCAAVLDVVTEAGRDWLLLGEVPGQDLLSSHLAPAEKVSIMA	264	0
NeoR/KanR-018	---MIEQDGLHAGSPAAWVERLFGYDWAQQTIGCSDAAVFRLSAQGRPVLFVKTDLSGALNELQDEAARLSWLATTGVPCAAVLDVVTEAGRDWLLLGEVPGQDLLSSHLAPAEKVSIMA	264	0
NeoR/KanR-020	---MIEQDGLHAGSPAAWVERLFGYDWAQQTIGCSDAAVFRLSAQGRPVLFVKTDLSGALNELQDEAARLSWLATTGVPCAAVLDVVTEAGRDWLLLGEVPGQDLLSSHLAPAEKVSIMA	264	0
NeoR/KanR-023	MGSAIEQDGLHAGSPAAWVERLFGYDWAQQTIGCSDAAVFRLSAQGRPVLFVKTDLSGALNELQDEAARLSWLATTGVPCAAVLDVVTEAGRDWLLLGEVPGQDLLSSHLAPAEKVSIMA	267	0
NeoR/KanR-003	MGSAIEQDGLHAGSPAAWVERLFGYDWAQQTIGCSDAAVFRLSAQGRPVLFVKTDLSGALNELQDEAARLSWLATTGVPCAAVLDVVTEAGRDWLLLGEVPGQDLLSSHLAPAEKVSIMA	267	0
NeoR/KanR-005	---MIEQDGLHAGSPAAWVERLFGYDWAQQTIGCSDAAVFRLSAQGRPVLFVKTDLSGALNELQDEAARLSWLATTGVPCAAVLDVVTEAGRDWLLLGEVPGQDLLSSHLAPAEKVSIMA	264	0
NeoR/KanR-006	---MLEQDGLHAGSPAAWVERLFGYDWAQQTIGCSDAAVFRLSAQGRPVLFVKTDLSGALNELQDEAARLSWLATTGVPCAAVLDVVTEAGRDWLLLGEVPGQDLLSSHLAPAEKVSIMA	264	0
NeoR/KanR-011	---MIEQDGLHAGSPAAWVERLFGYDWAQQTIGCSDAAVFRLSAQGRPVLFVKTDLSGALNELQDEAARLSWLATTGVPCAAVLDVVTEAGRDWLLLGEVPGQDLLSSHLAPAEKVSIMA	264	0
NeoR/KanR-019	---MIEQDGLHAGSPAAWVERLFGYDWAQQTIGCSDAAVFRLSAQGRPVLFVKTDLSGALNELQDEAARLSWLATTGVPCAAVLDVVTEAGRDWLLLGEVPGQDLLSSHLAPAEKVSIMA	264	0
NeoR/KanR-021	---MIEQDGLHAGSPAAWVERLFGYDWAQQTIGCSDAAVFRLSAQGRPVLFVKTDLSGALNELQDEAARLSWLATTGVPCAAVLDVVTEAGRDWLLLGEVPGQDLLSSHLAPAEKVSIMA	264	0
	***		

NeoR/KanR-013	DAMRRLHTLDPATCPFDHQAKHRIERARTRMEAGLVDQDDLDEEHQGLAPAELFARLKASMPDGEDLVVTHGDACLPNIMVENGRFSGFIDCGRLGVAD...IAAPDSQRIAFYRLLDEFF
NeoR/KanR-002	DAMRRLHTLDPATCPFDHQAKHRIERARTRMEAGLVDQDDLDEEHQGLAPAELFARLKARMPDGEDLVVTHGDACLPNIMVENGRFSGFIDCGRLGVAD...IAAPDSQRIAFYRLLDEFF
NeoR/KanR-016	DAMRRLHTLDPATCPFDHQAKHRIERARTRMEAGLVDQDDLDEEHQGLAPAELFARLKARMPDGEDLVVTHGDACLPNIMVENGRFSGFIDCGRLGVAD...IAAPDSQRIAFYRLLDEFF
NeoR/KanR-012	DAMRRLHTLDPATCPFDHQAKHRIERARTRMEAGLVDQDDLDEEHQGLAPAELFARLKASMPDGEDLVVTHGDACLPNIMVENGRFSGFIDCGRLGVAD...IAAPDSQRIAFYRLLDEFF
NeoR/KanR-017	DAMRRLHTLDPATCPFDHQAKHRIERARTRMEAGLVDQDDLDEEHQGLAPAELFARLKASMPDGEDLVVTHGDACLPNIMVENGRFSGFIDCGRLGVAD...IAAPDSQRIAFYRLLDEFF
NeoR/KanR-008	DAMRRLHTLDPATCPFDHQAKHRIERARTRMEAGLVDQDDLDEEHQGLAPAELFARLKARMPDGEDLVVTHGDACLPNIMVENGRFSGFIDCGRLGVAD...IAAPDSQRIAFYRLLDEFF
NeoR/KanR-014	DAMRRLHTLDPATCPFDHQAKHRIERARTRMEAGLVDQDDLDEEHQGLAPAELFARLKARMPDGEDLVVTHGDACLPNIMVENGRFSGFIDCGRLGVAD...IAAPDSQRIAFYRLLDEFF
NeoR/KanR-022	DAMRRLHTLDPATCPFDHQAKHRIERARTRMEAGLVDQDDLDEEHQGLAPAELFARLKARMPDGDDLVVTHGDACLPNIMVENGRFSGFIDCGRLGVAD...IAAPDSQRIAFYRLLDEFF
NeoR/KanR-004	DAMRRLHTLDPATCPFDHQAKHRIERARTRMEAGLVDQDDLDEEHQGLAPAELFARLKARMPDGEDLVVTHGDACLPNIMVENGRFSGFIDCGRLGVAD...IAAPDSQRIAFYRLLDEFF
NeoR/KanR-001	DAMRRLHTLDPATCPFDHQAKHRIERARTRMEAGLVDQDDLDEEHQGLAPAELFARLKARMPDGDDLVVTHGDACLPNIMVENGRFSGFIDCGRLGVAD...IAAPDSQRIAFYRLLDEFF
NeoR/KanR-010	DAMRRLHTLDPATCPFDHQAKHRIERARTRMEAGLVDQDDLDEEHQGLAPAELFARLKARMPDGEDLVVTHGDACLPNIMVENGRFSGFIDCGRLGVAD...IAAPDSQRIAFYRLLDEFF
NeoR/KanR-015	DAMRRLHTLDPATCPFDHQAKHRIERARTRMEAGLVDQDDLDEEHQGLAPAELFARLKASMPDGEDLVVTHGDACLPNIMVENGRFSGFIDCGRLGVAD...IAAPDSQRIAFYRLLDEFF
NeoR/KanR-007	DAMRRLHTLDPATCPFDHQAKHRIERGRTRMEAGLVDQDDLDEEHQGLAPAELFARLKARMPDGEDLVVTQGDACLPNIMVENGRFSGFIDCGRLGVAD...IAAPDSQRIAFYRLLDEFF
NeoR/KanR-009	DAMRRLHTLDPATCPFDHQAKHRIERARTRMEAGLVDQDDLDEEHQGLAPAELFARLKARMPDGDDLVVTHGDACLPNIMVENGRFSGFIDCGRLGVAD...IAAPDSQRIAFYRLLDEFF
NeoR/KanR-018	DAMRRLHTLDPATCPFDHQAKHRIERARTRMEAGLVDQDDLDEEHQGLAPAELFARLKARMPDGEDLVVTHGDACLPNIMVENGRFSGFIDCGRLGVAD...IAAPDSQRIAFYRLLDEFF
NeoR/KanR-020	DAMRRLHTLDPATCPFDHQAKHRIERARTRMEAGLVDQDDLDEEHQGLAPAELFARLKASMPDGEDLVVTHGDACLPNIMVENGRFSGFIDCGRLGVAD...IAAPDSQRIAFYRLLDEFF
NeoR/KanR-023	DAMRRLHTLDPATCPFDHQAKHRIERARTRMEAGLVDQDDLDEEHQGLAPAELFARLKARMPDGDDLVVTHGDACLPNIMVENGRFSGFIDCGRLGVAD...IAAPDSQRIAFYRLLDEFF
NeoR/KanR-003	DAMRRLHTLDPATCPFDHQAKHRIERARTRMEAGLVDQDDLDEEHQGLAPAELFARLKARMPDGEDLVVTHGDACLPNIMVENGRFSGFIDCGRLGVAD...IAAPDSQRIAFYRLLDEFF
NeoR/KanR-005	DAMRRLHTLDPATCPFDHQAKHRIERARTRMEAGLVDQDDLDEEHQGLAPAELFARLKASMPDGEDLVVTHGDACLPNIMVENGRFSGFIDCGRLGVAD...IAAPDSQRIAFYRLLDEFF
NeoR/KanR-006	DAMRRLHTLDPATCPFDHQAKHRIERARTRMEAGLVDQDDLDEEHQGLAPAELFARLKARMPDGEDLVVTHGDACLPNIMVENGRFSGFIDCGRLGVAD...IAAPDSQRIAFYRLLDEFF
NeoR/KanR-011	DAMRRLHTLDPATCPFDHQAKHRIERARTRMEAGLVDQDDLDEEHQGLAPAELFARLKASMPDGEDLVVTHGDACLPNIMVENGRFSGFIDCGRLGMAD...IAAPDSQRIAFYRLLDEFF
NeoR/KanR-019	DAMRRLHTLDPATCPFDHQAKHRIERARTRMEAGLVDQDDLDEEHQGLAPAELFARLKARMPDGEDLVVTHGDACLPNIMVENGRFSGFIDCGRLGVAD...IAAPDSQRIAFYRLLDEFF
NeoR/KanR-021	DAMRRLHTLDPATCPFDHQAKHRIERARTRMEAGLVDQDDLDEEHQGLAPAELFARLKASMPDGEDLVVTHGDACLPNIMVENGRFSGFIDCGRLGVAD...IAAPDSQRIAFYRLLDEFF
	**************************.******************************* ****:*****:*************************:** *******************

Key
ATGC = change in nucleotide relative to consensus sequence – synonymous
ATGC = change in nucleotide relative to consensus sequence – conservative/semi-conservative amino acid substitution
ATGC = change in nucleotide relative to consensus sequence – non-conservative amino acid substitution

Sequence Alignments

```
Name of       # of      Alignment                                                                                                                     Size    # Var   Sources*
Variant       Occur                                                                                                                                   (bp)    bp
PuroR-002     23        ATG---ACCGAGTACAAGCCCACGGTGCGCCTCGCCACCCGCGACGACGTCCCCAGGGCCGTACGCACCCTCGCCGCCGCGTTCGCCGACTACCCCGCCACGCGCCACACCGTCGATCCG    600     1       Cl,AG
PuroR-004     15        ATG---ACCGAGTACAAGCCCACGGTGCGCCTCGCCACCCGCGACGACGTCCCCAGGGCCGTACGCACCCTCGCCGCCGCGTTCGCCGACTACCCCGCCACGCGCCACACCGTCGATCCG    600     2       Th
PuroR-009     10        ATG---ACCGAGTACAAGCCCACGGTGCGCCTCGCCACCCGCGACGACGTCCCCCGGGCCGTACGCACCCTCGCCGCCGCGTTCGCCGACTACCCCGCCACGCGCCACACCGTCGACCCG    600     3       Cl
PuroR-006     7         ATG---ACCGAGTACAAGCCCACGGTGCGCCTCGCCACCCGCGACGACGTCCCCCGGGCCGTACGCACCCTCGCCGCCGCGTTCGCCGACTACCCCGCCACGCGCCACACCGTCGACCCG    600     1       Cl
PuroR-007     6         ATG---ACCGAGTACAAGCCTACCGTGCGCCTGGCCACTCGCGATGATGTGCCCCGCGCCGTCCGCACTCTGGCCGCCGCTTTCGCCGACTACCCCGCTACCCGGCACACCGTGGACCCC    600     106     Pr
PuroR-011     6         ATG---ACCGAGTACAAGCCCACGGTGCGCCTCGCCACCCGCGACGACGTCCCCAGGGCCGTACGCACCCTCGCCGCCGCGTTCGCCGACTACCCCGCCACGCGCCACACCGTCGATCCG    600     3       Or,2194165
PuroR-005     2         ATG---ACCGAGTACAAGCCCACGGTGCGCCTCGCCACCCGCGACGACGTCCCCCGGGCAGTACGCACCCTCGCCGCCGCGTTCGCCGACTACCCCGCCACGCGCCACACCGTCGATCCA    600     2       Or
PuroR-008     2         ATGGCCACCGAGTACAAGCCCACGGTGCGCCTCGCCACCCGCGACGACGTCCCCCGGGCCGTACGCACCCTCGCCGCCGCGTTCGCCGACTACCCCGCCACGCGCCACACCGTCGACCCG    603     6       OB
PuroR-010     2         ATG---ACCGAGTACAAGCCCACGGTGCGCCTCGCCACCCGCGACGACGTCCCCAGGGCCGTACGCACCCTCGCCGCCGCGTTCGCCGACTACCCCGCCACGCGCCACACCGTCGATCCG    600     2       21706014
PuroR-003     1         ATG---ACCGAGTACAAGCCCACGGTGCGCCTCGCCACCCGCGACGACGTCCCCAGGGCCGTACGCACCCTCGCCGCCGCGTTCGCCGACTACCCCGCCACGCGCCACACCGTCGATCCG    600     4       Or
PuroR-001     1         ATG---ACCGAGTACAAGCCCACGGTGCGCCTCGCCACCCGCGACGACGTCCCCCGGGCCGTACGCACCCTCGCCGCCGCGTTCGCCGACTACCCCGCCACGCGCCACACCGTCGACCCG    597     4       Cl
PuroR-001corr           ATG---ACCGAGTACAAGCCCACGGTGCGCCTCGCCACCCGCGACGACGTCCCCCGGGCCGTACGCACCCTCGCCGCCGCGTTCGCCGACTACCCCGCCACGCGCCACACCGTCGACCCG    624     1
                        ***   ************** ** ******** ***** ***** ** ** *** * ** ** ***** ** ******** ***************** ** ** ******** ** **

PuroR-002               GACCGCCACATCGAGCGGGTCACCGAGCTGCAAGAACTCTTCCTCACGCGCGTCGGGCTCGACATCGGCAAGGTGTGGGTCGCGGACGACGGCGCCGCGGTGGCGGTCTGGACCACGCCG
PuroR-004               GACCGCCACATCGAGCGGGTCACCGAGCTGCAAGAACTCTTCCTCACGCGCGTCGGGCTCGACATCGGCAAGGTGTGGGTCGCGGACGACGGCGCCGCGGTGGCGGTCTGGACCACGCCG
PuroR-009               GACCGCCACATCGAGCGGGTCACCGAGCTGCAAGAACTCTTCCTCACGCGCGTCGGGCTCGACATCGGCAAGGTGTGGGTCGCGGACGACGGCGCCGCGGTGGCGGTCTGGACCACGCCG
PuroR-006               GACCGCCACATCGAGCGGGTCACCGAGCTGCAAGAACTCTTCCTCACGCGCGTCGGGCTCGACATCGGCAAGGTGTGGGTCGCGGACGACGGCGCCGCGGTGGCGGTCTGGACCACGCCG
PuroR-007               GACCGGCACATCGAGCGTGTGACAGAGTTGCAGGAGCTGTTCCTGACCCGCGTCGGGCTGGACATCGGCAAGGTGTGGGTAGCCGACGACGGCGCGGCCGTGGCCGTGTGGACTACCCCC
PuroR-011               GACCGCCACATCGAGCGGGTCACCGAGCTGCAAGAACTCTTCCTCACGCGCGTCGGGCTCGACATCGGCAAGGTGTGGGTCGCGGACGACGGCGCCGCGGTGGCGGTCTGGACCACGCCG
PuroR-005               GACCGCCACATCGAGCGGGTCACCGAGCTGCAAGAACTCTTCCTCACGCGCGTCGGGCTCGACATCGGCAAGGTGTGGGTCGCGGACGACGGCGCCGCGGTGGCGGTCTGGACCACGCCG
PuroR-008               GACCGCCACATCGAGCGGGTCACCGAGCTGCAAGAACTCTTCCTCACGCGCGTCGGGCTCGACATCGGCAAGGTGTGGGTCGCGGACGACGGCGCCGCGGTGGCGGTCTGGACCACGCCG
PuroR-010               GACCGCCACATCGAGCGGGTCACCGAGCTGCAAGAACTCTTCCTCACGCGCGTCGGGCTCGACATCGGCAAGGTGTGGGTCGCGGACGACGGCGCCGCCGTGGCGGTCTGGACCACGCCG
PuroR-003               GACCGCCACATCGAGCGGGTCACCGAGCTGCAAGAACTCTTCCTCACGCGCGTCGGGCTCGACATCGGCAAGGTGTGGGTCGCGGACGACGGCGCCGCGGTGGCGGTCTGGACCACGCCG
PuroR-001               GACCGCCACATCGAGCGGGTCACCGAGCTGCAAGAACTCTTCCTCACGCGCGTCGGGCTCGACATCGGCAAGGTGTGGGTCGCGGACGACGGCGCCGCGGTGGCGGTCTGGACCACGCCG
PuroR-001corr           GACCGCCACATCGAGCGGGTCACCGAGCTGCAAGAACTCTTCCTCACGCGCGTCGGGCTCGACATCGGCAAGGTGTGGGTCGCGGACGACGGCGCCGCGGTGGCGGTCTGGACCACGCCG
                        ***** *********** ** ** *** **** ** ** ***** ** *********** ******************** ** *********** ** ***** ** ***** ** **

PuroR-002               GAGAGCGTCGAAGCGGGGGCGGTGTTCGCCGAGATCGGCCCGCGCATGGCCGAGTTGAGCGGTTCCCGGCTGGCCGCGCAGCAACAGATGGAAGGCCTCCTGGCGCCGCACCGGCCCAAG
PuroR-004               GAGAGCGTCGAAGCGGGGGCGGTGTTCGCCGAGATCGGCCCGCGCATGGCCGAGTTGAGCGGTTCCCGGCTGGCCGCGCAGCAACAGATGGAAGGCCTCCTGGCGCCGCACCGGCCCAAG
PuroR-009               GAGAGCGTCGAAGCGGGGGCGGTGTTCGCCGAGATCGGCCCGCGCATGGCCGAGTTGAGCGGTTCCCGGCTGGCCGCGCAGCAACAGATGGAAGGCCTCCTGGCGCCGCACCGGCCCAAG
PuroR-006               GAGAGCGTCGAAGCGGGGGCGGTGTTCGCCGAGATCGGCCCGCGCATGGCCGAGTTGAGCGGTTCCCGGCTGGCCGCGCAGCAACAGATGGAAGGCCTCCTGGCGCCGCACCGGCCCAAG
PuroR-007               GAGAGCGTTGAGGCCGGCGCCGTGTTCGCCGAGATCGGCCCCCGAATGGCCGAGCTGAGCGGCAGCCGCCTGGCCGCCCAGCAGCAAATGGAGGGCCTGCTTGCCCCCCATCGTCCCAAG
PuroR-011               GAGAGCGTCGAAGCGGGGGCGGTGTTCGCCGAGATCGGCCCGCGCATGGCCGAGTTGAGCGGTTCCCGGCTGGCCGCGCAGCAACAGATGGAAGGCCTCCTGGCGCCGCACCGGCCCAAG
PuroR-005               GAGAGCGTCGAAGCGGGGGCGGTGTTCGCCGAGATCGGCCCGCGCATGGCCGAGTTGAGCGGTTCCCGGCTGGCCGCGCAGCAACAGATGGAAGGCCTCCTGGCGCCGCACCGGCCCAAG
PuroR-008               GAGAGCGTCGAAGCGGGGGCGGTGTTCGCCGAGATCGGCTCGCGCATGGCCGAGTTGAGCGGTTCCCGGCTGGCCGCGCAGCAACAGATGGAAGGCCTCCTGGCGCCGCACCGGCCCAAG
PuroR-010               GAGAGCGTCGAAGCGGGGGCGGTGTTCGCCGAGATCGGCCCGCGCATGGCCGAGTTGAGCGGTTCCCGGCTGGCCGCGCAGCAACAGATGGAAGGCCTCCTGGCGCCGCACCGGCCCAAG
PuroR-003               GAGAGCGTCGAAGCGGGGGCGGTGTTCGCCGAGATCGGCCCGCGCATGGCCGAGTTGAGCGGTTCCCGGCTGGCCGCGCAGCAACAGATGGAAGGCCTCCTGGCGCCGCACCGGCCCAAG
PuroR-001               GAGAGCGTCGAAGCGGGGGCGGTGTTCGCCGAGATCGGCCCGCGCATGGCCGAGTTGAGCGGTTCCCGGCTGGCCGCGCAGCAACAGATGGAAGGCCTCCTGGCGCCGCACCGGCCCAAG
PuroR-001corr           GAGAGCGTCGAAGCGGGGGCGGTGTTCGCCGAGATCGGCCCGCGCATGGCCGAGTTGAGCGGTTCCCGGCTGGCCGCGCAGCAACAGATGGAAGGCCTCCTGGCGCCGCACCGGCCCAAG
                        ******** ** ** ** ** ****************** * ** ********* *******   *** ******** ***** ** ***** ***** ** ** ** ** ** ******

PuroR-002               GAGCCCGCGTGGTTCCTGGCCACCGTCGGCGTCTCGCCCGACCACCAGGGCAAGGGTCTGGGCAGCGCCGTCGTGCTCCCCGGAGTGGAGGCGGCCGAGCGCGCCGGGGTGCCCGCCTTC
PuroR-004               GAGCCCGCGTGGTTCCTGGCCACCGTCGGCGTCTCGCCCGACCACCAGGGCAAGGGTCTGGGCAGCGCCGTCGTGCTCCCCGGAGTGGAGGCGGCCGAGCGCGCCGGGGTGCCCGCCTTC
PuroR-009               GAGCCCGCGTGGTTCCTGGCCACCGTCGGCGTCTCGCCCGACCACCAGGGCAAGGGTCTGGGCAGCGCCGTCGTGCTCCCCGGAGTGGAGGCGGCCGAGCGCGCCGGGGTGCCCGCCTTC
PuroR-006               GAGCCCGCGTGGTTCCTGGCCACCGTCGGCGTCTCGCCCGACCACCAGGGCAAGGGTCTGGGCAGCGCCGTCGTGCTCCCCGGAGTGGAGGCGGCCGAGCGCGCCGGGGTGCCCGCCTTC
PuroR-007               GAGCCTGCCTGGTTTCTGGCCACTGTAGGAGTGAGCCCCGACCACCAGGGCAAGGGCTTGGGCAGCGCCGTCGTGTTGCCCGGCGTAGAGGCCGCCGAACGCGCCGGTGTGCCCGCCTTT
PuroR-011               GAGCCCGCGTGGTTCCTGGCCACCGTCGGCGTCTCGCCCGACCACCAGGGCAAGGGTCTGGGCAGCGCCGTCGTGCTCCCCGGAGTGGAGGCGGCCGAGCGCGCCGGGGTGCCCGCCTTC
PuroR-005               GAGCCCGCGTGGTTCCTGGCCACCGTCGGCGTCTCGCCCGACCACCAGGGCAAGGGTCTGGGCAGCGCCGTCGTGCTCCCCGGAGTGGAGGCGGCCGAGCGCGCCGGGGTGCCCGCCTTC
PuroR-008               GAGCCCGCGTGGTTCCTGGCCACCGTCGGCGTCTCGCCCGACCACCAGGGCAAGGGTCTGGGCAGCGCCGTCGTGCTCCCCGGAGTGGAGGCGGCCGAGCGCGCTGGGGTGCCCGCCTTC
PuroR-010               GAGCCCGCGTGGTTCCTGGCCACCGTCGGCGTCTCGCCCGACCACCAGGGCAAGGGTCTGGGCAGCGCCGTCGTGCTCCCCGGAGTGGAGGCGGCCGAGCGCGCCGGGGTGCCCGCCTTC
PuroR-003               GAGCCCGCGTGGTTCCTGGCCACCGTCGGCGTGTCGCCCGACCACCAGGGCAAGGGTCTGGGCAGCGCCGTCGTGCTCCCCGGAGTGGAGGCGGCCGAGCGCGCCGGGGTGCCCGCCTTC
PuroR-001               GAGCCCGCGTGGTTCCTGGCCACCGTCGGCGTCTCGCCCGACCACCAGGGCAAGGGTCTGGGCAGCGCCGTCGTGCTCCCCGGAGTGGAGGCGGCCGAGCGCGCCGGGGTGCCCGCCTTC
PuroR-001corr           GAGCCCGCGTGGTTCCTGGCCACCGTCGGCGTCTCGCCCGACCACCAGGGCAAGGGTCTGGGCAGCGCCGTCGTGCTCCCCGGAGTGGAGGCGGCCGAGCGCGCCGGGGTGCCCGCCTTC
                        ***** ** ***** ******** ** ** **    ********************  ***************** * ***** ** ***** ***** ***** ** ***********

PuroR-002               CTGGAGACCTCCGCGCCCCGCAACCTCCCCTTCTACGAGCGGCTCGGCTTCACCGTCACCGCCGACGTCGAGGTGCCCGAAGGACCGCGC-ACCTGGTGCATGACCCGCAAGCCCGGTGCCTGA------------------------
PuroR-004               CTGGAGACATCCGCGCCCCGCAACCTCCCCTTCTACGAGCGGCTCGGCTTCACCGTCACCGCCGACGTCGAGGTGCCCGAAGGACCGCGC-ACCTGGTGCATGACCCGCAAGCCCGGTGCCTGA------------------------
PuroR-009               CTGGAGACCTCCGCGCCCCGCAACCTCCCCTTCTACGAGCGGCTCGGCTTCACCGTCACCGCCGACGTCGAGGTGCCCGAAGGACCGCGC-ACCTGGTGCATGACCCGCAAGCCCGGTGCCTAG------------------------
PuroR-006               CTGGAGACCTCCGCGCCCCGCAACCTCCCCTTCTACGAGCGGCTCGGCTTCACCGTCACCGCCGACGTCGAGGTGCCCGAAGGACCGCGC-ACCTGGTGCATGACCCGCAAGCCCGGTGCCTGA------------------------
PuroR-007               CTCGAAACAAGCGCACCAAGAAACCTTCCATTCTACGAGCGCCTGGGCTTCACCGTGACCGCCGATGTCGAGGTGCCCGAGGGACCTAGG-ACCTGGTGTATGACACGAAAACCTGGCGCCTAA------------------------
PuroR-011               CTGGAGACCTCCGCGCCCCGCAACCTCCCCTTCTACGAGCGGCTCGGCTTCACCGTCACCGCCGACGTCGAG-TGCCCGAAGGACCGCGCGACCTGGTGCATGACCCGCAAGCCCGGTGCCTGA------------------------
PuroR-005               CTGGAGACCTCCGCGCCCCGCAACCTCCCCTTCTACGAGCGGCTCGGCTTCACCGTCACCGCCGACGTCGAGGTGCCCGAAGGACCGCGC-ACCTGGTGCATGACCCGCAAGCCCGGTGCCTGA------------------------
PuroR-008               CTGGAGACCTCCGCGCCCCGCAACCTCCCCTTCTACGAGCGGCTCGGCTTCACCGTCACCGCCGACGTCGAGGTGCCCGAAGGACCGCGC-ACCTGGTGCATGACCCGCAAGCCCGGTGCCTGA------------------------
PuroR-010               CTGGAGACCTCCGCGCCCCGCAACCTCCCCTTCTACGAGCGGCTCGGCTTCACCGTCACCGCCGACGTCGAGGTGCCCGAAGGACCGCGC-ACCTGGTGCATGACCCGCAAGCCCGGTGCCTGA------------------------
PuroR-003               CTGGAGACCTCCGCGCCCCGCAACCTCCCCTTCTACGAGCGGCTCGGCTTCACCGTCACCGCCGACGTCGAG-TGCCCGAAGGACCGCGCGACCTGGTGCATGACCCGCAAGCCCGGTGCCTGA------------------------
PuroR-001               CTGGAGACCTCCGCGCCCCGCAACCTCCCCTTCTACGAGCGGCTCGGCTTCACCGTCACCGCCGACGTCGAGGTGCCCGAAGGACCGCGC-ACCTGGTGCATGACCCGCAAGCCCGGTGCC---------------------------
PuroR-001corr           CTGGAGACCTCCGCGCCCCGCAACCTCCCCTTCTACGAGCGGCTCGGCTTCACCGTCACCGCCGACGTCGAGGTGCCCGAAGGACCGCGC-ACCTGGTGCATGACCCGCAAGCCCGGTGCCACCGGTGCGGCTTCGAGGATAAAATAA
                        ** ** **    *** **   * ***** ** *********** ** *********** ******** ****** ******* *****   *   ******** ***** ** ** ** ** ***
```

PuroR gene is *Streptomyces alboniger pac* (puromycin N-acetyltransferase)

Sequences in bold are incorrectly annotated

PuroR-001 missing STOP codon. Corrected sequence still unique variant

Sequence Alignments

```
Name of          Alignment of Translated Protein                                                                                                  Size     # Var
Variant                                                                                                                                           (aa)     aa
PuroR-002        M-TEYKPTVRLATRDDVPRAVRTLAAAFADYPATRHTVDPDRHIERVTELQELFLTRVGLDIGKVWVADDGAAVAVWTTPESVEAGAVFAEIGPRMAELSGSRLAAQQQMEGLLAPHRPK         199      0
PuroR-004        M-TEYKPTVRLATRDDVPRAVRTLAAAFADYPATRHTVDPDRHIERVTELQELFLTRVGLDIGKVWVADDGAAVAVWTTPESVEAGAVFAEIGPRMAELSGSRLAAQQQMEGLLAPHRPK         199      0
PuroR-009        M-TEYKPTVRLATRDDVPRAVRTLAAAFADYPATRHTVDPDRHIERVTELQELFLTRVGLDIGKVWVADDGAAVAVWTTPESVEAGAVFAEIGPRMAELSGSRLAAQQQMEGLLAPHRPK         199      0
PuroR-006        M-TEYKPTVRLATRDDVPRAVRTLAAAFADYPATRHTVDPDRHIERVTELQELFLTRVGLDIGKVWVADDGAAVAVWTTPESVEAGAVFAEIGPRMAELSGSRLAAQQQMEGLLAPHRPK         199      0
PuroR-007        M-TEYKPTVRLATRDDVPRAVRTLAAAFADYPATRHTVDPDRHIERVTELQELFLTRVGLDIGKVWVADDGAAVAVWTTPESVEAGAVFAEIGPRMAELSGSRLAAQQQMEGLLAPHRPK         199      0
PuroR-011        M-TEYKPTVRLATRDDVPRAVRTLAAAFADYPATRHTVDPDRHIERVTELQELFLTRVGLDIGKVWVADDGAAVAVWTTPESVEAGAVFAEIGPRMAELSGSRLAAQQQMEGLLAPHRPK         199      5
PuroR-005        M-TEYKPTVRLATRDDVPRAVRTLAAAFADYPATRHTVDPDRHIERVTELQELFLTRVGLDIGKVWVADDGAAVAVWTTPESVEAGAVFAEIGPRMAELSGSRLAAQQQMEGLLAPHRPK         199      0
PuroR-008        MATEYKPTVRLATRDDVPRAVRTLAAAFADYPATRHTVDPDRHIERVTELQELFLTRVGLDIGKVWVADDGAAVAVWTTPESVEAGAVFAEIGSRMAELSGSRLAAQQQMEGLLAPHRPK         200      2
PuroR-010        M-TEYKPTVRLATRDDVPRAVRTLAAAFADYPATRHTVDPDRHIERVTELQELFLTRVGLDIGKVWVADDGAAVAVWTTPESVEAGAVFAEIGPRMAELSGSRLAAQQQMEGLLAPHRPK         199      0
PuroR-003        M-TEYKPTVRLATRDDVPRAVRTLAAAFADYPATRHTVDPDRHIERVTELQELFLTRVGLDIGKVWVADDGAAVAVWTTPESVEAGAVFAEIGPRMAELSGSRLAAQQQMEGLLAPHRPK         199      5
PuroR-001corr    M-TEYKPTVRLATRDDVPRAVRTLAAAFADYPATRHTVDPDRHIERVTELQELFLTRVGLDIGKVWVADDGAAVAVWTTPESVEAGAVFAEIGPRMAELSGSRLAAQQQMEGLLAPHRPK         207      0
                 * *******************************************************************************************.**************************

PuroR-002        EPAWFLATVGVSPDHQGKGLGSAVVLPGVEAAERAGVPAFLETSAPRNLPFYERLGFTVTADVEVPEGPRTWCMTRKPGA--------
PuroR-004        EPAWFLATVGVSPDHQGKGLGSAVVLPGVEAAERAGVPAFLETSAPRNLPFYERLGFTVTADVEVPEGPRTWCMTRKPGA--------
PuroR-009        EPAWFLATVGVSPDHQGKGLGSAVVLPGVEAAERAGVPAFLETSAPRNLPFYERLGFTVTADVEVPEGPRTWCMTRKPGA--------
PuroR-006        EPAWFLATVGVSPDHQGKGLGSAVVLPGVEAAERAGVPAFLETSAPRNLPFYERLGFTVTADVEVPEGPRTWCMTRKPGA--------
PuroR-007        EPAWFLATVGVSPDHQGKGLGSAVVLPGVEAAERAGVPAFLETSAPRNLPFYERLGFTVTADVEVPEGPRTWCMTRKPGA--------
PuroR-011        EPAWFLATVGVSPDHQGKGLGSAVVLPGVEAAERAGVPAFLETSAPRNLPFYERLGFTVTADVECPKDRATWCMTRKPGA--------
PuroR-005        EPAWFLATVGVSPDHQGKGLGSAVVLPGVEAAERAGVPAFLETSAPRNLPFYERLGFTVTADVEVPEGPRTWCMTRKPGA--------
PuroR-008        EPAWFLATVGVSPDHQGKGLGSAVVLPGVEAAERAGVPAFLETSAPRNLPFYERLGFTVTADVEVPEGPRTWCMTRKPGA--------
PuroR-010        EPAWFLATVGVSPDHQGKGLGSAVVLPGVEAAERAGVPAFLETSAPRNLPFYERLGFTVTADVEVPEGPRTWCMTRKPGA--------
PuroR-003        EPAWFLATVGVSPDHQGKGLGSAVVLPGVEAAERAGVPAFLETSAPRNLPFYERLGFTVTADVECPKDRATWCMTRKPGA--------
PuroR-001corr    EPAWFLATVGVSPDHQGKGLGSAVVLPGVEAAERAGVPAFLETSAPRNLPFYERLGFTVTADVEVPEGPRTWCMTRKPGATGAASRIK
                 **************************************************************** *:.  **********
```

Sequence Alignments

Name of Variant	# of Occur	Alignment — Common Restriction Enzyme Sites in MCS	Size (bp)	MCS	Sources*
		HindIII KpnI XhoI SphI PstI EcoRV SalI XbaI			
lacZ-α-060	24	ATGACCATGATTAC--	234	207,328,061,183,195	Ca,7919298
lacZ-α-031	12	ATGACCATGATTACGCCAAGCGCGCAATTAACCCTCACTAAAGGGAACAAAAGCTGGAGCTCCACCGCGGTGGCGGCCGC------------------------------TCTAGA----	579	115,MCS 2-008	2659436
lacZ-α-034	4	ATGACCATGATTAC--	231	207,328,061,183,195	Ta,7919298
**lacZ-α-036**		**ATGACCATGATTACGCCAAGCTT---GCATGC------CTGCAG----------------GTCGACTCTAGA----**	**285**		
lacZ-α-057	4	ATGACCATGATTACGCCAAGCTT---GCATGC------CTGCAG----------------GTCGACTCTAGA----	324	207,328,061,183,195	3073106
lacZ-α-073	4	ATGACCATGATTACGCCAAGCTATTTAGGTGACACTATAGAATACTCAAGCTATGCATCCAACGCGTTACTCGAGCTCAAACATATGAAA--------------GTCGACCTGCAGG---	126	327	CB
lacZ-α-076	4	--	255		CB
lacZ-α-032	3	ATGACCATGATTACGCCAAGCTCGGAATTAACCCTCACTAAAGGGAACAAAAGCTGGAGCTCCACCGCGGTGGCGGCCGC------------------------------TCTAGA----	390	115,MCS 2-008	10890530
lacZ-α-068	3	ATGACCATGATTACGCCAAGCTATTTAGGTGAGACTATAGAATACTCAAGCT-TGCATGCGATACGTATCGTTAACGAT---	273		20040575
lacZ-α-001	2	ATGACCATGATTACGCCAAGCTT---GCATGC------CTGCAG----------------GTCGACTCTAGA----	309	207,328,061,183,195	33277753,8566803
lacZ-α-004	2	ATGACCATGATTAC--	327	207,183	Ta,3323803
lacZ-α-005	2	ATGACCATGATTACGCCAAGCGCGCAATTAACCCTCACTAAAGGGAACAAAAGCTGGAGCTCCACCGCGGTGGCGGCCGC------------------------------TCTAG-----	576	051,MCS 2-008	Ag
lacZ-α-006	2	ATGACCATGATTAC--	198	049,117,MCS1-008	Ca
lacZ-α-010	2	ATGACCATGATTACGCCAAGCGCGCAATTAACCCTCACTAAAGGGAACAAAAGCTGGGTACCGGGCCCCCCCTCGAGGTCGACGGTATCGATAAGCTTGATATC----------------	576	223,MCS2,MCS 2-008	Ag,2555794
lacZ-α-014	2	ATGACCATGATTACGCCAAGCT--CTAATACGACTCACTATAGGGAAAGCTCGGTACCACGCATGCTGCAGACGCGTTACGTATC-----------------------------------	381	077,3'MCS-2	No,Qi
lacZ-α-015	2	ATGACCATGATTACGCCAAGCGCGCAATTAACCCTCACTAAAGGGAACAAAAGCTGGAGCTCCACCGCGGTGGCGGCCGC------------------------------TCTAGA----	576	115,MCS 2-008	Ag,2555794
lacZ-α-020	2	ATGACCATGATTACGCCAAGCTT---GTCGA-----------	198		Lu
lacZ-α-022	2	ATGACCATGATTACGCCAAGCTT---GCATGCAGGCCTCTGCAGTCGACGGGCCCGGGATCCGATATCTAGA----	348	097	GE
lacZ-α-028	2	ATGACCATGATTACGCCAAGCTATTTAGGTGACACTATAGAATACTCAAGCTT-----------------GCATGC------CTGCAG----------------GTCGACTCTAGA----	381	328,061,195	Pr,Ne
lacZ-α-039	2	ATGACCATGATTAC--	303	328,061,195	3333305
lacZ-α-045	2	ATGACCATGATTAC--	291	328,061,195	3333305
lacZ-α-046	2	ATGACCATGATTACGCCAAGCT--CTAATACGACTCACTATAGGGAAAGCTCGGTACCACGCATGCTGCAGACGCGTTACGTATC-----------------------------------	198	149,261	Ca
lacZ-α-051	2	ATGACCATGATTACGCCAAGCT--CTAATACGACTCACTATAGGGAAAGCTCGGTACCACGCATGCTGCAGACGCGTTACGTATC-----------------------------------	366		In
lacZ-α-052	2+4	ATGACCATGATTACGCCAAGCTT---GCATGC------CTGCAG----------------GTCGACTCTAGA----	339	207,183	3073106
lacZ-α-036corr		ATGACCATGATTACGCCAAGCTT---GCATGC------CTGCAG----------------GTCGACTCTAGA----	339	207,183	3073106
lacZ-α-002	1	ATGACTATGATTACAACGCCCGGGCA--	261		No
lacZ-α-003	1	ATGACTATGATTACAACGCCCAACTTAATTAACATTAGTGGTGGTGGTGGTGGTGCTCGAGATCCTCTGGGTCTTCAGGAGCGAGTTCTGGCTGGCTTGCACGTGTATACAGCTGTGCGG	438	MCS 2-008	No
lacZ-α-007	1	ATGACCATGATTACG--AA----TTTAATACGACTCACTATAGGGAATTTAAATTTAATTAA-GGCGCGCCCATGGATCGATGTTAACATGCATGATATCACGCGTGGCGCCACTAGTGC	504		Mo
lacZ-α-008	1	ATGACCATGATTACGCCAAGCTT---GCATGC------CTGCAGGTTTAAACA-------GTCGACTCTAGA----	351	053	Ne
lacZ-α-009	1	ATGACCATGATTACGCCAAGCTT---GCATGC------CTGCAG----------------GTCGACTCTAGA----	231	328,061,195	Ta
lacZ-α-011	1	ATGACCATGATTACGCCAAGCTT---GCATGC------CTGCAG----------------GTCGACTCTAGA----	327	328,061,195	3323803
lacZ-α-012	1	ATGACCATGATTACGCCAAGCTCGGAATTAACCCTCACTAAAGGGAACAAAAGCTGGAGCTCCACCGCGGTGGCGGCCGC------------------------------TCTAGA----	363	115,MCS 2-008	10890530
lacZ-α-013	1	ATGACCATGATTAC--	324	207,183	2985470
lacZ-α-016	1	ATGACCATGATTACGCCAAGCTATTTAGGTGACGCGTTAGAATACTCAAGCTATGCATC-AAGCTTGGTACCGAGCTC--	360		In
lacZ-α-017	1	ATGACCATGATTACGCCAAGCTTGGTACCGAGCTC---	387	088	Ag
lacZ-α-018	1	ATGACCTTGATTACGCCAAGCTCGAAATTAACCCTCACTAAAGGGAACAAAAGCTGGAGCTCGCGCGC--------------CTGCAG----------------GTCGAC----------	549	091,3'MCS-2	No
lacZ-α-019	1	ATGACCATGATTACGCCAAGCTCGAAATTAACCCTCACTAAAGGGAACAAAAGCTGGGTACCGGGCCCCCCCTCGAGGTCGACGGTATCGATAAGCTTGATATC----------------	573	223,MCS2,MCS 2-008	2970625
lacZ-α-021	1	ATGACCATGATTACGCCAAGCTATTTAGGTGACACTATAGAATACTCAAGCTATGCATC-AAGCTTGGTACCGAGCTC--	360		In
lacZ-α-023	1+1	ATGACCATGATTACGCCAAGCTCGGAATTAACCCTCACTAAAGGGAACAAAAGCTGGAGCTCCACCGCGGTGGCGGCCGC------------------------------TCTAGA----	438	115,MCS 2-008	10890530
lacZ-α-041corr		ATGACCATGATTACGCCAAGCTCGGAATTAACCCTCACTAAAGGGAACAAAAGCTGGAGCTCCACCGCGGTGGCGGCCGC------------------------------TCTAGA----	342	115,MCS 2-008	10890530
lacZ-α-024	1	ATGACCATGATTACGCCAAGCT--CTAATACGACTCACTATAGGGA-AAGCTGGTACGC-----------------------CTGCAG--------------------------------	369		In
lacZ-α-025	1	ATGACCATGATTACGCCAAGCTCGAAATTAACCCTCACTAAAGGGAACAAAAGCTGGAGCTCCACCGCGGTGGCGGCCGC------------------------------TCTAGA----	573	115,MCS 2-008	2970625
lacZ-α-026	1	ATGACCATGATTACGCCAAGCT--CTAATACGACTCACTATAGGGAAAGCTCGGTACCACGCATGCTGCAGACGCGTTACGTATC-----------------------------------	351	077,3'MCS-2	No
lacZ-α-027	1	ATGACCATGATTACGCCAAGCTTGGTACCGAGCTC---	354		In
lacZ-α-029	1	ATGACCATGATTACGCCAAGCTATTTAGGTGACACTATAGAATACTCAAGCTATGCATC-AAGCTTGGTACCGAGCTC--	342	107,301,045	In
lacZ-α-030	1	ATGACCATGATTACGCCAAGCTT---GCATGC------CTGCAG----------------GTCGACTCTAGA----	324	328,061,195	6095209
lacZ-α-033	1	ATGACCATGATTACGCCAAGCT--CTAATACGACTCACTATAGGGA-AAGCTT-----------------GCATGC------CTGCAG----------------GTCGACTCTAGAGGAT	333	116	No
lacZ-α-035	1	ATGACCATGATTACGCCAAGCTCTAATACGACTCACTATAGGGAGACAAGCTT-----------------GCATGC------CTGCAG----------------GTCGACTCTAGA----	384		Pr
lacZ-α-037	1	ATGACCATGATTACGCCAAGCT--CTAATACGACTCACTATAGGGA-AAGCTGGTAC-----------------------GCCTGCAG--------------------------------	576	223,MCS2,MCS 2-008	
lacZ-α-038	1	ATGACCATGATTACGCCAAGCTACGTAATACGACTCACTAGTGGGTACCAGAGCTCCCTAGGTTCTAGAACCGGTGACGTCTCCCATGGTG-AAGCTT-----------------------	282		In
lacZ-α-040	1	ATGACCATGATTACGCCAAGCTATTTAGGTGACACTATAGAATACTCAAGCTATGCATCCAACGCGTTGGGAGCTCTC--CCATATG-----------------GTCGACCTGCAGG---	384		Pr
**lacZ-α-041**		**ATGACCATGATTACGCCAAGCTCGGAATTAACCCTCACTAAAGGGAACAAAAGCTGGAGCTCCACCGCGGTGGCGGCCGC------------------------------TCTAGA----**	**342**	**139,MCS 2-008**	**10890530**
lacZ-α-042	1	ATGACCATGATTACGCCAAGCTCGGAATTAACCCTCACTAAAGGGAACAAAAGCTGGAGCTCCACCGCGGTGGCGGCCGC------------------------------TCTAGA----	576	115,MCS 2-008	
lacZ-α-043	1	ATGACCATGATTACGCCAAGCTACGTAATACGACTCACTAGTGGG---	369	142,157	7669286
lacZ-α-044	1	ATGACCATGATTACGCCAAGCT-----------------GGCGCGCCAAGCTT-----------------GCATGC------CTGCAG----------------GTCGACTCTAGA----	351	141,207,183	7655517
lacZ-α-047	1	ATGACCATGATTACGCCAAGCTCGGAATTAACCCTCACTAAAGGGAACAAAAGCTGGAGCTCCACCGCGGTGGCGGCCGC------------------------------TCTAGA----	651	115,MCS 2-008	10890530
lacZ-α-048	1	ATGACCATGATTACGCCAAGCTATTTAGGTGACACTATAGAATACTCAAGCTATGCATCCAACGCGTTGGGAGCTCTC--CCATATG-----------------GTCGACCTGCAGG---	369		Pr
lacZ-α-049	1	ATGACCATGATTACGCCAAGCTTGGTACCGAGCTC---	513	328,061,195	
lacZ-α-050	1	ATGACCATGATTACGCCAAGCTACGTAATACGACTCACTATAGGG---	372	157	7669286
lacZ-α-053	1	ATGACCATGATTACGCCAAGCTATTTAGGTGACACTATAGAATACTCAAGCTATGCATC-AAGCTTGGTACCGAGCTC--	369	176,5' MCS-004	7669286
lacZ-α-054	1	ATGATTAC--	303	207,183	3327753
lacZ-α-055	1	ATGACCATGATTACGCCAAGCGCGCAATTAACCCTCACTAAAGGGAACAAAAGCTGGGTACCGGGCCCCCCCTCGAGGTCGACGGTATCGATAAGCTTGATATC----------------	390	184,MCS2,MCS 2-008	IMAGE
lacZ-α-056	1	ATGACCATGATTACGCCAAGCTACGTAATACGACTCACTAGTGGG-GCCCGTGCAATTGAAGCCGGCTGGCGCCAAGCTTCTCTGCAG----------GATATC----------------	363	209	7669286
lacZ-α-058	1	ATGACCATGATTACGCCAAGCTACGTAATACGACTCACTATAGGG-GCCCGTGCAATTGAAGCCGGCTGGCGCCAAGCTTCTCTGCAG----------GATATC----------------	363	209	7669286
lacZ-α-059	1	ATGACCATGATTACGCCAAGCTT---GCATGCCC----CTCGAGGG--------------GTCGACTCTAGA----	243	208	3327753
lacZ-α-061	1	ATGACCATGATTACGCCAAGCTACGTAATACGACTCACTAGTGGGTCGACC-------------------GCATGC--	366	224	7669286
lacZ-α-062	1	ATGACCATGATTACGCCAAGCTATTTAGGTGACACTATAGAATACTCAAGCTATGCATCCAACGCGTTGGGAGCTCTC--CCATATG-----------------GTCGACCTGCAGG---	121	229	CB
lacZ-α-063	1	ATGACCATGATTAC--	507	328,061,195	Ne
lacZ-α-064	1	--	263		CB
lacZ-α-065	1	ATGACCATGATTACGCCAAGCTT---GCATGC------CTGCAG----------------GTCGACTCTAGA----	507	207,183	Ne
lacZ-α-066	1	ATGACCATGATTACGCCAAGCTATTTAGGTGACACTATAGAATACTCAAGCTATGCATCCAACGCGTTGGGAGCTCTC--CCATATG-----------------GTCGACCTGCAGG---	115	268	CB
lacZ-α-067	1	ATGGCCATGATTAC--	270	328,061,195	Ta
lacZ-α-069	1	--	254		CB
lacZ-α-070	1	ATGACCATGATTACGCCAAGCT--CTAATACGACTCACTATAGGGA-AAGCTT-----------------GCATGC------CTGCAG----------------GTCGACTCTAGA----	267	328,061,195	Th
lacZ-α-071	1	ATGACCATGATTACGCCAAGCTATTTAGGTGACACTATAGAATACTCAAGCTATGCATC-AAGCTTGGTACCGAGCTC--	606		In
lacZ-α-072	1	ATGACCATGATTACGCCAAGCT--CTAATACGACTCACTATAGGGA-AAGCTT-----------------GCATGC------CTGCAG----------------GTCGACTCTAGA----	477	328,061,195	Th
lacZ-α-074	1	ATGACCATGATTACGCC---	309	328,061,195	8566803
lacZ-α-075	1	ATGACCATGATTACGCCAAGCT--CTAATACGACTCACTATAGGGA-AAGCTT-----------------GCATGCAGGCCTCTGCAG-----------------TCGAC----------	291	097	Th
		*** **			

Sequence Alignments

```
                                                                                            Common Restriction Enzyme Sites in MCS
                              SpeI  BamHI  SpeI  EagI SmaI KpnI SacI PstI  EcoRI BamHI AflII EcoRI PstI EcoRV HindIII EcoRI   SalI XhoI SmaI SpeI   BamHI  NotI         SacI  KpnI SmaI  XbaI BamHI
lacZ-α-060      -----------------------------------------------------------GAATTC---------------------------------------------------------------------------------------GAGCTCGGTACCCGGGGATCCTCTAGAG
lacZ-α-031      -------------ACTAGT-GGATCC---------------------CCCGGGCTGCAGGAATTC------------------------GATATCAAGCTTATCGATACCGTCGACCTCGAGGGGGGGCCCGGTA---------------------------------CCCAATTCGCCC
lacZ-α-034      -----------------------------------------------------------GAATTC---------------------------------------------------------------------------------------GAGCTCGGTACCCGGGGATCCTCTAGAG
lacZ-α-036      --------------------GGATCC------------------CCGGGTACCGAGCTCGAATTC-------------------------------------------------------------------------------------------------------------------
lacZ-α-057      --------------------GGATCC------------------CCGGGTACCGAGCTCGAATTC-------------------------------------------------------------------------------------------------------------------
lacZ-α-073      ------------------------------------------CGGCCGC-----------------------------------------------------------------ACTAGTGGATCCTGAT--------------------------------------------------
lacZ-α-076      -------------------------------------------TCAGGCCTGATCCGGAGAATTC--------------------------------------------------------------------------------------GTTTAAACCCATGGGGGCCCAATTCGCCC
lacZ-α-032      -------------ACTAGT-GGATCC---------------------CCCGGGCTGCAGGAATTC------------------------GATATCAAGCTTATCGATACCGTCGACCTCGAGGGGGGGCCCGGTA---------------------------------CCCAATTCGCCC
lacZ-α-068      --------------------GGATCC--------GACGCACGTGC--------------GAATTC---------------------------------------------------------------------------------------------------------------GCCC
lacZ-α-001      --------------------GGATCC------------------CCGGGTACCGAGCTCGAATTC-------------------------------------------------------------------------------------------------------------------
lacZ-α-004      -----------------------------------------------------------GAATTC---------------------------------------------------------------------------------------GAGCTCGGTACCCGGGGATCCTCTAGAG
lacZ-α-005      -------------CCCGGGCGGATCC---------------------CCCGGGCTGCAGGAATTC------------------------GATATCAAGCTTATCGATACCGTCGACCTCGAGGGGGGGCCCGGTA---------------------------------CCCAATTCGCCC
lacZ-α-006      -----------------------------------------------------------GAATTC--------------------------------------------------------------------------------------------------CCGGGGATCC------G
lacZ-α-010      -----------------------------------------------------------GAATTC-------------------CTGCAGCCCGGGGGATCCACTAGTTCTAGAGCGGCCGCCACCGCGGTGGAGC--------------------------------TCCAATTCGCCC
lacZ-α-014      --------------------GGATCC--------------------------------AGAATTC----------------------GTGATATCTGAATTCGTCGACAAGCTTCTCGAGCCTAGGCTAGCTCTAGACCACACGTGTGGGGGCCCGAGCTCGCGGCCGCTGTATTCTATA
lacZ-α-015      -------------ACTAGT-GGATCC---------------------CCCGGGCTGCAGGAATTC------------------------GATATCAAGCTTATCGATACCGTCGACCTCGAGGGGGGGCCCGGTA---------------------------------CCCAATTCGCCC
lacZ-α-020      ----------------------------------------------------------CGAATTC-------------------------------------------------------------------------------------------------------------------
lacZ-α-022      -------------------TGCATTC-----------------GCGAGGTACCGAGCTCGAATTC-------------------------------------------------------------------------------------------------------------------
lacZ-α-028      --------------------GGATCC------------------CCGGGTACCGAGCTCGAATTC---------------------------------------------------------------------------------------------------------------GCCC
lacZ-α-039      -----------------------------------------------------------GAATTC---------------------------------------------------------------------------------------GAGCTCGGTACCCGGGGATCCTCTAGAG
lacZ-α-045      -----------------------------------------------------------GAATTC---------------------------------------------------------------------------------------GAGCTCGGTACCCGGGGATCCTCTAGAG
lacZ-α-046      --------------------GGATCC--------------------------------AGAATTC----------------------GTGATATCTGAATTCGTCGACAAGCTTCTCGAGCCTAGGCTAGCTCTAGACCACACGTGTGGGGGCCCGAGCTCGCGGCCGC-----------
lacZ-α-051      --------------------GGATCC--------------------------------AGAATTC----------------------GTGATATCTGAATTCGTCGACAAGCTTCTCGAGCCTAGGCTAGCTCTAGACCACACGTGTGGGGGCCCGAGCTCGCGGCCGCTGTATTCTATA
lacZ-α-052      --------------------GGATCC------------------CCGGGTACCGAGCTCGAATTC-------------------------------------------------------------------------------------------------------------------
lacZ-α-036corr  --------------------GGATCC------------------CCGGGTACCGAGCTCGAATTC-------------------------------------------------------------------------------------------------------------------
lacZ-α-002      ----------------------------------------------------------GGAATTC------------------------GATATC-------------------------------------------------------TATATCTCCTTCTTAAAGTTAAACAAAATT
lacZ-α-003      CCGCAAGCTTCGAACGCGTATCGATGGTACCGTCGACGTCCTGCAGGCGCGCCTGTACAGAATTC------GGATCCACGAGCTCCCGGGATATC------------------------------------------------GCCATGGTATATCTCCTTCTTAAAGTTAAACAAAATT
lacZ-α-007      TAGCAGATC-----------TGGCCGGCCCACCGGTGGGCCATATGGGCCCGCGGCCGCGAATTCGAGCTCGGTACCTACGTACTCGAGTCGCGACGTACGTTCGAACAATTGGTTTAAACGCCCGGGCACGTGGGATCCTCTAGAGTCGACCTGCAGGCATGCAAGCTTCGGTCCGCCT
lacZ-α-008      -----------CTTAATTAAGGATCC---------GGCGCGCCCCCGGGTACCGAGCTCGAATTC-------------------------------------------------------------------------------------------------------------------
lacZ-α-009      --------------------GGATCC------------------CCGGGTACCGAGCTCGAATTC-------------------------------------------------------------------------------------------------------------------
lacZ-α-011      --------------------GGATCC------------------CCGGGTACCGAGCTCGAATTC-------------------------------------------------------------------------------------------------------------------
lacZ-α-012      -------------ACTAGT-GGATCC---------------------CCCGGGCTGCAGGAATTC------------------------GATATCAAGCTTATCGATACCGTCGACCTCGAGGGGGGGCCCGGTA---------------------------------CCCAATTCGCCC
lacZ-α-013      -----------------------------------------------------------GAATTC---------------------------------------------------------------------------------------GAGCTCGGTACCCGGGGATCCTCTAGAG
lacZ-α-016      --------------------GGATCC--------ACTAGTAACGGCCGCCAGTGTGCTGGAATTC--GCCCTTAAGGGCGAATTCTGCAGATATC-----------------------------------CATCACACTGGCGGCCGCTCGAGCATGCATCTAGAGGGCCCAATTCGCCC
lacZ-α-017      --------------------GGATCC--------ACTAGTAACGGCCGCCAGTGTGCTGGAATTC--GCCCTTAAGGGCGAATTCTGCAGATATC-----------------------------------CATCACACTGGCGGCCGCTCGAGCATGCATCTAGAGGGCCCAATTCGCCC
lacZ-α-018      -------------ACTAGT-GGATCC------------------------------AAAGAATTC------------------------AAAAAGCTTCTCGAGAGTACTTCTAGAGCGGCCGCGGGCCCATCGATTTTCCACCCGGGTGGGGTACCAGGTAAGTGTACCCAATTCGCCC
lacZ-α-019      -----------------------------------------------------------GAATTC-------------------CTGCAGCCCGGGGGATCCACTAGTTCTAGAGCGGCCGCCACCGCGGTGGAGC--------------------------------TCCAATTCGCCC
lacZ-α-021      --------------------GGATCC--------ACTAGTAACGGCCGCCAGTGTGCTGGAATTC--------------------TGCAGATATC-----------------------------------CATCACACTGGCGGCCGCTCGAGCATGCATCTAGAGGGCCCAATTCGCCC
lacZ-α-023      -------------ACTAGT-GGATCC---------------------CCCGGGCTGCAGGAATTC------------------------GATATCAAGCTTATCGATACCGTCGACCTCGAGGGGGGGCCCGGTA---------------------------------CCCAATTCGCCC
lacZ-α-041corr  -------------ACTAGT-GGATCC---------------------CCCGGGCTGCAGGAATTC------------------------GATATCAAGCTTATCGATACCGTCGACCTCGAGGGGGGGCCCGGTA---------------------------------CCCAATTCGCCC
lacZ-α-024      ----------------GTACCGGTCC--------------------------------GGAATTC------------------------------CCGGGGCGGCCGCTGTCACTAGTGAGCTCGTCGACTCTAGAGGATCCAAGCTTACGTACGCGTGCATGCGACGTCATAGCTCTTC
lacZ-α-025      -------------ACTAGT-GGATCC---------------------CCCGGGCTGCAGGAATTC------------------------GATATCAAGCTTATCGATACCGTCGACCTCGAGGGGGGGCCCGGTA---------------------------------CCCAATTCGCCC
lacZ-α-026      --------------------GGATCC--------------------------------AGAATTC----------------------GTGATATCTGAATTCGTCGACAAGCTTCTCGAGCCTAGGCTAGCTCTAGACCACACGTGTGGGGGCCCGAGCTCGCGGCCGCTGTATTCTATA
lacZ-α-027      --------------------GGATCC--------ACTAGTAACGGCCGCCAGTGTGCTGGAATTC--GCCCTTAAGGGCGAATTCTGCAGATATC-----------------------------------CATCACACTGGCGGCCGCTCGAGCATGCATCTAGAGGGCCCAATTCGCCC
lacZ-α-029      --------------------GGATCC--------ACTAGTAACGGCCGCCAGTGTGCTGGAATTC--------------------TGCAGATATC-----------------------------------CATCACACTGGCGGCCGCTCGAGCATGCATCTAGAGGGCCCAATTCGCCC
lacZ-α-030      --------------------GGATCC------------------CCGGGTACCGAGCTCGAATTC-------------------------------------------------------------------------------------------------------------------
lacZ-α-033      CTACTAGTCATATGGATATCGGATCC------------------CCGGGTACCGAGCTCGAATTC-------------------------------------------------------------------------------------------------------------------
lacZ-α-035      --------------------GGATCC------------------CCGGGTACCGAGCTCGAATTC-------------------------------------------------------------------------------------------------------------GTATTC
lacZ-α-037      ----------------GTACCGGTCC--------------------------------GGAATTC------------------------------CCGGGGCGGCCGCTGTCACTAGTGAGCTCGTCGACTCTAGAGGATCCAAGCTTACGTACGCGTGCATGCGACGTCATAGCTCTTC
lacZ-α-038      --------------------GGATCC-------------------ACGATATCCTGCAGGAATTC-------------------------------------------------------------CTCGAGACCGTACGTGCGCGCGAATGCATCCAGATCTTCCCTCTAGTCAAGGCC
lacZ-α-040      ------------------------------------------CGGCCGC----------GAATTC------------------ACTAGTGATATC-GAATTCCCGCGGCCGCCATGGCGGCCGGGAGCATGCGACGTCGGG---------------------------CCCAATTCGCCC
lacZ-α-041      -------------ACTAGT-GGATCC---------------------CCCGGGCTGCAGGAAT-C------------------------GATATCAAGCTTATCGATACCGTCGACCTCGAGGGGGGGCCCGGTA---------------------------------CCCAATTCGCCC
lacZ-α-042      -------------ACTAGT-GGATCC---------------------CCCGGGCTGCAGGAATTC------------------------GATATCAAGCTTATCGATACCGTCGACCTCGAGGGGGGGCCCGGTA---------------------------------CCCAATTCGCCC
lacZ-α-043      --------------------CAGATCTTCGAATGCATCGCGCGCACCGTACGTCTCGAGGAATTC------------------CTGCAGGATATCTGGATCCACGA---AGCTTCCCATGGTGACGTCACCGGTTCTAGATACCTAGGTGAGCTCTGGT----ACCCTCTAGTCAAGGCC
lacZ-α-044      --------------------GGATCC------------------CCGGGTACCGAGCTCGAATTC-------------------------------------------------------------------------------------------------------------------
lacZ-α-047      -------------ACTAGT-GGATCC---------------------CCCGGGCTGCAGGAATTC------------------------GATATCAAGCTTATCGATACCGTCGACCTCGAGGGGGGGCCCGGTA---------------------------------CCCAATTCGCCC
lacZ-α-048      ------------------------------------------CGGCCGC----------------------------------ACTAGTGATATC----------CCGCGGCCATGGCGGCCGGGAGCATGCGACGTCGGG---------------------------CCCAATTCGCCC
lacZ-α-049      --------------------GGATCC--------ACTAGTAACGGCCGCCAGTGTGCTGGAATTC--GCCCTTAAGGGCGAATTCTGCAGATATC-----------------------------------CATCACACTGGCGGCCGCTCGAGCATGCATCTAGAGGGCCCAATTCGCCC
lacZ-α-050      --------------------CAGATCTTCGAATGCATCGCGCGCACCGTACGTCTCGAGGAATTC-------------------CTGCAGGATATCTGGATCCACGA--AGCTTCCCATGGTGACGTCACCGGTTCTAGATACCTAGGTGAGCTCTGGT----ACCCTCTAGTCAAGGCC
lacZ-α-053      --------------------GGATCC--------ACTAGTAACGGCCGCCAGTGTGCTGGAATTC--GCCCTTAAGGGCGAATTCTGCAGATATC-----------------------------------CATCACACTGGCGGCCGCTCGAGCATGCATCTAGAGGGCCCAATTCGCCC
lacZ-α-054      -----------------------------------------------------------GAATTC---------------------------------------------------------------------------------------GAGCTCGGTACCCGGGGATCCTCTAGAG
lacZ-α-055      -----------------------------------------------------------GAATTC-------------------CTGCAGCCCAAATGATGGCCAACAAGGCCAAACAATGGATCCACTAGTTCTAGAGCGGCCGCCACCGCGGTGGAGC--------TCCAATTCGCCC
lacZ-α-056      -------------------TGGATCC-------------------------------ACGAATTC--------------------------------------GCTAGCTTCGGCCGTGACGCGTCTCCGGATGTACAGGCATGCGTCG--------------ACCCTCTAGTCAAGGCC
lacZ-α-058      -------------------TGGATCC-------------------------------ACGAATTC--------------------------------------GCTAGCTTCGGCCGTGACGCGTCTCCGGATGTACAGGCATGCGTCG--------------ACCCTCTAGTCAAGGCC
lacZ-α-059      --------------------GGATCC----------CCCATCGATGGGGTACCGAGCTCGAATTC-------------------------------------------------------------------------------------------------------------------
lacZ-α-061      -----------------------CTGTACACTCCGGAAGACGCGTTCGGCCGTGCTAGCGAATTC------------------------------TGGATCCACGA-TATCCTGCAGGAAGCTTTCTGGCGCCTGGCCGGCGCAATTGAATGGG---------CCCCTCTAGTCAAGGCC
lacZ-α-062      ------------------------------------------CGGCCGC----------GAATTC-------------------------------------------------ACTAGTAGAT--------------------------------------------------------
lacZ-α-063      -----------------------------------------------------------GAATTC---------------------------------------------------------------------------------------GAGCTCGGTACCCGGGGATCCTCTAGAG
lacZ-α-064      ---------------------------------------------------------TCGAATTC-------------------------------------CCGCGGCCGCCATGGCGGCCGGGAGCATGCGACGTCGGG---------------------------CCCAATTCGCCC
lacZ-α-065      --------------------GGATCC------------------CCGGGTACCGAGCTCGAATTC-------------------------------------------------------------------------------------------------------------------
lacZ-α-066      ------------------------------------------CGGCCGC-----------------------------------------------------------------ACTAGTAGAT--------------------------------------------------------
lacZ-α-067      -----------------------------------------------------------GAATTC---------------------------------------------------------------------------------------GAGCTCGGTACCCGGGGATCCTCTAGAG
lacZ-α-069      -------------------------------------------------------------------------------------------------------TCCCGCGGCCATGGCGGCCGGGAGCATGCGACGTCGGG---------------------------CCCAATTCGCCC
lacZ-α-070      --------------------GGATCC------------------CCGGGTACCGAGCTCGAATTC-------------------------------------------------------------------------------------------------------------------
lacZ-α-071      --------------------GGATCC--------ACTAGTAACGGCCGCCAGTGTGCTGGAATTC--------------------TGCAGATATC-----------------------------------CATCACACTGGCGGCCGCTCGAGCATGCATCTAGAGGGCCCAATTCGCCC
lacZ-α-072      --------------------GGATCC------------------CCGGGTACCGAGCTCGAATTC-------------------------------------------------------------------------------------------------------------------
lacZ-α-074      -----------------------------------------------------------GAATTCGAGCTCGGTACC---------------------------------------------------------------------------------------CGGGGATCCTCTAGAG
lacZ-α-075      -------------GGGCCCGGGATCCGATATCTAGATGCATTCGCGAGGTACCGAGCTCGAATTC-------------------------------------------------------------------------------------------------------------------
```

Sequence Alignments

```
lacZ-α-060        TCGACCTGCAGGCATGCAAGCTTGGCACTGGCCGTCGTTTTACAACGTCGTGACTGGGAAAACCCTGGCGTTACCCAACTTAATCGCCTTGCAGCACATCCCCCTTTCGCCAGCTGGCGTAATAGCGAAGAGGCCCGCACCGATCGCCCTTCCCAACAGTTGCGCAGCCTGAATGGCGAA
lacZ-α-031        TATAGTGAGTCGTATTACGCGCGCTCACTGGCCGTCGTTTTACAACGTCGTGACTGGGAAAACCCTGGCGTTACCCAACTTAATCGCCTTGCAGCACATCCCCCTTTCGCCAGCTGGCGTAATAGCGAAGAGGCCCGCACCGATCGCCCTTCCCAACAGTTGCGCAGCCTGAATGGCGAA
lacZ-α-034        TCGACCTGCAGGCATGCAAGCTTGGCACTGGCCGTCGTTTTACAACGTCGTGACTGGGAAAACCCTGGCGTTACCCAACTTAATCGCCTTGCAGCACATCCCCCTTTCGCCAGCTGGCGTAATAGCGAAGAGGCCCGCACCGATCGCCCTTCCCAACAGTTGCGCAGCCTGAATGGCGAA
lacZ-α-036        --------------------------ACTGGCCGTCGTTTTACAACGTCGTGACTGGGAAAACCCTGGCGTTACCCAACTTAATCGCCTTGCAGCACATCCCCCTTTCGCCAGCTGGCGTAATAGCGAAGAGGCCCGCACCGATCGCCCTTCCCAACAGTTGCGCAGCCTGAATGGCGAA
lacZ-α-057        --------------------------ACTGGCCGTCGTTTTACAACGTCGTGACTGGGAAAACCCTGGCGTTACCCAACTTAATCGCCTTGCAGCACATCCCCCTTTCGCCAGCTGGCGTAATAGCGAAGAGGCCCGCACCGATCGCCCTTCCCAACAGTTGCGCAGCCTGAATGGCGAA
lacZ-α-073        ------------------------------------------------------------------------------------------------------------------------------------------------------------------------------------
lacZ-α-076        TATAGTGAGTCGTATT---ACAATTCACTGGCCGTCGTTTTACAACGTCGTGACTGGGAAAACCCTGGCGTTACCCAACTTAATCGCCTTGCAGCACATCCCCCTTTCGCCAGCTGGCGTAATAGCGAAGAGGCCCGCACCGATCGCCCTTCCCAACAGTTGCGCAGCCTGAATGGCGAA
lacZ-α-032        TATAGTGAGTCGTATT---ACAATTCACTGGCCGTCGTTTTACAACGTCGTGACTGGGAAAACCCTGGCGTTACCCAACTTAATCGCCTTGCAGCACATCCCCCTTTCGCCAGCTGGCGTAATAGCGAAGAGGCCCGCACCGATCGCCCTTCCCAACAGTTGCGCAGCCTGAATGGCGAA
lacZ-α-068        TATAGTGAGTCGTATT---ACAATTCACTGGCCGTCGTTTTACAACGTCGTGACTGGGAAAACCCTGGCGTTACCCAACTTAATCGCCTTGCAGCACATCCCCCTTTCGCCAGCTGGCGTAATAGCGAAGAGGCCCGCACCGATCGCCCTTCCCAACAGTTGCGCAGCTGA---------
lacZ-α-001        --------------------------ACTGGCCGTCGTTTTACAACGTCGTGACTGGGAAAACCCTGGCGTTACCCAACTTAATCGCCTTGCAGCACATCCCCCTTTCGCCAGCTGGCGTAATAGCGAAGAGGCCCGCACCGATCGCCCTTCCCAACAGTTGCGCAGCCTGAATGGCGAA
lacZ-α-004        TCGACCTGCAGGCATGCAAGCTTGGCACTGGCCGTCGTTTTACAACGTCGTGACTGGGAAAACCCTGGCGTTACCCAACTTAATCGCCTTGCAGCACATCCCCCTTTCGCCAGCTGGCGTAATAGCGAAGAGGCCCGCACCGATCGCCCTTCCCAACAGTTGCGCAGCCTGAATGGCGAA
lacZ-α-005        TATAGTGAGTCGTATTACGCGCGCTCACTGGCCGTCGTTTTACAACGTCGTGACTGGGAAAACCCTGGCGTTACCCAACTTAATCGCCTTGCAGCACATCCCCCTTTCGCCAGCTGGCGTAATAGCGAAGAGGCCCGCACCGATCGCCCTTCCCAACAGTTGCGCAGCCTGAATGGCGAA
lacZ-α-006        TCGACCTGCAGC----CAAGCTTGGCACTGGCCGTCGTTTTACAACGTCGTGACTGGGAAAACCCTGGCGTTACCCAACTTAATCGCCTTGCAGCACATCCCCCTTTCGCCAGCTGGCGTAATAGCGAAGAGGCCCGCACCGATCGCCCTTCCCAACAGTTGCGCAGCCTG---------
lacZ-α-010        TATAGTGAGTCGTATTACGCGCGCTCACTGGCCGTCGTTTTACAACGTCGTGACTGGGAAAACCCTGGCGTTACCCAACTTAATCGCCTTGCAGCACATCCCCCTTTCGCCAGCTGGCGTAATAGCGAAGAGGCCCGCACCGATCGCCCTTCCCAACAGTTGCGCAGCCTGAATGGCGAA
lacZ-α-014        GTGTCACCTAAATGGCCGCACAATTCACTGGCCGTCGTTTTACAACGTCGTGACTGGGAAAACCCTGGCGTTACCCAACTTAATCGCCTTGCAGCACATCCCCCTTTCGCCAGCTGGCGTAATAGCGAAGAGGCCCGCACCGATCGCCCTTCCCAACAGTTGCGCAGCCTGAATGGCGAA
lacZ-α-015        TATAGTGAGTCGTATTACGCGCGCTCACTGGCCGTCGTTTTACAACGTCGTGACTGGGAAAACCCTGGCGTTACCCAACTTAATCGCCTTGCAGCACATCCCCCTTTCGCCAGCTGGCGTAATAGCGAAGAGGCCCGCACCGATCGCCCTTCCCAACAGTTGCGCAGCCTGAATGGCGAA
lacZ-α-020        --------------------------ACTGGCCGTCGTTTTACAACGTCGTGACTGGGAAAACCCTGGCGTTACCCAACTTAATCGCCTTGCAGCACATCCCCCTTTCGCCAGCTGGCGTAATAGCGAAGAGGCCCGCACCGATCGCCCTTCCCAACAGTTGCGCAGCCTGAATGGCGAA
lacZ-α-022        --------------------------ACTGGCCGTCGTTTTACAACGTCGTGACTGGGAAAACCCTGGCGTTACCCAACTTAATCGCCTTGCAGCACATCCCCCTTTCGCCAGCTGGCGTAATAGCGAAGAGGCCCGCACCGATCGCCCTTCCCAACAGTTGCGCAGCCTGAATGGCGAA
lacZ-α-028        TATAGTGAGTCGTATT---ACAATTCACTGGCCGTCGTTTTACAACGTCGTGACTGGGAAAACCCTGGCGTTACCCAACTTAATCGCCTTGCAGCACATCCCCCTTTCGCCAGCTGGCGTAATAGCGAAGAGGCCCGCACCGATCGCCCTTCCCAACAGTTGCGCAGCCTGAATGGCGAA
lacZ-α-039        TCGACCTGCAGGCATGCAAGCTTGGCACTGGCCGTCGTTTTACAACGTCGTGACTGGGAAAACCCTGGCGTTACCCAACTTAATCGCCTTGCAGCACATCCCCCTTTCGCCAGCTGGCGTAATAGCGAAGAGGCCCGCACCGATCGCCCTTCCCAACAGTTGCGTAGCCTGAATGGCGAA
lacZ-α-045        TCGACCTGCAGGCATGCAAGCTTGGCACTGGCCGTCGTTTTACAACGTCGTGACTGGGAAAACCCTGGCGTTACCCAACTTAATCGCCTTGCAGCACATCCCCCTTTCGCCAGCTGGCGTAATAGCGAAGAGGCCCGCACCGATCGCCCTTCCCAACAGTTGCGCAGCCTGAATGGCGAA
lacZ-α-046        -------------------ACAATTCACTGGCCGTCGTTTTACAACGTCGTGACTGGGAAAACCCTGGCGTTACCCAACTTAATCGCCTTGCAGCACATCCCCCTTTCGCCAGCTGGCGTAATAGCGAAGAGGCCCGCACCGATCGCCCTTCCCAACAGTTGCGCAGCCTGAATGGCGAA
lacZ-α-051        GTGTCACCTAAATGGCCGCACAATTCACTGGCCGTCGTTTTACAACGTCGTGACTGGGAAAACCCTGGCGTTACCCAACTTAATCGCCTTGCAGCACATCCCCCTTTCGCCAGCTGGCGTAATAGCGAAGAGGCCCGCACCGATCGCCCTTCCCAACAGTTGCGCAGCCTGAATGGCGAA
lacZ-α-052        --------------------------ACTGGCCGTCGTTTTACAACGTCGTGACTGGGAAAACCCTGGCGTTACCCAACTTAATCGCCTTGCAGCACATCCCCCTTTCGCCAGCTGGCGTAATAGCGAAGAGGCCCGCACCGATCGCCCTTCCCAACAGTTGCGCAGCCTGAATGGCGAA
lacZ-α-036corr    --------------------------ACTGGCCGTCGTTTTACAACGTCGTGACTGGGAAAACCCTGGCGTTACCCAACTTAATCGCCTTGCAGCACATCCCCCTTTCGCCAGCTGGCGTAATAGCGAAGAGGCCCGCACCGATCGCCCTTCCCAACAGTTGCGCAGCCTGAATGGCGAA
lacZ-α-002        ATTCTGCTAGCCAATCAAGATCTCTCGCTGGCCGTCGTTTTACAACGTCGTGACTGGGAGAATCCTGGCGTTACCCAACTTAATCGCCTTGCAGCGCACCCACCGTTCGCAAGCTGGCGTAATAGCGAAGAGGCCCGCACCGATCGCCCTTCCCAACAGTTGCGCAGCCTGAATGGCGAA
lacZ-α-003        ATTCTGCTAGCCAATCAAGATCTCTCGCTGGCCGTCGTTTTACAACGTCGTGACTGGGAGAATCCTGGCGTTACCCAACTTAATCGCCTTGCAGCGCACCCACCGTTCGCAAGCTGGCGTAATAGCGAAGAGGCCCGCACCGATCGCCCTTCCCAACAGTTGCGCAGCCTGAATGGCGAA
lacZ-α-007        AGGGATAACAGGGTAATTAGCTTGGCACTGGCCGTCGTTTTACAACGTCGTGACTGGGAAAACCCTGGCGTTACCCAACTTAATCGCCTTGCAGCACATCCCCCTTTCGCCAGCTGGCGTAATAGCGAAGAGGCCCGCACCGATCGCCCTTCCCAACAGTTGCGCAGCCTGAATGGCGAA
lacZ-α-008        --------------------------ACTGGCCGTCGTTTTACAACGTCGTGACTGGGAAAACCCTGGCGTTACCCAACTTAATCGCCTTGCAGCACATCCCCCTTTCGCCAGCTGGCGTAATAGCGAAGAGGCCCGCACCGATCGCCCTTCCCAACAGTTGCGCAGCCTGAATGGCGAA
lacZ-α-009        --------------------------ACTGGCCGTCGTTTTACAACGTCGTGACTGGGAAAACCCTGGCGTTACCCAACTTAATCGCCTTGCAGCACATCCCCCTTTCGCCAGCTGGCGTAATAGCGAAGAGGCCCGCACCGATCGCCCTTCCCAACAGTTGCGCAGCCTGAATGGCGAA
lacZ-α-011        --------------------------ACTGGCCGTCGTTTTACAACGTCGTGACTGGGAAAACCCTGGCGTTACCCAACTTAATCGCCTTGCAGCACATCCCCCTTTCGCCAGCTGGCGTAATAGCGAAGAGGCCCGCACCGATCGCCCTTCCCAACAGTTGCGCAGCCTGAATGGCGAA
lacZ-α-012        TATAGTGAGTCGTATT---ACAATTCACTGGCCGTCGTTTTACAACGTCGTGACTGGGAAAACCCTGGCGTTACCCAACTTAATCGCCTTGCAGCACATCCCCCTTTCGCCAGCTGGCGTAATAGCGAAGAGGCCCGCACCGATCGCCCTTCCCAACAGTTGCGCAGCCTGAATGGCGAA
lacZ-α-013        TCGACCTGCAGGCATGCAAGCTTGGCACTGGCCGTCGTTTTACAACGTCGTGACTGGGAAAACCCTGGCGTTACCCAACTTAATCGCCTTGCAGCACATCCCCCTTTCGCCAGCTGGCGTAATAGCGAAGAGGCCCGCACCGATCGCCCTTCCCAACAGTTGCGCAGCCTGAATGGCGAA
lacZ-α-016        TATAGTGAGTCGTATT---ACAATTCACTGGCCGTCGTTTTACAACGTCGTGACTGGGAAAACCCTGGCGTTACCCAACTTAATCGCCTTGCAGCACATCCCCCTTTCGCCAGCTGGCGTAATAGCGAAGAGGCCCGCACCGATCGCCCTTCCCAACAGTTGCGCAGCCTA---------
lacZ-α-017        TATAGTGAGTCGTATT---ACAATTCACTGGCCGTCGTTTTACAACGTCGTGACTGGGAAAACCCTGGCGTTACCCAACTTAATCGCCTTGCAGCACATCCCCCTTTCGCCAGCTGGCGTAATAGCGAAGAGGCCCGCACCGATCGCCCTTCCCAACAGTTGCGCAGCCTGAATGGCGAA
lacZ-α-018        TATAGTGAGTCGTATT---ACAATTCACTGGCCGTCGTTTTACAACGTCGTGACTGGGAAAACCCTGGCGTTACCCAACTTAATCGCCTTGCAGCACATCCCCCTTTCGCCAGCTGGCGTAATAGCGAAGAGGCCCGCACCGATCGCCCTTCCCAACAGTTGCGCAGCCTGAATGGCGAA
lacZ-α-019        TATAGTGAGTCGTATT---ACAATTCACTGGCCGTCGTTTTACAACGTCGTGACTGGGAAAACCCTGGCGTTACCCAACTTAATCGCCTTGCAGCACATCCCCCTTTCGCCAGCTGGCGTAATAGCGAAGAGGCCCGCACCGATCGCCCTTCCCAACAGTTGCGCAGCCTGAATGGCGAA
lacZ-α-021        TATAGTGAGTCGTATT---ACAATTCACTGGCCGTCGTTTTACAACGTCGTGACTGGGAAAACCCTGGCGTTACCCAACTTAATCGCCTTGCAGCACATCCCCCTTTCGCCAGCTGGCGTAATAGCGAAGAGGCCCGCACCGATCGCCCTTCCCAACAGTTGCGCAGCCTA---------
lacZ-α-023        TATAGTGAGTCGTATT---ACAATTCACTGGCCGTCGTTTTACAACGTCGTGACTGGGAAAACCCTGGCGTTACCCAACTTAATCGCCTTGCAGCACATCCCCCTTTCGCCAGCTGGCGTAATAGCGAAGAGGCCCGCACCGATCGCCCTTCCCAACAGTTGCGCAGCCTGAATGGCGAA
lacZ-α-041corr    TATAGTGAGTCGTATT---ACAATTCACTGGCCGTCGTTTTACAACGTCGTGACTGGGAAAACCCTGGCGTTACCCAACTTAATCGCCTTGCAGCACATCCCCCTTTCGCCAGCTGGCGTAATAGCGAAGAGGCCCGCACCGATCGCCCTTCCCAACAGTTGCGCAGCCTGAATGGCGAA
lacZ-α-024        TATAGTGTCACCTAAATT--CAATTCACTGGCCGTCGTTTTACAACGTCGTGACTGGGAAAACCCTGGCGTTACCCAACTTAATCGCCTTGCAGCACATCCCCCTTTCGCCAGCTGGCGTAATAGCGAAGAGGCCCGCACCGATCGCCCTTCCCAACAGTTGCGCAGCCTGAATGGCGAA
lacZ-α-025        TATAGTGAGTCGTATT---ACAATTCACTGGCCGTCGTTTTACAACGTCGTGACTGGGAAAACCCTGGCGTTACCCAACTTAATCGCCTTGCAGCACATCCCCCTTTCGCCAGCTGGCGTAATAGCGAAGAGGCCCGCACCGATCGCCCTTCCCAACAGTTGCGCAGCCTGAATGGCGAA
lacZ-α-026        GTGTCACCTAAATGGCCGCACAATTCACTGGCCGTCGTTTTACAACGTCGTGACTGGGAAAACCCTGGCGTTACCCAACTTAATCGCCTTGCAGCACATCCCCCTTTCGCCAGCTGGCGTAATAGCGAAGAGGCCCGCACCGATCGCCCTTCCCAACAGTTGCGCAGCCTGAATGGCGAA
lacZ-α-027        TATAGTGAGTCGTATT---ACAATTCACTGGCCGTCGTTTTACAACGTCGTGACTGGGAAAACCCTGGCGTTACCCAACTTAATCGCCTTGCAGCACATCCCCCTTTCGCCAGCTGGCGTAATAGCGAAGAGGCCCGCACCGATCGCCCTTCCCAACAGTTGCGCAGCCTGAATGGCGAA
lacZ-α-029        TATAGTGAGTCGTATT---ACAATTCACTGGCCGTCGTTTTACAACGTCGTGACTGGGAAAACCCTGGCGTTACCCAACTTAATCGCCTTGCAGCACATCCCCCTTTCGCCAGCTGGCGTAATAGCGAAGAGGCCCGCACCGATCGCCCTTCCCAACAGTTGCGCAGCCTGAATGGCGAA
lacZ-α-030        --------------------------ACTGGCCGTCGTTTTACAACGTCGTGACTGGGAAAACCCTGGCGTTACCCAACTTAATCGCCTTGCAGCACATCCCCCTTTCGCCAGCTGGCGTAATAGCGAAGAGGCCCGCACCGATCGCCCTTCCCAACAGTTGCGCAGCCTGAATGGCG--
lacZ-α-033        --------------------------ACTGGCCGTCGTTTTACAACGTCGTGACTGGGAAAACCCTGGCGTTACCCAACTTAATCGCCTTGCAGCACATCCCCCTTTCGCCAGCTGGCGTAATAGCGAAGAGGCCCGCACCGATCGCCCTTCCCAACAGTTGCGCAGCCTGAATGGCGAA
lacZ-α-035        TATAGTGTCACCTAAATC--CAATTCACTGGCCGTCGTTTTACAACGTCGTGACTGGGAAAACCCTGGCGTTACCCAACTTAATCGCCTTGCAGCACATCCCCCTTTCGCCAGCTGGCGTAATAGCGAAGAGGCCCGCACCGATCGCCCTTCCCAACAGTTGCGCAGCCTGAATGGCGAA
lacZ-α-037        TATAGTGTCACCTAAATT--CAATTCACTGGCCGTCGTTTTACAACGTCGTGACTGGGAAAACCCTGGCGTTACCCAACTTAATCGCCTTGCAGCACATCCCCCTTTCGCCAGCTGGCGTAATAGCGAAGAGGCCCGCACCGATCGCCCTTCCCAACAGTTGCGCAGCCTGAATGGCGAA
lacZ-α-038        TTAAGTGAGTCGTATTACGG------ACTGGCCGTCGTTTTACAACGTCGTGACTGGGAAAACCCTGGCGTTACCCAACTTAATCGCCTTGCAGCACATCCCCCTTTCGCCAGCTGGCGTAATAGCGAAGAGGCCCGCACCGATCGCCCTTCCCAACAGTTGCGCAGCCTGAATGGCGAA
lacZ-α-040        TATAGTGAGTCGTATT---ACAATTCACTGGCCGTCGTTTTACAACGTCGTGACTGGGAAAACCCTGGCGTTACCCAACTTAATCGCCTTGCAGCACATCCCCCTTTCGCCAGCTGGCGTAATAGCGAAGAGGCCCGCACCGATCGCCCTTCCCAACAGTTGCGCAGCCTGAATGGCGAA
lacZ-α-041        TATAGTGAGTCGTATT---ACAATTCACTGGCCGTCGTTTTACAACGTCGTGACTGGGAAAACCCTGGCGTTACCCAACTTAATCGCCTTGCAGCACATCCCCCTTTCGCCAGCTGGCGTAATAGCGAAGAGGCCCGCACCGATCGCCCTTCCCAACAGTTGCGCAGCCTGA--------
lacZ-α-042        TATAGTGAGTCGTATT---ACAATTCACTGGCCGTCGTTTTACAACGTCGTGACTGGGAAAACCCTGGCGTTACCCAACTTAATCGCCTTGCAGCACATCCCCCTTTCGCCAGCTGGCGTAATAGCGAAGAGGCCCGCACCGATCGCCCTTCCCAACAGTTGCGCAGCCTGAATGGCGAA
lacZ-α-043        TTAAGTGAGTCGTATTACGG------ACTGGCCGTCGTTTTACAACGTCGTGACTGGGAAAACCCTGGCGTTACCCAACTTAATCGCCTTGCAGCACATCCCCCTTTCGCCAGCTGGCGTAATAGCGAAGAGGCCCGCACCGATCGCCCTTCCCAACAGTTGCGCAGCCTGAATGGCGAA
lacZ-α-044        ------------TTAATTAACAATTCACTGGCCGTCGTTTTACAACGTCGTGACTGGGAAAACCCTGGCGTTACCCAACTTAATCGCCTTGCAGCACATCCCCCTTTCGCCAGCTGGCGTAATAGCGAAGAGGCCCGCACCGATCGCCCTTCCCAACAGTTGCGCAGCCTGAATGGCG--
lacZ-α-047        TATAGTGAGTCGTATT---ACAATTCACTGGCCGTCGTTTTACAACGTCGTGACTGGGAAAACCCTGGCGTTACCCAACTTAATCGCCTTGCAGCACATCCCCCTTTCGCCAGCTGGCGTAATAGCGAAGAGGCCCGCACCGATCGCCCTTCCCAACAGTTGCGCAGCCTGAATGGCGAA
lacZ-α-048        TATAGTGAGTCGTATT---ACAATTCACTGGCCGTCGTTTTACAACGTCGTGACTGGGAAAACCCTGGCGTTACCCAACTTAATCGCCTTGCAGCACATCCCCCTTTCGCCAGCTGGCGTAATAGCGAAGAGGCCCGCACCGATCGCCCTTCCCAACAGTTGCGCAGCCTGAATGGCGAA
lacZ-α-049        TATAGTGAGTCGTATT---ACAATTCACTGGCCGTCGTTTTACAACGTCGTGACTGGGAAAACCCTGGCGTTACCCAACTTAATCGCCTTGCAGCACATCCCCCTTTCGCCAGCTGGCGTAATAGCGAAGAGGCCCGCACCGATCGCCCTTCCCAACAGTTGCGCAGCCTGAATGGCGAA
lacZ-α-050        TATAGTGAGTCGTATTACGG------ACTGGCCGTCGTTTTACAACGTCGTGACTGGGAAAACCCTGGCGTTACCCAACTTAATCGCCTTGCAGCACATCCCCCTTTCGCCAGCTGGCGTAATAGCGAAGAGGCCCGCACCGATCGCCCTTCCCAACAGTTGCGCAGCCTGAATGGCGAA
lacZ-α-053        TATAGTGAGTCGTATT---ACAATTCACTGGCCGTCGTTTTACAACGTCGTGACTGGGAAAACCCTGGCGTTACCCAACTTAATCGCCTTGCAGCACATCCCCCTTTCGCCAGCTGGCGTAATAGCGAAGAGGCCCGCACCGATCGCCCTTCCCAACAGTTGCGCAGCCTGAATGGCGAA
lacZ-α-054        TCGACCTGCAGGCATGCAAGCTTGGCACTGGCCGTCGTTTTACAACGTCGTGACTGGGAAAACCCTGGCGTTACCCAACTTAATCGCCTTGCAGCACATCCCCCTTTCGCCAGCTGGCGTAATAGCGAAGAGGCCCGCACCGATCGCCCTTCCCAACAGTTGCGCAGCCTGAATGGCGAA
lacZ-α-055        TATAGTGAGTCGTATTACGCGCGCTCACTGGCCGTCGTTTTACAACGTCGTGACTGGGAAAACCCTGGCGTTACCCAACTTAATCGCCTTGCAGCACATCCCCCTTTCGCCAGCTGGCGTAATAGCGAAGAGGCCCGCACCGATCGCCCTTCCCAACAGTTGCGCAGCCTGAATGGCGAA
lacZ-α-056        TTAAGTGAGTCGTATTACGG------ACTGGCCGTCGTTTTACAACGTCGTGACTGGGAAAACCCTGGCGTTACCCAACTTAATCGCCTTGCAGCACATCCCCCTTTCGCCAGCTGGCGTAATAGCGAAGAGGCCCGCACCGATCGCCCTTCCCAACAGTTGCGCAGCCTGAATGGCGAA
lacZ-α-058        TATAGTGAGTCGTATTACGG------ACTGGCCGTCGTTTTACAACGTCGTGACTGGGAAAACCCTGGCGTTACCCAACTTAATCGCCTTGCAGCACATCCCCCTTTCGCCAGCTGGCGTAATAGCGAAGAGGCCCGCACCGATCGCCCTTCCCAACAGTTGCGCAGCCTGAATGGCGAA
lacZ-α-059        --------------------------ACTGGCCGTCGTTTTACAACGTCGTGACTGGGAAAACCCTGGCGTTACCCAACTTAATCGCCTTGCAGCACATCCCCCTTTCGCCAGCTGGCGTAATAGCGAAGAGGCCCGCACCGATCGCCCTTCCCAACAGTTGCGCAGCCTGAATGGCGAA
lacZ-α-061        TTAAGTGAGTCGTATTACGG------ACTGGCCGTCGTTTTACAACGTCGTGACTGGGAAAACCCTGGCGTTACCCAACTTAATCGCCTTGCAGCACATCCCCCTTTCGCCAGCTGGCGTAATAGCGAAGAGGCCCGCACCGATCGCCCTTCCCAACAGTTGCGCAGCCTGAATGGCGAA
lacZ-α-062        ------------------------------------------------------------------------------------------------------------------------------------------------------------------------------------
lacZ-α-063        TCGACCTGCAGGCATGCAAGCTTGGCACTGGCCGTCGTTTTACAACGTCGTGACTGGGAAAACCCTGGCGTTACCCAACTTAATCGCCTTGCAGCACATCCCCCTTTCGCCAGCTGGCGTAATAGCGAAGAGGCCCGCACCGATCGCCCTTCCCAACAGTTGCGCAGCCTGAATGGCGAA
lacZ-α-064        TATAGTGAGTCGTATT---ACAATTCACTGGCCGTCGTTTTACAACGTCGTGACTGGGAAAACCCTGGCGTTACCCAACTTAATCGCCTTGCAGCACATCCCCCTTTCGCCAGCTGGCGTAATAGCGAAGAGGCCCGCACCGATCGCCCTTCCCAACAGTTGCGCAGCCTGAATGGCGAA
lacZ-α-065        --------------------------ACTGGCCGTCGTTTTACAACGTCGTGACTGGGAAAACCCTGGCGTTACCCAACTTAATCGCCTTGCAGCACATCCCCCTTTCGCCAGCTGGCGTAATAGCGAAGAGGCCCGCACCGATCGCCCTTCCCAACAGTTGCGCAGCCTGAATGGCGAA
lacZ-α-066        ------------------------------------------------------------------------------------------------------------------------------------------------------------------------------------
lacZ-α-067        TCGACCTGCAGGCATGCAAGCTTGGCACTGGCCGTCGTTTTACAACGTCGTGACTGGGAAAACCCTGGCGTTACCCAACTTAATCGCCTTGCAGCACATCCCCCTTTCGCCAGCTGGCGTAATAGCGAAGAGGCCCGCACCGATCGCCCTTCCCAACAGTTGCGCAGCCTGAATGGCGAA
lacZ-α-069        TATAGTGAGTCGTATT---ACAATTCACTGGCCGTCGTTTTACAACGTCGTGACTGGGAAAACCCTGGCGTTACCCAACTTAATCGCCTTGCAGCACATCCCCCTTTCGCCAGCTGGCGTAATAGCGAAGAGGCCCGCACCGATCGCCCTTCCCAACAGTTGCGCAGCCTGAATGGCGAA
lacZ-α-070        --------------------------ACTGGCCGTCGTTTTACAACGTCGTGACTGGGAAAACCCTGGCGTTACCCAACTTAATCGCCTTGCAGCACATCCCCCTTTCGCCAGCTGGCGTAATAGCGAAGAGGCCCGCACCGATCGCCCTTCCCAACAGTTGCGCAGCCTGAATGGCGAA
lacZ-α-071        TATAGTGAGTCGTATT---ACAATTCACTGGCCGTCGTTTTACAACGTCGTGACTGGGAAAACCCTGGCGTTACCCAACTTAATCGCCTTGCAGCACATCCCCCTTTCGCCAGCTGGCGTAATAGCGAAGAGGCCCGCACCGATCGCCCTTCCCAACAGTTGCGCAGCCTGAATGGCGAA
lacZ-α-072        --------------------------ACTGGCCGTCGTTTTACAACGTCGTGACTGGGAAAACCCTGGCGTTACCCAACTTAATCGCCTTGCAGCACATCCCCCTTTCGCCAGCTGGCGTAATAGCGAAGAGGCCCGCACCGATCGCCCTTCCCAACAGTTGCGCAGCCTGAATGGCGAA
lacZ-α-074        TCGACCTGCAGGCATGCAAGCTT---ACTGGCCGTCGTTTTACAACGTCGTGACTGGGAAAACCCTGGCGTTACCCAACTTAATCGCCTTGCAGCACATCCCCCTTTCGCCAGCTGGCGTAATAGCGAAGAGGCCCGCACCGATCGCCCTTCCCAACAGTTGCGCAGCCTGAATGGCGAA
lacZ-α-075        --------------------------ACTGGCCGTCGTTTTACAACGTCGTGACTGGGAAAACCCTGGCGTTACCCAACTTAATCGCCTTGCAGCACATCCCCCTTTCGCCAGCTGGCGTAATAGCGAAGAGGCCCGCACCGATCGCCCTTCCCAACAGTTGCGCAGCCTGAATGGCGAA
                                            ************************************************************************ ** ** ***** ***************************************************** ****
```

Sequence Alignments

```
lacZ-α-060       TGCTAG------------------------------------------------------------------------------------------------------------------------------------------------------------------------------
lacZ-α-031       TGGCGCGACGCGCCCTGTAGCGGCGCATTAAGCGCGGCGGGTGTGGTGGTTACGCGCAGCGTGACCGCTACACTTGCCAGCGCCCTAGCGCCCGCTCCTTTCGCTTTCTTCCCTTCCTTTCTCGCCACGTTCGCCGGCTTTCCCCGTCAAGCTCTAAATCGGGGGCTCCCTTTAGGGTTC...
lacZ-α-034       TGA---------------------------------------------------------------------------------------------------------------------------------------------------------------------------------
lacZ-α-036       TGGCGCCTGATGCGGTATTTTCTCCTTACGCATCTGTGCGGTATTTCACACCGCATA---------------------------------------------------------------------------------------------------------------------------
lacZ-α-057       TGGCGCCTGATGCGGTATTTTCTCCTTACGCATCTGTGCGGTATTTCACACCGCATATGGTGCACTCTCAGTACAATCTGCTCTGATGCCGCATAG------------------------------------------------------------------------------------
lacZ-α-073       ------------------------------------------------------------------------------------------------------------------------------------------------------------------------------------
lacZ-α-076       TGGA----CGCGCCCTGTAGCGGCGCATTAA-----------------------------------------------------------------------------------------------------------------------------------------------------
lacZ-α-032       TGGCGCGAAATTGTAAACGTTAATGTTATCGATACATGA---------------------------------------------------------------------------------------------------------------------------------------------
lacZ-α-068       ------------------------------------------------------------------------------------------------------------------------------------------------------------------------------------
lacZ-α-001       TGCGATTTATTCAACAAAGCCGCCGTCCCGTCAAGTCAGCGTAATGCTCTGCCAGTGTTACAACCAATTAACCAATTCTGA---------------------------------------------------------------------------------------------------
lacZ-α-004       TGGCGCCTGATGCGGTATTTTCTCCTTACGCATCTGTGCGGTATTTCACACCGCATACGTCAAAGCAACCATAGTACGCGCCCTGTAGCGGCGCATTAA-------------------------------------------------------------------------------
lacZ-α-005       TGG---GACGCGCCCTGTAGCGGCGCATTAAGCGCGGCGGGTGTGGTGGTTACGCGCAGCGTGACCGCTACACTTGCCAGCGCCCTAGCGCCCGCTCCTTTCGCTTTCTTCCCTTCCTTTCTCGCCACGTTCGCCGGCTTTCCCCGTCAAGCTCTAAATCGGGGGCTCCCTTTAGGGTTC...
lacZ-α-006       ------------------------------------------------------------------------------------------------------------------------------------------------------------------------------------
lacZ-α-010       TGGG---ACGCGCCCTGTAGCGGCGCATTAAGCGCGGCGGGTGTGGTGGTTACGCGCAGCGTGACCGCTACACTTGCCAGCGCCCTAGCGCCCGCTCCTTTCGCTTTCTTCCCTTCCTTTCTCGCCACGTTCGCCGGCTTTCCCCGTCAAGCTCTAAATCGGGGGCTCCCTTTAGGGTTC...
lacZ-α-014       TGG----AAATTGTAA--------------------------------------------------------------------------------------------------------------------------------------------------------------------
lacZ-α-015       TGG---GACGCGCCCTGTAGCGGCGCATTAAGCGCGGCGGGTGTGGTGGTTACGCGCAGCGTGACCGCTACACTTGCCAGCGCCCTAGCGCCCGCTCCTTTCGCTTTCTTCCCTTCCTTTCTCGCCACGTTCGCCGGCTTTCCCCGTCAAGCTCTAAATCGGGGGCTCCCTTTAGGGTTC...
lacZ-α-020       TGGCGCTAA---------------------------------------------------------------------------------------------------------------------------------------------------------------------------
lacZ-α-022       TGGCGCCTGATGCGGTATTTTCTCCTTACGCATCTGTGCGGTATTTCACACCGCATATGGTGCACTCTCAGTACAATCTGCTCTGATGCCGCATAG------------------------------------------------------------------------------------
lacZ-α-028       TGGCGCCTGATGCGGTATTTTCTCCTTACGCATCTGTGCGGTATTTCACACCGCATATGGTGCACTCTCAGTACAATCTGCTCTGATGCCGCATAG------------------------------------------------------------------------------------
lacZ-α-039       TGGCGCCTGATGCGGTATTTTCTCCTTACGCATCTGTGCGGTATTTCACACCGCATAGATCCGGTACGTCGTTAA---------------------------------------------------------------------------------------------------------
lacZ-α-045       TGGCGCCTGATGCGGTATTTTCTCCTTACGCATCTGTGCGGTATTTCACACCGCATAGGGTAA---------------------------------------------------------------------------------------------------------------------
lacZ-α-046       TGG----AAATTGTAA--------------------------------------------------------------------------------------------------------------------------------------------------------------------
lacZ-α-051       TGG----AAATTGTAA--------------------------------------------------------------------------------------------------------------------------------------------------------------------
lacZ-α-052       TGGCGCCTGATGCGGTATTTTCTCCTTACGCATCTGTGCGGTATTTCACACCGCATATATCGCTGGGCCATTCTCATGAAGAATATCTTGAATTTATTGTCATATTACTAG---------------------------------------------------------------------
lacZ-α-036corr   TGGCGCCTGATGCGGTATTTTCTCCTTACGCATCTGTGCGGTATTTCACACCGCATATATCGCTGGGCCATTCTCATGAAGAATATCTTGAATTTATTGTCATATTACTAG---------------------------------------------------------------------
lacZ-α-002       TGG----AAATTGTAA--------------------------------------------------------------------------------------------------------------------------------------------------------------------
lacZ-α-003       TGG----AAATTGTAA--------------------------------------------------------------------------------------------------------------------------------------------------------------------
lacZ-α-007       TGGGA--AATTGTAAACGTTAATATTTTGTTAATATTTTGTTAA----------------------------------------------------------------------------------------------------------------------------------------
lacZ-α-008       TGGCGCCTGATGCGGTATTTTCTCCTTACGCATCTGTGCGGTATTTCACACCGCATATGGTGCACTCTCAGTACAATCTGCTCTGATGCCGCATAG------------------------------------------------------------------------------------
lacZ-α-009       TGA---------------------------------------------------------------------------------------------------------------------------------------------------------------------------------
lacZ-α-011       TGGCGCCTGATGCGGTATTTTCTCCTTACGCATCTGTGCGGTATTTCACACCGCATACGTCAAAGCAACCATAGTACGCGCCCTGTAGCGGCGCATTAA-------------------------------------------------------------------------------
lacZ-α-012       TGG----AAATTGTAA--------------------------------------------------------------------------------------------------------------------------------------------------------------------
lacZ-α-013       TGGCGCCTGATGCGGTATTTTCTCCTTACGCATCTGTGCGGTATTTCACACCGCATATGGTGCACTCTCAGTACAATCTGCTCTGATGCCGCATAG------------------------------------------------------------------------------------
lacZ-α-016       ------------------------------------------------------------------------------------------------------------------------------------------------------------------------------------
lacZ-α-017       TGG----ACGCGCCCTGTAGCGGCGCATTAA-----------------------------------------------------------------------------------------------------------------------------------------------------
lacZ-α-018       TGG---AGATCCAATTTTTAA---------------------------------------------------------------------------------------------------------------------------------------------------------------
lacZ-α-019       TGGG---ACGCGCCCTGTAGCGGCGCATTAAGCGCGGCGGGTGTGGTGGTTACGCGCAGCGTGACCGCTACACTTGCCAGCGCCCTAGCGCCCGCTCCTTTCGCTTTCTTCCCTTCCTTTCTCGCCACGTTCGCCGGCTTTCCCCGTCAAGCTCTAAATCGGGGGCTCCCTTTAGGGTTC...
lacZ-α-021       ------------------------------------------------------------------------------------------------------------------------------------------------------------------------------------
lacZ-α-023       TGGCGCGAAATTGTAAACGTTAATGTTATCGTACCCCTACTCCAAAAATGTCAAAGATACAGTCTCAGAAGACCAAAGGGCTATTGA---------------------------------------------------------------------------------------------
lacZ-α-041corr   TGGCGCGAAATTGTAAACGTTAATGTTATCGTACCCCTACTCCAAAAATGTCAAAGATACAGTCTCAGAAGACCAAAGGGCTATTGA---------------------------------------------------------------------------------------------
lacZ-α-024       TGG----ACGCGCCCTGTAGCGGCGCATTAA-----------------------------------------------------------------------------------------------------------------------------------------------------
lacZ-α-025       TGG---GACGCGCCCTGTAGCGGCGCATTAAGCGCGGCGGGTGTGGTGGTTACGCGCAGCGTGACCGCTACACTTGCCAGCGCCCTAGCGCCCGCTCCTTTCGCTTTCTTCCCTTCCTTTCTCGCCACGTTCGCCGGCTTTCCCCGTCAAGCTCTAAATCGGGGGCTCCCTTTAGGGTTC...
lacZ-α-026       TGG----AAATTGTAA--------------------------------------------------------------------------------------------------------------------------------------------------------------------
lacZ-α-027       TGG----ACGCGCCCTGTAGCGGCGCATTAA-----------------------------------------------------------------------------------------------------------------------------------------------------
lacZ-α-029       TGGG---ACGCGCCCTGTAGCGGCGCATTAAGCGCGGCGGGTGTGGTGGTTACGCGCAGCGTGACCGCTACACTTGCCAGCGCCCTAGCGCCCGCTCCTTTCGCTTTCTTCCCTTCCTTTCTCGCCACGTTCGCCGGCTTTCCCCGTCAAGCTCTAAATCGGGGGCTCCCTTTAGGGTTC...
lacZ-α-030       ------------------------------------------------------------------------------------------CCCGCTCCTTTCGCTTTCTTCCCTTCCTTTCTCGCCACGTTCGCCGGCTTTCCCCGTCAAGCTCTAAATCGGGGGCTCCCTTTAGGGTTC...
lacZ-α-033       TGGG--AAATTGTAAACGTTAATATTTTGTTAAAATTCGCGTTAAATTTTTGTTAA----------------------------------------------------------------------------------------------------------------------------
lacZ-α-035       TGGCGCCTGATGCGGTATTTTCTCCTTACGCATCTGTGCGGTATTTCACACCGCATATGGTGCACTCTCAGTACAATCTGCTCTGATGCCGCATAG------------------------------------------------------------------------------------
lacZ-α-037       TGG----ACGCGCCCTGTAGCGGCGCATTAA-----------------------------------------------------------------------------------------------------------------------------------------------------
lacZ-α-038       TGGCGCTTCGCTTGGTAA------------------------------------------------------------------------------------------------------------------------------------------------------------------
lacZ-α-040       TGG----ACGCGCCCTGTAGCGGCGCATTAA-----------------------------------------------------------------------------------------------------------------------------------------------------
lacZ-α-041       ------------------------------------------------------------------------------------------------------------------------------------------------------------------------------------
lacZ-α-042       TGGCGCGACGCGCCCTGTAGCGGCGCATTAAGCGCGGCGGGTGTGGTGGTTACGCGCAGCGTGACCGCTACACTTGCCAGCGCCCTAGCGCCCGCTCCTTTCGCTTTCTTCCCTTCCTTTCTCGCCACGTTCGCCGGCTTTCCCCGTCAAGCTCTAAATCGGGGGCTCCCTTTAGGGTTC...
lacZ-α-043       TGGCGCTTCGCTTGGTAA------------------------------------------------------------------------------------------------------------------------------------------------------------------
lacZ-α-044       ------------------------------------------------------------------------------------------CCCGCTCCTTTCGCTTTCTTCCCTTCCTTTCTCGCCACGTTCGCCGGCTTTCCCCGTCAAGCTCTAAATCGGGGGCTCCCTTTAGGGTTC...
lacZ-α-047       TGGCGCGAAATTGTAAACGTTAATGTTAACGTTACACCACAATATATCCTGCCAAGATCTCTAATTCCGGGGATCGGAAATCCAGAAGCCCGAGAGGTTGCCGCCTTTCGGGCTTTTTCTTTTTCAAAAAAAAAAATTTATAAAACGATCTGTTGCGGCCGGCCGCCGGGTTGTGGGCAA...
lacZ-α-048       TGG----ACGCGCCCTGTAGCGGCGCATTAA-----------------------------------------------------------------------------------------------------------------------------------------------------
lacZ-α-049       TGG----ACGCGCCCTGTAGCGGCGCATTAA-----------------------------------------------------------------------------------------------------------------------------------------------------
lacZ-α-050       TGGCGCTTCGCTTGGTAA------------------------------------------------------------------------------------------------------------------------------------------------------------------
lacZ-α-053       TGGCGCTTCGCTTGGTAA------------------------------------------------------------------------------------------------------------------------------------------------------------------
lacZ-α-054       TGCGATTTATTCAACAAAGCCGCCGTCCCGTCAAGTCAGCGTAATGCTCTGCCAGTGTTACAACCAATTAACCAATTCTGA---------------------------------------------------------------------------------------------------
lacZ-α-055       TGGA---AATTGTAA---------------------------------------------------------------------------------------------------------------------------------------------------------------------
lacZ-α-056       TGGCGCTTCGCTTGGTAA------------------------------------------------------------------------------------------------------------------------------------------------------------------
lacZ-α-058       TGGCGCTTCGCTTGGTAA------------------------------------------------------------------------------------------------------------------------------------------------------------------
lacZ-α-059       TGA---------------------------------------------------------------------------------------------------------------------------------------------------------------------------------
lacZ-α-061       TGGCGCTTCGCTTGGTAA------------------------------------------------------------------------------------------------------------------------------------------------------------------
lacZ-α-062       ------------------------------------------------------------------------------------------------------------------------------------------------------------------------------------
lacZ-α-063       TGGCGCTTTGCCTGGTTTCCGGCACCAGAAGCGGTGCCGGAAAGCTGGCTGGAGTGCGATCTTCCTGAGGCCGATACTGTCGTCGTCCCCTCAAACTGGCAGATGCACGGTTACGATGCGCCCATCTACACCAACGTGACCTATCCCATTACGGTCAATCCGCCGTTTGTTCCCACGGAG...
lacZ-α-064       TGG----ACGCGCCCTGTAGCGGCGCATTAA-----------------------------------------------------------------------------------------------------------------------------------------------------
lacZ-α-065       TGGCGCTTTGCCTGGTTTCCGGCACCAGAAGCGGTGCCGGAAAGCTGGCTGGAGTGCGATCTTCCTGAGGCCGATACTGTCGTCGTCCCCTCAAACTGGCAGATGCACGGTTACGATGCGCCCATCTACACCAACGTGACCTATCCCATTACGGTCAATCCGCCGTTTGTTCCCACGGAG...
lacZ-α-066       ------------------------------------------------------------------------------------------------------------------------------------------------------------------------------------
lacZ-α-067       TGGCGTCTAAGAAACCATTATTATCATGACATTAACCTATAA------------------------------------------------------------------------------------------------------------------------------------------
lacZ-α-069       TGG----ACGCGCCCTGTAGCGGCGCATTAA-----------------------------------------------------------------------------------------------------------------------------------------------------
lacZ-α-070       TGG---AAATTGTAA---------------------------------------------------------------------------------------------------------------------------------------------------------------------
lacZ-α-071       TGGG---ACGCGCCCTGTAGCGGCGCATTAAGCGCGGCGGGTGTGGTGGTTACGCGCAGCGTGACCGCTACACTTGCCAGCGCCCTAGCGCCCGCTCCTTTCGCTTTCTTCCCTTCCTTTCTCGCCACGTTCGCCGGCTTTCCCCGTCAAGCTCTAAATCGGGGGCTCCCTTTAGGGTTC...
lacZ-α-072       TGGG---ACGCGCCCTGTAGCGGCGCATTAAGCGCGGCGGGTGTGGTGGTTACGCGCAGCGTGACCGCTACACTTGCCAGCGCCCTAGCGCCCGCTCCTTTCGCTTTCTTCCCTTCCTTTCTCGCCACGTTCGCCGGCTTTCCCCGTCAAGCTCTAAATCGGGGGCTCCCTTTAGGGTTC...
lacZ-α-074       TGCGATTTATTCAACAAAGCCGCCGTCCCGTCAAGTCAGCGTAATGCTCTGCCAGTGTTACAACCAATTAACCAATTCTGA---------------------------------------------------------------------------------------------------
lacZ-α-075       TGG---AAATTGTAA---------------------------------------------------------------------------------------------------------------------------------------------------------------------
```

Sequences in yellow correspond to the f1 origin of replication, which is in frame with lacZ-α in these cases.
lacZ-α-016 and lacZ-α-021 are missing STOP codons because these variants are in frame fusions with ccdB. -016 identical to -053. -021 identical to -029
Sequences in bold are incorrectly annotated
lacZ-α-041 sequence has a single bp deletion that generates a frame-shift and premature STOP codon. Very likely sequence error corrected above. -041 merged with -023
lacZ-α-036 is annotated without a STOP codon. Sequence actually extends another 54 bp and is identical to lacZ-α-052. -036 merged with -052.

Sequence Alignments

```
Name of             Alignment                                                                                                                 Plasmids
Variant
lacZ-α-040          ATGACCATGATTACGCCAAGCTATTTAGGTGACACTATAGAATACTCAAGCTATGCATCCAACGCGTTGG--GAGCTCTCCCATATGGTCGACCTGCAGGCGGCCGCGAATTCACTAGT-          pGEM-T
lacZ-α-062          ATGACCATGATTACGCCAAGCTATTTAGGTGACACTATAGAATACTCAAGCTATGCATCCAACGCGTTGG--GAGCTCTCCCATATGGTCGACCTGCAGGCGGCCGCGAATTCACTAGTA          pSpark Done (linearized)
lacZ-α-064          ------------------------------------------------------------------------------------------------------------------------          pSpark Done (linearized)
lacZ-α-048          ATGACCATGATTACGCCAAGCTATTTAGGTGACACTATAGAATACTCAAGCTATGCATCCAACGCGTTGG--GAGCTCTCCCATATG------GTCGACCTGCAGGCGGCCGCACTAGT-          pGEM-T Easy
lacZ-α-066          ATGACCATGATTACGCCAAGCTATTTAGGTGACACTATAGAATACTCAAGCTATGCATCCAACGCGTTGG--GAGCTCTCCCATATG------GTCGACCTGCAGGCGGCCGCACTAGTA          pSpark II (linearized)
lacZ-α-069          ------------------------------------------------------------------------------------------------------------------------          pSpark II (linearized)
lacZ-α-073          ATGACCATGATTACGCCAAGCTATTTAGGTGACACTATAGAATACTCAAGCTATGCATCCAACGCGTTACTCGAGCTCAAACATATG---AAAGTCGACCTGCAGGCGGCCGCACTAGTG          pSpark III, IV and V (linearized)
lacZ-α-076          ------------------------------------------------------------------------------------------------------------------------          pSpark III, IV and V (linearized)


lacZ-α-040          ------GATATC--------------GAATTCCCGCGGCCGCCATGGCGGCCGGGAGCATGCGACGTCGGGCCCAATTCGCCCTATAGTGAGTCGTATTACAATTCACTGGCCGTCGTTT
lacZ-α-062          ------GAT---------------------------------------------------------------------------------------------------------------
lacZ-α-064          ---------ATC--------------GAATTCCCGCGGCCGCCATGGCGGCCGGGAGCATGCGACGTCGGGCCCAATTCGCCCTATAGTGAGTCGTATTACAATTCACTGGCCGTCGTTT
lacZ-α-048          ------GATATC--------------------CCGCGG---CCATGGCGGCCGGGAGCATGCGACGTCGGGCCCAATTCGCCCTATAGTGAGTCGTATTACAATTCACTGGCCGTCGTTT
lacZ-α-066          ------GAT---------------------------------------------------------------------------------------------------------------
lacZ-α-069          ---------ATC--------------------CCGCGG---CCATGGCGGCCGGGAGCATGCGACGTCGGGCCCAATTCGCCCTATAGTGAGTCGTATTACAATTCACTGGCCGTCGTTT
lacZ-α-073          GATCCTGAT---------------------------------------------------------------------------------------------------------------
lacZ-α-076          ---------ATCAGGCCTGATCCGGAGAATTCGTTTAAAC-CCATGG---------------------GGGCCCAATTCGCCCTATAGTGAGTCGTATTACAATTCACTGGCCGTCGTTT


lacZ-α-040          TACAACGTCGTGACTGGGAAAACCCTGGCGTTACCCAACTTAATCGCCTTGCAGCACATCCCCCTTTCGCCAGCTGGCGTAATAGCGAAGAG...GACGCGCCCTGTAGCGGCGCATTAA
lacZ-α-062          ------------------------------------------------------------------------------------------------------------------------
lacZ-α-064          TACAACGTCGTGACTGGGAAAACCCTGGCGTTACCCAACTTAATCGCCTTGCAGCACATCCCCCTTTCGCCAGCTGGCGTAATAGCGAAGAG...GACGCGCCCTGTAGCGGCGCATTAA
lacZ-α-048          TACAACGTCGTGACTGGGAAAACCCTGGCGTTACCCAACTTAATCGCCTTGCAGCACATCCCCCTTTCGCCAGCTGGCGTAATAGCGAAGAG...GACGCGCCCTGTAGCGGCGCATTAA
lacZ-α-066          ------------------------------------------------------------------------------------------------------------------------
lacZ-α-069          TACAACGTCGTGACTGGGAAAACCCTGGCGTTACCCAACTTAATCGCCTTGCAGCACATCCCCCTTTCGCCAGCTGGCGTAATAGCGAAGAG...GACGCGCCCTGTAGCGGCGCATTAA
lacZ-α-073          ------------------------------------------------------------------------------------------------------------------------
lacZ-α-076          TACAACGTCGTGACTGGGAAAACCCTGGCGTTACCCAACTTAATCGCCTTGCAGCACATCCCCCTTTCGCCAGCTGGCGTAATAGCGAAGAG...GACGCGCCCTGTAGCGGCGCATTAA
```

Combined lacZ-α Fragments from Recircularized pSpark Series

```
Name of             Alignment                                                                   Common Restriction Enzyme Sites in MCS
Variant
                                                                                           SacI      NdeI     SalI PstI NotI    EcoRI  SpeI
lacZ-α-040          ATGACCATGATTACGCCAAGCTATTTAGGTGACACTATAGAATACTCAAGCTATGCATCCAACGCGTTGG--GAGCTCTCCCATATG---GTCGACCTGCAGGCGGCCGCGAATTCACTA
lacZ-α-062+064      ATGACCATGATTACGCCAAGCTATTTAGGTGACACTATAGAATACTCAAGCTATGCATCCAACGCGTTGG--GAGCTCTCCCATATG---GTCGACCTGCAGGCGGCCGCGAATTCACTA
lacZ-α-048          ATGACCATGATTACGCCAAGCTATTTAGGTGACACTATAGAATACTCAAGCTATGCATCCAACGCGTTGG--GAGCTCTCCCATATG---GTCGACCTGCAGGCGGCCGC------ACTA
lacZ-α-066+069      ATGACCATGATTACGCCAAGCTATTTAGGTGACACTATAGAATACTCAAGCTATGCATCCAACGCGTTGG--GAGCTCTCCCATATG---GTCGACCTGCAGGCGGCCGC------ACTA
lacZ-α-073+076      ATGACCATGATTACGCCAAGCTATTTAGGTGACACTATAGAATACTCAAGCTATGCATCCAACGCGTTACTCGAGCTCAAACATATGAAAGTCGACCTGCAGGCGGCCGC------ACTA
                    ********************************************************************    ******   ******   ********************      ****

                              EcoRV SacII        EcoRI SacII NotI              SphI
lacZ-α-040          GT-------GATATC--------------GAATTCCCGCGGCCGCCATGGCGGCCGGGAGCATGCGACGTCGGGCCCAATTCGCCCTATAGTGAGTCGTATTACAATTCACTGGCCGTCG
lacZ-α-062+064      GTA------GATATC--------------GAATTCCCGCGGCCGCCATGGCGGCCGGGAGCATGCGACGTCGGGCCCAATTCGCCCTATAGTGAGTCGTATTACAATTCACTGGCCGTCG
lacZ-α-048          GT-------GATATCCCGCGGCC-----------------------ATGGCGGCCGGGAGCATGCGACGTCGGGCCCAATTCGCCCTATAGTGAGTCGTATTACAATTCACTGGCCGTCG
lacZ-α-066+069      GTA------GATATCCCGCGGCC-----------------------ATGGCGGCCGGGAGCATGCGACGTCGGGCCCAATTCGCCCTATAGTGAGTCGTATTACAATTCACTGGCCGTCG
lacZ-α-073+076      GTGGATCCTGATATCAGGCCTGATCCGGAGAATTC-GTTTAAACCCATGG---------------------GGGCCCAATTCGCCCTATAGTGAGTCGTATTACAATTCACTGGCCGTCG
                    **       ******                               ****                     *************************************************

lacZ-α-040          TTTTACAACGTCGTGACTGGGAAAACCCTGGCGTTACCCAACTTAATCGCCTTGCAGCAC...TCCCAACAGTTGCGCAGCCTGAATGGCGAATGGACGCGCCCTGTAGCGGCGCATTAA
lacZ-α-062+064      TTTTACAACGTCGTGACTGGGAAAACCCTGGCGTTACCCAACTTAATCGCCTTGCAGCAC...TCCCAACAGTTGCGCAGCCTGAATGGCGAATGGACGCGCCCTGTAGCGGCGCATTAA
lacZ-α-048          TTTTACAACGTCGTGACTGGGAAAACCCTGGCGTTACCCAACTTAATCGCCTTGCAGCAC...TCCCAACAGTTGCGCAGCCTGAATGGCGAATGGACGCGCCCTGTAGCGGCGCATTAA
lacZ-α-066+069      TTTTACAACGTCGTGACTGGGAAAACCCTGGCGTTACCCAACTTAATCGCCTTGCAGCAC...TCCCAACAGTTGCGCAGCCTGAATGGCGAATGGACGCGCCCTGTAGCGGCGCATTAA
lacZ-α-073+076      TTTTACAACGTCGTGACTGGGAAAACCCTGGCGTTACCCAACTTAATCGCCTTGCAGCAC...TCCCAACAGTTGCGCAGCCTGAATGGCGAATGGACGCGCCCTGTAGCGGCGCATTAA
                    ************************************************************   *********************************************************
```

lacZ-α-062, lacZ-α–066 and lacZ-α–073 are linearized plasmids and have added A at end possibly because vector is like Topo with 3′ A overhang to make PCR cloning more efficient. A is removed in alignment. Plasmid comes linearized by EcoRV.

lacZ-α–064, lacZ-α–069 and lacZ-α–076 missing A at start to complete EcoRV site of linearization (blunt) added in alignment.

Sequence Alignments

**Name of Variant**	**Alignment of Translated Protein**	**Size (aa)**
**lacZ-α-041**	**MTMITPSSELTLTKGNKSWSSTAVAAALELVDPPGCRN---RYQAYRYRRPRGGARYPIRPIVSRITIHWPSFYNVVTGKTLALPNLIALQHIP**	**113**
lacZ-α-041_corr	MTMITPSSELTLTKGNKSWSSTAVAAALELVDPPGCRNSISSLSIPSTSRGGP---VPNS--PYSESYY-NSLAVVLQRRDWENPGVTQLNRLAAHPP	141
lacZ-α-023	MTMITPSSELTLTKGNKSWSSTAVAAALELVDPPGCRNSISSLSIPSTSRGGP---VPNS--PYSESYY-NSLAVVLQRRDWENPGVTQLNRLAAHPP	145
lacZ-α-032	MTMITPSSELTLTKGNKSWSSTAVAAALELVDPPGCRNSISSLSIPSTSRGGP---VPNS--PYSESYY-NSLAVVLQRRDWENPGVTQLNRLAAHPP	129
lacZ-α-047	MTMITPSSELTLTKGNKSWSSTAVAAALELVDPPGCRNSISSLSIPSTSRGGP---VPNS--PYSESYY-NSLAVVLQRRDWENPGVTQLNRLAAHPP	216
lacZ-α-012	MTMITPSSELTLTKGNKSWSSTAVAAALELVDPPGCRNSISSLSIPSTSRGGP---VPNS--PYSESYY-NSLAVVLQRRDWENPGVTQLNRLAAHPP	120
lacZ-α-015	MTMITPSAQLTLTKGNKSWSSTAVAAALELVDPPGCRNSISSLSIPSTSRGGP---VPNS--PYSESYYARSLAVVLQRRDWENPGVTQLNRLAAHPP	191
lacZ-α-031	MTMITPSAQLTLTKGNKSWSSTAVAAALELVDPPGCRNSISSLSIPSTSRGGP---VPNS--PYSESYYARSLAVVLQRRDWENPGVTQLNRLAAHPP	192
lacZ-α-005	MTMITPSAQLTLTKGNKSWSSTAVAAALARADPPGCRNSISSLSIPSTSRGGP---VPNS--PYSESYYARSLAVVLQRRDWENPGVTQLNRLAAHPP	191
lacZ-α-025	MTMITPSSKLTLTKGNKSWSSTAVAAALELVDPPGCRNSISSLSIPSTSRGGP---VPNS--PYSESYY-NSLAVVLQRRDWENPGVTQLNRLAAHPP	190
lacZ-α-042	MTMITPSSELTLTKGNKSWSSTAVAAALELVDPPGCRNSISSLSIPSTSRGGP---VPNS--PYSESYY-NSLAVVLQRRDWENPGVTQLNRLAAHPP	191
lacZ-α-019	MTMITPSSKLTLTKGNKSWVPGPPSRSTVSISLISNSCSPGD---PLVLERPPPRWSSNS--PYSESYY-NSLAVVLQRRDWENPGVTQLNRLAAHPP	190
lacZ-α-037	MTMITPSSELTLTKGNKSWVPGPPSRSTVSISLISNSCSPGD---PLVLERPPPRWSSNS--PYSESYY-NSLAVVLQRRDWENPGVTQLNRLAAHPP	191
lacZ-α-010	MTMITPSAQLTLTKGNKSWVPGPPSRSTVSISLISNSCSPGD---PLVLERPPPRWSSNS--PYSESYYARSLAVVLQRRDWENPGVTQLNRLAAHPP	191
lacZ-α-055	MTMITPSAQLTLTKGNKSWVPGPPSRSTVSISLISNSCSPNDGQQGQTMD---PLVLERPPPRWSSNS--PYSESYYARSLAVVLQRRDWENPGVTQLNRLAAHPP	129
lacZ-α-038	MTMITPSSELTLTKGTSPAGLNEFALKGEFAAAKF---NS--PYSESYY-NSLAVVLQRRDWENPGVTQLNRLAAHPP	94
lacZ-α-028	MTMITPS-YLGDTIEY---SSLHACR--STLED-------------------------PRVPSSNS--PYSESYY-NSLAVVLQRRDWENPGVTQLNRLAAHPP	126
lacZ-α-017	MTLITPSSKLTLTKGNKSW-SSRACR--STLVDPKNSKSFSRVLLERPRAHRFSTRVGYQVSVPNS--PYSESYY-NSLAVVLQRRDWENPGVTQLNRLAAHPP	128
lacZ-α-049	MTMITPSSELTLTKGNK---SLHACR--STLED-------------------------PRVPSSNS--PYSESYY-NSLAVVLQRRDWENPGVTQLNRLAAHPP	170
lacZ-α-008	MTMITP--------------SLHACR---FKQSTLDL-------------------IKDPARPRVPSSNS------------LAVVLQRRDWENPGVTQLNRLAAHPP	116
lacZ-α-030	MTMITP--------------SLHACR--STLED-------------------------PRVPSSNS------------LAVVLQRRDWENPGVTQLNRLAAHPP	107
lacZ-α-065	MTMITP--------------SLHACR--STLED-------------------------PRVPSSNS------------LAVVLQRRDWENPGVTQLNRLAAHPP	168
lacZ-α-009	MTMITP--------------SLHACR--STLED-------------------------PRVPSSNS------------LAVVLQRRDWENPGVTQLNRLAAHPP	76
lacZ-α-057	MTMITP--------------SLHACR--STLED-------------------------PRVPSSNS------------LAVVLQRRDWENPGVTQLNRLAAHPP	107
**lacZ-α-036**	**MTMITP--------------SLHACR--STLED-------------------------PRVPSSNS------------LAVVLQRRDWENPGVTQLNRLAAHPP**	**95**
lacZ-α-036corr/052	MTMITP--------------SLHACR--STLED-------------------------PRVPSSNS------------LAVVLQRRDWENPGVTQLNRLAAHPP	112
lacZ-α-011	MTMITP--------------SLHACR--STLED-------------------------PRVPSSNS------------LAVVLQRRDWENPGVTQLNRLAAHPP	384
lacZ-α-001	MTMITP--------------SLHACR--STLED-------------------------PRVPSSNS------------LAVVLQRRDWENPGVTQLNRLAAHPP	108
lacZ-α-072	MTMITPSSNTTHYRE-----SLHACR--STLED-------------------------PRVPSSNS------------LAVVLQRRDWENPGVTQLNRLAAHPP	158
lacZ-α-070	MTMITPSSNTTHYRE-----SLHACR--STLED-------------------------PRVPSSNS------------LAVVLQRRDWENPGVTQLNRLAAHPP	88
lacZ-α-035	MTMITPSSNTTHYRET----SLHACR--STLED-------------------------PRVPSSNSYSIVSPKS--NSLAVVLQRRDWENPGVTQLNRLAAHPP	127
lacZ-α-033	MTMITPSSNTTHYRE-----SLHACR--STLED-----LLVIWISD------------PRVPSSNS------------LAVVLQRRDWENPGVTQLNRLAAHPP	110
lacZ-α-075	MTMITPSSNTTHYRE-----SLHAGLCSRRARDPISRCIRE---VPSSNS------------LAVVLQRRDWENPGVTQLNRLAAHPP	96
lacZ-α-022	MTMITP--------------SLHAGLCSRRARDPISRCIRE---VPSSNS------------LAVVLQRRDWENPGVTQLNRLAAHPP	115
lacZ-α-059	MTMITP--------------SLHAPRGVDSRGSPIDG---VPSSNS------------LAVVLQRRDWENPGVTQLNRLAAHPP	80
lacZ-α-016	MTMITPSYLGDTIEYSSYASSLVPSSDPLVTAASVLEFALKGEFCRYPSHWRPLEH---ASRGPNS--PYSESYY-NSLAVVLQRRDWENPGVTQLNRLAAHPP	120
lacZ-α-029	MTMITPSYLGDTIEYSSYASSLVPSSDPLVTAASVLEF------CRYPSHWRPLEH---ASRGPNS--PYSESYY-NSLAVVLQRRDWENPGVTQLNRLAAHPP	114
lacZ-α-021	MTMITPSYLGDALEYSSYASSLVPSSDPLVTAASVLEFALKGEFCRYPSHWRPLEH---ASRGPNS--PYSESYY-NSLAVVLQRRDWENPGVTQLNRLAAHPP	120
lacZ-α-027	MTMITP--------------SLVPSSDPLVTAASVLEFALKGEFCRYPSHWRPLEH---ASRGPNS--PYSESYY-NSLAVVLQRRDWENPGVTQLNRLAAHPP	117
lacZ-α-071	MTMITPSYLGDTIEYSSYASSLVPSSDPLVTAASVLEF------CRYPSHWRPLEH---ASRGPNS--PYSESYY-NSLAVVLQRRDWENPGVTQLNRLAAHPP	201
lacZ-α-040	MTMITPSYLGDTIEYSSYASNALGALPYGRPAGGREFTSDIEF-PRPP--WRPGACDV--GPNS--PYSESYY-NSLAVVLQRRDWENPGVTQLNRLAAHPP	127
lacZ-α-048	MTMITPSYLGDTIEYSSYASNALGALPYGRPAGGR--TSDI---PR-P--WRPGACDV--GPNS--PYSESYY-NSLAVVLQRRDWENPGVTQLNRLAAHPP	122
lacZ-α-068	MTMITPSYLGETIEYSSLHAIRIVNDGSDARA--NS--PYSESYY-NSLAVVLQRRDWENPGVTQLNRLAAHPP	90
lacZ-α-044	MTMITP-----------SWRAKLACLQVDSRGSPGTELE---FLINNS------------LAVVLQRRDWENPGVTQLNRLAAHPP	116
lacZ-α-002	MTMITTP--GQEFDIYISFLKLNKIILLANQDLSLAVVLQRRDWENPGVTQLNRLAAHPP	86
lacZ-α-003	MTMITTP---------------------------------NLINISGGGGGARDPLGLQERVLAGLHVYTAVRPQASNAYRWYRRRPAGAPVQNSDPRAPGISPWYISFLKLNKIILLANQDLSLAVVLQRRDWENPGVTQLNRLAAHPP	145
lacZ-α-006	MTMIT--NSRGSVDLQPSLALAVVLQRRDWENPGVTQLNRLAAHPP	66
lacZ-α-063	MTMIT--NSSSVPG------------------DPLESTCRHA-----------SLALAVVLQRRDWENPGVTQLNRLAAHPP	168
lacZ-α-004	MTMIT--NSSSVPG------------------DPLESTCRHA-----------SLALAVVLQRRDWENPGVTQLNRLAAHPP	108
lacZ-α-045	MTMIT--NSSSVPG------------------DPLESTCRHA-----------SLALAVVLQRRDWENPGVTQLNRLAAHPP	96
lacZ-α-039	MTMIT--NSSSVPG------------------DPLESTCRHA-----------SLALAVVLQRRDWENPGVTQLNRLAAHPP	100
lacZ-α-013	MTMIT--NSSSVPG------------------DPLESTCRHA-----------SLALAVVLQRRDWENPGVTQLNRLAAHPP	107
lacZ-α-034	MTMIT--NSSSVPG------------------DPLESTCRHA-----------SLALAVVLQRRDWENPGVTQLNRLAAHPP	76
lacZ-α-067	MAMIT--NSSSVPG------------------DPLESTCRHA-----------SLALAVVLQRRDWENPGVTQLNRLAAHPP	89
lacZ-α-054	--MIT--NSSSVPG------------------DPLESTCRHA-----------SLALAVVLQRRDWENPGVTQLNRLAAHPP	100
lacZ-α-074	MTMITP---NSSSVPG------------------DPLESTCRHA-----------SL-LAVVLQRRDWENPGVTQLNRLAAHPP	102
lacZ-α-060	MTMIT--NSSSVPG------------------DPLESTCRHA-----------SLALAVVLQRRDWENPGVTQLNRLAAHPP	77
lacZ-α-007	MTMIT---------------------NLIRLTIGNLNLIKARPWIDVNMHDITRGATSASRSGRPTGGPYGPAAANSSSVPTYSSRDVRSNNWFKRPGTWDPLESTCRHASFGPPRDNRVISLALAVVLQRRDWENPGVTQLNRLAAHPP	167
lacZ-α-024	MTMITPSSNTTHYRES--WYACRYRS-GIPGAAAVTSELVD----SRGSKLTYACMRRHSS--SIVSPKF--NSLAVVLQRRDWENPGVTQLNRLAAHPP	122
lacZ-α-018	MTMITPSSNTTHYRESSLVLFAEAQNKRSTLATMGVTKETAAAKFERQHIDSGTGGSDDDDKSPGFSSKLQTRYVSDPYLGDTIEFRYLVDKLLEPRLALDHTCGGPSSR----------PHNSLAVVLQRRDWENPGVTQLNRLAAHPP	182
lacZ-α-014	MTMITPSSNTTHYRESS--VPRMLQTRYVSDP-EFVISEF---VDKLLEPRLALDHTCGGPSSRPLYSIVSPKWPHNSLAVVLQRRDWENPGVTQLNRLAAHPP	126
lacZ-α-026	MTMITPSSNTTHYRESS--VPRMLQTRYVSDP-EFVISEF---VDKLLEPRLALDHTCGGPSSRPLYSIVSPKWPHNSLAVVLQRRDWENPGVTQLNRLAAHPP	126
lacZ-α-051	MTMITPSSNTTHYRESS--VPRMLQTRYVSDP-EFVISEF---VDKLLEPRLALDHTCGGPSSRPLYSIVSPKWPHNSLAVVLQRRDWENPGVTQLNRLAAHPP	126
lacZ-α-046	MTMITPSSNTTHYRESS--VPRMLQTRYVSDP-EFVISEF---VDKLLEPRLALDHTCGGPSSR----------PHNSLAVVLQRRDWENPGVTQLNRLAAHPP	116
lacZ-α-043	MTMITPSYVIRLTSGQIFECIARTVRLEEF--LQDIWIHEASHGDVTGSRY-LGELWYPLVKALSESYY--GLAVVLQRRDWENPGVTQLNRLAAHPP	123
lacZ-α-050	MTMITPSYVIRLTIGQIFECIARTVRLEEF--LQDIWIHEASHGDVTGSRY-LGELWYPLVKAYSESYY--GLAVVLQRRDWENPGVTQLNRLAAHPP	123
lacZ-α-056	MTMITPSYVIRLTSGARAIEAGWRQAS---LQDIWIHEFASFGRDASPD-VQACVDPLVKALSESYY--GLAVVLQRRDWENPGVTQLNRLAAHPP	120
lacZ-α-058	MTMITPSYVIRLTIGARAIEAGWRQAS---LQDIWIHEFASFGRDASPD-VQACVDPLVKAYSESYY--GLAVVLQRRDWENPGVTQLNRLAAHPP	120
lacZ-α-053	MTMITPSYVIRLTSGYQSSLGSRTGDVSHG--EAWIHDILQEFLETVRARECIQIFPLVKALSESYY--GLAVVLQRRDWENPGVTQLNRLAAHPP	122
lacZ-α-061	MTMITPSYVIRLTSGSTACLYTPEDAFGRAS---EFWIHDILQEAFWRLAG--AIEWAPLVKALSESYY--GLAVVLQRRDWENPGVTQLNRLAAHPP	121
lacZ-α-020	MTMITPSLSTN--SLAVVLQRRDWENPGVTQLNRLAAHPP	65
lacZ-α-062+064	MTMITPSYLGDTIEYSSYASNALGALPYGRPAGGREFTSRYRIPAAAMAAGSMRRR-AQFAL--	61
lacZ-α-066+069	MTMITPSYLGDTIEYSSYASNALGALPYGRPAGGR--TSRY---PAAMAAGSMRRR-AQFAL--	56
lacZ-α-073+076	MTMITPSYLGDTIEYSSYASNALLELKHMKVDLQA--AALVDPDIRPDPENSFKPMGAQFAL--	60
	:**** *************************	

Sequence Alignments

```
lacZ-α-041              LSPAGVIAKRPAPIALPNSCAA------------------------------------------------------------------------------------------------------
lacZ-α-041_corr         FASWRNSEEARTDRPSQQLRSLNGEWREIVNVNVIVPLLQKCQRYSLRRPKGY-----------------------------------------------------------------------
lacZ-α-023              FASWRNSEEARTDRPSQQLRSLNGEWREIVNVNVIVPLLQKCQRYSLRRPKGY-----------------------------------------------------------------------
lacZ-α-032              FASWRNSEEARTDRPSQQLRSLNGEWREIVNVNVIDT---------------------------------------------------------------------------------------
lacZ-α-047              FASWRNSEEARTDRPSQQLRSLNGEWREIVNVNVNVTPQYILPRSLIPGIGNPEAREVAAFRAFSFSKKKIYKTICCGRPPGCGQRRWRSTVGNRLRLSTGGAGARSALSTSQGRPIIDIYIHN
lacZ-α-012              FASWRNSEEARTDRPSQQLRSLNGEWKL------------------------------------------------------------------------------------------------
lacZ-α-015              FASWRNSEEARTDRPSQQLRSLNGEW-DAPCSGALSAAGVVVTRSVTATLASALAPAPFAFFPSFLATFAGFPRQALNRGLPLGFRFSALRHLDPKKLD-------------------------
lacZ-α-031              FASWRNSEEARTDRPSQQLRSLNGEWRDAPCSGALSAAGVVVTRSVTATLASALAPAPFAFFPSFLATFAGFPRQALNRGLPLGFRFSALRHLDPKKLD-------------------------
lacZ-α-005              FASWRNSEEARTDRPSQQLRSLNGEW-DAPCSGALSAAGVVVTRSVTATLASALAPAPFAFFPSFLATFAGFPRQALNRGLPLGFRFSALRHLDPKKLD-------------------------
lacZ-α-025              FASWRNSEEARTDRPSQQLRSLNGEW-DAPCSGALSAAGVVVTRSVTATLASALAPAPFAFFPSFLATFAGFPRQALNRGLPLGFRFSALRHLDPKKLD-------------------------
lacZ-α-042              FASWRNSEEARTDRPSQQLRSLNGEWRDAPCSGALSAAGVVVTRSVTATLASALAPAPFAFFPSFLATFAGFPRQALNRGLPLGFRFSALRHLDPKKLD-------------------------
lacZ-α-019              FASWRNSEEARTDRPSQQLRSLNGEW-DAPCSGALSAAGVVVTRSVTATLASALAPAPFAFFPSFLATFAGFPRQALNRGLPLGFRFSALRHLDPKKLD-------------------------
lacZ-α-037              FASWRNSEEARTDRPSQQLRSLNGEWRDAPCSGALSAAGVVVTRSVTATLASALAPAPFAFFPSFLATFAGFPRQALNRGLPLGFRFSALRHLDPKKLD-------------------------
lacZ-α-010              FASWRNSEEARTDRPSQQLRSLNGEW-DAPCSGALSAAGVVVTRSVTATLASALAPAPFAFFPSFLATFAGFPRQALNRGLPLGFRFSALRHLDPKKLD-------------------------
lacZ-α-055              FASWRNSEEARTDRPSQQLRSLNGEWKL------------------------------------------------------------------------------------------------
lacZ-α-038              FASWRNSEEARTDRPSQQLRSL------------------------------------------------------------------------------------------------------
lacZ-α-028              FASWRNSEEARTDRPSQQLRSLNGEWRLMRYFLLTHLCGISHRIWCTLSTICSDAA--------------------------------------------------------------------
lacZ-α-017              FASWRNSEEARTDRPSQQLRSLNGEWRSNF----------------------------------------------------------------------------------------------
lacZ-α-049              FASWRNSEEARTDRPSQQLRSLNGEWRDAPCSGALSAAGVVVTRSVTATLASALAPAPFAFFPSFLATFAGFPRQALNRGLPLGFRFSALRHLDPKKLD-------------------------
lacZ-α-008              FASWRNSEEARTDRPSQQLRSLNGEWRLMRYFLLTHLCGISHRIWCTLSTICSDAA--------------------------------------------------------------------
lacZ-α-030              FASWRNSEEARTDRPSQQLRSLNGARSFRFLPFLSRHVRRLSPSSSKSGAPFRVPI--------------------------------------------------------------------
lacZ-α-065              FASWRNSEEARTDRPSQQLRSLNGEWRFAWFPAPEAVPESWLECDLPEADTVVVPSNWQMHGYDAPIYTNVTYPITVNPPFVPTENPTGCYSLTFNVDESWLQEGQTRIIFDGVPIG-------
lacZ-α-009              FASWRNSEEARTDRPSQQLRSLNGE---------------------------------------------------------------------------------------------------
lacZ-α-057              FASWRNSEEARTDRPSQQLRSLNGEWRLMRYFLLTHLCGISHRIWCTLSTICSDAA--------------------------------------------------------------------
lacZ-α-036              FASWRNSEEARTDRPSQQLRSLNGEWRLMRYFLLTHLCGISHRI--------------------------------------------------------------------------------
lacZ-α-036corr/052      FASWRNSEEARTDRPSQQLRSLNGEWRLMRYFLLTHLCGISHRIYRWAILMKNILNLLSYY---------------------------------------------------------------
lacZ-α-011              FASWRNSEEARTDRPSQQLRSLNGEWRLMRYFLLTHLCGISHRIRQSNHSTRPVAAH-------------------------------------------------------------------
lacZ-α-001              FASWRNSEEARTDRPSQQLRSLNGECDLFNKAAVPSSQRNALPVLQPINQF-------------------------------------------------------------------------
lacZ-α-072              FASWRNSEEARTDRPSQQLRSLNGEW-DAPCSGALSAAGVVVTRSVTATLASALAPAPFAFFPSFLATFAGFPRQALNRGLPLGFRFSALRHLDPKKLD-------------------------
lacZ-α-070              FASWRNSEEARTDRPSQQLRSLNGEWKL------------------------------------------------------------------------------------------------
lacZ-α-035              FASWRNSEEARTDRPSQQLRSLNGEWRLMRYFLLTHLCGISHRIWCTLSTICSDAA--------------------------------------------------------------------
lacZ-α-033              FASWRNSEEARTDRPSQQLRSLNGEWEIVNVNILLKFALNFC----------------------------------------------------------------------------------
lacZ-α-075              FASWRNSEEARTDRPSQQLRSLNGEWKL------------------------------------------------------------------------------------------------
lacZ-α-022              FASWRNSEEARTDRPSQQLRSLNGEWRLMRYFLLTHLCGISHRIWCTLSTICSDAA--------------------------------------------------------------------
lacZ-α-059              FASWRNSEEARTDRPSQQLRSLNGE---------------------------------------------------------------------------------------------------
lacZ-α-016              FASWRNSEEARTDRPSQQLRSL------------------------------------------------------------------------------------------------------
lacZ-α-029              FASWRNSEEARTDRPSQQLRSL------------------------------------------------------------------------------------------------------
lacZ-α-021              FASWRNSEEARTDRPSQQLRSL------------------------------------------------------------------------------------------------------
lacZ-α-027              FASWRNSEEARTDRPSQQLRSLNGEWTRPVAAH-------------------------------------------------------------------------------------------
lacZ-α-071              FASWRNSEEARTDRPSQQLRSLNGEW-DAPCSGALSAAGVVVTRSVTATLASALAPAPFAFFPSFLATFAGFPRQALNRGLPLGFRFRALRHLDRKKLDLGDGSRSGPSP--------------
lacZ-α-040              FASWRNSEEARTDRPSQQLRSLNGEWTRPVAAH-------------------------------------------------------------------------------------------
lacZ-α-048              FASWRNSEEARTDRPSQQLRSLNGEWTRPVAAH-------------------------------------------------------------------------------------------
lacZ-α-068              FASWRNSEEARTDRPSQQLRS-------------------------------------------------------------------------------------------------------
lacZ-α-044              FASWRNSEEARTDRPSQQLRSLNGARSFRFLPFLSRHVRRLSPSSSKSGAPFRVPI--------------------------------------------------------------------
lacZ-α-002              FASWRNSEEARTDRPSQQLRSLNGEWKL------------------------------------------------------------------------------------------------
lacZ-α-003              FASWRNSEEARTDRPSQQLRSLNGEWKL------------------------------------------------------------------------------------------------
lacZ-α-006              FASWRNSEEARTDRPSQQLRSL------------------------------------------------------------------------------------------------------
lacZ-α-063              FASWRNSEEARTDRPSQQLRSLNGEWRFAWFPAPEAVPESWLECDLPEADTVVVPSNWQMHGYDAPIYTNVTYPITVNPPFVPTENPTGCYSLTFNVDESWLQEGQTRIIFDGVPIG-------
lacZ-α-004              FASWRNSEEARTDRPSQQLRSLNGEWRLMRYFLLTHLCGISHRIRQSNHSTRPVAAH-------------------------------------------------------------------
lacZ-α-045              FASWRNSEEARTDRPSQQLRSLNGEWRLMRYFLLTHLCGISHRIG-------------------------------------------------------------------------------
lacZ-α-039              FASWRNSEEARTDRPSQQLRSLNGEWRLMRYFLLTHLCGISHRIDPVRR---------------------------------------------------------------------------
lacZ-α-013              FASWRNSEEARTDRPSQQLRSLNGEWRLMRYFLLTHLCGISHRIWCTLSTICSDAA--------------------------------------------------------------------
lacZ-α-034              FASWRNSEEARTDRPSQQLRSLNGE---------------------------------------------------------------------------------------------------
lacZ-α-067              FASWRNSEEARTDRPSQQLRSLNGEWRLRNHYYHDINL--------------------------------------------------------------------------------------
lacZ-α-054              FASWRNSEEARTDRPSQQLRSLNGECDLFNKAAVPSSQRNALPVLQPINQF-------------------------------------------------------------------------
lacZ-α-074              FASWRNSEEARTDRPSQQLRSLNGECDLFNKAAVPSSQRNALPVLQPINQF-------------------------------------------------------------------------
lacZ-α-060              FASWRNSEEARTDRPSQQLRSLNGEC--------------------------------------------------------------------------------------------------
lacZ-α-007              FASWRNSEEARTDRPSQQLRSLNGEWEIVNVNILLIFC--------------------------------------------------------------------------------------
lacZ-α-024              FASWRNSEEARTDRPSQQLRSLNGEWTRPVAAH-------------------------------------------------------------------------------------------
lacZ-α-018              FASWRNSEEARTDRPSQQLRSLNGEWEIVNVNILLKFALNFC----------------------------------------------------------------------------------
lacZ-α-014              FASWRNSEEARTDRPSQQLRSLNGEWKL------------------------------------------------------------------------------------------------
lacZ-α-026              FASWRNSEEARTDRPSQQLRSLNGEWKL------------------------------------------------------------------------------------------------
lacZ-α-051              FASWRNSEEARTDRPSQQLRSLNGEWKL------------------------------------------------------------------------------------------------
lacZ-α-046              FASWRNSEEARTDRPSQQLRSLNGEWKL------------------------------------------------------------------------------------------------
lacZ-α-043              FASWRNSEEARTDRPSQQLRSLNGEWRFAW----------------------------------------------------------------------------------------------
lacZ-α-050              FASWRNSEEARTDRPSQQLRSLNGEWRFAW----------------------------------------------------------------------------------------------
lacZ-α-056              FASWRNSEEARTDRPSQQLRSLNGEWRFAW----------------------------------------------------------------------------------------------
lacZ-α-058              FASWRNSEEARTDRPSQQLRSLNGEWRFAW----------------------------------------------------------------------------------------------
lacZ-α-053              FASWRNSEEARTDRPSQQLRSLNGEWRFAW----------------------------------------------------------------------------------------------
lacZ-α-061              FASWRNSEEARTDRPSQQLRSLNGEWRFAW----------------------------------------------------------------------------------------------
lacZ-α-020              FASWRNSEEARTDRPSQQLRSLNGEWR-------------------------------------------------------------------------------------------------
lacZ-α-062+064          ----------------------------------------------------------------------------------------------------------------------------
lacZ-α-066+069          ----------------------------------------------------------------------------------------------------------------------------
lacZ-α-073+076          ----------------------------------------------------------------------------------------------------------------------------
                        ************************
```

Key
ATGC = change in nucleotide relative to consensus sequence – synonymous
ATGC = change in nucleotide relative to consensus sequence – conservative/semi-conservative amino acid substitution
ATGC = change in nucleotide relative to consensus sequence – non-conservative amino acid substitution

Sequence Alignments

```
Name of   # of    Alignment                                                                                                                  Size   # Var  Sources*
Variant   Occur                                                                                                                              (bp)   bp
MBP-004   10    ------------------------------------------------------------------------ATG---AAAATCGAAGAAGGTAAACTGGTAATCTGGATTAACGGCGAT   1101   0      16497515
MBP-010   10    ------------------------------------------------------------------------------AAAATCGAAGAAGGTAAACTGGTAATCTGGATTAACGGCGAT   1098   3d     20213425
MBP-001   7     ------------------------------------------------------------------------------AAAATCGAAGAAGGTAAACTGGTAATCTGGATTAACGGCGAT   1098   1/3d   16377204
MBP-002   2     ------------------------------------------------------------------------ATGGGTAAAATCGAAGAAGGTAAACTGGTAATCTGGATTAACGGCGAT   1104   2      16377204
MBP-009   2     ------------------------------------------------------------------------ATGGC------CGAAGAAGGTAAACTGGTAATCTGGATTAACGGCGAT   1098   4/3d   AB
MBP-003   1     ------------------------------------------------------------------------ATG---AAAATCGAAGAAGGTAAACTGGTAATCTGGATTAACGGCGAT   1101   1      19906724
MBP-005   1     ------------------------------------------------------------------------------AAAACTGAAGAAGGTAAACTGGTAATCTGGATTAACGGCGAT   1098   3      19906724
MBP-006   1     ------------------------------------------------------------------------------AAAATCGAAGAAGGTAAACTGGTAATCTGGATTAACGGCGAT   1074   14     MCSG
MBP-007   1     ATGAAAATAAAAACAGGTGCACGCATCCTCGCATTATCCGCATTAACGACGATGATGTTTTCCGCCTCGGCTCTCGCCAAAATCGAAGAAGGTAAACTGGTAATCTGGATTAACGGCGAT   1176   3      ?
MBP-008   1     ------------------------------------------------------------------------------AAAATCGAAGAAGGTAAACTGGTAATCTGGATTAACGGCGAT   1074   1/3d   MCSG
                                                                                                    ************************************

MBP-004         AAAGGCTATAACGGTCTCGCTGAAGTCGGTAAGAAATTCGAGAAAGATACCGGAATTAAAGTCACCGTTGAGCATCCGGATAAACTGGAAGAGAAATTCCCACAGGTTGCGGCAACTGGC
MBP-010         AAAGGCTATAACGGTCTCGCTGAAGTCGGTAAGAAATTCGAGAAAGATACCGGAATTAAAGTCACCGTTGAGCATCCGGATAAACTGGAAGAGAAATTCCCACAGGTTGCGGCAACTGGC
MBP-001         AAAGGCTATAACGGTCTCGCTGAAGTCGGTAAGAAATTCGAGAAAGATACCGGAATTAAAGTCACCGTTGAGCATCCGGATAAACTGGAAGAGAAATTCCCACAGGTTGCGGCAACTGGC
MBP-002         AAAGGCTATAACGGTCTCGCTGAAGTCGGTAAGAAATTCGAGAAAGATACCGGAATTAAAGTCACCGTTGAGCATCCGGATAAACTGGAAGAGAAATTCCCACAGGTTGCGGCAACTGGC
MBP-009         AAAGGCTATAACGGTCTCGCTGAAGTCGGTAAGAAATTCGAGAAAGATACCGGAATTAAAGTCACCGTTGAGCATCCGGATAAACTGGAAGAGAAATTCCCACAGGTTGCGGCAACAGGC
MBP-003         AAAGGCTATAACGGTCTCGCTGAAGTCGGTAAGAAATTCGAGAAAGATACCGGAATTAAAGTCACCGTTGAGCATCCGGATAAACTGGAAGAGAAATTCCCACAGGTTGCGGCAACTGGC
MBP-005         AAAGGCTATAACGGTCTCGCTGAAGTCGGTAAGAAATTCGAGAAAGATACCGGAATTAAAGTCACCGTTGAGCATCCGGATAAACTGGAAGAGAAATTCCCACAGGTTGCGGCAACTGGC
MBP-006         AAAGGCTATAACGGTCTCGCTGAAGTCGGTAAGAAATTCGAGAAAGATACCGGAATTAAAGTCACCGTTGAGCATCCGGATAAACTGGAAGAGAAATTCCCACAGGTTGCGGCAACTGGC
MBP-007         AAAGGCTATAACGGTCTCGCTGAAGTCGGTAAGAAATTCGAGAAAGATACCGGAATTAAAGTCACCGTTGAGCATCCGGATAAACTGGAAGAGAAATTCCCACAGGTTGCGGCAACTGGC
MBP-008         AAAGGCTATAACGGTCTCGCTGAAGTCGGTAAGAAATTCGAGAAAGATACCGGAATTAAAGTCACCGTTGAGCATCCGGATAAACTGGAAGAGAAATTCCCACAGGTTGCGGCAACTGGC
                ******************************************************************************************************************** ***

MBP-004         GATGGCCCTGACATTATCTTCTGGGCACACGACCGCTTTGGTGGCTACGCTCAATCTGGCCTGTTGGCTGAAATCACCCCGGACAAAGCGTTCCAGGACAAGCTGTATCCGTTTACCTGG
MBP-010         GATGGCCCTGACATTATCTTCTGGGCACACGACCGCTTTGGTGGCTACGCTCAATCTGGCCTGTTGGCTGAAATCACCCCGGACAAAGCGTTCCAGGACAAGCTGTATCCGTTTACCTGG
MBP-001         GATGGCCCTGACATTATCTTCTGGGCACACGACCGCTTTGGTGGCTACGCTCAATCTGGCCTGTTGGCTGAAATCACCCCGGACAAAGCGTTCCAGGACAAGCTGTATCCGTTTACCTGG
MBP-002         GATGGCCCTGACATTATCTTCTGGGCACACGACCGCTTTGGTGGCTACGCTCAATCTGGCCTGTTGGCTGAAATCACCCCGGACAAAGCGTTCCAGGACAAGCTGTATCCGTTTACCTGG
MBP-009         GATGGCCCTGACATTATCTTCTGGGCACACGACCGCTTTGGTGGCTACGCTCAATCTGGCCTGTTGGCTGAAATCACCCCGGACAAAGCGTTCCAGGACAAGCTGTATCCGTTTACCTGG
MBP-003         GATGGCCCTGACATTATCTTCTGGGCACACGACCGCTTTGGTGGCTACGCTCAATCTGGCCTGTTGGCTGAAATCACCCCGGACAAAGCGTTCCAGGACAAGCTGTATCCGTTTACCTGG
MBP-005         GATGGCCCTGACATTATCTTCTGGGCACACGACCGCTTTGGTGGCTACGCTCAATCTGGCCTGTTGGCTGAAATCACCCCGGACAAAGCGTTCCAGGACAAGCTGTATCCGTTTACCTGG
MBP-006         GATGGCCCTGACATTATCTTCTGGGCACACGACCGCTTTGGTGGCTACGCTCAATCTGGCCTGTTGGCTGAAATCACCCCGGCAGCAGCGTTCCAGGACAAGCTGTATCCGTTTACCTGG
MBP-007         GATGGCCCTGACATTATCTTCTGGGCACACGACCGCTTTGGTGGCTACGCTCAATCTGGCCTGTTGGCTGAAATCACCCCGGACAAAGCGTTCCAGGACAAGCTGTATCCGTTTACCTGG
MBP-008         GATGGCCCTGACATTATCTTCTGGGCACACGACCGCTTTGGTGGCTACGCTCAATCTGGCCTGTTGGCTGAAATCACCCCGGACAAAGCGTTCCAGGACAAGCTGTATCCGTTTACCTGG
                **********************************************************************************    **********************************

MBP-004         GATGCCGTACGTTACAACGGCAAGCTGATTGCTTACCCGATCGCTGTTGAAGCGTTATCGCTGATTTATAACAAAGATCTGCTGCCGAACCCGCCAAAAACCTGGGAAGAGATCCCGGCG
MBP-010         GATGCCGTACGTTACAACGGCAAGCTGATTGCTTACCCGATCGCTGTTGAAGCGTTATCGCTGATTTATAACAAAGATCTGCTGCCGAACCCGCCAAAAACCTGGGAAGAGATCCCGGCG
MBP-001         GATGCCGTACGTTACAACGGCAAGCTGATTGCTTACCCGATCGCTGTTGAAGCGTTATCGCTGATTTATAACAAAGATCTGCTGCCGAACCCGCCAAAAACCTGGGAAGAGATCCCGGCG
MBP-002         GATGCGGTACGTTACAACGGCAAGCTGATTGCTTACCCGATCGCTGTTGAAGCGTTATCGCTGATTTATAACAAAGATCTGCTGCCGAACCCGCCAAAAACCTGGGAAGAGATCCCGGCG
MBP-009         GATGCCGTACGTTACAACGGCAAGCTGATTGCTTACCCGATCGCTGTTGAAGCGTTATCGCTGATTTATAACAAAGATCTGCTGCCGAACCCGCCAAAAACCTGGGAAGAGATCCCGGCG
MBP-003         GATGCCGTACGTTACAACGGCAAGCTGATTGCTTACCCGATCGCTGTTGAAGCGTTATCGCTGATTTATAACAAAGATCTGCTGCCGAACCCGCCAAAAACCTGGGAAGAGATCCCGGCG
MBP-005         GATGCCGTACGTTACAACGGCAAGCTGATTGCTTACCCGATCGCTGTTGAAGCGTTATCGCTGATTTATAACAAAGATCTGCTGCCGAACCCGCCAAAAACCTGGGAAGAGATCCCGGCG
MBP-006         GATGCCGTACGTTACAACGGCAAGCTGATTGCTTACCCGATCGCTGTTGAAGCGTTATCGCTGATTTATAACAAAGATCTGCTGCCGAACCCGCCAAAAACCTGGGAAGAGATCCCGGCG
MBP-007         GATGCCGTACGTTACAACGGCAAGCTGATTGCTTACCCGATCGCTGTTGAAGCGTTATCGCTGATTTATAACAAAGATCTGCTGCCGAACCCGCCAAAAACCTGGGAAGAGATCCCGGCG
MBP-008         GATGCCGTACGTTACAACGGCAAGCTGATTGCTTACCCGATCGCTGTTGAAGCGTTATCGCTGATTTATAACAAAGATCTGCTGCCGAACCCGCCAAAAACCTGGGAAGAGATCCCGGCG
                ***** **************************************************************************************************************

MBP-004         CTGGATAAAGAACTGAAAGCGAAAGGTAAGAGCGCGCTGATGTTCAACCTGCAAGAACCGTACTTCACCTGGCCGCTGATTGCTGCTGACGGGGGTTATGCGTTCAAGTATGAAAACGGC
MBP-010         CTGGATAAAGAACTGAAAGCGAAAGGTAAGAGCGCGCTGATGTTCAACCTGCAAGAACCGTACTTCACCTGGCCGCTGATTGCTGCTGACGGGGGTTATGCGTTCAAGTATGAAAACGGC
MBP-001         CTGGATAAAGAACTGAAAGCGAAAGGTAAGAGCGCGCTGATGTTCAACCTGCAAGAACCGTACTTCACCTGGCCGCTGATTGCTGCTGACGGGGGTTATGCGTTCAAGTATGAAAACGGC
MBP-002         CTGGATAAAGAACTGAAAGCGAAAGGTAAGAGCGCGCTGATGTTCAACCTGCAAGAACCGTACTTCACCTGGCCGCTGATTGCTGCTGACGGGGGTTATGCGTTCAAGTATGAAAACGGC
MBP-009         CTGGATAAAGAACTGAAAGCGAAAGGTAAGAGCGCGCTGATGTTCAACCTGCAAGAACCGTACTTCACCTGGCCGCTGATTGCTGCTGACGGGGGTTATGCGTTCAAGTATGAAAACGGC
MBP-003         CTGGATAAAGAACTGAAAGCGAAAGGTAAGAGCGCGCTGATGTTCAACCTGCAAGAACCGTACTTCACCTGGCCGCTGATTGCTGCTGACGGGGGTTATGCGTTCAAGTATGAAAACGGC
MBP-005         CTGGATAAAGAACTGAAAGCGAAAGGTAAGAGCGCGCTGATGTTCAACCTGCAAGAACCGTACTTCACCTGGCCGCTGATTGCTGCTGACGGGGGTTATGCGTTCAAGTATGAAAACGGC
MBP-006         CTGGATAAAGAACTGAAAGCGAAAGGTAAGAGCGCGCTGATGTTCAACCTGCAAGAACCGTACTTCACCTGGCCGCTGATTGCTGCTGACGGGGGTTATGCGTTCAAGTATGCAGCAGGC
MBP-007         CTGGATAAAGAACTGAAAGCGAAAGGTAAGAGCGCGCTGATGTTCAACCTGCAAGAACCGTACTTCACCTGGCCGCTGATTGCTGCTGACGGGGGTTATGCGTTCAAGTATGAAAACGGC
MBP-008         CTGGATAAAGAACTGAAAGCGAAAGGTAAGAGCGCGCTGATGTTCAACCTGCAAGAACCGTACTTCACCTGGCCGCTGATTGCTGCTGACGGGGGTTATGCGTTCAAGTATGAAAACGGC
                **************************************************************************************************************** *   ***

MBP-004         AAGTACGACATTAAAGACGTGGGCGTGGATAACGCTGGCGCGAAAGCGGGTCTGACCTTCCTGGTTGACCTGATTAAAAACAAACACATGAATGCAGACACCGATTACTCCATCGCAGAA
MBP-010         AAGTACGACATTAAAGACGTGGGCGTGGATAACGCTGGCGCGAAAGCGGGTCTGACCTTCCTGGTTGACCTGATTAAAAACAAACACATGAATGCAGACACCGATTACTCCATCGCAGAA
MBP-001         AAGTACGACATTAAAGACGTGGGCGTGGATAACGCTGGCGCGAAAGCGGGTCTGACCTTCCTGGTTGACCTGATTAAAAACAAACACATGAATGCAGACACCGATTACTCCATCGCAGAA
MBP-002         AAGTACGACATTAAAGACGTGGGCGTGGATAACGCTGGCGCGAAAGCGGGTCTGACCTTCCTGGTTGACCTGATTAAAAACAAACACATGAATGCAGACACCGATTACTCCATCGCAGAA
MBP-009         AAGTACGACATTAAAGACGTGGGCGTGGATAACGCTGGCGCGAAAGCGGGTCTGACCTTCCTGGTTGACCTGATTAAAAACAAACACATGAATGCAGACACCGATTACTCCATCGCAGAA
MBP-003         AAGTACGACATTAAAGACGTGGGCGTGGATAACGCTGGCGCGAAAGCGGGTCTGACCTTCCTGGTTGACCTGATTAAAAACAAACACATGAATGCAGACACCGATTACTCCATCGCAGAA
MBP-005         AAGTACGACATTAAAGACGTGGGCGTGGATAACGCTGGCGCGAAAGCGGGTCTGACCTTCCTGGTTGACCTGATTAAAAACAAACACATGAATGCAGACACCGATTACTCCATCGCAGAA
MBP-006         AAGTACGACATTAAAGACGTGGGCGTGGATAACGCTGGCGCGAAAGCGGGTCTGACCTTCCTGGTTGACCTGATTAAAAACAAACACATGAATGCAGACACCGATTACTCCATCGCAGAA
MBP-007         AAGTACGACATTAAAGACGTGGGCGTGGATAACGCTGGCGCGAAAGCGGGTCTGACCTTCCTGGTTGACCTGATTAAAAACAAACACATGAATGCAGACACCGATTACTCCATCGCAGAA
MBP-008         AAGTACGACATTAAAGACGTGGGCGTGGATAACGCTGGCGCGAAAGCGGGTCTGACCTTCCTGGTTGACCTGATTAAAAACAAACACATGAATGCAGACACCGATTACTCCATCGCAGAA
                ************************************************************************************************************************
```

Sequence Alignments

```
MBP-004             GCTGCCTTTAATAAAGGCGAAACAGCGATGACCATCAACGGCCCGTGGGCATGGTCCAACATCGACACCAGCAAAGTGAATTATGGTGTAACGGTACTGCCGACCTTCAAGGGTCAACCA
MBP-010             GCTGCCTTTAATAAAGGCGAAACAGCGATGACCATCAACGGCCCGTGGGCATGGTCCAACATCGACACCAGCAAAGTGAATTATGGTGTAACGGTACTGCCGACCTTCAAGGGTCAACCA
MBP-001             GCTGCCTTTAATAAAGGCGAAACAGCGATGACCATCAACGGCCCGTGGGCATGGTCCAACATCGACACCAGCAAAGTGAATTATGGTGTAACGGTACTGCCGACCTTCAAGGGTCAACCA
MBP-002             GCTGCCTTTAATAAAGGCGAAACAGCGATGACCATCAACGGCCCGTGGGCATGGTCCAACATCGACACCAGCAAAGTGAATTATGGTGTAACGGTACTGCCGACCTTCAAGGGTCAACCA
MBP-009             GCTGCCTTTAATAAAGGCGAAACAGCGATGACCATCAACGGCCCGTGGGCATGGTCCAACATCGACACCAGCAAAGTGAATTATGGTGTAACGGTACTGCCGACCTTCAAGGGTCAACCA
MBP-003             GCTGCCTTTAATAAAGGCGAAACAGCGATGACCATCAACGGCCCGTGGGCATGGTCCAACATCGACACCAGCAAAGTGAATTATGGTGTAACGGTACTGCCGACCTTCAAGGGTCAACCA
MBP-005             GCTGCCTTTAATAAAGGCGAAACAGCGATGACCATCAACGGCCCGTGGGCATGGTCCAACATCGACACCAGCAAAGTGAATTATGGTGTAACGGTACTGCCGACCTTCAAGGGTCAACCA
MBP-006             GCTGCCTTTAATAAAGGCGAAACAGCGATGACCATCAACGGCCCGTGGGCATGGTCCAACATCGACACCAGCGCAGTGAATTATGGTGTAACGGTACTGCCGACCTTCAAGGGTCAACCA
MBP-007             GCTGCCTTTAATAAAGGCGAAACAGCGATGACCATCAACGGCCCGTGGGCATGGTCCAACATCGACACCAGCAAAGTGAATTATGGTGTAACGGTACTGCCGACCTTCAAGGGTCAACCA
MBP-008             GCTGCCTTTAATAAAGGCGAAACAGCGATGACCATCAACGGCCCGTGGGCATGGTCCAACATCGACACCAGCAAAGTGAATTATGGTGTAACGGTACTGCCGACCTTCAAGGGTCAACCA
                    ************************************************************************  **********************************************

MBP-004             TCCAAACCGTTCGTTGGCGTGCTGAGCGCAGGTATTAACGCCGCCAGTCCGAACAAAGAGCTGGCAAAAGAGTTCCTCGAAAACTATCTGCTGACTGATGAAGGTCTGGAAGCGGTTAAT
MBP-010             TCCAAACCGTTCGTTGGCGTGCTGAGCGCAGGTATTAACGCCGCCAGTCCGAACAAAGAGCTGGCAAAAGAGTTCCTCGAAAACTATCTGCTGACTGATGAAGGTCTGGAAGCGGTTAAT
MBP-001             TCCAAACCGTTCGTTGGCGTGCTGAGCGCAGGTATTAACGCCGCCAGTCCGAACAAAGAGCTGGCAAAAGAGTTCCTCGAAAACTATCTGCTGACTGATGAAGGTCTGGAAGCGGTTAAT
MBP-002             TCCAAACCGTTCGTTGGCGTGCTGAGCGCAGGTATTAACGCCGCCAGTCCGAACAAAGAGCTGGCAAAAGAGTTCCTCGAAAACTATCTGCTGACTGATGAAGGTCTGGAAGCGGTTAAT
MBP-009             TCCAAACCGTTCGTTGGCGTGCTGAGCGCAGGTATTAACGCCGCCAGTCCGAACAAAGAGCTGGCAAAAGAGTTCCTCGAAAACTATCTGCTGACTGATGAAGGTCTGGAAGCGGTTAAT
MBP-003             TCCAAACCGTTCGTTGGCGTGCTGAGCGCAGGTATTAACGCCGCCAGTCCGAACAAAGAGCTGGCAAAAGAGTTCCTCGAAAACTATCTGCTGACTGATGAAGGTCTGGAAGCGGTTAAT
MBP-005             TCCAAACCGTTCGTTGGCGTGCTGAGCGCAGGTATTAACGCCGCCAGTCCGAACAAAGAGCTGGCGAAAGAGTTCCTCGAAAACTATCTGCTGACTGATGAAGGTCTGGAAGCGGTTAAT
MBP-006             TCCAAACCGTTCGTTGGCGTGCTGAGCGCAGGTATTAACGCCGCCAGTCCGAACAAAGAGCTGGCGAAAGAGTTCCTCGAAAACTATCTGCTGACTGATGAAGGTCTGGAAGCGGTTAAT
MBP-007             TCCAAACCGTTCGTTGGCGTGCTGAGCGCAGGTATTAACGCCGCCAGTCCGAACAAAGAGCTGGCAAAAGAGTTCCTCGAAAACTATCTGCTGACTGATGAAGGTCTGGAAGCGGTTAAT
MBP-008             TCCAAACCGTTCGTTGGCGTGCTGAGCGCAGGTATTAACGCCGCCAGTCCGAACAAAGAGCTGGCGAAAGAGTTCCTCGAAAACTATCTGCTGACTGATGAAGGTCTGGAAGCGGTTAAT
                    ***************************************************************** ******************************************************

MBP-004             AAAGACAAACCGCTGGGTGCCGTAGCGCTGAAGTCTTACGAGGAAGAGTTGGCGAAAGATCCACGTATTGCCGCCACCATGGAAAACGCCCAGAAAGGTGAAATCATGCCGAACATCCCG
MBP-010             AAAGACAAACCGCTGGGTGCCGTAGCGCTGAAGTCTTACGAGGAAGAGTTGGCGAAAGATCCACGTATTGCCGCCACCATGGAAAACGCCCAGAAAGGTGAAATCATGCCGAACATCCCG
MBP-001             AAAGACAAACCGCTGGGTGCCGTAGCGCTGAAGTCTTACGAGGAAGAGTTGGCGAAAGATCCACGTATTGCCGCCACTATGGAAAACGCCCAGAAAGGTGAAATCATGCCGAACATCCCG
MBP-002             AAAGACAAACCGCTGGGTGCCGTAGCGCTGAAGTCTTACGAGGAAGAGTTGGCGAAAGATCCACGTATTGCCGCCACTATGGAAAACGCCCAGAAAGGTGAAATCATGCCGAACATCCCG
MBP-009             AAAGACAAACCGCTGGGTGCCGTAGCGCTGAAGTCTTACGAGGAAGAGTTGGCGAAAGATCCACGTATTGCCGCCACTATGGAAAACGCCCAGAAAGGTGAAATCATGCCGAACATCCCG
MBP-003             AAAGACAAACCGCTGGGTGCCGTAGCGCTGAAGTCTTACGAGGAAGAGTTGGCGAAAGATCCACGTATTGCCGCCACTATGGAAAACGCCCAGAAAGGTGAAATCATGCCGAACATCCCG
MBP-005             AAAGACAAACCGCTGGGTGCCGTAGCGCTGAAGTCTTACGAGGAAGAGTTGGCGAAAGATCCACGTATTGCCGCCACCATGGAAAACGCCCAGAAAGGTGAAATCATGCCGAACATCCCG
MBP-006             AAAGACAAACCGCTGGGTGCCGTAGCGCTGAAGTCTTACGAGGAAGAGTTGGCGAAAGATCCACGTATTGCCGCCACCATGGAAAACGCCCAGAAAGGTGAAATCATGCCGAACATCCCG
MBP-007             AAAGACAAACCGCTGGGTGCCGTAGCGCTGAAGTCTTACGAGGAAGAGTTGGTGAAAGATCCGCGTATTGCCGCCACTATGGAAAACGCCCAGAAAGGTGAAATCATGCCGAACATCCCG
MBP-008             AAAGACAAACCGCTGGGTGCCGTAGCGCTGAAGTCTTACGAGGAAGAGTTGGCGAAAGATCCACGTATTGCCGCCACCATGGAAAACGCCCAGAAAGGTGAAATCATGCCGAACATCCCG
                    **************************************************** ********* ************** ******************************************

MBP-004             CAGATGTCCGCTTTCTGGTATGCCGTGCGTACTGCGGTGATCAACGCCGCCAGCGGTCGTCAGACTGTCGATGAAGCCCTGAAAGACGCGCAGACT
MBP-010             CAGATGTCCGCTTTCTGGTATGCCGTGCGTACTGCGGTGATCAACGCCGCCAGCGGTCGTCAGACTGTCGATGAAGCCCTGAAAGACGCGCAGACT
MBP-001             CAGATGTCCGCTTTCTGGTATGCCGTGCGTACTGCGGTGATCAACGCCGCCAGCGGTCGTCAGACTGTCGATGAAGCCCTGAAAGACGCGCAGACT
MBP-002             CAGATGTCCGCTTTCTGGTATGCCGTGCGTACTGCGGTGATCAACGCCGCCAGCGGTCGTCAGACTGTCGATGAAGCCCTGAAAGACGCGCAGACT
MBP-009             CAGATGTCCGCTTTCTGGTATGCCGTGCGTACTGCGGTGATCAACGCCGCCAGCGGTCGTCAGACTGTCGATGAAGCCCTGAAAGACGCGCAGACT
MBP-003             CAGATGTCCGCTTTCTGGTATGCCGTGCGTACTGCGGTGATCAACGCCGCCAGCGGTCGTCAGACTGTCGATGAAGCCCTGAAAGACGCGCAGACT
MBP-005             CAGATGTCCGCTTTCTGGTATGCCGTGCGTACTGCGGTGATCAACGCCGCCAGCGGTCGTCAGACTGTCGATGAAGCCCTGAAAGACGCGCAGACT
MBP-006             CAGATGTCCGCTTTCTGGTATGCCGTGCGTACTGCGGTGATCAACGCCGCCAGCGGTCGTCAGACTGTCGAT------------------------
MBP-007             CAGATGTCCGCTTTCTGGTATGCCGTGCGTACTGCGGTGATCAACGCCGCCAGCGGTCGTCAGACTGTCGATGAAGCCCTGAAAGACGCGCAGACT
MBP-008             CAGATGTCCGCTTTCTGGTATGCCGTGCGTACTGCGGTGATCAACGCCGCCAGCGGTCGTCAGACTGTCGAT------------------------
                    ************************************************************************
```

MBP-010 is identical to MBP-004 but missing START codon for C-terminal in-frame protein tagging
MBP-001 is identical to MBP-003 but missing START codon for C-terminal in-frame protein tagging

```
Key     ATGC = change in nucleotide relative to consensus sequence – synonymous
        ATGC = change in nucleotide relative to consensus sequence – conservative/semi-conservative amino acid substitution
        ATGC = change in nucleotide relative to consensus sequence – non-conservative amino acid substitution
```

Sequence Alignments

```
Name of             Alignment of Translated Protein                                                                                                                     Size    # Var
Variant                                                                                                                                                                 (aa)    aa
MBP-004             -------------------------MKIEEGKLVIWINGDKGYNGLAEVGKKFEKDTGIKVTVEHPDKLEEKFPQVAATGDGPDIIFWAHDRFGGYAQSGLLAEITPDKAFQDKLYPFTW                        367     0
MBP-010             --------------------------KIEEGKLVIWINGDKGYNGLAEVGKKFEKDTGIKVTVEHPDKLEEKFPQVAATGDGPDIIFWAHDRFGGYAQSGLLAEITPDKAFQDKLYPFTW                        366     0
MBP-001             --------------------------KIEEGKLVIWINGDKGYNGLAEVGKKFEKDTGIKVTVEHPDKLEEKFPQVAATGDGPDIIFWAHDRFGGYAQSGLLAEITPDKAFQDKLYPFTW                        366     0
MBP-002             ------------------------MGKIEEGKLVIWINGDKGYNGLAEVGKKFEKDTGIKVTVEHPDKLEEKFPQVAATGDGPDIIFWAHDRFGGYAQSGLLAEITPDKAFQDKLYPFTW                        368     0
MBP-009             -------------------------M-AEEGKLVIWINGDKGYNGLAEVGKKFEKDTGIKVTVEHPDKLEEKFPQVAATGDGPDIIFWAHDRFGGYAQSGLLAEITPDKAFQDKLYPFTW                        366     2
MBP-003             -------------------------MKIEEGKLVIWINGDKGYNGLAEVGKKFEKDTGIKVTVEHPDKLEEKFPQVAATGDGPDIIFWAHDRFGGYAQSGLLAEITPDKAFQDKLYPFTW                        367     0
MBP-005             --------------------------KTEEGKLVIWINGDKGYNGLAEVGKKFEKDTGIKVTVEHPDKLEEKFPQVAATGDGPDIIFWAHDRFGGYAQSGLLAEITPDKAFQDKLYPFTW                        366     1
MBP-006             --------------------------KIEEGKLVIWINGDKGYNGLAEVGKKFEKDTGIKVTVEHPDKLEEKFPQVAATGDGPDIIFWAHDRFGGYAQSGLLAEITPAAAFQDKLYPFTW                        358     5
MBP-007             MKIKTGARILALSALTTMMFSASALAKIEEGKLVIWINGDKGYNGLAEVGKKFEKDTGIKVTVEHPDKLEEKFPQVAATGDGPDIIFWAHDRFGGYAQSGLLAEITPDKAFQDKLYPFTW                        392     1
MBP-008             --------------------------KIEEGKLVIWINGDKGYNGLAEVGKKFEKDTGIKVTVEHPDKLEEKFPQVAATGDGPDIIFWAHDRFGGYAQSGLLAEITPDKAFQDKLYPFTW                        358     0
                                                ******************************************************************************  ***********

MBP-004             DAVRYNGKLIAYPIAVEALSLIYNKDLLPNPPKTWEEIPALDKELKAKGKSALMFNLQEPYFTWPLIAADGGYAFKYENGKYDIKDVGVDNAGAKAGLTFLVDLIKNKHMNADTDYSIAE
MBP-010             DAVRYNGKLIAYPIAVEALSLIYNKDLLPNPPKTWEEIPALDKELKAKGKSALMFNLQEPYFTWPLIAADGGYAFKYENGKYDIKDVGVDNAGAKAGLTFLVDLIKNKHMNADTDYSIAE
MBP-001             DAVRYNGKLIAYPIAVEALSLIYNKDLLPNPPKTWEEIPALDKELKAKGKSALMFNLQEPYFTWPLIAADGGYAFKYENGKYDIKDVGVDNAGAKAGLTFLVDLIKNKHMNADTDYSIAE
MBP-002             DAVRYNGKLIAYPIAVEALSLIYNKDLLPNPPKTWEEIPALDKELKAKGKSALMFNLQEPYFTWPLIAADGGYAFKYENGKYDIKDVGVDNAGAKAGLTFLVDLIKNKHMNADTDYSIAE
MBP-009             DAVRYNGKLIAYPIAVEALSLIYNKDLLPNPPKTWEEIPALDKELKAKGKSALMFNLQEPYFTWPLIAADGGYAFKYENGKYDIKDVGVDNAGAKAGLTFLVDLIKNKHMNADTDYSIAE
MBP-003             DAVRYNGKLIAYPIAVEALSLIYNKDLLPNPPKTWEEIPALDKELKAKGKSALMFNLQEPYFTWPLIAADGGYAFKYENGKYDIKDVGVDNAGAKAGLTFLVDLIKNKHMNADTDYSIAE
MBP-005             DAVRYNGKLIAYPIAVEALSLIYNKDLLPNPPKTWEEIPALDKELKAKGKSALMFNLQEPYFTWPLIAADGGYAFKYENGKYDIKDVGVDNAGAKAGLTFLVDLIKNKHMNADTDYSIAE
MBP-006             DAVRYNGKLIAYPIAVEALSLIYNKDLLPNPPKTWEEIPALDKELKAKGKSALMFNLQEPYFTWPLIAADGGYAFKYAAGKYDIKDVGVDNAGAKAGLTFLVDLIKNKHMNADTDYSIAE
MBP-007             DAVRYNGKLIAYPIAVEALSLIYNKDLLPNPPKTWEEIPALDKELKAKGKSALMFNLQEPYFTWPLIAADGGYAFKYENGKYDIKDVGVDNAGAKAGLTFLVDLIKNKHMNADTDYSIAE
MBP-008             DAVRYNGKLIAYPIAVEALSLIYNKDLLPNPPKTWEEIPALDKELKAKGKSALMFNLQEPYFTWPLIAADGGYAFKYENGKYDIKDVGVDNAGAKAGLTFLVDLIKNKHMNADTDYSIAE
                    *****************************************************************************  *****************************************

MBP-004             AAFNKGETAMTINGPWAWSNIDTSKVNYGVTVLPTFKGQP...LEAVNKDKPLGAVALKSYEEELAKDPRIAATMENAQKGEIMPNIPQMSAFWYAVRTAVINAASGRQTVDEALKDAQT
MBP-010             AAFNKGETAMTINGPWAWSNIDTSKVNYGVTVLPTFKGQP...LEAVNKDKPLGAVALKSYEEELAKDPRIAATMENAQKGEIMPNIPQMSAFWYAVRTAVINAASGRQTVDEALKDAQT
MBP-001             AAFNKGETAMTINGPWAWSNIDTSKVNYGVTVLPTFKGQP...LEAVNKDKPLGAVALKSYEEELAKDPRIAATMENAQKGEIMPNIPQMSAFWYAVRTAVINAASGRQTVDEALKDAQT
MBP-002             AAFNKGETAMTINGPWAWSNIDTSKVNYGVTVLPTFKGQP...LEAVNKDKPLGAVALKSYEEELAKDPRIAATMENAQKGEIMPNIPQMSAFWYAVRTAVINAASGRQTVDEALKDAQT
MBP-009             AAFNKGETAMTINGPWAWSNIDTSKVNYGVTVLPTFKGQP...LEAVNKDKPLGAVALKSYEEELAKDPRIAATMENAQKGEIMPNIPQMSAFWYAVRTAVINAASGRQTVDEALKDAQT
MBP-003             AAFNKGETAMTINGPWAWSNIDTSKVNYGVTVLPTFKGQP...LEAVNKDKPLGAVALKSYEEELAKDPRIAATMENAQKGEIMPNIPQMSAFWYAVRTAVINAASGRQTVDEALKDAQT
MBP-005             AAFNKGETAMTINGPWAWSNIDTSKVNYGVTVLPTFKGQP...LEAVNKDKPLGAVALKSYEEELAKDPRIAATMENAQKGEIMPNIPQMSAFWYAVRTAVINAASGRQTVDEALKDAQT
MBP-006             AAFNKGETAMTINGPWAWSNIDTSAVNYGVTVLPTFKGQP...LEAVNKDKPLGAVALKSYEEELAKDPRIAATMENAQKGEIMPNIPQMSAFWYAVRTAVINAASGRQTVD--------
MBP-007             AAFNKGETAMTINGPWAWSNIDTSKVNYGVTVLPTFKGQP...LEAVNKDKPLGAVALKSYEEELVKDPRIAATMENAQKGEIMPNIPQMSAFWYAVRTAVINAASGRQTVDEALKDAQT
MBP-008             AAFNKGETAMTINGPWAWSNIDTSKVNYGVTVLPTFKGQP...LEAVNKDKPLGAVALKSYEEELAKDPRIAATMENAQKGEIMPNIPQMSAFWYAVRTAVINAASGRQTVD--------
                    ************************ ***************...**********************.**********************************************
```

Key
ATGC = change in nucleotide relative to consensus sequence – synonymous
ATGC = change in nucleotide relative to consensus sequence – conservative/semi-conservative amino acid substitution
ATGC = change in nucleotide relative to consensus sequence – non-conservative amino acid substitution

Sequence Alignments

Name of Variant	# of Occur	Alignment	Size (bp)	# Var bp	Sources*
GST-002	44	ATG---TCCCCTATACTAGGTTATTGGAAAATTAAGGGCCTTGTGCAACCCACTCGACTT.GCATGGCCTTTGCAGGGCTGGCAAGCCACGTTTGGTGGTGGCGACCATCCTCCAAAA---	654	0	GE,No,BD
GST-004	3	ATGGCATCCCCTATACTAGGTTATTGGAAAATTAAGGGCCTTGTGCAACCCACTCGACTT.GCATGGCCTTTGCAGGGCTGGCAAGCCACGTTTGGTGGTGGCGACCATCCTCCAAAA---	657	0/3i	No
GST-005	2	------TCCCCTATACTAGGTTATTGGAAAATTAAGGGCCTTGTGCAACCCACTCGACTT.GCATGGCCTTTGCAGGGCTGGCAAGCCACGTTTGGTGGTGGCGACCATCCTCCAAAA---	651	0/3d	19906724
GST-001	2	ATG---GCCCCTATACTAGGTTATTGGAAAATTAAGGGCCTTGTGCAACCCACTCGACTT.GCATGGCCTTTGCAGGGCTGGCAAGCCACGTTTGGTGGTGGCGACCATCCTCCAAAA---	654	1	No
GST-003	1	ATG---TCCCCTATACTAGGTTATTGGAAAATTAAGGGCCTTGTGCAACCCACTCGACTT.GCATGGCCTTTGCAGGGCTGGCAAGCCACGTTTGGTGGTGGCGACCATCCTCCAAAATAA	657	0/3i	#
GST-006	1	ATG---AGCCCTATACTAGGTTATTGGAAAATTAAGGGCCTTGTGCAACCCACTCGACTT.GCATGGCCTTTGCAGGGCTGGCAAGCCACGTTTGGTGGTGGCGACCATCCTCCAAAA---	654	1	AB
GST-007	1	---------CCTATACTAGGTTATTGGAAAATTAAGGGCCTTGTGCAACCCACTCGACTT.GCATGGCCTTTGCAGGGCTGGCAAGCCACGTTTGGTGGTGGCGACCATCCTCCAAAA---	648	6	15232106
GST-008	1	ATGGAATCCCCTATACTAGGTTATTGGAAAATTAAGGGCCTTGTGCAACCCACTCGACTT.GCATGGCCTTTGCAGGGCTGGCAAGCCACGTTTGGTGGTGGCGACCATCCTCCAAAA---	657	3	15782178
		*** ** ***			

Name of Variant	Alignment of Translated Protein	Size (aa)	Tag Pos	# Var aa
GST-002	-MSPILGYWKIKGLVQPTRLLLEYLEEKYEEHLYERDEGDKWRNKKFELGLEFPNLPYYI.KKRIEAIPQIDKYLKSSKYIAWPLQGWQATFGGGDHPPK	218	N/C[1]	0
GST-004	MASPILGYWKIKGLVQPTRLLLEYLEEKYEEHLYERDEGDKWRNKKFELGLEFPNLPYYI.KKRIEAIPQIDKYLKSSKYIAWPLQGWQATFGGGDHPPK	219	N	1i
GST-005	--SPILGYWKIKGLVQPTRLLLEYLEEKYEEHLYERDEGDKWRNKKFELGLEFPNLPYYI.KKRIEAIPQIDKYLKSSKYIAWPLQGWQATFGGGDHPPK	217	N[2]	1d
GST-001	-MAPILGYWKIKGLVQPTRLLLEYLEEKYEEHLYERDEGDKWRNKKFELGLEFPNLPYYI.KKRIEAIPQIDKYLKSSKYIAWPLQGWQATFGGGDHPPK	218	N	1
GST-003	-MSPILGYWKIKGLVQPTRLLLEYLEEKYEEHLYERDEGDKWRNKKFELGLEFPNLPYYI.KKRIEAIPQIDKYLKSSKYIAWPLQGWQATFGGGDHPPK	218	#	0
GST-006	-MSPILGYWKIKGLVQPTRLLLEYLEEKYEEHLYERDEGDKWRNKKFELGLEFPNLPYYI.KKRIEAIPQIDKYLKSSKYIAWPLQGWQATFGGGDHPPK	218	N	0
GST-007	---PILGYWKIKGLVQPTRLLLEYLEEKYEEHLYERDEGDKWRNKKFELGLEFPNLPYYI.KKRIEAIPQIDKYLKSSKYIAWPLQGWQATFGGGDHPPK	216	C	2d
GST-008	MESPILGYWKIKGLVQPTRLLLEYLEEKYEEHLYERDEGDKWRNKKFELGLEFPNLPYYI.KKRIEAIPQIDKYLKSSKYIAWPLQGWQATFGGGDHPPK	219	N	1i
	*** ***************************************			

from feature file. Not found in any vector in the plasmid library.
1 followed by STOP codon after additional in-frame residues or followed by another tag.
2 preceded either by another tag or a START codon and additional in-frame residues.

GST-005 is identical to GST-002 but missing START codon for C-terminal in-frame protein tagging

Name of Variant	#of Occur	Alignment	Size (bp)	# Var bp	Express Host	CAIHost/Non-Host[1]	Sources*
HA-008	10	TACCCATACGATGTTCCAGATTACGCT	27	5	Mammal-Plant	0.783/0.508	Or,Cl
HA-007	5	TATCCGTACGACGTACCAGACTACGCA	27	3	Mammal	0.463/0.510	Cl
HA-003	4	TATCCGTATGATGTGCCGGACTACGCA	27	5	E. coli	0.424/0.571	MCSG
HA-009	3	TATCCTTATGACGTGCCTGACTATGCC	27	5	Mammal-S. cer.	0.941-0.071/0.155	Cl
HA-002	2	TATCCGTACGACGTACCGGACTACGCA	27	4	E. coli	0.642/0.395	MCSG
HA-004	2	TACCCATACGATGTTCCGGATTACGCT	27	6	Mammal	0.668/0.640	Mo
HA-006	2	TACCCATACGACGTACCAGATTACGCT	27	4	S. cer.	0.182/0.478	Cl
HA-001	1	TATCCGTATGACGTGCCCGACTATGCC	27	4	Mammal	0.784/0.140	Lu
HA-005	1	TACCCTTATGATGTGCCAGATTATGCC	27	5	S. cer.	0.108/0.153	8242750
HA-010	1	TACCCCTACGACGTGCCCGACTACGCC	27	1	Mammal	1.000/0.096	15232106
		** ** ** ** ** ** ** ** **					

1 Non-Host is *E.coli* for Mammal, Plant and *S. cerevisiae*. Non-Host is Mammal (Mouse) for *E. coli*. Carbone et al. (2003) reference set and Eyre-Walker (1996) equation.

Key ATGC = change in nucleotide relative to consensus sequence – synonymous
ATGC = change in nucleotide relative to consensus sequence – conservative/semi-conservative amino acid substitution
ATGC = change in nucleotide relative to consensus sequence – non-conservative amino acid substitution
