# Supplementary material for: GenoLIB: a database of biological parts derived from a library of common plasmid features
Source: Nucleic Acids Res. 2015 Apr 29;43(10):4823–32. doi: 10.1093/nar/gkv272 (PMC4446419; doi:10.1093/nar/gkv272)
Supplement: SUPPLEMENTARY DATA [file supp_gkv272_nar-03388-h-2014-File007.pdf]

Suggested Minimal Sequences for Highly Variant Features

These suggested sequences are identified as recommended in the GenoCAD parts library.

>AmpR_promoter (variant -004) 72 bp
CATTCAAATATGTATCCGCTCATGAGACAATAACCCTGATAAATGCTTCAATAATATTG
AAAAAGGAAGAGT

>cat_promoter (variant -004) 103 bp
TGATCGGCACGTAAGAGGTTCCAACTTTCACCATAATGAAATAAGATCACTACCGGGCG
TATTTTTTGAGTTATCGAGATTTTCAGGAGCTAAGGAAGCTAAA

>NeoR/KanR_promoter (consensus seq) 114 bp
TCTGGTAAGGTTGGGAAGCCCTGCAAAGTAAACTGGATGGCTTTCTTGCCGCCAAGGAT
CTGATGGCGCAGGGGATCAAGATCTGATCAAGAGACAGGATGAGGATCGTTTCGC

>KanR-aph(3')-Ia_promoter (consensus seq) 99 bp
GTGTCTCAAAATCTCTGATGTTACATTGCACAAGATAAAAATATATCATCATGAACAAT
AAAACTGTCTGCTTACATAAACAGTAATACAAGGGGTGTT

>KanR-aphA-3_promoter (consensus seq) 182 bp
GGTTTCAAAATCGGCTCCGTCGATACTATGTTATACGCCAACTTTGAAAACAACTTTGA
AAAAGCTGTTTTCTGGTATTTAAGGTTTTAGAATGCAAGGAACAGTGAATTGGAGTTCG
TCTTGTTATAATTAGCTTCTTGGGGTATCTTTAAATACTGTAGAAAAGAGGAAGGAAAT
AATAA

>SmR-PcS-P2S_promoter (consensus seq) 231 bp
ACCCAGTTGACATAAGCCTGTTCGGTTCGTAAACTGTAATGCAAGTAGCGTAACTGCCG
TCACGCAACTGGTCCAGAACCTTGACCGAACGCAGCGGTGGTAACGGCGCAGTGGCGGT
TTTCATGGCTTCTTGTTATGACTGTTTTTTTGGGGTACAGTCTATGCCTCGGGCATCCA
AGCAGCAAGCGCGTTACGCCGTGGGTCGATGTTTGATGTTATGGAGCAGCAACG

>SmR-PcW-P2S_promoter (consensus seq) 232 bp
ACCCAGTGGACATAAGCCTCGTTCGGTTCGTAAGCTGTAATGCAAGTAGCGTAACTGCC
GTCACGCAACTGGTCCAGAACCTTGACCGAACGCAGCGGTGGTAACGGCGCAGTGGCGG
TTTTCATGGCTTCTTGTTATGACTGTTTTTTTGGGGTACAGTCTATGCCTCGGGCATCC
AAGCAGCAAGCGCGTTACGCCGTGGGTCGATGTTTGATGTTATGGAGCAGCAACG

>SmR-PcW-P2W_promoter (consensus seq) 233 bp
ACCCAGTGGACATAAGCCTCGTTCGGTTCGTAAGCTGTAATGCAAGTAGCGTAACTGCC
GTCACGCAACTGGTCCAGAACCTTGACCGAACGCAGCGGTGGTAACGGCGCAGTGGCGG
TTTTCATGGCTTCTTGTTATGACATGTTTTTTTGGGGTACAGTCTATGCCTCGGGCATC
CAAGCAGCAAGCGCGTTACGCCGTGGGTCGATGTTTGATGTTATGGAGCAGCAACG

>ccdB_promoter (consensus seq) 107 bp
GGCTTACTAAAAGCCAGATAACAGTATGCGTATTTGCGCGCTGATTTTTGCGGTATAAG
AATATATACTGATATGTATACCCGAAGTATGTCAAAAAGAGGTATGCT

```
>CMV_enhancer (variant -003) 304 bp
CGTTACATAACTTACGGTAAATGGCCCGCCTGGCTGACCGCCCAACGACCCCCGCCCAT
TGACGTCAATAATGACGTATGTTCCCATAGTAACGCCAATAGGGACTTTCCATTGACGT
CAATGGGTGGAGTATTTACGGTAAACTGCCCACTTGGCAGTACATCAAGTGTATCATAT
GCCAAGTACGCCCCCTATTGACGTCAATGACGGTAAATGGCCCGCCTGGCATTATGCCC
AGTACATGACCTTATGGGACTTTCCTACTTGGCAGTACATCTACGTATTAGTCATCGCT
ATTACCATG

>CMV_promoter (variant -008) 199 bp
TGATGCGGTTTTGGCAGTACATCAATGGGCGTGGATAGCGGTTTGACTCACGGGGATTT
CCAAGTCTCCACCCCATTGACGTCAATGGGAGTTTGTTTTGGCACCAAAATCAACGGGA
CTTTCCAAAATGTCGTAACAACTCCGCCCCATTGACGCAAATGGGCGGTAGGCGTGTAC
GGTGGGAGGTCTATATAAGCAG

>SV40_ori (variant -004) 136 bp
ATCCCGCCCCTAACTCCGCCCAGTTCCGCCCATTCTCCGCCCCATGGCTGACTAATTTT
TTTTATTTATGCAGAGGCCGAGGCCGCCTCGGCCTCTGAGCTATTCCAGAAGTAGTGAG
GAGGCTTTTTTGGAGGCC

>SV40_promoter (variant -008) 196 bp
TGCATCTCAATTAGTCAGCAACCATAGTCCCGCCCCTAACTCCGCCCATCCCGCCCCTA
ACTCCGCCCAGTTCCGCCCATTCTCCGCCCCATGGCTGACTAATTTTTTTTATTTATGC
AGAGGCCGAGGCCGCCTCGGCCTCTGAGCTATTCCAGAAGTAGTGAGGAGGCTTTTTTG
GAGGCCTAGGCTTTTGCAA

>SV40_enhancer-promoter (promoter variant -002) 303 bp
GGTGTGGAAAGTCCCCAGGCTCCCCAGCAGGCAGAAGTATGCAAAGCATGCATCTCAAT
TAGTCAGCAACCAGGTGTGGAAAGTCCCCAGGCTCCCCAGCAGGCAGAAGTATGCAAAG
CATGCATCTCAATTAGTCAGCAACCATAGTCCCGCCCCTAACTCCGCCCATCCCGCCCC
TAACTCCGCCCAGTTCCGCCCATTCTCCGCCCCATGGCTGACTAATTTTTTTTATTTAT
GCAGAGGCCGAGGCCGCCTCGGCCTCTGAGCTATTCCAGAAGTAGTGAGGAGGCTTTTT
TGGAGGCC

>f1_ori_minimal (consensus seq) 381 bp
ACGCGCCCTGTAGCGGCGCATTAAGCGCGGCGGGTGTGGTGGTTACGCGCAGCGTGACC
GCTACACTTGCCAGCGCCCTAGCGCCCGCTCCTTTCGCTTTCTTCCCTTCCTTTCTCGC
CACGTTCGCCGGCTTTCCCCGTCAAGCTCTAAATCGGGGGCTCCCTTTAGGGTTCCGAT
TTAGTGCTTTACGGCACCTCGACCCCAAAAAACTTGATTAGGGTGATGGTTCACGTAGT
GGGCCATCGCCCTGATAGACGGTTTTTCGCCCTTTGACGTTGGAGTCCACGTTCTTTAA
TAGTGGACTCTTGTTCCAAACTGGAACAACACTCAACCCTATCTCGGTCTATTCTTTTG
ATTTATAAGGGATTTTGCCGATTTCGG

>f1_ori_plus-strand enhancer (consensus seq) 456 bp
ACGCGCCCTGTAGCGGCGCATTAAGCGCGGCGGGTGTGGTGGTTACGCGCAGCGTGACC
GCTACACTTGCCAGCGCCCTAGCGCCCGCTCCTTTCGCTTTCTTCCCTTCCTTTCTCGC
CACGTTCGCCGGCTTTCCCCGTCAAGCTCTAAATCGGGGGCTCCCTTTAGGGTTCCGAT
TTAGTGCTTTACGGCACCTCGACCCCAAAAAACTTGATTAGGGTGATGGTTCACGTAGT
```

GGGCCATCGCCCTGATAGACGGTTTTTCGCCCTTTGACGTTGGAGTCCACGTTCTTTAA
TAGTGGACTCTTGTTCCAAACTGGAACAACACTCAACCCTATCTCGGTCTATTCTTTTG
ATTTATAAGGGATTTTGCCGATTTCGGCCTATTGGTTAAAAAATGAGCTGATTTAACAA
AAATTTAACGCGAATTTTAACAAAATATTAACGTTTACAATTT

>pMB1-ColE1_ori-high_copy (variant -014) 589 bp
TTGAGATCCTTTTTTTCTGCGCGTAATCTGCTGCTTGCAAACAAAAAAACCACCGCTAC
CAGCGGTGGTTTGTTTGCCGGATCAAGAGCTACCAACTCTTTTTCCGAAGGTAACTGGC
TTCAGCAGAGCGCAGATACCAAATACTGTCCTTCTAGTGTAGCCGTAGTTAGGCCACCA
CTTCAAGAACTCTGTAGCACCGCCTACATACCTCGCTCTGCTAATCCTGTTACCAGTGG
CTGCTGCCAGTGGCGATAAGTCGTGTCTTACCGGGTTGGACTCAAGACGATAGTTACCG
GATAAGGCGCAGCGGTCGGGCTGAACGGGGGGTTCGTGCACACAGCCCAGCTTGGAGCG
AACGACCTACACCGAACTGAGATACCTACAGCGTGAGCTATGAGAAAGCGCCACGCTTC
CCGAAGGGAGAAAGGCGGACAGGTATCCGGTAAGCGGCAGGGTCGGAACAGGAGAGCGC
ACGAGGGAGCTTCCAGGGGGAAACGCCTGGTATCTTTATAGTCCTGTCGGGTTTCGCCA
CCTCTGACTTGAGCGTCGATTTTTGTGATGCTCGTCAGGGGGGCGGAGCCTATGGAAA

>pMB1-ColE1_ori-medium copy (variant -014) 589 bp
TTGAGATCCTTTTTTTCTGCGCGTAATCTGCTGCTTGCAAACAAAAAAACCACCGCTAC
CAGCGGTGGTTTGTTTGCCGGATCAAGAGCTACCAACTCTTTTTCCGAAGGTAACTGGC
TTCAGCAGAGCGCAGATACCAAATACTGTTCTTCTAGTGTAGCCGTAGTTAGGCCACCA
CTTCAAGAACTCTGTAGCACCGCCTACATACCTCGCTCTGCTAATCCTGTTACCAGTGG
CTGCTGCCAGTGGCGATAAGTCGTGTCTTACCGGGTTGGACTCAAGACGATAGTTACCG
GATAAGGCGCAGCGGTCGGGCTGAACGGGGGGTTCGTGCACACAGCCCAGCTTGGAGCG
AACGACCTACACCGAACTGAGATACCTACAGCGTGAGCTATGAGAAAGCGCCACGCTTC
CCGAAGGGAGAAAGGCGGACAGGTATCCGGTAAGCGGCAGGGTCGGAACAGGAGAGCGC
ACGAGGGAGCTTCCAGGGGGAAACGCCTGGTATCTTTATAGTCCTGTCGGGTTTCGCCA
CCTCTGACTTGAGCGTCGATTTTTGTGATGCTCGTCAGGGGGGCGGAGCCTATGGAAA

>EMCV-IRES_high_express (consensus seq) 589 bp
CCCCTCTCCCTCCCCCCCCCCTAACGTTACTGGCCGAAGCCGCTTGGAATAAGGCCGGT
GTGCGTTTGTCTATATGTTATTTTCCACCATATTGCCGTCTTTTGGCAATGTGAGGGCC
CGGAAACCTGGCCCTGTCTTCTTGACGAGCATTCCTAGGGGTCTTTCCCCTCTCGCCAA
AGGAATGCAAGGTCTGTTGAATGTCGTGAAGGAAGCAGTTCCTCTGGAAGCTTCTTGAA
GACAAACAACGTCTGTAGCGACCCTTTGCAGGCAGCGGAACCCCCCACCTGGCGACAGG
TGCCTCTGCGGCCAAAAGCCACGTGTATAAGATACACCTGCAAAGGCGGCACAACCCCA
GTGCCACGTTGTGAGTTGGATAGTTGTGGAAAGAGTCAAATGGCTCTCCTCAAGCGTAT
TCAACAAGGGGCTGAAGGATGCCCAGAAGGTACCCCATTGTATGGGATCTGATCTGGGG
CCTCGGTGCACATGCTTTACATGTGTTTAGTCGAGGTTAAAAAACGTCTAGGCCCCCCG
AACCACGGGGACGTGGTTTTCCTTTGAAAAACACGATGATAATATGGCCACAACCATG

>EMCV-IRES_attenuated (consensus seq) 578 bp
CCCCTCTCCCTCCCCCCCCCCTAACGTTACTGGCCGAAGCCGCTTGGAATAAGGCCGGT
GTGCGTTTGTCTATATGTTATTTTCCACCATATTGCCGTCTTTTGGCAATGTGAGGGCC
CGGAAACCTGGCCCTGTCTTCTTGACGAGCATTCCTAGGGGTCTTTCCCCTCTCGCCAA
AGGAATGCAAGGTCTGTTGAATGTCGTGAAGGAAGCAGTTCCTCTGGAAGCTTCTTGAA
GACAAACAACGTCTGTAGCGACCCTTTGCAGGCAGCGGAACCCCCCACCTGGCGACAGG
TGCCTCTGCGGCCAAAAGCCACGTGTATAAGATACACCTGCAAAGGCGGCACAACCCCA

GTGCCACGTTGTGAGTTGGATAGTTGTGGAAAGAGTCAAATGGCTCTCCTCAAGCGTAT
TCAACAAGGGGCTGAAGGATGCCCAGAAGGTACCCCATTGTATGGGATCTGATCTGGGG
CCTCGGTGCACATGCTTTACATGTGTTTAGTCGAGGTTAAAAAAACGTCTAGGCCCCCC
GAACCACGGGGACGTGGTTTTCCTTTGAAAAACACGATGATAATATG

>EMCV-IRES_minimal_attenuated (consensus seq) 467 bp
GAGGGCCCGGAAACCTGGCCCTGTCTTCTTGACGAGCATTCCTAGGGGTCTTTCCCCTC
TCGCCAAAGGAATGCAAGGTCTGTTGAATGTCGTGAAGGAAGCAGTTCCTCTGGAAGCT
TCTTGAAGACAAACAACGTCTGTAGCGACCCTTTGCAGGCAGCGGAACCCCCCACCTGG
CGACAGGTGCCTCTGCGGCCAAAAGCCACGTGTATAAGATACACCTGCAAAGGCGGCAC
AACCCCAGTGCCACGTTGTGAGTTGGATAGTTGTGGAAAGAGTCAAATGGCTCTCCTCA
AGCGTATTCAACAAGGGGCTGAAGGATGCCCAGAAGGTACCCCATTGTATGGGATCTGA
TCTGGGGCCTCGGTGCACATGCTTTACATGTGTTTAGTCGAGGTTAAAAAAACGTCTAG
GCCCCCCGAACCACGGGGACGTGGTTTTCCTTTGAAAAACACGATGATAATATG

>AmpR-bla (*E. coli*; variant -013) 861 bp
ATGAGTATTCAACATTTCCGTGTCGCCCTTATTCCCTTTTTTGCGGCATTTTGCCTTCC
TGTTTTTGCTCACCCAGAAACGCTGGTGAAAGTAAAAGATGCTGAAGATCAGTTGGGTG
CACGAGTGGGTTACATCGAACTGGATCTCAACAGCGGTAAGATCCTTGAGAGTTTTCGC
CCCGAAGAACGTTTTCCAATGATGAGCACTTTTAAAGTTCTGCTATGTGGCGCGGTATT
ATCCCGTATTGACGCCGGGCAAGAGCAACTCGGTCGCCGCATACACTATTCTCAGAATG
ACTTGGTTGAGTACTCACCAGTCACAGAAAAGCATCTTACGGATGGCATGACAGTAAGA
GAATTATGCAGTGCTGCCATAACCATGAGTGATAACACTGCGGCCAACTTACTTCTGAC
AACGATCGGAGGACCGAAGGAGCTAACCGCTTTTTTGCACAACATGGGGGATCATGTAA
CTCGCCTTGATCGTTGGGAACCGGAGCTGAATGAAGCCATACCAAACGACGAGCGTGAC
ACCACGATGCCTGTAGCAATGGCAACAACGTTGCGCAAACTATTAACTGGCGAACTACT
TACTCTAGCTTCCCGGCAACAATTAATAGACTGGATGGAGGCGGATAAAGTTGCAGGAC
CACTTCTGCGCTCGGCCCTTCCGGCTGGCTGGTTTATTGCTGATAAATCTGGAGCCGGT
GAGCGTGGGTCTCGCGGTATCATTGCAGCACTGGGGCCAGATGGTAAGCCCTCCCGTAT
CGTAGTTATCTACACGACGGGGAGTCAGGCAACTATGGATGAACGAAATAGACAGATCG
CTGAGATAGGTGCCTCACTGATTAAGCATTGGTAA

>AmpR-bla(M) (variant -002) 795 bp
ATGGACCCAGAAACGCTGGTGAAAGTAAAAGATGCTGAAGATCAGTTGGGTGCACGAGT
GGGTTACATCGAACTGGATCTCAACAGCGGTAAGATCCTTGAGAGTTTTCGCCCCGAAG
AACGTTTTCCAATGATGAGCACTTTTAAAGTTCTGCTATGTGGCGCGGTATTATCCCGT
ATTGACGCCGGGCAAGAGCAACTCGGTCGCCGCATACACTATTCTCAGAATGACTTGGT
TGAGTACTCACCAGTCACAGAAAAGCATCTTACGGATGGCATGACAGTAAGAGAATTAT
GCAGTGCTGCCATAACCATGAGTGATAACACTGCGGCCAACTTACTTCTGACAACGATC
GGAGGACCGAAGGAGCTAACCGCTTTTTTGCACAACATGGGGGATCATGTAACTCGCCT
TGATCGTTGGGAACCGGAGCTGAATGAAGCCATACCAAACGACGAGCGTGACACCACGA
TGCCTGTAGCAATGGCAACAACGTTGCGCAAACTATTAACTGGCGAACTACTTACTCTA
GCTTCCCGGCAACAATTAATAGACTGGATGGAGGCGGATAAAGTTGCAGGACCACTTCT
GCGCTCGGCCCTTCCGGCTGGCTGGTTTATTGCTGATAAATCTGGAGCCGGTGAGCGTG
GGTCTCGCGGTATCATTGCAGCACTGGGGCCAGATGGTAAGCCCTCCCGTATCGTAGTT
ATCTACACGACGGGGAGTCAGGCAACTATGGATGAACGAAATAGACAGATCGCTGAGAT
AGGTGCCTCACTGATTAAGCATTGGTAA

>BleoR-ble (*Streptoalloteichus*; variant -003) 375 bp
ATGGCCAAGTTGACCAGTGCCGTTCCGGTGCTCACCGCGCGCGACGTCGCCGGAGCGGT
CGAGTTCTGGACCGACCGGCTCGGGTTCTCCCGGGACTTCGTGGAGGACGACTTCGCCG
GTGTGGTCCGGGACGACGTGACCCTGTTCATCAGCGCGGTCCAGGACCAGGTGGTGCCG
GACAACACCCTGGCCTGGGTGTGGGTGCGCGGCCTGGACGAGCTGTACGCCGAGTGGTC
GGAGGTCGTGTCCACGAACTTCCGGGACGCCTCCGGGCCGGCCATGACCGAGATCGGCG
AGCAGCCGTGGGGGCGGGAGTTCGCCCTGCGCGACCCGGCCGGCAACTGCGTGCACTTC
GTGGCCGAGGAGCAGGACTGA

>BlpR-bar (*Streptomyces*; variant -002) 552 bp
ATGAGCCCAGAACGACGCCCGGCCGACATCCGCCGTGCCACCGAGGCGGACATGCCGGC
GGTCTGCACCATCGTCAACCACTACATCGAGACAAGCACGGTCAACTTCCGTACCGAGC
CGCAGGAACCGCAGGAGTGGACGGACGACCTCGTCCGTCTGCGGGAGCGCTATCCCTGG
CTCGTCGCCGAGGTGGACGGCGAGGTCGCCGGCATCGCCTACGCGGGCCCCTGGAAGGC
ACGCAACGCCTACGACTGGACGGCCGAGTCGACCGTGTACGTCTCCCCCCGCCACCAGC
GGACGGGACTGGGCTCCACGCTCTACACCCACCTGCTGAAGTCCCTGGAGGCACAGGGC
TTCAAGAGCGTGGTCGCTGTCATCGGGCTGCCCAACGACCCGAGCGTGCGCATGCACGA
GGCGCTCGGATATGCCCCCCGCGGCATGCTGCGGGCGGCCGGCTTCAAGCACGGGAACT
GGCATGACGTGGGTTTCTGGCAGCTGGACTTCAGCCTGCCGGTACCGCCCCGTCCGGTC
CTGCCCGTCACCGAGATTTGA

>BlpR-pat1 (SnapGene feature) 456 bp
ATGGCGGCGGTCTGCGACATCGTCAATCACTACATCGAGACGAGCACGGTCAACTTCCG
TACGGAGCCGCAGACTCCGCAGGAGTGGATCGACGACCTGGAGCGCCTCCAGGACCGCT
ACCCCTGGCTCGTCGCCGAGGTGGAGGGCGTCGTCGCCGGCATCGCCTACGCCGGCCCC
TGGAAGGCCCGCAACGCCTACGACTGGACCGTCGAGTCGACGGTGTACGTCTCCCACCG
GCACCAGCGGCTCGGACTGGGCTCCACCCTCTACACCCACCTGCTGAAGTCCATGGAGA
CCCAGGGCTTCAAGAGCGTGGTCGCCGTCATCGGACTGCCCAACGACCCGAGCGTGCGC
CTGCACGAGGCGCTCGGATACACCGCGCGCGGGACGCTGCGGGCAGCCGGCTACAAGCA
CGGGGGCTGGCACGACGTGGGGTTCTGGCAGCGCGACTTCTAG

>BSD (*Aspergillus*; variant -005) 396 bp
ATGGCCAAGCCTTTGTCTCAAGAAGAATCCACCCTCATTGAAAGAGCAACGGCTACAAT
CAACAGCATCCCCATCTCTGAAGACTACAGCGTCGCCAGCGCAGCTCTCTCTAGCGACG
GCCGCATCTTCACTGGTGTCAATGTATATCATTTTACTGGGGGACCTTGTGCAGAACTC
GTGGTGCTGGGCACTGCTGCTGCTGCGGCAGCTGGCAACCTGACTTGTATCGTCGCGAT
CGGAAATGAGAACAGGGGCATCTTGAGCCCCTGCGGACGGTGCCGACAGGTGCTTCTCG
ATCTGCATCCTGGGATCAAAGCCATAGTGAAGGACAGTGATGGACAGCCGACGGCAGTT
GGGATTCGTGAATTGCTGCCCTCTGGTTATGTGTGGGAGGGCTAA

>ccdB (F plasmid; variant -004) 306 bp
ATGCAGTTTAAGGTTTACACCTATAAAAGAGAGAGCCGTTATCGTCTGTTTGTGGATGT
ACAGAGTGATATTATTGACACGCCCGGGCGACGGATGGTGATCCCCCTGGCCAGTGCAC
GTCTGCTGTCAGATAAAGTCTCCCGTGAACTTTACCCGGTGGTGCATATCGGGGATGAA
AGCTGGCGCATGATGACCACCGATATGGCCAGTGTGCCGGTCTCCGTTATCGGGGAAGA

AGTGGCTGATCTCAGCCACCGCGAAAATGACATCAAAAACGCCATTAACCTGATGTTCT
GGGGAATATAA

>CmR-cat (*E. coli*; variant -013) 660 bp
ATGGAGAAAAAAATCACTGGATATACCACCGTTGATATATCCCAATGGCATCGTAAAGA
ACATTTTGAGGCATTTCAGTCAGTTGCTCAATGTACCTATAACCAGACCGTTCAGCTGG
ATATTACGGCCTTTTTAAAGACCGTAAAGAAAAATAAGCACAAGTTTTATCCGGCCTTT
ATTCACATTCTTGCCCGCCTGATGAATGCTCATCCGGAATTCCGTATGGCAATGAAAGA
CGGTGAGCTGGTGATATGGGATAGTGTTCACCCTTGTTACACCGTTTTCCATGAGCAAA
CTGAAACGTTTTCATCGCTCTGGAGTGAATACCACGACGATTTCCGGCAGTTTCTACAC
ATATATTCGCAAGATGTGGCGTGTTACGGTGAAAACCTGGCCTATTTCCCTAAAGGGTT
TATTGAGAATATGTTTTTCGTCTCAGCCAATCCCTGGGTGAGTTTCACCAGTTTTGATT
TAAACGTGGCCAATATGGACAACTTCTTCGCCCCCGTTTTCACCATGGGCAAATATTAT
ACGCAAGGCGACAAGGTGCTGATGCCGCTGGCGATTCAGGTTCATCATGCCGTTTGTGA
TGGCTTCCATGTCGGCAGAATGCTTAATGAATTACAACAGTACTGCGATGAGTGGCAGG
GCGGGGCGTAA

>FKBP12-DmrA (dimerizes w FRAP-DmrC; FKBP(DmrA)-005) 321 bp
GGAGTGCAGGTGGAAACCATCTCCCCAGGAGACGGGCGCACCTTCCCCAAGCGCGGCCA
GACCTGCGTGGTGCACTACACCGGGATGCTTGAAGATGGAAAGAAATTTGATTCCTCCC
GGGACAGAAACAAGCCCTTTAAGTTTATGCTAGGCAAGCAGGAGGTGATCCGAGGCTGG
GAAGAAGGGGTTGCCCAGATGAGTGTGGGTCAGAGAGCCAAACTGACTATATCTCCAGA
TTATGCCTATGGTGCCACTGGGCACCCAGGCATCATCCCACCACATGCCACTCTCGTCT
TCGATGTGGAGCTTCTAAAACTGGAA

>FKBP12-DmrB (F36V; homodimerizes; DmrB-001) 321 bp
GGAGTGCAGGTGGAGACTATCTCCCCAGGAGACGGGCGCACCTTCCCCAAGCGCGGCCA
GACCTGCGTGGTGCACTACACCGGGATGCTTGAAGATGGAAAGAAAGTTGATTCCTCCC
GGGACAGAAACAAGCCCTTTAAGTTTATGCTAGGCAAGCAGGAGGTGATCCGAGGCTGG
GAAGAAGGGGTTGCCCAGATGAGTGTGGGTCAGAGAGCCAAACTGACTATATCTCCAGA
TTATGCCTATGGTGCCACTGGGCACCCAGGCATCATCCCACCACATGCCACTCTCGTCT
TCGATGTGGAGCTTCTAAAACTGGAA

>FRAP-DmrC (wild-type; dimerizes w FKBP-DmrA; FRB) 279 bp
ATCCTCTGGCATGAGATGTGGCATGAAGGCCTGGAAGAGGCATCTCGTTTGTACTTTGG
GGAAAGGAACGTGAAAGGCATGTTTGAGGTGCTGGAGCCCTTGCATGCTATGATGGAAC
GGGGCCCCCAGACTCTGAAGGAAACATCCTTTAATCAGGCCTATGGTCGAGATTTAATG
GAGGCCCAAGAGTGGTGCAGGAAGTACATGAAATCAGGGAATGTCAAGGACCTCACCCA
AGCCTGGGACCTCTATTATCATGTGTTCCGACGAATCTCAAAG

>FRAP-DmrC (T2098L; dimerizes w FKBP-DmrA; FRB*) 279 bp
ATCCTCTGGCATGAGATGTGGCATGAAGGCCTGGAAGAGGCATCTCGTTTGTACTTTGG
GGAAAGGAACGTGAAAGGCATGTTTGAGGTGCTGGAGCCCTTGCATGCTATGATGGAAC
GGGGCCCCCAGACTCTGAAGGAAACATCCTTTAATCAGGCCTATGGTCGAGATTTAATG
GAGGCCCAAGAGTGGTGCAGGAAGTACATGAAATCAGGGAATGTCAAGGACCTCCTCCA
AGCCTGGGACCTCTATTATCATGTGTTCCGACGAATCTCAAAG

>FKBP-DmrD (F36M;AP21998 monomerizes; DmrD-001) 321 bp

GGAGTGCAGGTGGAGACTATCTCCCCAGGAGACGGGCGCACCTTCCCCAAGCGCGGCCA
GACCTGCGTGGTGCACTACACCGGGATGCTTGAAGATGGAAAGAAAATGGATTCCTCCC
GGGACAGAAACAAGCCCTTTAAGTTTATGCTAGGCAAGCAGGAGGTGATCCGAGGCTGG
GAAGAAGGGGTTGCCCAGATGAGTGTGGGTCAGAGAGCCAAACTGACTATATCTCCAGA
TTATGCCTATGGTGCCACTGGGCACCCAGGCATCATCCCACCACATGCCACTCTCGTCT
TCGATGTGGAGCTTCTAAAACTGGAA

>FKBP-DD (F36V,L106P;Shield1 stabilizes; DD) 324 bp
ATGGGAGTGCAGGTGGAAACCATCTCCCCAGGAGACGGGCGCACCTTCCCCAAGCGCGG
CCAGACCTGTGTGGTGCACTACACCGGGATGCTTGAAGATGGAAAGAAAGTCGATTCCT
CCCGGGACAGAAACAAGCCCTTTAAGTTTATGCTAGGCAAGCAGGAGGTGATCCGAGGC
TGGGAAGAAGGGGTTGCCCAGATGAGTGTGGGTCAGAGAGCCAAACTGACTATATCTCC
AGATTATGCCTATGGTGCCACTGGGCACCCAGGCATCATCCCACCACATGCCACTCTCG
TCTTCGATGTGGAGCTTCTAAAACCGGAA

>FKBP-DD-C (E31G,F36V,R71G,K105E; Shield1 stabilizes; DD-C)
324 bp
GGAGTGCAGGTGGAAACCATCTCCCCAGGAGACGGGCGCACCTTCCCCAAGCGCGGCCA
GACCTGCGTGGTGCACTACACCGGGATGCTTGGAGATGGAAAGAAAGTTGACTCCTCCC
GGGACAGAAACAAGCCCTTTAAGTTTATGCTAGGCAAGCAGGAGGTGATCCGAGGCTGG
GAAGAAGGGGTTGCCCAGATGAGTGTGGGTCAGGGAGCCAAACTGACTATATCTCCAGA
TTATGCCTATGGTGCCACTGGGCACCCAGGCATCATCCCACCACATGCCACTCTCGTCT
TCGATGTGGAGCTTCTAGAACTGGAATAG

>GmR-aacC1 (Class 1 integron; variant -004) 543 bp
ATGTTACGCAGCAGCAACGATGTTACGCAGCAGGGCAGTCGCCCTAAAACAAAGTTAGG
TGGCTCAAGTATGGGCATCATTCGCACATGTAGGCTCGGCCCTGACCAAGTCAAATCCA
TGCGGGCTGCTCTTGATCTTTTCGGTCGTGAGTTCGGAGACGTAGCCACCTACTCCCAA
CATCAGCCGGACTCCGATTACCTCGGGAACTTGCTCCGTAGTAAGACATTCATCGCGCT
TGCTGCCTTCGACCAAGAAGCGGTTGTTGGCGCTCTCGCGGCTTACGTTCTGCCCAGGT
TTGAGCAGCCGCGTAGTGAGATCTATATCTATGATCTCGCAGTCTCCGGCGAGCACCGG
AGGCAGGGCATTGCCACCGCGCTCATCAATCTCCTCAAGCATGAGGCCAACGCGCTTGG
TGCTTATGTGATCTACGTGCAAGCAGATTACGGTGACGATCCCGCAGTGGCTCTCTATA
CAAAGTTGGGCATACGGGAAGAAGTGATGCACTTTGATATCGACCCAAGTACCGCCACC
TAA

>HygR-aph(4)-Ia (*E. coli*; variant -008) 1026 bp
ATGAAAAAGCCTGAACTCACCGCGACGTCTGTCGAGAAGTTTCTGATCGAAAAGTTCGA
CAGCGTCTCCGACCTGATGCAGCTCTCGGAGGGCGAAGAATCTCGTGCTTTCAGCTTCG
ATGTAGGAGGGCGTGGATATGTCCTGCGGGTAAATAGCTGCGCCGATGGTTTCTACAAA
GATCGTTATGTTTATCGGCACTTTGCATCGGCCGCGCTCCCGATTCCGGAAGTGCTTGA
CATTGGGGAATTCAGCGAGAGCCTGACCTATTGCATCTCCCGCCGTGCACAGGGTGTCA
CGTTGCAAGACCTGCCTGAAACCGAACTGCCCGCTGTTCTGCAGCCGGTCGCGGAGGCC
ATGGATGCGATCGCTGCGGCCGATCTTAGCCAGACGAGCGGGTTCGGCCCATTCGGACC
GCAAGGAATCGGTCAATACACTACATGGCGTGATTTCATATGCGCGATTGCTGATCCCC
ATGTGTATCACTGGCAAACTGTGATGGACGACACCGTCAGTGCGTCCGTCGCGCAGGCT
CTCGATGAGCTGATGCTTTGGGCCGAGGACTGCCCCGAAGTCCGGCACCTCGTGCACGC
GGATTTCGGCTCCAACAATGTCCTGACGGACAATGGCCGCATAACAGCGGTCATTGACT

GGAGCGAGGCGATGTTCGGGGATTCCCAATACGAGGTCGCCAACATCTTCTTCTGGAGG
CCGTGGTTGGCTTGTATGGAGCAGCAGACGCGCTACTTCGAGCGGAGGCATCCGGAGCT
TGCAGGATCGCCGCGGCTCCGGGCGTATATGCTCCGCATTGGTCTTGACCAACTCTATC
AGAGCTTGGTTGACGGCAATTTCGATGATGCAGCTTGGGCGCAGGGTCGATGCGACGCA
ATCGTCCGATCCGGAGCCGGGACTGTCGGGCGTACACAAATCGCCCGCAGAAGCGCGGC
CGTCTGGACCGATGGCTGTGTAGAAGTACTCGCCGATAGTGGAAACCGACGCCCCAGCA
CTCGTCCGAGGGCAAAGGAATAG

>KanR-aph(3')-Ia (*E. coli*; variant -009) 816 bp
ATGAGCCATATTCAACGGGAAACGTCTTGCTCGAGGCCGCGATTAAATTCCAACATGGA
TGCTGATTTATATGGGTATAAATGGGCTCGCGATAATGTCGGGCAATCAGGTGCGACAA
TCTATCGATTGTATGGGAAGCCCGATGCGCCAGAGTTGTTTCTGAAACATGGCAAAGGT
AGCGTTGCCAATGATGTTACAGATGAGATGGTCAGACTAAACTGGCTGACGGAATTTAT
GCCTCTTCCGACCATCAAGCATTTTATCCGTACTCCTGATGATGCATGGTTACTCACCA
CTGCGATCCCCGGGAAAACAGCATTCCAGGTATTAGAAGAATATCCTGATTCAGGTGAA
AATATTGTTGATGCGCTGGCAGTGTTCCTGCGCCGGTTGCATTCGATTCCTGTTTGTAA
TTGTCCTTTTAACAGCGATCGCGTATTTCGTCTCGCTCAGGCGCAATCACGAATGAATA
ACGGTTTGGTTGATGCGAGTGATTTTGATGACGAGCGTAATGGCTGGCCTGTTGAACAA
GTCTGGAAAGAAATGCATAAACTTTTGCCATTCTCACCGGATTCAGTCGTCACTCATGG
TGATTTCTCACTTGATAACCTTATTTTTGACGAGGGGAAATTAATAGGTTGTATTGATG
TTGGACGAGTCGGAATCGCAGACCGATACCAGGATCTTGCCATCCTATGGAACTGCCTC
GGTGAGTTTTCTCCTTCATTACAGAAACGGCTTTTTCAAAAATATGGTATTGATAATCC
TGATATGAATAAATTGCAGTTTCATTTGATGCTCGATGAGTTTTTCTAA

>KanR-aphA-3 (*Staphylococcus*; variant -020) 795 bp
ATGGCTAAAATGAGAATATCACCGGAATTGAAAAAACTGATCGAAAAATACCGCTGCGT
AAAAGATACGGAAGGAATGTCTCCTGCTAAGGTATATAAGCTGGTGGGAGAAAATGAAA
ACCTATATTTAAAAATGACGGACAGCCGGTATAAAGGGACCACCTATGATGTGGAACGG
GAAAAGGACATGATGCTATGGCTGGAAGGAAAGCTGCCTGTTCCAAAGGTCCTGCACTT
TGAACGGCATGATGGCTGGAGCAATCTGCTCATGAGTGAGGCCGATGGCGTCCTTTGCT
CGGAAGAGTATGAAGATGAACAAAGCCCTGAAAAGATTATCGAGCTGTATGCGGAGTGC
ATCAGGCTCTTTCACTCCATCGACATATCGGATTGTCCCTATACGAATAGCTTAGACAG
CCGCTTAGCCGAATTGGATTACTTACTGAATAACGATCTGGCCGATGTGGATTGCGAAA
ACTGGGAAGAAGACACTCCATTTAAAGATCCGCGCGAGCTGTATGATTTTTTAAAGACG
GAAAAGCCCGAAGAGGAACTTGTCTTTTCCCACGGCGACCTGGGAGACAGCAACATCTT
TGTGAAAGATGGCAAAGTAAGTGGCTTTATTGATCTTGGGAGAAGCGGCAGGGCGGACA
AGTGGTATGACATTGCCTTCTGCGTCCGGTCGATCAGGGAGGATATCGGGGAAGAACAG
TATGTCGAGCTATTTTTTGACTTACTGGGGATCAAGCCTGATTGGGAGAAAATAAAATA
TTATATTTTACTGGATGAATTGTTTTAG

>lacI (GTG START codon; variant -002) 1083 bp
GTGAAACCAGTAACGTTATACGATGTCGCAGAGTATGCCGGTGTCTCTTATCAGACCGT
TTCCCGCGTGGTGAACCAGGCCAGCCACGTTTCTGCGAAAACGCGGGAAAAAGTGGAAG
CGGCGATGGCGGAGCTGAATTACATTCCCAACCGCGTGGCACAACAACTGGCGGGCAAA
CAGTCGTTGCTGATTGGCGTTGCCACCTCCAGTCTGGCCCTGCACGCGCCGTCGCAAAT
TGTCGCGGCGATTAAATCTCGCGCCGATCAACTGGGTGCCAGCGTGGTGGTGTCGATGG
TAGAACGAAGCGGCGTCGAAGCCTGTAAAGCGGCGGTGCACAATCTTCTCGCGCAACGC
GTCAGTGGGCTGATCATTAACTATCCGCTGGATGACCAGGATGCCATTGCTGTGGAAGC

TGCCTGCACTAATGTTCCGGCGTTATTTCTTGATGTCTCTGACCAGACACCCATCAACA
GTATTATTTTCTCCCATGAAGACGGTACGCGACTGGGCGTGGAGCATCTGGTCGCATTG
GGTCACCAGCAAATCGCGCTGTTAGCGGGCCCATTAAGTTCTGTCTCGGCGCGTCTGCG
TCTGGCTGGCTGGCATAAATATCTCACTCGCAATCAAATTCAGCCGATAGCGGAACGGG
AAGGCGACTGGAGTGCCATGTCCGGTTTTCAACAAACCATGCAAATGCTGAATGAGGGC
ATCGTTCCCACTGCGATGCTGGTTGCCAACGATCAGATGGCGCTGGGCGCAATGCGCGC
CATTACCGAGTCCGGGCTGCGCGTTGGTGCGGATATCTCGGTAGTGGGATACGACGATA
CCGAAGACAGCTCATGTTATATCCCGCCGTTAACCACCATCAAACAGGATTTTCGCCTG
CTGGGGCAAACCAGCGTGGACCGCTTGCTGCAACTCTCTCAGGGCCAGGCGGTGAAGGG
CAATCAGCTGTTGCCCGTCTCACTGGTGAAAAGAAAAACCACCCTGGCGCCCAATACGC
AAACCGCCTCTCCCCGCGCGTTGGCCGATTCATTAATGCAGCTGGCACGACAGGTTTCC
CGACTGGAAAGCGGGCAGTGA

lacI-003 and -004 have a T-C transition (red highlight shows position) that results in L286S. According to Markiewicz et al. (1994) *J. Mol. Biol.* **240**:421-433, this impairs lacI function.

>Luciferase-luc (*Photinus*; wild-type variant -005 w PTS1 signal) 1653 bp
ATGGAAGACGCCAAAAACATAAAGAAAGGCCCGGCGCCATTCTATCCTCTAGAGGATGG
AACCGCTGGAGAGCAACTGCATAAGGCTATGAAGAGATACGCCCTGGTTCCTGGAACAA
TTGCTTTTACAGATGCACATATCGAGGTGAACATCACGTACGCGGAATACTTCGAAATG
TCCGTTCGGTTGGCAGAAGCTATGAAACGATATGGGCTGAATACAAATCACAGAATCGT
CGTATGCAGTGAAAACTCTCTTCAATTCTTTATGCCGGTGTTGGGCGCGTTATTTATCG
GAGTTGCAGTTGCGCCCGCGAACGACATTTATAATGAACGTGAATTGCTCAACAGTATG
AACATTTCGCAGCCTACCGTAGTGTTTGTTTCCAAAAAGGGGTTGCAAAAAATTTTGAA
CGTGCAAAAAAAATTACCAATAATCCAGAAAATTATTATCATGGATTCTAAAACGGATT
ACCAGGGATTTCAGTCGATGTACACGTTCGTCACATCTCATCTACCTCCCGGTTTTAAT
GAATACGATTTTGTACCAGAGTCCTTTGATCGTGACAAAACAATTGCACTGATAATGAA
TTCCTCTGGATCTACTGGGTTACCTAAGGGTGTGGCCCTTCCGCATAGAACTGCCTGCG
TCAGATTCTCGCATGCCAGAGATCCTATTTTTGGCAATCAAATCATTCCGGATACTGCG
ATTTTAAGTGTTGTTCCATTCCATCACGGTTTTGGAATGTTTACTACACTCGGATATTT
GATATGTGGATTTCGAGTCGTCTTAATGTATAGATTTGAAGAAGAGCTGTTTTTACGAT
CCCTTCAGGATTACAAAATTCAAAGTGCGTTGCTAGTACCAACCCTATTTTCATTCTTC
GCCAAAAGCACTCTGATTGACAAATACGATTTATCTAATTTACACGAAATTGCTTCTGG
GGGCGCACCTCTTTCGAAAGAAGTCGGGGAAGCGGTTGCAAAACGCTTCCATCTTCCAG
GGATACGACAAGGATATGGGCTCACTGAGACTACATCAGCTATTCTGATTACACCCGAG
GGGGATGATAAACCGGGCGCGGTCGGTAAAGTTGTTCCATTTTTTGAAGCGAAGGTTGT
GGATCTGGATACCGGGAAAACGCTGGGCGTTAATCAGAGAGGCGAATTATGTGTCAGAG
GACCTATGATTATGTCCGGTTATGTAAACAATCCGGAAGCGACCAACGCCTTGATTGAC
AAGGATGGATGGCTACATTCTGGAGACATAGCTTACTGGGACGAAGACGAACACTTCTT
CATAGTTGACCGCTTGAAGTCTTTAATTAAATACAAAGGATATCAGGTGGCCCCCGCTG
AATTGGAATCGATATTGTTACAACACCCCAACATCTTCGACGCGGGCGTGGCAGGTCTT
CCCGACGATGACGCCGGTGAACTTCCCGCCGCCGTTGTTGTTTTGGAGCACGGAAAGAC
GATGACGGAAAAAGAGATCGTGGATTACGTCGCCAGTCAAGTAACAACCGCGAAAAAGT
TGCGCGGAGGAGTTGTGTTTGTGGACGAAGTACCGAAAGGTCTTACCGGAAAACTCGAC
GCAAGAAAAATCAGAGAGATCCTCATAAAGGCCAAGAAGGGCGGAAAGTCCAAATTGTA
A

>Luciferase-luc+ (*Photinus*; N50D,N119G, ΔPTS1 enhanced variant -008) 1653 bp
ATGGAAGACGCCAAAAACATAAAGAAAGGCCCGGCGCCATTCTATCCGCTGGAAGATGG
AACCGCTGGAGAGCAACTGCATAAGGCTATGAAGAGATACGCCCTGGTTCCTGGAACAA
TTGCTTTTACAGATGCACATATCGAGGTGGACATCACTTACGCTGAGTACTTCGAAATG
TCCGTTCGGTTGGCAGAAGCTATGAAACGATATGGGCTGAATACAAATCACAGAATCGT
CGTATGCAGTGAAAACTCTCTTCAATTCTTTATGCCGGTGTTGGGCGCGTTATTTATCG
GAGTTGCAGTTGCGCCCGCGAACGACATTTATAATGAACGTGAATTGCTCAACAGTATG
GGCATTTCGCAGCCTACCGTGGTGTTCGTTTCCAAAAAGGGGTTGCAAAAAATTTTGAA
CGTGCAAAAAAAGCTCCCAATCATCCAAAAAATTATTATCATGGATTCTAAAACGGATT
ACCAGGGATTTCAGTCGATGTACACGTTCGTCACATCTCATCTACCTCCCGGTTTTAAT
GAATACGATTTTGTGCCAGAGTCCTTCGATAGGGACAAGACAATTGCACTGATCATGAA
CTCCTCTGGATCTACTGGTCTGCCTAAAGGTGTCGCTCTGCCTCATAGAACTGCCTGCG
TGAGATTCTCGCATGCCAGAGATCCTATTTTTGGCAATCAAATCATTCCGGATACTGCG
ATTTTAAGTGTTGTTCCATTCCATCACGGTTTTGGAATGTTTACTACACTCGGATATTT
GATATGTGGATTTCGAGTCGTCTTAATGTATAGATTTGAAGAAGAGCTGTTTCTGAGGA
GCCTTCAGGATTACAAGATTCAAAGTGCGCTGCTGGTGCCAACCCTATTCTCCTTCTTC
GCCAAAAGCACTCTGATTGACAAATACGATTTATCTAATTTACACGAAATTGCTTCTGG
TGGCGCTCCCCTCTCTAAGGAAGTCGGGGAAGCGGTTGCCAAGAGGTTCCATCTGCCAG
GTATCAGGCAAGGATATGGGCTCACTGAGACTACATCAGCTATTCTGATTACACCCGAG
GGGGATGATAAACCGGGCGCGGTCGGTAAAGTTGTTCCATTTTTTGAAGCGAAGGTTGT
GGATCTGGATACCGGGAAAACGCTGGGCGTTAATCAAAGAGGCGAACTGTGTGTGAGAG
GTCCTATGATTATGTCCGGTTATGTAAACAATCCGGAAGCGACCAACGCCTTGATTGAC
AAGGATGGATGGCTACATTCTGGAGACATAGCTTACTGGGACGAAGACGAACACTTCTT
CATCGTTGACCGCCTGAAGTCTCTGATTAAGTACAAAGGCTATCAGGTGGCTCCCGCTG
AATTGGAATCCATCTTGCTCCAACACCCCAACATCTTCGACGCAGGTGTCGCAGGTCTT
CCCGACGATGACGCCGGTGAACTTCCCGCCGCCGTTGTTGTTTTGGAGCACGGAAAGAC
GATGACGGAAAAAGAGATCGTGGATTACGTCGCCAGTCAAGTAACAACCGCGAAAAAGT
TGCGCGGAGGAGTTGTGTTTGTGGACGAAGTACCGAAAGGTCTTACCGGAAAACTCGAC
GCAAGAAAAATCAGAGAGATCCTCATAAAGGCCAAGAAGGGCGGAAAGATCGCCGTGTA
A

>Luciferase-luc2 (N50D,N119G, ΔPTS1 enhanced synthetic variant -006) 1650 bp
ATGGAAGATGCCAAAAACATTAAGAAGGGCCCAGCGCCATTCTACCCACTCGAAGACGG
GACCGCCGGCGAGCAGCTGCACAAAGCCATGAAGCGCTACGCCCTGGTGCCCGGCACCA
TCGCCTTTACCGACGCACATATCGAGGTGGACATTACCTACGCCGAGTACTTCGAGATG
AGCGTTCGGCTGGCAGAAGCTATGAAGCGCTATGGGCTGAATACAAACCATCGGATCGT
GGTGTGCAGCGAGAATAGCTTGCAGTTCTTCATGCCCGTGTTGGGTGCCCTGTTCATCG
GTGTGGCTGTGGCCCCAGCTAACGACATCTACAACGAGCGCGAGCTGCTGAACAGCATG
GGCATCAGCCAGCCCACCGTCGTATTCGTGAGCAAGAAAGGGCTGCAAAAGATCCTCAA
CGTGCAAAAGAAGCTACCGATCATACAAAAGATCATCATCATGGATAGCAAGACCGACT
ACCAGGGCTTCCAAAGCATGTACACCTTCGTGACTTCCCATTTGCCACCCGGCTTCAAC
GAGTACGACTTCGTGCCCGAGAGCTTCGACCGGGACAAAACCATCGCCCTGATCATGAA
CAGTAGTGGCAGTACCGGATTGCCCAAGGGCGTAGCCCTACCGCACCGCACCGCTTGTG
TCCGATTCAGTCATGCCCGCGACCCCATCTTCGGCAACCAGATCATCCCCGACACCGCT
ATCCTCAGCGTGGTGCCATTTCACCACGGCTTCGGCATGTTCACCACGCTGGGCTACTT
GATCTGCGGCTTTCGGGTCGTGCTCATGTACCGCTTCGAGGAGGAGCTATTCTTGCGCA

GCTTGCAAGACTATAAGATTCAATCTGCCCTGCTGGTGCCCACACTATTTAGCTTCTTC
GCTAAGAGCACTCTCATCGACAAGTACGACCTAAGCAACTTGCACGAGATCGCCAGCGG
CGGGGCGCCGCTCAGCAAGGAGGTAGGTGAGGCCGTGGCCAAACGCTTCCACCTACCAG
GCATCCGCCAGGGCTACGGCCTGACAGAAACAACCAGCGCCATTCTGATCACCCCCGAA
GGGGACGACAAGCCTGGCGCAGTAGGCAAGGTGGTGCCCTTCTTCGAGGCTAAGGTGGT
GGACTTGGACACCGGTAAGACACTGGGTGTGAACCAGCGCGGCGAGCTGTGCGTCCGTG
GCCCCATGATCATGAGCGGCTACGTTAACAACCCCGAGGCTACAAACGCTCTCATCGAC
AAGGACGGCTGGCTGCACAGCGGCGACATCGCCTACTGGGACGAGGACGAGCACTTCTT
CATCGTGGACCGGCTGAAGAGCCTGATCAAATACAAGGGCTACCAGGTAGCCCCAGCCG
AACTGGAGAGCATCCTGCTGCAACACCCCAACATCTTCGACGCCGGGGTCGCCGGCCTG
CCCGACGACGATGCCGGCGAGCTGCCCGCCGCAGTCGTCGTGCTGGAACACGGTAAAAC
CATGACCGAGAAGGAGATCGTGGACTATGTGGCCAGCCAGGTTACAACCGCCAAGAAGC
TGCGCGGTGGTGTTGTGTTCGTGGACGAGGTGCCTAAAGGACTGACCGGCAAGTTGGAC
GCCCGCAAGATCCGCGAGATTCTCATTAAGGCCAAGAAGGGCGGCAAGATCGCCGTG

Luciferase-002 and -005 are wild-type. Luciferase -007 and -008 are enhanced luciferase. Luciferase-001, -003, -004 and -006 are synthetic enhanced.

>MBP (variant -004) 1101 bp
ATGAAAATCGAAGAAGGTAAACTGGTAATCTGGATTAACGGCGATAAAGGCTATAACGG
TCTCGCTGAAGTCGGTAAGAAATTCGAGAAAGATACCGGAATTAAAGTCACCGTTGAGC
ATCCGGATAAACTGGAAGAGAAATTCCCACAGGTTGCGGCAACTGGCGATGGCCCTGAC
ATTATCTTCTGGGCACACGACCGCTTTGGTGGCTACGCTCAATCTGGCCTGTTGGCTGA
AATCACCCCGGACAAAGCGTTCCAGGACAAGCTGTATCCGTTTACCTGGGATGCCGTAC
GTTACAACGGCAAGCTGATTGCTTACCCGATCGCTGTTGAAGCGTTATCGCTGATTTAT
AACAAAGATCTGCTGCCGAACCCGCCAAAAACCTGGGAAGAGATCCCGGCGCTGGATAA
AGAACTGAAAGCGAAAGGTAAGAGCGCGCTGATGTTCAACCTGCAAGAACCGTACTTCA
CCTGGCCGCTGATTGCTGCTGACGGGGGTTATGCGTTCAAGTATGAAAACGGCAAGTAC
GACATTAAAGACGTGGGCGTGGATAACGCTGGCGCGAAAGCGGGTCTGACCTTCCTGGT
TGACCTGATTAAAAACAAACACATGAATGCAGACACCGATTACTCCATCGCAGAAGCTG
CCTTTAATAAAGGCGAAACAGCGATGACCATCAACGGCCCGTGGGCATGGTCCAACATC
GACACCAGCAAAGTGAATTATGGTGTAACGGTACTGCCGACCTTCAAGGGTCAACCATC
CAAACCGTTCGTTGGCGTGCTGAGCGCAGGTATTAACGCCGCCAGTCCGAACAAAGAGC
TGGCAAAAGAGTTCCTCGAAAACTATCTGCTGACTGATGAAGGTCTGGAAGCGGTTAAT
AAAGACAAACCGCTGGGTGCCGTAGCGCTGAAGTCTTACGAGGAAGAGTTGGCGAAAGA
TCCACGTATTGCCGCCACCATGGAAAACGCCCAGAAAGGTGAAATCATGCCGAACATCC
CGCAGATGTCCGCTTTCTGGTATGCCGTGCGTACTGCGGTGATCAACGCCGCCAGCGGT
CGTCAGACTGTCGATGAAGCCCTGAAAGACGCGCAGACT

>nos-nptII (KanR variant -016) 822 bp
ATGGCAATTACCTTATCCGCAACTTCTTTACCTATTTCCGCCCGGATCCGGGCAGGTTC
TCCGGCCGCTTGGGTGGAGAGGCTATTCGGCTATGACTGGGCACAACAGACAATCGGCT
GCTCTGATGCCGCCGTGTTCCGGCTGTCAGCGCAGGGGCGCCCGGTTCTTTTTGTCAAG
ACCGACCTGTCCGGTGCCCTGAATGAACTGCAGGACGAGGCAGCGCGGCTATCGTGGCT
GGCCACGACGGGCGTTCCTTGCGCAGCTGTGCTCGACGTTGTCACTGAAGCGGGAAGGG
ACTGGCTGCTATTGGGCGAAGTGCCGGGGCAGGATCTCCTGTCATCTCACCTTGCTCCT
GCCGAGAAAGTATCCATCATGGCTGATGCAATGCGGCGGCTGCATACGCTTGATCCGGC
TACCTGCCCATTCGACCACCAAGCGAAACATCGCATCGAGCGAGCACGTACTCGGATGG

AAGCCGGTCTTGTCGATCAGGATGATCTGGACGAAGAGCATCAGGGGCTCGCGCCAGCC
GAACTGTTCGCCAGGCTCAAGGCGCGCATGCCCGACGGCGAGGATCTCGTCGTGACCCA
TGGCGATGCCTGCTTGCCGAATATCATGGTGGAAAATGGCCGCTTTTCTGGATTCATCG
ACTGTGGCCGGCTGGGTGTGGCGGACCGCTATCAGGACATAGCGTTGGCTACCCGTGAT
ATTGCTGAAGAGCTTGGCGGCGAATGGGCTGACCGCTTCCTCGTGCTTTACGGTATCGC
CGCTCCCGATTCGCAGCGCATCGCCTTCTATCGCCTTCTTGACGAGTTCTTCTGA

>NeoR-KanR-nptII (variant -002) 795 bp
ATGATTGAACAAGATGGATTGCACGCAGGTTCTCCGGCCGCTTGGGTGGAGAGGCTATT
CGGCTATGACTGGGCACAACAGACAATCGGCTGCTCTGATGCCGCCGTGTTCCGGCTGT
CAGCGCAGGGGCGCCCGGTTCTTTTTGTCAAGACCGACCTGTCCGGTGCCCTGAATGAA
CTGCAGGACGAGGCAGCGCGGCTATCGTGGCTGGCCACGACGGGCGTTCCTTGCGCAGC
TGTGCTCGACGTTGTCACTGAAGCGGGAAGGGACTGGCTGCTATTGGGCGAAGTGCCGG
GGCAGGATCTCCTGTCATCTCACCTTGCTCCTGCCGAGAAAGTATCCATCATGGCTGAT
GCAATGCGGCGGCTGCATACGCTTGATCCGGCTACCTGCCCATTCGACCACCAAGCGAA
ACATCGCATCGAGCGAGCACGTACTCGGATGGAAGCCGGTCTTGTCGATCAGGATGATC
TGGACGAAGAGCATCAGGGGCTCGCGCCAGCCGAACTGTTCGCCAGGCTCAAGGCGCGC
ATGCCCGACGGCGAGGATCTCGTCGTGACCCATGGCGATGCCTGCTTGCCGAATATCAT
GGTGGAAAATGGCCGCTTTTCTGGATTCATCGACTGTGGCCGGCTGGGTGTGGCGGACC
GCTATCAGGACATAGCGTTGGCTACCCGTGATATTGCTGAAGAGCTTGGCGGCGAATGG
GCTGACCGCTTCCTCGTGCTTTACGGTATCGCCGCTCCCGATTCGCAGCGCATCGCCTT
CTATCGCCTTCTTGACGAGTTCTTCTGA

>PuroR-pac (*Streptomyces*; variant -006) 600 bp
ATGACCGAGTACAAGCCCACGGTGCGCCTCGCCACCCGCGACGACGTCCCCCGGGCCGT
ACGCACCCTCGCCGCCGCGTTCGCCGACTACCCCGCCACGCGCCACACCGTCGACCCGG
ACCGCCACATCGAGCGGGTCACCGAGCTGCAAGAACTCTTCCTCACGCGCGTCGGGCTC
GACATCGGCAAGGTGTGGGTCGCGGACGACGGCGCCGCGGTGGCGGTCTGGACCACGCC
GGAGAGCGTCGAAGCGGGGGCGGTGTTCGCCGAGATCGGCCCGCGCATGGCCGAGTTGA
GCGGTTCCCGGCTGGCCGCGCAGCAACAGATGGAAGGCCTCCTGGCGCCGCACCGGCCC
AAGGAGCCCGCGTGGTTCCTGGCCACCGTCGGCGTCTCGCCCGACCACCAGGGCAAGGG
TCTGGGCAGCGCCGTCGTGCTCCCCGGAGTGGAGGCGGCCGAGCGCGCCGGGGTGCCCG
CCTTCCTGGAGACCTCCGCGCCCCGCAACCTCCCCTTCTACGAGCGGCTCGGCTTCACC
GTCACCGCCGACGTCGAGGTGCCCGAAGGACCGCGCACCTGGTGCATGACCCGCAAGCC
CGGTGCCTGA

>TcR-tetA1 (Tn10; variant -001) 1206 bp
ATGAATAGTTCGACAAAGATCGCATTGGTAATTACGTTACTCGATGCCATGGGGATTGG
CCTTATCATGCCAGTCTTGCCAACGTTATTACGTGAATTTATTGCTTCGGAAGATATCG
CTAACCACTTTGGCGTATTGCTTGCACTTTATGCGTTAATGCAGGTTATCTTTGCTCCT
TGGCTTGGAAAAATGTCTGACCGATTTGGTCGGCGCCCAGTGCTGTTGTTGTCATTAAT
AGGCGCATCGCTGGATTACTTATTGCTGGCTTTTTCAAGTGCGCTTTGGATGCTGTATT
TAGGCCGTTTGCTTTCAGGGATCACAGGAGCTACTGGGGCTGTCGCGGCATCGGTCATT
GCCGATACCACCTCAGCTTCTCAACGCGTGAAGTGGTTCGGTTGGTTAGGGGCAAGTTT
TGGGCTTGGTTTAATAGCGGGGCCTATTATTGGTGGTTTTGCAGGAGAGATTTCACCGC
ATAGTCCCTTTTTTATCGCTGCGTTGCTAAATATTGTCACTTTCCTTGTGGTTATGTTT
TGGTTCCGTGAAACCAAAAATACACGTGATAATACAGATACCGAAGTAGGGGTTGAGAC
GCAATCAAATTCGGTGTACATCACTTTATTTAAAACGATGCCCATTTTGTTGATTATTT

ATTTTTCAGCGCAATTGATAGGCCAAATTCCCGCAACGGTGTGGGTGCTATTTACCGAA
AATCGTTTTGGATGGAATAGCATGATGGTTGGCTTTTCATTAGCGGGTCTTGGTCTTTT
ACACTCAGTATTCCAAGCCTTTGTGGCAGGAAGAATAGCCACTAAATGGGGCGAAAAAA
CGGCAGTACTGCTCGAATTTATTGCAGATAGTAGTGCATTTGCCTTTTTAGCGTTTATA
TCTGAAGGTTGGTTAGATTTCCCTGTTTTAATTTTATTGGCTGGTGGTGGGATCGCTTT
ACCTGCATTACAGGGAGTGATGTCTATCCAAACAAAGAGTCATGAGCAAGGTGCTTTAC
AGGGATTATTGGTGAGCCTTACCAATGCAACCGGTGTTATTGGCCCATTACTGTTTACT
GTTATTTATAATCATTCACTACCAATTTGGGATGGCTGGATTTGGATTATTGGTTTAGC
GTTTTACTGTATTATTATCCTGCTATCAATGACCTTCATGTTGACCCCTCAAGCTCAGG
GGAGTAAACAGGAGACAAGTGCTTAG

>TcR-tetA2 (IncP-α plasmids; ATG START codon; variant -003) 1200 bp
ATGAAACCCAACATACCCCTGATCGTAATTCTGAGCACTGTCGCGCTCGACGCTGTCGG
CATCGGCCTGATTATGCCGGTGCTGCCGGGCCTCCTGCGCGATCTGGTTCACTCGAACG
ACGTCACCGCCCACTATGGCATTCTGCTGGCGCTGTATGCGTTGGTGCAATTTGCCTGC
GCACCTGTGCTGGGCGCGCTGTCGGATCGTTTCGGGCGGCGGCCAATCTTGCTCGTCTC
GCTGGCCGGCGCCACTGTCGACTACGCCATCATGGCGACAGCGCCTTTCCTTTGGGTTC
TCTATATCGGGCGGATCGTGGCCGGCATCACCGGGGCGACTGGGGCGGTAGCCGGCGCT
TATATTGCCGATATCACTGATGGCGATGAGCGCGCGCGGCACTTCGGCTTCATGAGCGC
CTGTTTCGGGTTCGGGATGGTCGCGGGACCTGTGCTCGGTGGGCTGATGGGCGGTTTCT
CCCCCCACGCTCCGTTCTTCGCCGCGGCAGCCTTGAACGGCCTCAATTTCCTGACGGGC
TGTTTCCTTTTGCCGGAGTCGCACAAAGGCGAACGCCGGCCGTTACGCCGGGAGGCTCT
CAACCCGCTCGCTTCGTTCCGGTGGGCCCGGGGCATGACCGTCGTCGCCGCCCTGATGG
CGGTCTTCTTCATCATGCAACTTGTCGGACAGGTGCCGGCCGCGCTTTGGGTCATTTTC
GGCGAGGATCGCTTTCACTGGGACGCGACCACGATCGGCATTTCGCTTGCCGCATTTGG
CATTCTGCATTCACTCGCCCAGGCAATGATCACCGGCCCTGTAGCCGCCCGGCTCGGCG
AAAGGCGGGCACTCATGCTCGGAATGATTGCCGACGGCACAGGCTACATCCTGCTTGCC
TTCGCGACACGGGGATGGATGGCGTTCCCGATCATGGTCCTGCTTGCTTCGGGTGGCAT
CGGAATGCCGGCGCTGCAAGCAATGTTGTCCAGGCAGGTGGATGAGGAACGTCAGGGGC
AGCTGCAAGGCTCACTGGCGGCGCTCACCAGCCTGACCTCGATCGTCGGACCCCTCCTC
TTCACGGCGATCTATGCGGCTTCTATAACAACGTGGAACGGGTGGGCATGGATTGCAGG
CGCTGCCCTCTACTTGCTCTGCCTGCCGGCGCTGCGTCGCGGGCTTTGGAGCGGCGCAG
GGCAACGAGCCGATCGCTGA

>TcR-tetC (pSC101; variant -005) 1191 bp
ATGAAATCTAACAATGCGCTCATCGTCATCCTCGGCACCGTCACCCTGGATGCTGTAGG
CATAGGCTTGGTTATGCCGGTACTGCCGGGCCTCTTGCGGGATATCGTCCATTCCGACA
GCATCGCCAGTCACTATGGCGTGCTGCTAGCGCTATATGCGTTGATGCAATTTCTATGC
GCACCCGTTCTCGGAGCACTGTCCGACCGCTTTGGCCGCCGCCCAGTCCTGCTCGCTTC
GCTACTTGGAGCCACTATCGACTACGCGATCATGGCGACCACACCCGTCCTGTGGATCC
TCTACGCCGGACGCATCGTGGCCGGCATCACCGGCGCCACAGGTGCGGTTGCTGGCGCC
TATATCGCCGACATCACCGATGGGGAAGATCGGGCTCGCCACTTCGGGCTCATGAGCGC
TTGTTTCGGCGTGGGTATGGTGGCAGGCCCCGTGGCCGGGGGACTGTTGGGCGCCATCT
CCTTGCATGCACCATTCCTTGCGGCGGCGGTGCTCAACGGCCTCAACCTACTACTGGGC
TGCTTCCTAATGCAGGAGTCGCATAAGGGAGAGCGTCGACCGATGCCCTTGAGAGCCTT
CAACCCAGTCAGCTCCTTCCGGTGGGCGCGGGGCATGACTATCGTCGCCGCACTTATGA
CTGTCTTCTTTATCATGCAACTCGTAGGACAGGTGCCGGCAGCGCTCTGGGTCATTTTC

GGCGAGGACCGCTTTCGCTGGAGCGCGACGATGATCGGCCTGTCGCTTGCGGTATTCGG
AATCTTGCACGCCCTCGCTCAAGCCTTCGTCACTGGTCCCGCCACCAAACGTTTCGGCG
AGAAGCAGGCCATTATCGCCGGCATGGCGGCCGACGCGCTGGGCTACGTCTTGCTGGCG
TTCGCGACGCGAGGCTGGATGGCCTTCCCCATTATGATTCTTCTCGCTTCCGGCGGCAT
CGGGATGCCCGCGTTGCAGGCCATGCTGTCCAGGCAGGTAGATGACGACCATCAGGGAC
AGCTTCAAGGATCGCTCGCGGCTCTTACCAGCCTAACTTCGATCATTGGACCGCTGATC
GTCACGGCGATTTATGCCGCCTCGGCGAGCACATGGAACGGGTTGGCATGGATTGTAGG
CGCCGCCCTATACCTTGTCTGCCTCCCCGCGTTGCGTCGCGGTGCATGGAGCCGGGCCA
CCTCGACCTGA

>TcR-tetL (*Bacillus*; GTG START codon; variant -006) 1377 bp
GTGAATACATCCTATTCACAATCGAATTTACGACACAACCAAATTTTAATTTGGCTTTG
CATTTTATCTTTTTTTAGCGTATTAAATGAAATGGTTTTGAACGTCTCATTACCTGATA
TTGCAAATGATTTTAATAAACCACCTGCGAGTACAAACTGGGTGAACACAGCCTTTATG
TTAACCTTTTCCATTGGAACAGCTGTATATGGAAAGCTATCTGATCAATTAGGCATCAA
AAGGTTACTCCTATTTGGAATTATAATAAATTGTTTCGGGTCGGTAATTGGGTTTGTTG
GCCATTCTTTCTTTTCCTTACTTATTATGGCTCGTTTTATTCAAGGGGCTGGTGCAGCT
GCATTTCCAGCACTCGTAATGGTTGTAGTTGCGCGCTATATTCCAAAGGAAAATAGGGG
TAAAGCATTTGGTCTTATTGGATCGATAGTAGCCATGGGAGAAGGAGTCGGTCCAGCGA
TTGGTGGAATGATAGCCCATTATATTCATTGGTCCTATCTTCTACTCATTCCTATGATA
ACAATTATCACTGTTCCGTTTCTTATGAAATTATTAAAGAAAGAAGTAAGGATAAAAGG
TCATTTTGATATCAAAGGAATTATACTAATGTCTGTAGGCATTGTATTTTTTATGTTGT
TTACAACATCATATAGCATTTCTTTTCTTATCGTTAGCGTGCTGTCATTCCTGATATTT
GTAAAACATATCAGGAAAGTAACAGATCCTTTTGTTGATCCCGGATTAGGGAAAAATAT
ACCTTTTATGATTGGAGTTCTTTGTGGGGGAATTATATTTGGAACAGTAGCAGGGTTTG
TCTCTATGGTTCCTTATATGATGAAAGATGTTCACCAGCTAAGTACTGCCGAAATCGGA
AGTGTAATTATTTTCCCTGGAACAATGAGTGTCATTATTTTCGGCTACATTGGTGGGAT
ACTTGTTGATAGAAGAGGTCCTTTATACGTGTTAAACATCGGAGTTACATTTCTTTCTG
TTAGCTTTTTAACTGCTTCCTTTCTTTTAGAAACAACATCATGGTTCATGACAATTATA
ATCGTATTTGTTTTAGGTGGGCTTTCGTTCACCAAAACAGTTATATCAACAATTGTTTC
AAGTAGCTTGAAACAGCAGGAAGCTGGTGCTGGAATGAGTTTGCTTAACTTTACCAGCT
TTTTATCAGAGGGAACAGGTATTGCAATTGTAGGTGGTTTATTATCCATACCCTTACTT
GATCAAAGGTTGTTACCTATGGAAGTTGATCAGTCAACTTATCTGTATAGTAATTTGTT
ATTACTTTTTTCAGGAATCATTGTCATTAGTTGGCTGGTTACCTTGAATGTATATAAAC
ATTCTCAAAGGGATTTCTAA

>TetR (*Pseudomonas* RP4; variant -001) 651 bp
ATGACAAAGTTGCAGCCGAATACAGTGATCCGTGCCGCCCTGGACCTGTTGAACGAGGT
CGGCGTAGACGGTCTGACGACACGCAAACTGGCGGAACGGTTGGGGGTTCAGCAGCCGG
CGCTTTACTGGCACTTCAGGAACAAGCGGGCGCTGCTCGACGCACTGGCCGAAGCCATG
CTGGCGGAGAATCATACGCATTCGGTGCCGAGAGCCGACGACGACTGGCGCTCATTTCT
GATCGGGAATGCCCGCAGCTTCAGGCAGGCGCTGCTCGCCTACCGCGATGGCGCGCGCA
TCCATGCCGGCACGCGACCGGGCGCACCGCAGATGGAAACGGCCGACGCGCAGCTTCGC
TTCCTCTGCGAGGCGGGTTTTTCGGCCGGGGACGCCGTCAATGCGCTGATGACAATCAG
CTACTTCACTGTTGGGGCCGTGCTTGAGGAGCAGGCCGGCGACAGCGATGCCGGCGAGC
GCGGCGGCACCGTTGAACAGGCTCCGCTCTCGCCGCTGTTGCGGGCCGCGATAGACGCC
TTCGACGAAGCCGGTCCGGACGCAGCGTTCGAGCAGGGACTCGCGGTGATTGTCGATGG

ATTGGCGAAAAGGAGGCTCGTTGTCAGGAACGTTGAAGGACCGAGAAAGGGTGACGATT
GA

>TetR (Tn10; variant -004) 624 bp
ATGTCTAGATTAGATAAAAGTAAAGTGATTAACAGCGCATTAGAGCTGCTTAATGAGGT
CGGAATCGAAGGTTTAACAACCCGTAAACTCGCCCAGAAGCTAGGTGTAGAGCAGCCTA
CATTGTATTGGCATGTAAAAAATAAGCGGGCTTTGCTCGACGCCTTAGCCATTGAGATG
TTAGATAGGCACCATACTCACTTTTGCCCTTTAGAAGGGGAAAGCTGGCAAGATTTTTT
ACGTAATAACGCTAAAAGTTTTAGATGTGCTTTACTAAGTCATCGCGATGGAGCAAAAG
TACATTTAGGTACACGGCCTACAGAAAAACAGTATGAAACTCTCGAAAATCAATTAGCC
TTTTTATGCCAACAAGGTTTTTCACTAGAGAATGCATTATATGCACTCAGCGCTGTGGG
GCATTTTACTTTAGGTTGCGTATTGGAAGATCAAGAGCATCAAGTCGCTAAAGAAGAAA
GGGAAACACCTACTACTGATAGTATGCCGCCATTATTACGACAAGCTATCGAATTATTT
GATCACCAAGGTGCAGAGCCAGCCTTCTTATTCGGCCTTGAATTGATCATATGCGGATT
AGAAAAACAACTTAAATGTGAAAGTGGGTCTTAA

TetR-003 was mis-annotated and is actually a unique variant of TcR
